# Supplementary material for: Charting organellar importomes by quantitative mass spectrometry
Source: Nat Commun. 2017 May 9;8:15272. doi: 10.1038/ncomms15272 (PMC5436138; doi:10.1038/ncomms15272)
Supplement: Supplementary Information — Supplementary Figures and Supplementary References [file ncomms15272-s1.pdf]

Supplementary Figure 1

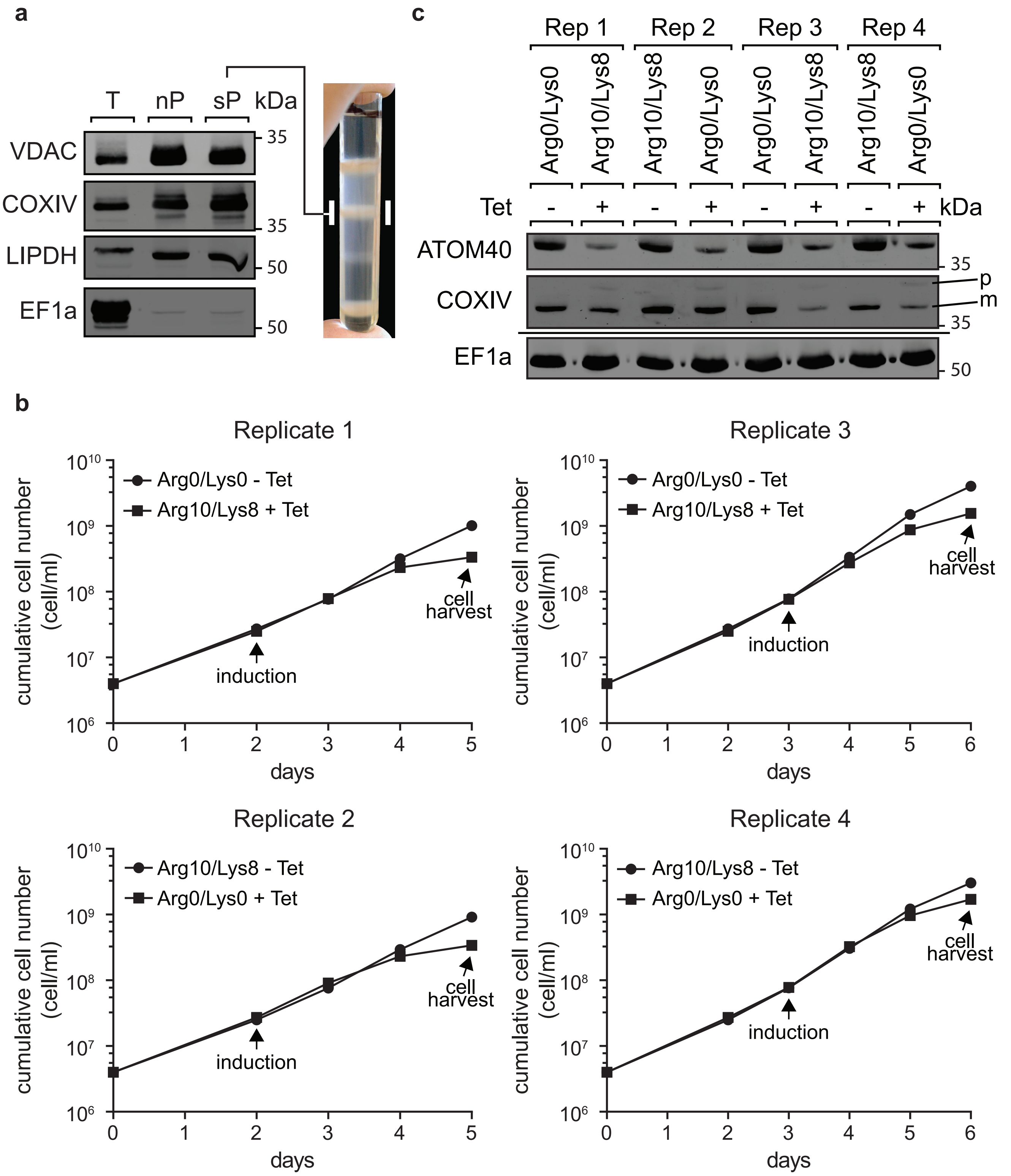

Supplementary Figure 1. Downscaling of mitochondrial preparation, growth curves, and confirmation of ATOM40 knockdown in SILAC experiments. **a.** Immunoblot analysis for mitochondrial proteins (VDAC, COXIV and LIPDH) and cytosolic EF1a in whole cell extracts (T) and gradient-purified mitochondria from a standard size preparation (nP) and a small scale preparation (sP). Equal amounts of proteins were loaded in all three lanes. Mitochondria isolated under isotonic conditions and purified on Nycodenz gradients are usually prepared from a minimum of approximately 1011 cells. To reduce the amount of starting material in SILAC experiments, the protocol has been adapted allowing for the isolation of mitochondria of equal purity from approximately 10^{10} cells. **b.** Growth curves of the eight ATOM40-RNAi cell cultures grown in light (Arg0/Lys0) or heavy (Arg10/Lys8) amino acid-containing medium as indicated. Cultures were grown for two or three days in the respective SILAC medium prior to induction of ATOM40-RNAi by tetracycline (+Tet). RNAi was induced for 3 days prior to cell harvest and isolation of mitochondria. As control, SILAC-labeled ATOM40-RNAi cells were cultured without RNAi induction by tetracycline (-Tet). In total, four independent biological replicates were prepared for SILAC-MS analyses including a label-switch. **c.** Immunoblot analysis for confirmation of ATOM40 knockdown in SILAC experiments. Total cell extracts were individually prepared from SILAC-labeled uninduced (-Tet) and induced (+Tet) ATOM40-RNAi cells for each replicate depicted in (b). The blot was probed for the RNAi target ATOM40 and the inner mitochondrial membrane protein COXIV for all replicates. Import of COXIV depends on ATOM40 leading to the accumulation of its cytosolic precursor form (p) and depletion of its mitochondrial mature form (m) following ATOM40 knockdown. The cytosolic protein EF1a serves as loading control.

Supplementary Figure 2

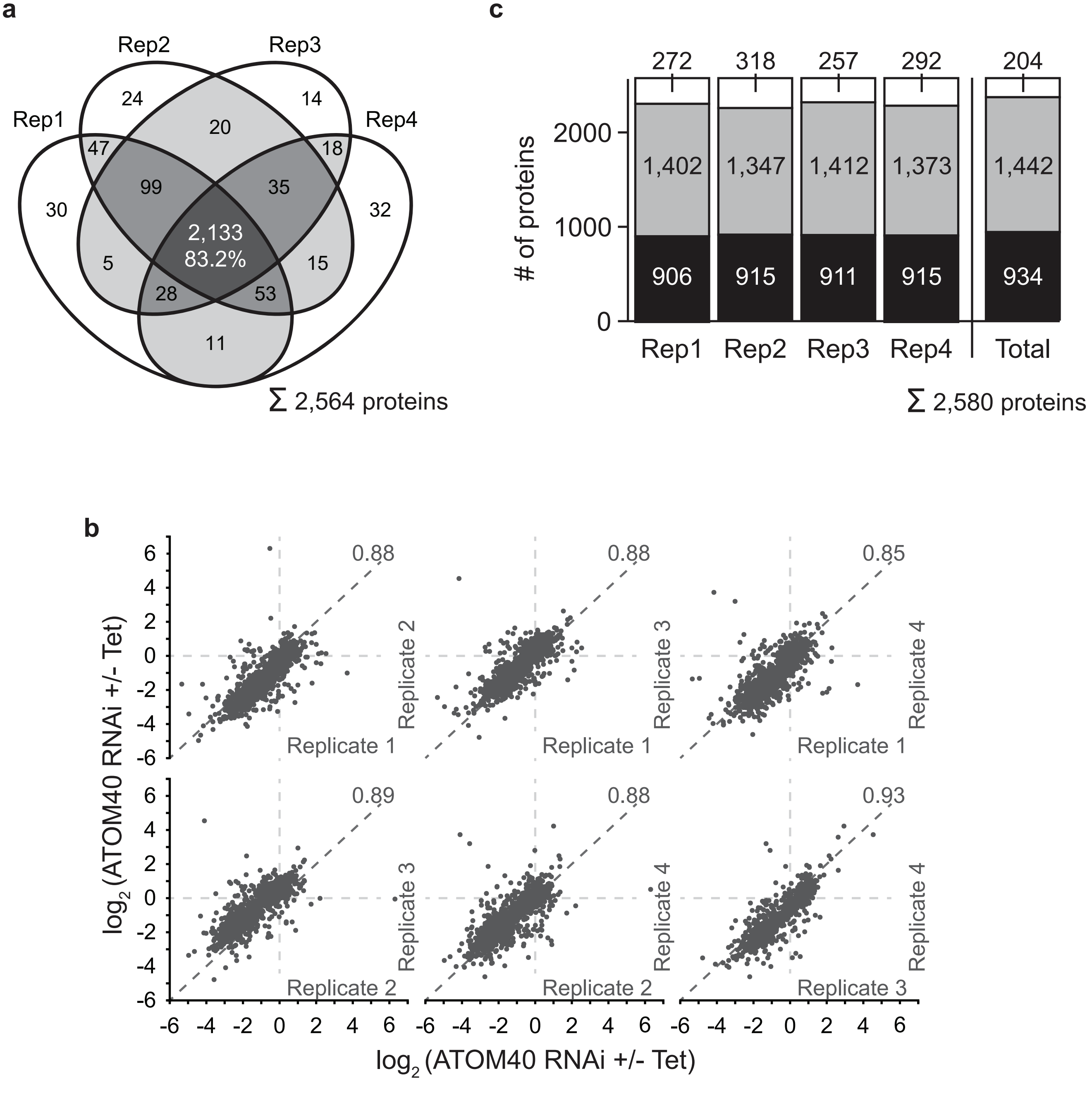

Supplementary Figure 2. Reproducibility of SILAC-based proteome data obtained from ATOM40-RNAi experiments following isolation of mitochondria by density gradient centrifugation. **a.** Overlap of proteins with at least one SILAC ratio in gradient-purified mitochondria isolated from tetracycline-induced and uninduced ATOM40-RNAi cells in four biological replicates. Rep, replicate. **b.** Multiscatter plot of protein abundance ratios determined in individual replicates. SILAC ratios of proteins detected in gradient-purified mitochondria of induced (+Tet) *versus* uninduced (-Tet) ATOM40-RNAi cells were calculated for each replicate by MaxQuant, log_2-transformed and plotted against each other. Values indicate the Pearson correlation coefficient between replicates. **c.** Number of proteins identified and quantified in individual replicates and in total. Black box, quantified proteins of the mitochondrial reference proteome defined in this study; gray box, other quantified proteins; white box, proteins not fulfilling our criteria for quantification and, thus, not taken into account for further data analysis. Rep, replicate.

Supplementary Figure 3

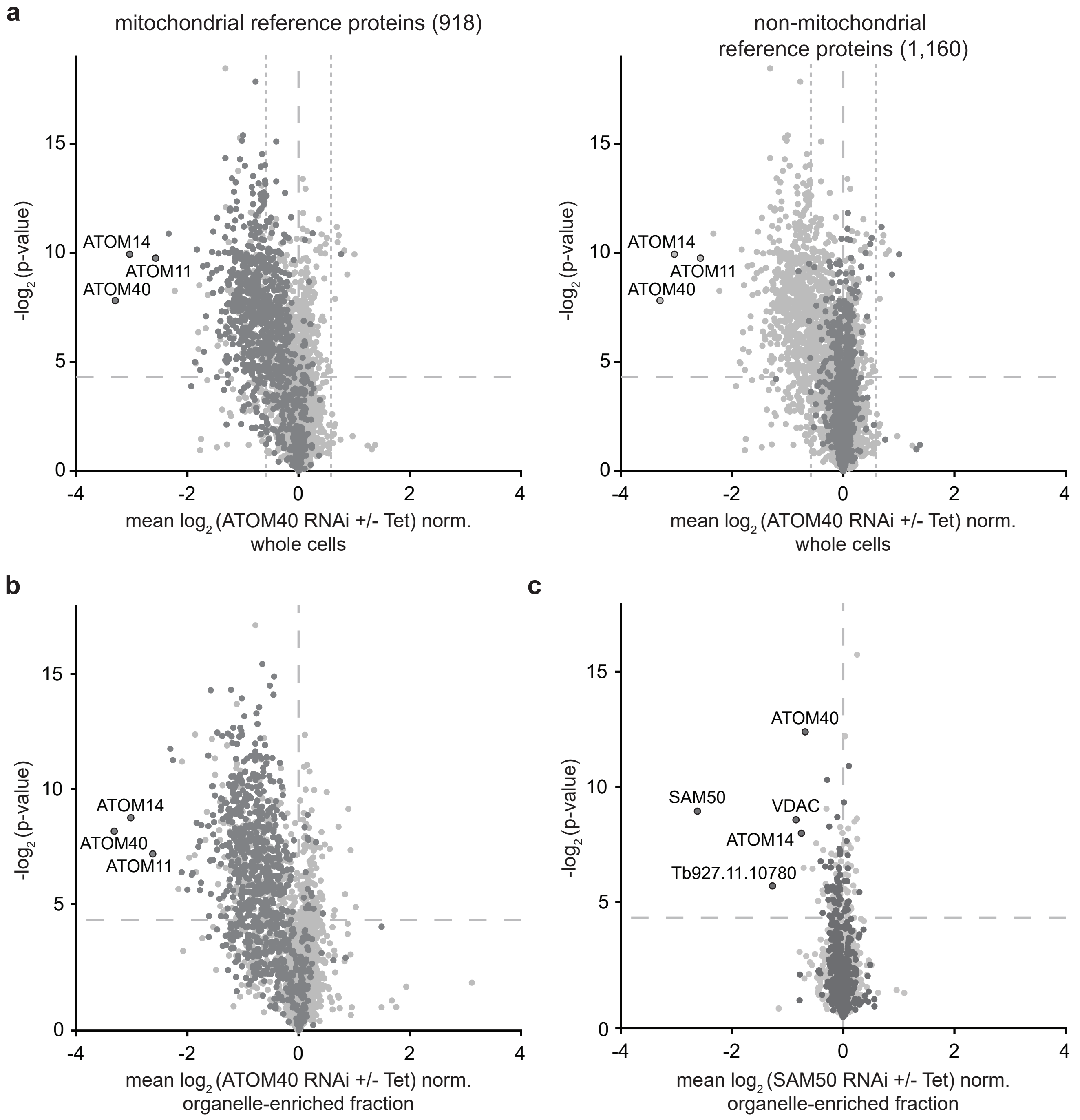

Supplementary Figure 3. Detection of RNAi-induced changes in protein abundances from whole cell extracts and organelle-enriched fractions. Changes in the proteome were determined by SILAC-MS in whole cell extracts (**a**) or organelle-enriched fractions (**b, c**) prepared from induced (+Tet) and uninduced (-Tet) ATOM40- (**a, b**) or SAM50- (**c**) RNAi cells. Mean $\log_2$ values of normalized (norm.) SILAC ratios of proteins quantified in RNAi knockdown experiments are plotted against their respective p-values (-$\log_2$ values; two-sided Student's t-test; n=3). Proteins of the mitochondrial (left plot in **a**; **b** and **c**) or the non-mitochondrial reference proteome (right plot in **a**) are marked in dark gray, other proteins in light gray. Numbers in brackets in (**a**) indicate the number of proteins of the respective reference proteome quantified in whole cell extracts of ATOM40 RNAi experiments. The ATOM40-RNAi cells used for this experiment were taken from the same cultures that were used to isolate mitochondria for defining the mitochondrial importome. The vertical dashed line indicates a p-value of 0.05. Core subunits of the ATOM complex are indicated in (**a**) and (**b**). The β-barrel proteins SAM50, VDAC, the VDAC-like protein Tb927.11.10780 and ATOM40 as well as its partner protein ATOM14 are marked in (**c**).

Supplementary Figure 4

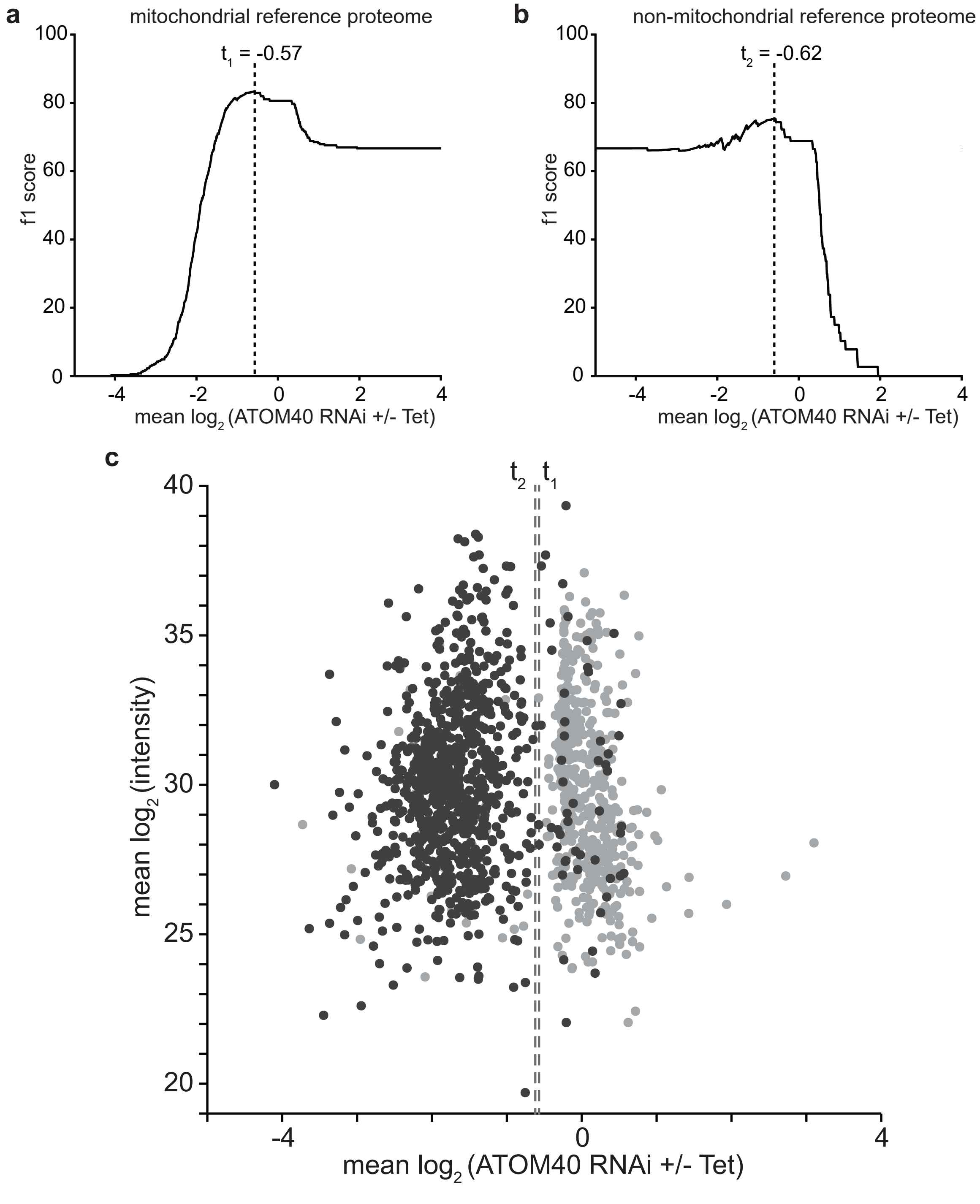

Supplementary Figure 4. Determination of significance thresholds for the delineation of the mitochondrial importome. **a.** F1 scores were calculated using a modified version of the mitochondrial reference proteome defined in this work (selected OM proteins were excluded; see Methods) as set for true positive (TP) hits. F1 scores were determined for defined SILAC ratios starting at the lowest ratio at which at least one TP of our mitochondrial reference proteome and one false positive (FP) hit, derived from the non-mitochondrial reference proteome, were present in the data pool and increasing stepwise in 0.01-increments until the maximum ratio was reached. The ratio giving the maximum F1 score is referred to as t_1. Proteins present in the mitochondrial and non-mitochondrial reference proteomes are listed in Supplementary Tables 2a and 2b. +Tet, induced, -Tet, uninduced ATOM40-RNAi cells. **b.** F1 scores calculated as described in (a) using the non-mitochondrial reference proteome as set for TP and the mitochondrial reference proteome as set for FP hits. The ratio with the highest F1 score for the non-mitochondrial reference proteins is referred to as t_2. +Tet, induced, -Tet, uninduced ATOM40-RNAi cells. **c.** Distribution of SILAC ratios of mitochondrial (black) and non-mitochondrial (gray) reference proteins obtained from gradient-purified mitochondria of induced (+Tet) and uninduced (-Tet) ATOM40-RNAi cells. The F1 score t_2, imposing a more stringent filter on the dataset, was used as significance threshold for the definition of the mitochondrial importome.

Supplementary Figure 5

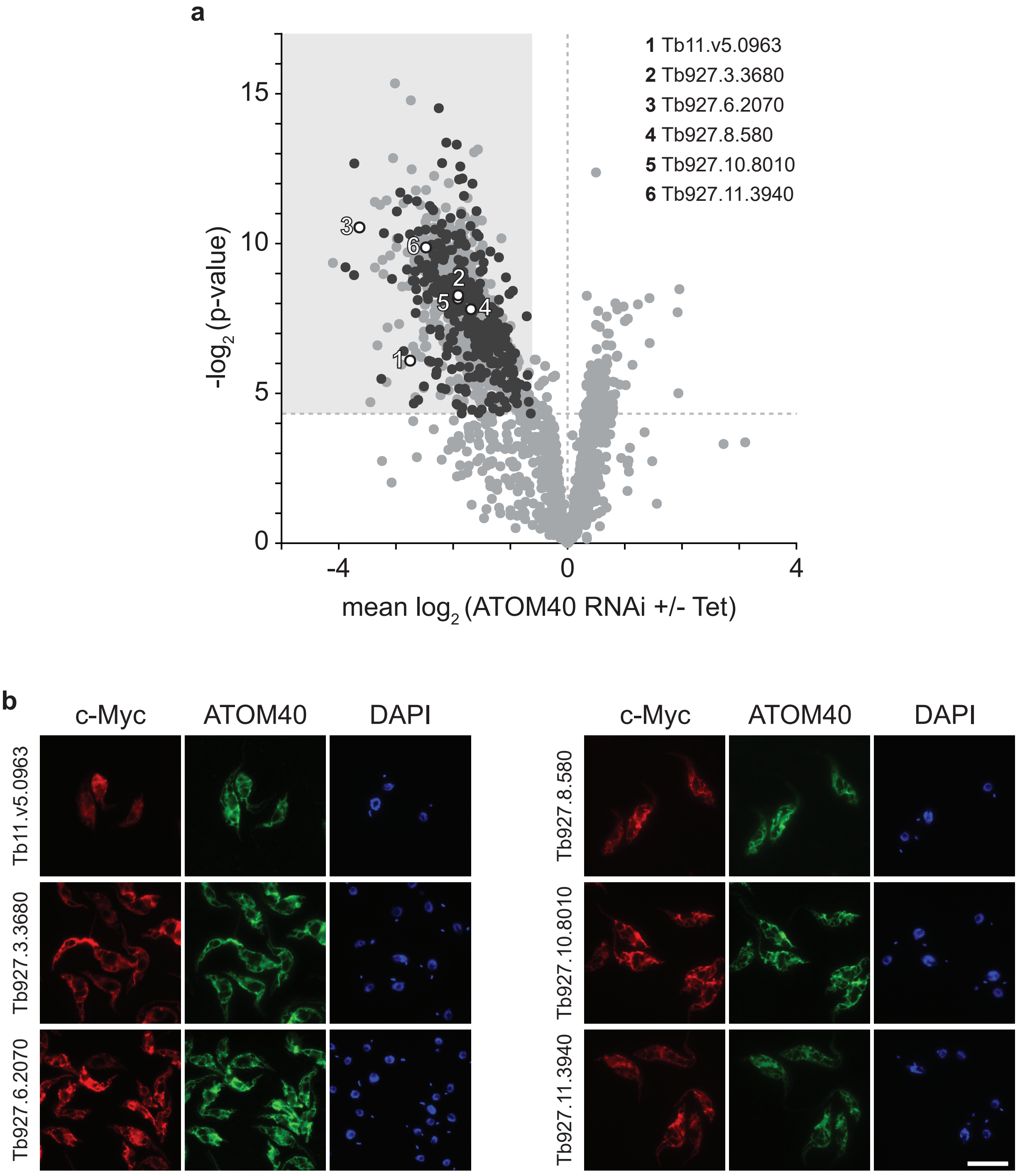

Supplementary Figure 5. Validation of six mitochondrial proteins. **a.** Volcano plot as shown in Fig. 2b. Proteins selected for further validation of their mitochondrial localization are annotated by gene IDs as shown. New mitochondrial candidate proteins are depicted in black, all other proteins in light gray. **b.** Immunofluorescence microscopy analysis of selected proteins of the mitochondrial importome as highlighted in a. Candidates expressed in *T. brucei* as C-terminally tagged c-Myc fusion proteins (red) were stained using anti-Myc antibodies. Mitochondria were visualized using anti-ATOM40 serum (green), nuclear and mitochondrial DNA were stained with DAPI (blue). Bar, 10 µm.

Supplementary Figure 6

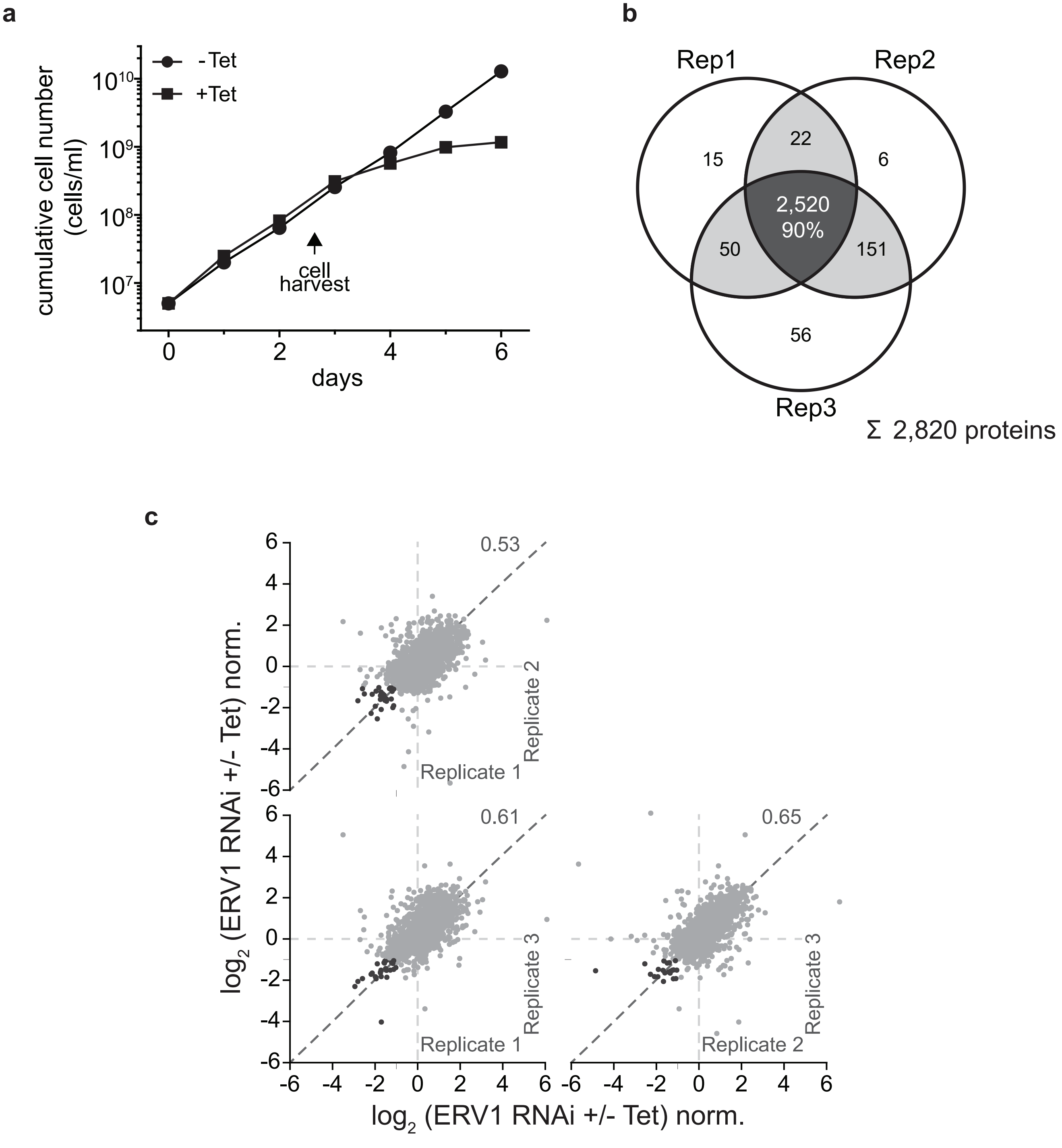

Supplementary Figure 6. ERV1-RNAi experiments. **a.** Growth curves of tetracycline-induced (+Tet) and uninduced (-Tet; control) ERV1-RNAi cells. For MS-based quantitative proteomics experiments, induced and uninduced cells were harvested at day 2.5 after addition of tetracycline, before the onset of a growth phenotype. **b.** Overlap of proteins quantified in organelle-enriched fractions prepared from ERV1-RNAi cells by quantitative MS employing peptide stable isotope dimethyl labeling. The experiment was performed in three biological replicates. Rep, replicate. **c.** Multiscatter plot of protein abundance ratios determined in individual replicates. For each replicate, normalized (norm.) abundance ratios of proteins identified in organelle-enriched fractions of induced (+Tet) *versus* uninduced (-Tet) ERV1-RNAi cells were determined by MaxQuant, $\log_2$-transformed and plotted against each other. Values indicate the Pearson correlation coefficient between replicates. Proteins with a mean $\log_2$ ratio of ≤ -1 are depicted in dark gray, all others in light gray.

Supplementary Figure 7

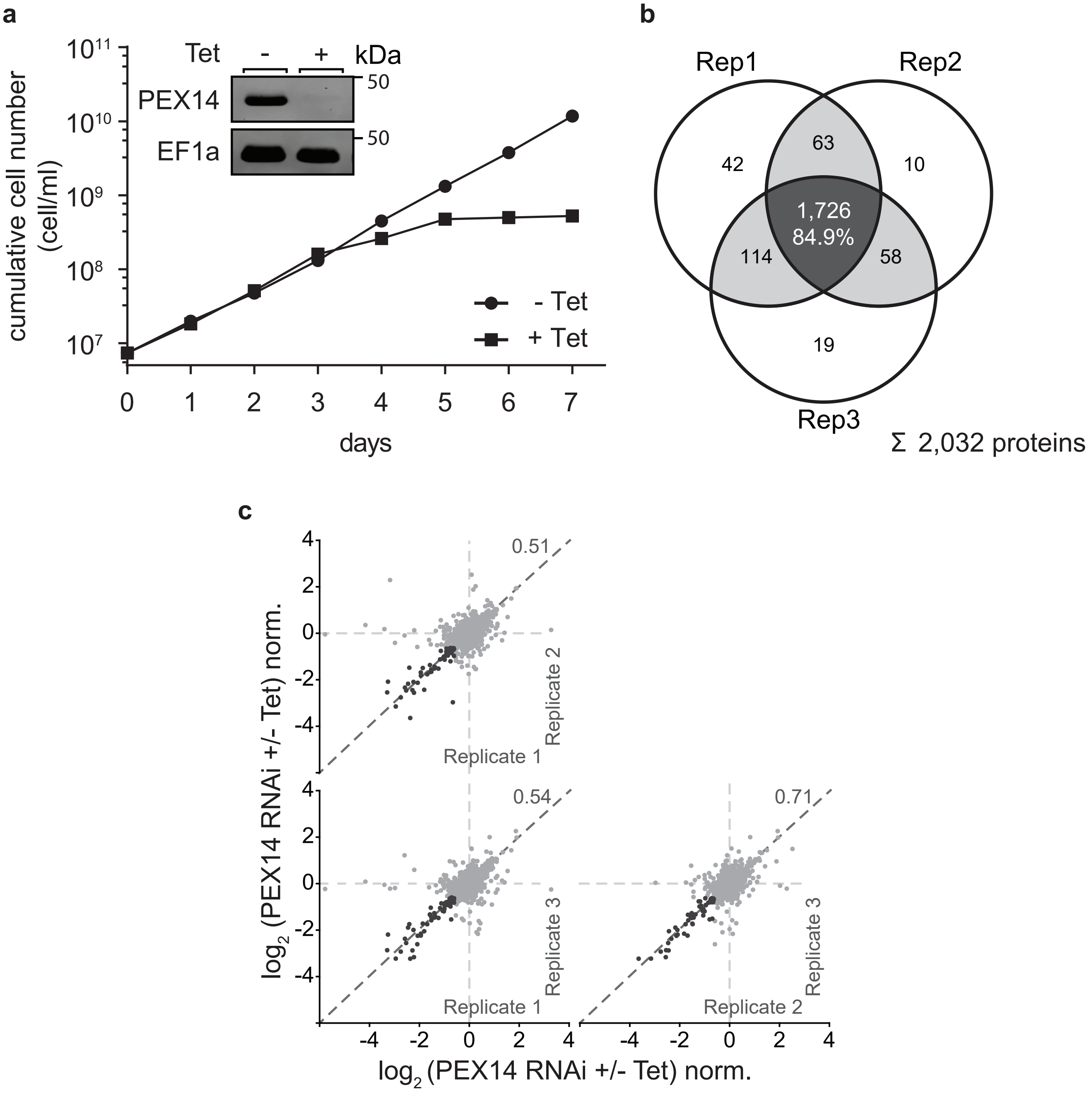

Supplementary Figure 7. PEX14-RNAi experiments. **a.** Growth curve of uninduced (-Tet) and induced (+Tet) PEX14-RNAi cells. The cells were grown for 3 days followed by induction (+Tet) of PEX14-RNAi for 4 days. Uninduced (-Tet) cells served as control. For SILAC-MS analyses, 3 biological replicates were performed. Insert: Immunoblot analysis for confirmation of PEX14 knockdown. Total cell extracts were prepared from uninduced (-Tet) and induced (+Tet) PEX14-RNAi cells. Shown are the relative amounts of the RNAi target PEX14 and the cytosolic protein EF1a, which served as loading control. **b.** Overlap of proteins quantified in glycosome-containing fractions from induced (+Tet) and uninduced (-Tet) SILAC-PEX14-RNAi cells in three biological replicates. Rep, replicate. **c.** Multiscatter plot of protein abundance ratios determined in individual replicates. Normalized (norm.) SILAC ratios of proteins detected in glycosome-containing fractions of induced (+Tet) *versus* uninduced (-Tet) PEX14-RNAi cells were calculated for each replicate by MaxQuant, log_2-transformed and plotted against each other. Values indicate the Pearson correlation coefficient between replicates. Proteins with a mean log_2 ratio of ≤ 0.59 are depicted in dark gray, all others in light gray.

Supplementary Figure 8

Full scans for Figure 1b

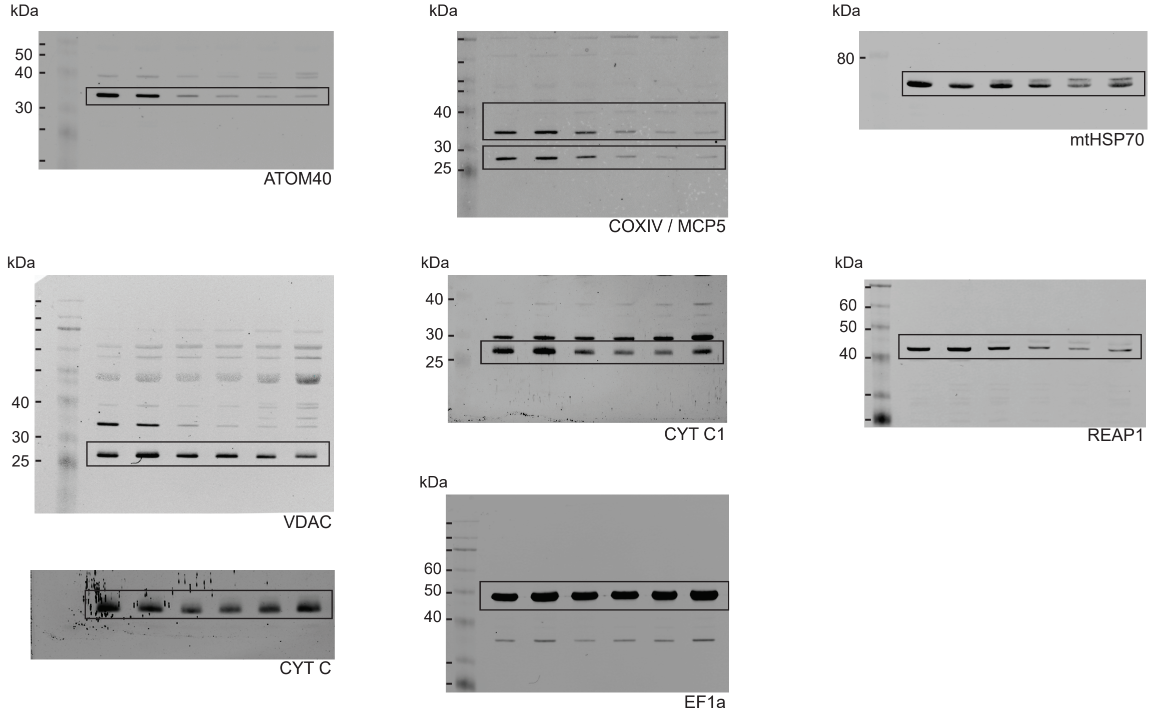

Full scans for Figure 1c

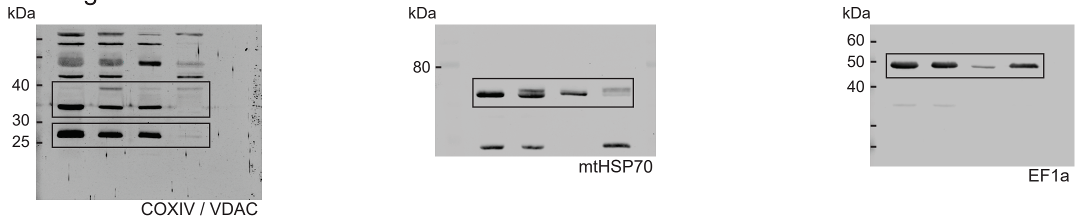

Full scans for Figure 1d

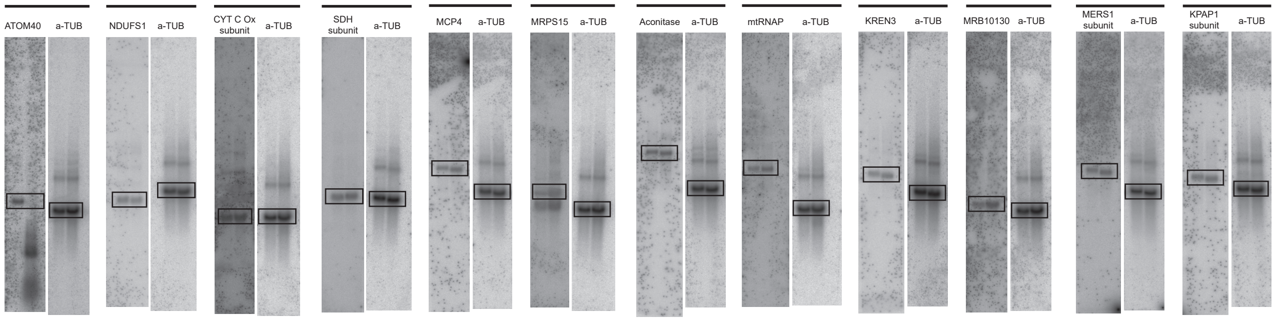

Full scans for Figure 1e

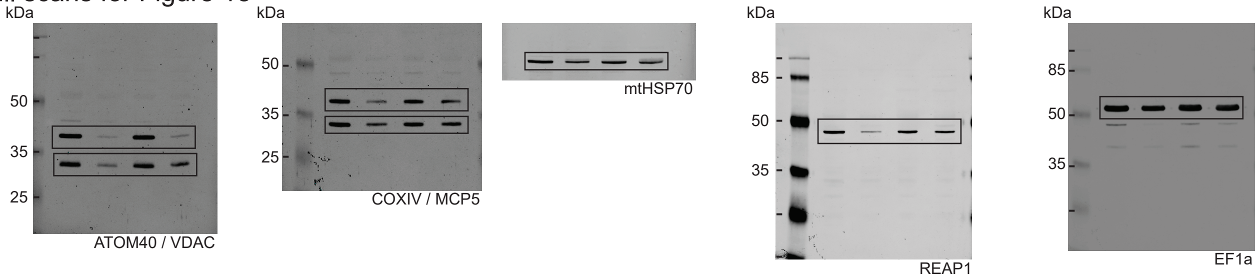

Full scans for Figure 4a

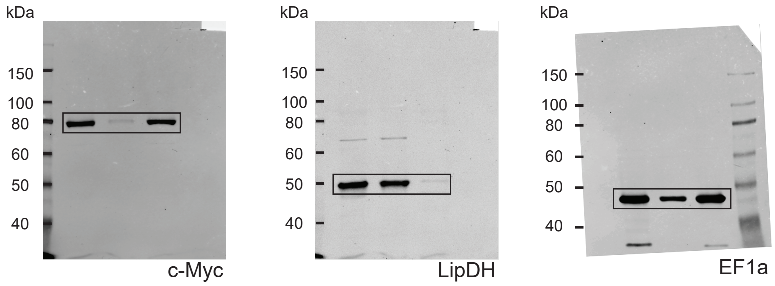

Invitrogen Prestained Protein Ladder, Broad Range (10-230 kDa)

Full scans for Figure 4b

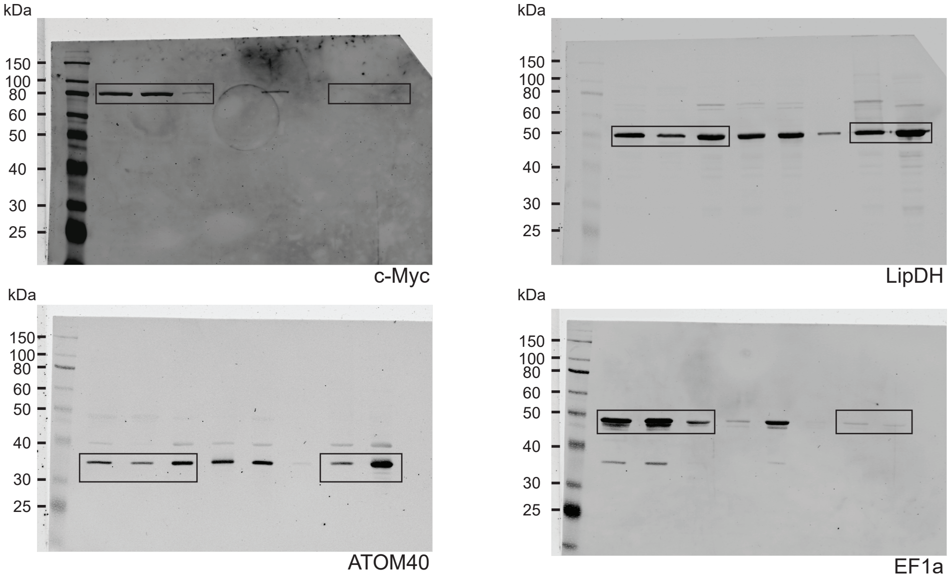

Full scans for Figure 5a

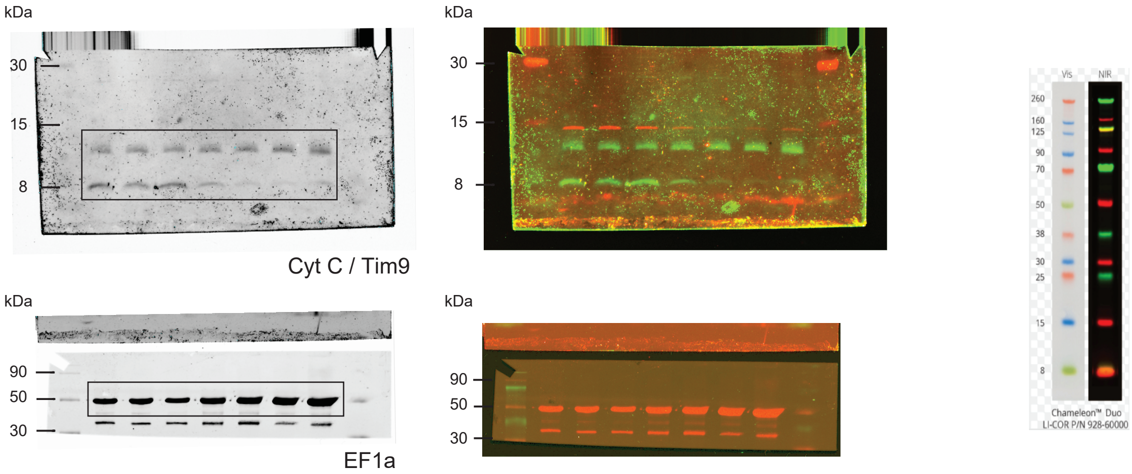

Full scans for Figure 6b

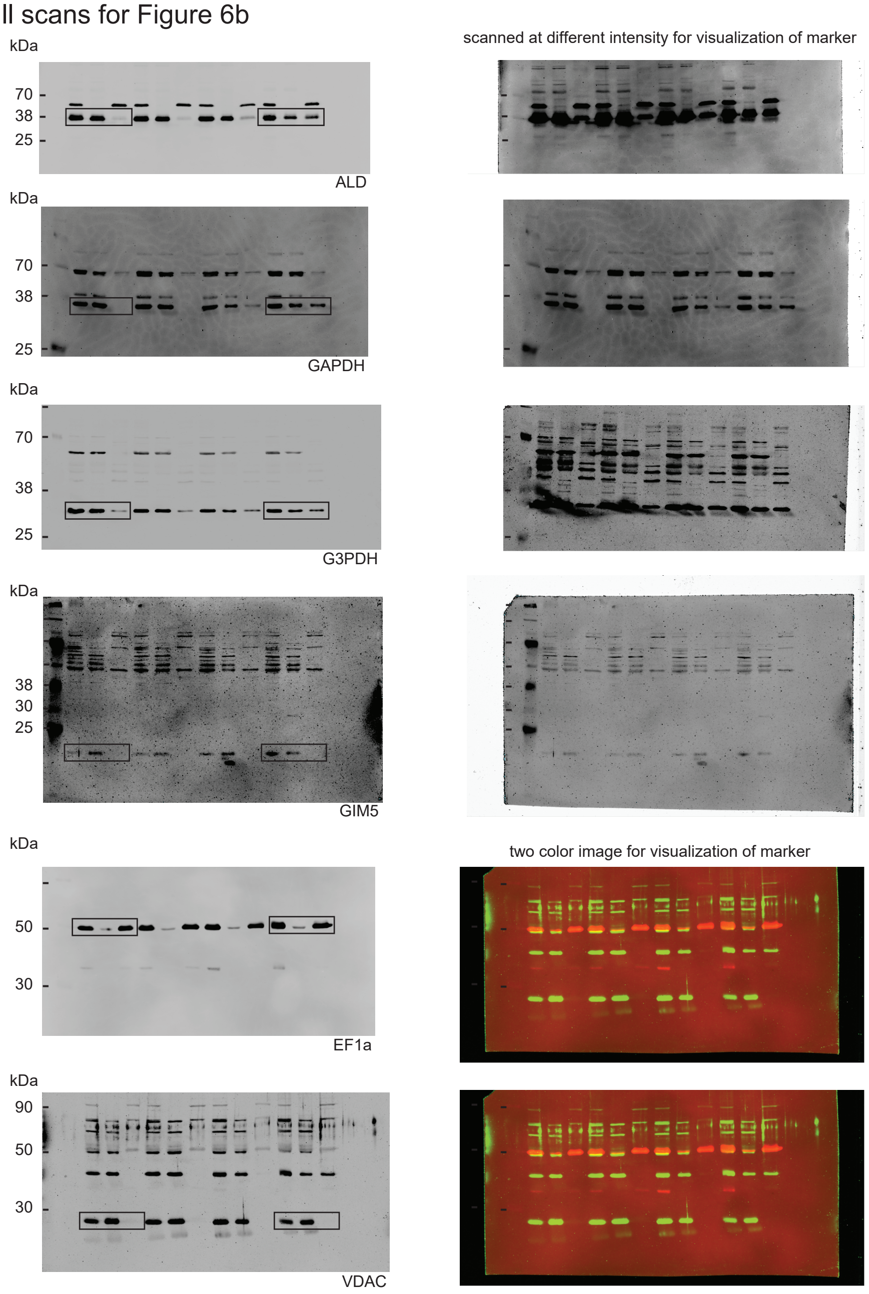

Full scans for Supplementary Figure 1a

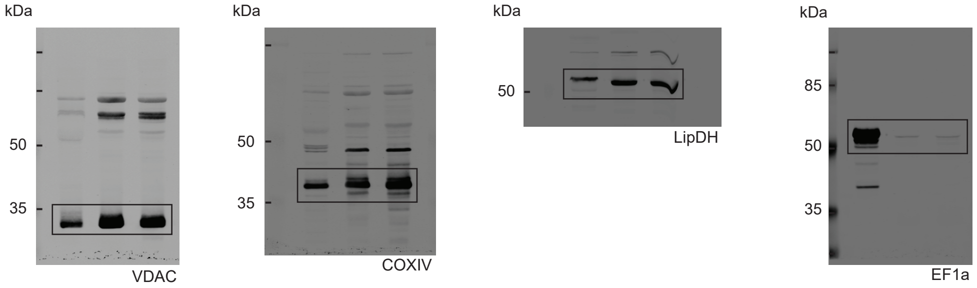

Full scans for Supplementary Figure 1c

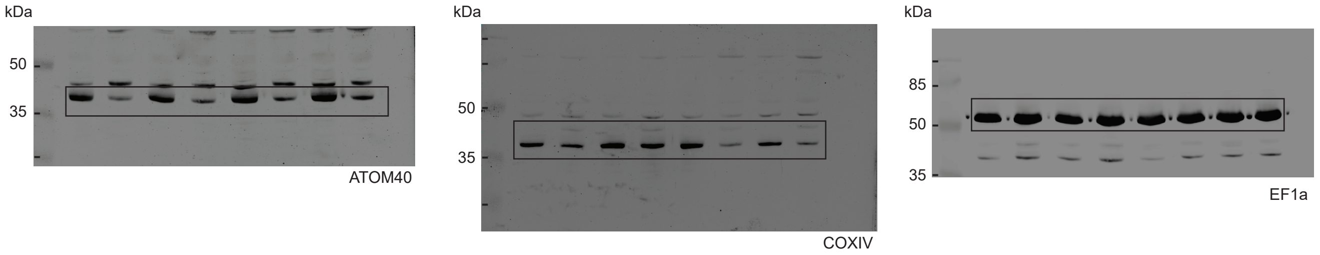

Full scans for Supplementary Figure 7a

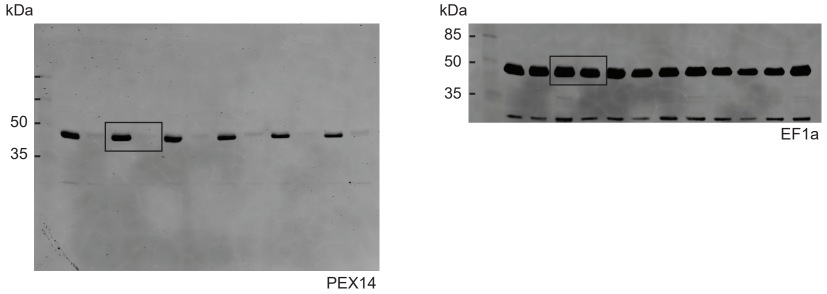

Supplementary Figure 8. Full scans of all blots (including molecular weight markers) shown in order of appearence.

Supplementary References (related to Supplementary Data)

1. Emanuelsson, O., Nielsen, H., Brunak, S. & von Heijne, G. Predicting subcellular localization of proteins based on their N-terminal amino acid sequence. *J Mol Biol* **300**, 1005-1016 (2000).
2. Fukasawa, Y. et al. MitoFates: improved prediction of mitochondrial targeting sequences and their cleavage sites. *Mol Cell Proteomics* **14**, 1113-1126 (2015).
3. Panigrahi, A.K. et al. A comprehensive analysis of Trypanosoma brucei mitochondrial proteome. *Proteomics* **9**, 434-450 (2009).
4. Mach, J. et al. An Advanced System of the Mitochondrial Processing Peptidase and Core Protein Family in Trypanosoma brucei and Multiple Origins of the Core I Subunit in Eukaryotes. *Genome Biol Evol* **5**, 860-875 (2013).
5. Acestor, N., Panigrahi, A.K., Ogata, Y., Anupama, A. & Stuart, K.D. Protein composition of Trypanosoma brucei mitochondrial membranes. *Proteomics* **9**, 5497-5508 (2009).
6. Niemann, M. et al. Mitochondrial outer membrane proteome of Trypanosoma brucei reveals novel factors required to maintain mitochondrial morphology. *Mol Cell Proteomics* **12**, 515-528 (2013).
7. Acestor, N. et al. Trypanosoma brucei mitochondrial respiratome: composition and organization in procyclic form. *Mol Cell Proteomics* **10**, M110.006908 (2011).
8. Colasante, C., Peña Diaz, P., Clayton, C. & Voncken, F. Mitochondrial carrier family inventory of Trypanosoma brucei brucei: Identification, expression and subcellular localisation. *Mol Biochem Parasitol* **167**, 104-117 (2009).
9. Zíková, A., Schnaufer, A., Dalley, R.A., Panigrahi, A.K. & Stuart, K.D. The F(0)F(1)-ATP synthase complex contains novel subunits and is essential for procyclic Trypanosoma brucei. *PLoS Pathog* **5**, e1000436 (2009).
10. Zíková, A. et al. Trypanosoma brucei mitochondrial ribosomes: affinity purification and component identification by mass spectrometry. *Mol Cell Proteomics* **7**, 1286-1296 (2008).
11. Göringer, H.U. 'Gestalt,' composition and function of the Trypanosoma brucei editosome. *Annu Rev Microbiol* **66**, 65-82 (2012).
12. Aphasizhev, R. & Aphasizheva, I. Mitochondrial RNA processing in trypanosomes. *Res Microbiol* **162**, 655-663 (2011).
13. Ammerman, M.L. et al. Architecture of the trypanosome RNA editing accessory complex, MRB1. *Nucleic Acids Res* **40**, 5637-5650 (2012).
14. Zhang, X. et al. The Trypanosoma brucei MitoCarta and its regulation and splicing pattern during development. *Nucleic Acids Res* **38**, 7378-7387 (2010).
15. Subota, I. et al. Proteomic analysis of intact flagella of procyclic Trypanosoma brucei cells identifies novel flagellar proteins with unique sub-localization and dynamics. *Mol Cell Proteomics* **13**, 1769-1786 (2014).
16. Hashem, Y. et al. High-resolution cryo-electron microscopy structure of the Trypanosoma brucei ribosome. *Nature* **494**, 385-389 (2013).
17. Mani, J. et al. Mitochondrial protein import receptors in Kinetoplastids reveal convergent evolution over large phylogenetic distances. *Nat Commun* **6**, 6646 (2015).
18. Desy, S., Mani, J., Harsman, A., Kaser, S. & Schneider, A. TbLOK1/ATOM19 is a novel subunit of the noncanonical mitochondrial outer membrane protein translocase of Trypanosoma brucei. *Mol Microbiol* (2016).
19. Gentle, I.E. et al. Conserved motifs reveal details of ancestry and structure in the small TIM chaperones of the mitochondrial intermembrane space. *Mol Biol Evol* **24**, 1149-1160 (2007).
20. Harsman, A. et al. The non-canonical mitochondrial inner membrane presequence translocase of trypanosomatids contains two essential rhomboid-like proteins. *Nat Commun* **7**, 13707 (2016).
21. Güther, M.L.S., Urbaniak, M.D., Tavendale, A., Prescott, A. & Ferguson, M.A.J. High-confidence glycosome proteome for procyclic form Trypanosoma brucei by epitope-tag organelle enrichment and SILAC proteomics. *J Proteome Res* **13**, 2796-2806 (2014).
