# Supplementary material for: Charting organellar importomes by quantitative mass spectrometry
Source: Nat Commun. 2017 May 9;8:15272. doi: 10.1038/ncomms15272 (PMC5436138; doi:10.1038/ncomms15272)

Supplementary Data 14

Supplementary Data 14. Annotated MS/MS spectra of peptides used for single-peptide protein identifications in gradient-purified mitochondria of tetracycline-induced and uninduced ATOM40-RNAi cells. Spectra were generated using the Viewer integrated in MaxQuant.

Raw file	Scan	Method	Score	m/z
QEplus003059	13514	FTMS; HCD	109.83	839.9

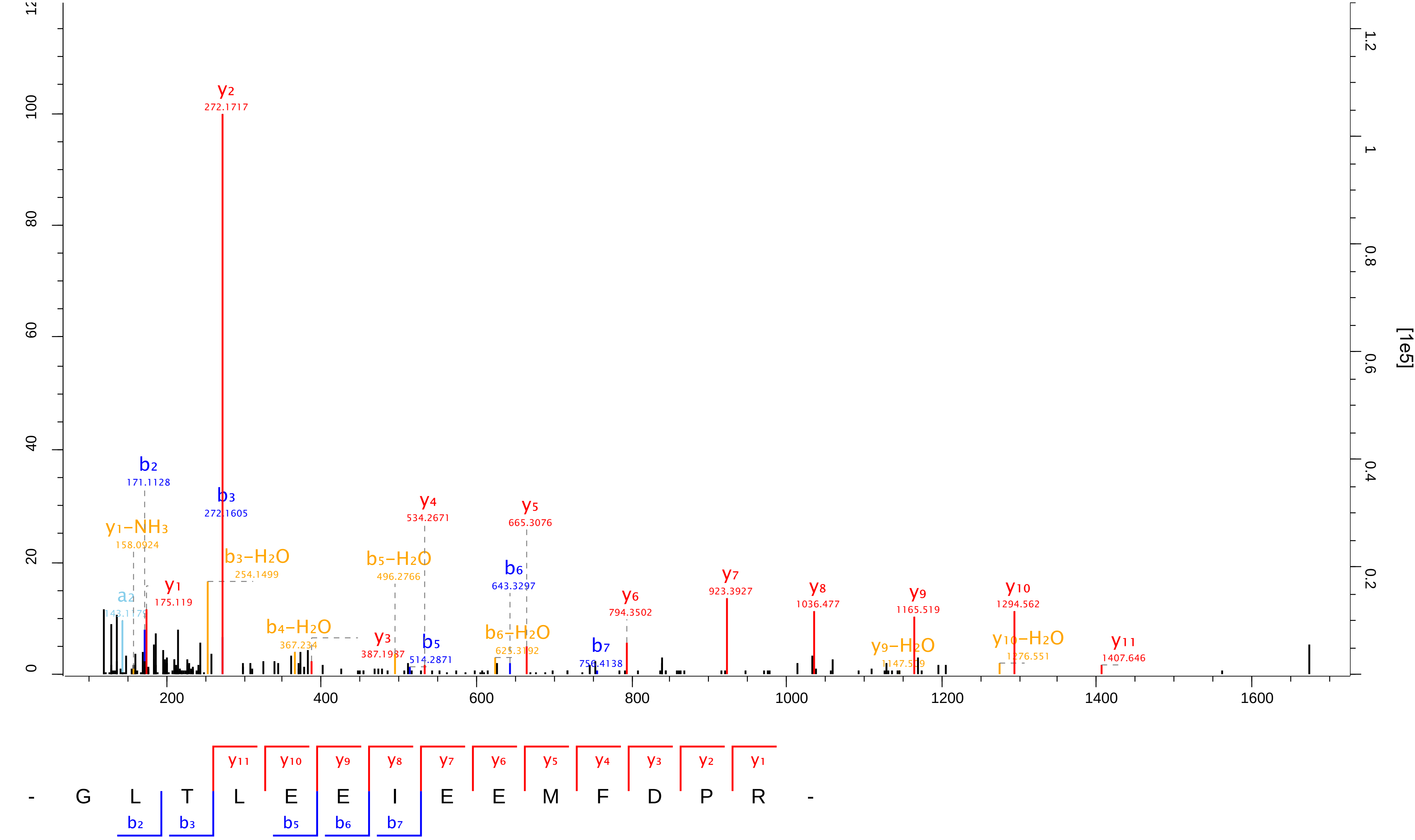

Raw file	Scan	Method	Score	m/z
QEplus003059	6457	FTMS; HCD	54.46	1076.02

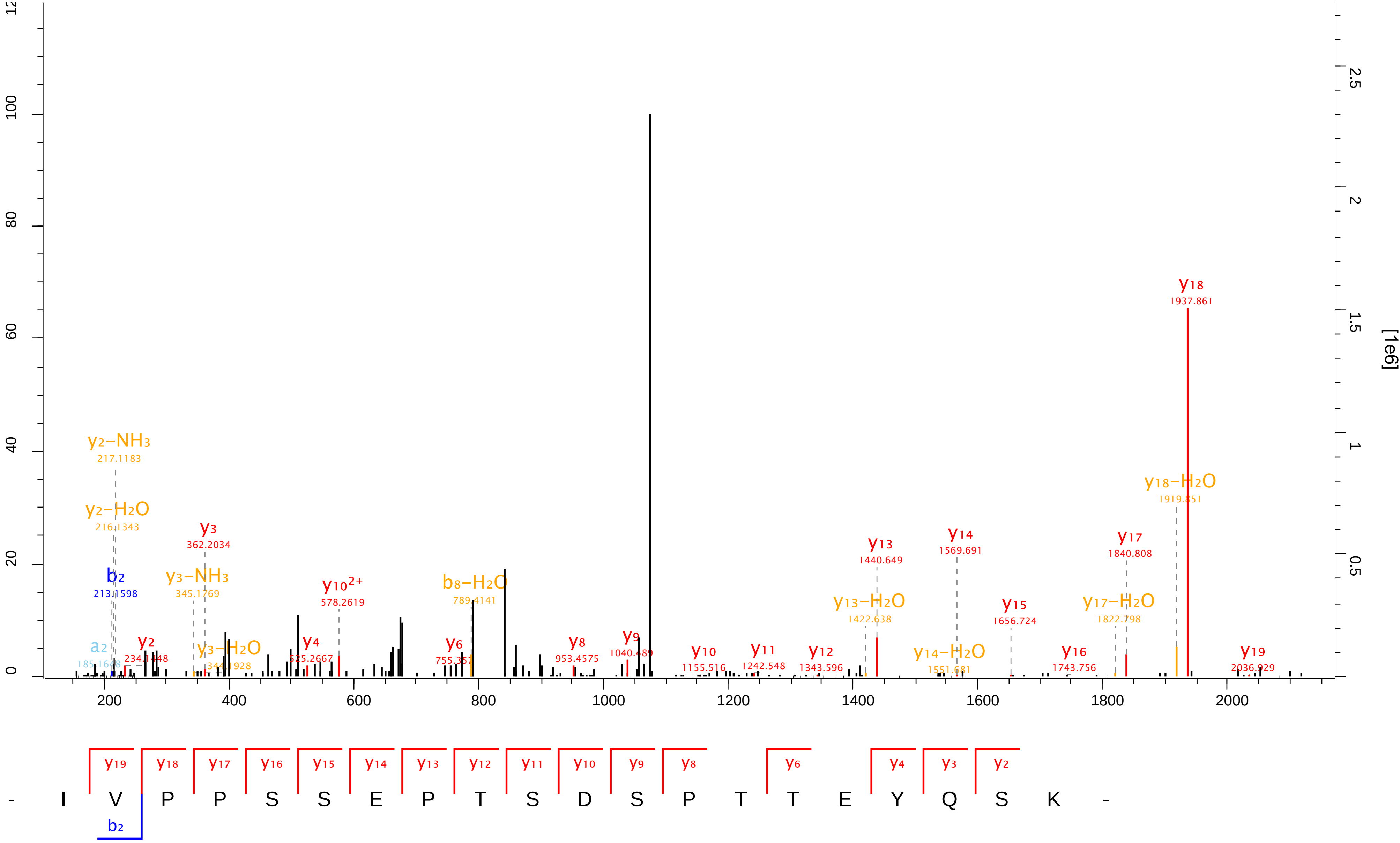

Raw file	Scan	Method	Score	m/z
QEplus003059	9617	FTMS; HCD	62.47	599.33

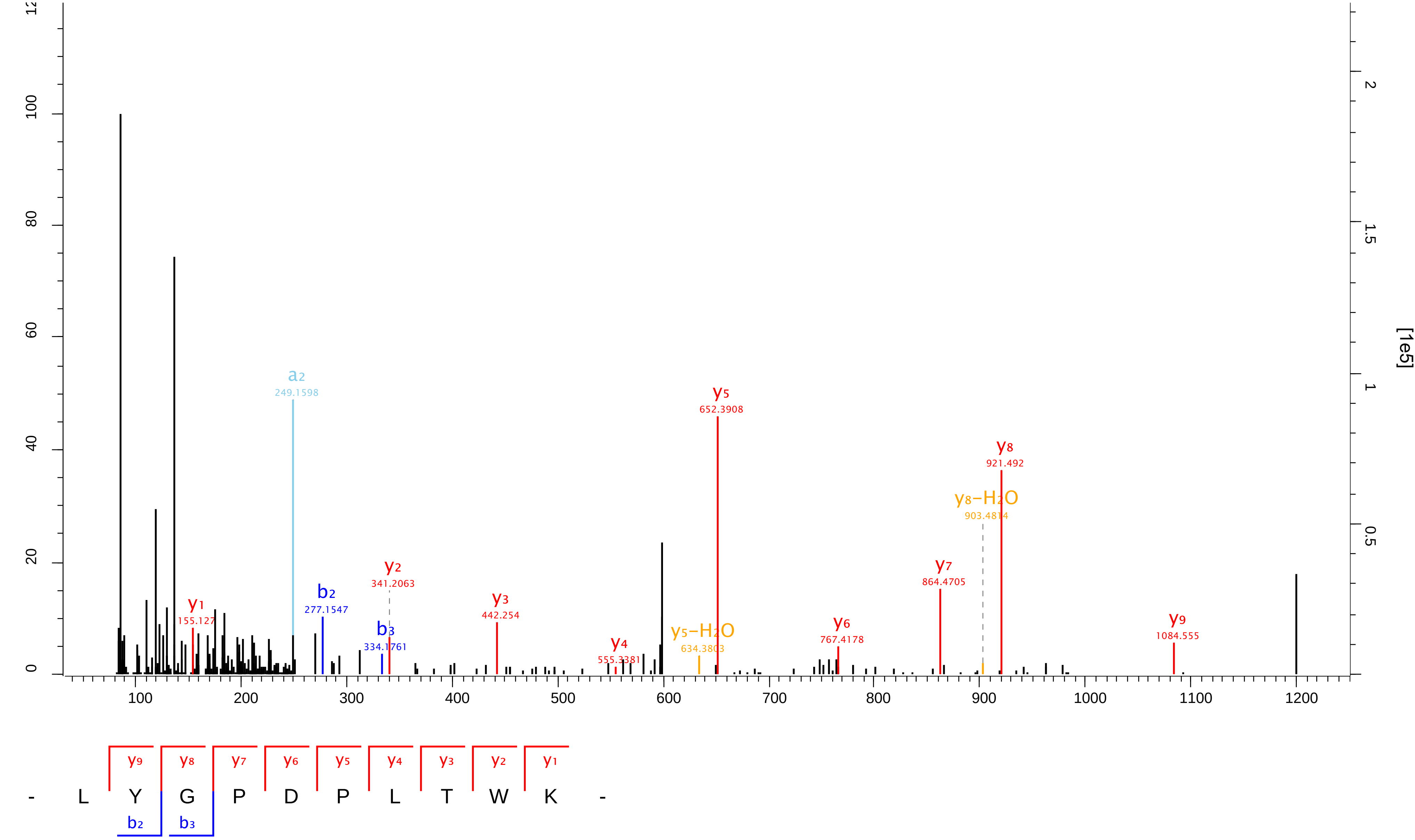

Raw file	Scan	Method	Score	m/z
QEplus003060	10715	FTMS; HCD	93.1	593.34

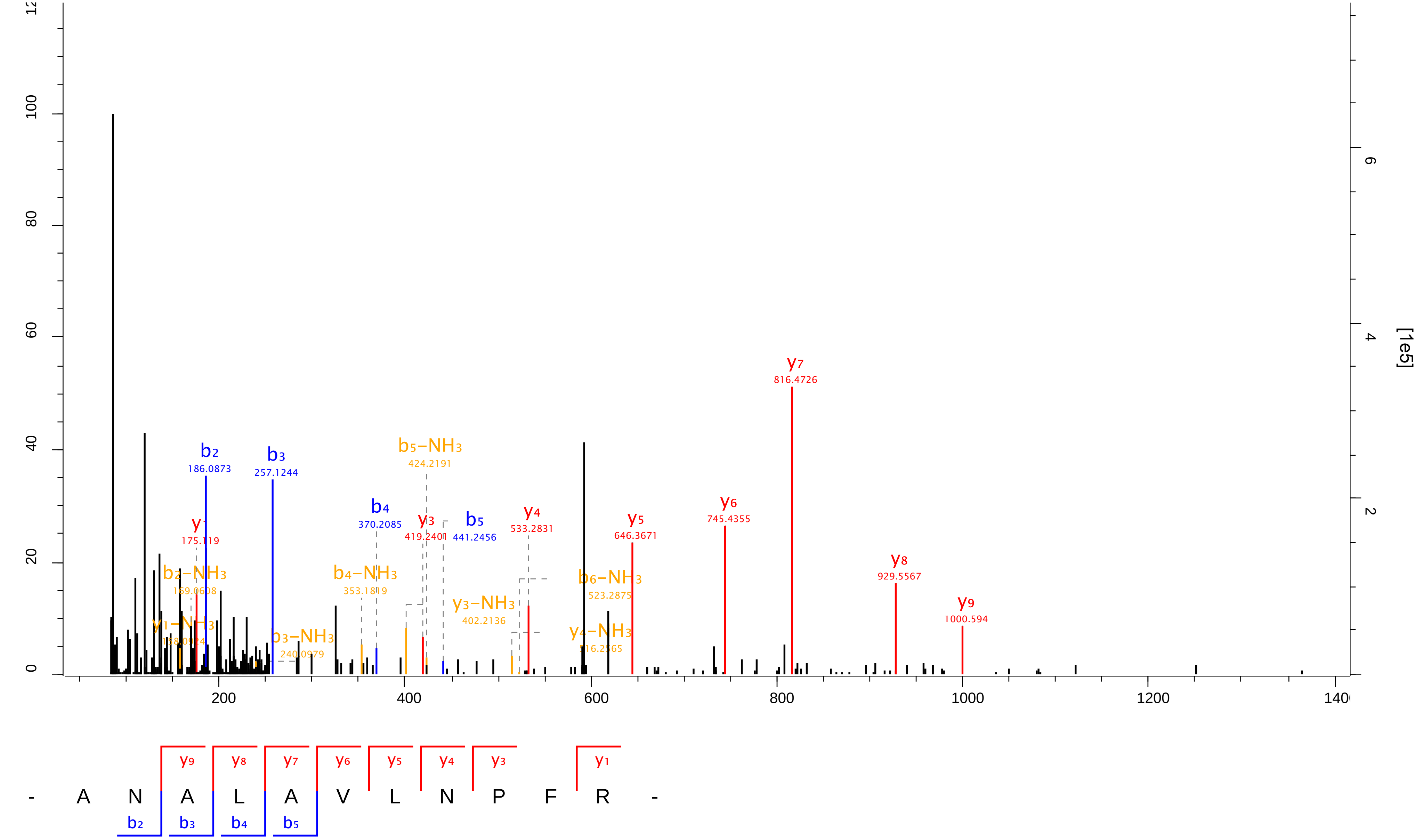

Raw file	Scan	Method	Score	m/z
QEplus003060	13661	FTMS; HCD	112.13	738.45

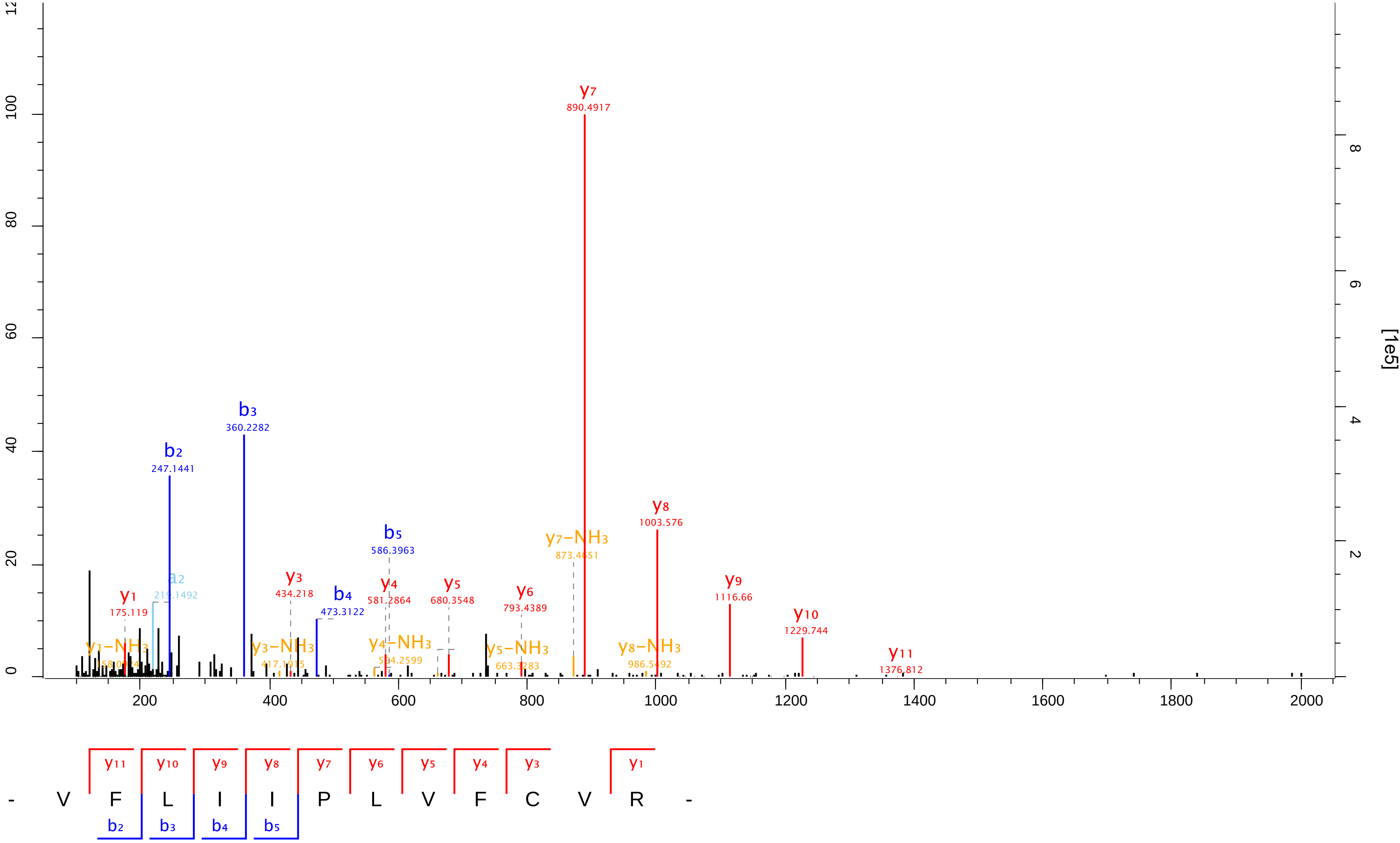

Raw file	Scan	Method	Score	m/z
QEplus003060	2984	FTMS; HCD	72.2	398.55

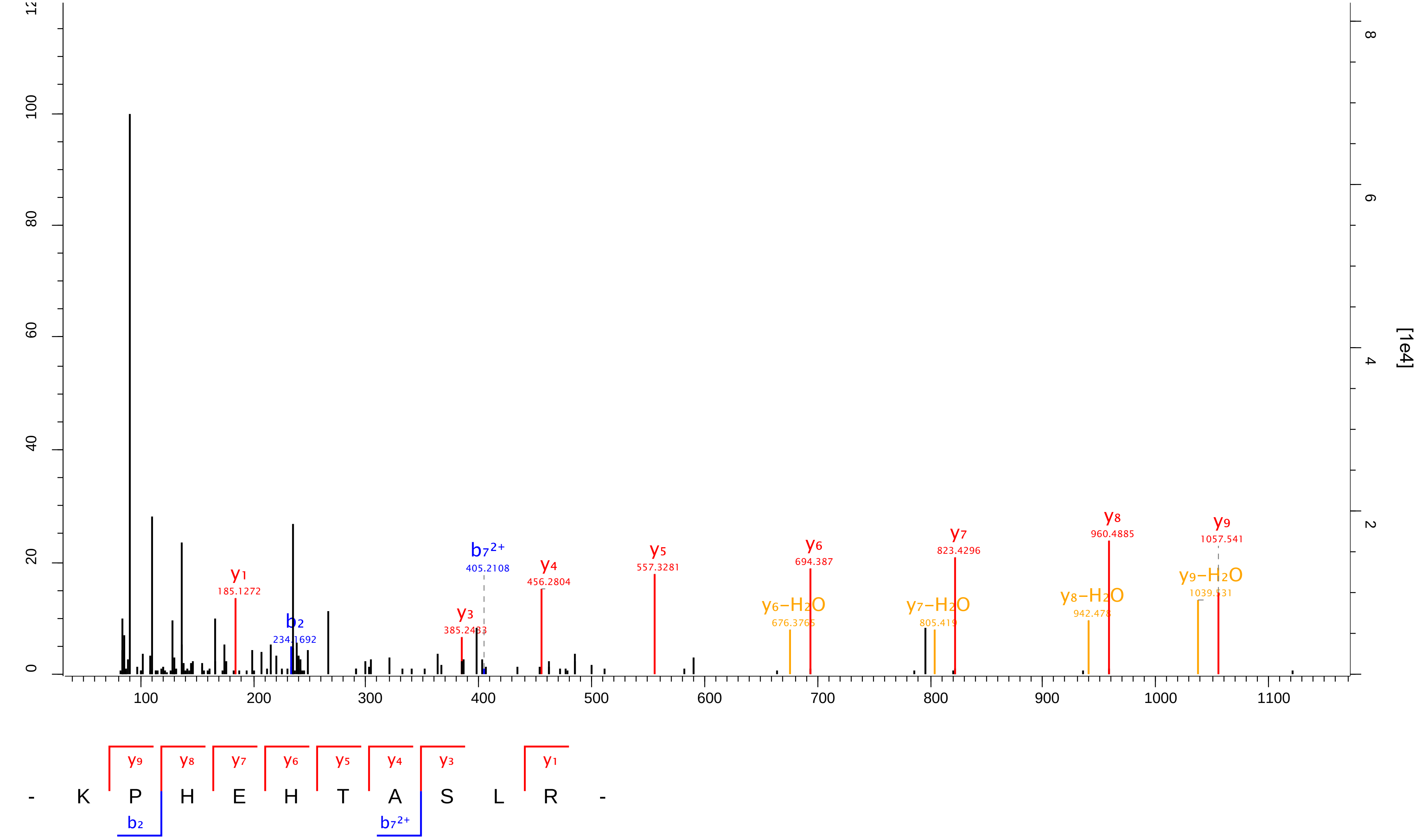

Raw file	Scan	Method	Score	m/z
QEplus003060	8417	FTMS; HCD	55.47	684.36

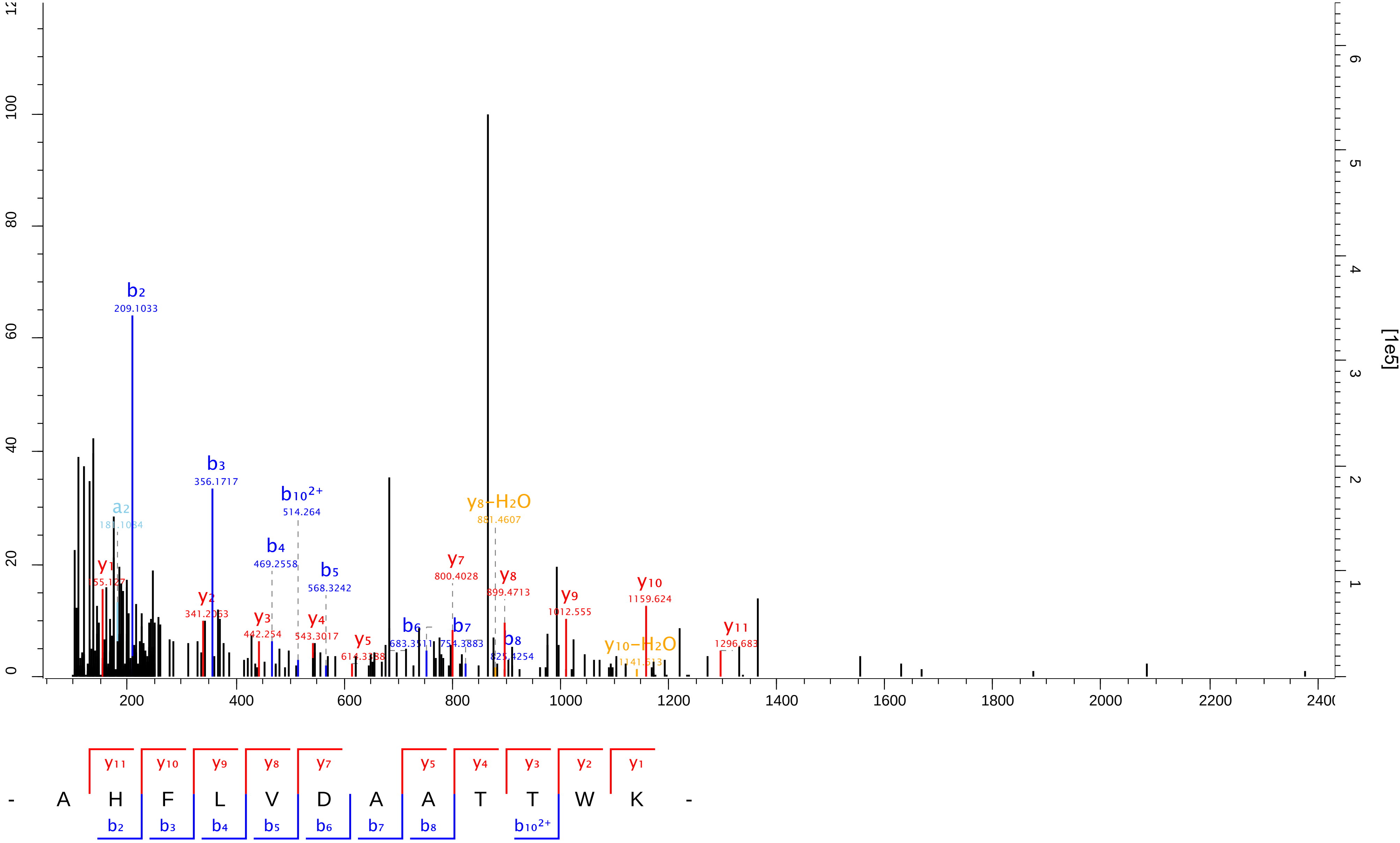

Raw file	Scan	Method	Score	m/z
QEplus003061	10966	FTMS; HCD	93.75	861.46

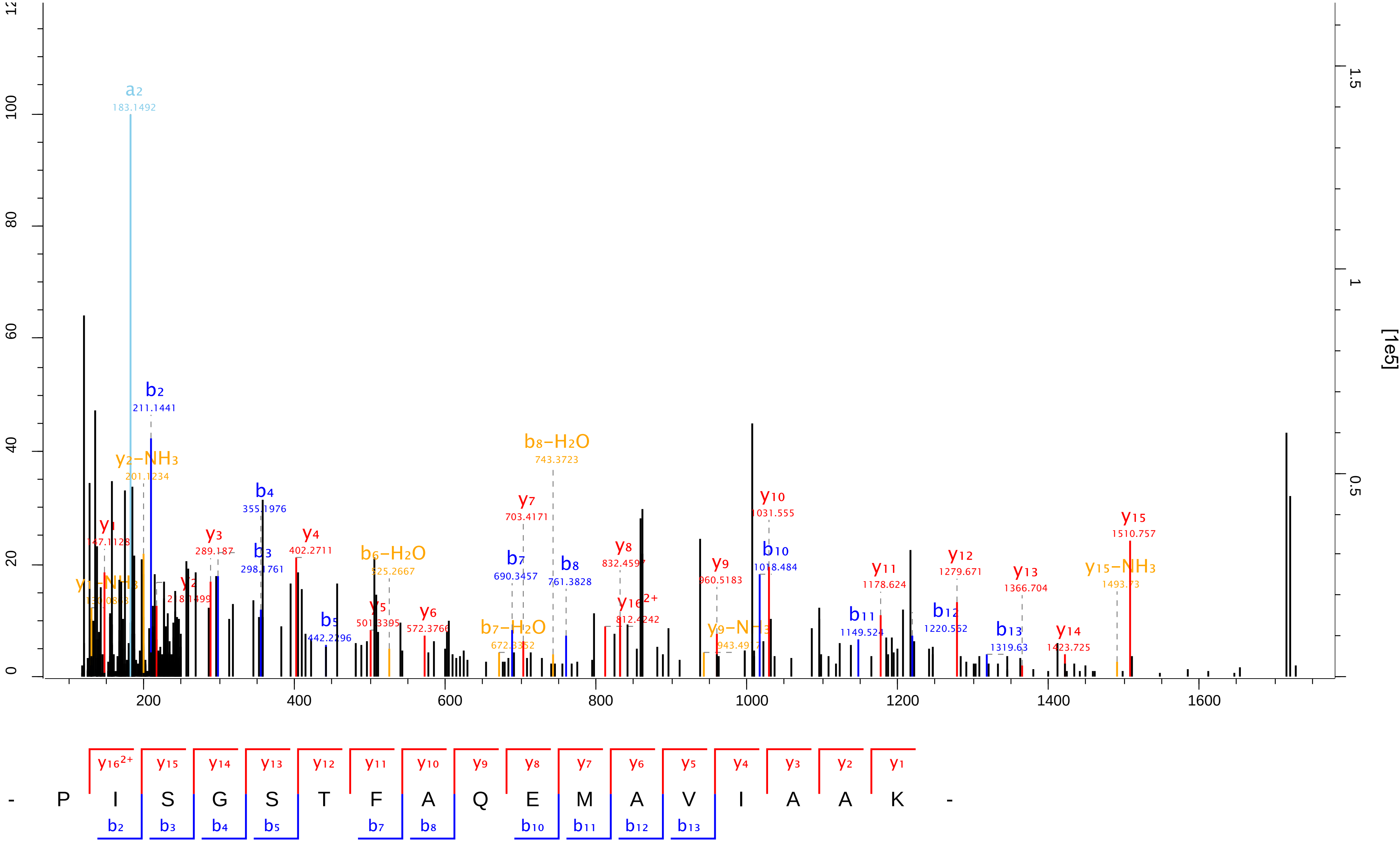

Raw file	Scan	Method	Score	m/z
QEplus003061	4757	FTMS; HCD	67.68	853.9

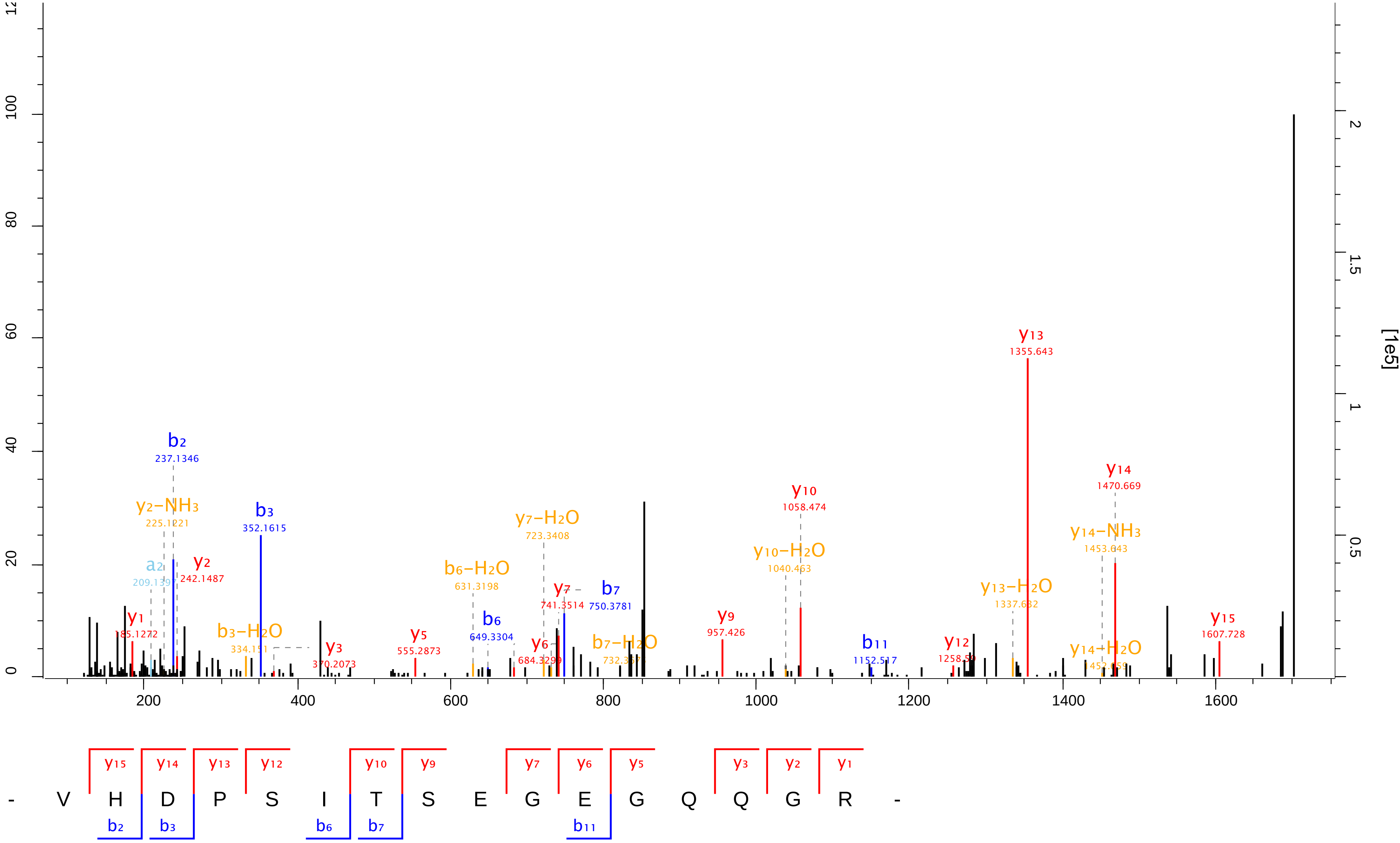

Raw file	Scan	Method	Score	m/z
QEplus003062	14451	FTMS; HCD	80.38	717.06

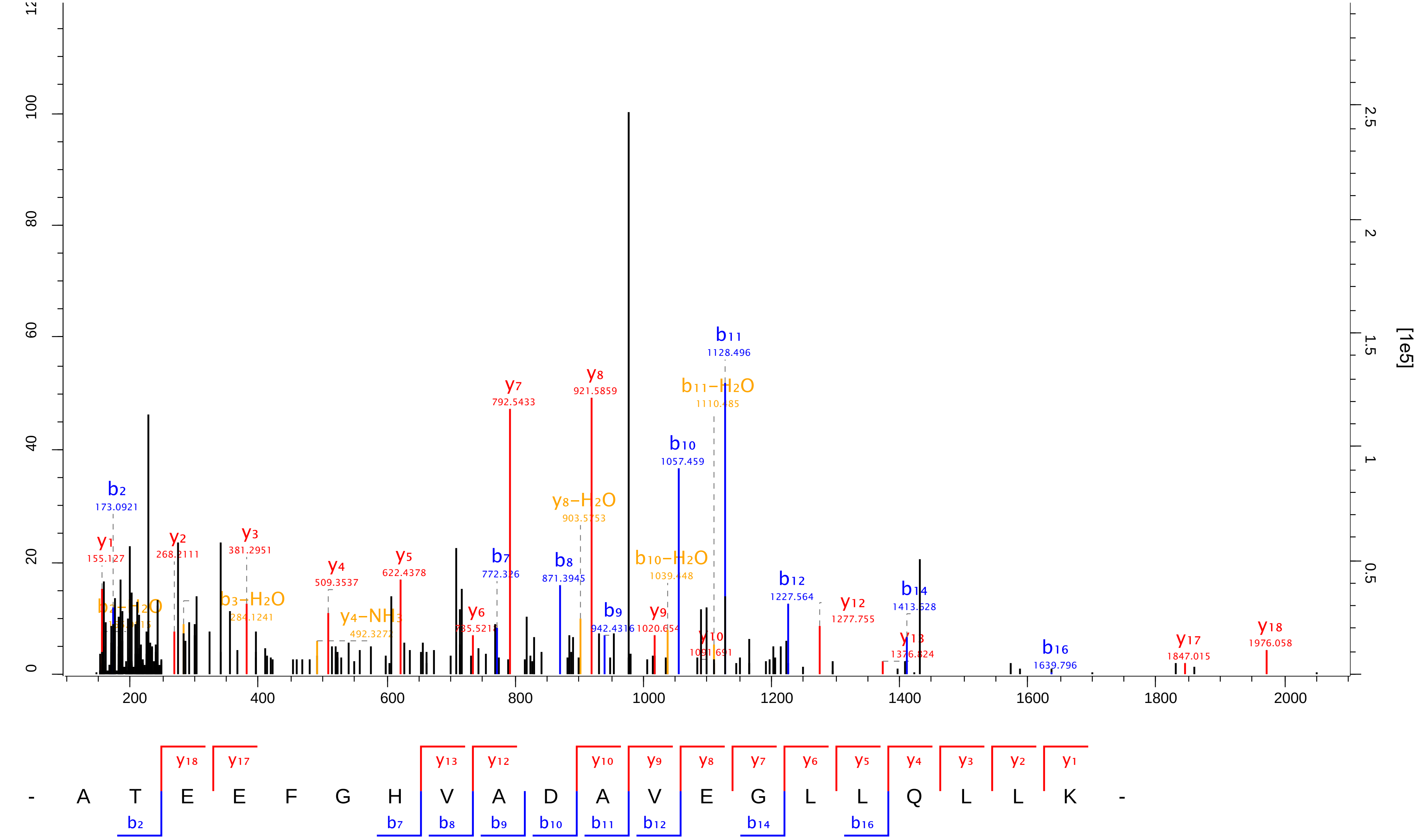

Raw file	Scan	Method	Score	m/z
QEplus003062	14457	FTMS; HCD	48.28	624.36

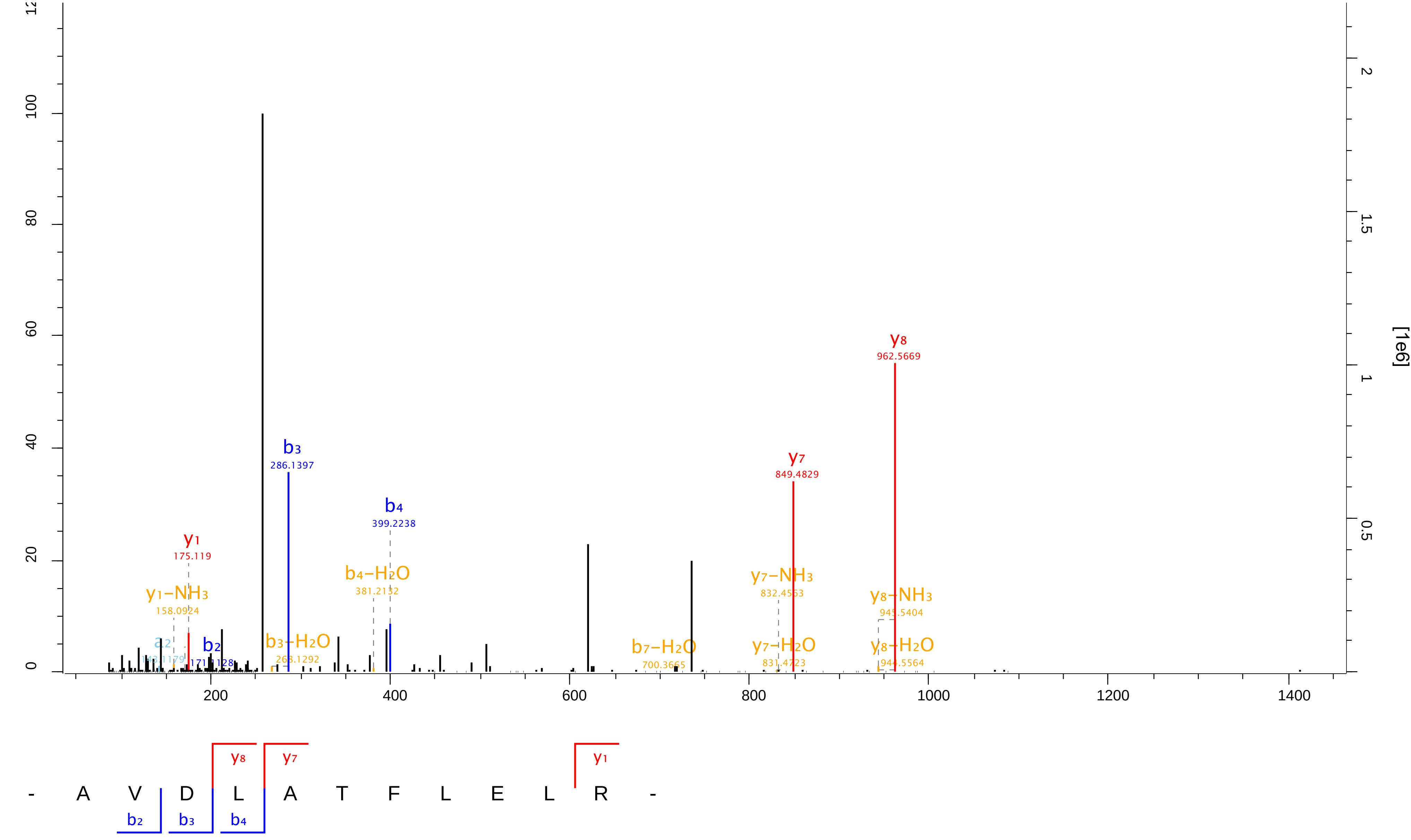

Raw file	Scan	Method	Score	m/z
QEplus003062	14860	FTMS; HCD	74.21	1010.05

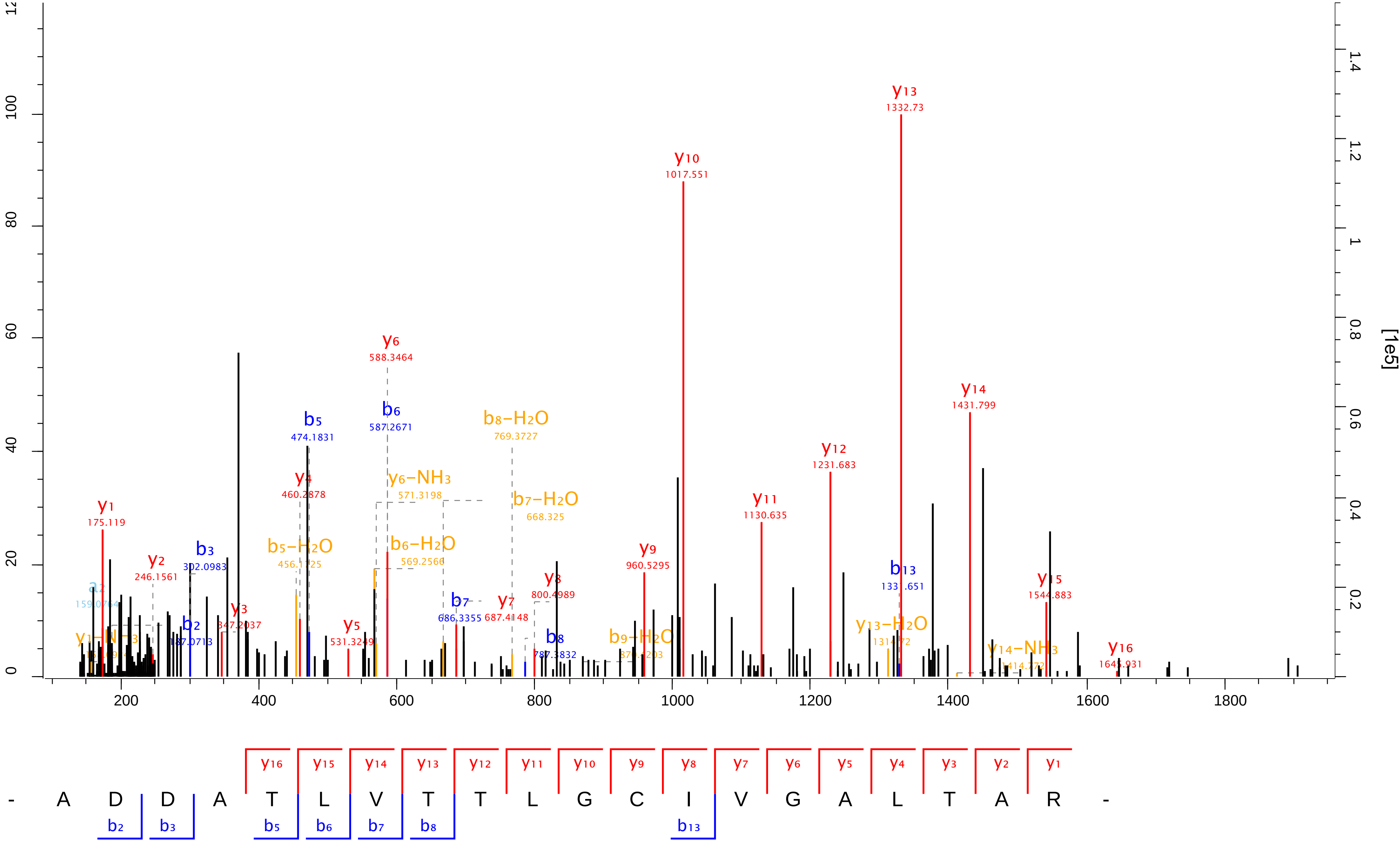

Raw file	Scan	Method	Score	m/z
QEplus003062	15095	FTMS; HCD	134.14	826.95

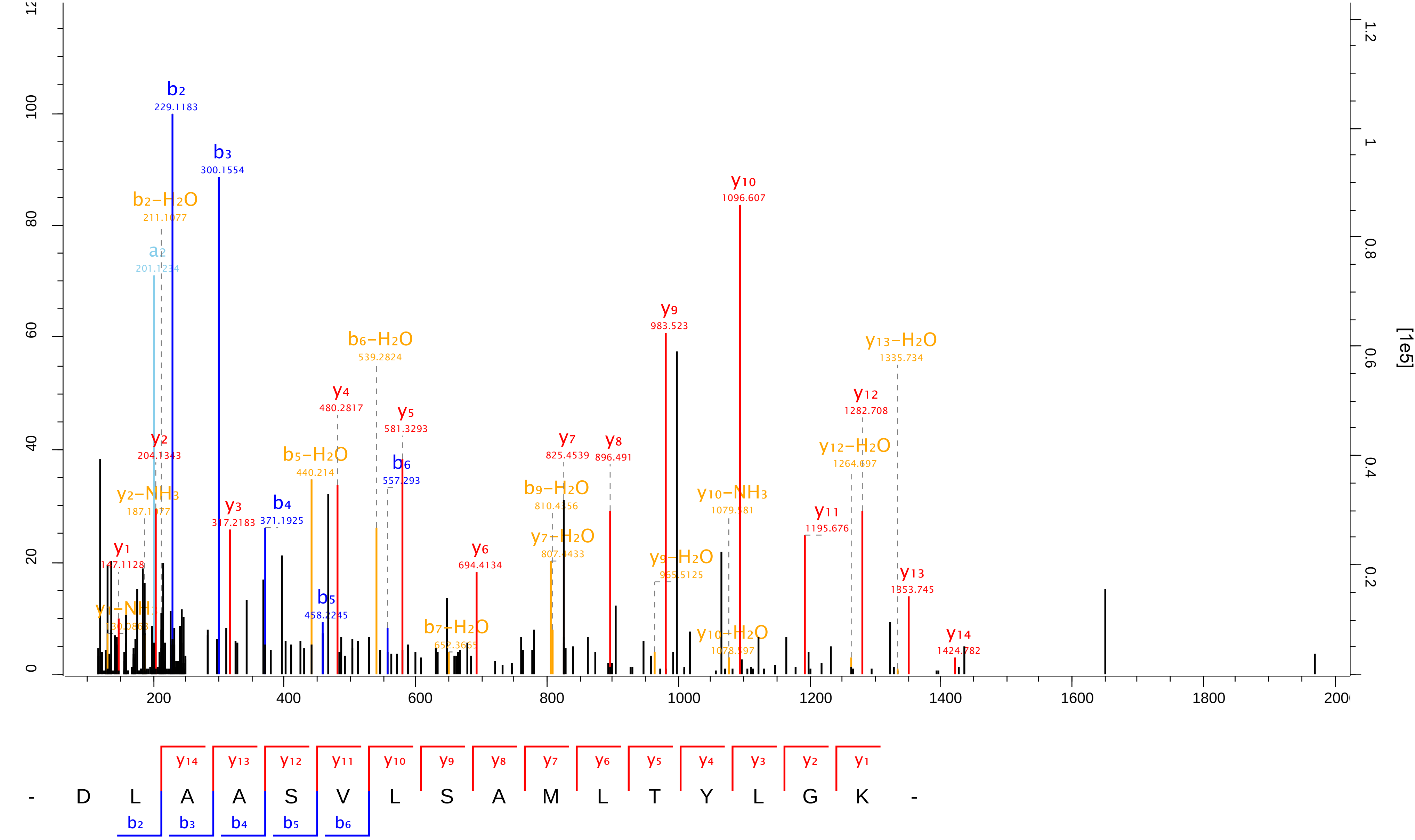

Raw file	Scan	Method	Score	m/z
QEplus003062	2970	FTMS; HCD	78.69	537.25

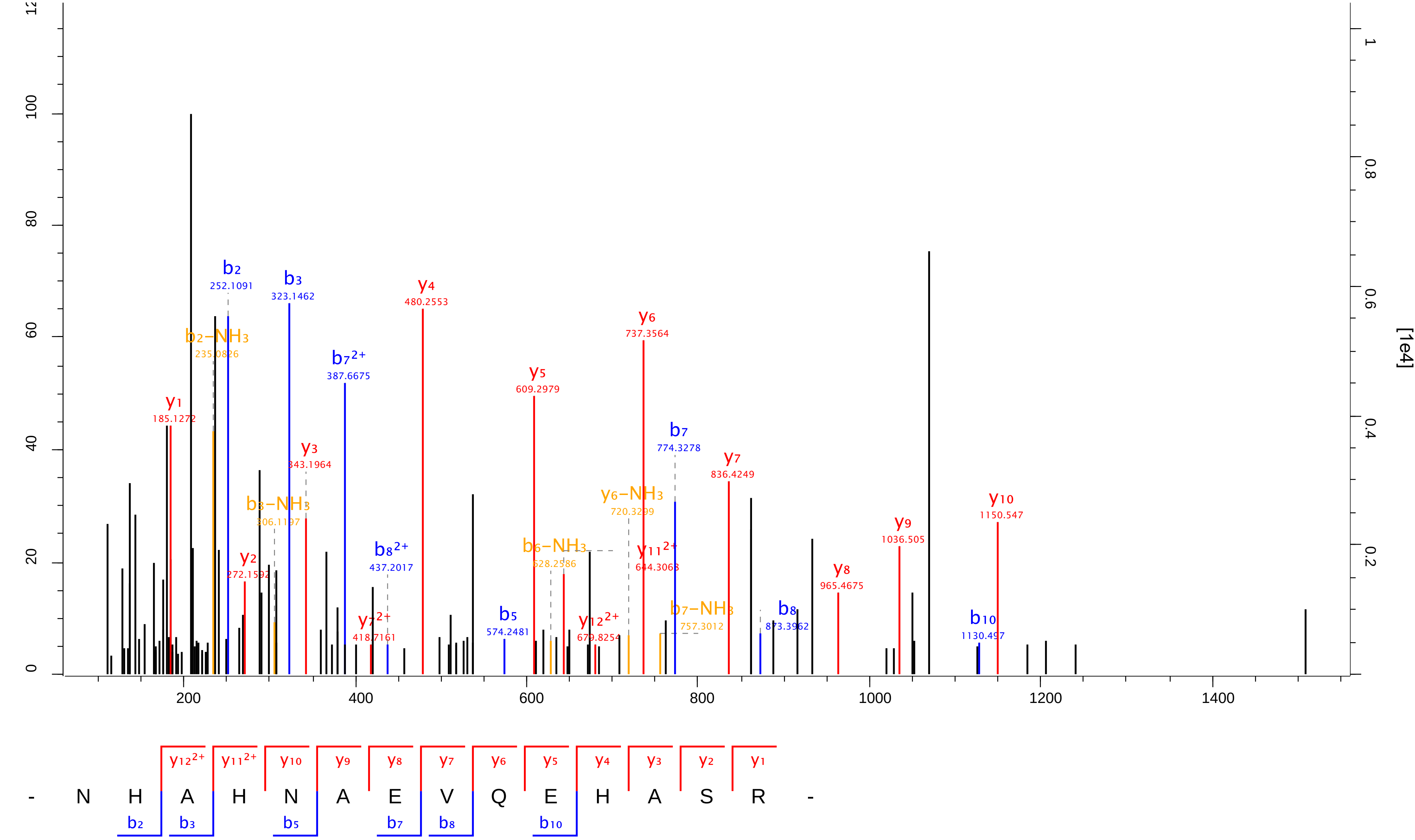

Raw file	Scan	Method	Score	m/z
QEplus003063	13416	FTMS; HCD	83.82	729.92

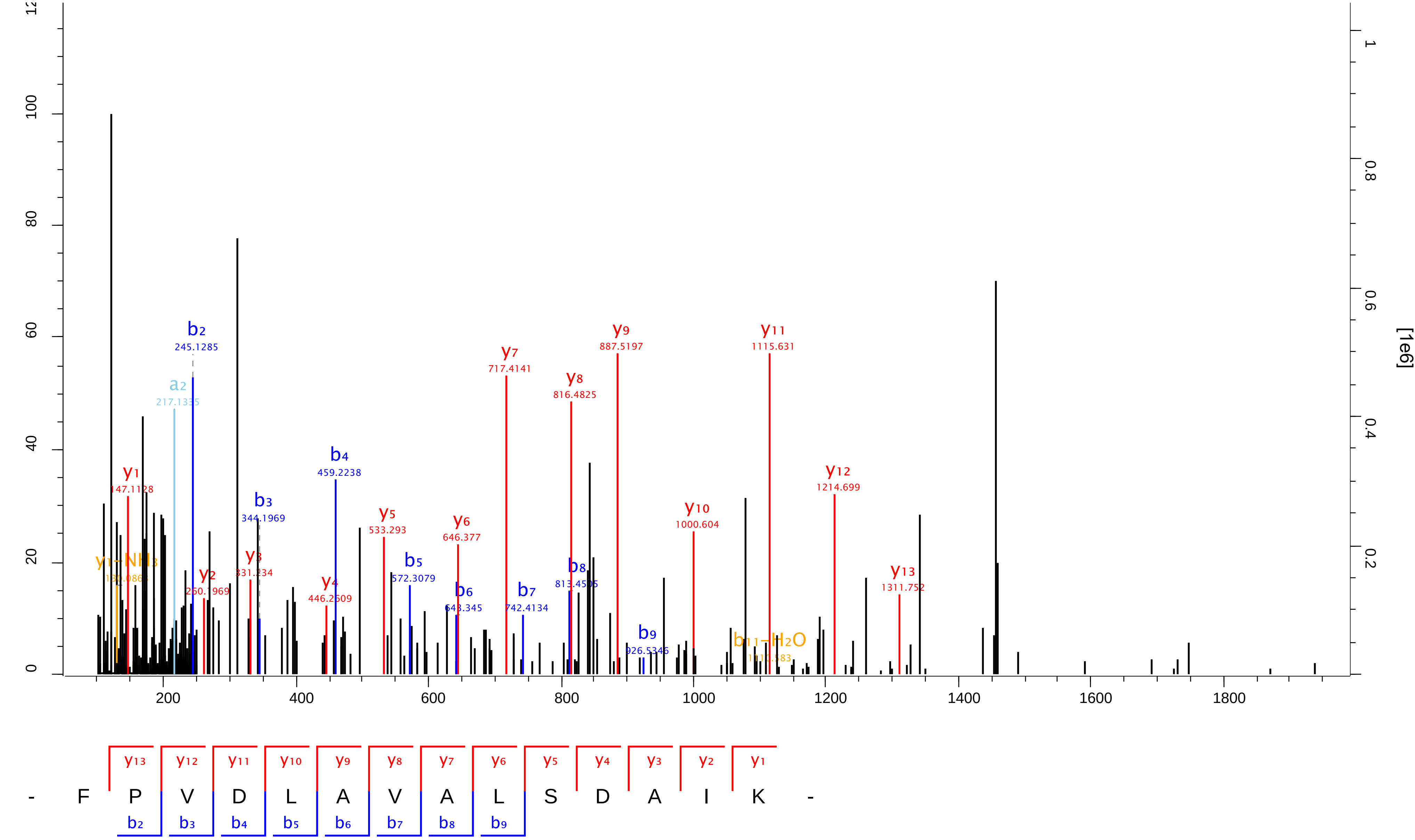

Raw file	Scan	Method	Score	m/z
QEplus003063	13858	FTMS; HCD	58.25	876.52

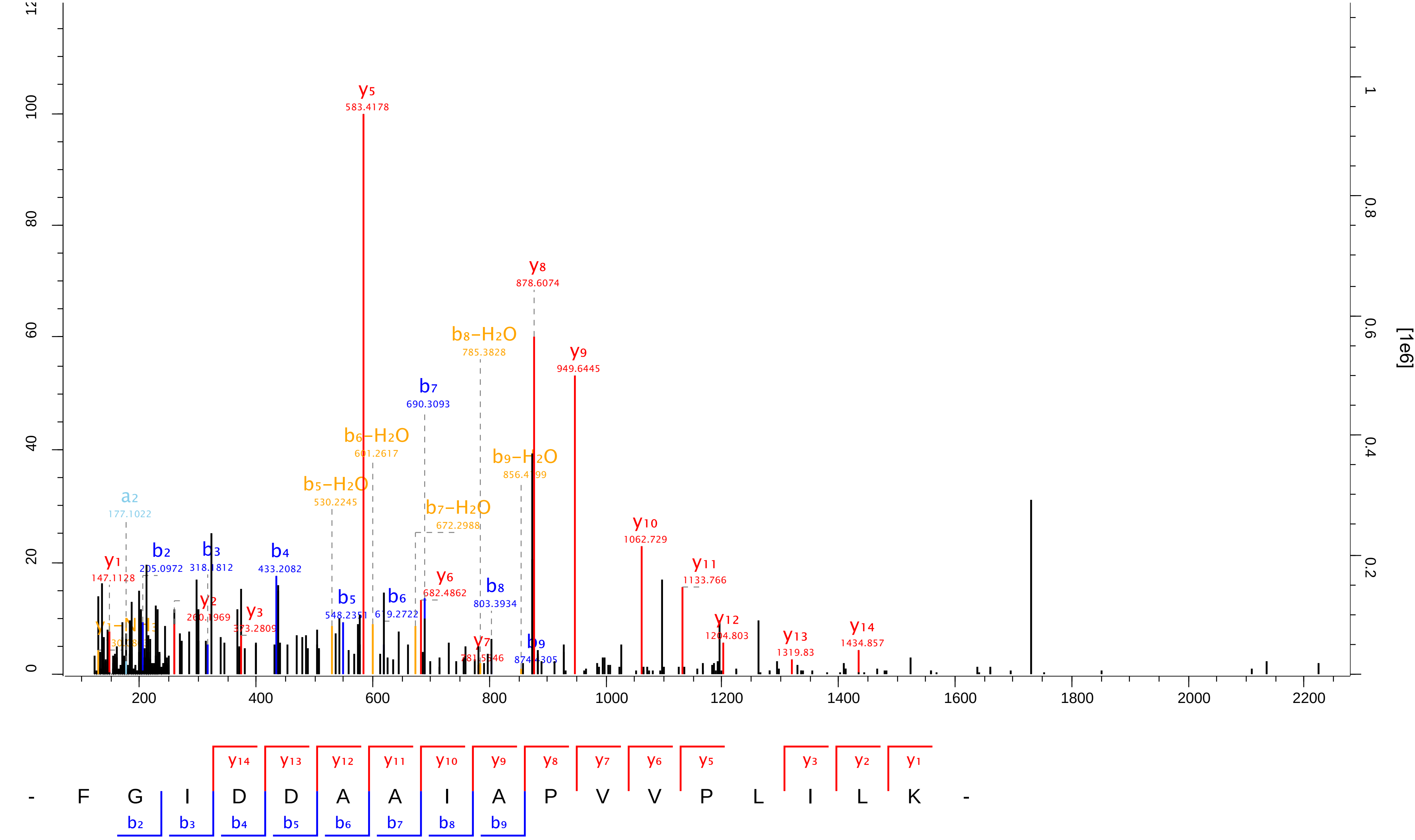

Raw file	Scan	Method	Score	m/z
QEplus003063	14951	FTMS; HCD	147.64	952.44

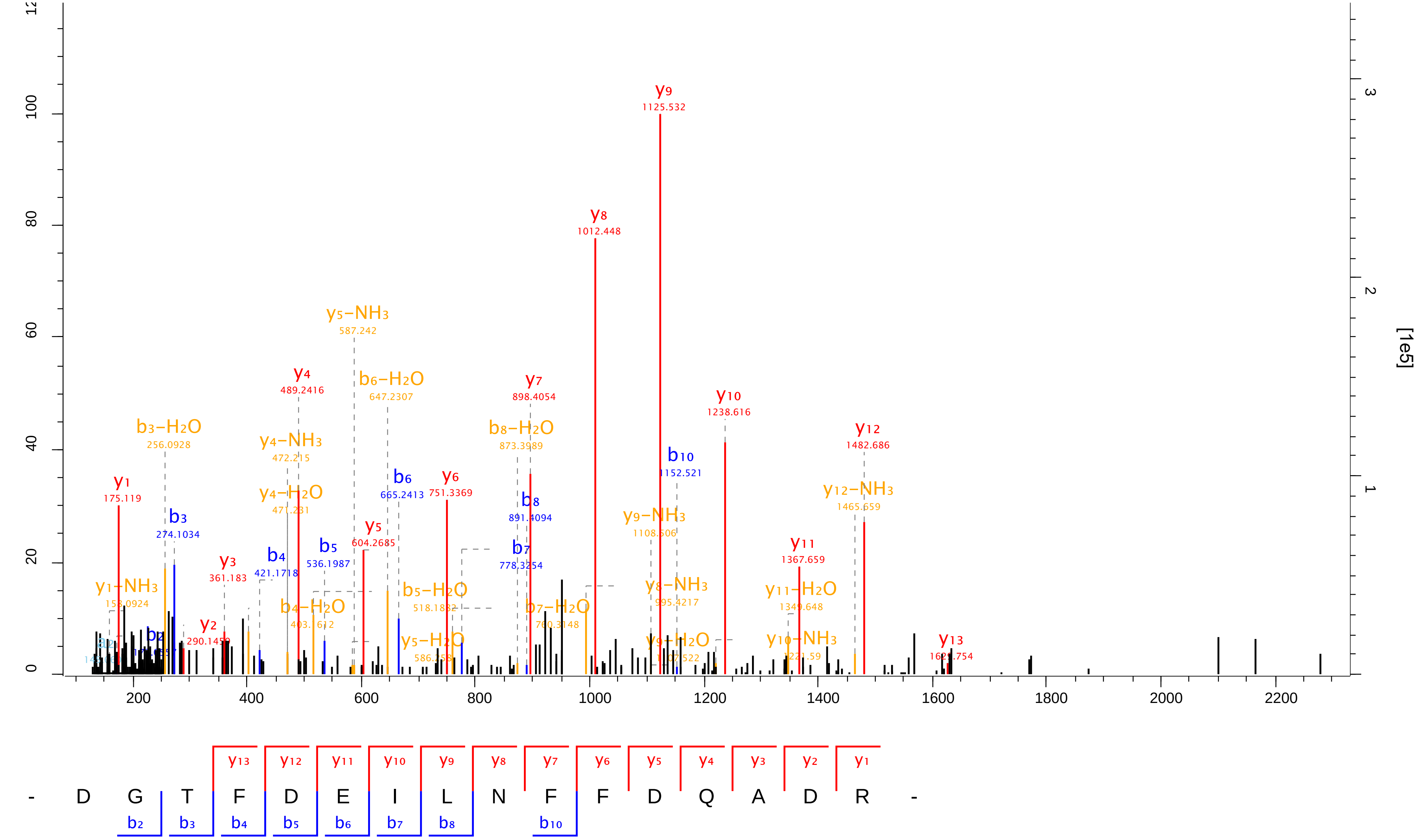

Raw file	Scan	Method	Score	m/z
QEplus003063	3048	FTMS; HCD	58.98	531.77

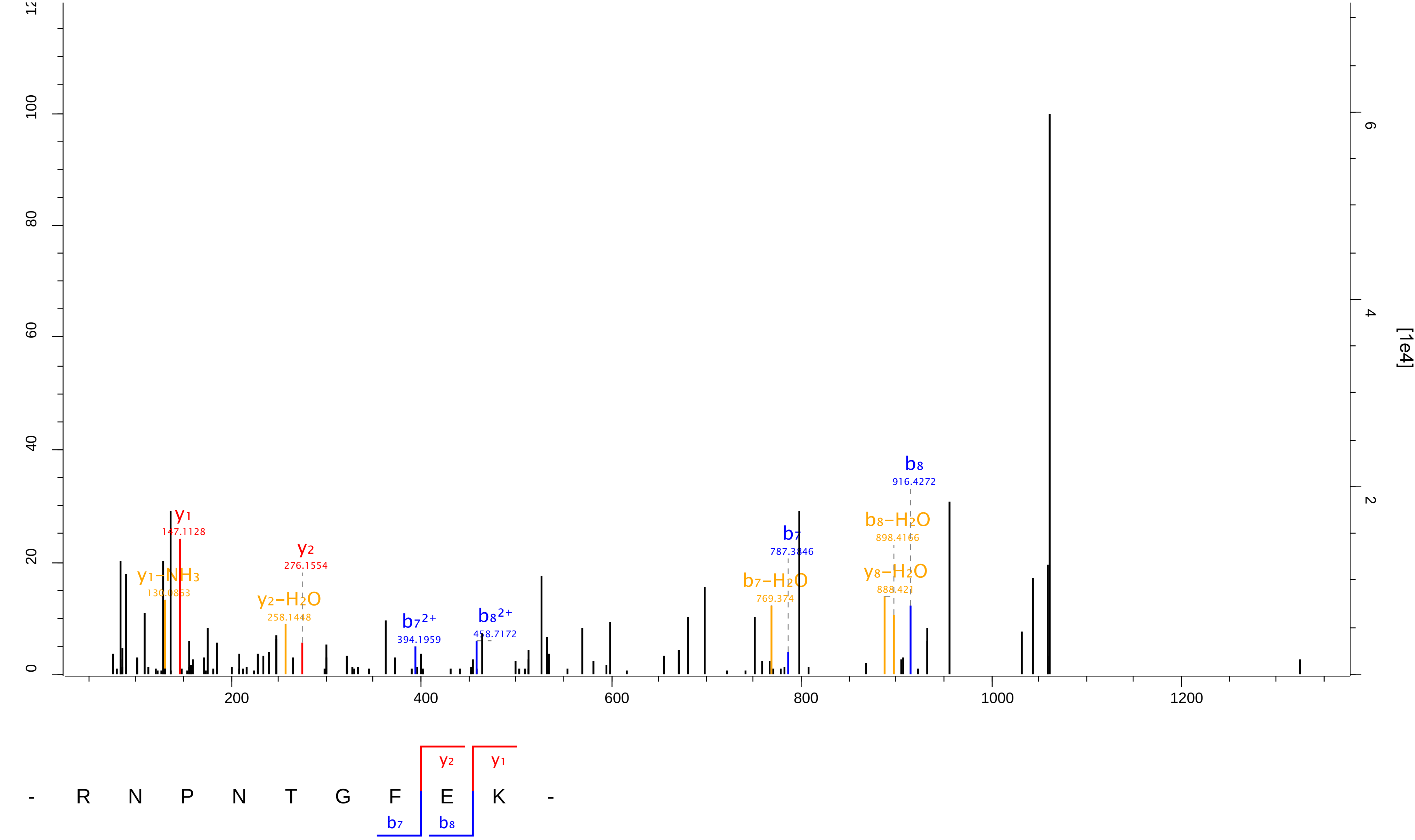

Raw file	Scan	Method	Score	m/z
QEplus003063	3678	FTMS; HCD	100.39	621.32

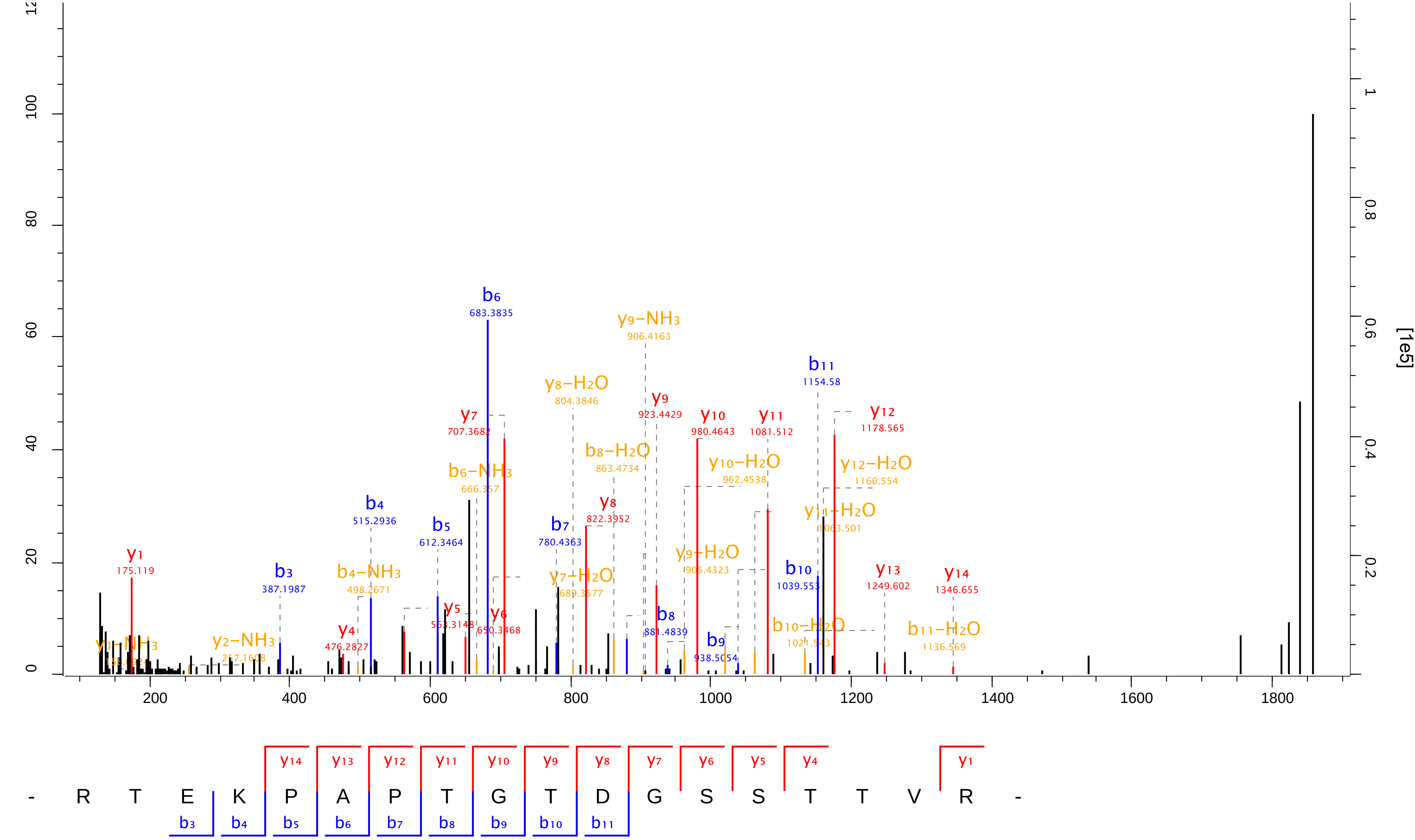

Raw file	Scan	Method	Score	m/z
QEplus003063	4193	FTMS; HCD	79.2	414.91

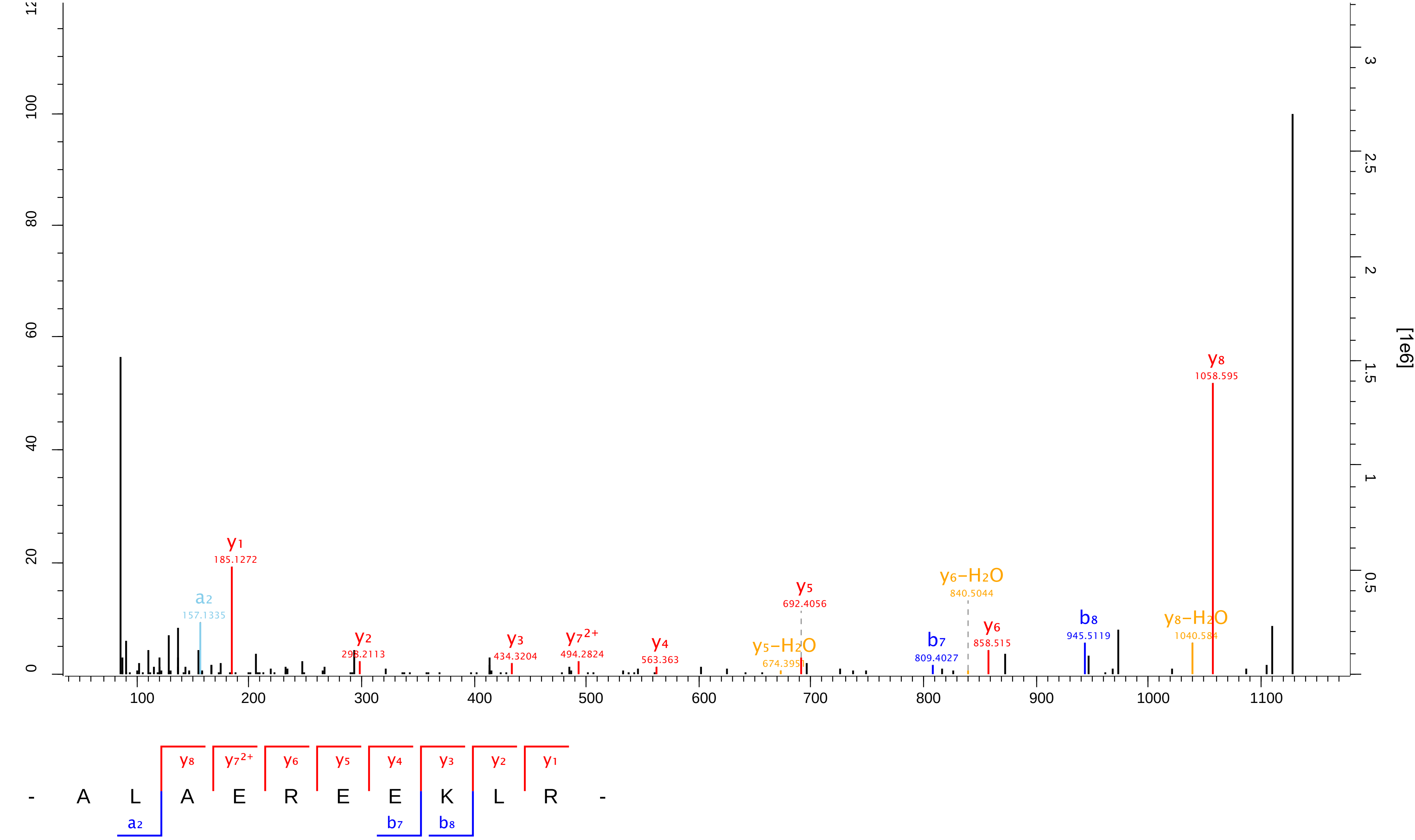

Raw file	Scan	Method	Score	m/z
QEplus003064	10411	FTMS; HCD	83.08	575.37

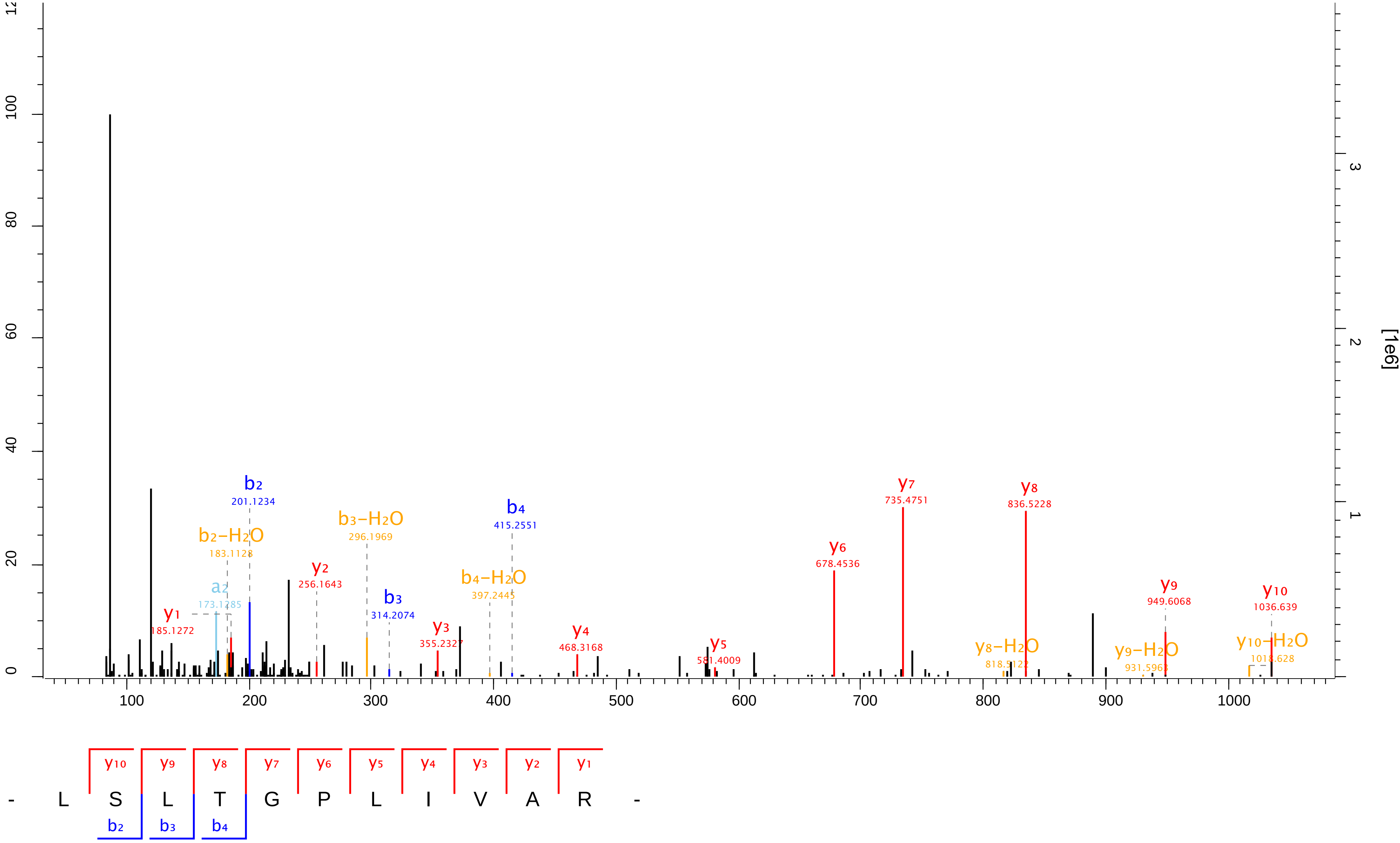

Raw file	Scan	Method	Score	m/z
QEplus003064	13111	FTMS; HCD	56.57	670.41

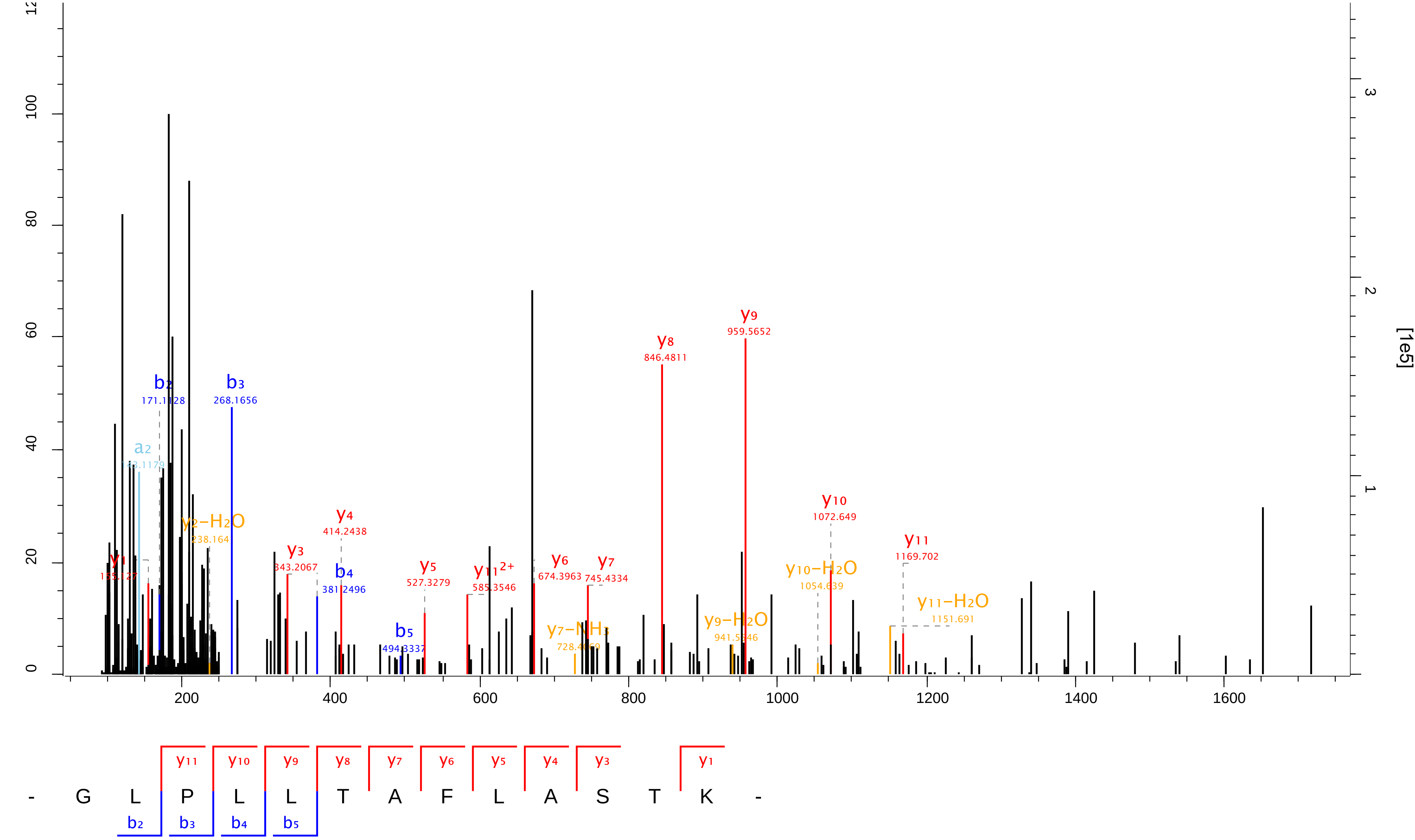

Raw file	Scan	Method	Score	m/z
QEplus003064	13217	FTMS; HCD	71.08	1001.53

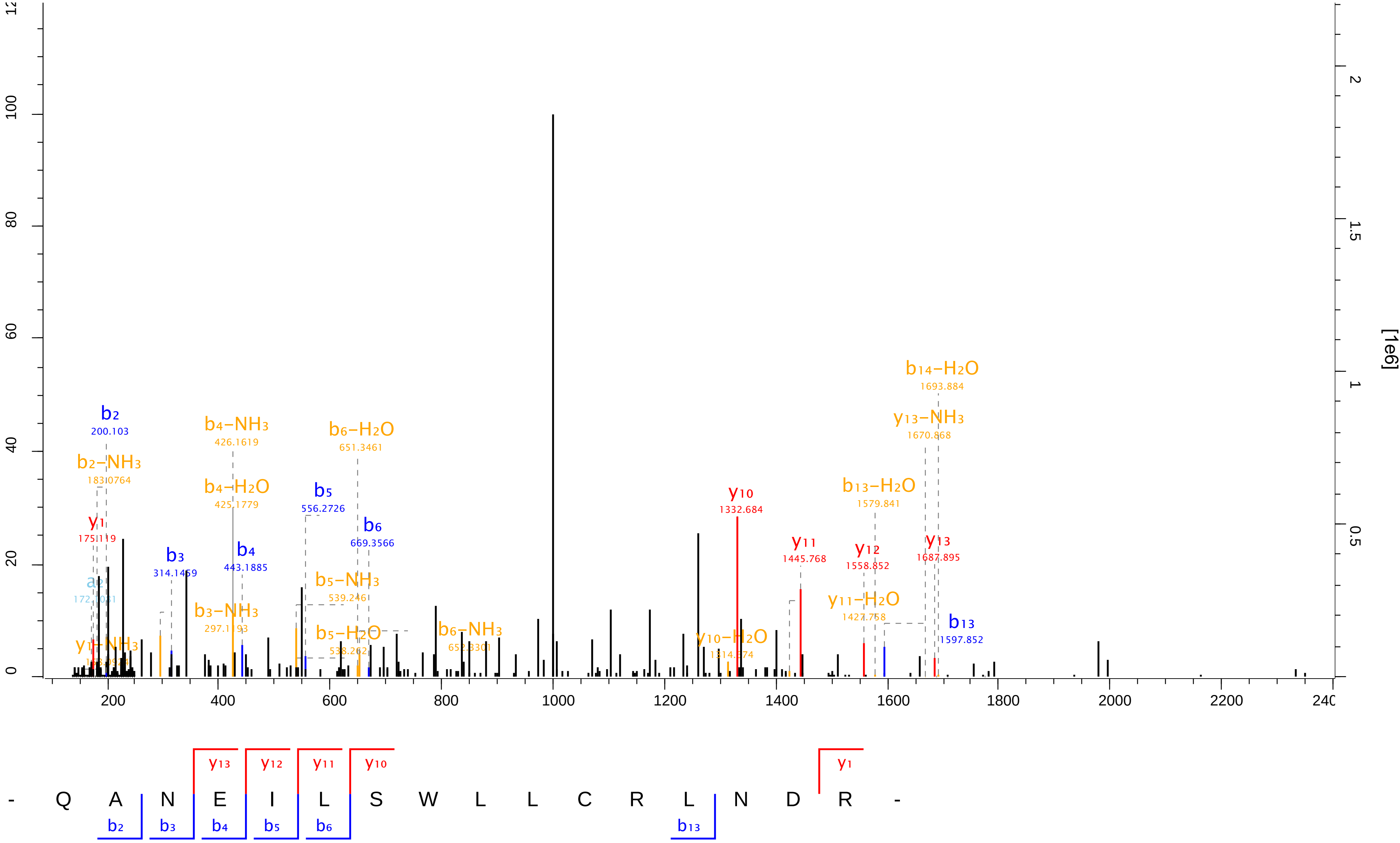

Raw file	Scan	Method	Score	m/z
QEplus003064	14169	FTMS; HCD	46.89	832.94

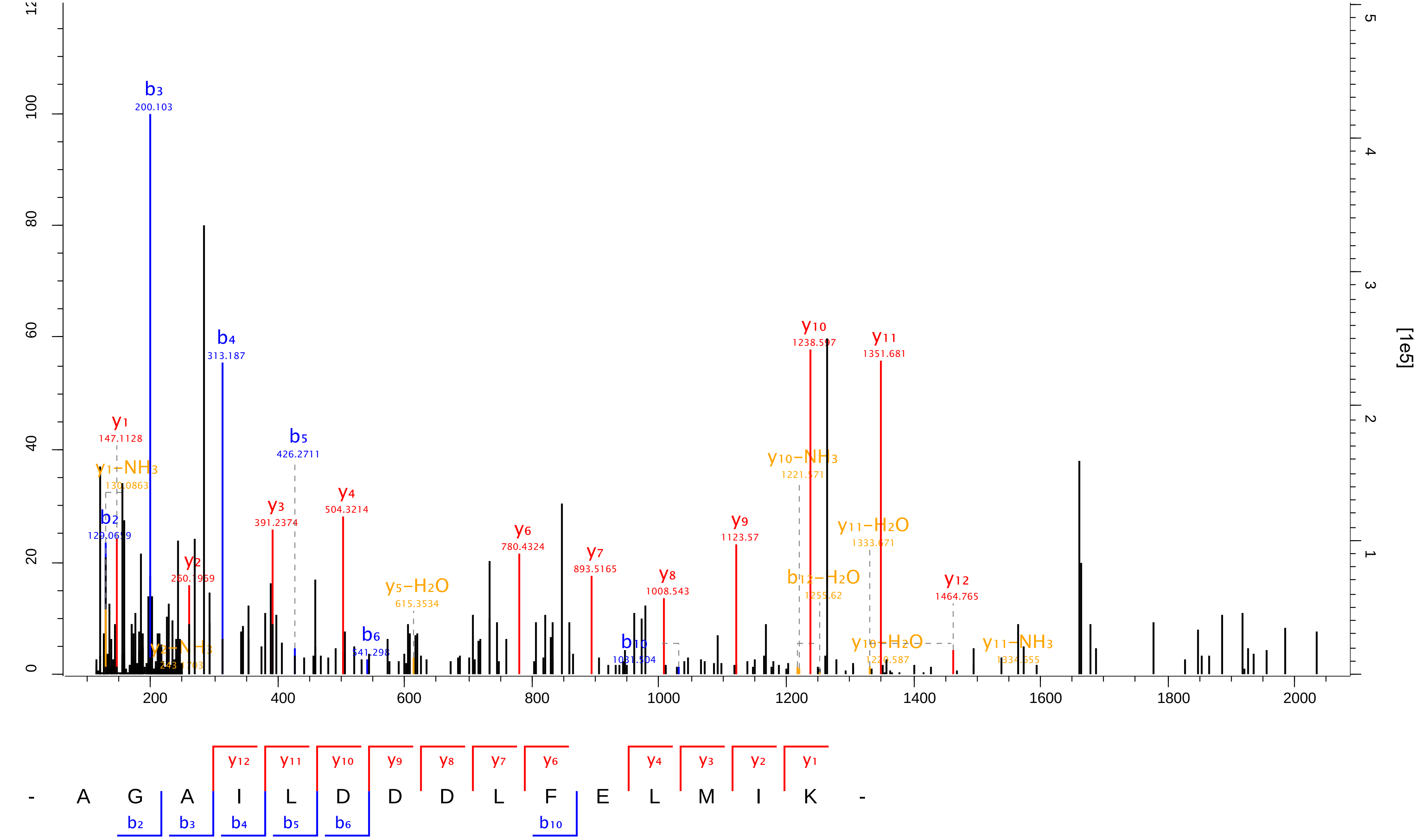

Raw file	Scan	Method	Score	m/z
QEplus003064	14896	FTMS; HCD	125.73	1034.1

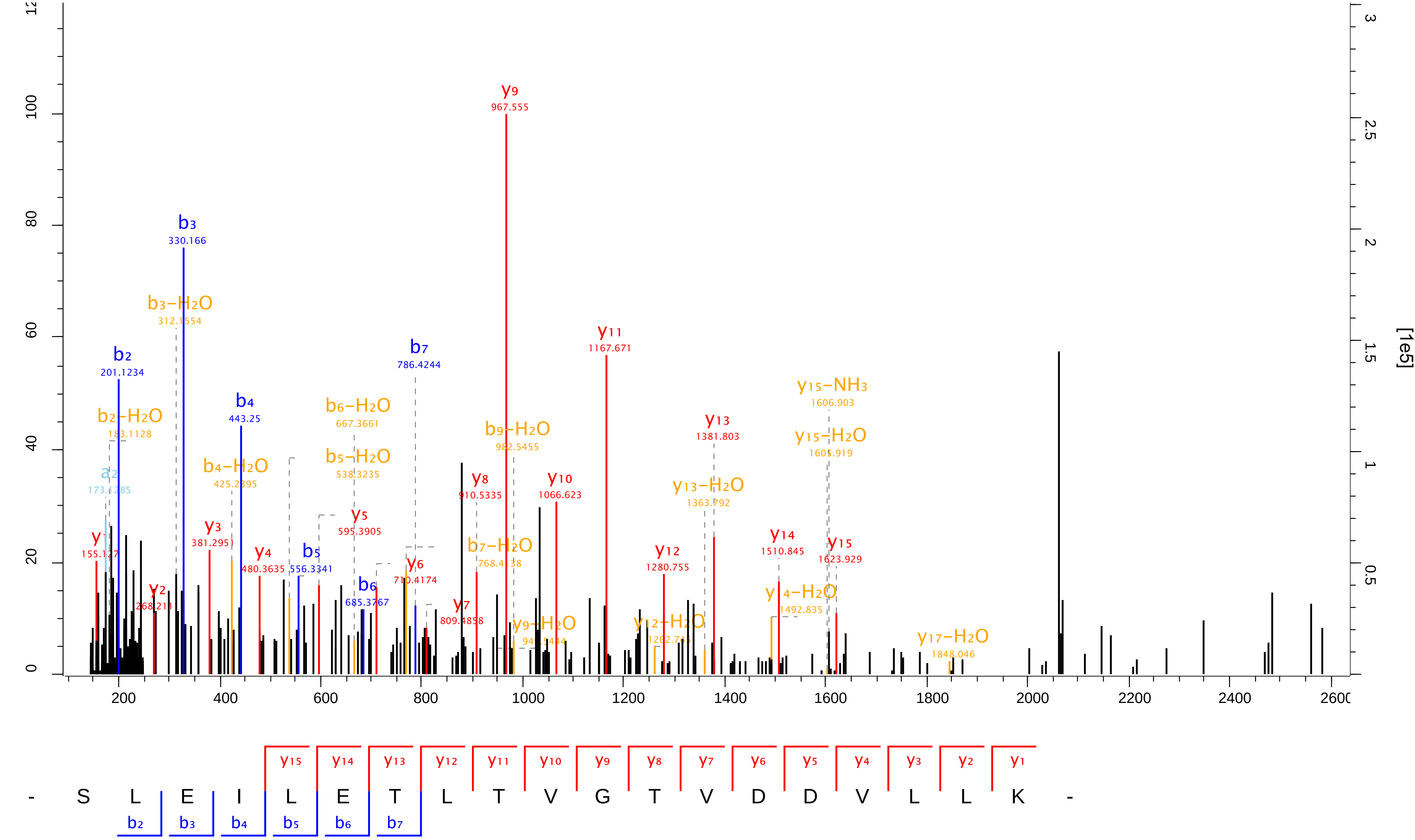

Raw file	Scan	Method	Score	m/z
QEplus003064	6422	FTMS; HCD	87.64	473.25

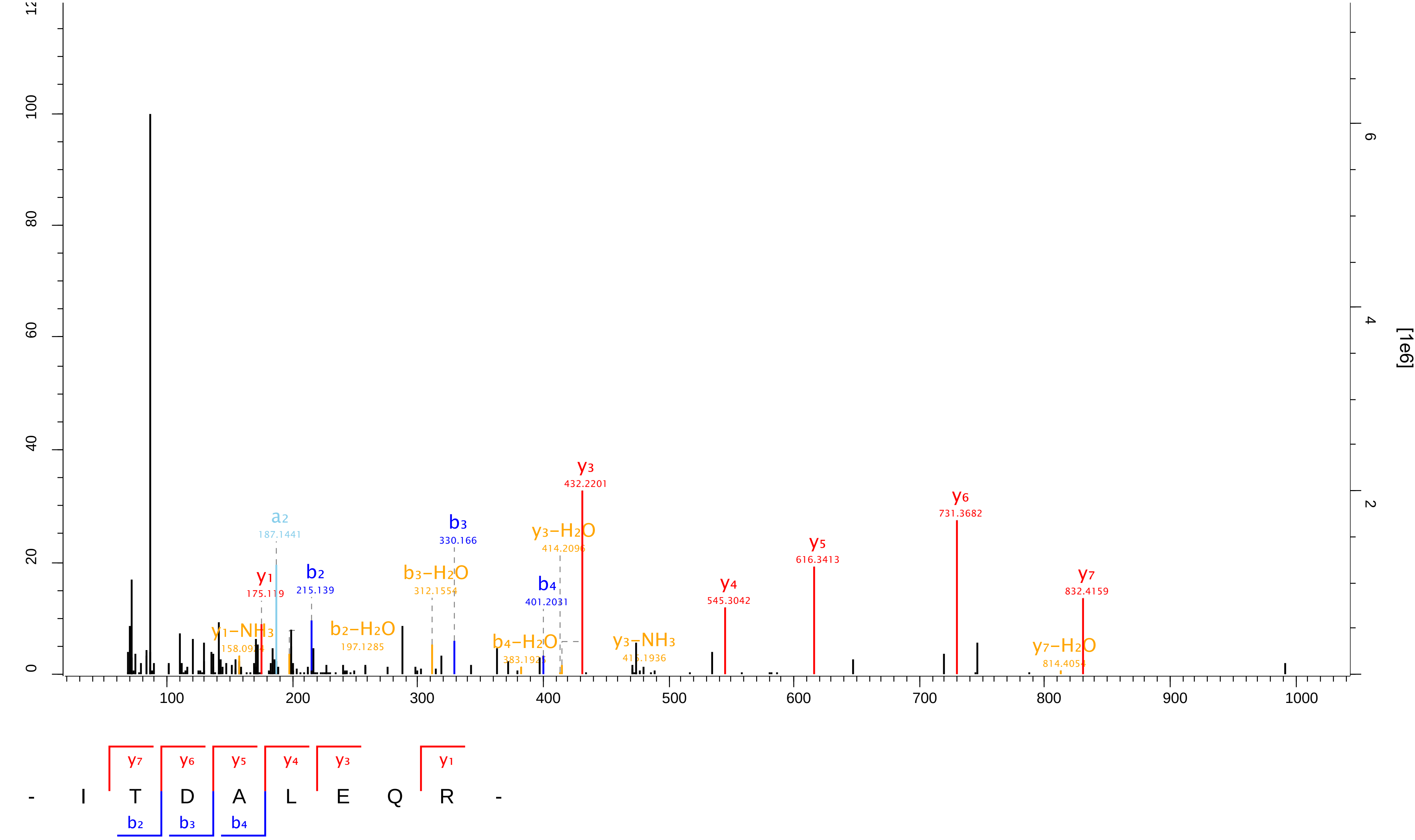

Raw file	Scan	Method	Score	m/z
QEplus003065	10560	FTMS; HCD	95.35	496.29

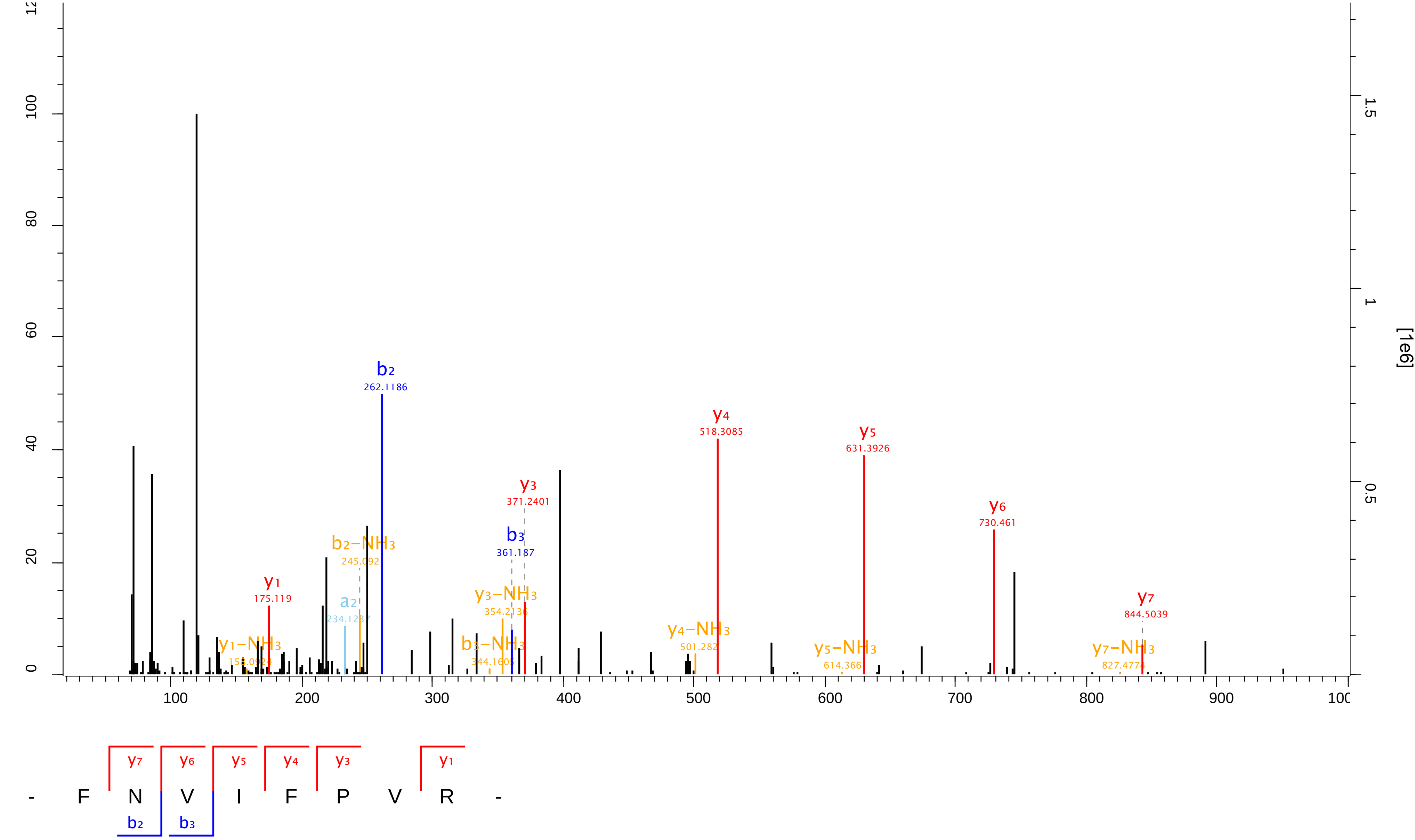

Raw file	Scan	Method	Score	m/z
QEplus003065	10750	FTMS; HCD	57.94	685.05

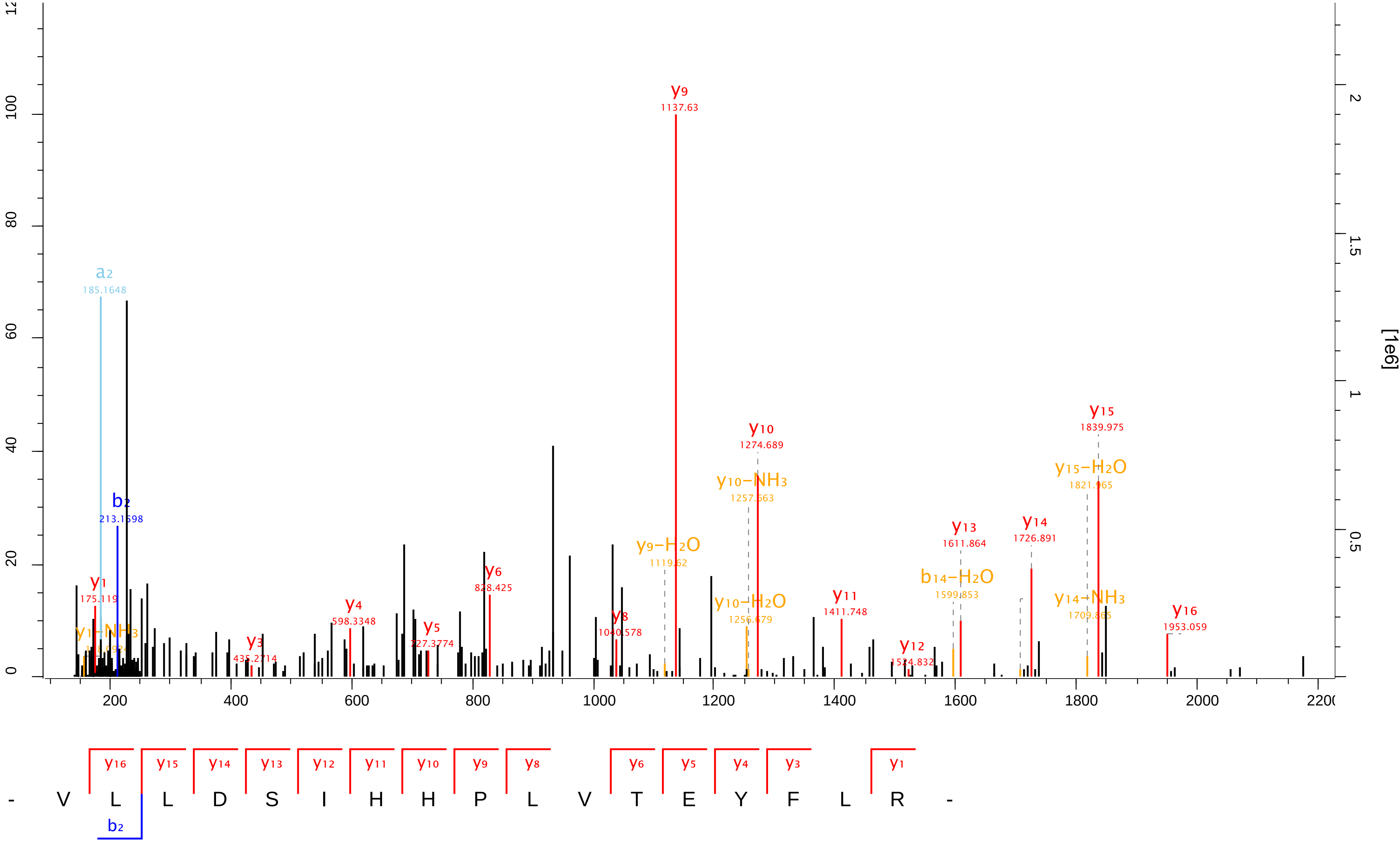

Raw file	Scan	Method	Score	m/z
QEplus003065	13502	FTMS; HCD	64.12	692.85

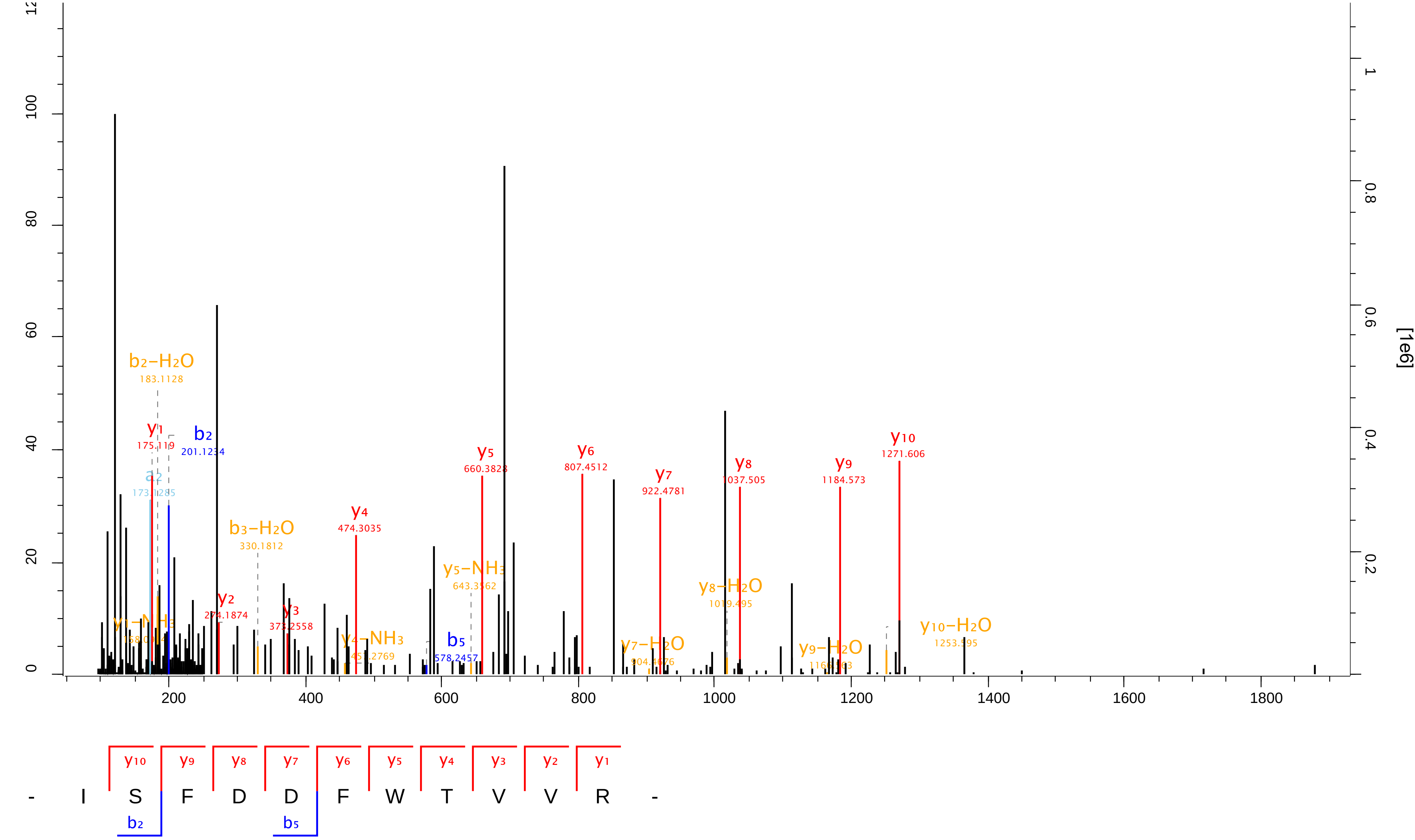

Raw file	Scan	Method	Score	m/z
QEplus003065	8128	FTMS; HCD	52.17	898.46

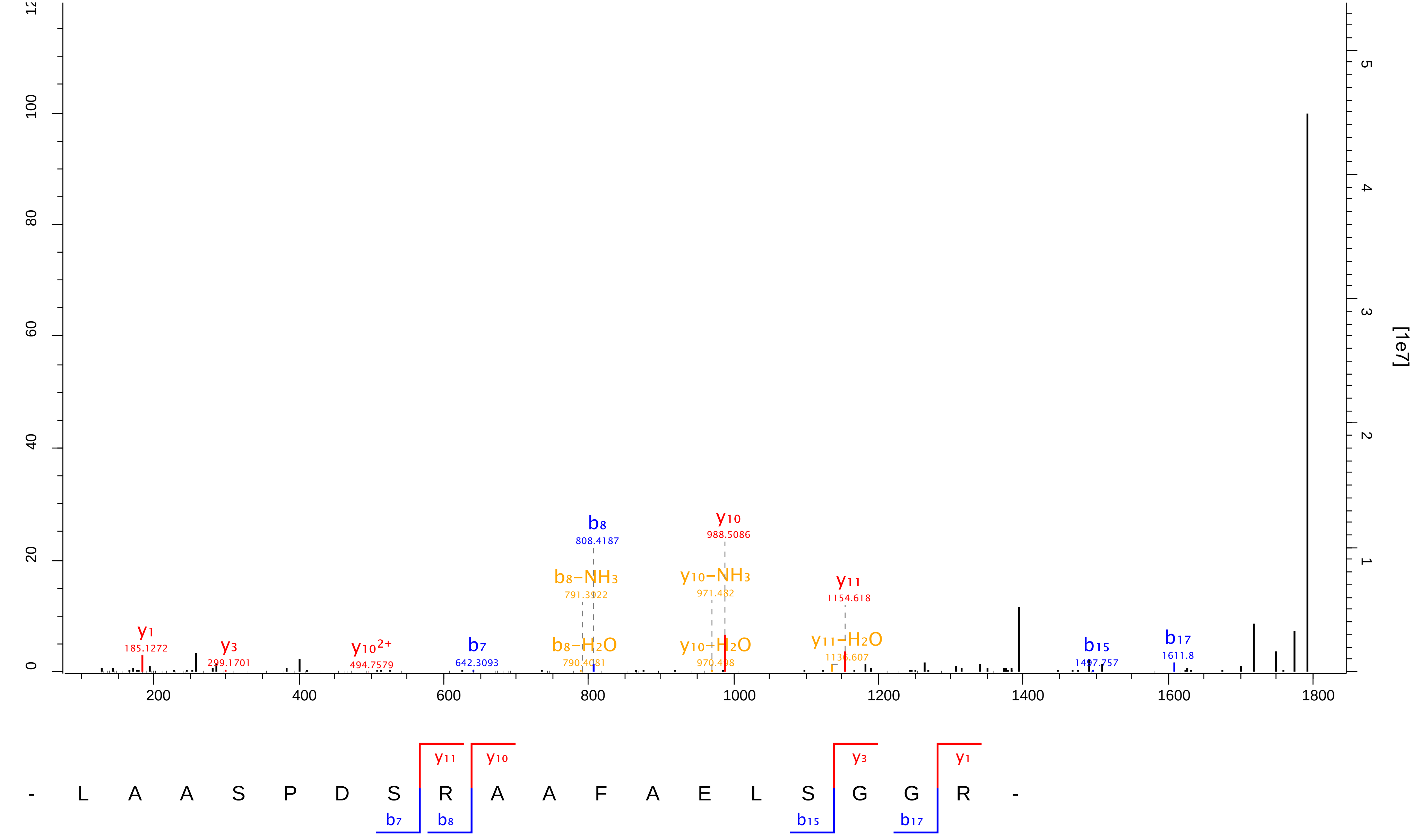

Raw file	Scan	Method	Score	m/z
QEplus003066	10409	FTMS; HCD	79.06	542.32

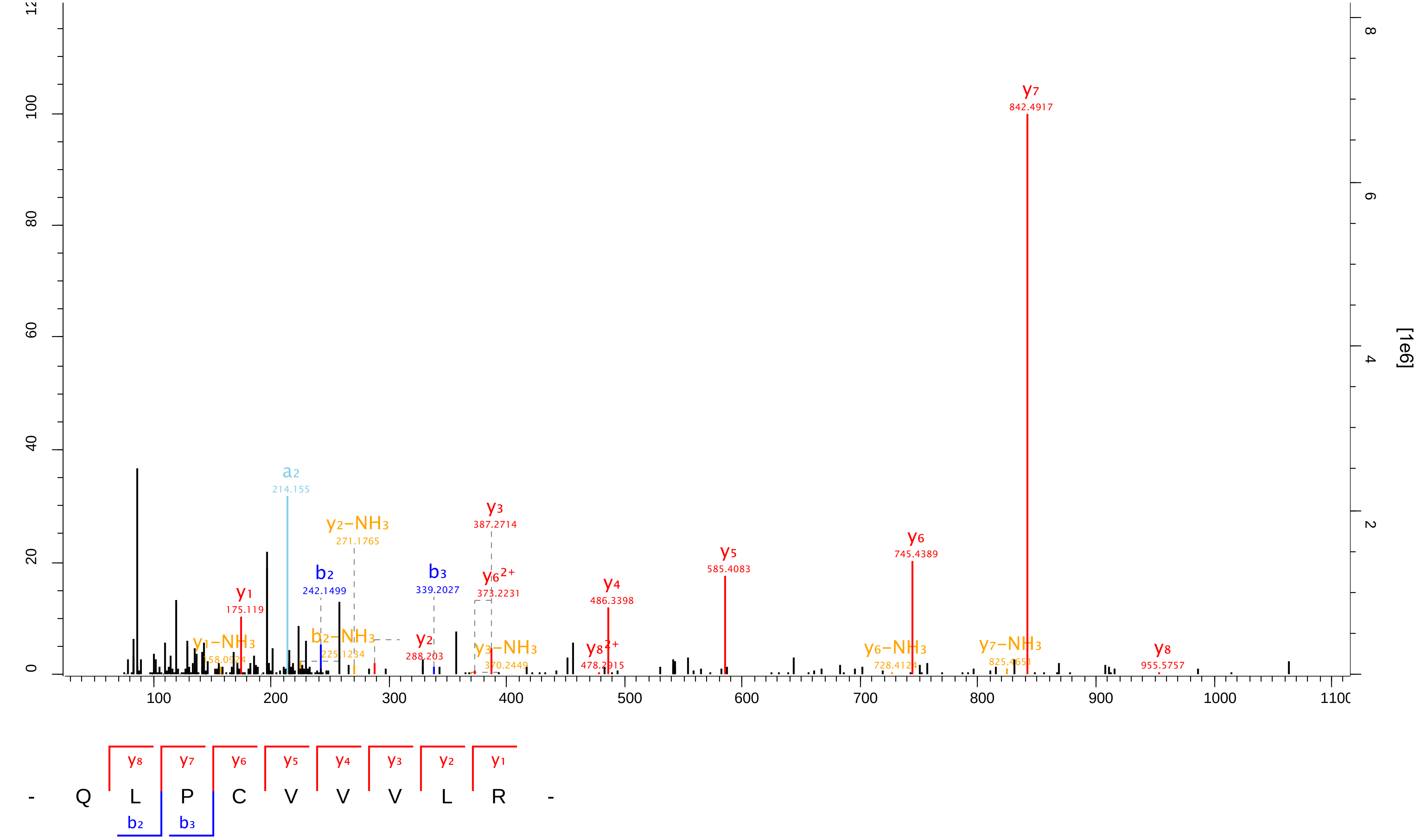

Raw file	Scan	Method	Score	m/z
QEplus003066	10739	FTMS; HCD	104.2	523.31

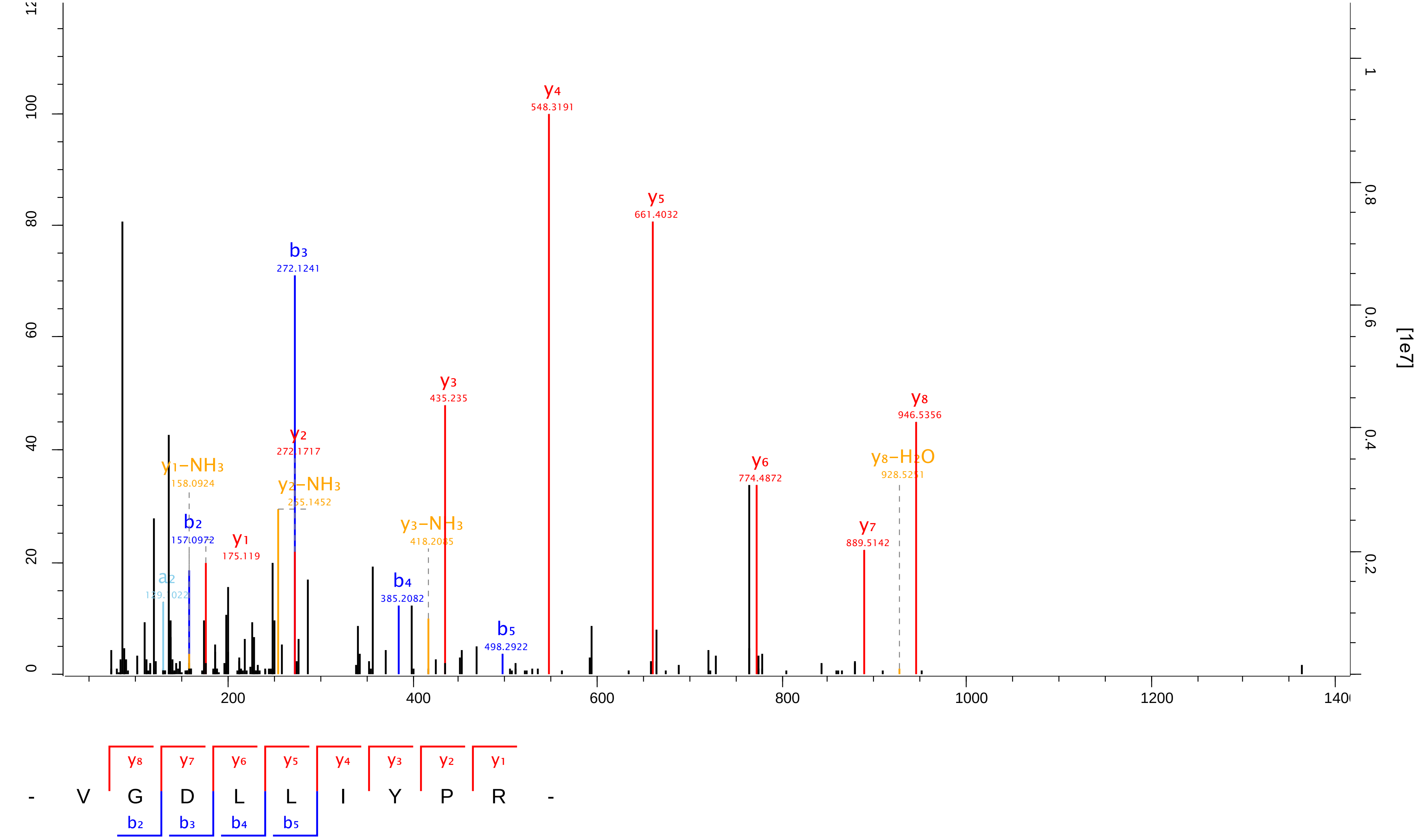

Raw file	Scan	Method	Score	m/z
QEplus003066	11970	FTMS; HCD	95.93	1050.06

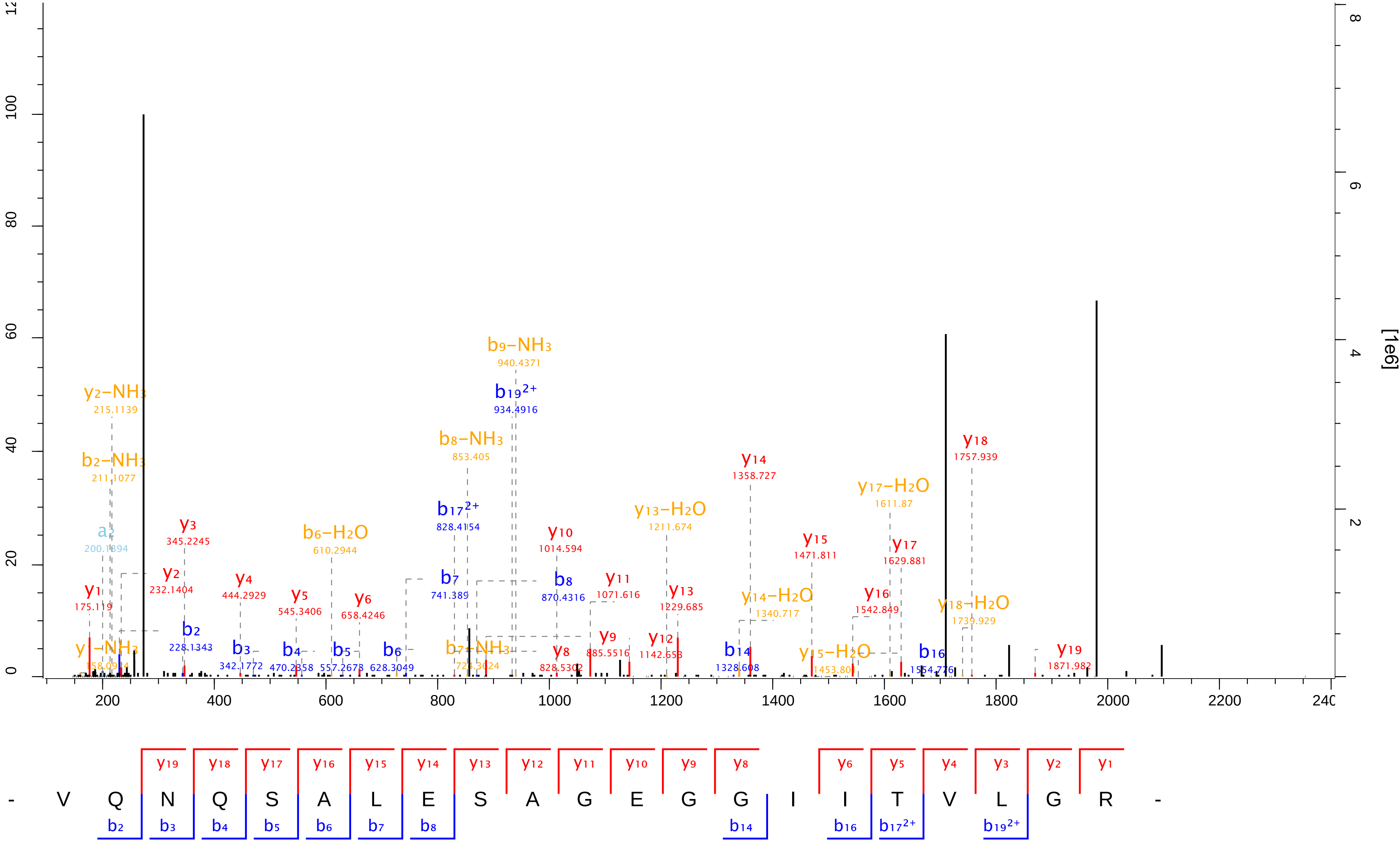

Raw file	Scan	Method	Score	m/z
QEplus003066	12200	FTMS; HCD	77.64	778.46

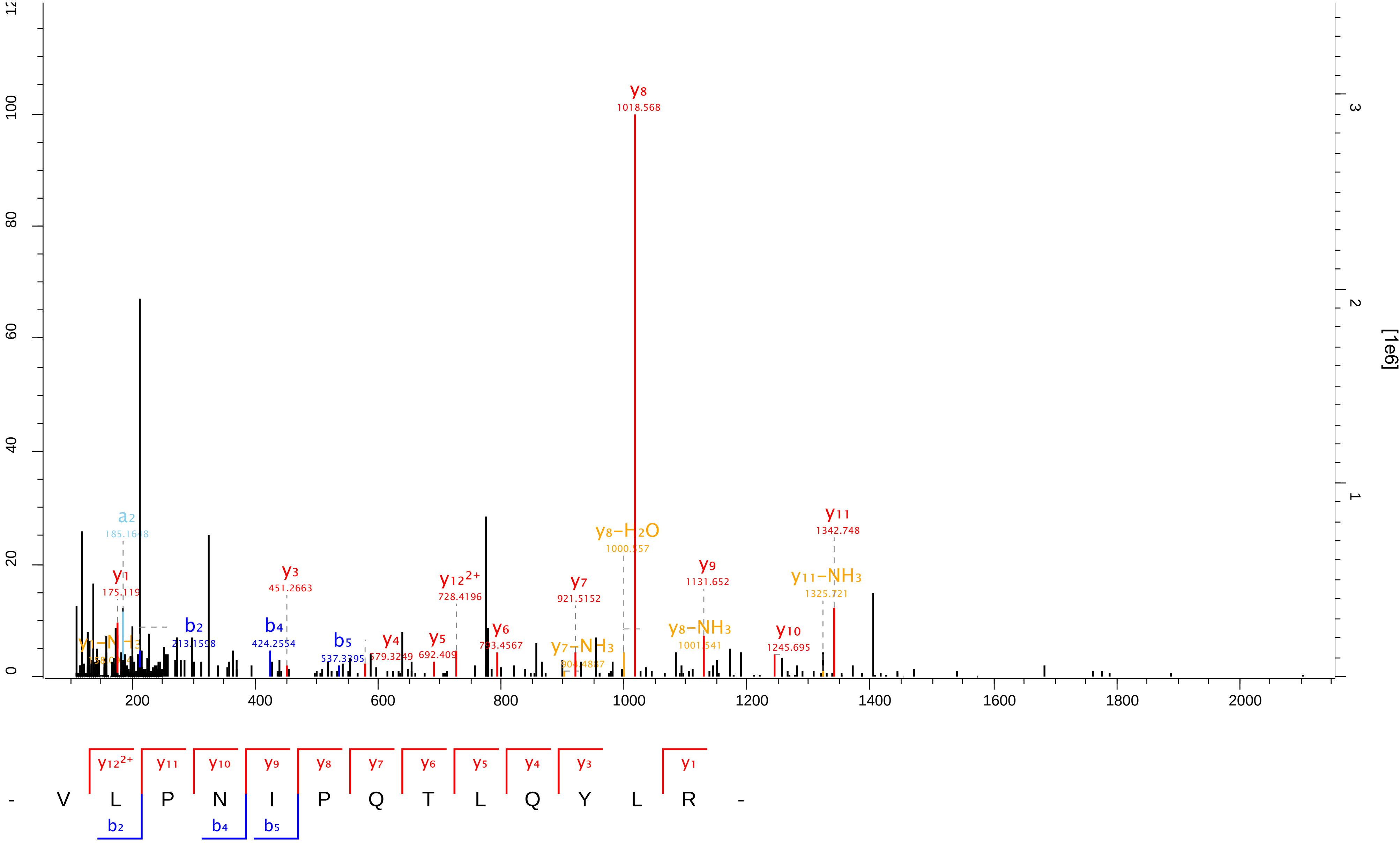

Raw file	Scan	Method	Score	m/z
QEplus003066	13403	FTMS; HCD	76.34	808.98

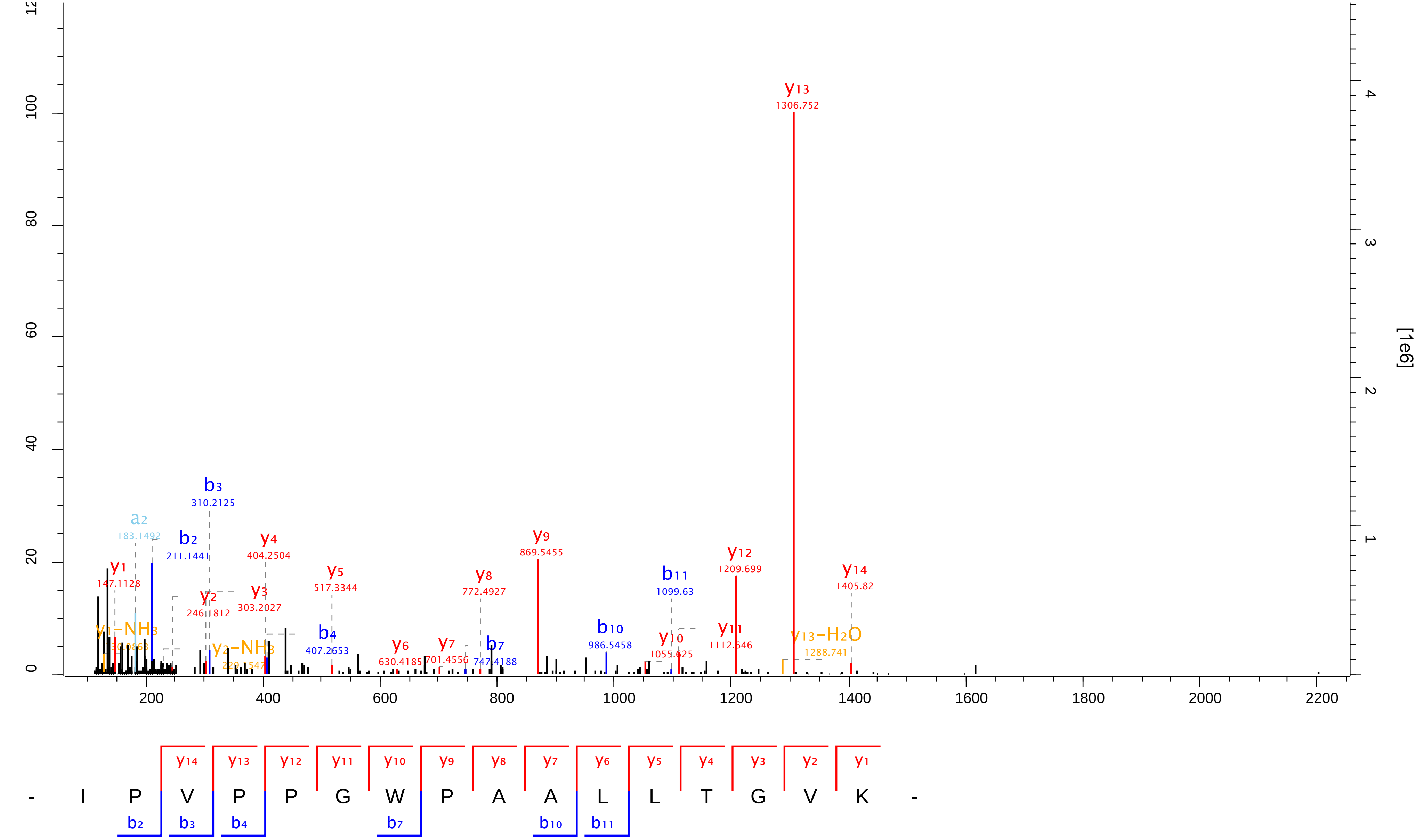

Raw file	Scan	Method	Score	m/z
QEplus003066	14714	FTMS; HCD	72.89	804.42

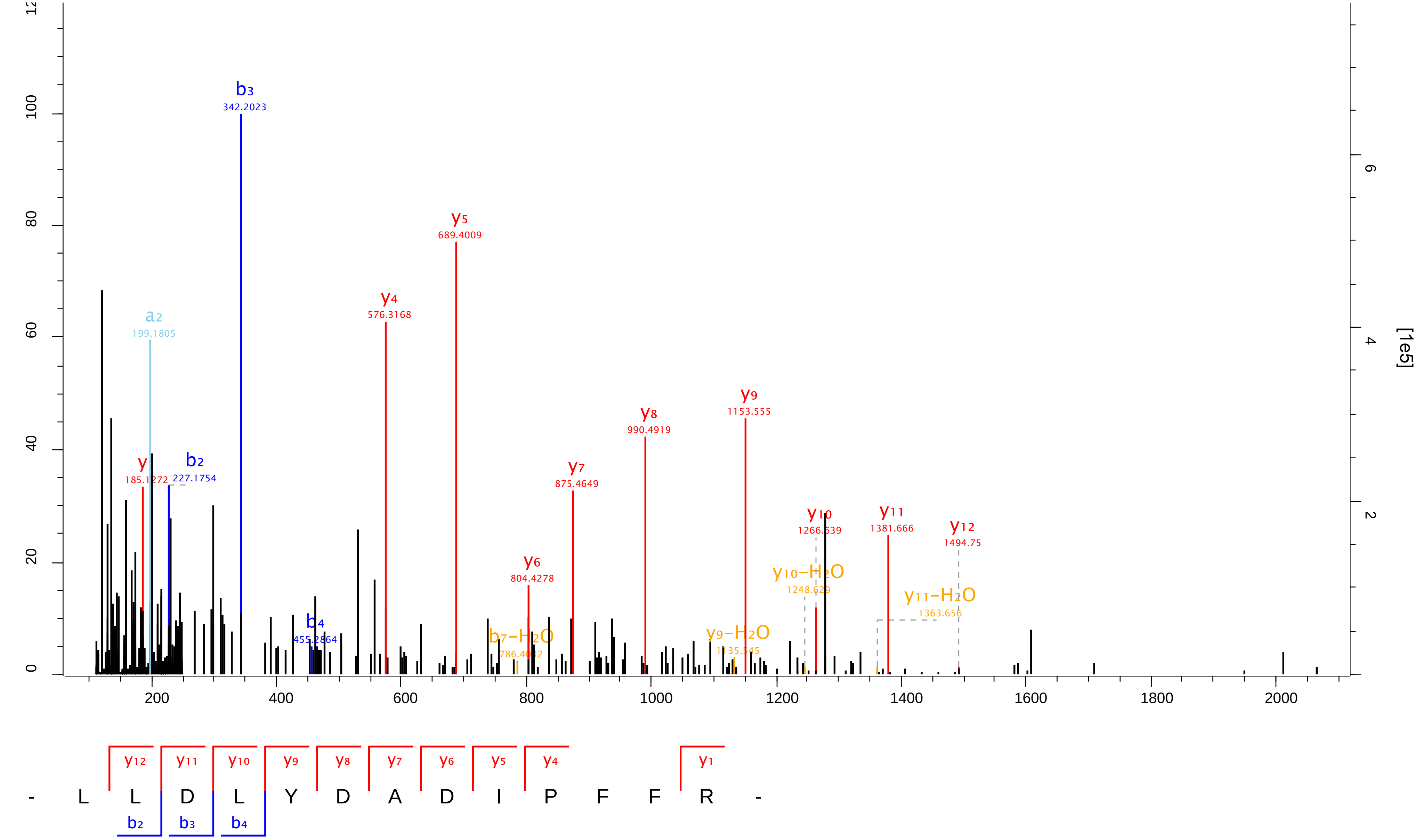

Raw file	Scan	Method	Score	m/z
QEplus003066	15127	FTMS; HCD	81.62	610.34

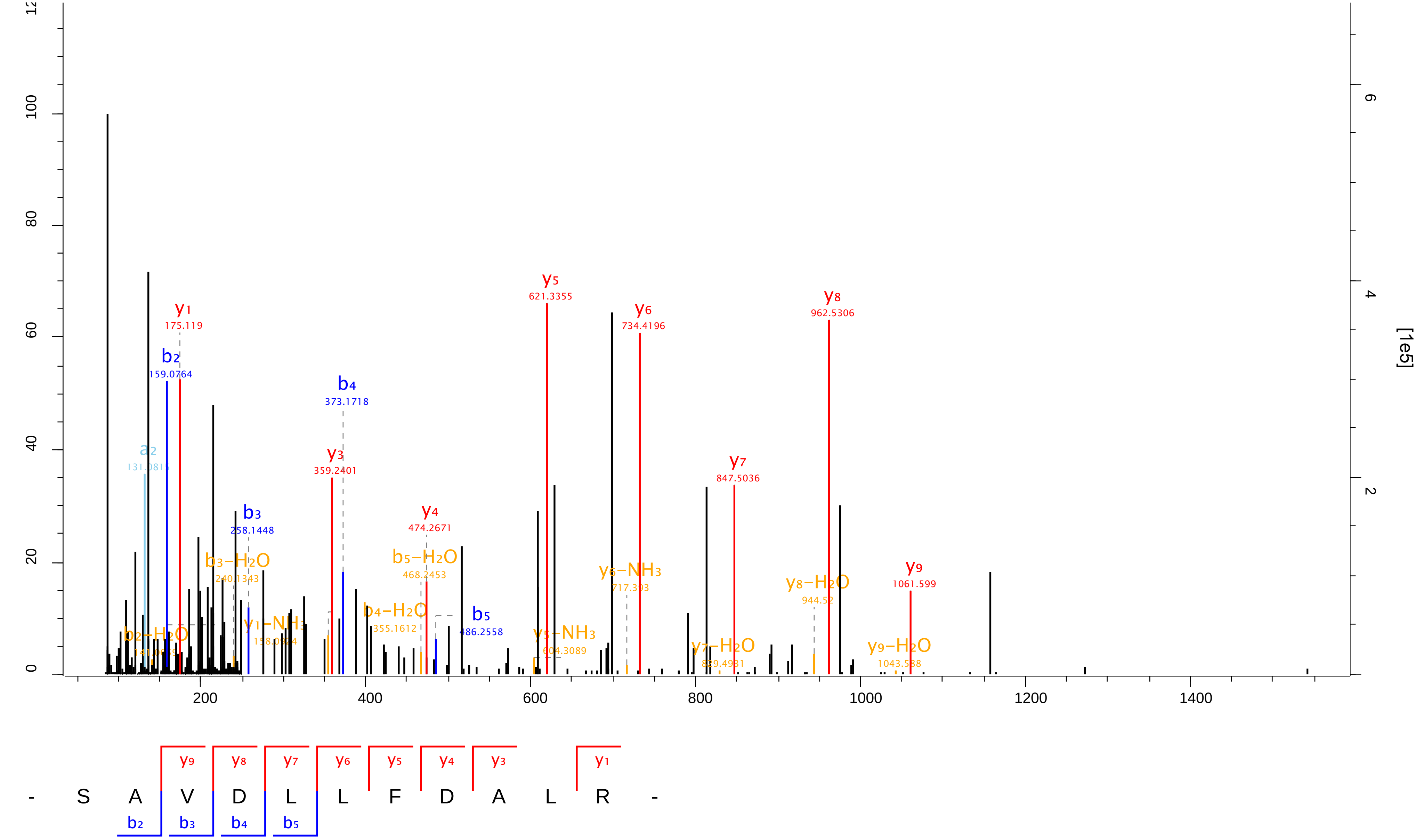

Raw file	Scan	Method	Score	m/z
QEplus003066	15533	FTMS; HCD	121.54	662.06

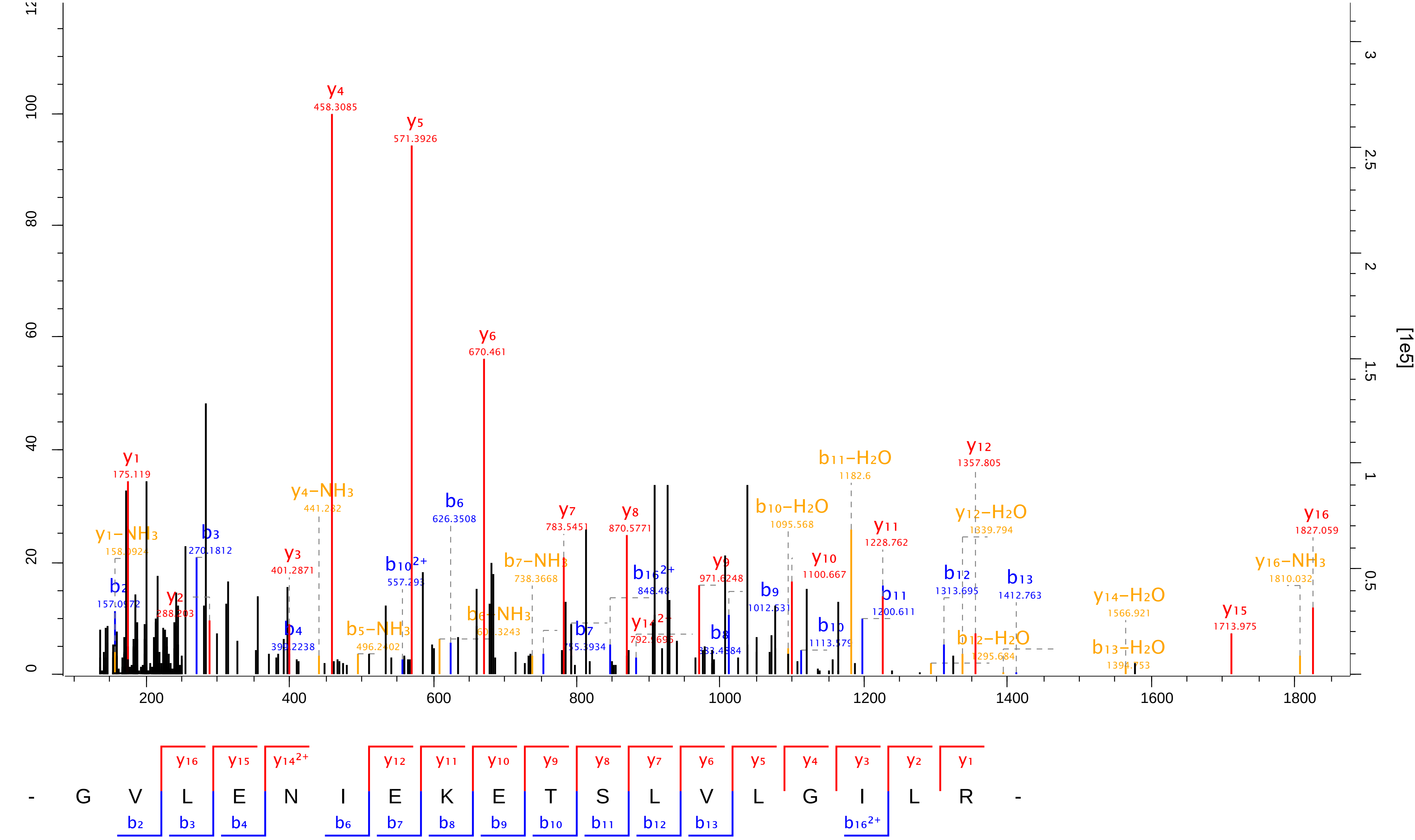

Raw file	Scan	Method	Score	m/z
QEplus003066	4733	FTMS; HCD	101.39	666.35

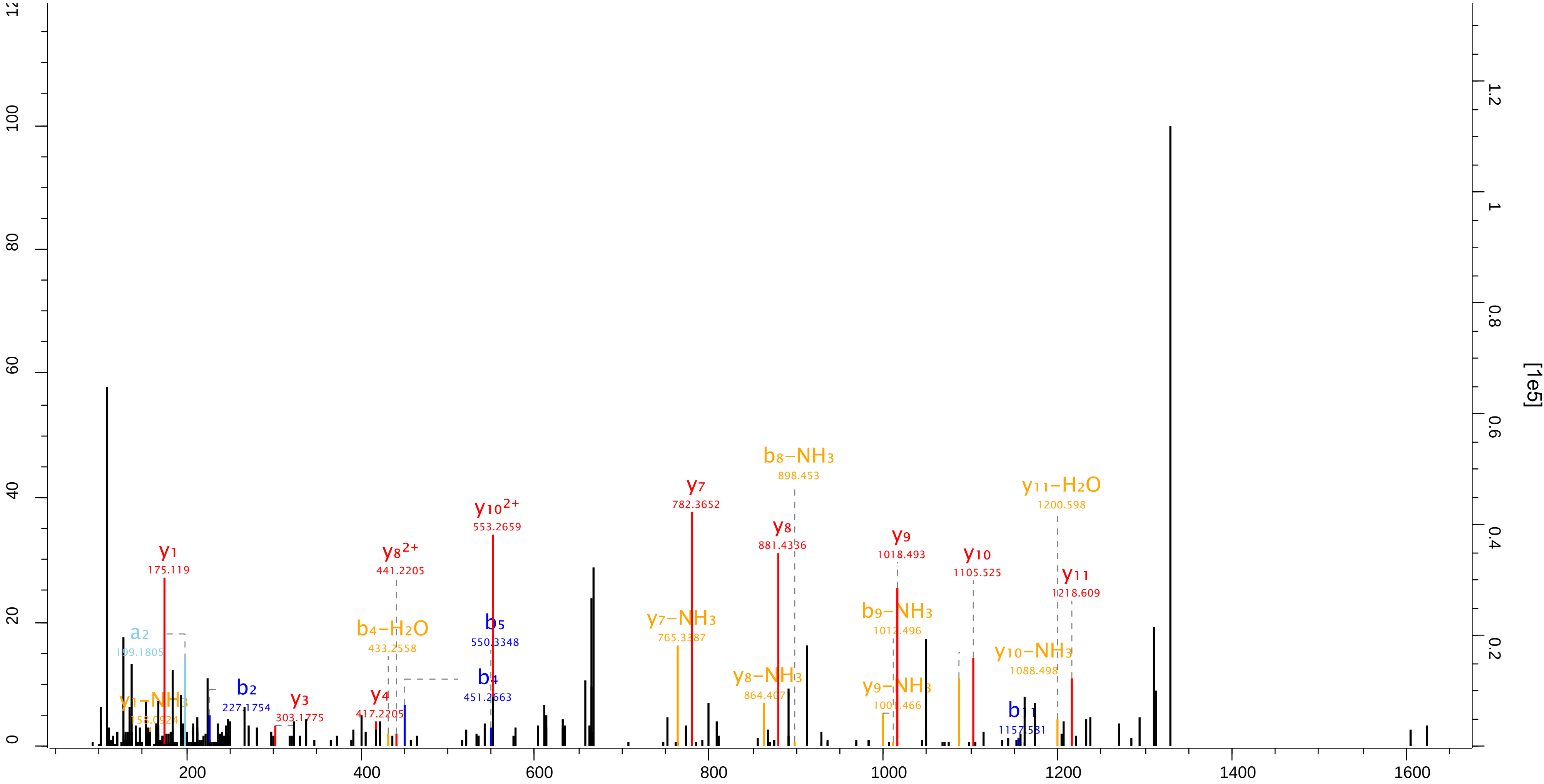

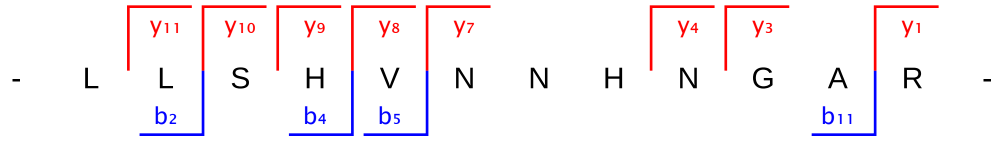

Raw file	Scan	Method	Score	m/z
QEplus003067	11828	FTMS; HCD	74.99	497.94

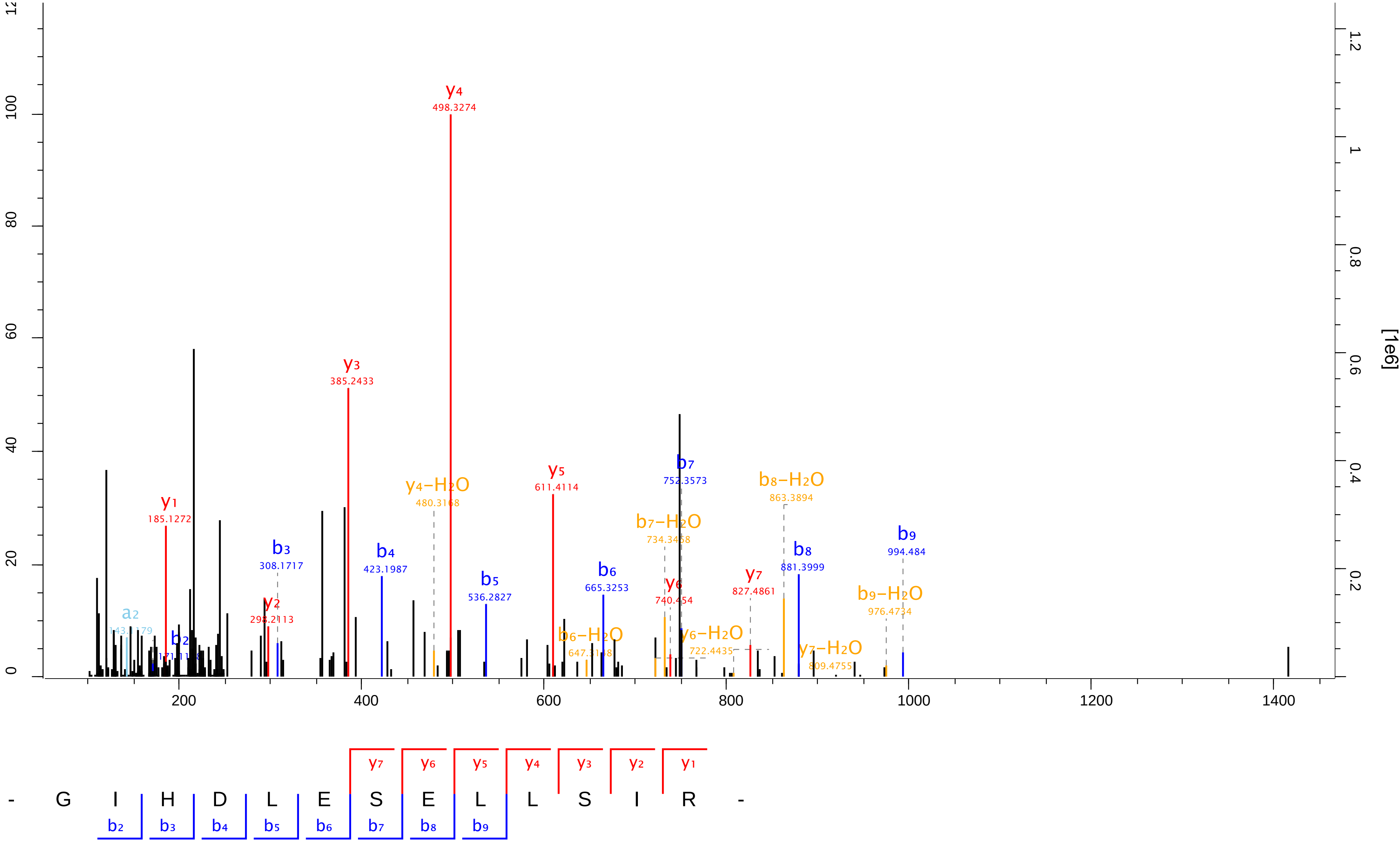

Raw file	Scan	Method	Score	m/z
QEplus003067	12655	FTMS; HCD	53.17	820.96

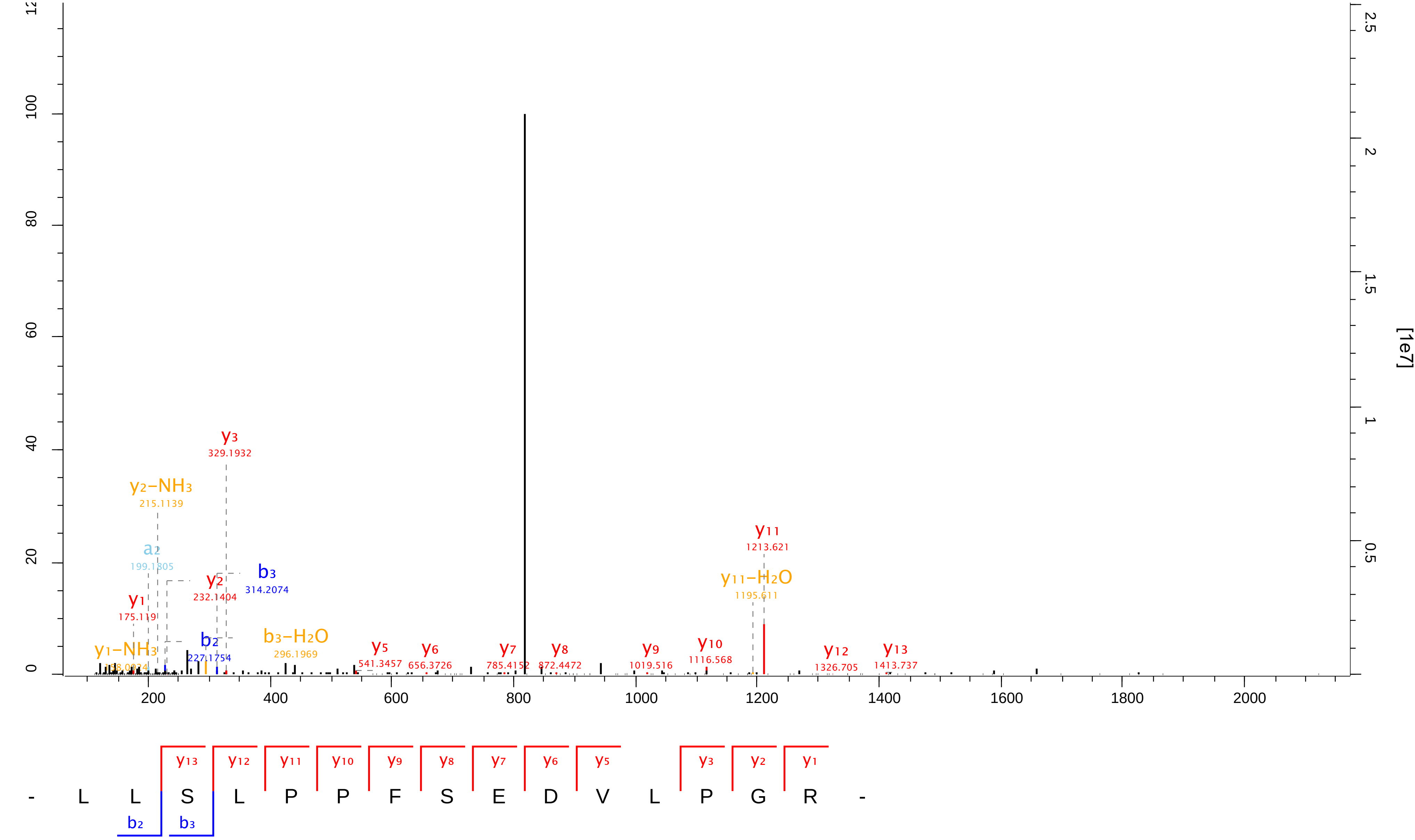

Raw file	Scan	Method	Score	m/z
QEplus003067	14003	FTMS; HCD	66.98	938.53

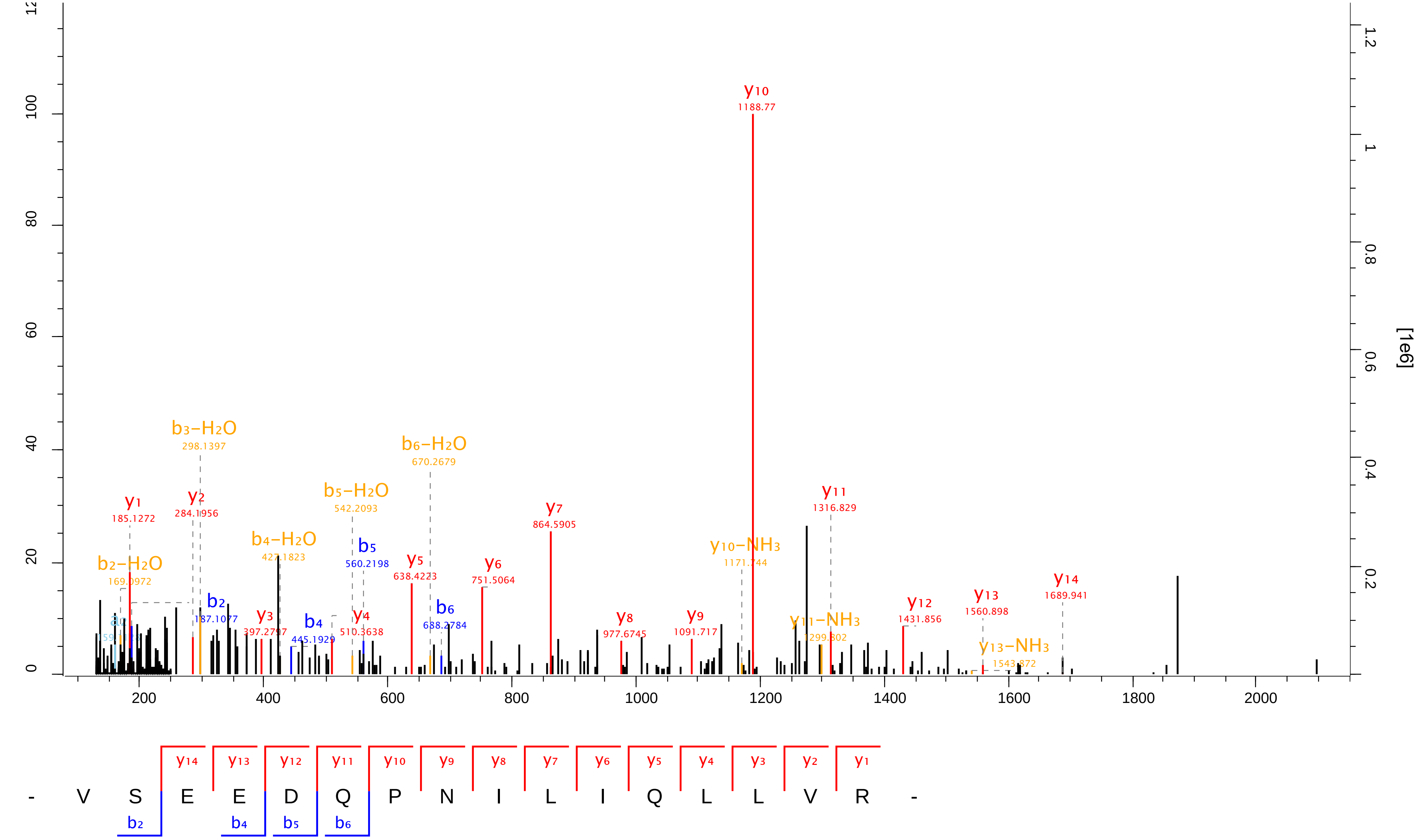

Raw file	Scan	Method	Score	m/z
QEplus003067	14027	FTMS; HCD	85.52	799.03

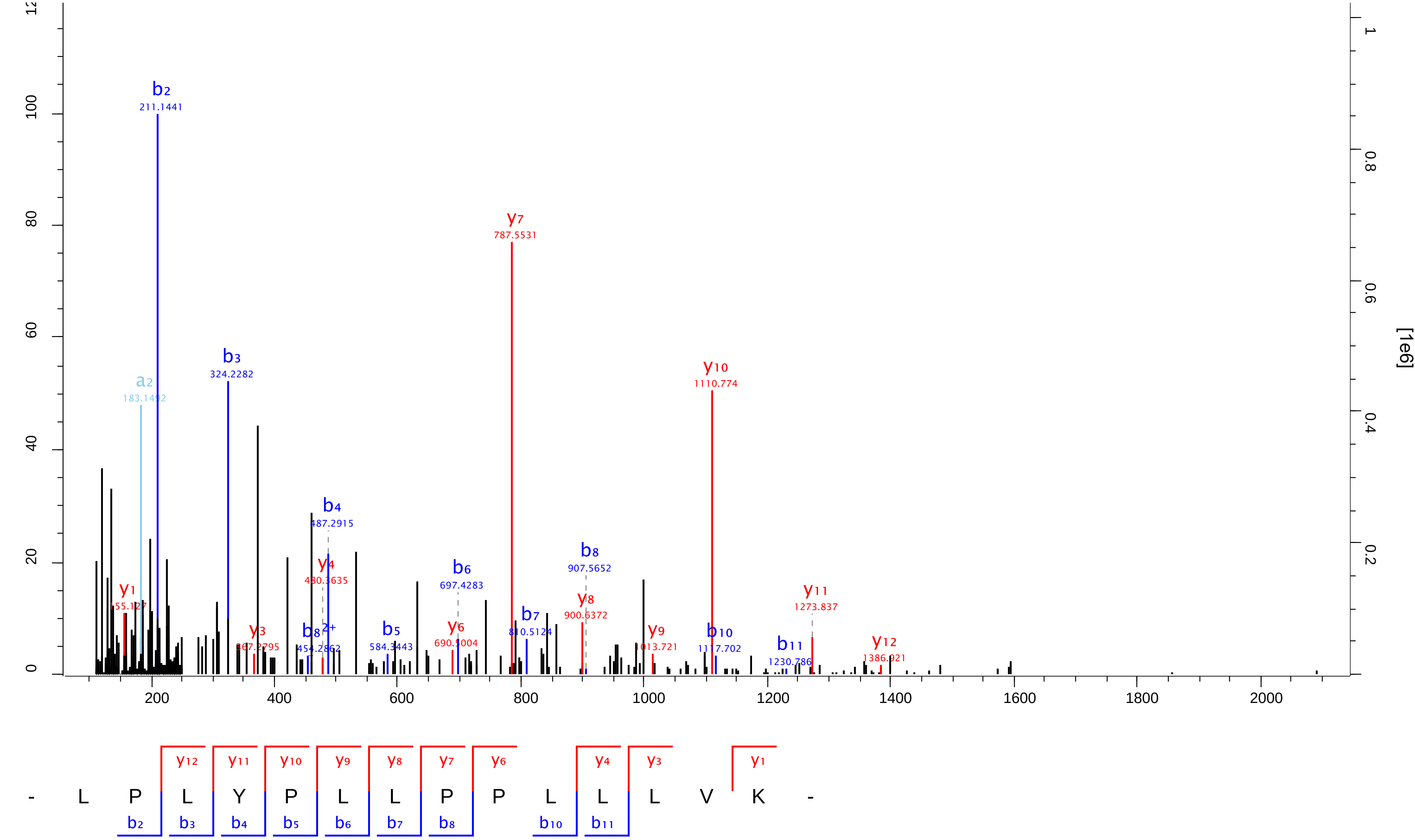

Raw file	Scan	Method	Score	m/z
QEplus003067	14032	FTMS; HCD	81.97	933.47

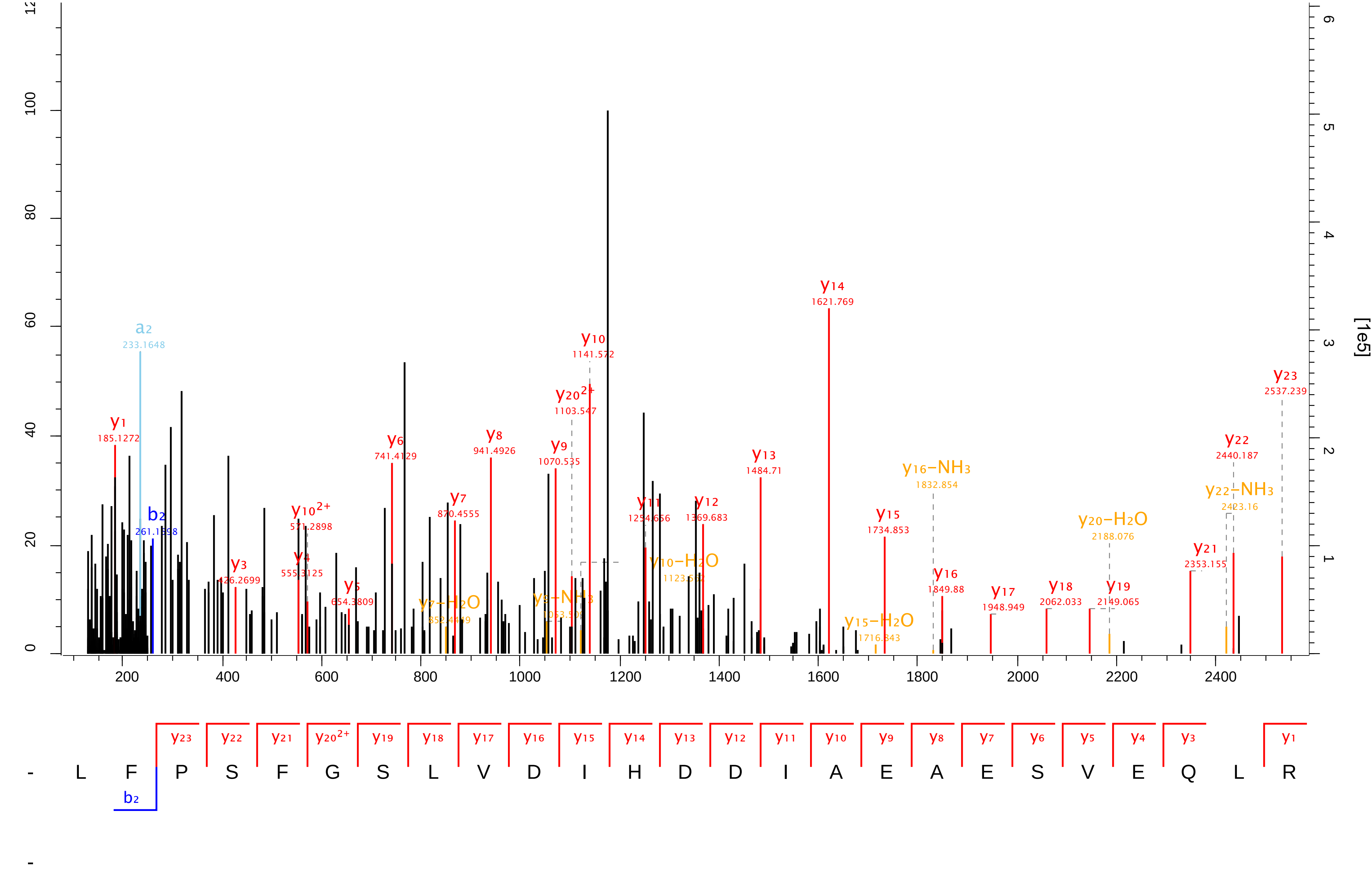

Raw file	Scan	Method	Score	m/z
QEplus003067	14447	FTMS; HCD	110.38	726.41

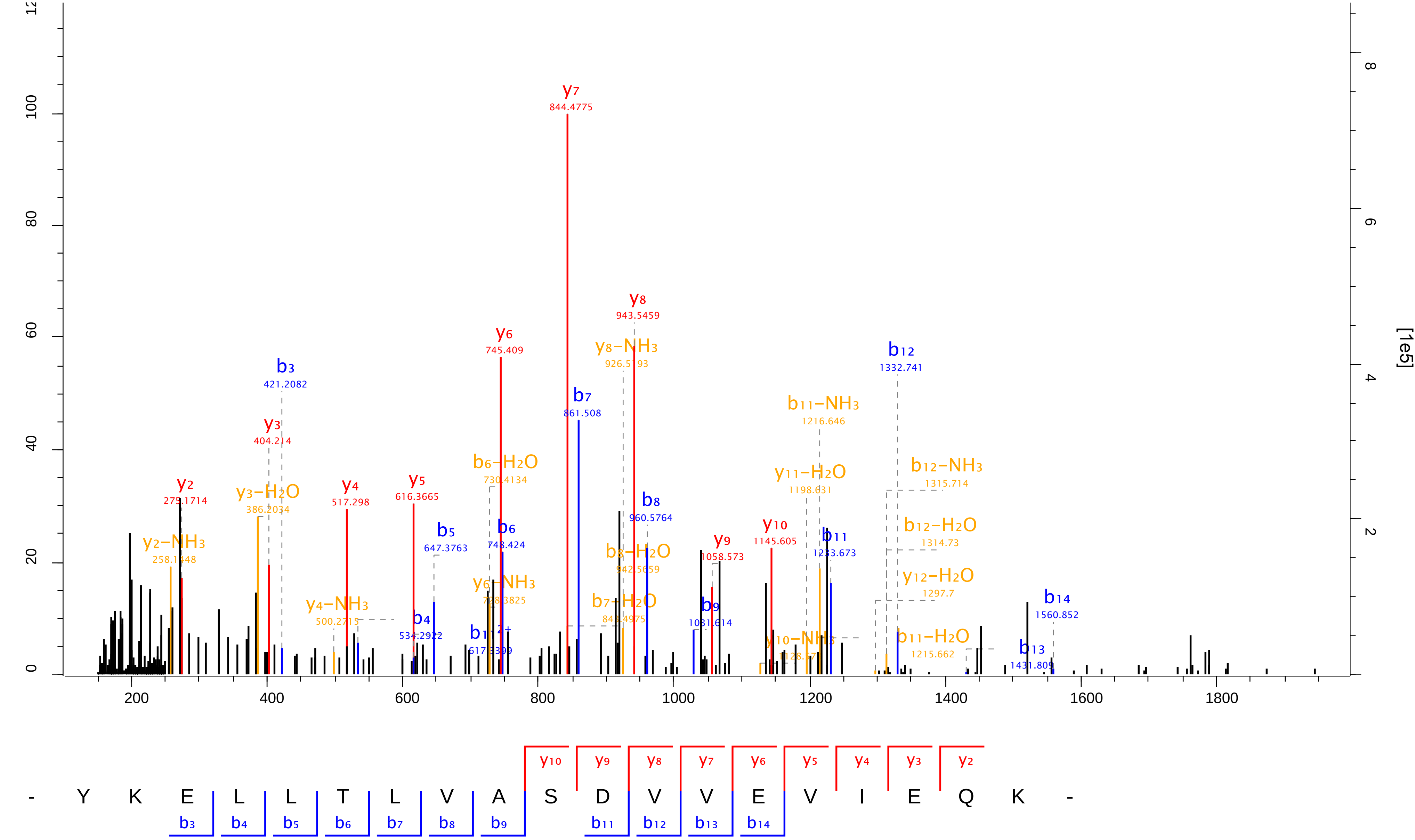

Raw file	Scan	Method	Score	m/z
QEplus003067	6994	FTMS; HCD	84.48	441.89

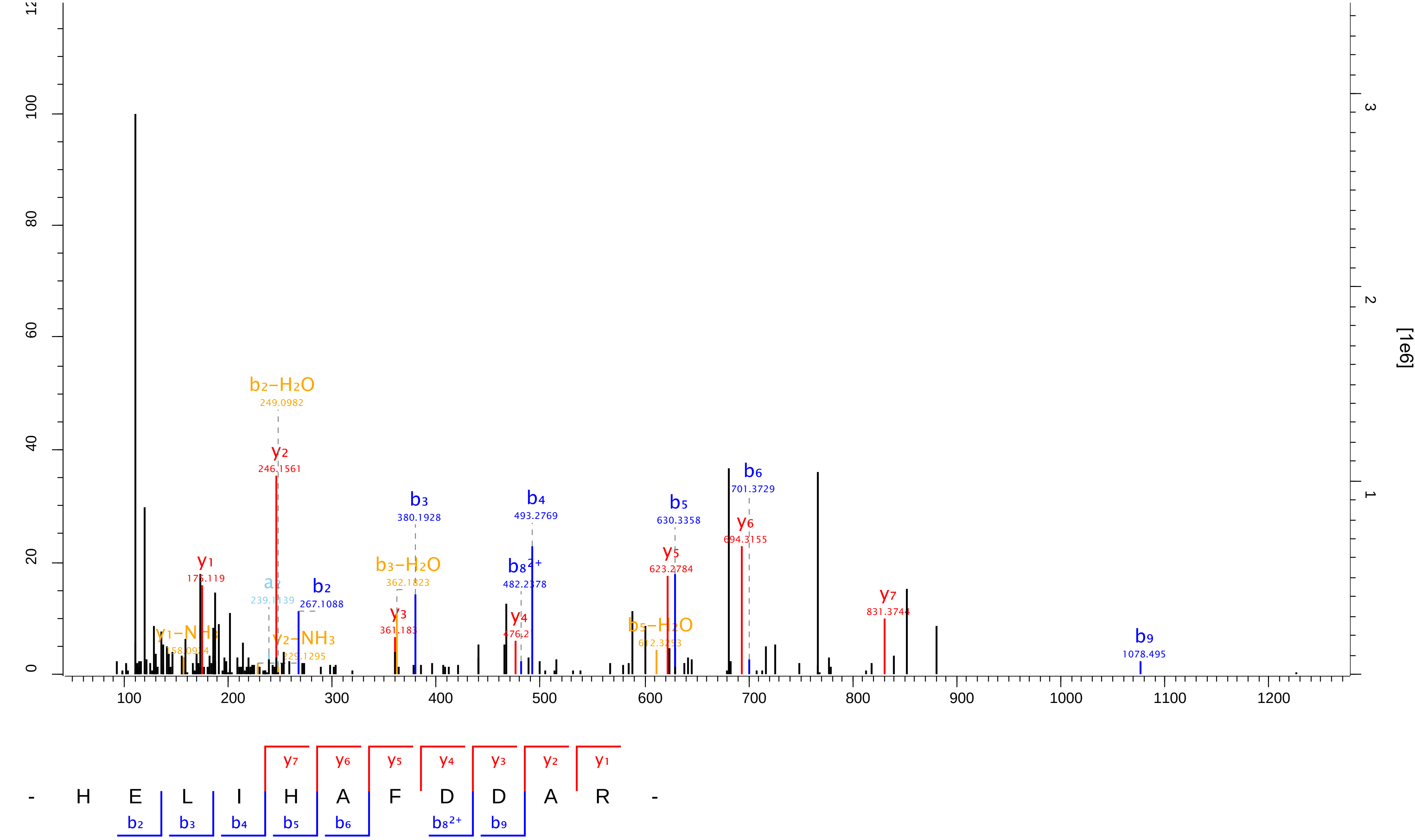

Raw file	Scan	Method	Score	m/z
QEplus003067	8178	FTMS; HCD	59.71	714.36

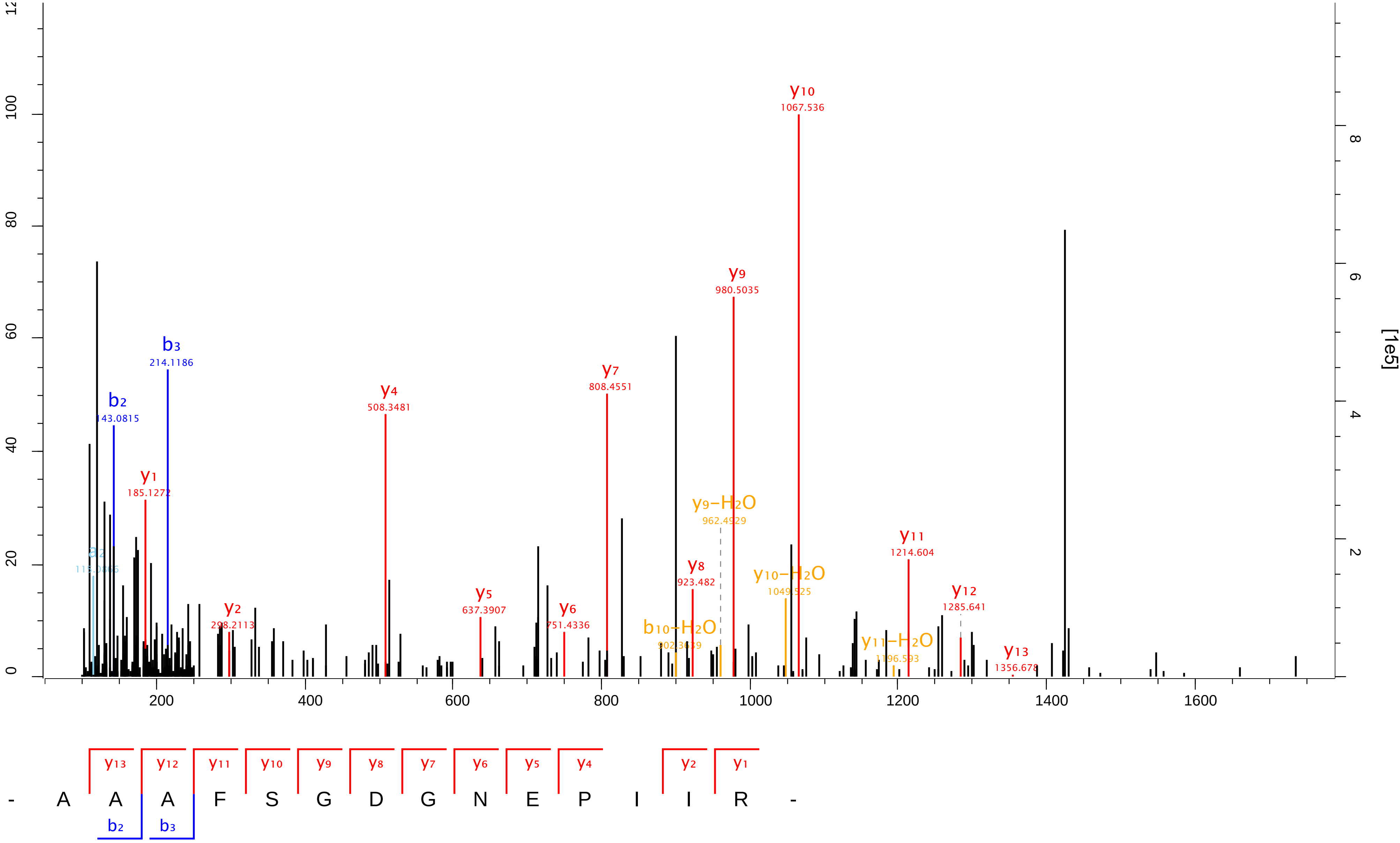

Raw file	Scan	Method	Score	m/z
QEplus003068	10724	FTMS; HCD	89.66	697.38

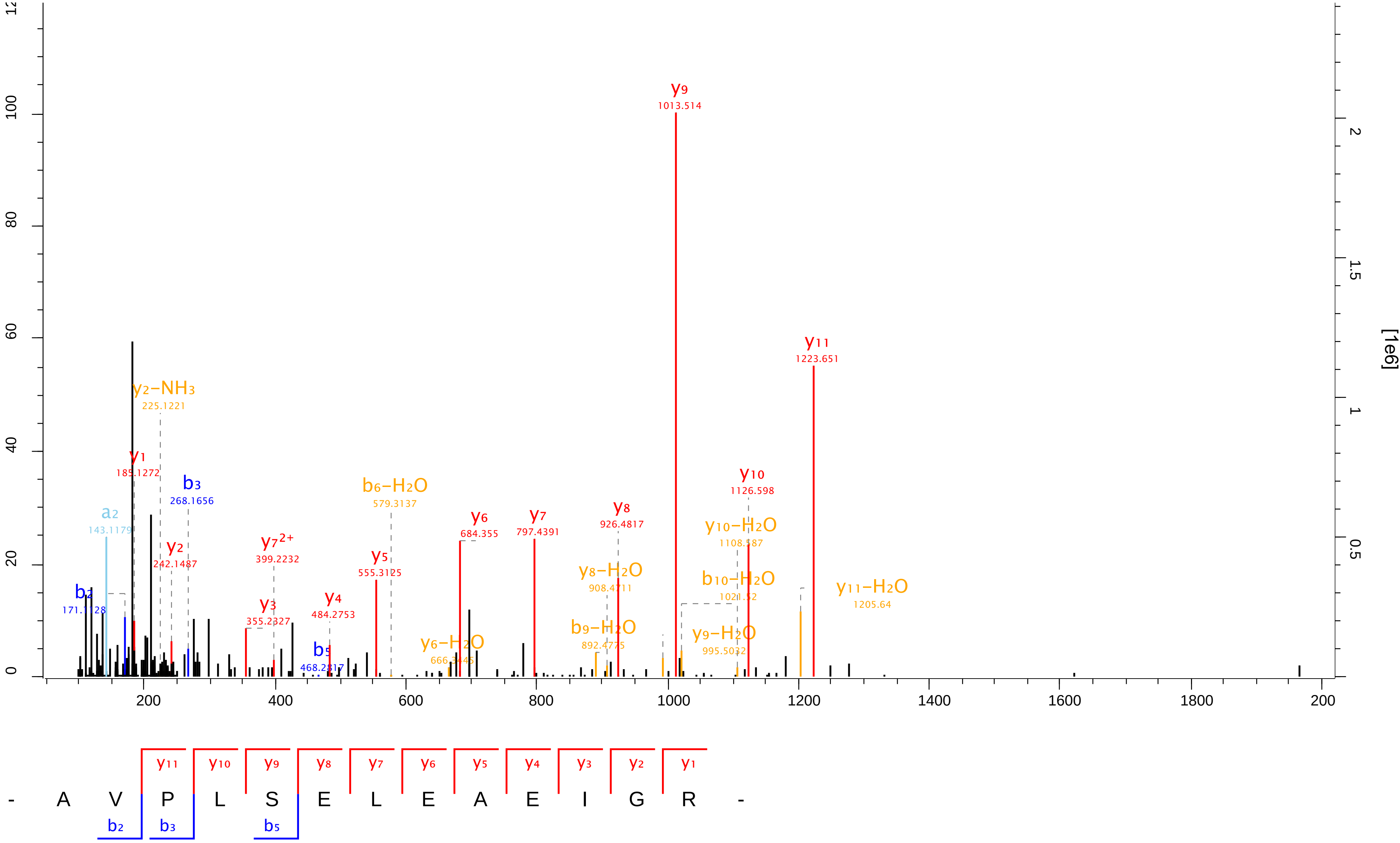

Raw file	Scan	Method	Score	m/z
QEplus003068	11434	FTMS; HCD	67.02	541.3

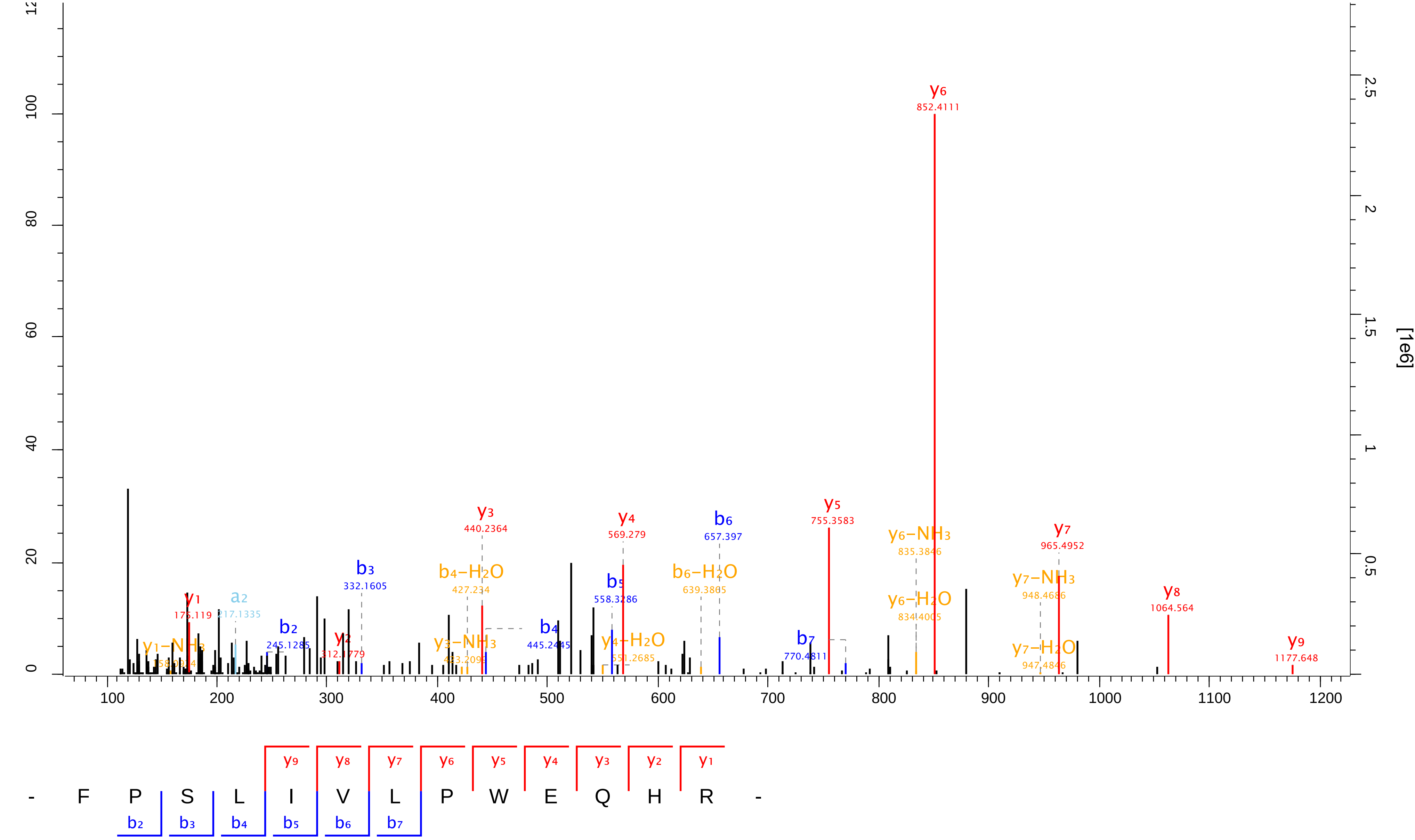

Raw file	Scan	Method	Score	m/z
QEplus003068	12033	FTMS; HCD	95.48	696.9

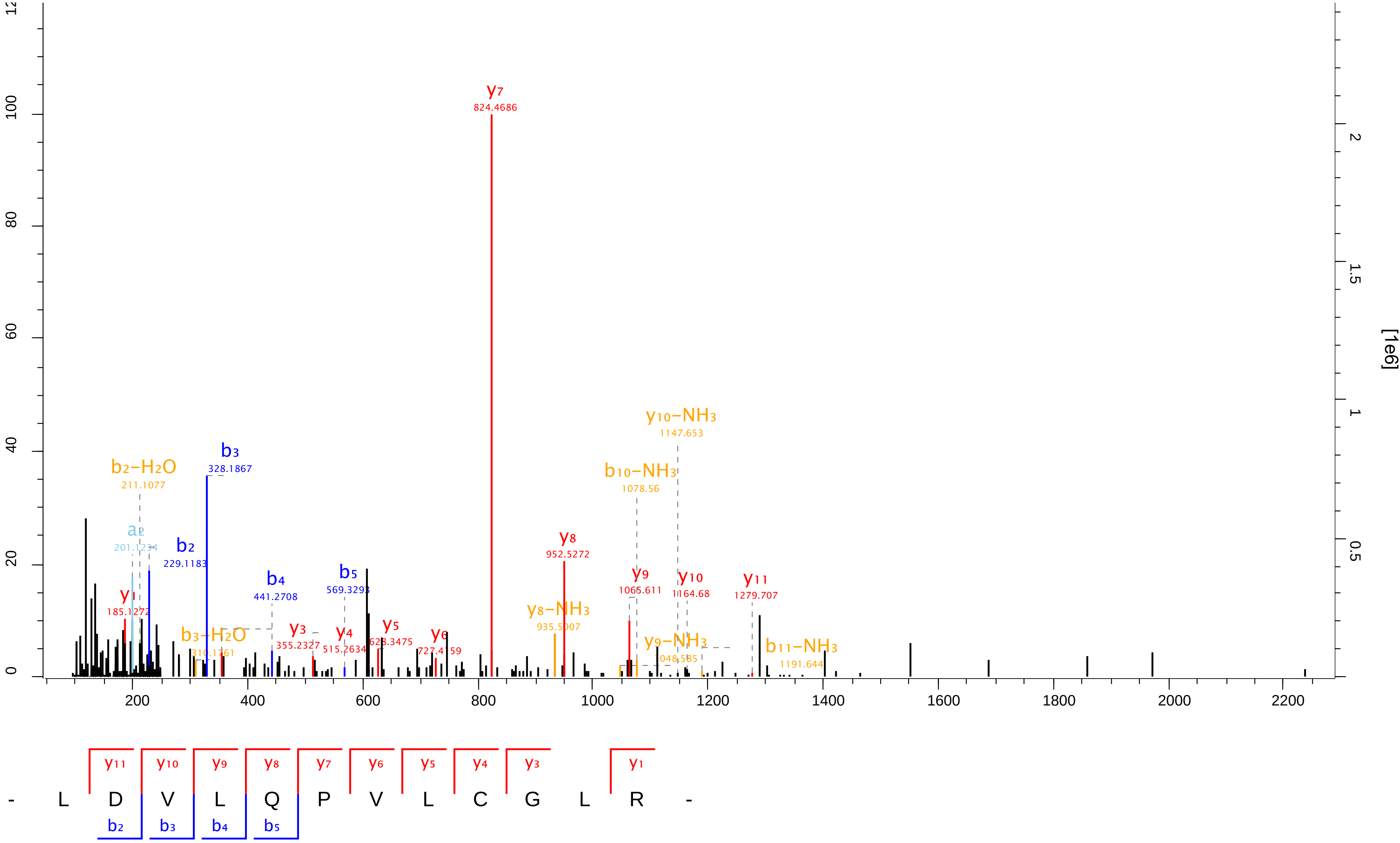

Raw file	Scan	Method	Score	m/z
QEplus003068	12589	FTMS; HCD	68.02	500.79

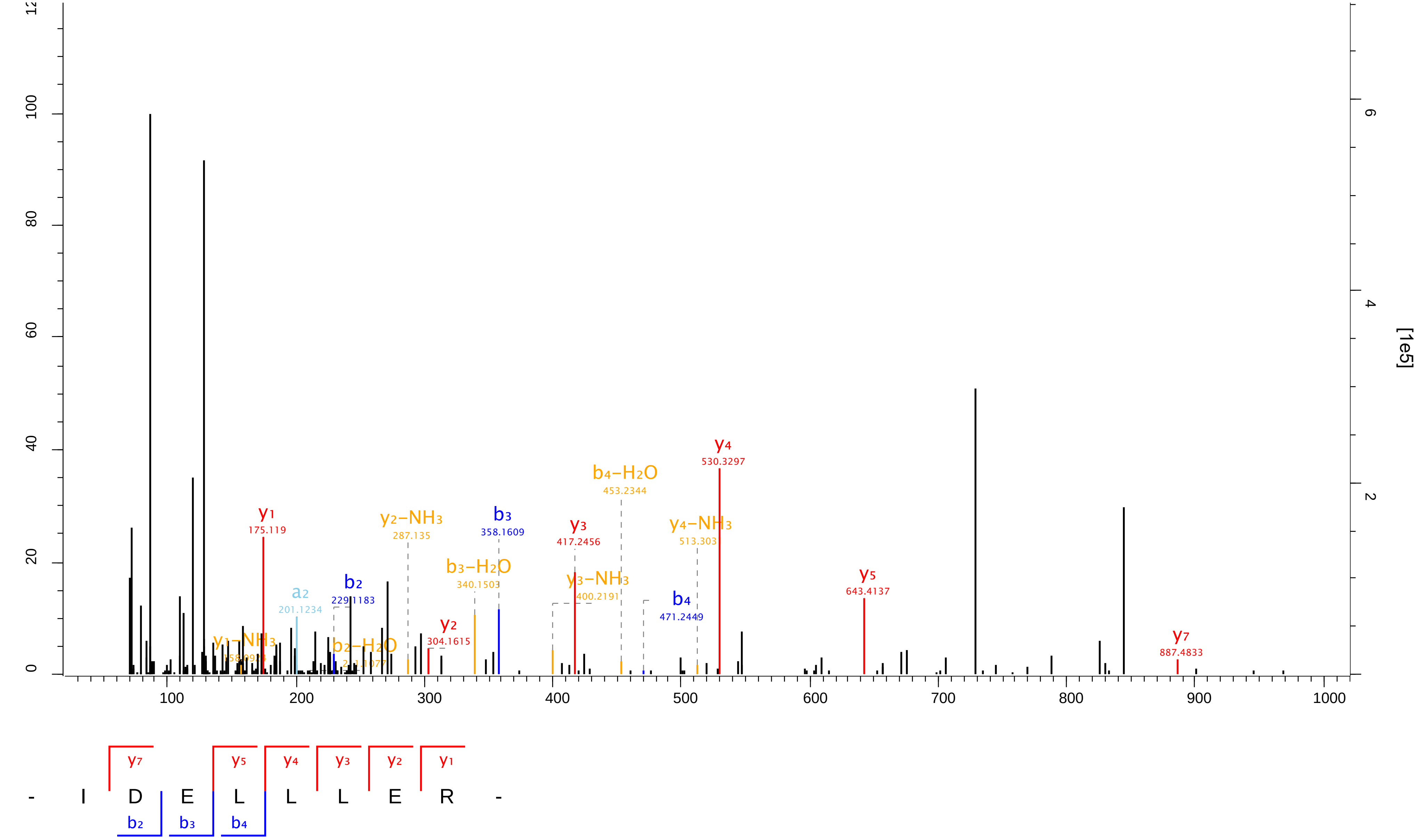

Raw file	Scan	Method	Score	m/z
QEplus003068	13055	FTMS; HCD	84.48	684.89

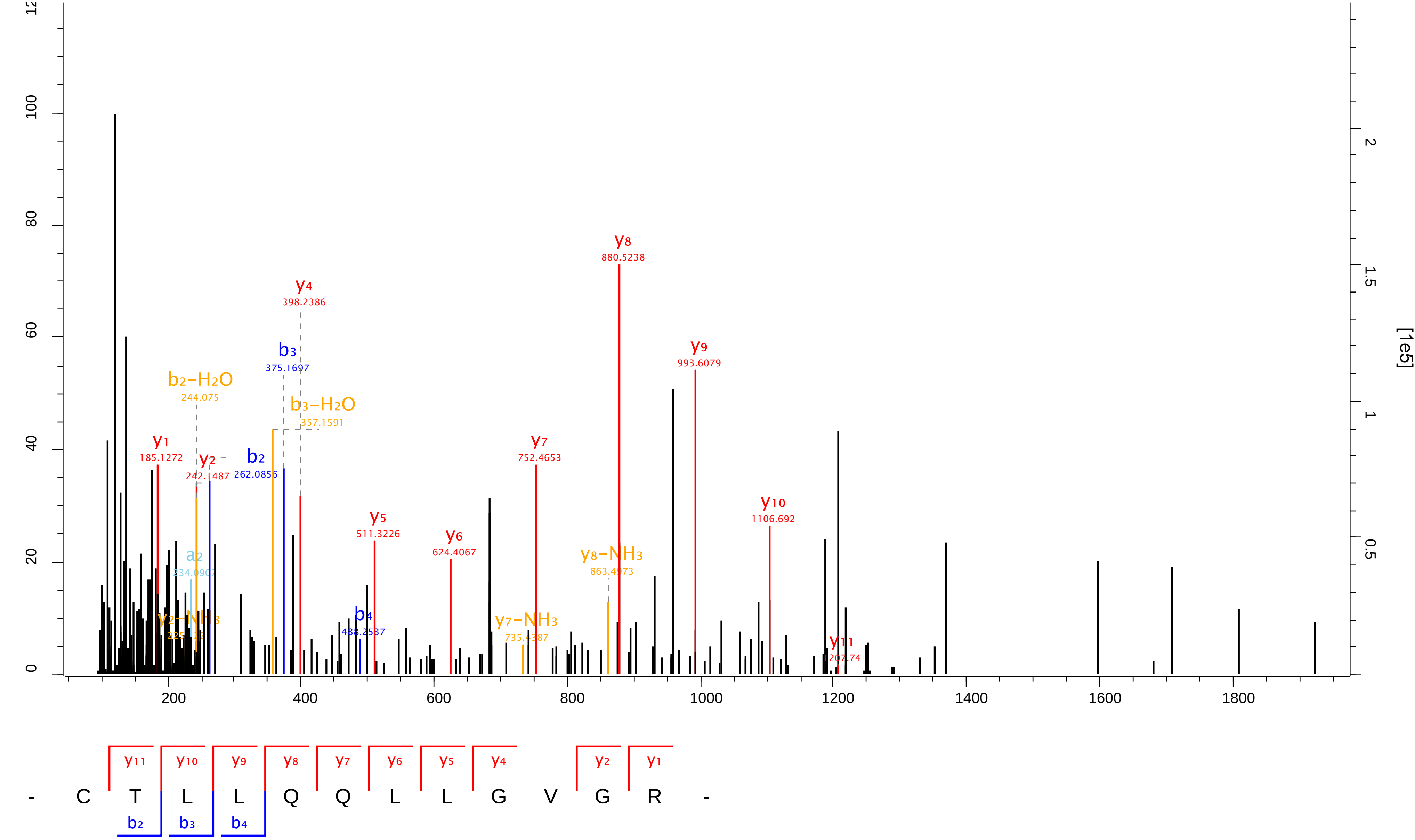

Raw file	Scan	Method	Score	m/z
QEplus003068	13682	FTMS; HCD	77.03	932.48

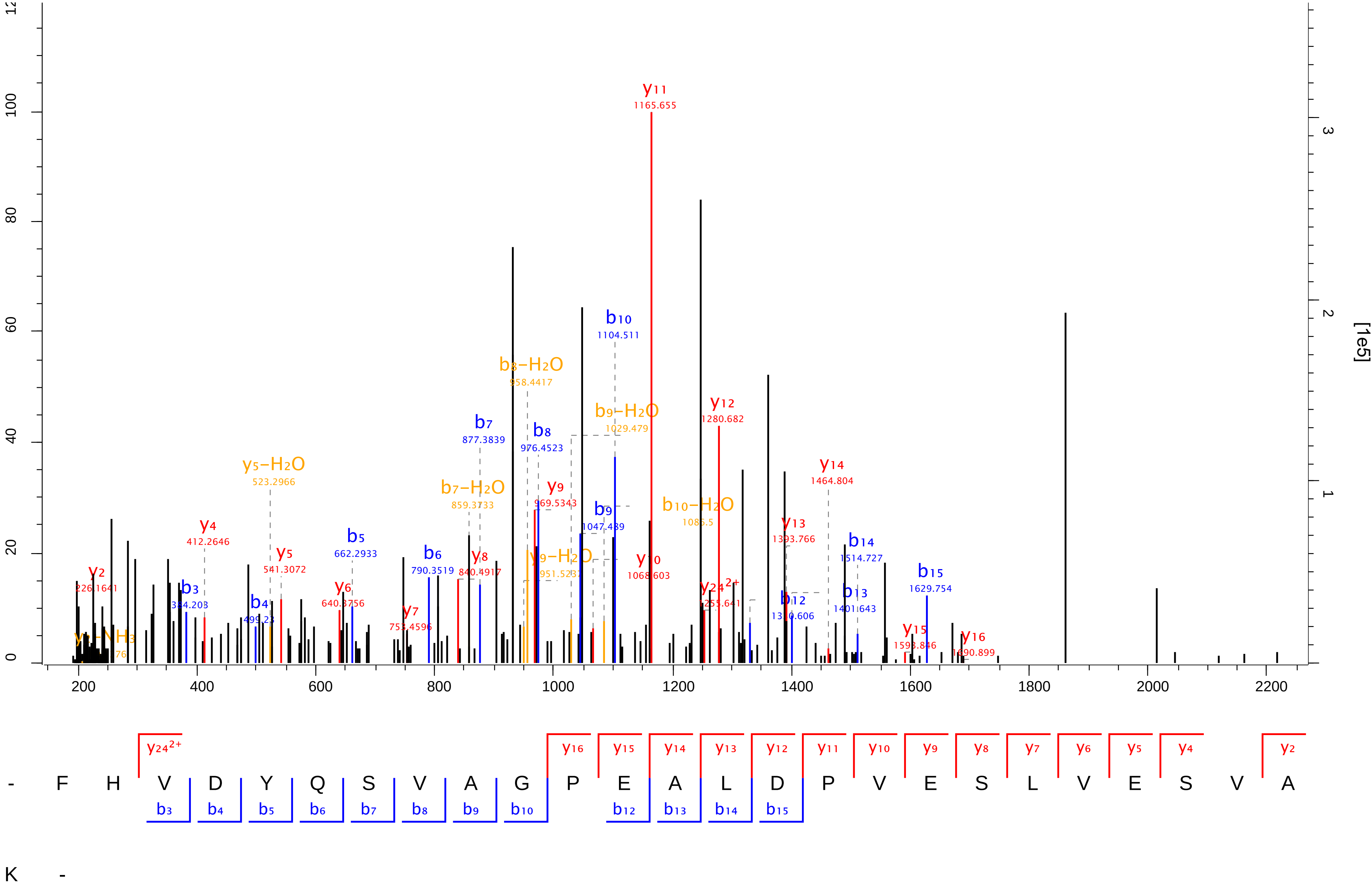

Raw file	Scan	Method	Score	m/z
QEplus003068	14053	FTMS; HCD	102.08	956.98

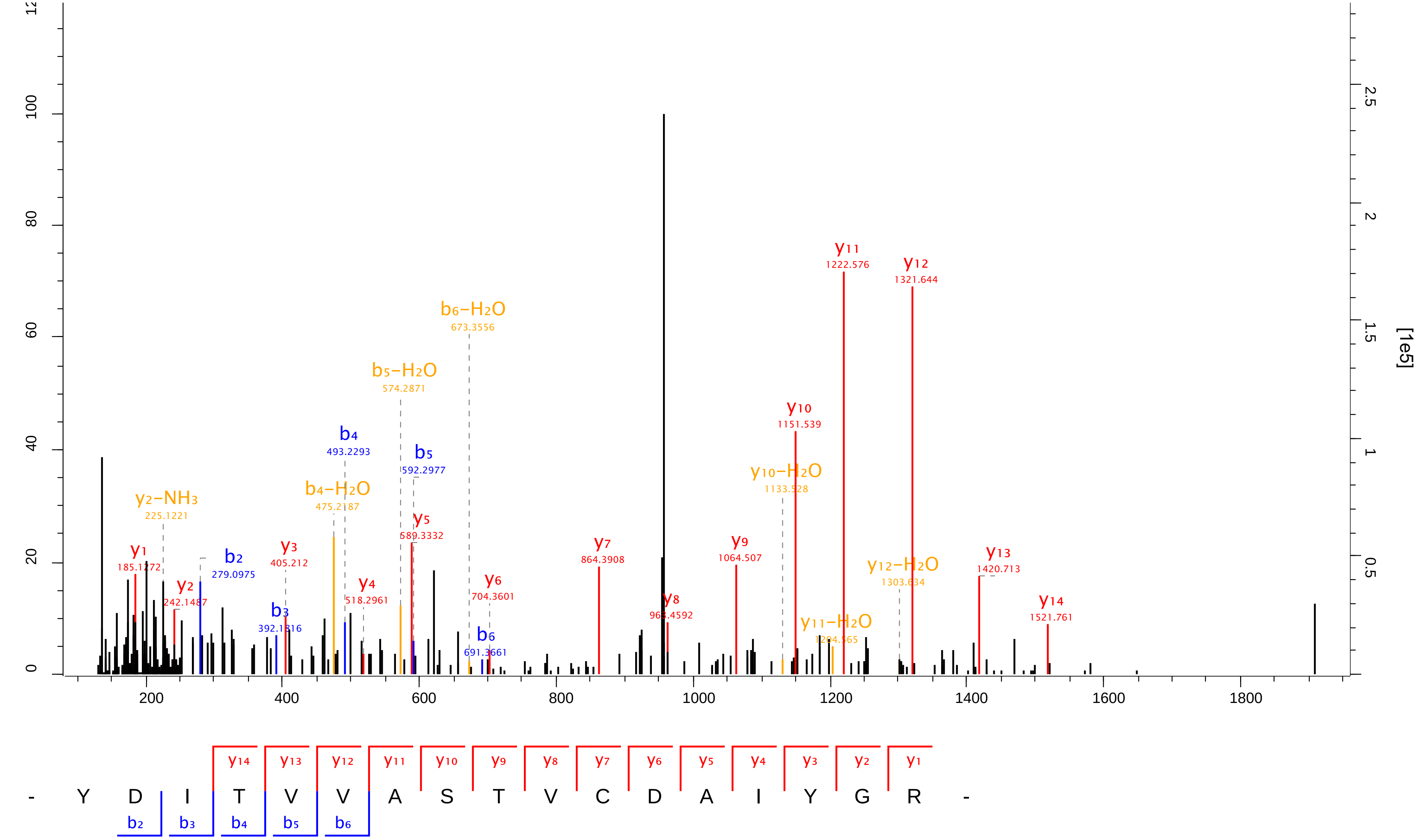

Raw file	Scan	Method	Score	m/z
QEplus003068	4123	FTMS; HCD	132.01	510.29

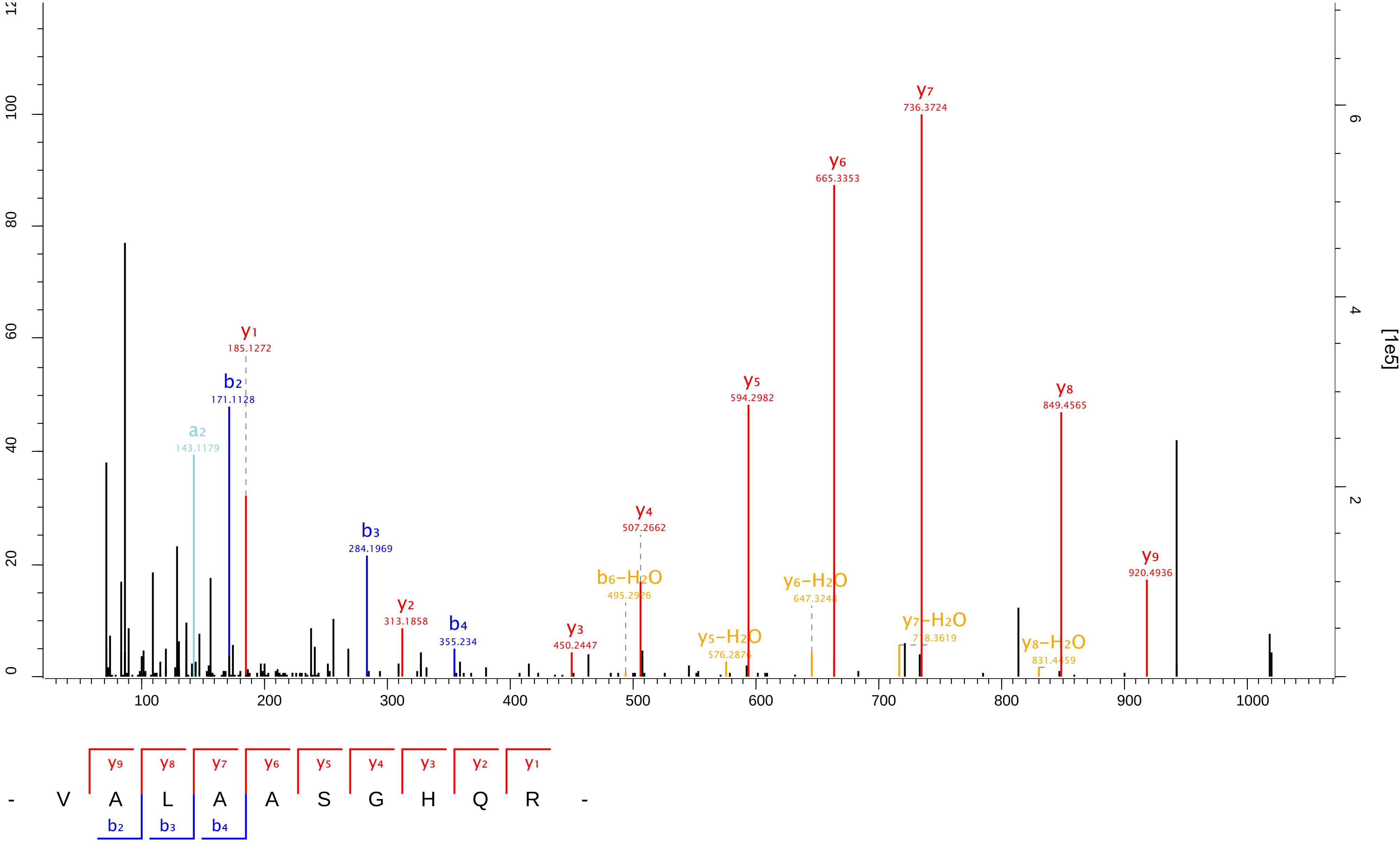

Raw file	Scan	Method	Score	m/z
QEplus003068	4209	FTMS; HCD	88.6	584.32

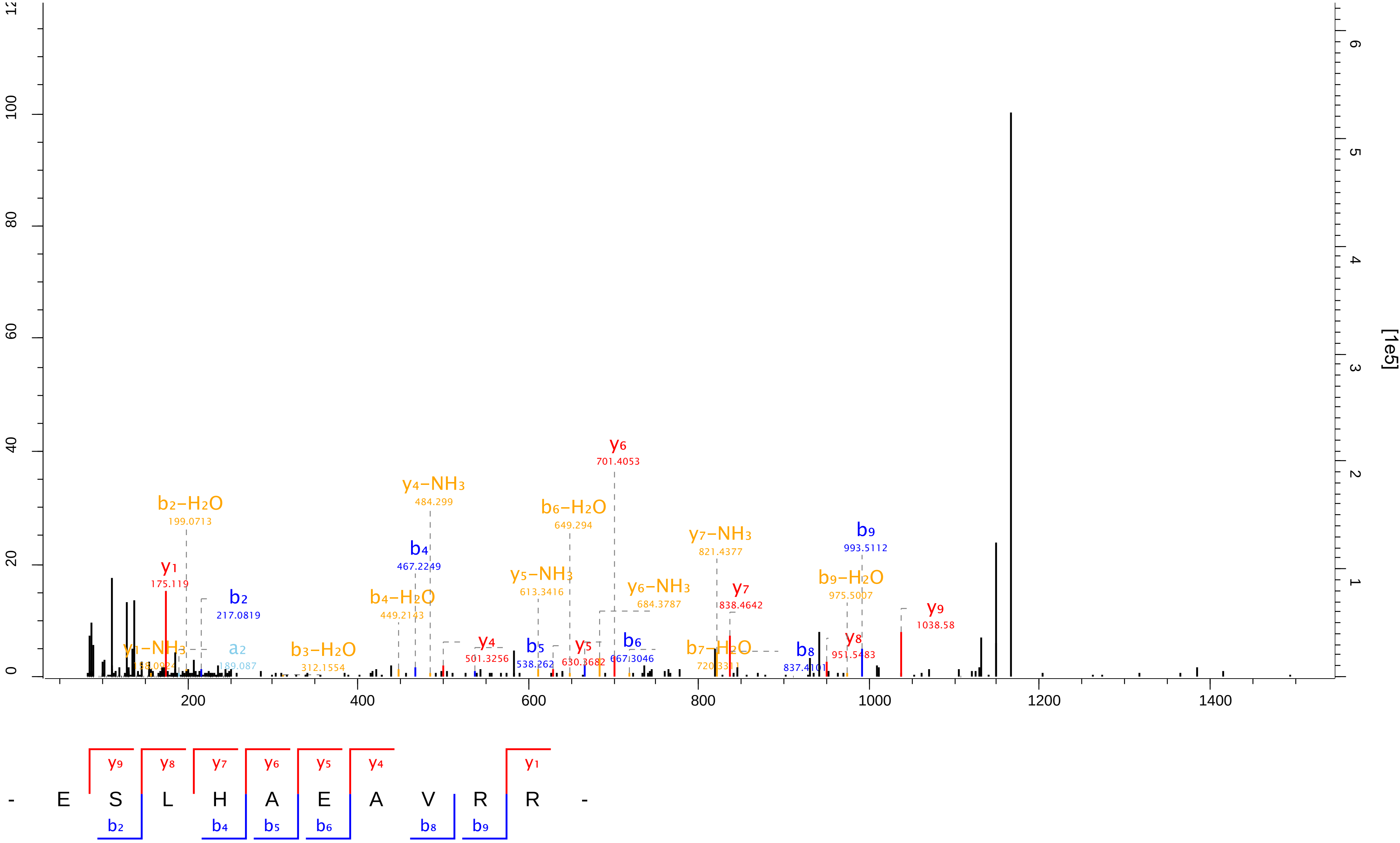

Raw file	Scan	Method	Score	m/z
QEplus003068	6420	FTMS; HCD	56.14	689.85

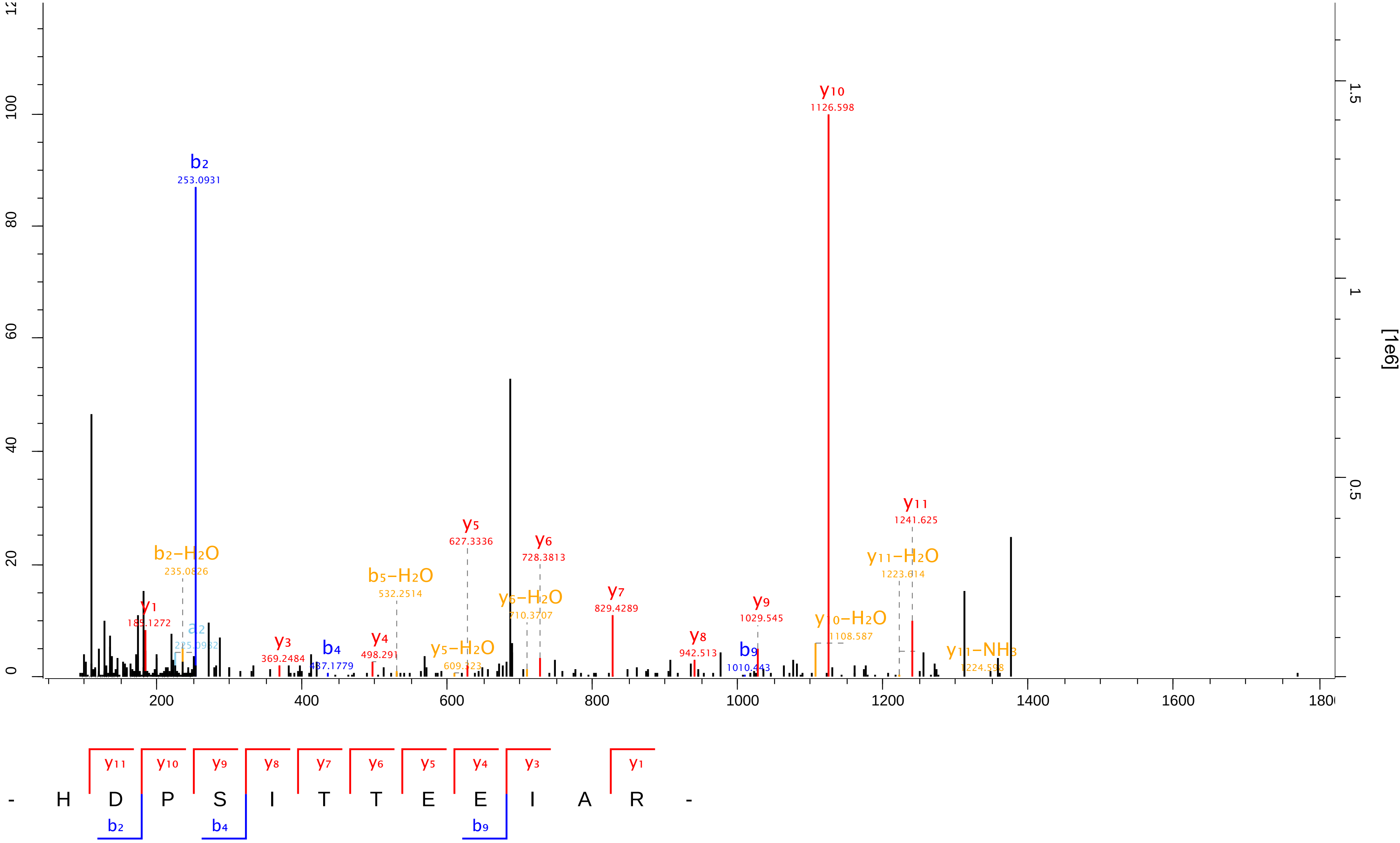

Raw file	Scan	Method	Score	m/z
QEplus003068	9426	FTMS; HCD	71.34	501.78

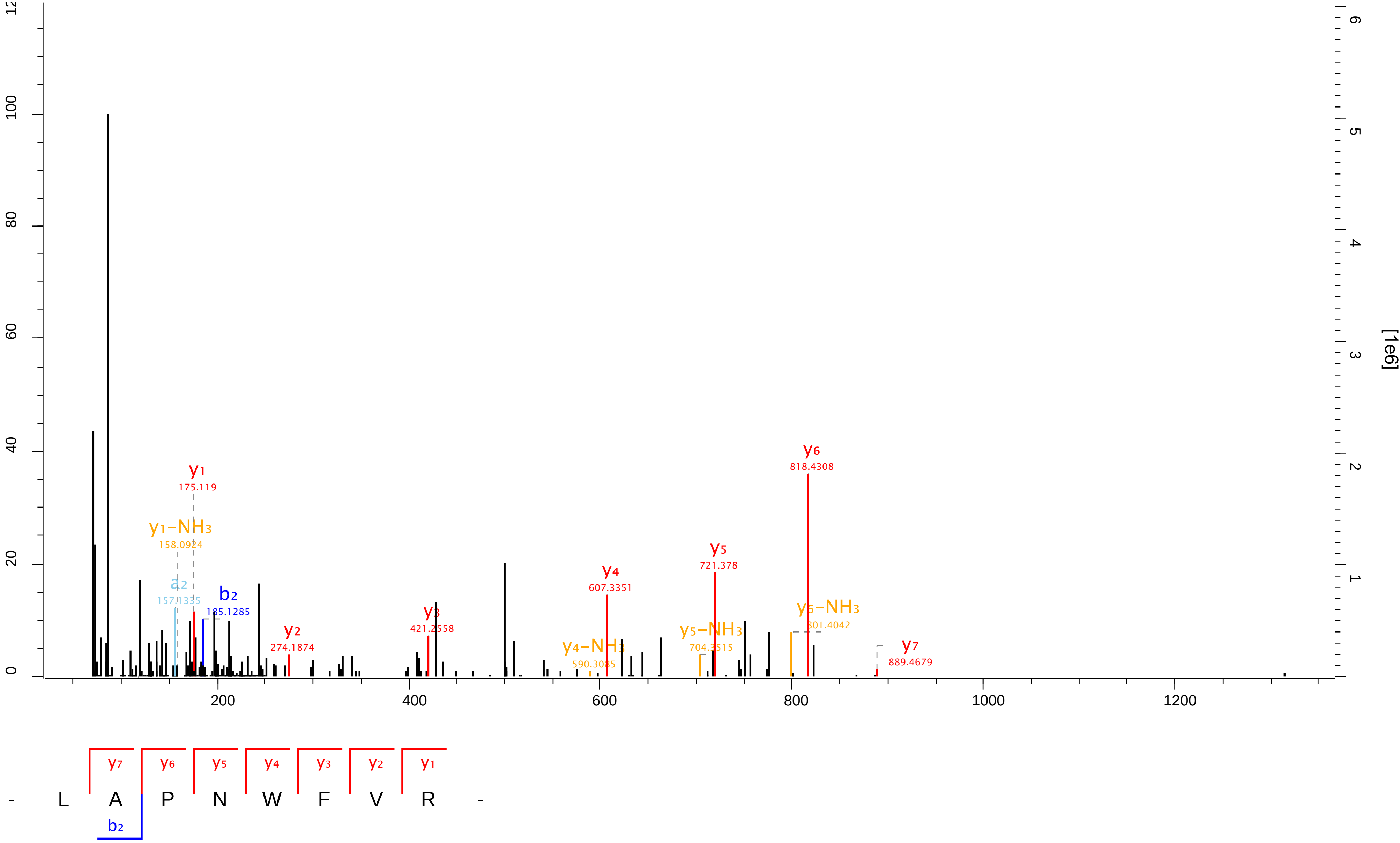

Raw file	Scan	Method	Score	m/z
QEplus003069	12617	FTMS; HCD	82.07	769.95

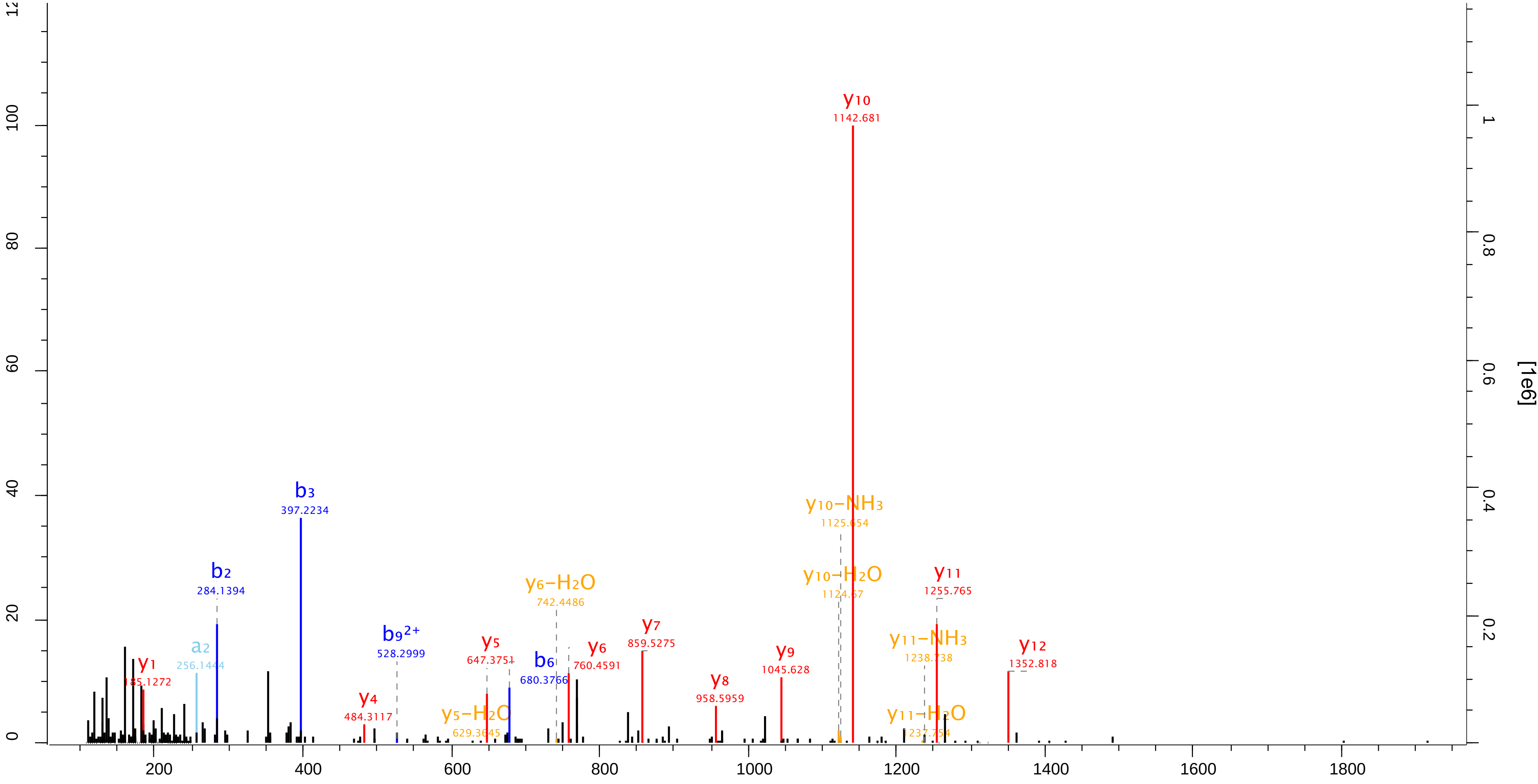

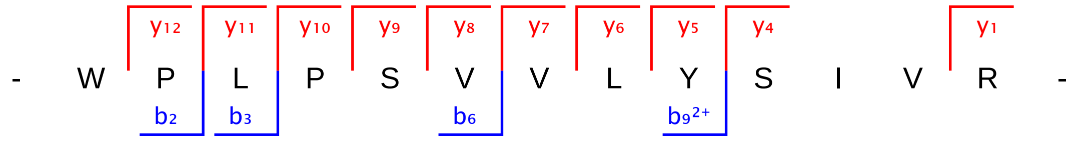

Raw file	Scan	Method	Score	m/z
QEplus003069	12717	FTMS; HCD	197.14	956.03

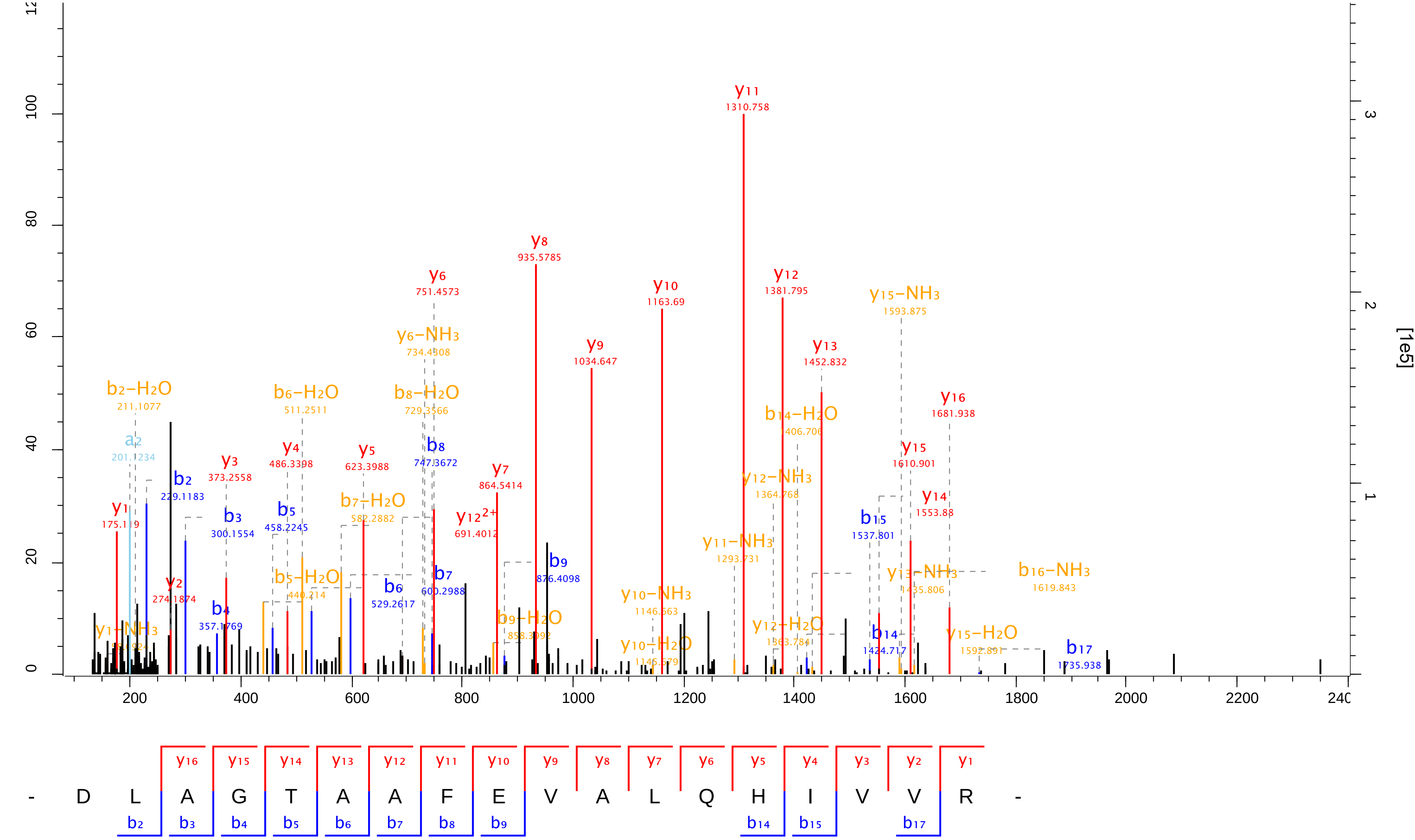

Raw file	Scan	Method	Score	m/z
QEplus003069	13225	FTMS; HCD	146.19	813.41

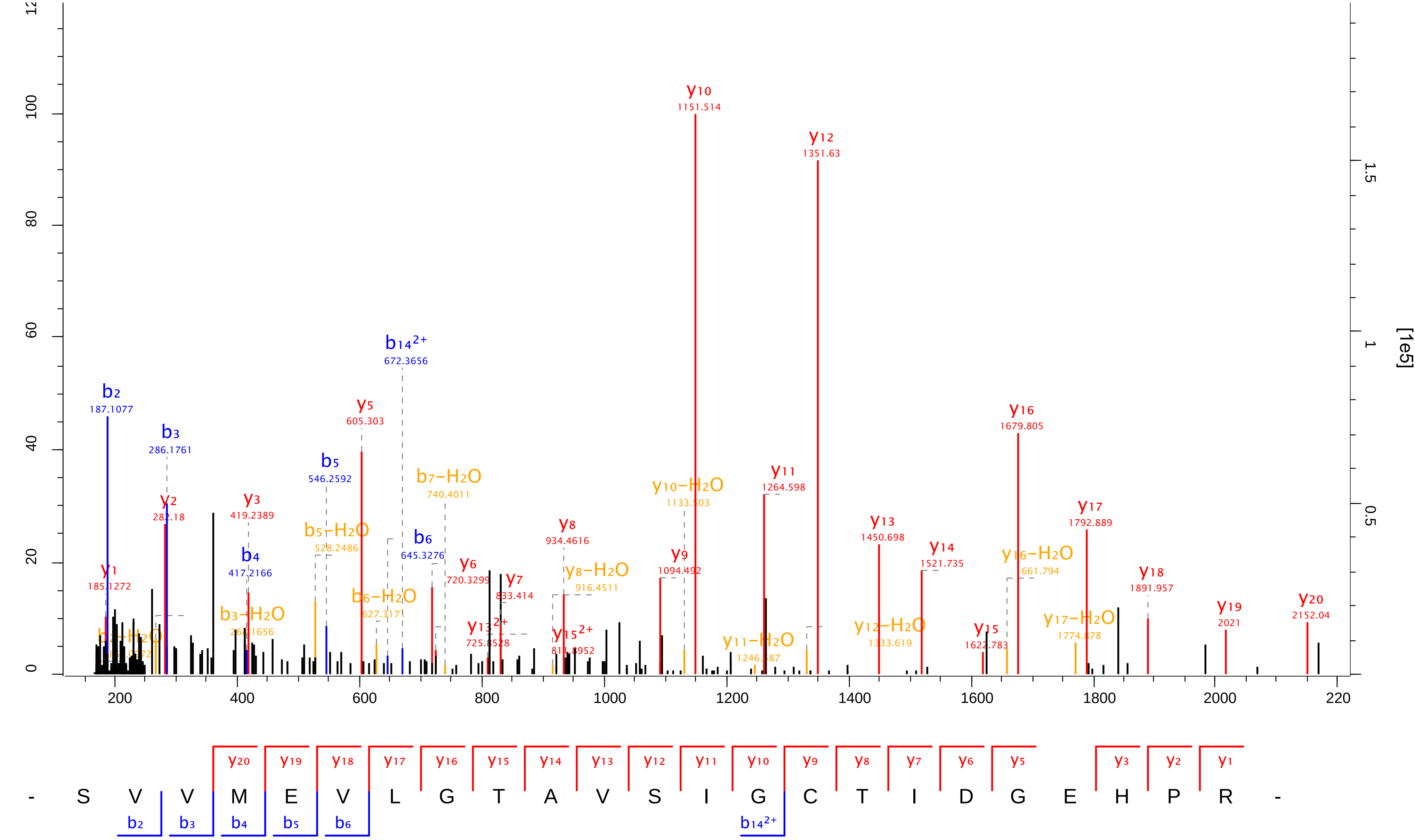

Raw file	Scan	Method	Score	m/z
QEplus003069	13785	FTMS; HCD	241.76	1315.7

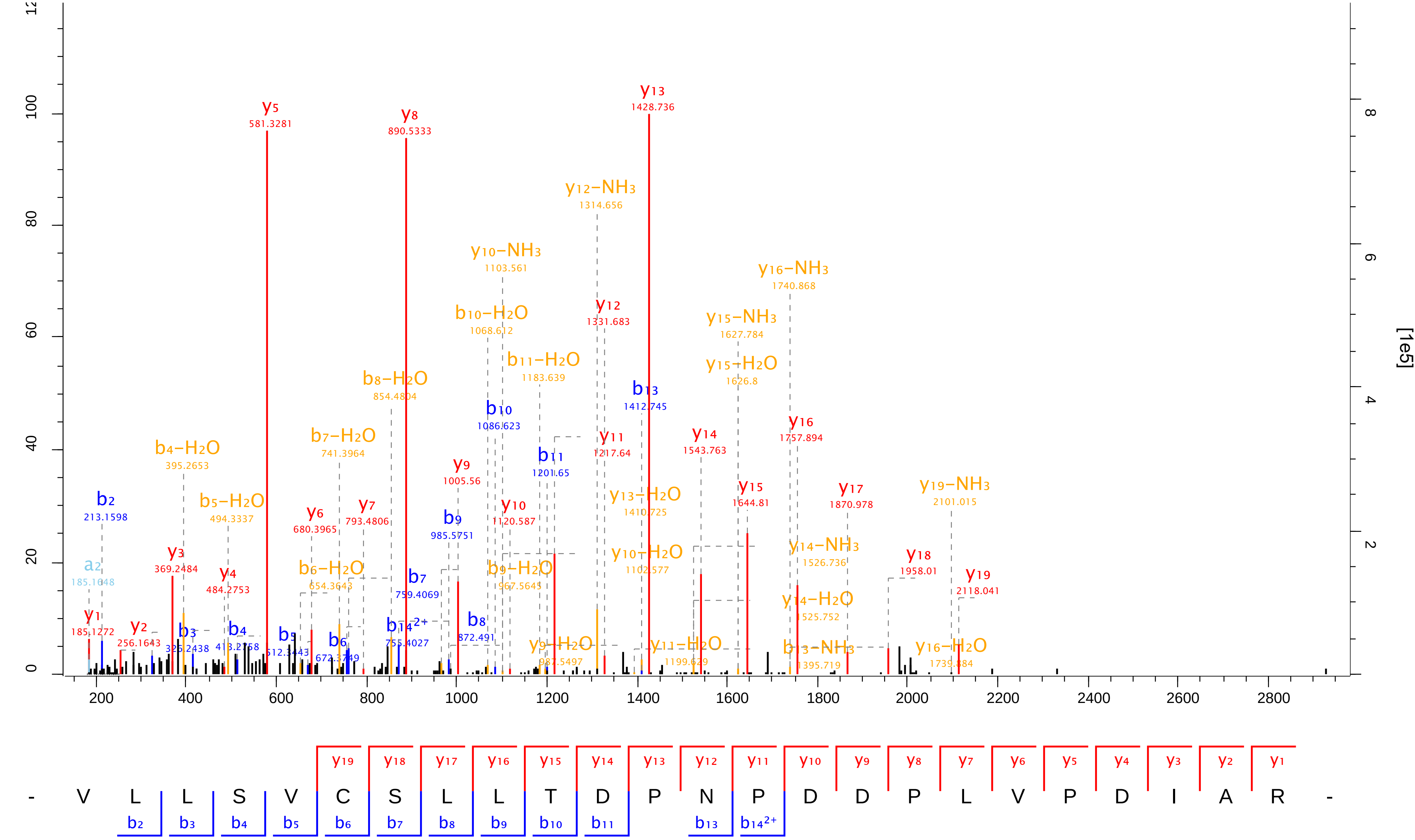

Raw file	Scan	Method	Score	m/z
QEplus003069	14563	FTMS; HCD	80.91	805.38

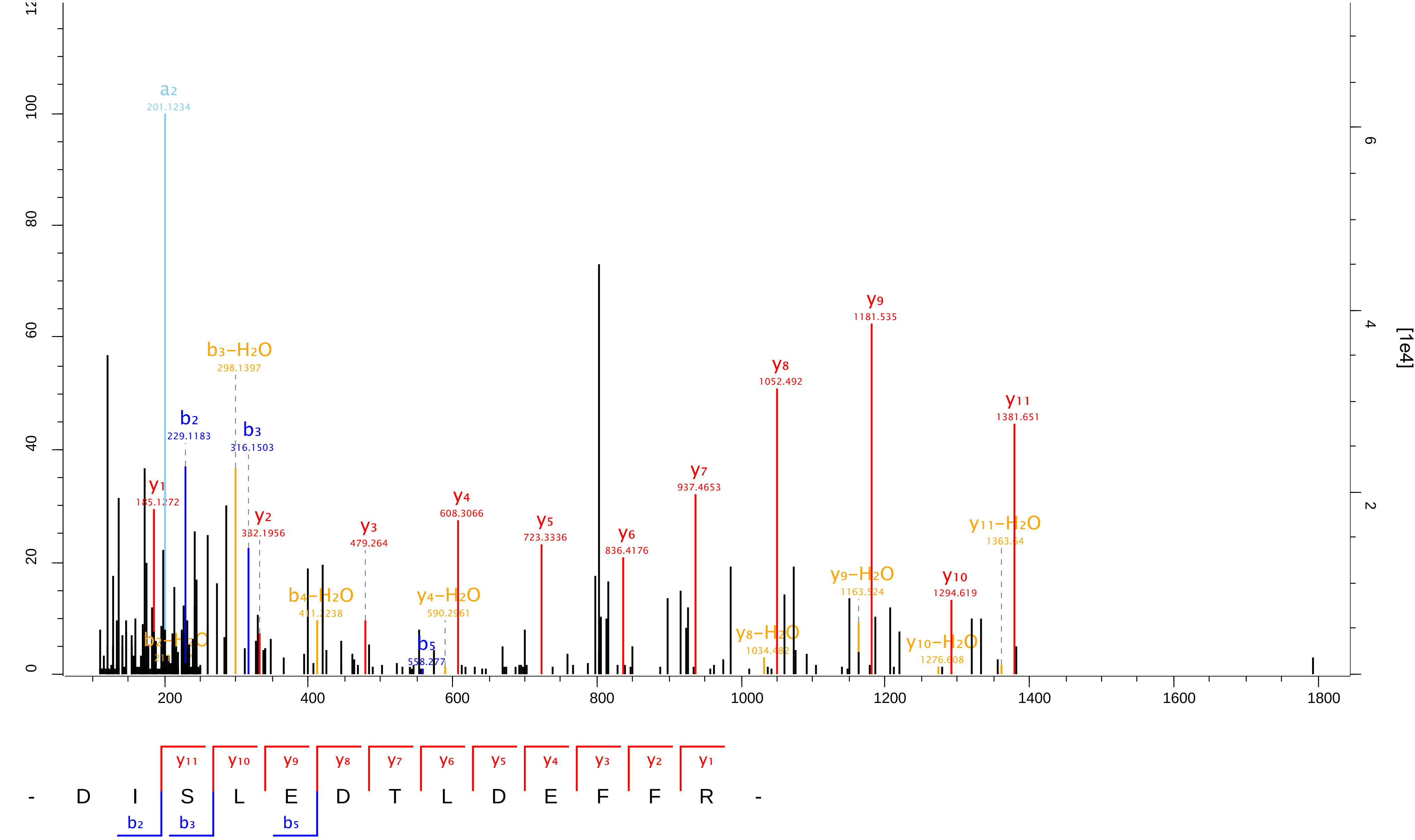

Raw file	Scan	Method	Score	m/z
QEplus003069	14717	FTMS; HCD	118.21	873.01

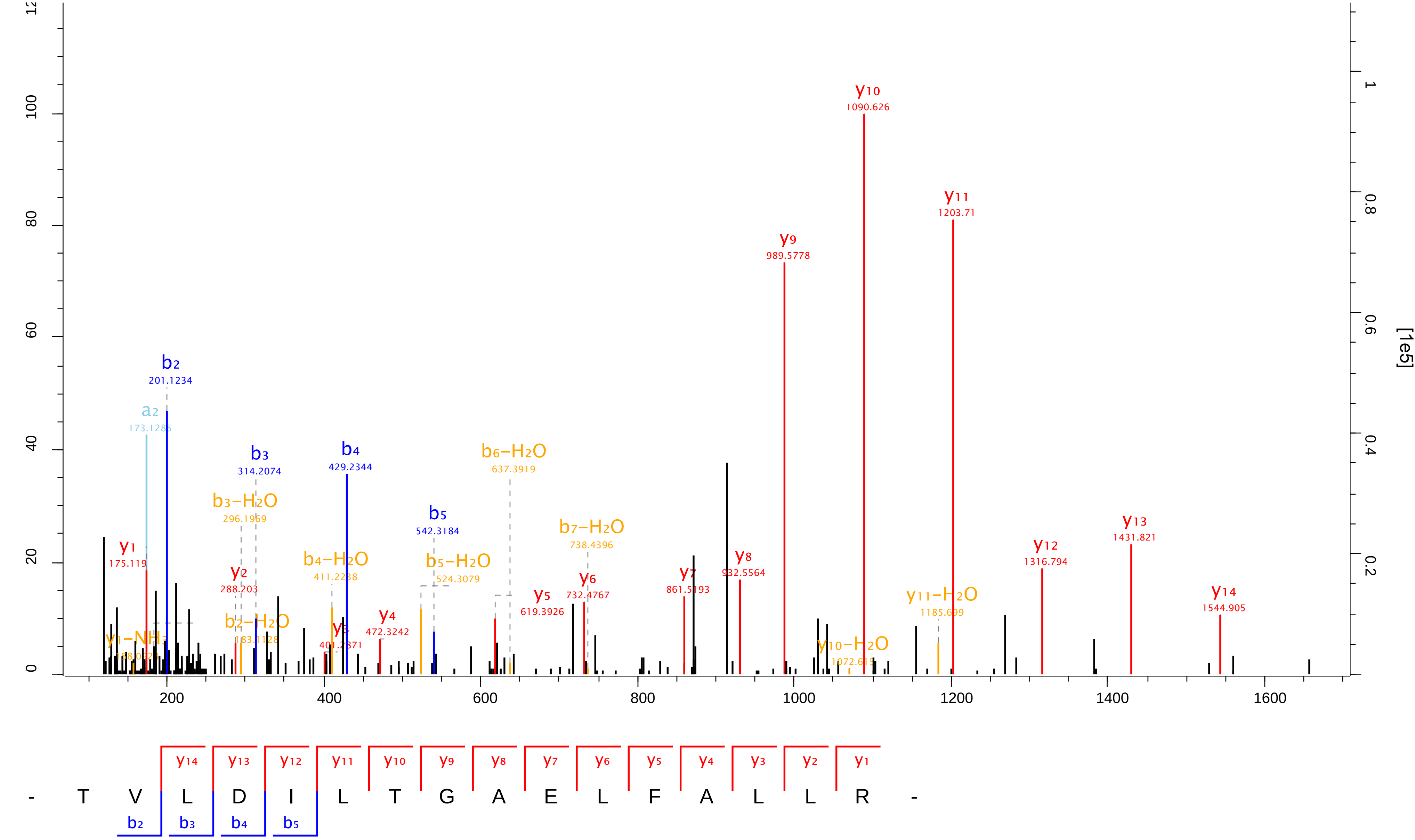

Raw file	Scan	Method	Score	m/z
QEplus003070	10599	FTMS; HCD	78.81	723.38

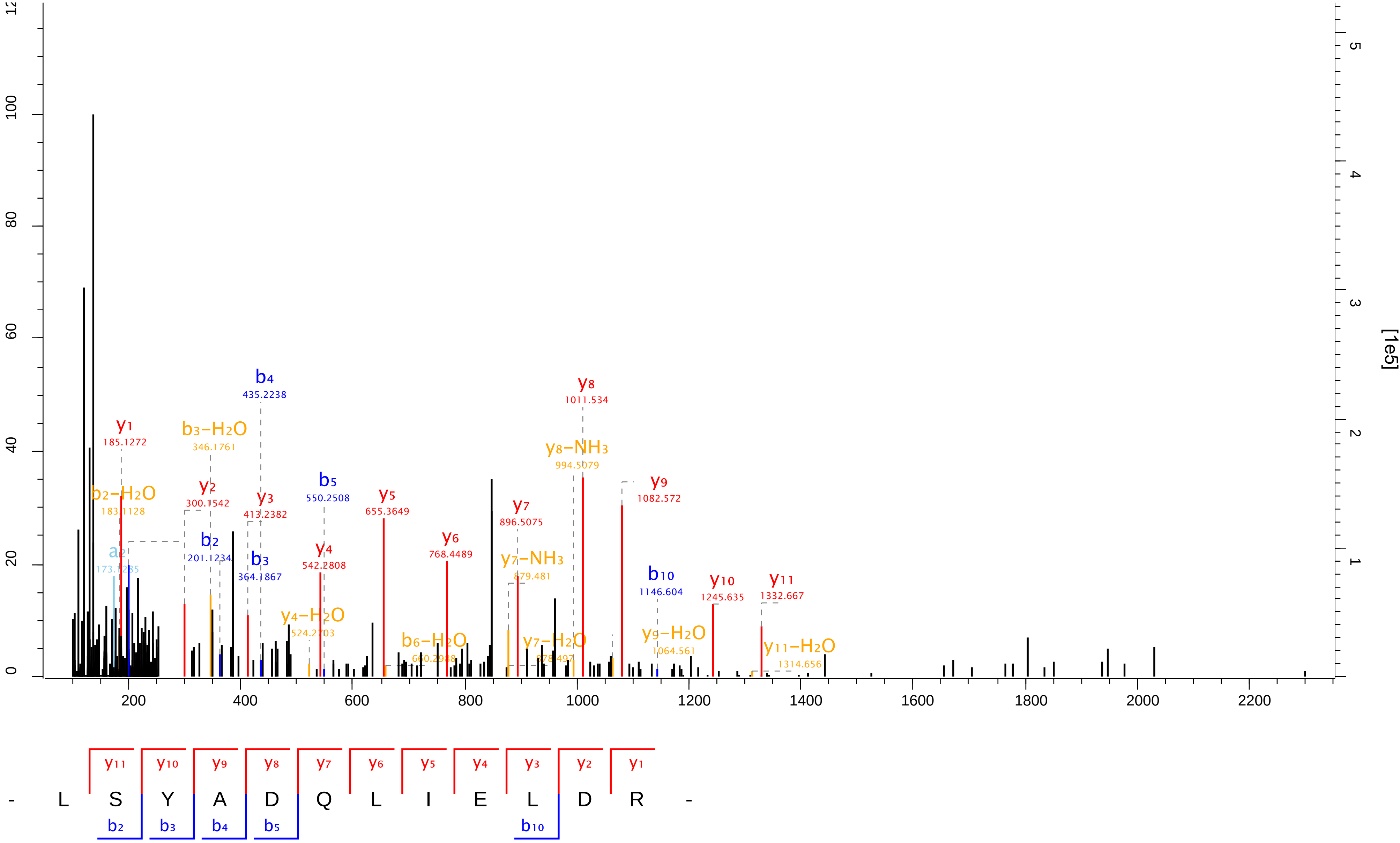

Raw file	Scan	Method	Score	m/z
QEplus003070	10699	FTMS; HCD	51.03	769.92

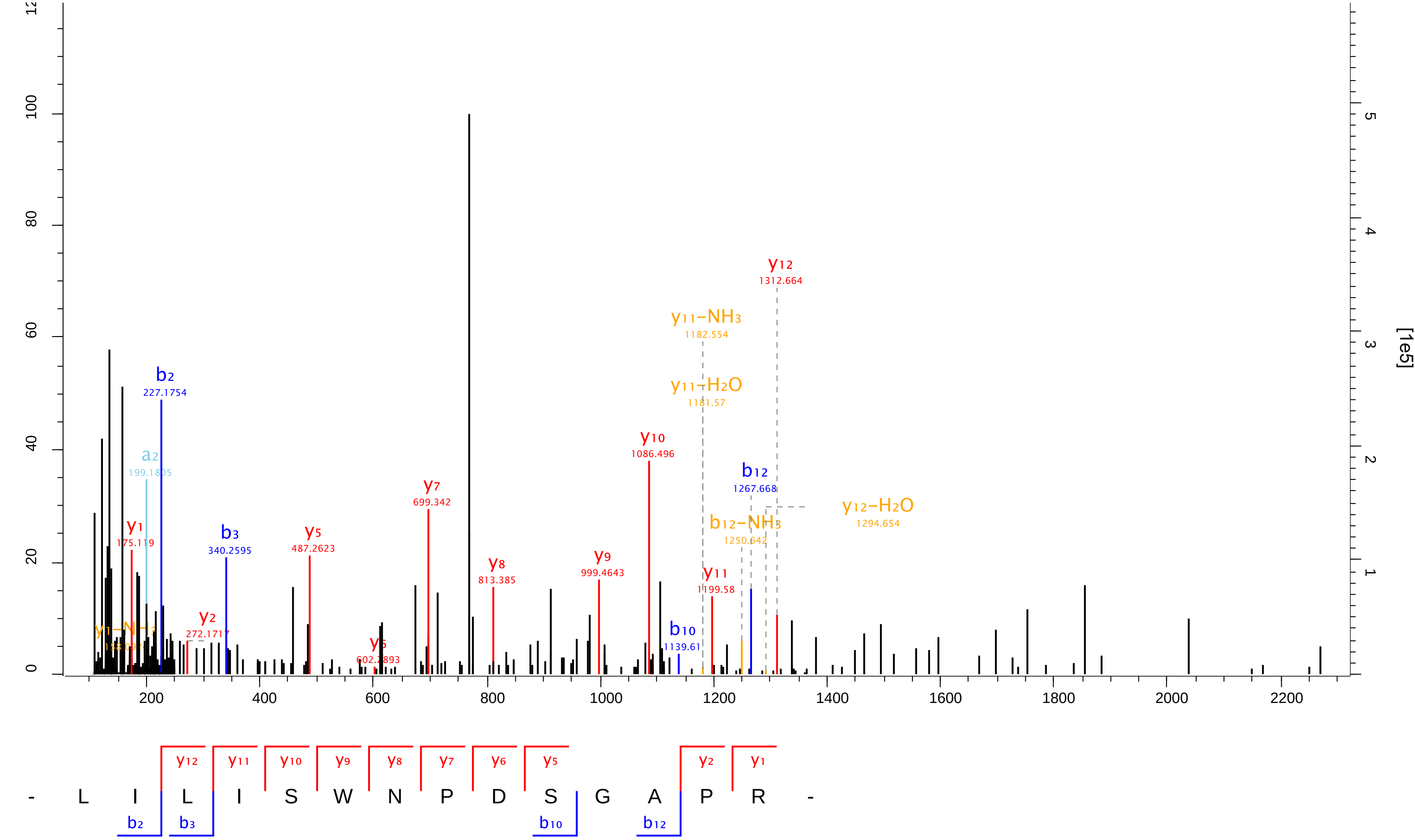

Raw file	Scan	Method	Score	m/z
QEplus003070	10999	FTMS; HCD	125.24	573.33

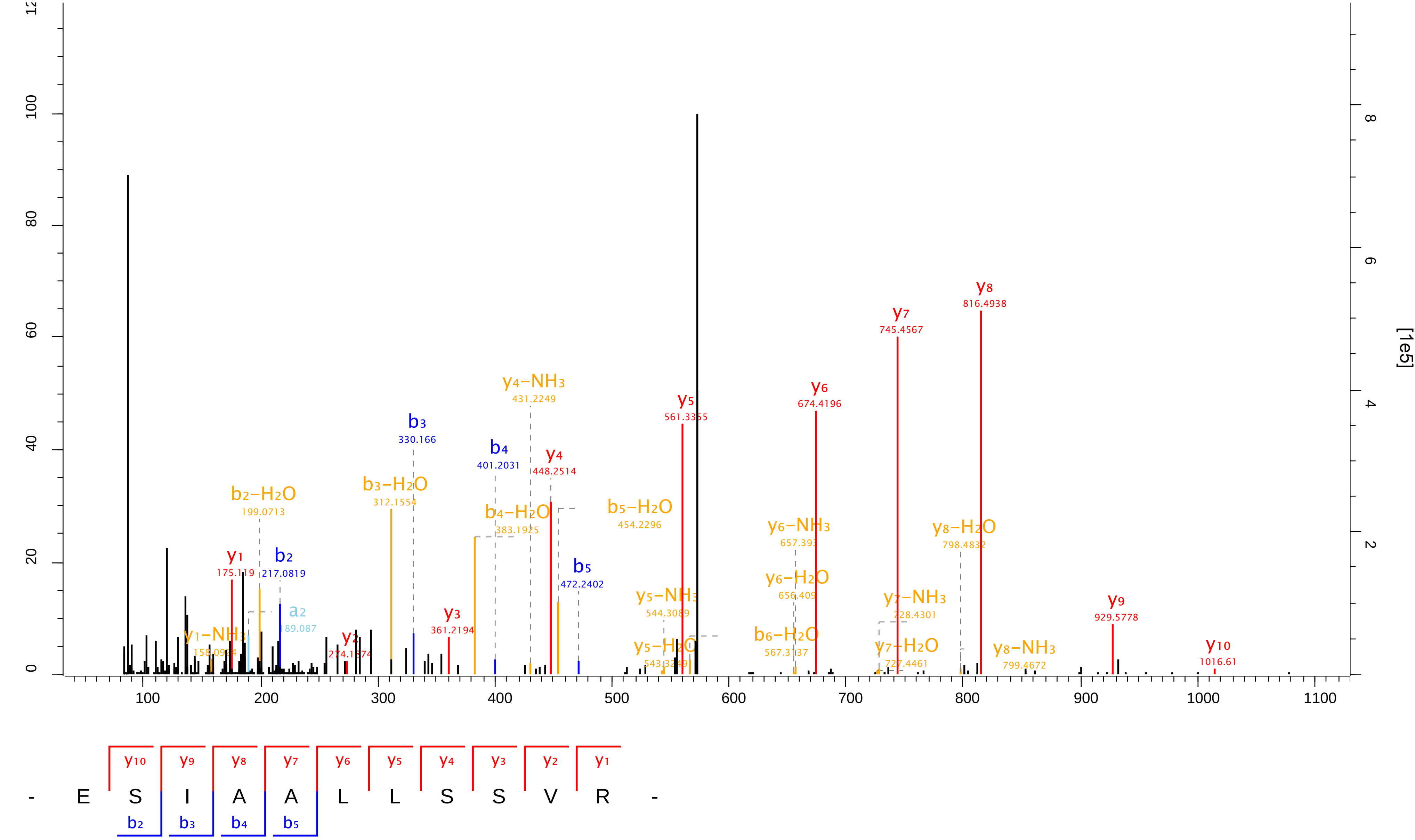

Raw file	Scan	Method	Score	m/z
QEplus003070	12405	FTMS; HCD	70.11	869.46

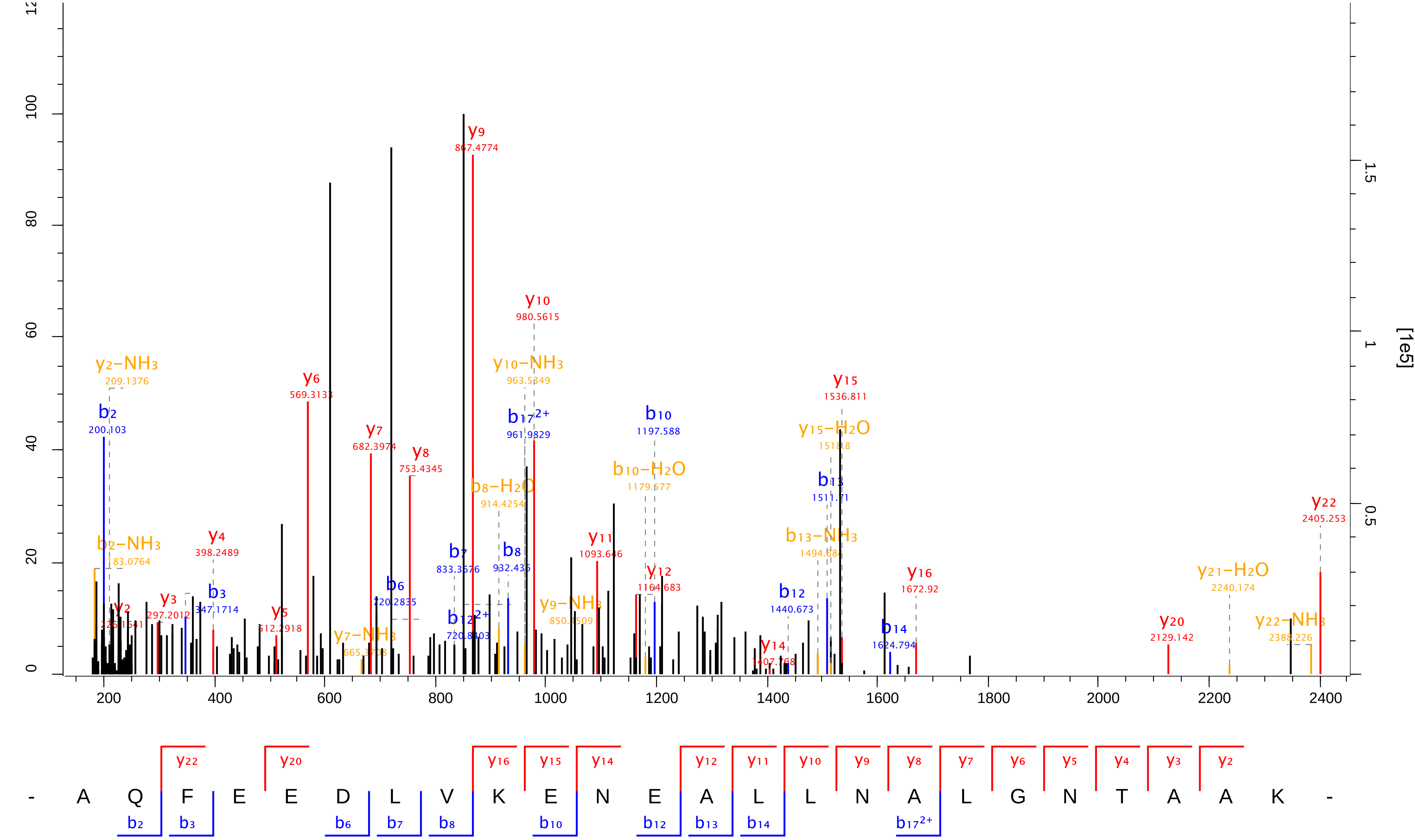

Raw file	Scan	Method	Score	m/z
QEplus003070	14010	FTMS; HCD	159.81	1023.49

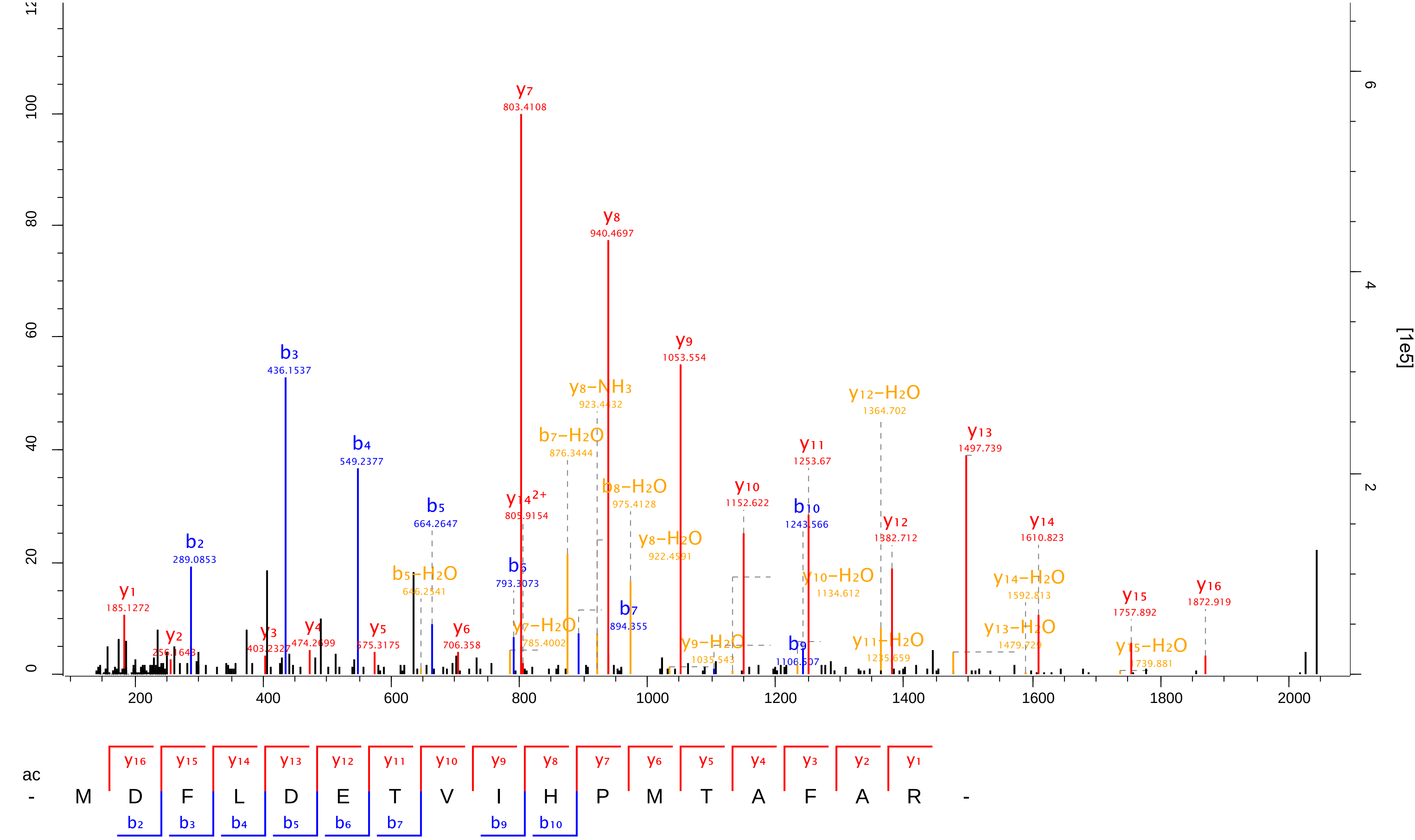

Raw file	Scan	Method	Score	m/z
QEplus003070	14276	FTMS; HCD	135.58	945.57

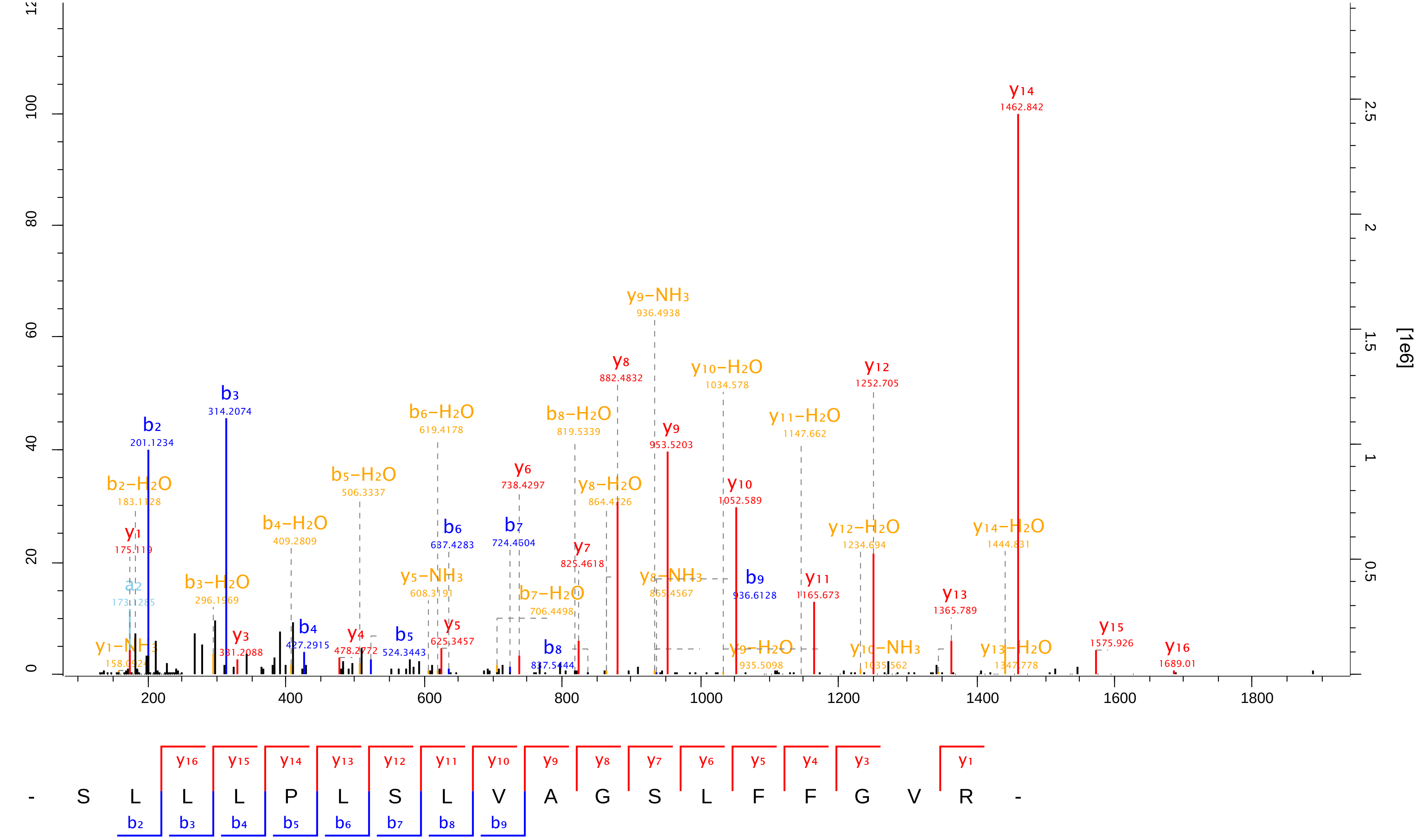

Raw file	Scan	Method	Score	m/z
QEplus003070	14579	FTMS; HCD	119.97	1416.17

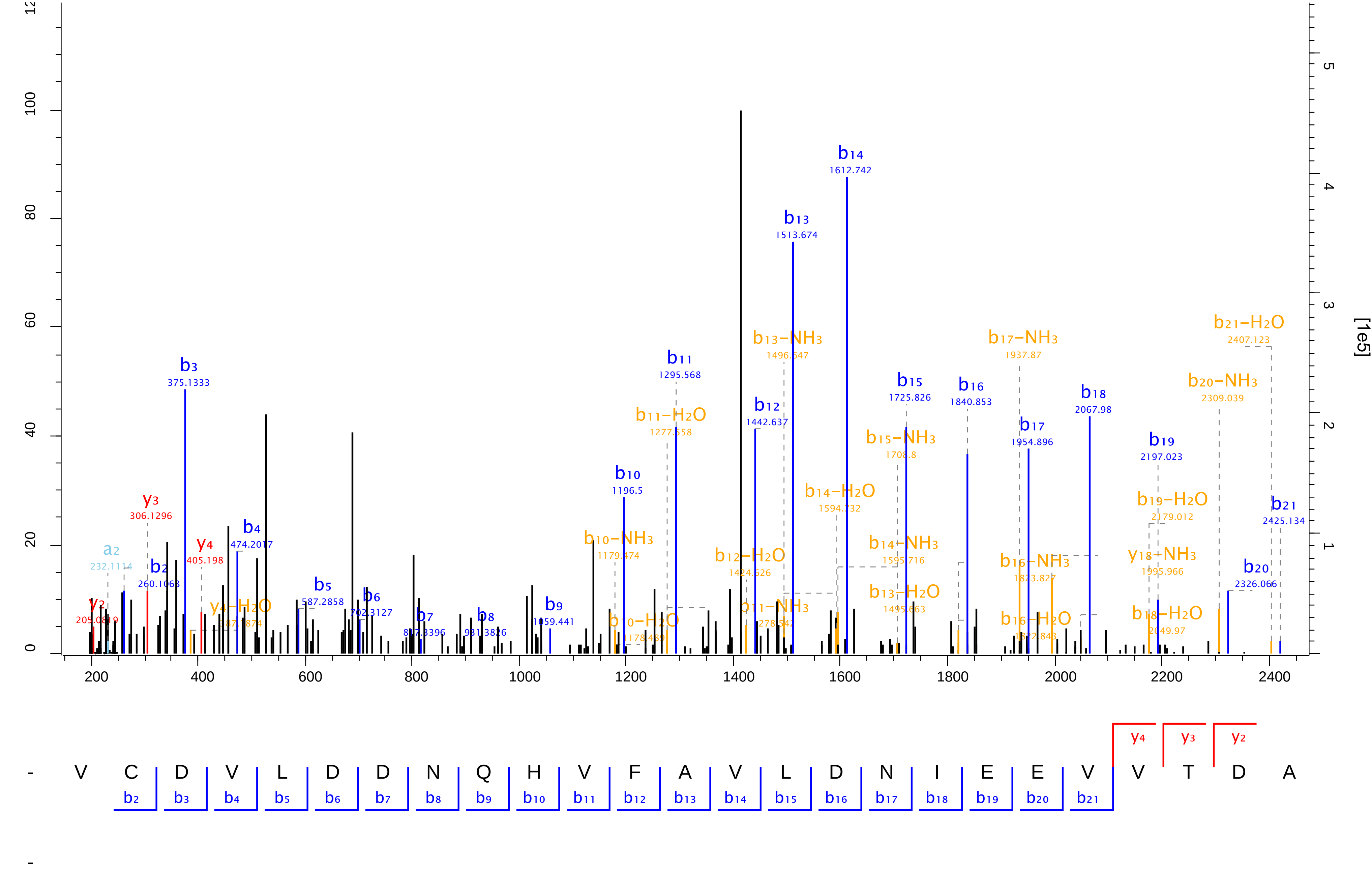

Raw file	Scan	Method	Score	m/z
QEplus003070	14684	FTMS; HCD	68.45	1263.14

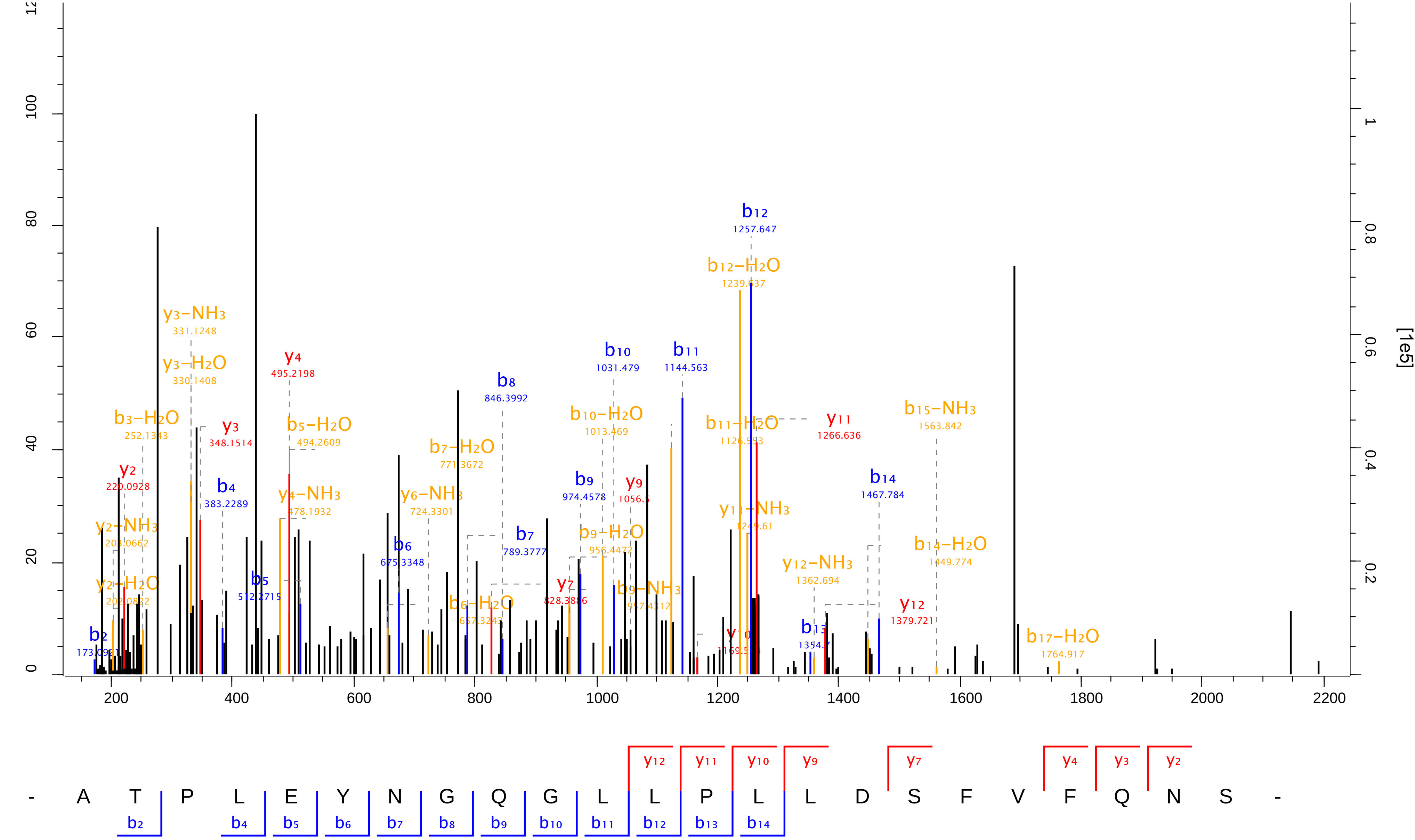

Raw file	Scan	Method	Score	m/z
QEplus003070	3181	FTMS; HCD	79.66	637.36

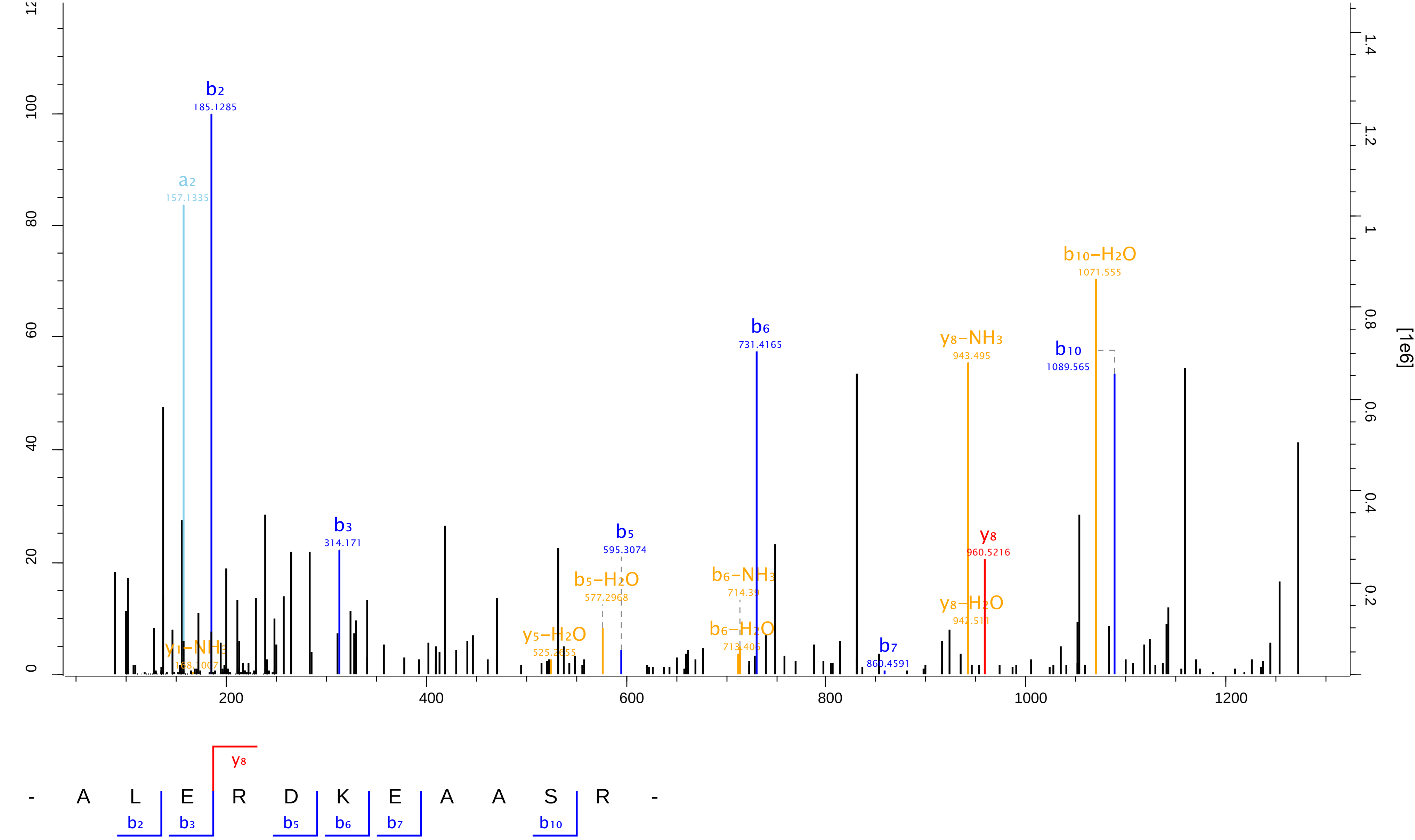

Raw file	Scan	Method	Score	m/z
QEplus003070	5224	FTMS; HCD	61.96	679.38

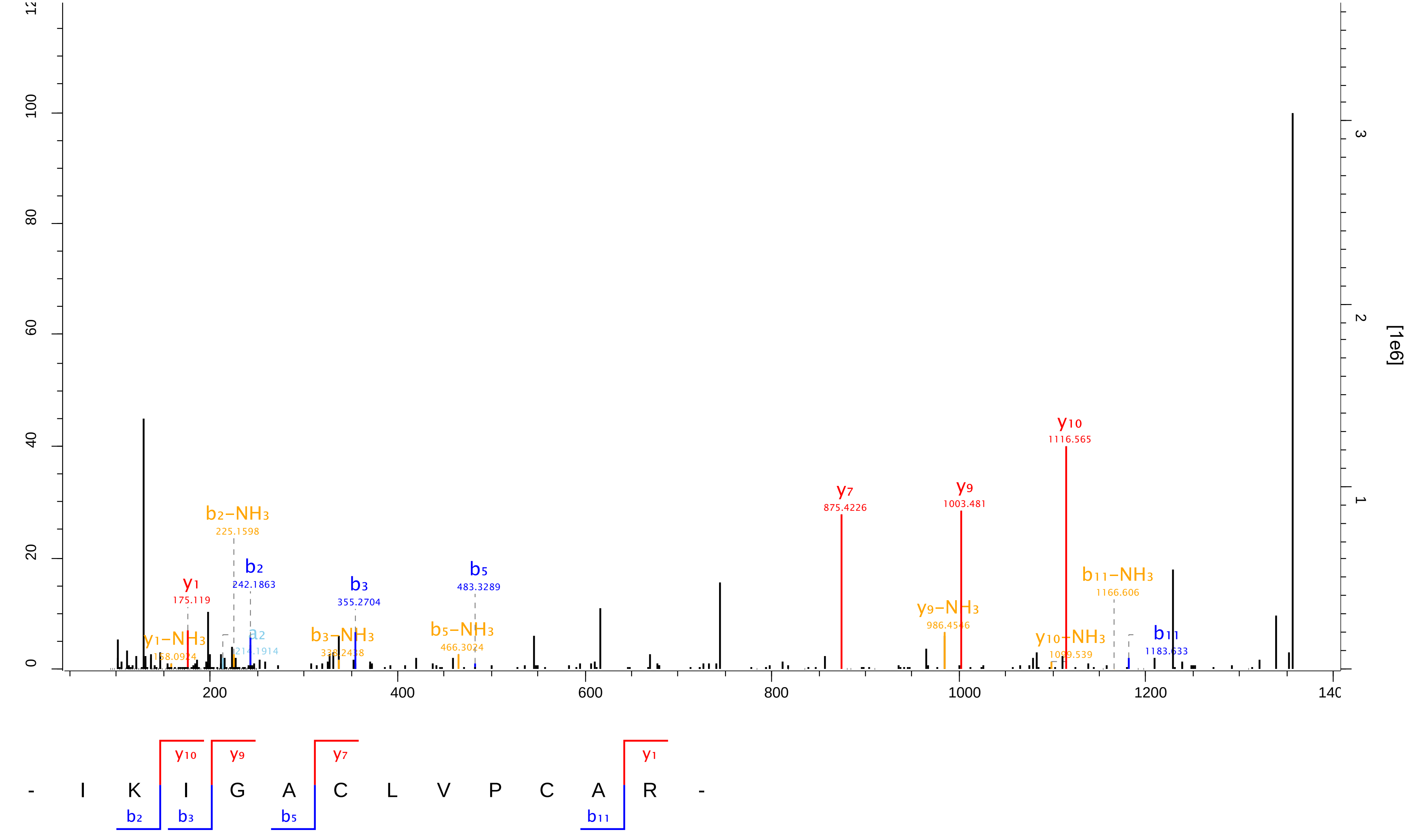

Raw file	Scan	Method	Score	m/z
QEplus003070	7956	FTMS; HCD	48.89	676.41

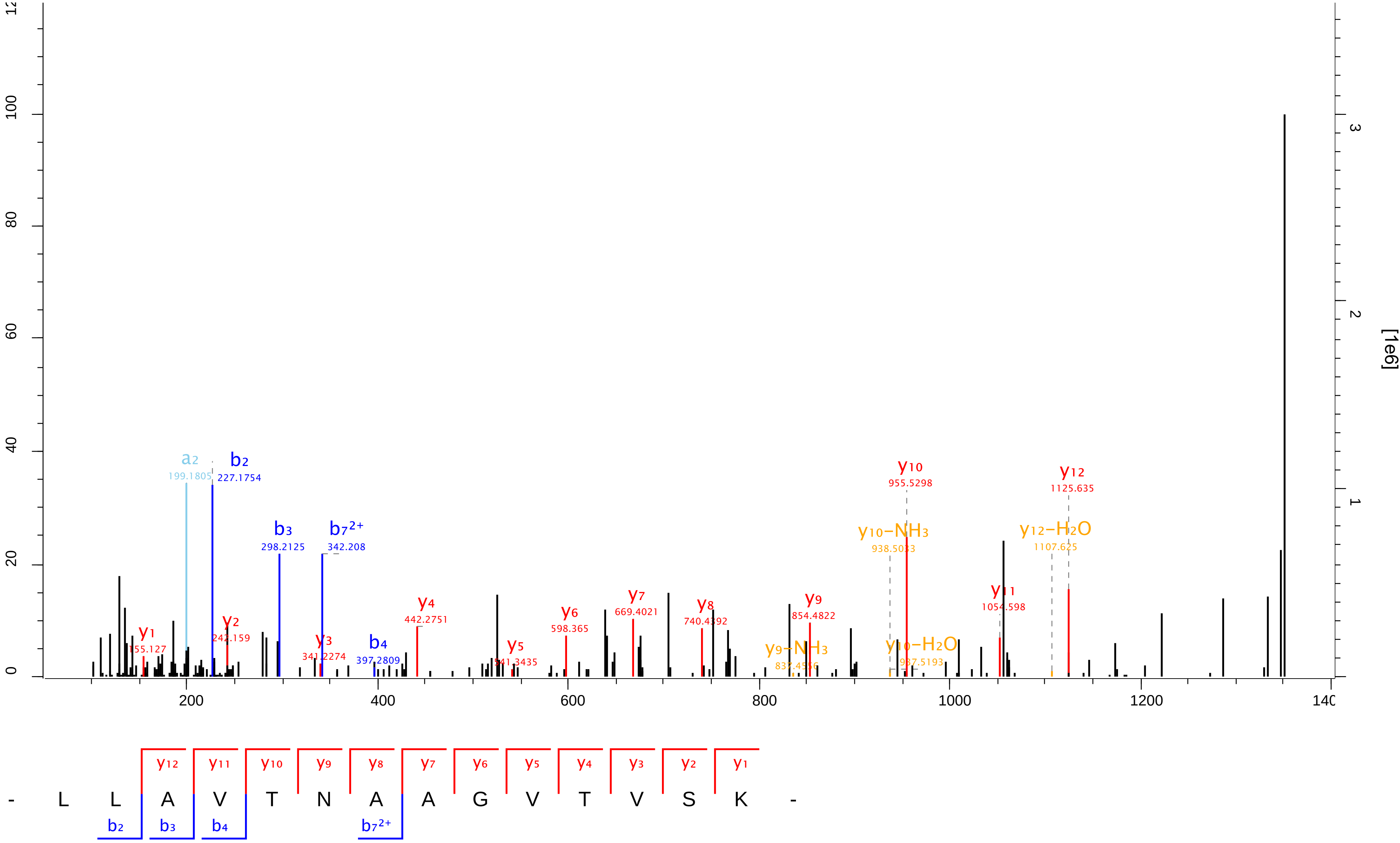

Raw file	Scan	Method	Score	m/z
QEplus003070	8421	FTMS; HCD	104.21	777.91

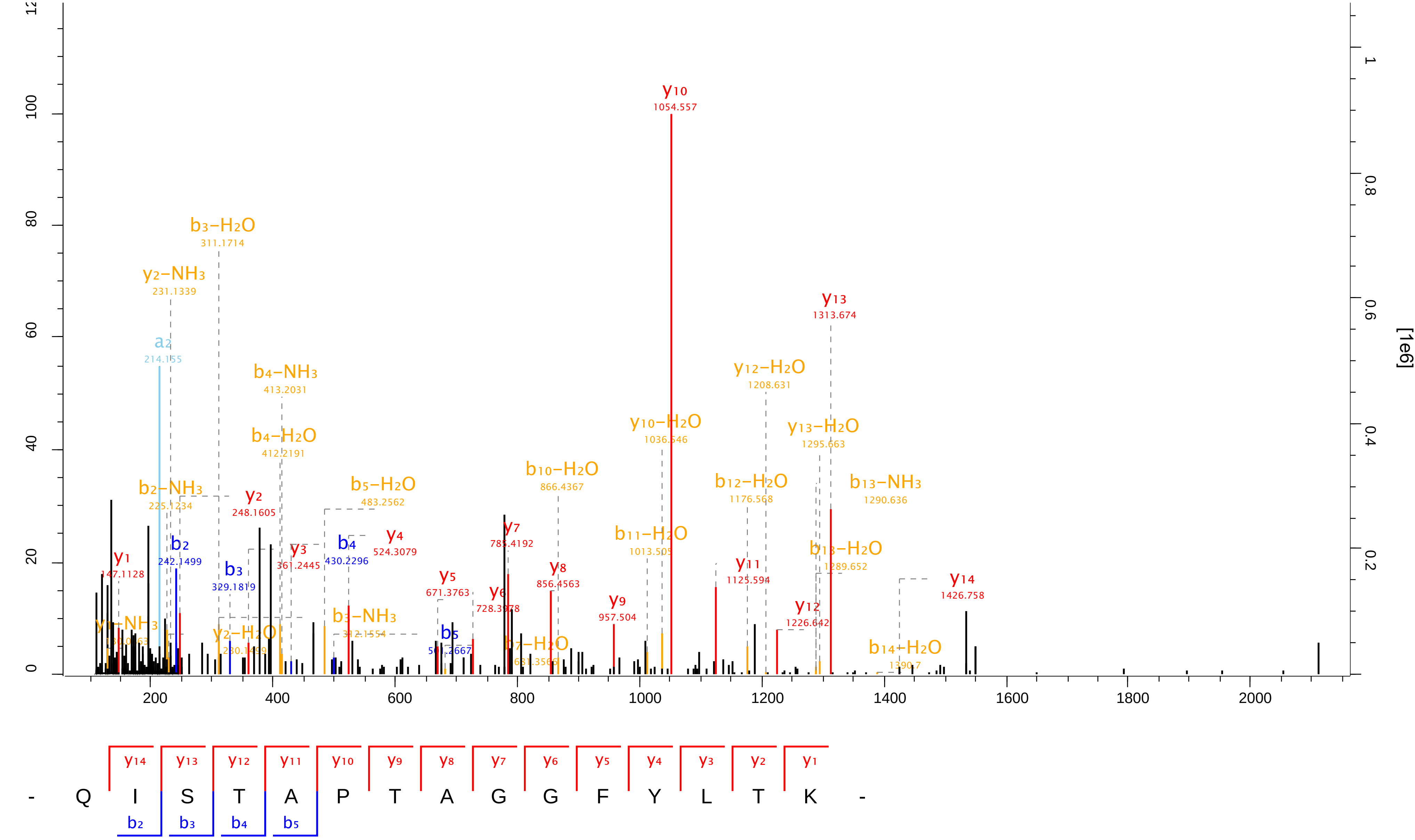

Raw file	Scan	Method	Score	m/z
QEplus003070	9389	FTMS; HCD	60.55	760.42

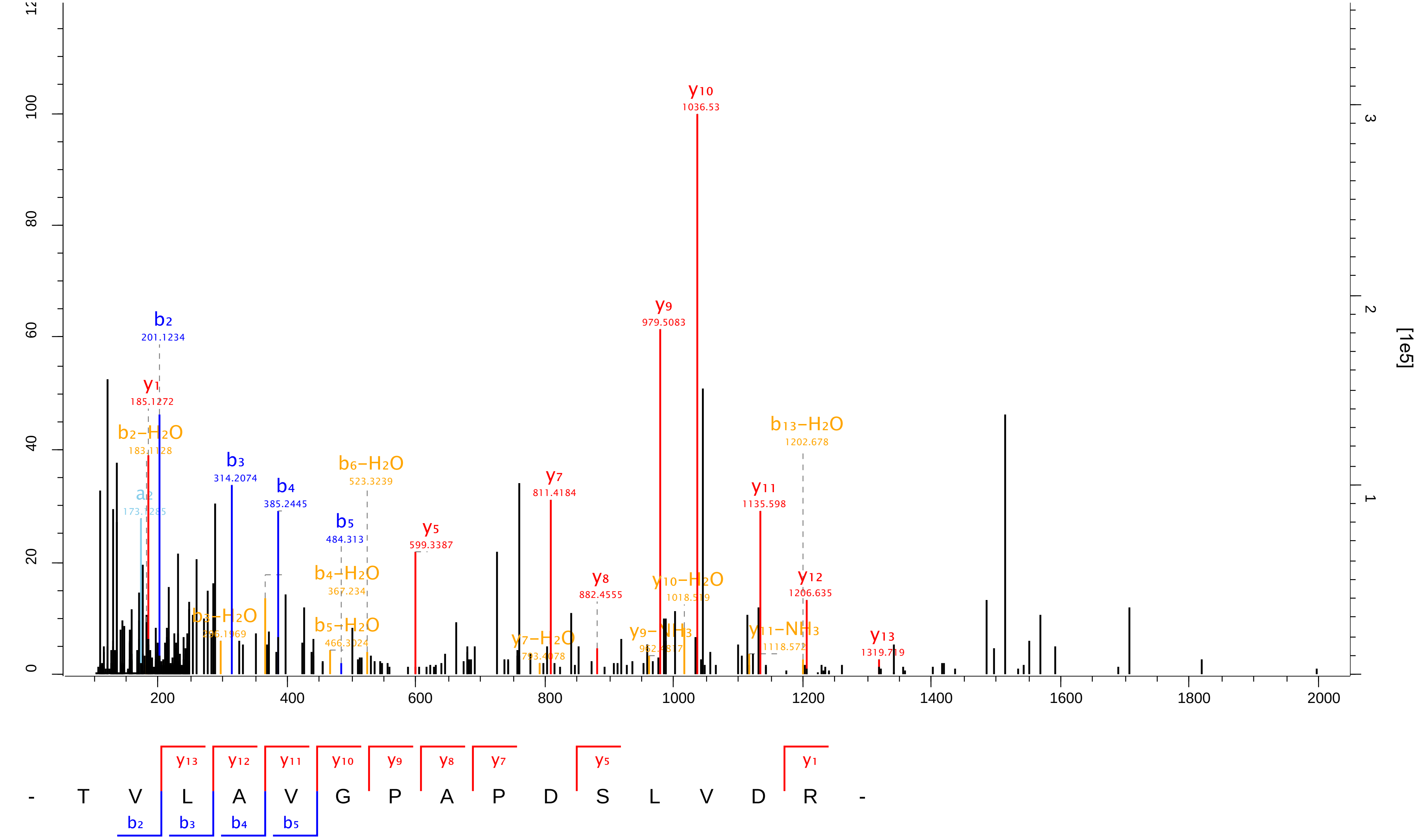

Raw file	Scan	Method	Score	m/z
QEplus003070	9537	FTMS; HCD	67.85	677.37

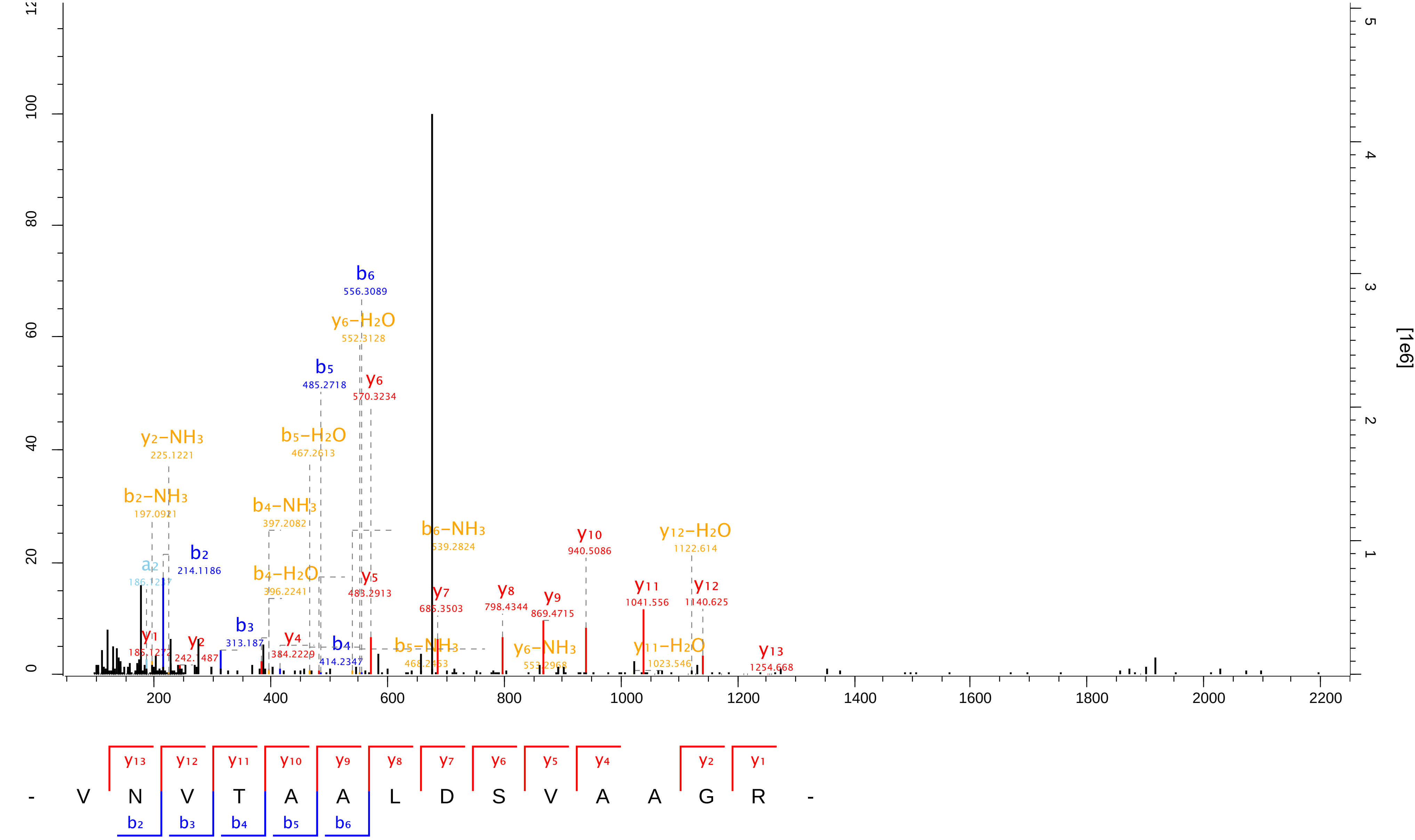

Raw file	Scan	Method	Score	m/z
QEplus003073	10478	FTMS; HCD	119.29	518.29

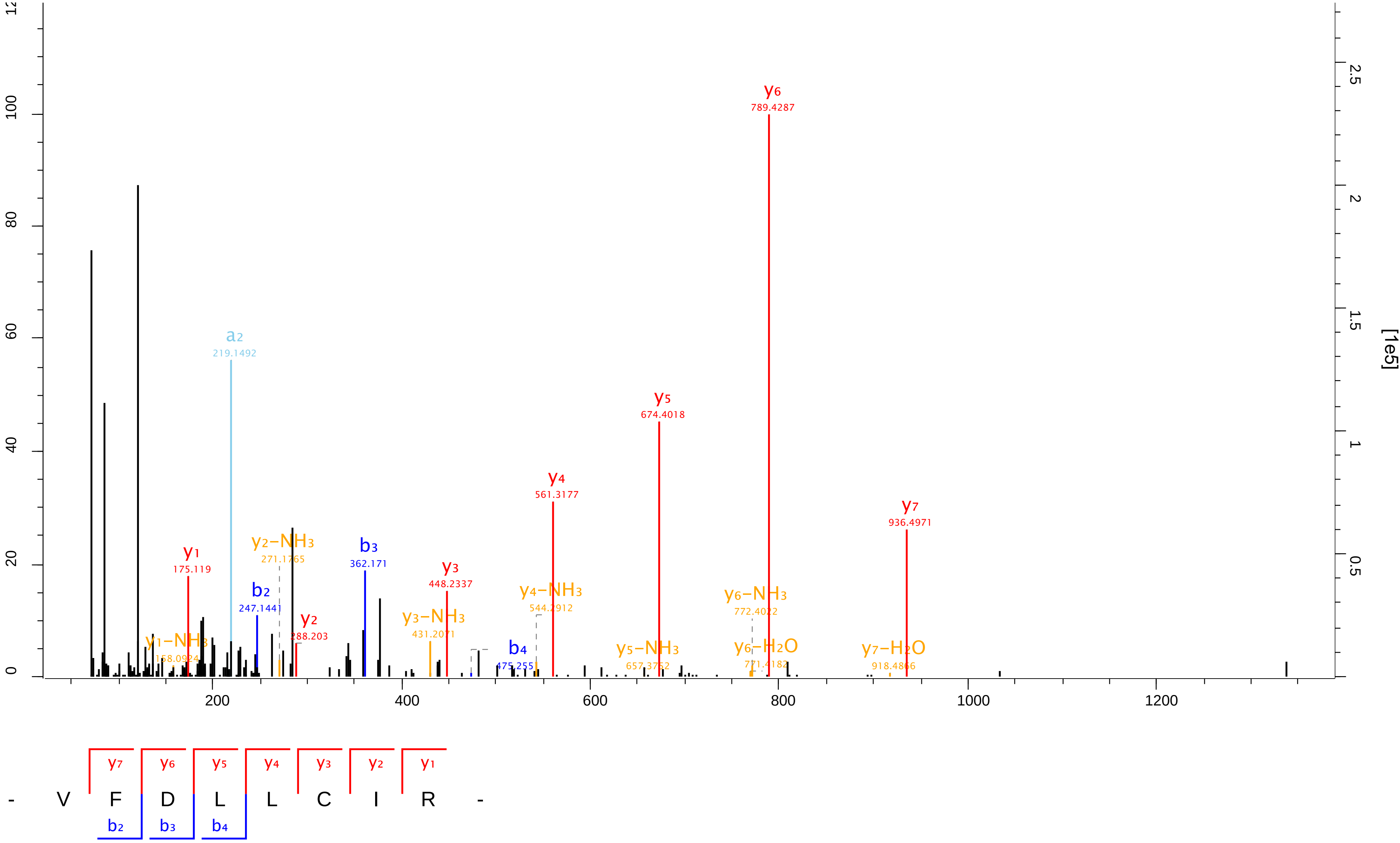

Raw file	Scan	Method	Score	m/z
QEplus003073	7464	FTMS; HCD	121.87	432.74

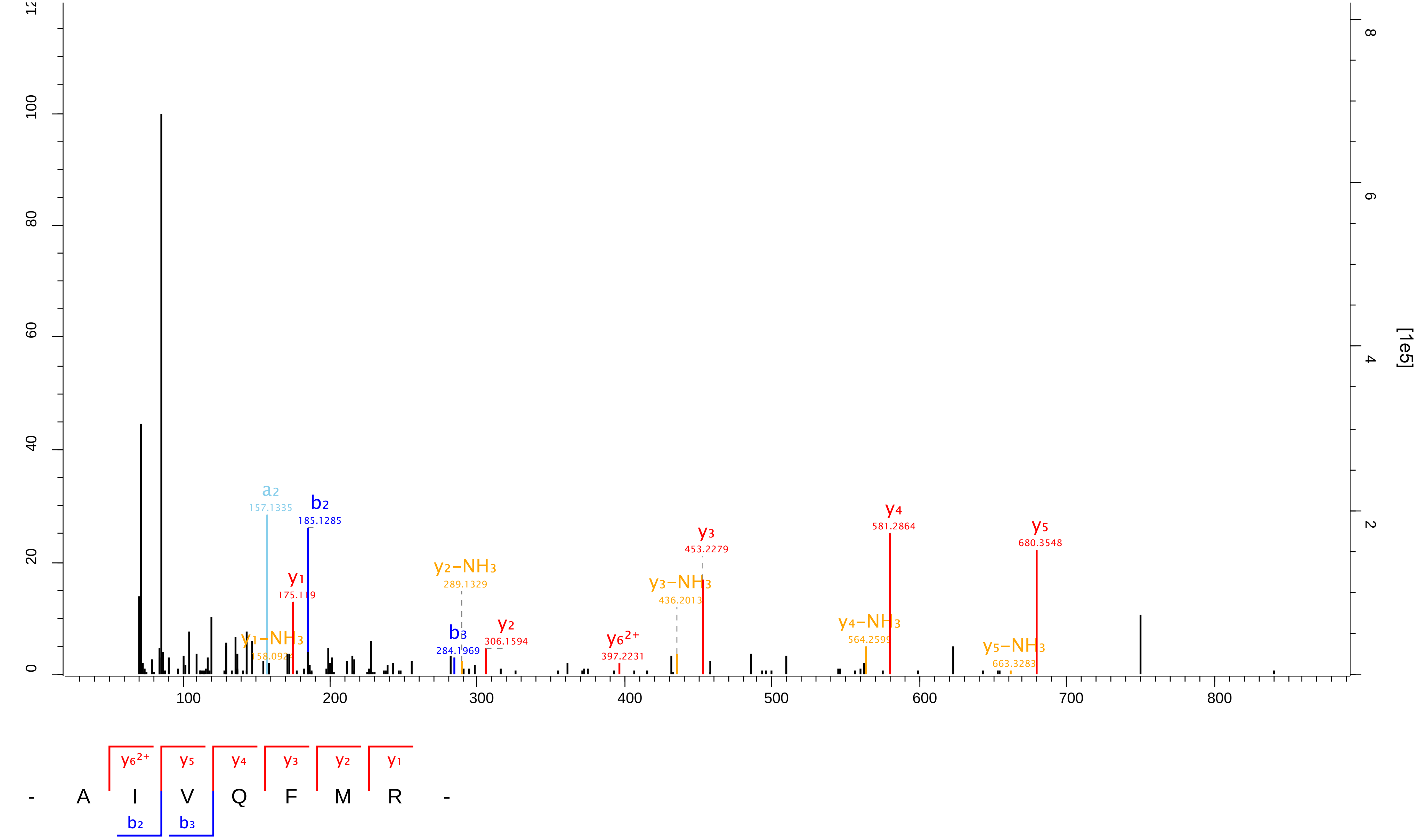

Raw file	Scan	Method	Score	m/z
QEplus003075	7515	FTMS; HCD	58.6	447.3

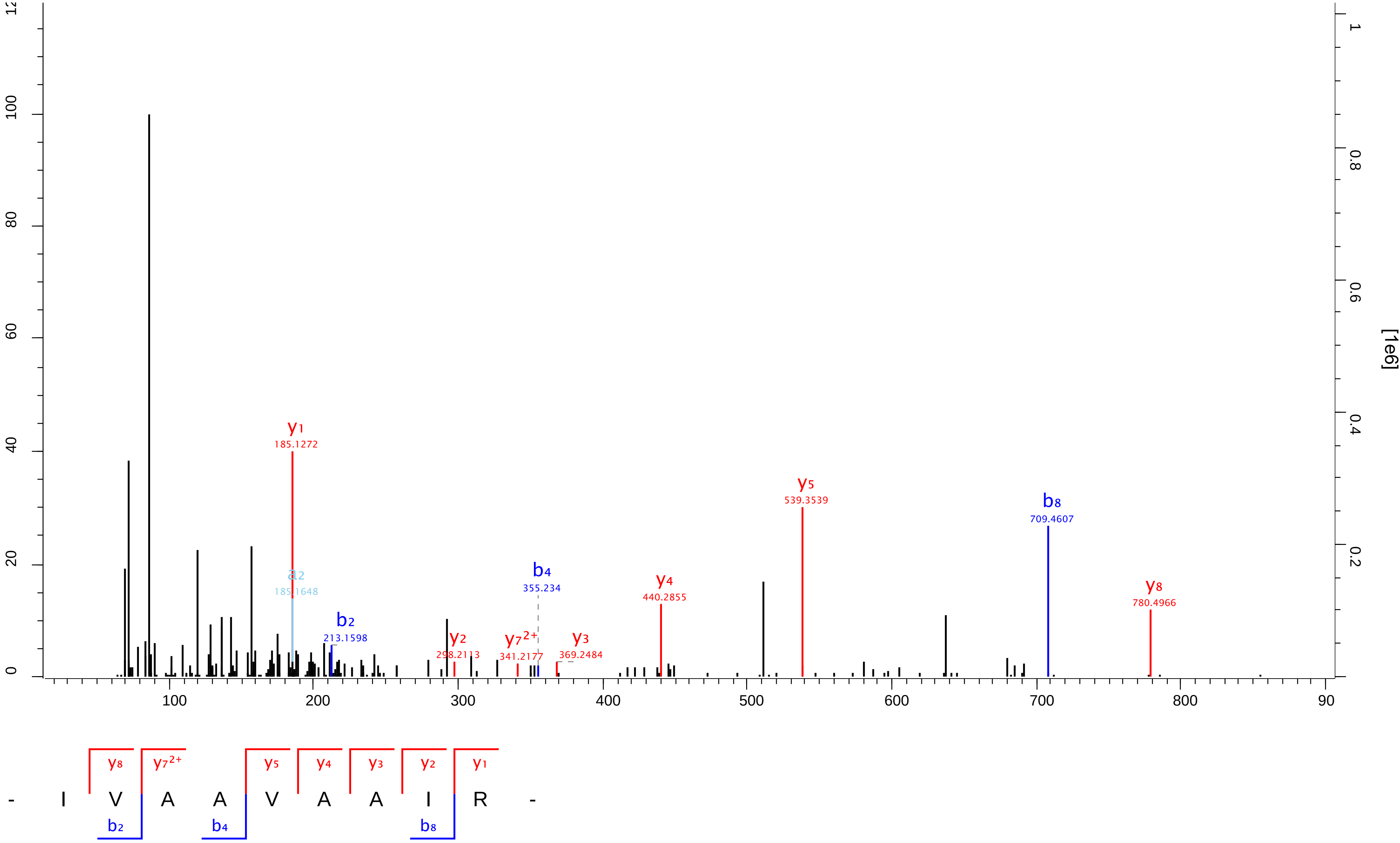

Raw file	Scan	Method	Score	m/z
QEplus003076	13733	FTMS; HCD	75.19	501.29

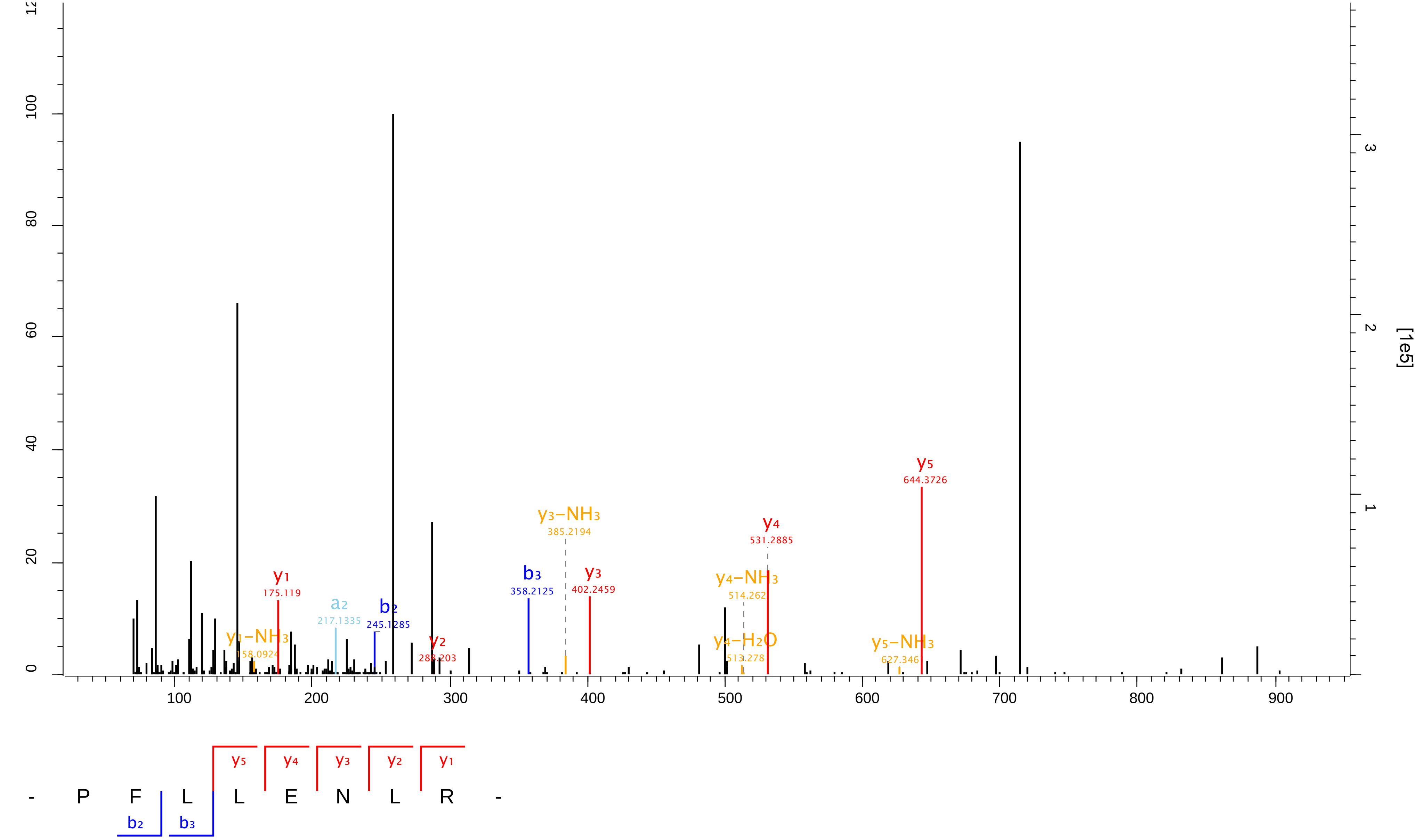

Raw file	Scan	Method	Score	m/z
QEplus003076	13977	FTMS; HCD	125.9	835.47

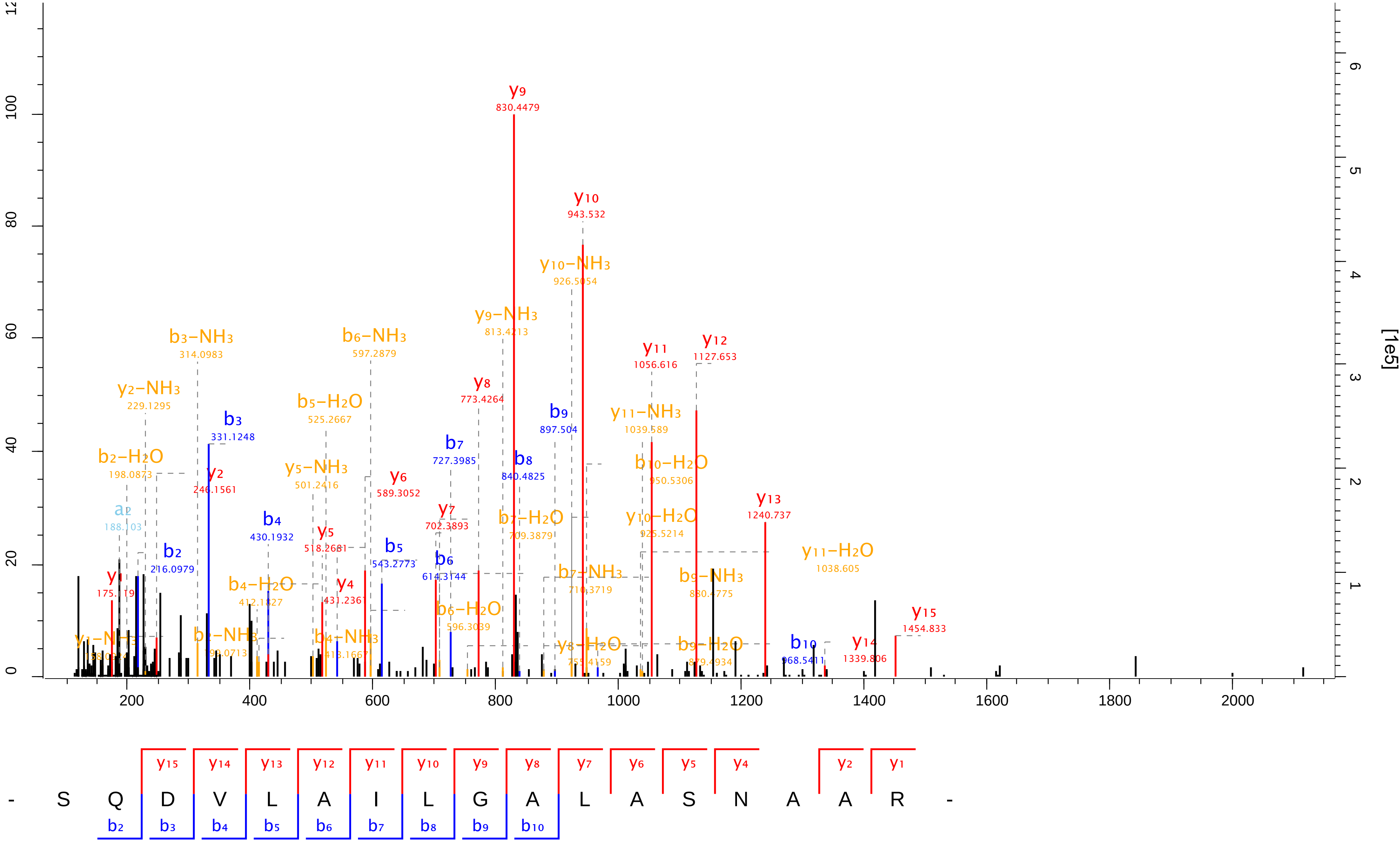

Raw file	Scan	Method	Score	m/z
QEplus003077	11613	FTMS; HCD	68.66	565.34

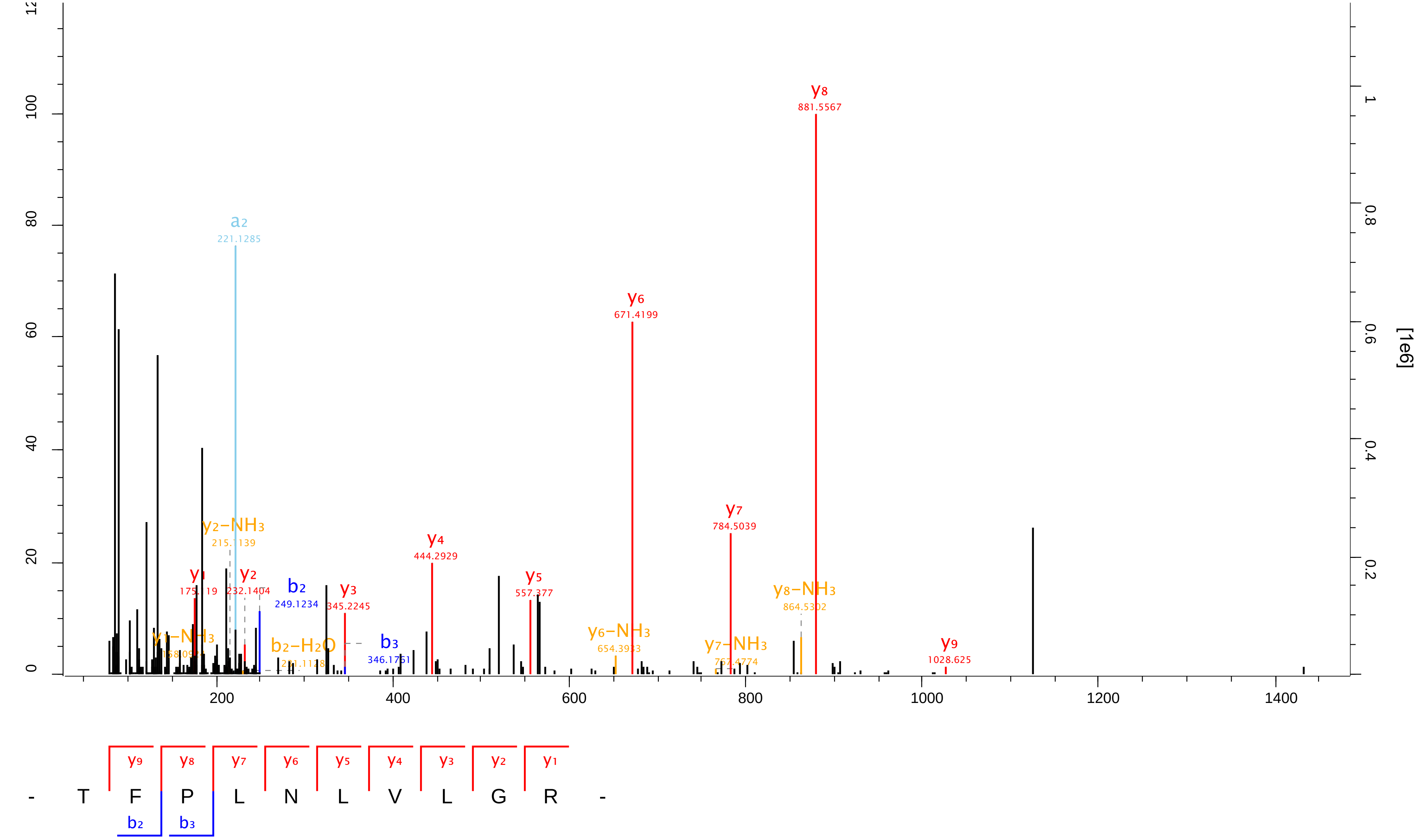

Raw file	Scan	Method	Score	m/z
QEplus003077	13377	FTMS; HCD	99.28	584.89

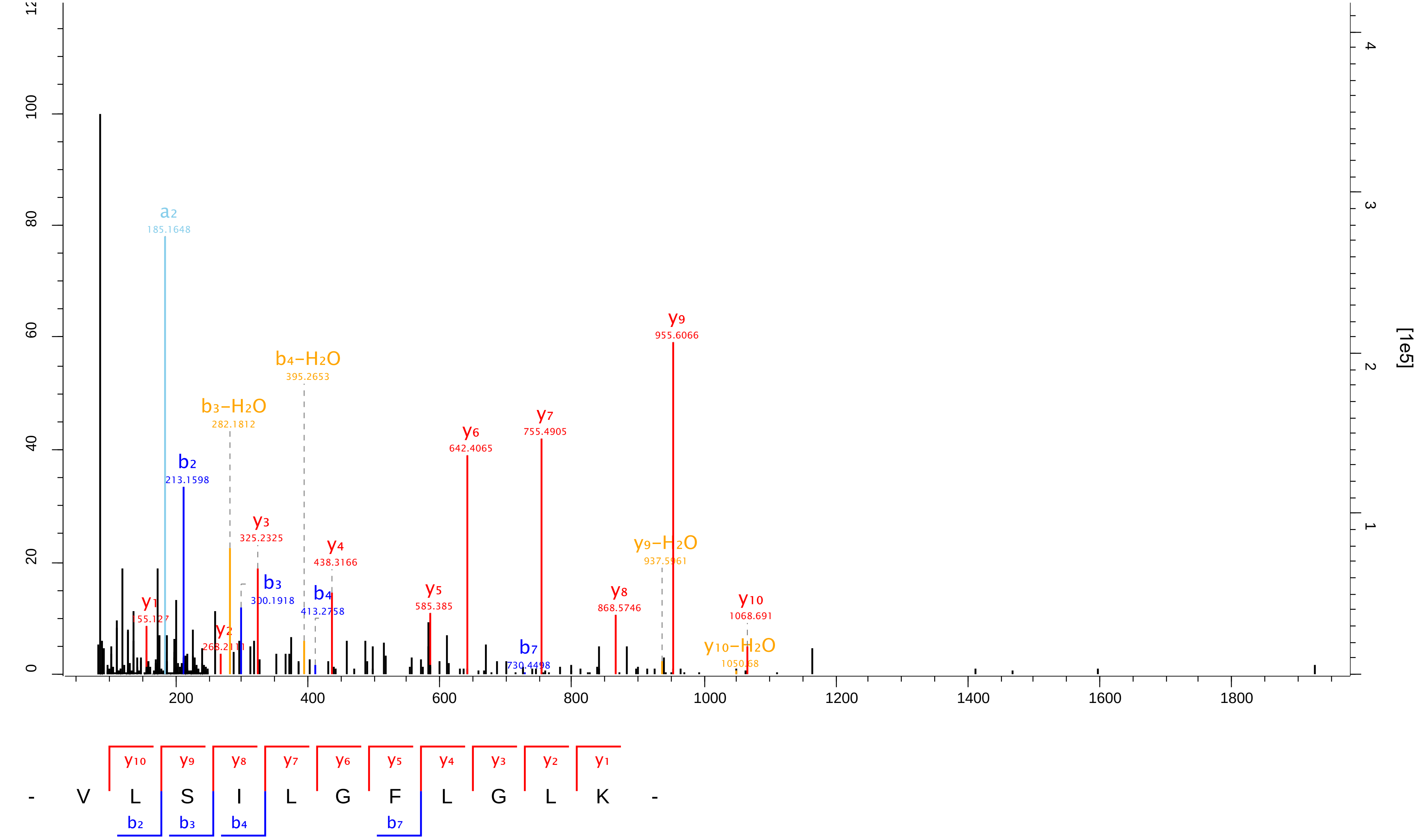

Raw file	Scan	Method	Score	m/z
QEplus003077	13635	FTMS; HCD	114.55	1011.04

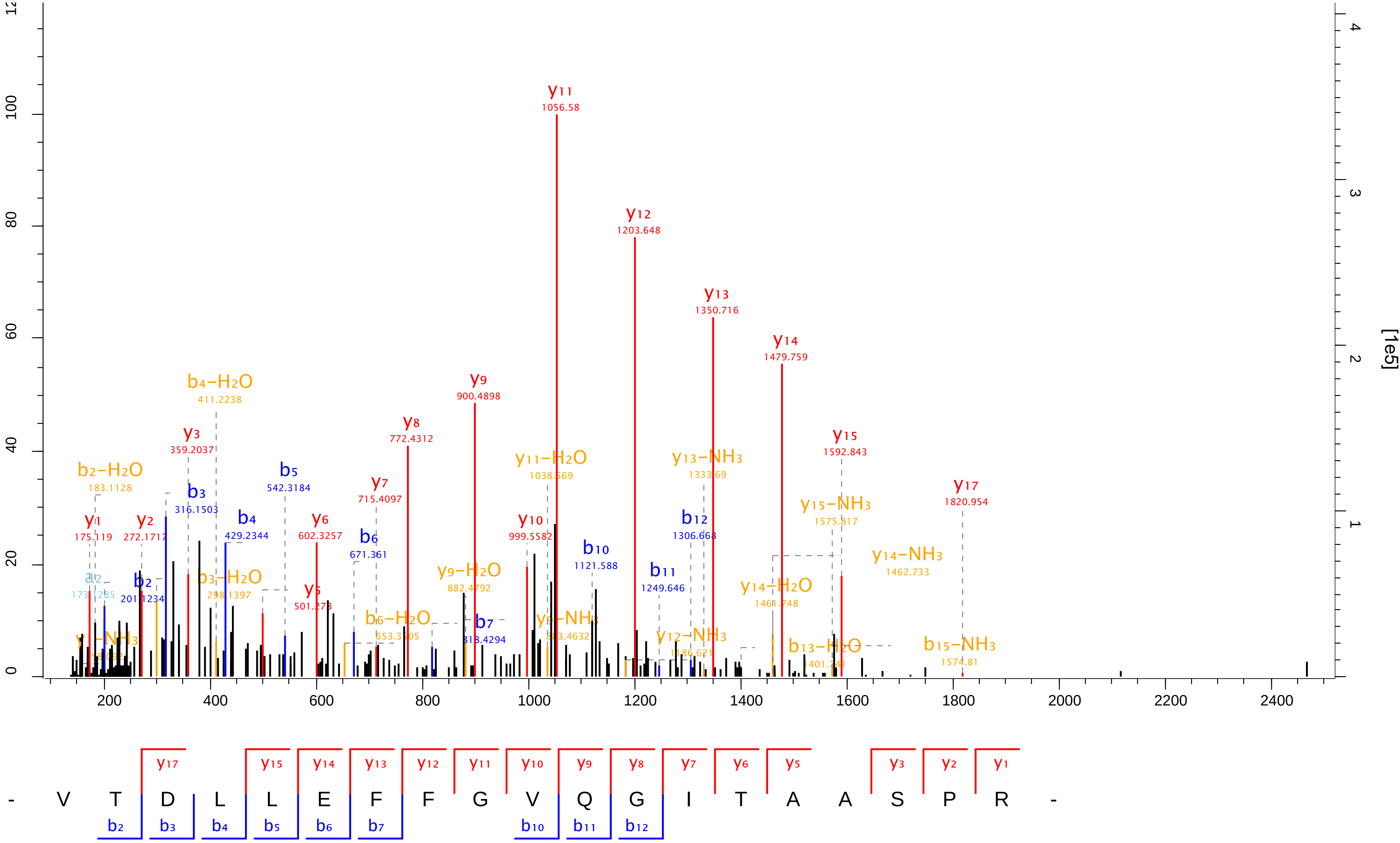

Raw file	Scan	Method	Score	m/z
QEplus003077	13997	FTMS; HCD	44.39	910.05

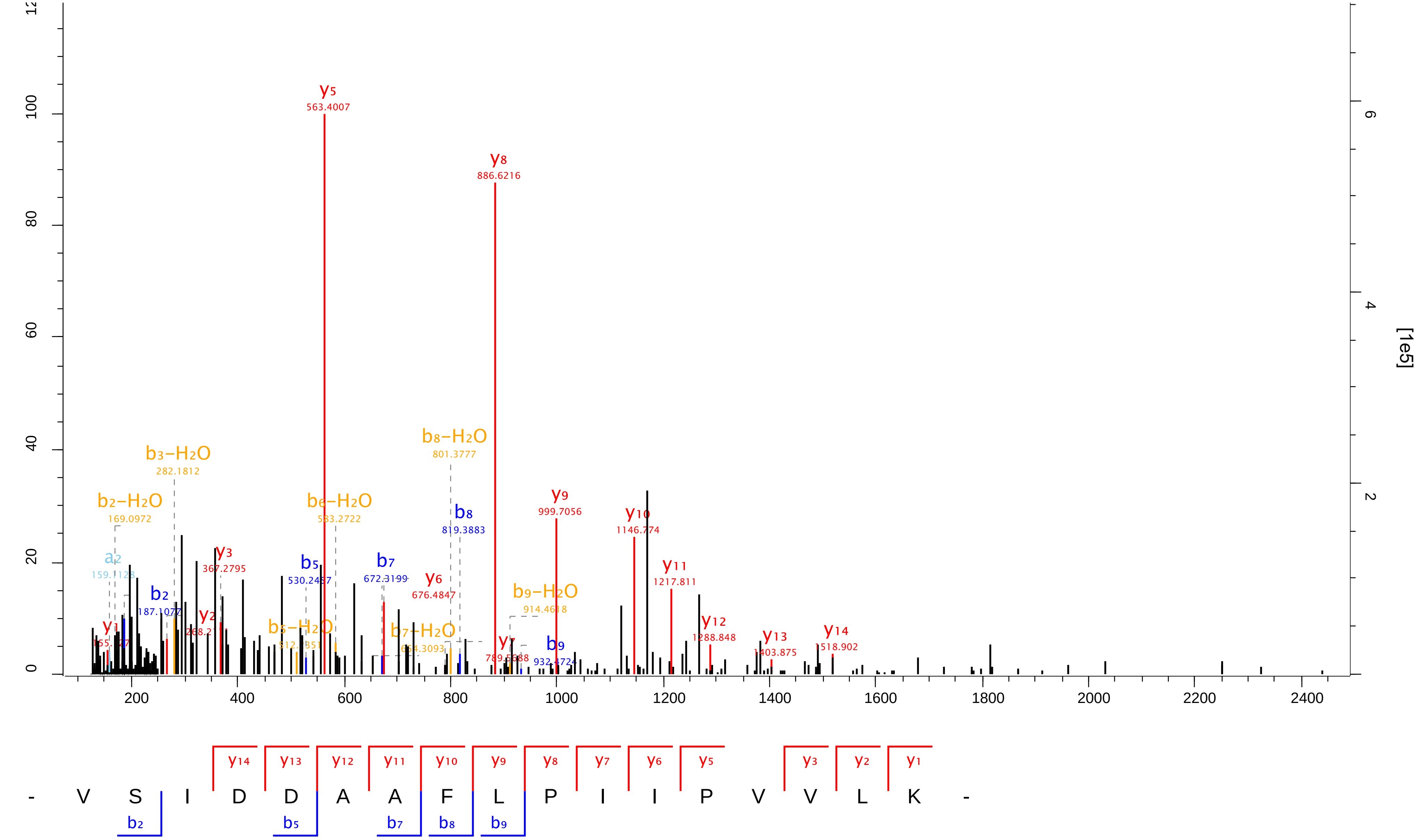

Raw file	Scan	Method	Score	m/z
QEplus003077	14259	FTMS; HCD	77.75	804.41

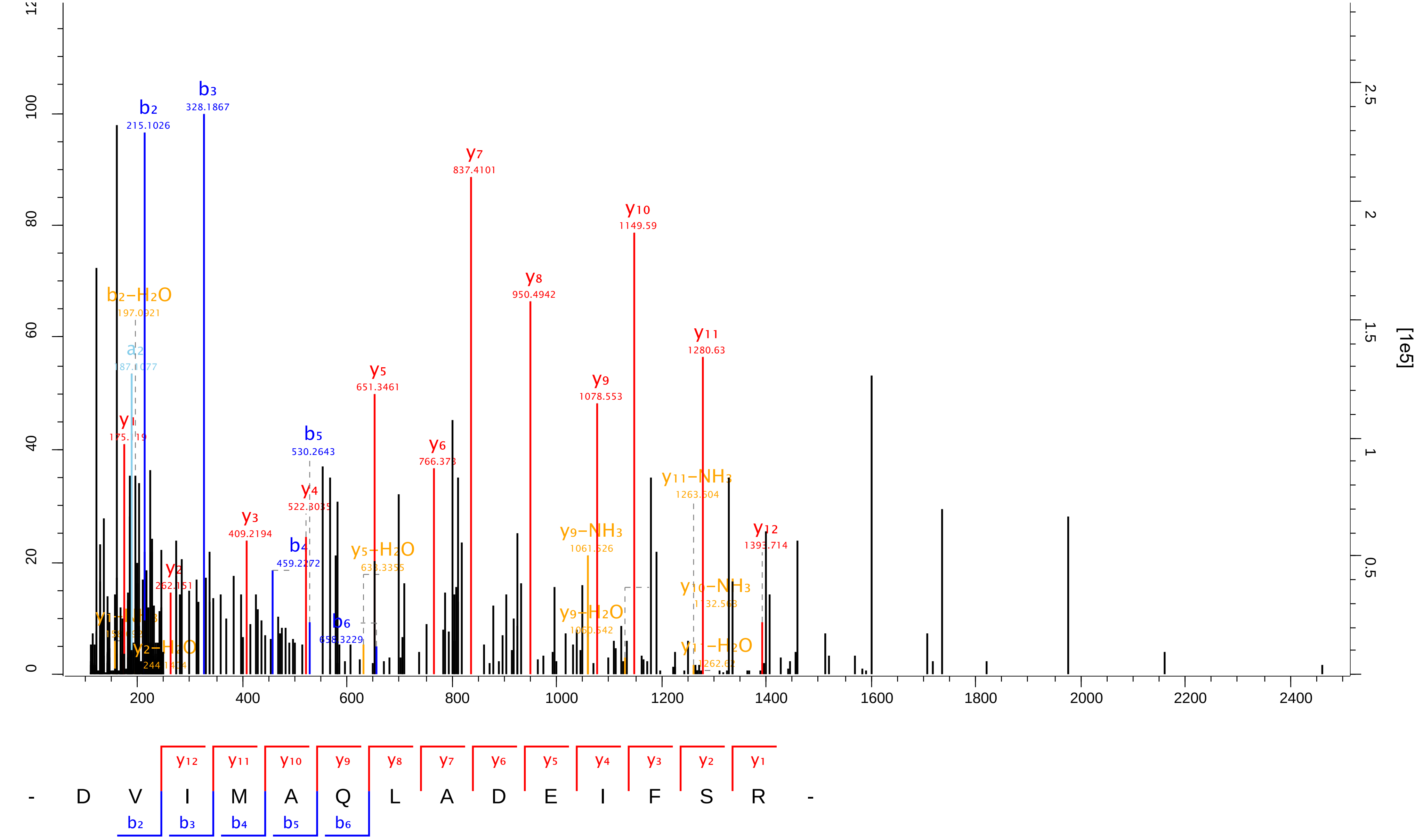

Raw file	Scan	Method	Score	m/z
QEplus003077	3457	FTMS; HCD	74.85	526.6

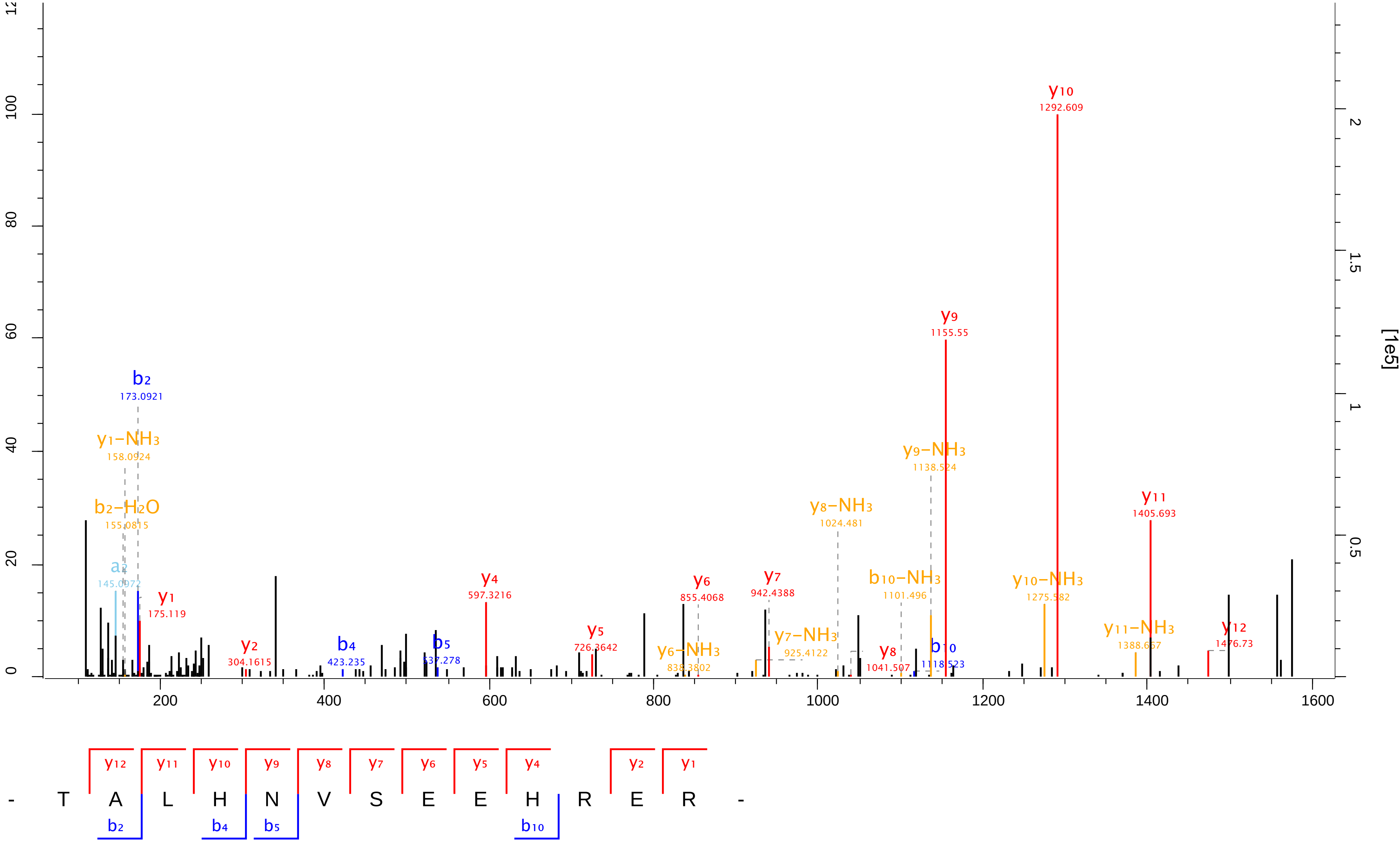

Raw file	Scan	Method	Score	m/z
QEplus003077	4200	FTMS; HCD	104.55	753.88

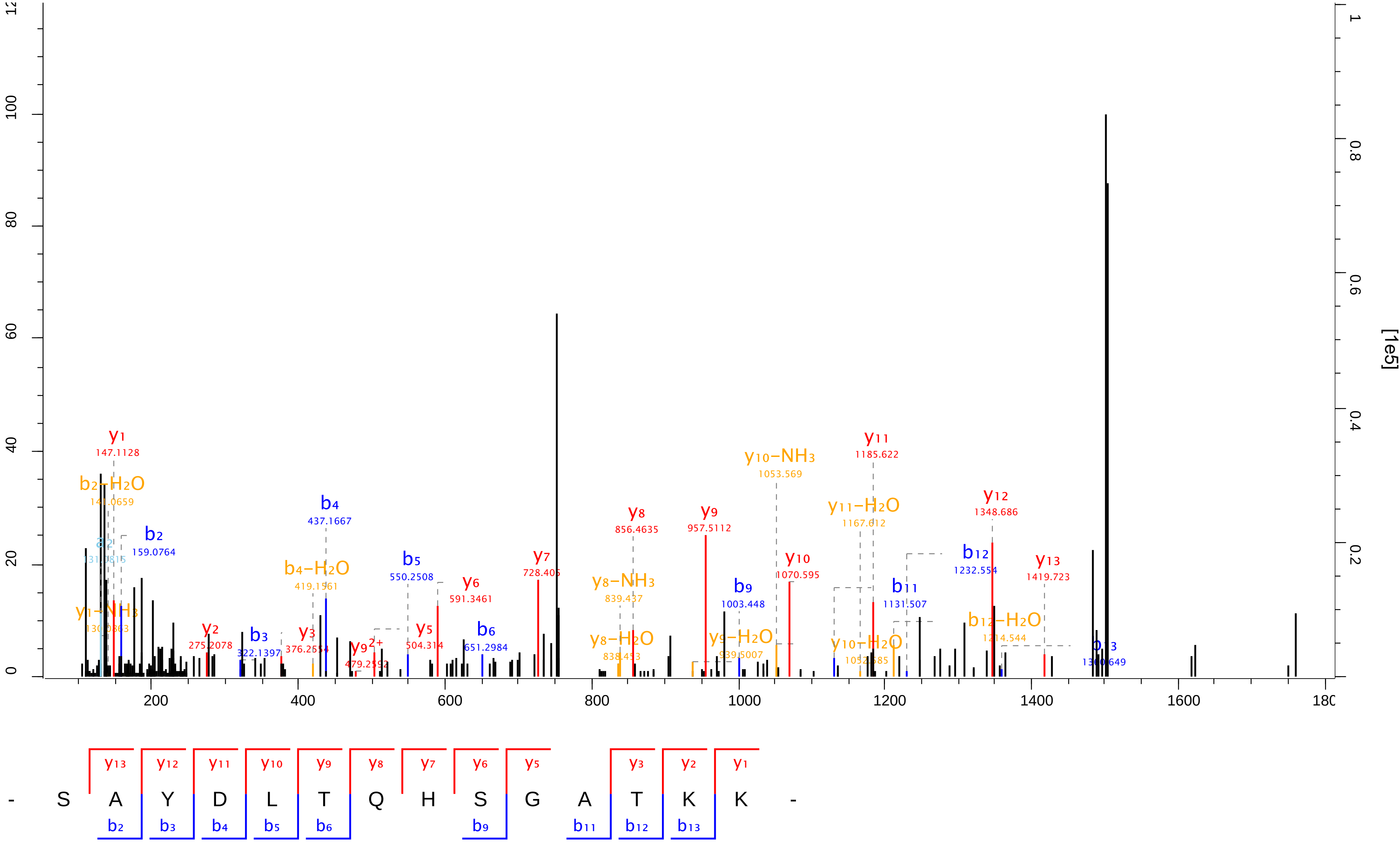

Raw file	Scan	Method	Score	m/z
QEplus003078	10424	FTMS; HCD	44.13	803.97

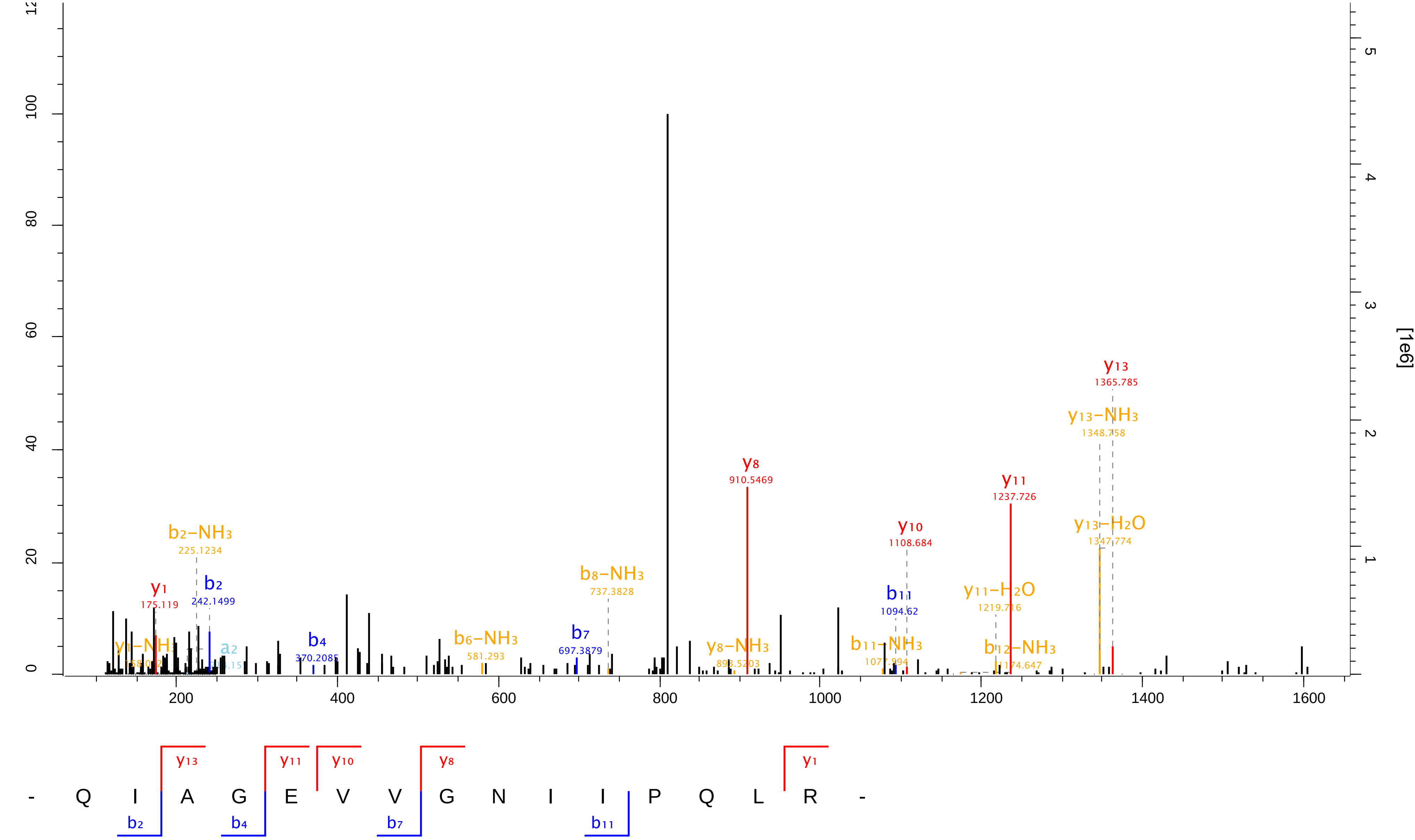

Raw file	Scan	Method	Score	m/z
QEplus003078	14197	FTMS; HCD	120.27	982.89

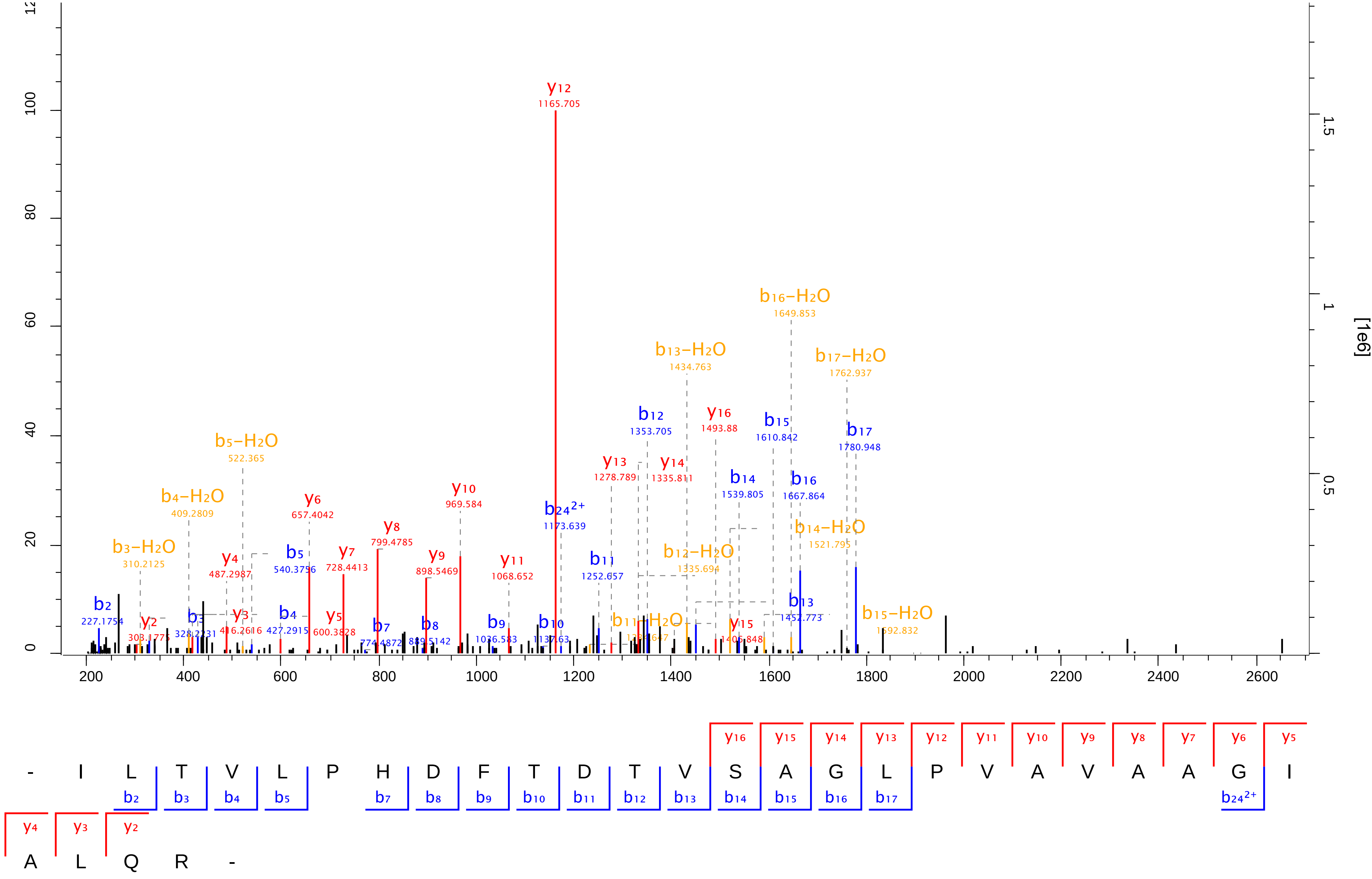

Raw file	Scan	Method	Score	m/z
QEplus003079	11306	FTMS; HCD	89.3	669.36

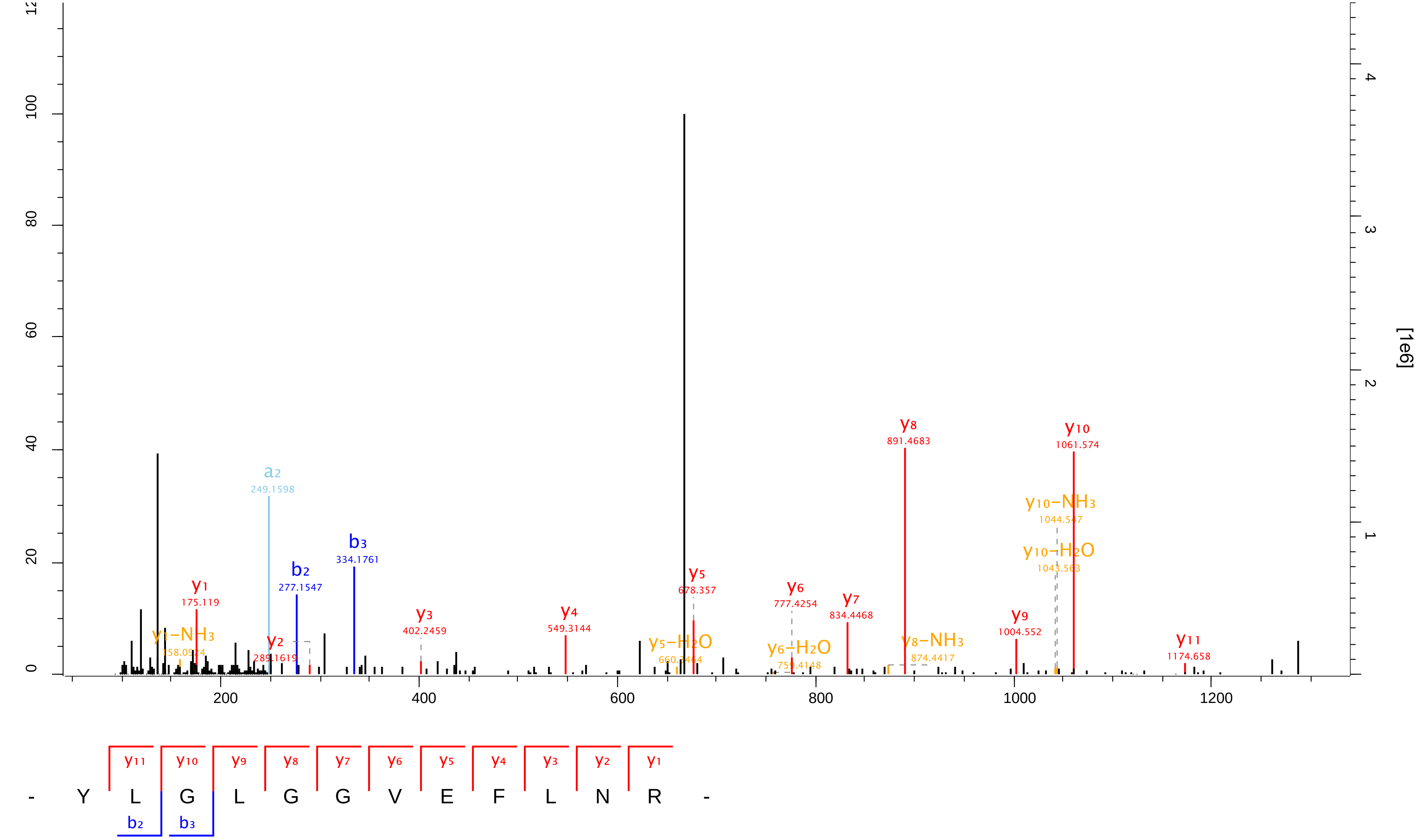

Raw file	Scan	Method	Score	m/z
QEplus003079	11982	FTMS; HCD	66.98	652.34

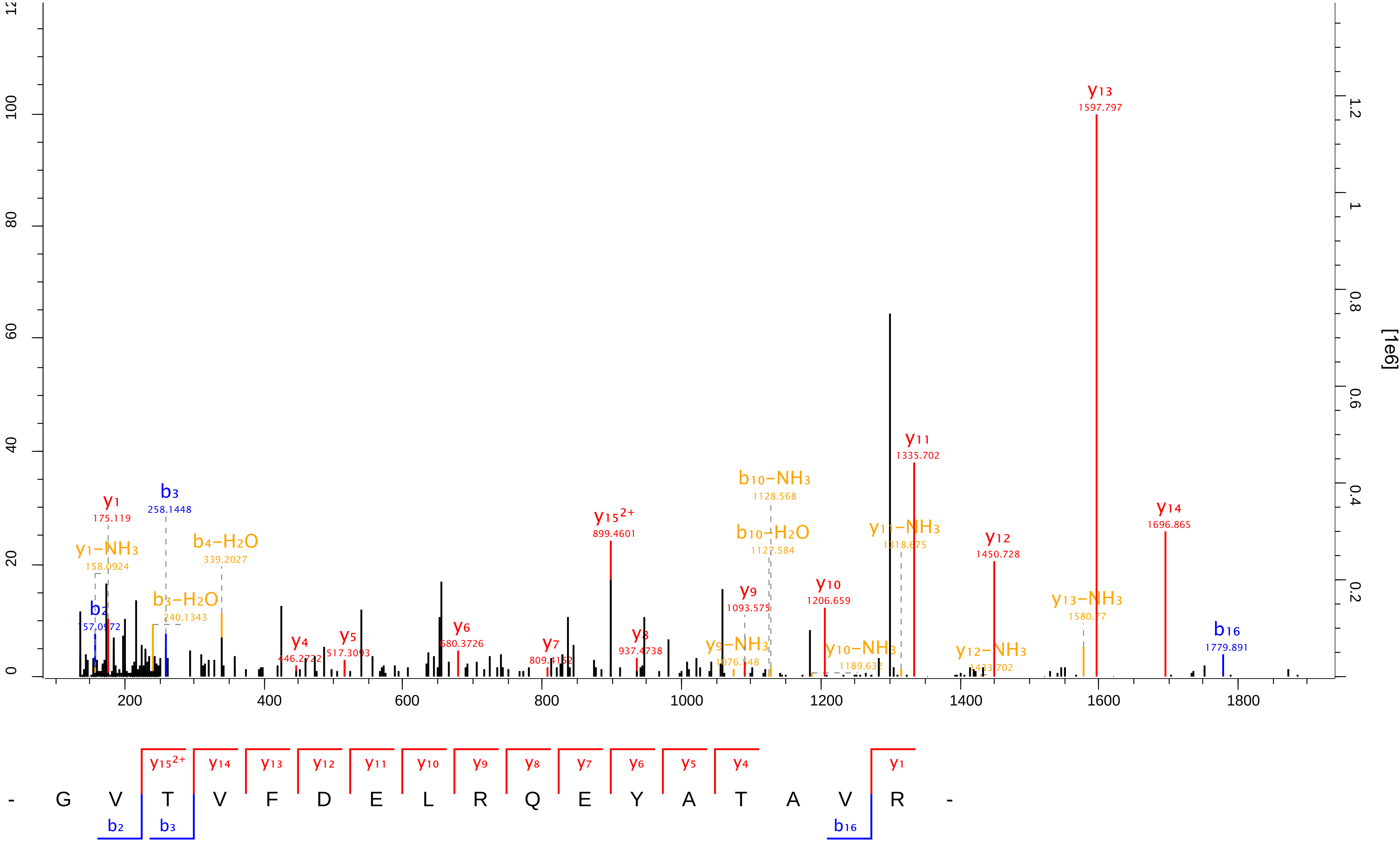

Raw file	Scan	Method	Score	m/z
QEplus003079	7394	FTMS; HCD	56.29	625.99

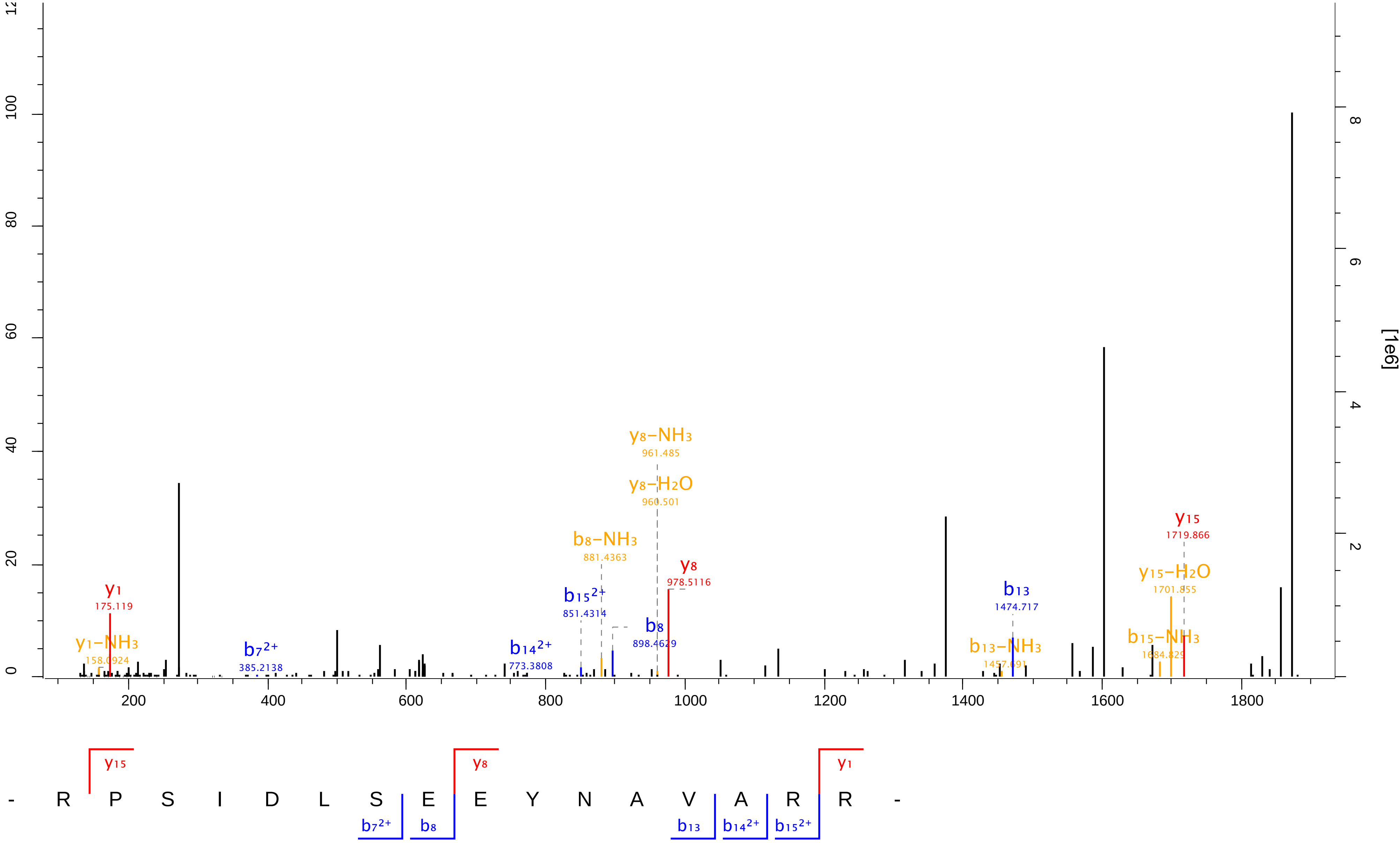

Raw file	Scan	Method	Score	m/z
QEplus003079	9426	FTMS; HCD	65.54	528.33

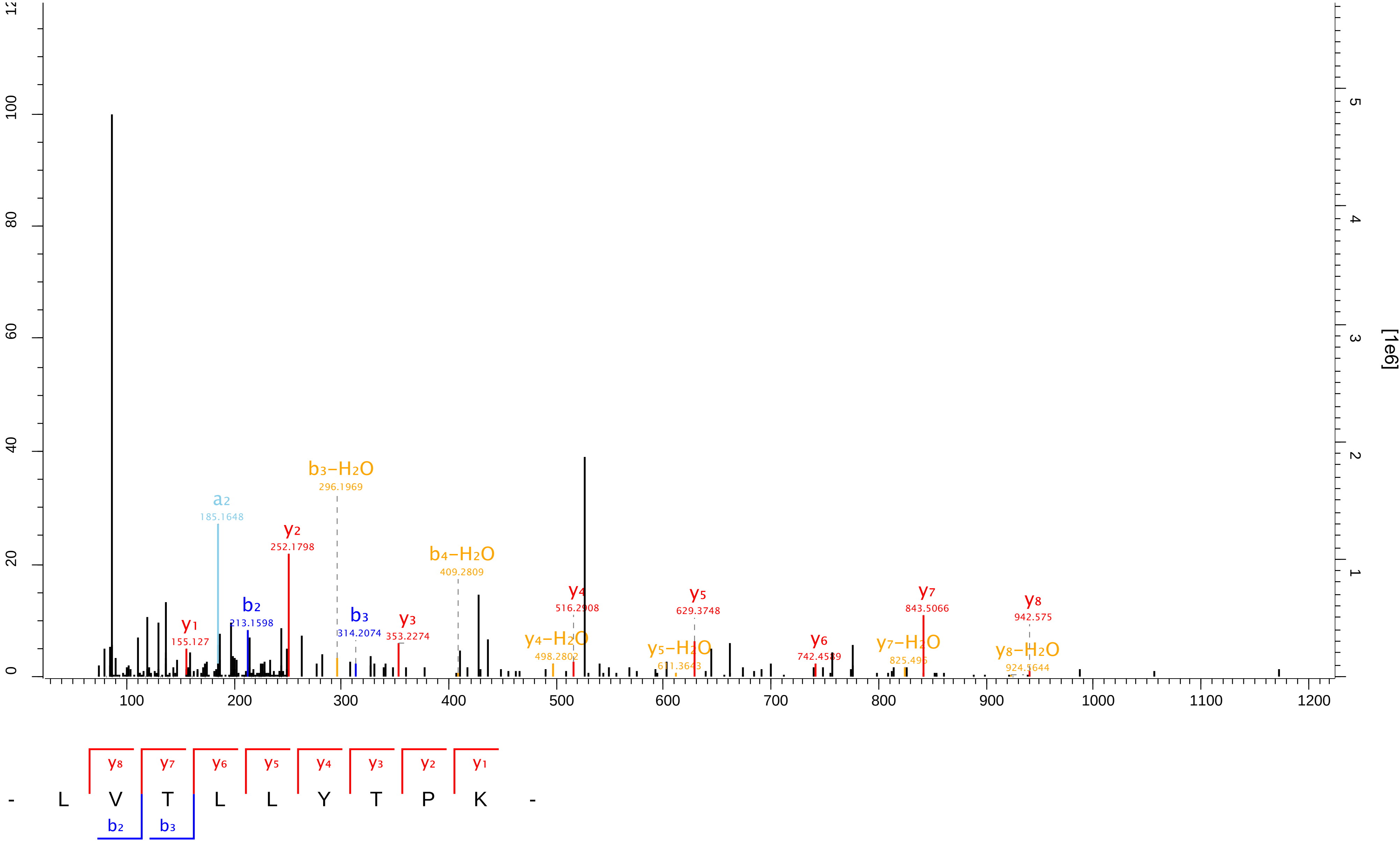

Raw file	Scan	Method	Score	m/z
QEplus003080	10799	FTMS; HCD	92.94	616.33

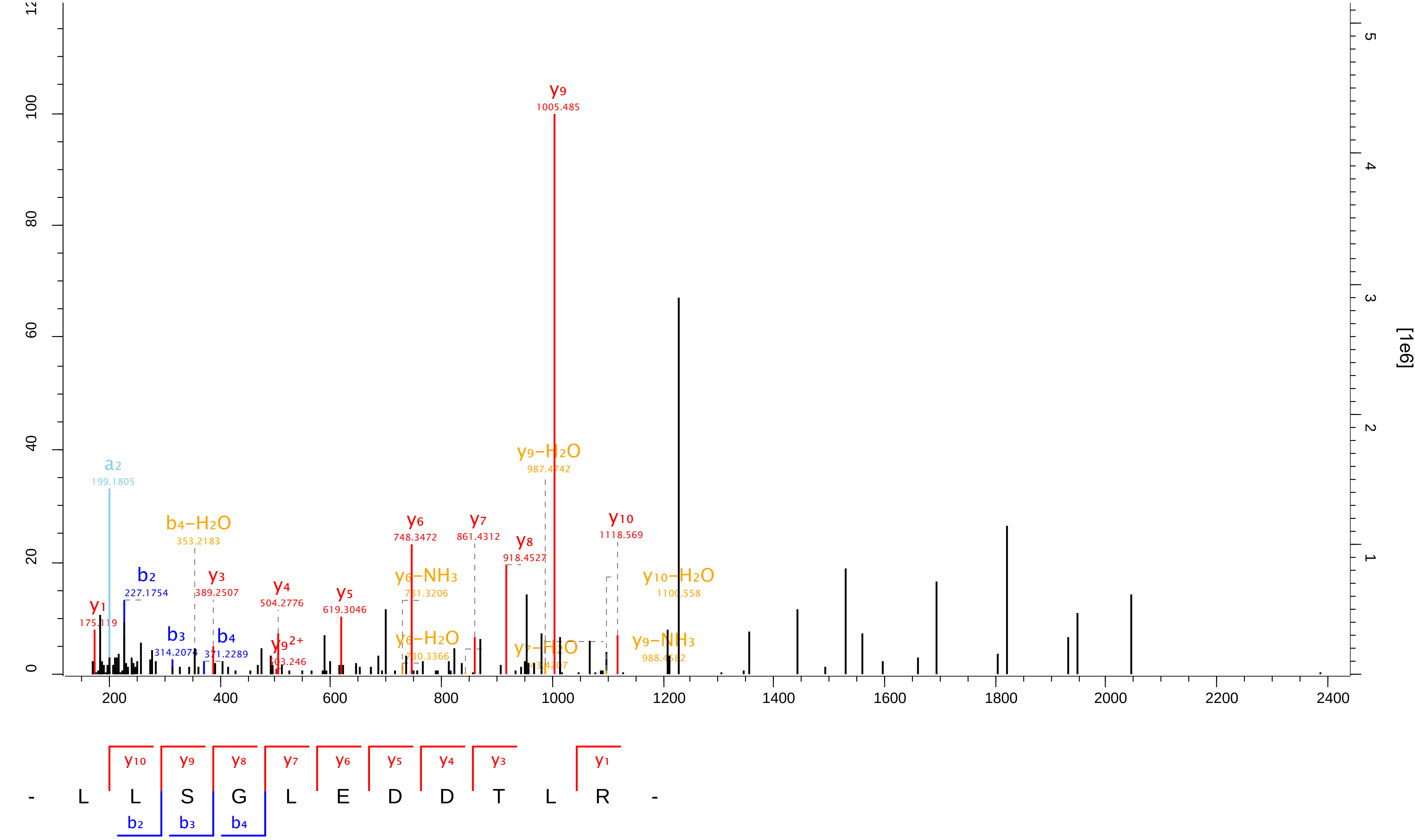

Raw file	Scan	Method	Score	m/z
QEplus003080	16289	FTMS; HCD	158.73	1009.87

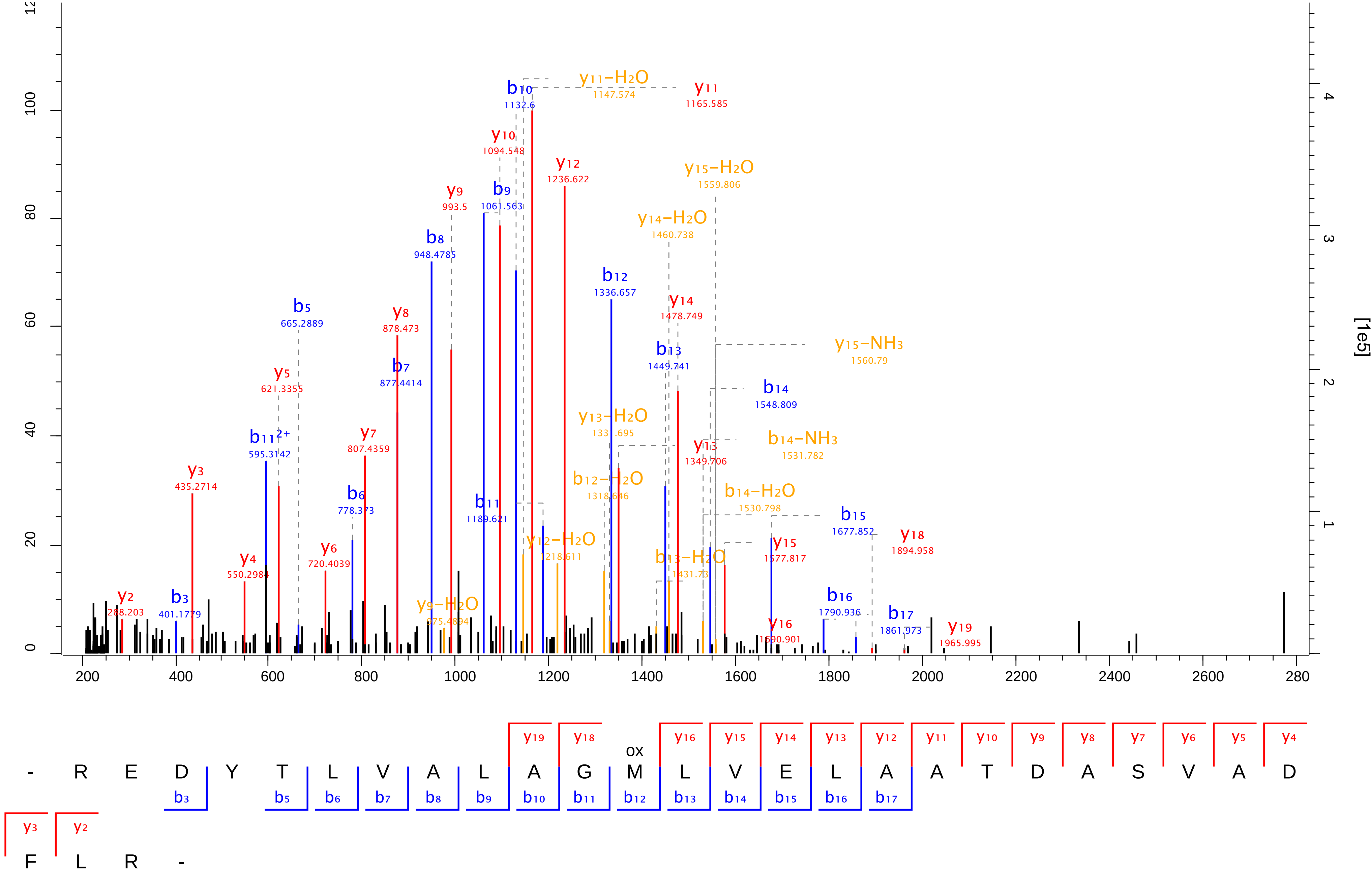

Raw file	Scan	Method	Score	m/z
QEplus003080	5180	FTMS; HCD	106.35	720.01

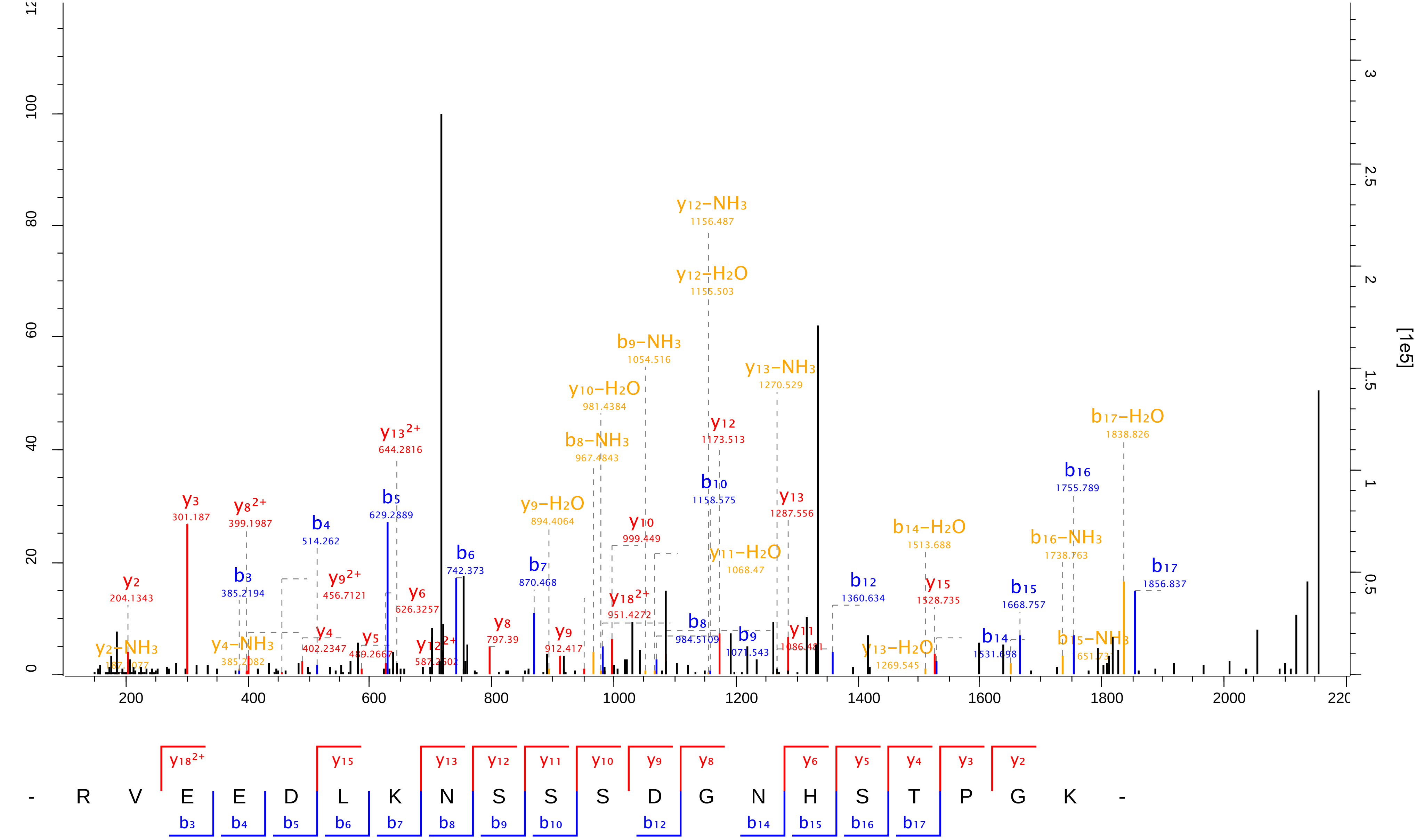

Raw file	Scan	Method	Score	m/z
QEplus003080	9871	FTMS; HCD	65.22	788.89

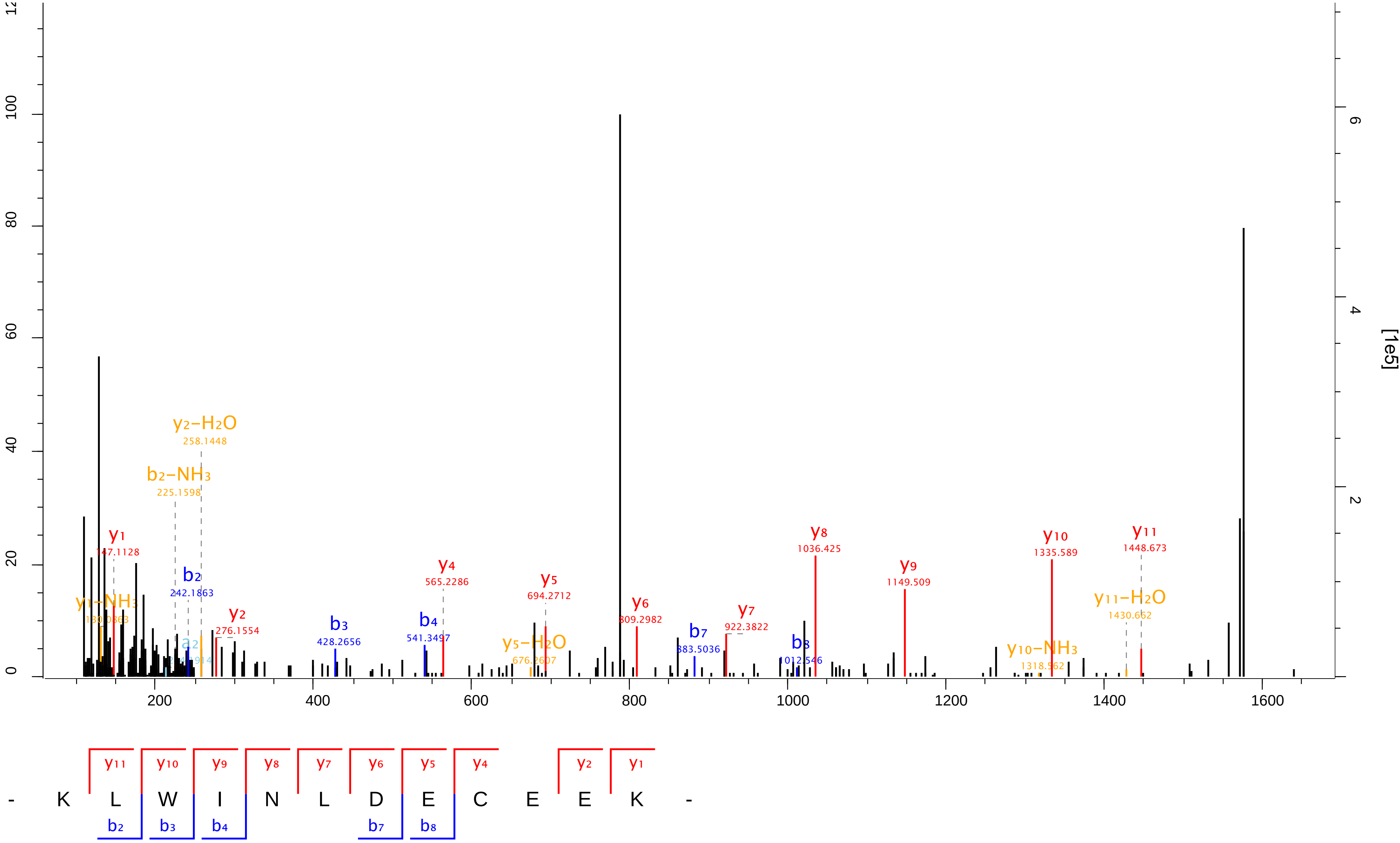

Raw file	Scan	Method	Score	m/z
QEplus003081	12349	FTMS; HCD	62.03	732.4

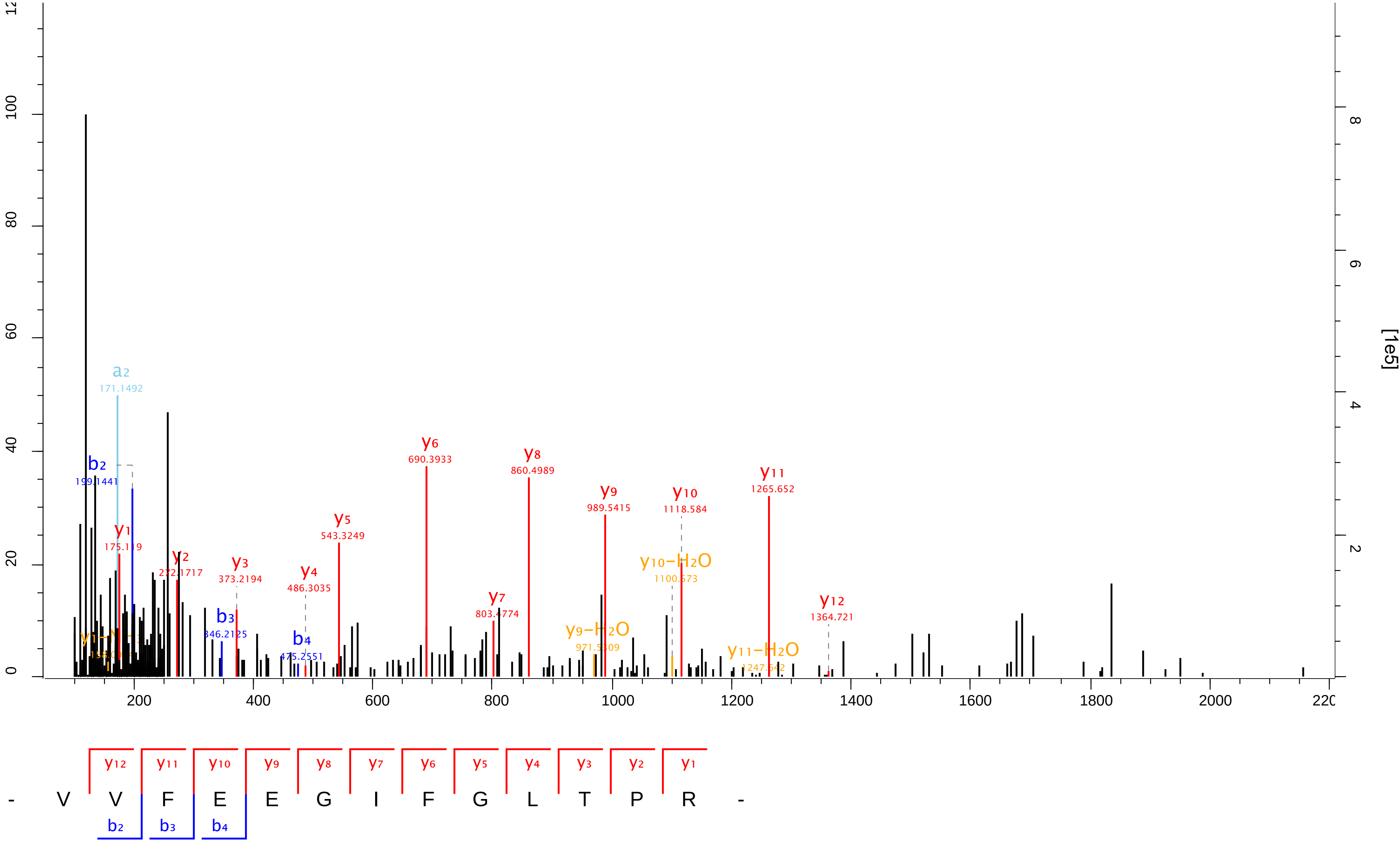

Raw file	Scan	Method	Score	m/z
QEplus003081	12786	FTMS; HCD	43.81	736.75

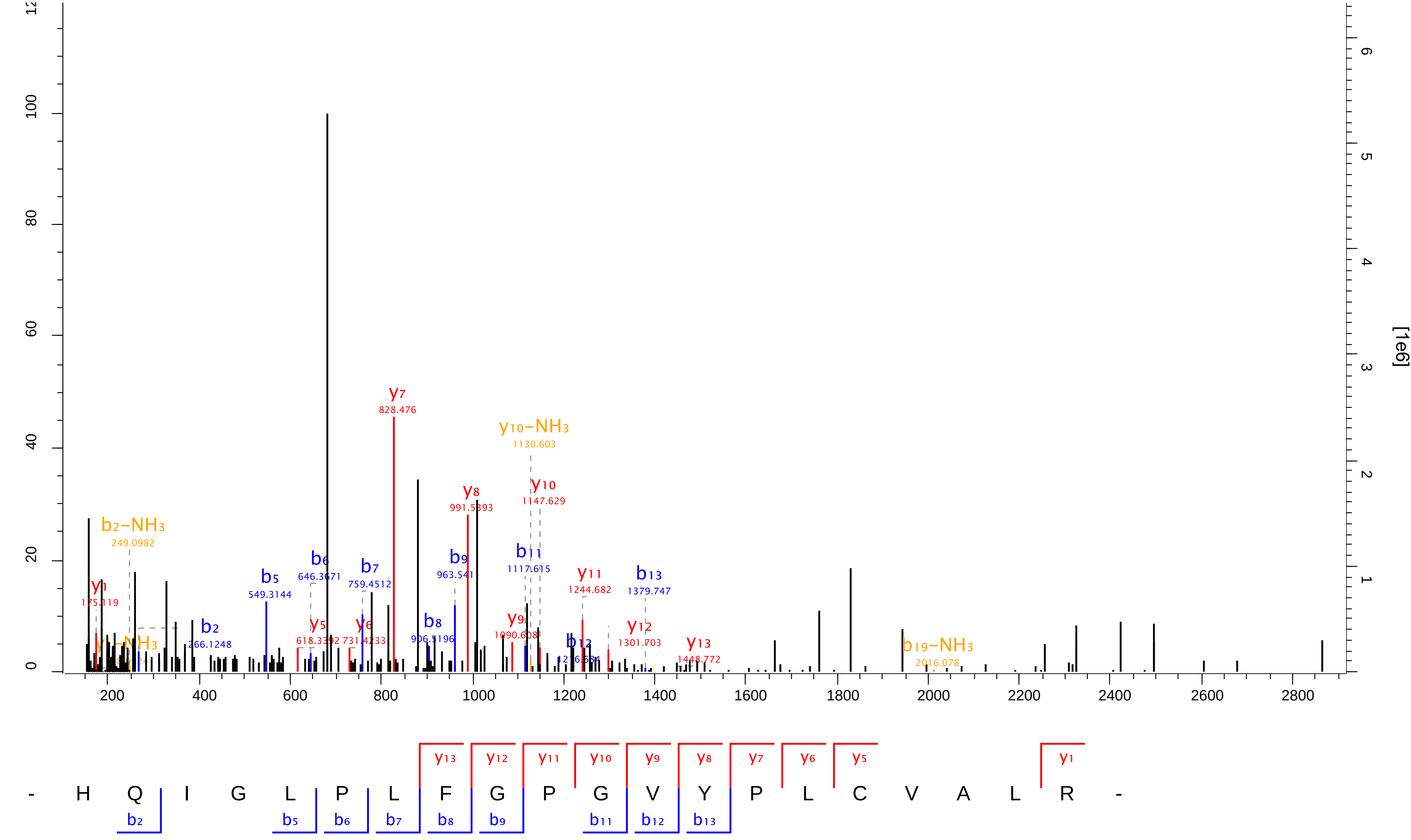

Raw file	Scan	Method	Score	m/z
QEplus003081	14049	FTMS; HCD	70.12	835.45

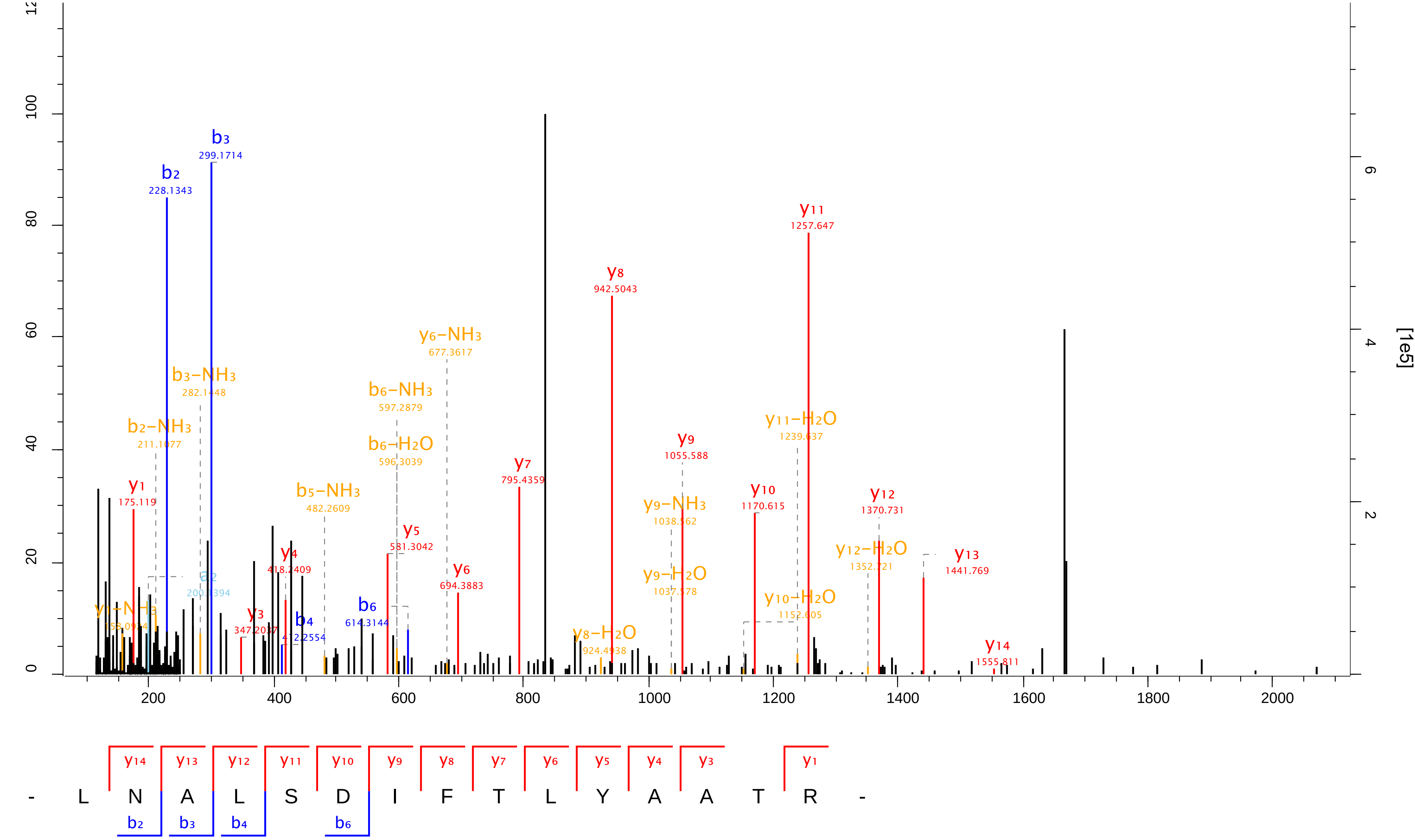

Raw file	Scan	Method	Score	m/z
QEplus003081	5098	FTMS; HCD	104.43	612.3

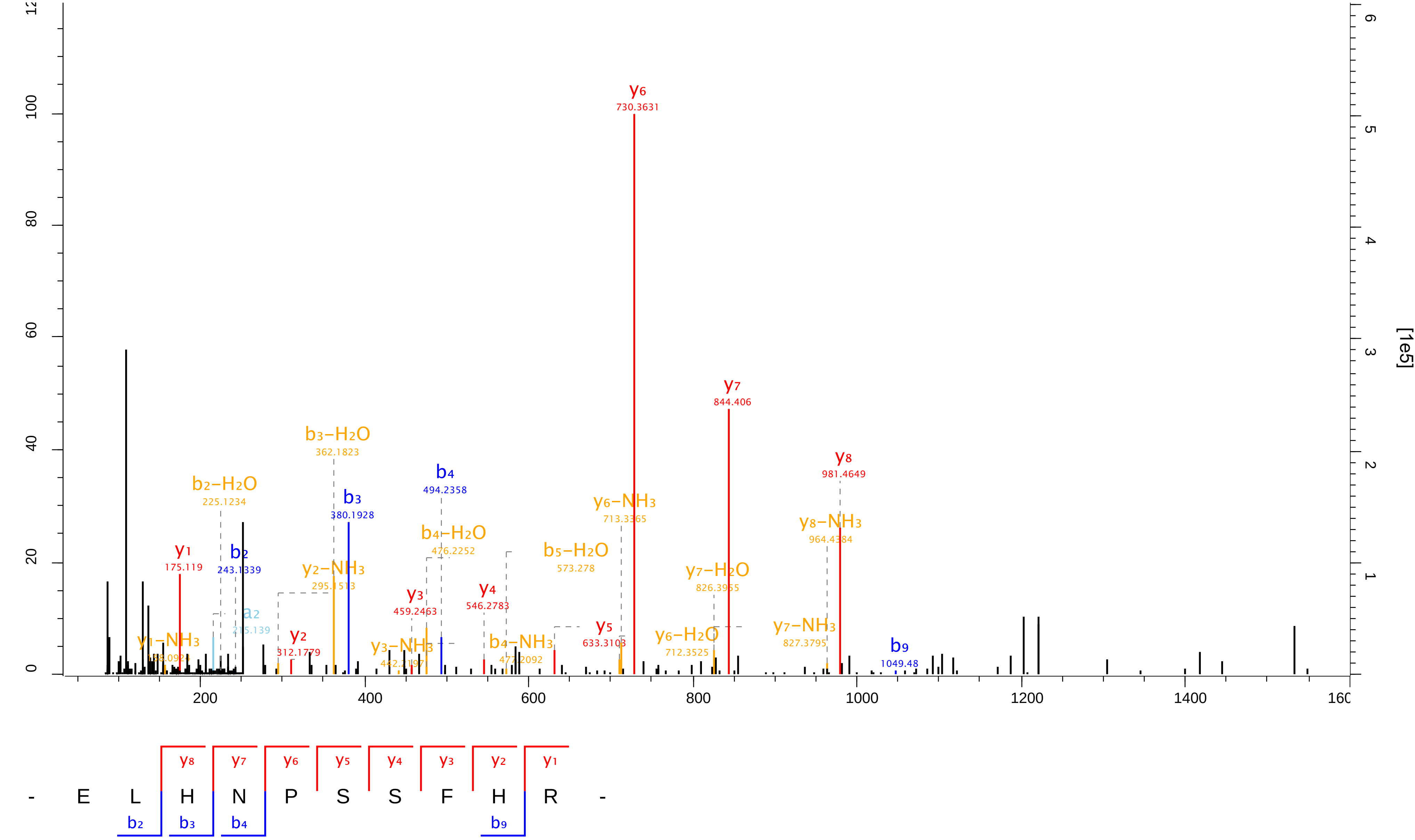

Raw file	Scan	Method	Score	m/z
QEplus003081	6636	FTMS; HCD	79.32	592.63

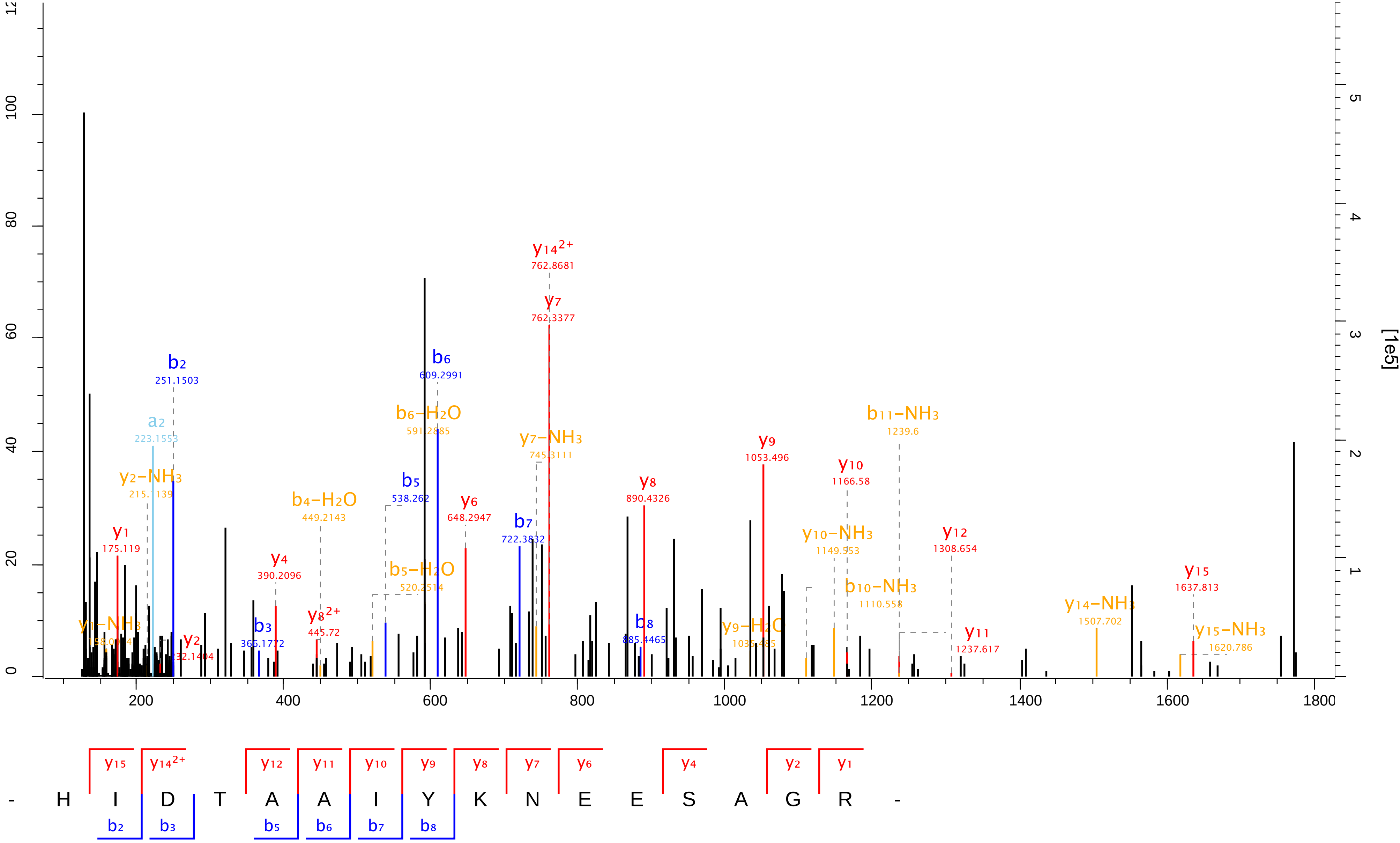

Raw file	Scan	Method	Score	m/z
QEplus003082	11879	FTMS; HCD	86.54	732.72

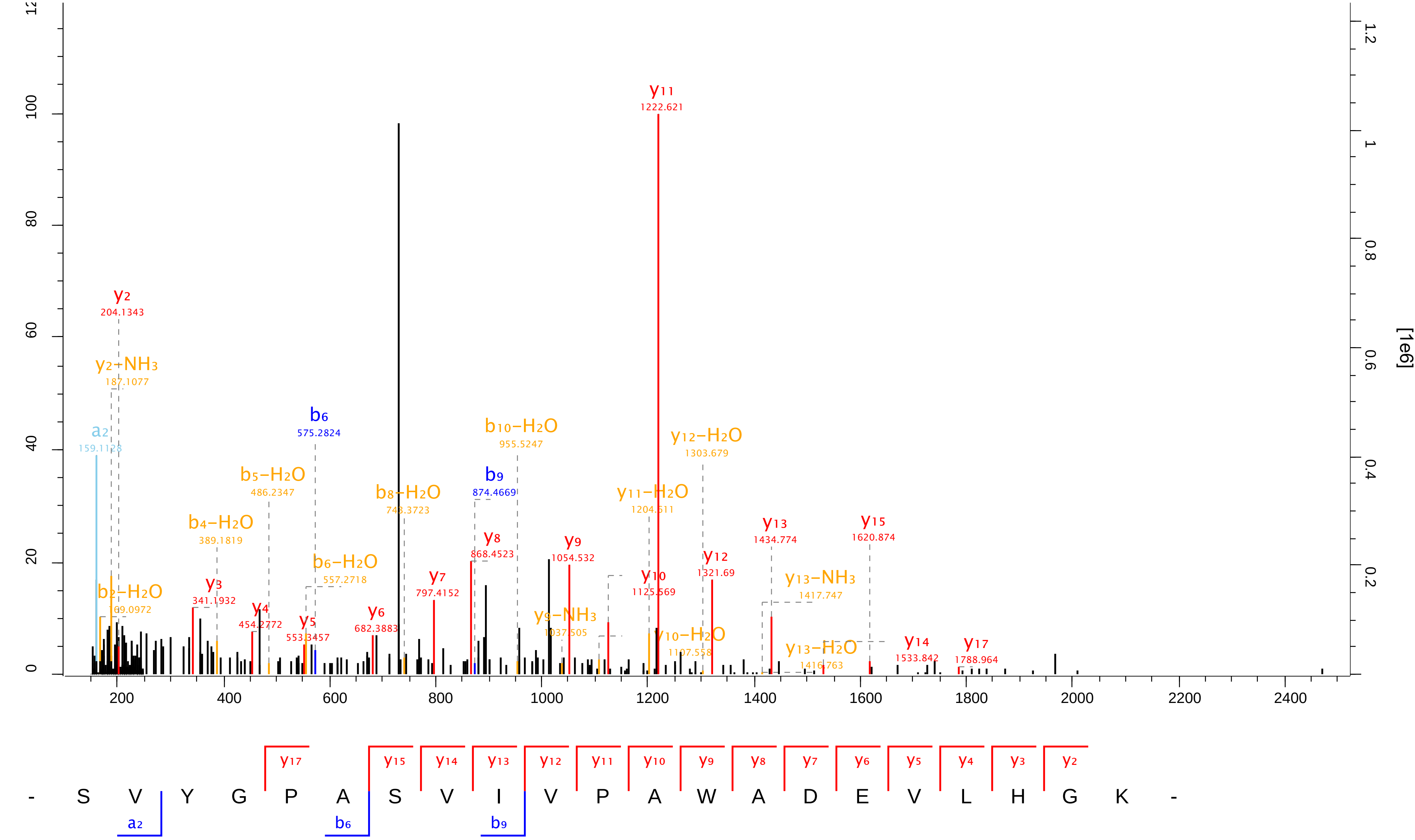

Raw file	Scan	Method	Score	m/z
QEplus003082	12708	FTMS; HCD	80.63	839.96

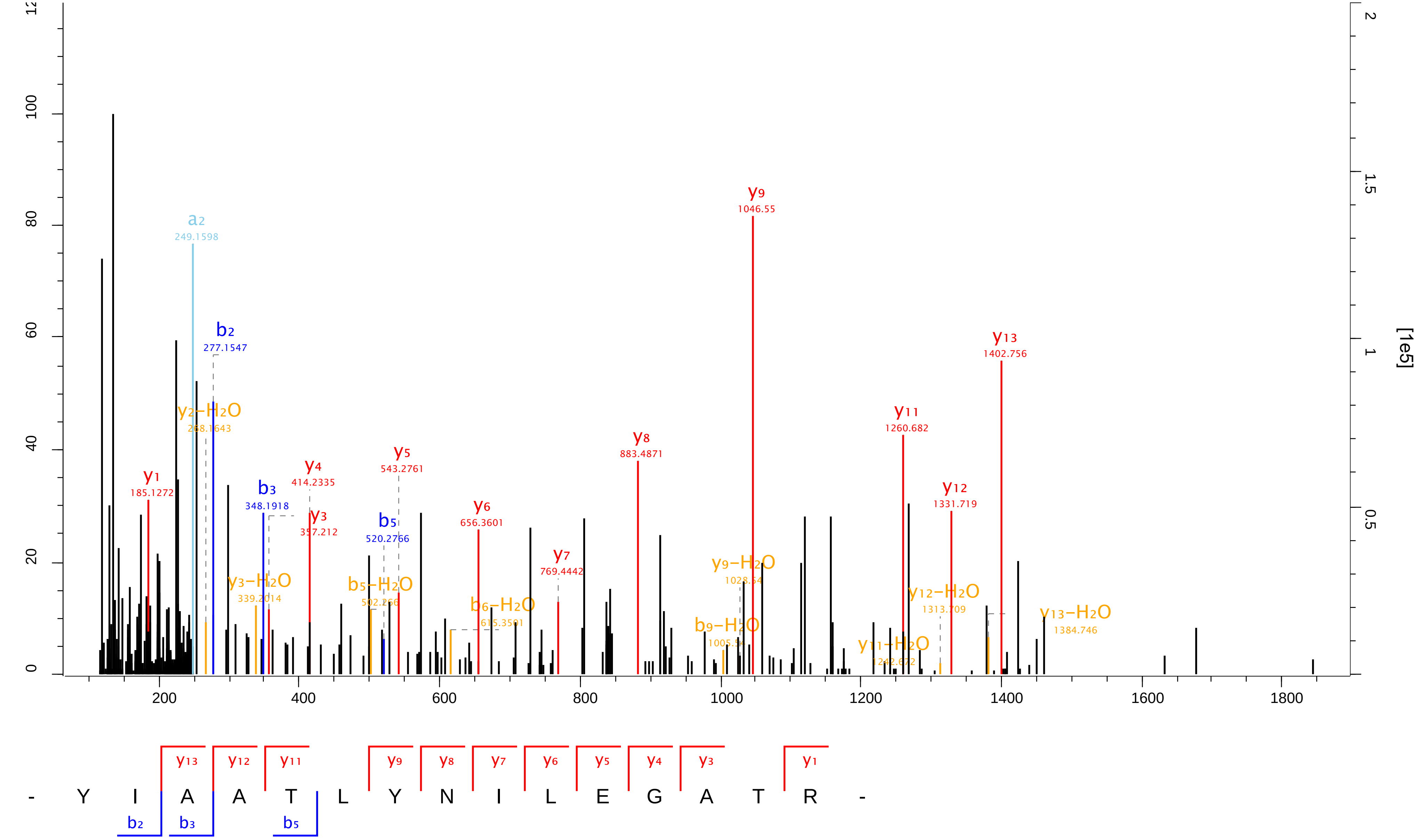

Raw file	Scan	Method	Score	m/z
QEplus003082	2950	FTMS; HCD	62.34	425.9

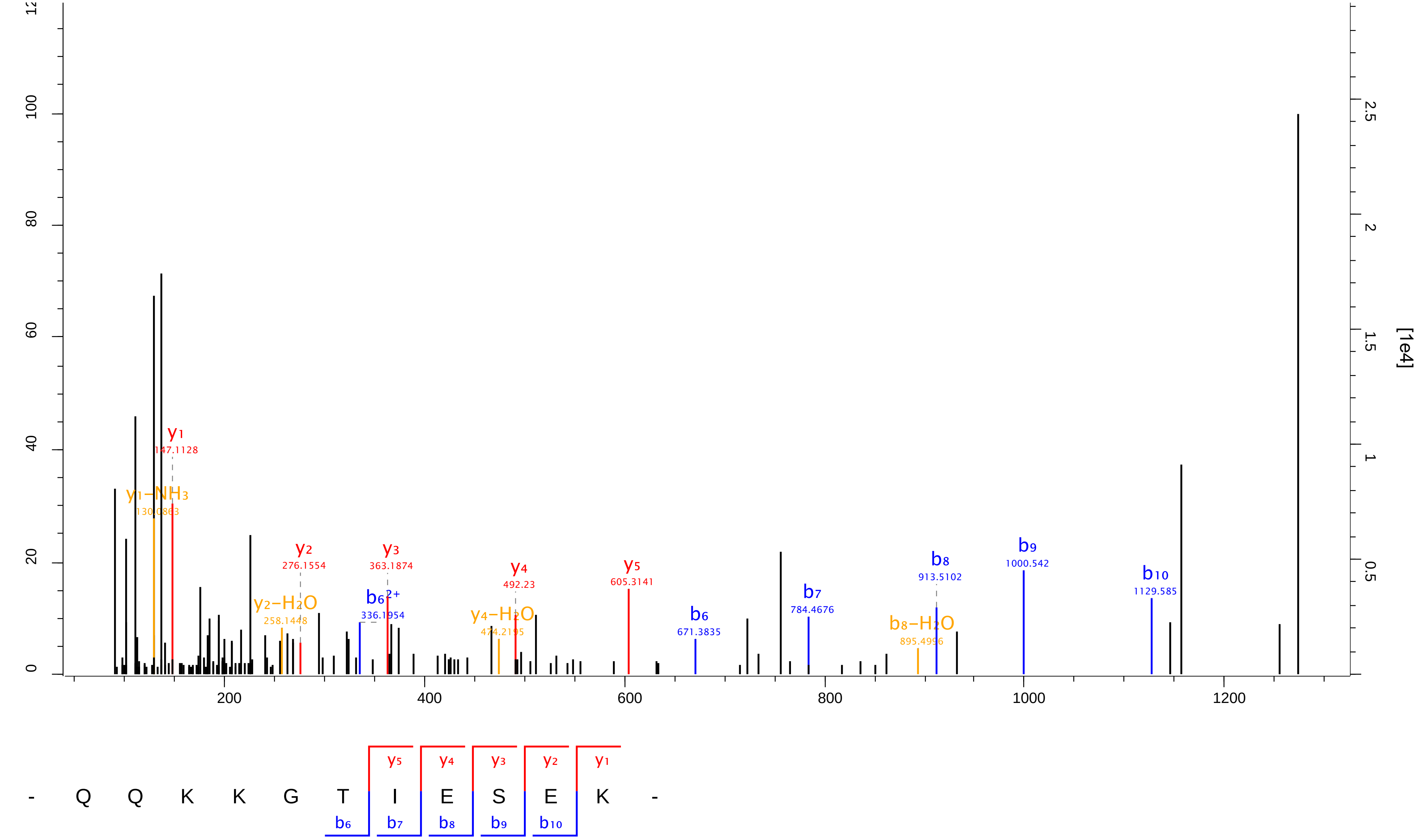

Raw file	Scan	Method	Score	m/z
QEplus003082	3029	FTMS; HCD	50.09	704.81

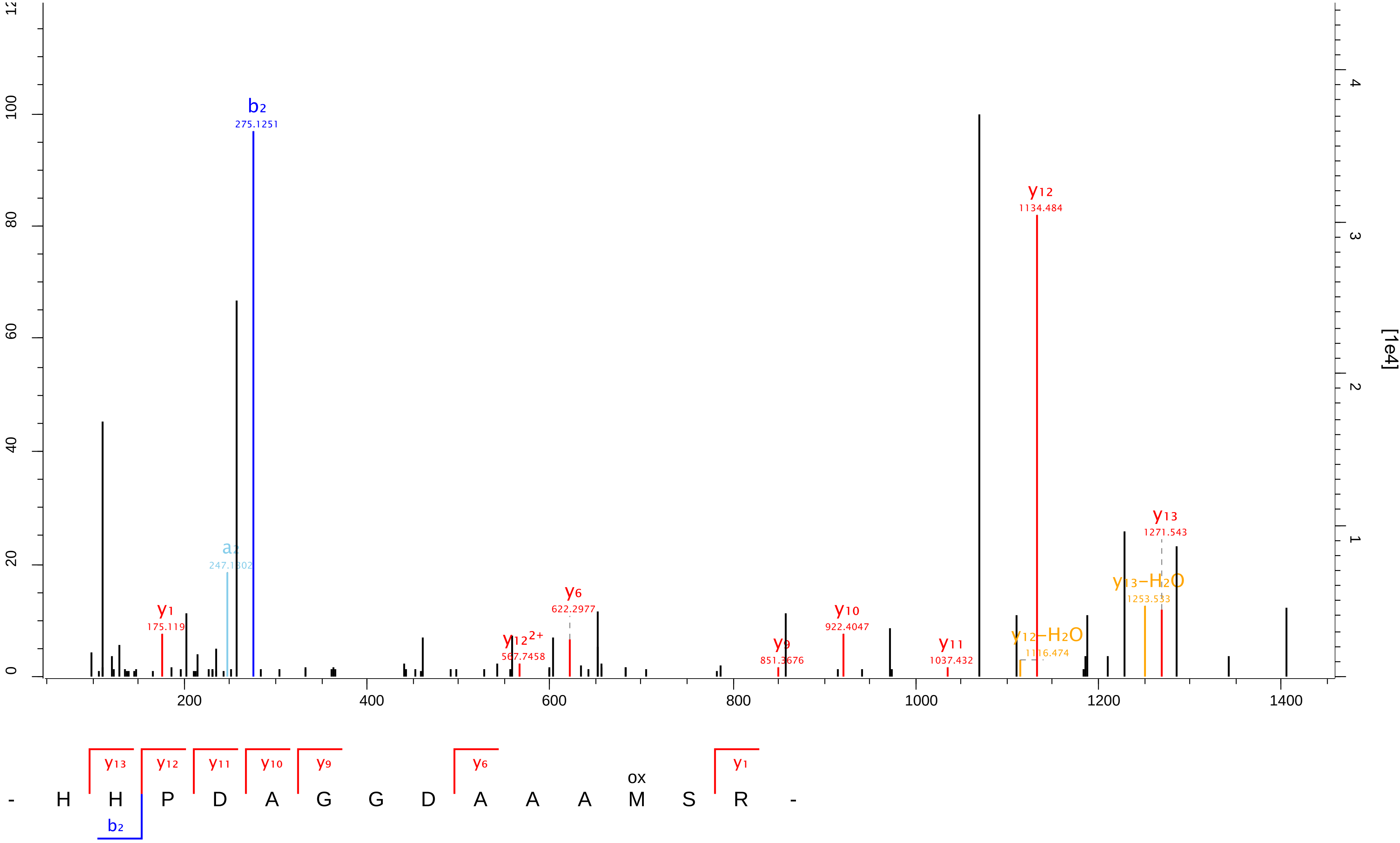

Raw file	Scan	Method	Score	m/z
QEplus003082	3899	FTMS; HCD	69.92	739.86

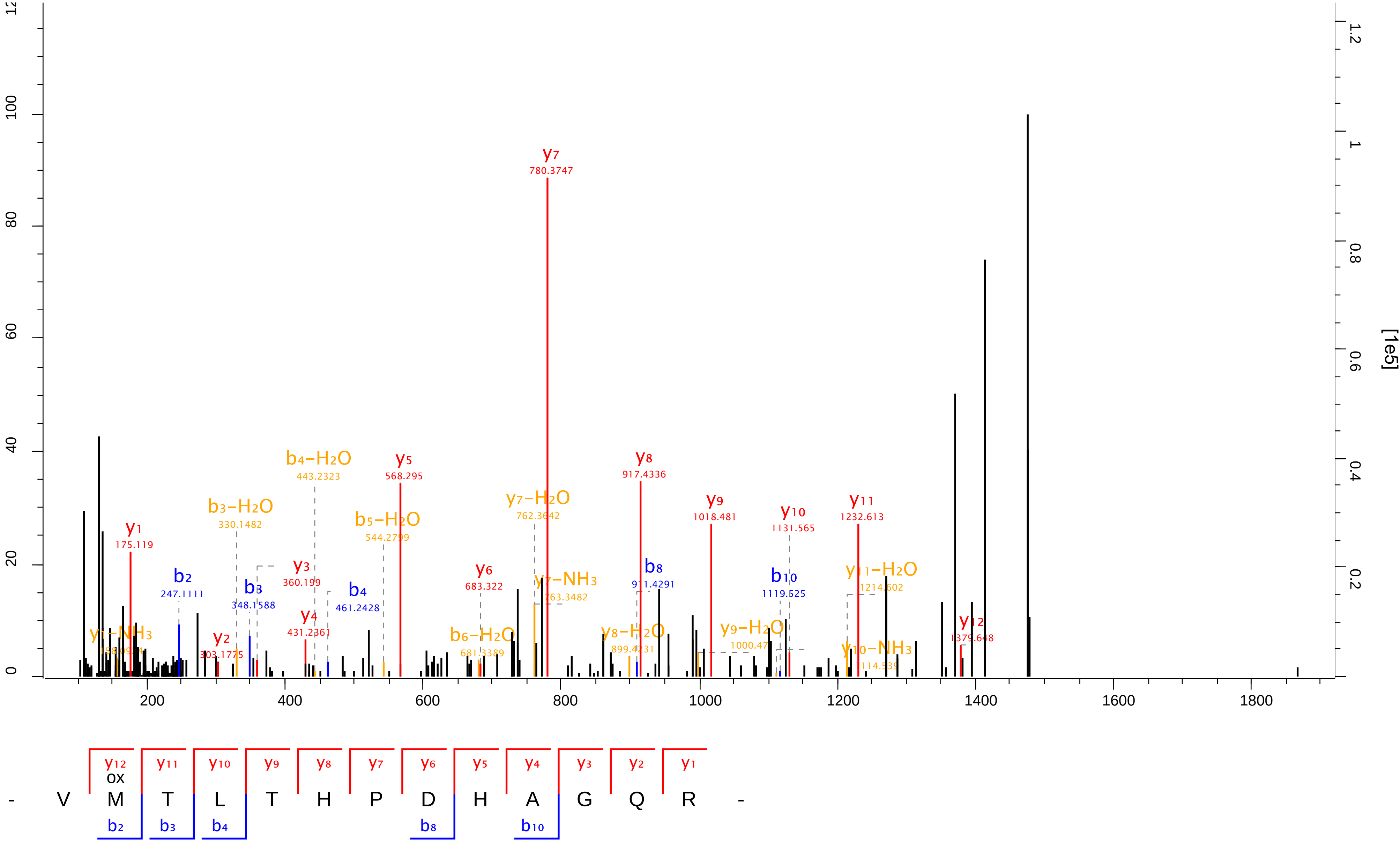

Raw file	Scan	Method	Score	m/z
QEplus003082	5184	FTMS; HCD	128.41	638.81

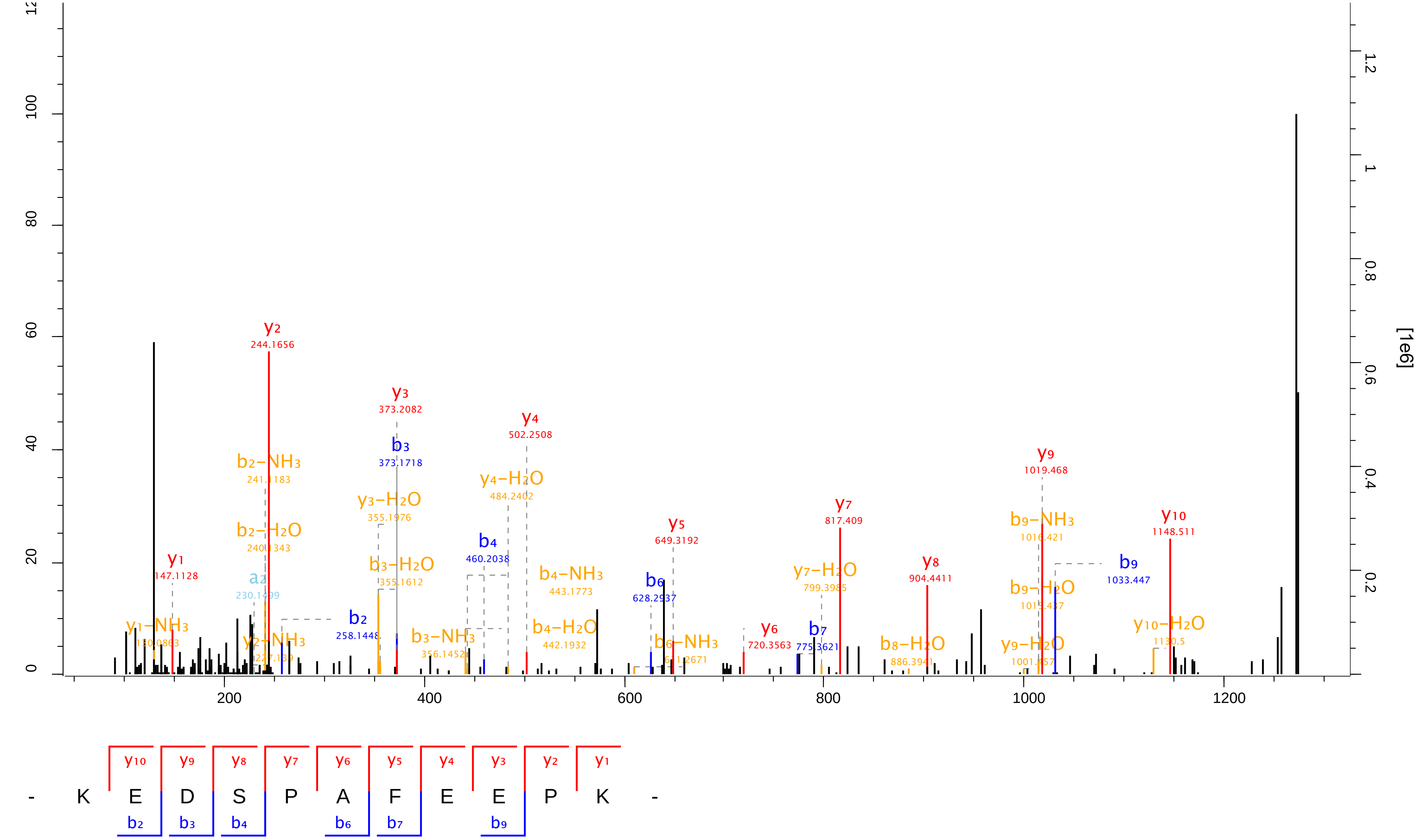

Raw file	Scan	Method	Score	m/z
QEplus003082	9534	FTMS; HCD	69.65	649.86

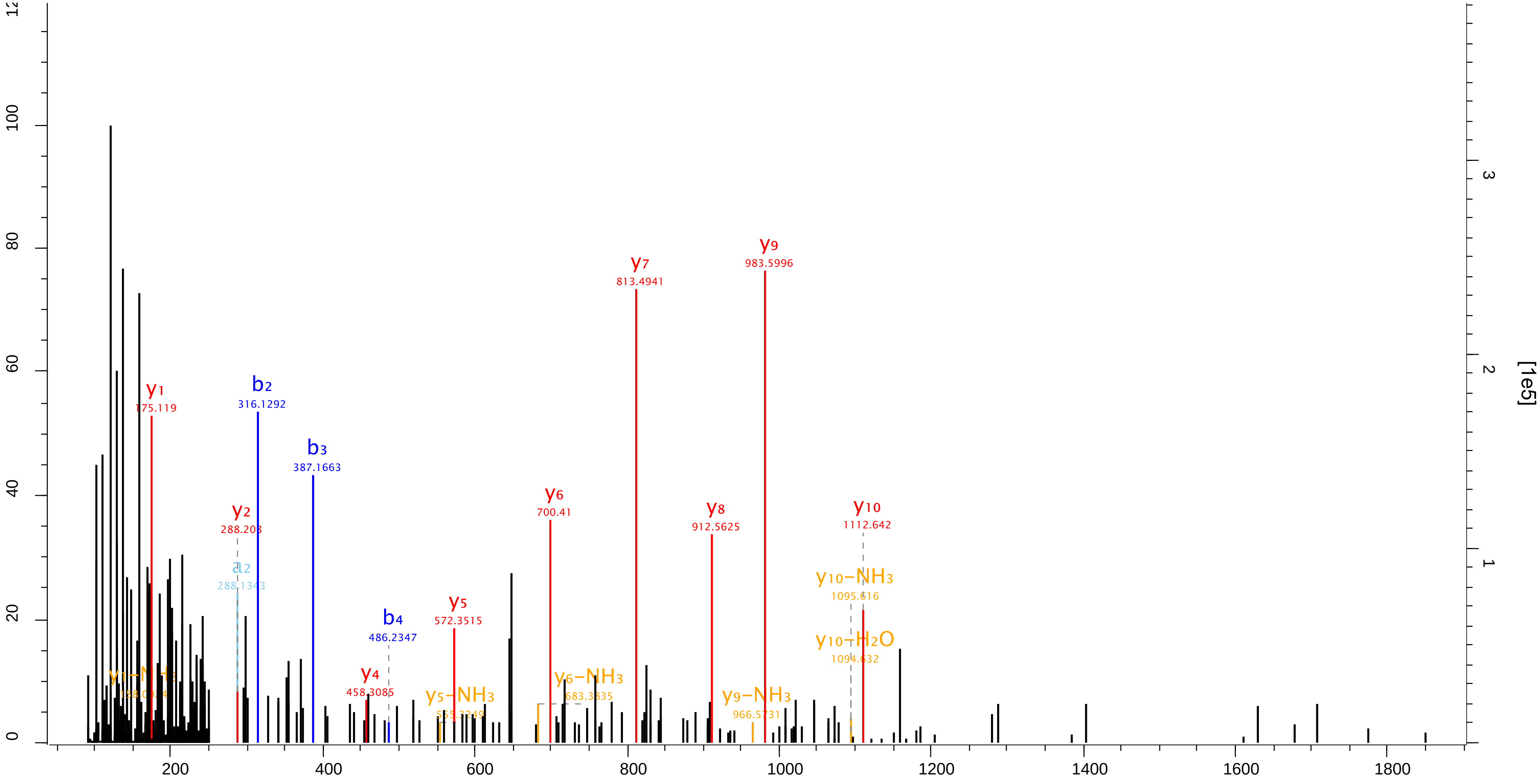

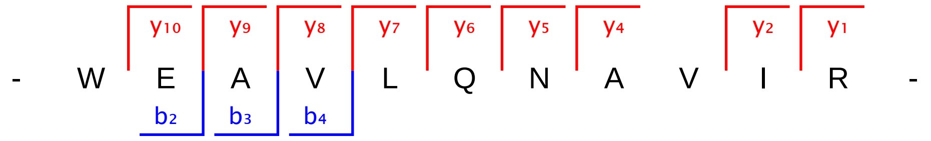

Raw file	Scan	Method	Score	m/z
QEplus003083	10005	FTMS; HCD	50.35	571.85

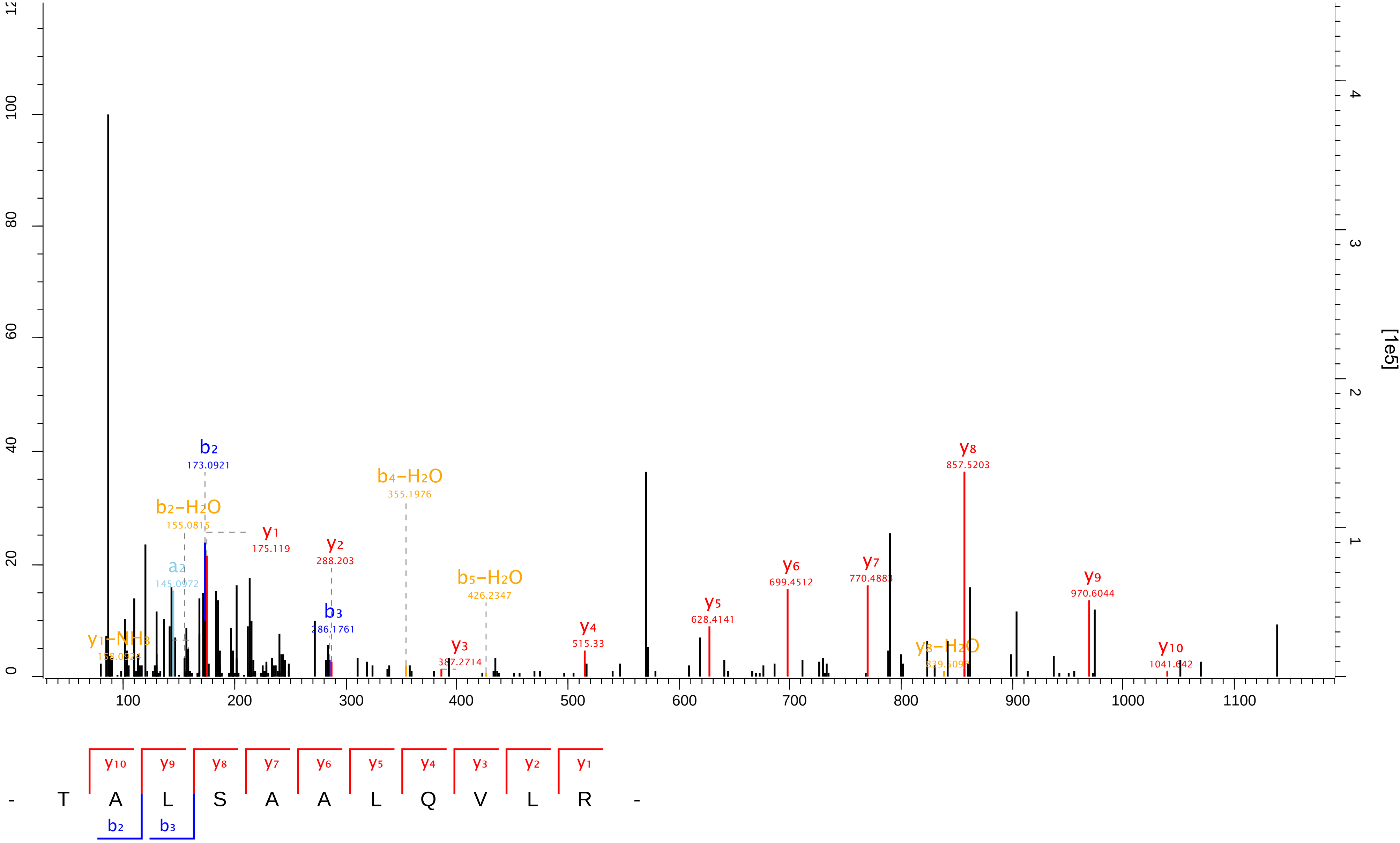

Raw file	Scan	Method	Score	m/z
QEplus003083	10195	FTMS; HCD	99.75	726.92

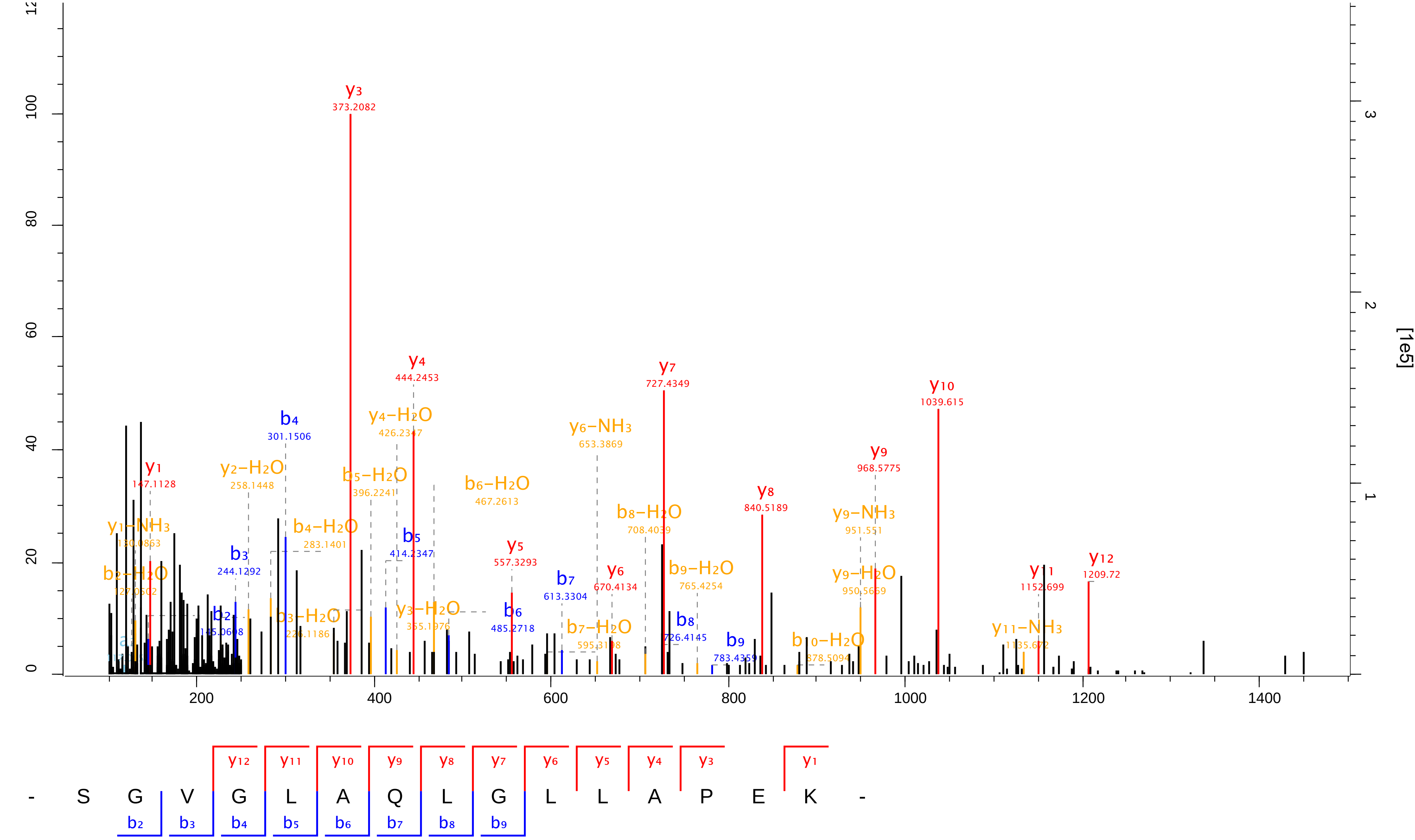

Raw file	Scan	Method	Score	m/z
QEplus003083	10656	FTMS; HCD	87.52	624.98

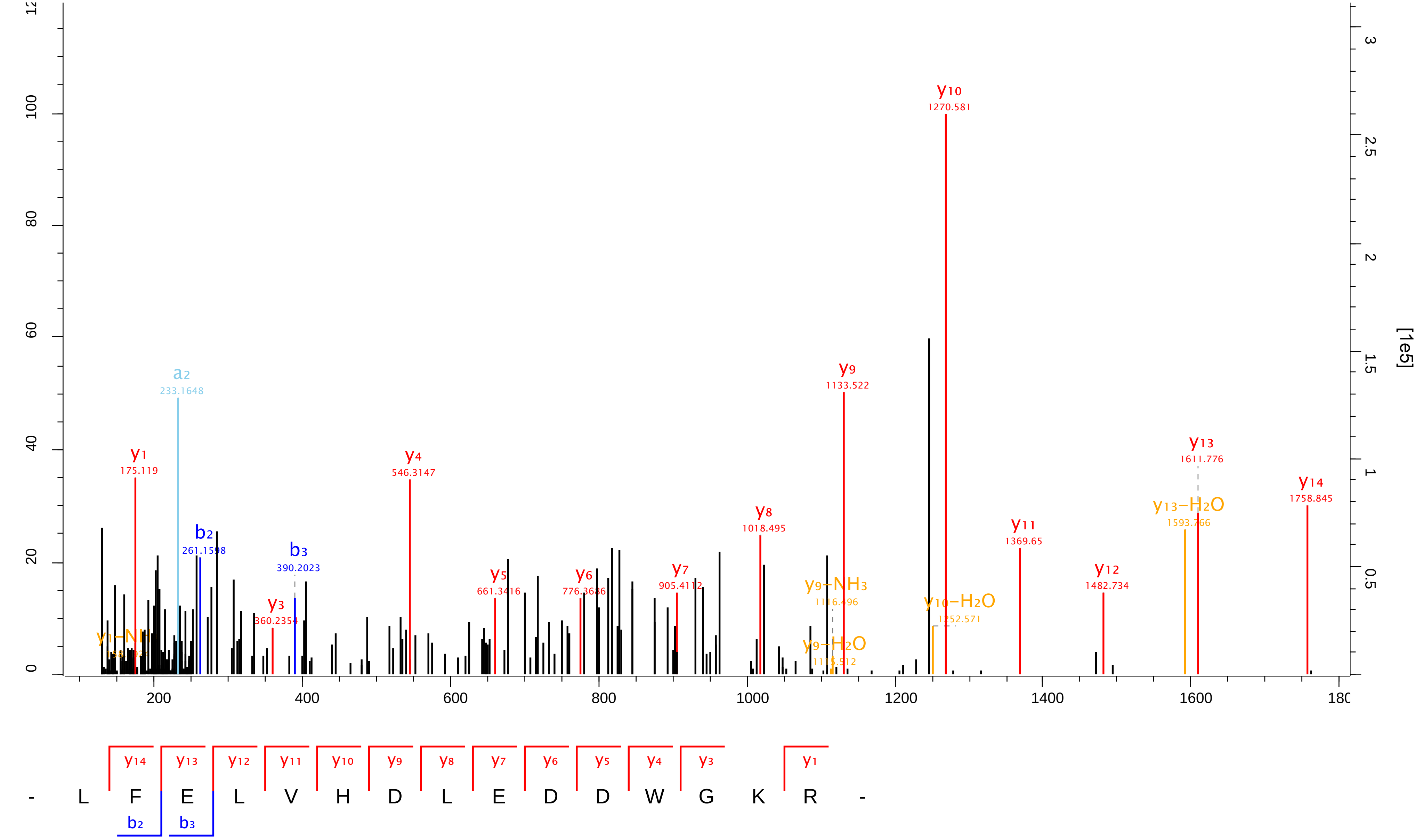

Raw file	Scan	Method	Score	m/z
QEplus003083	13089	FTMS; HCD	68.48	817.51

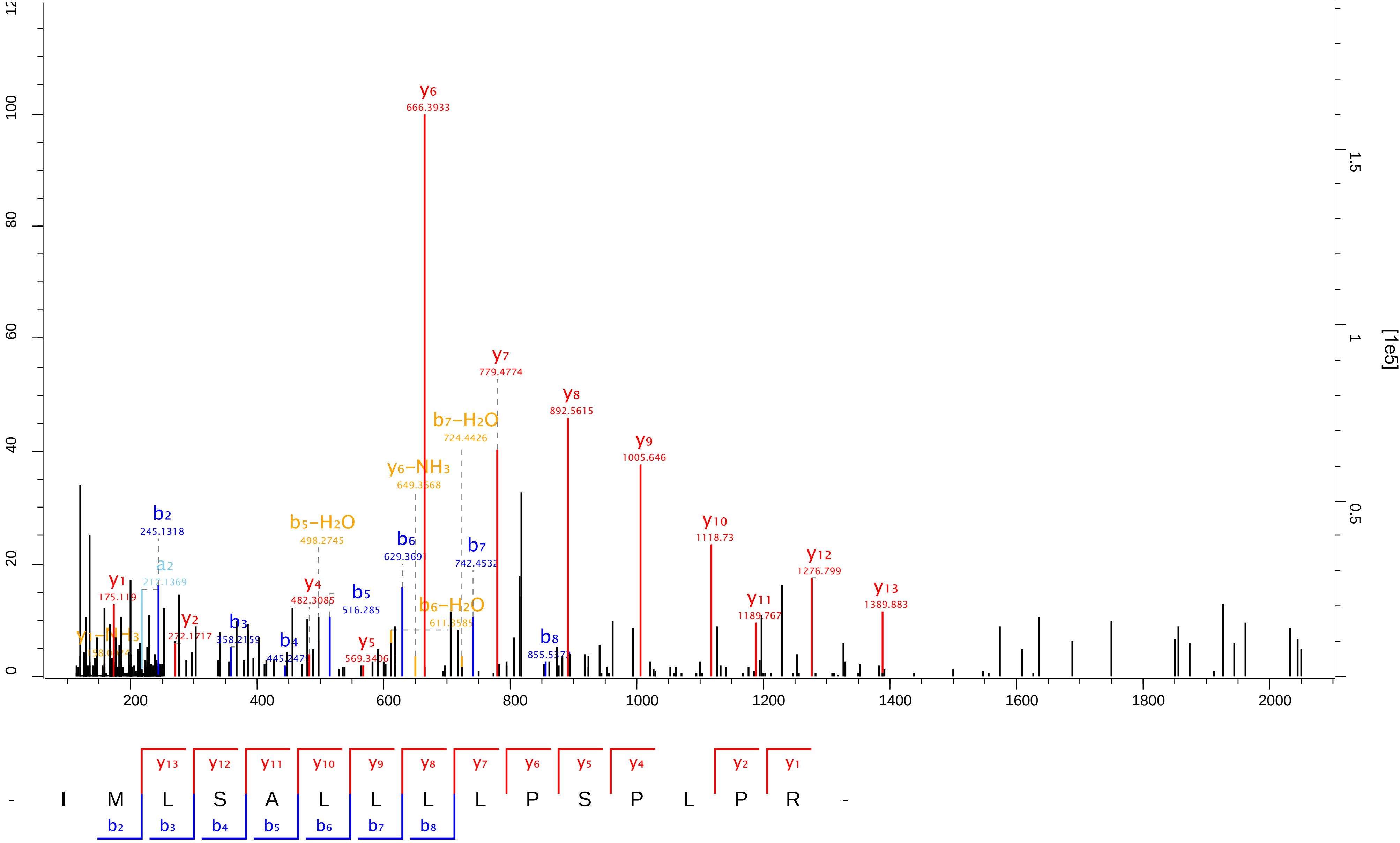

Raw file	Scan	Method	Score	m/z
QEplus003083	14063	FTMS; HCD	139.11	1012.04

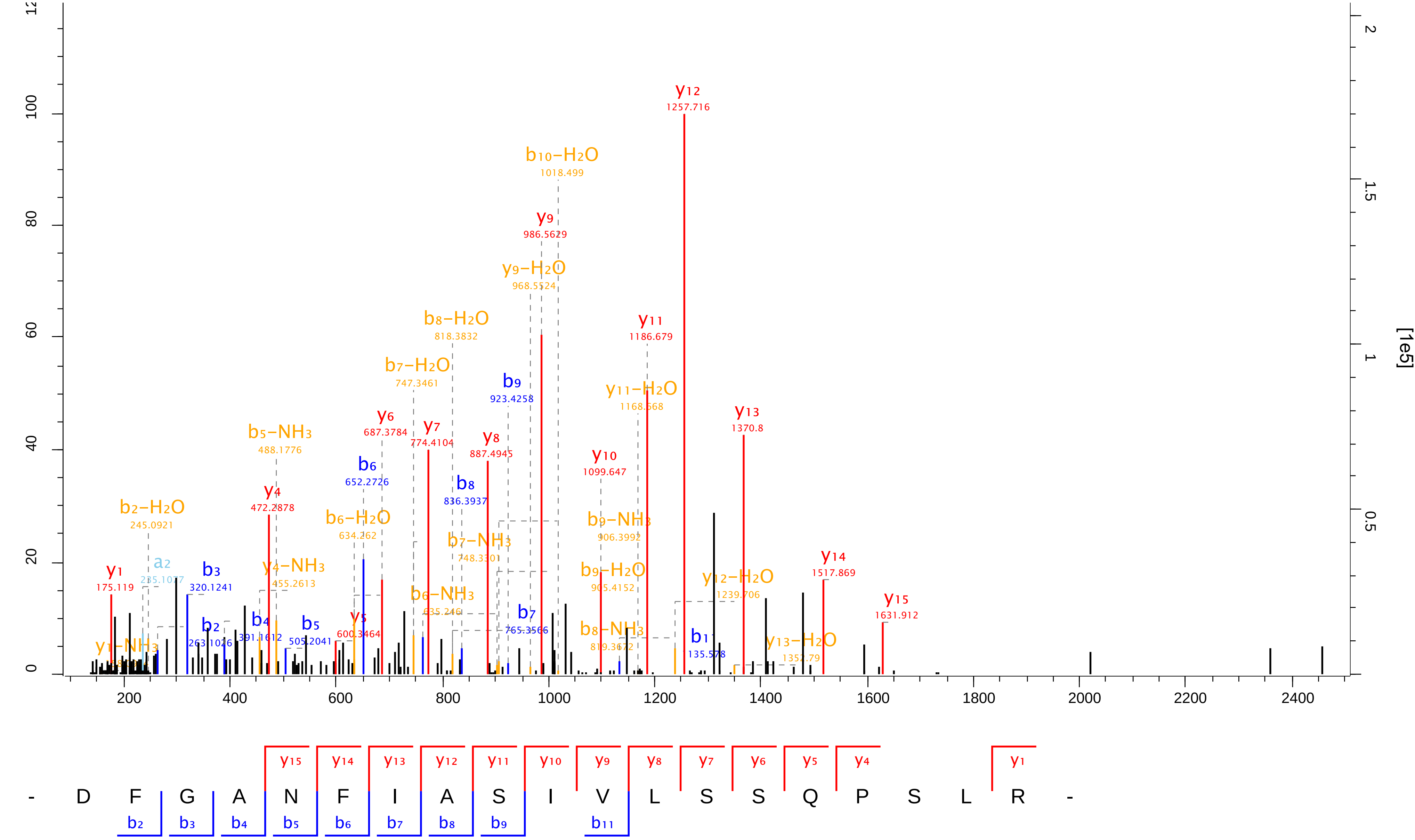

Raw file	Scan	Method	Score	m/z
QEplus003083	4677	FTMS; HCD	69.22	623.79

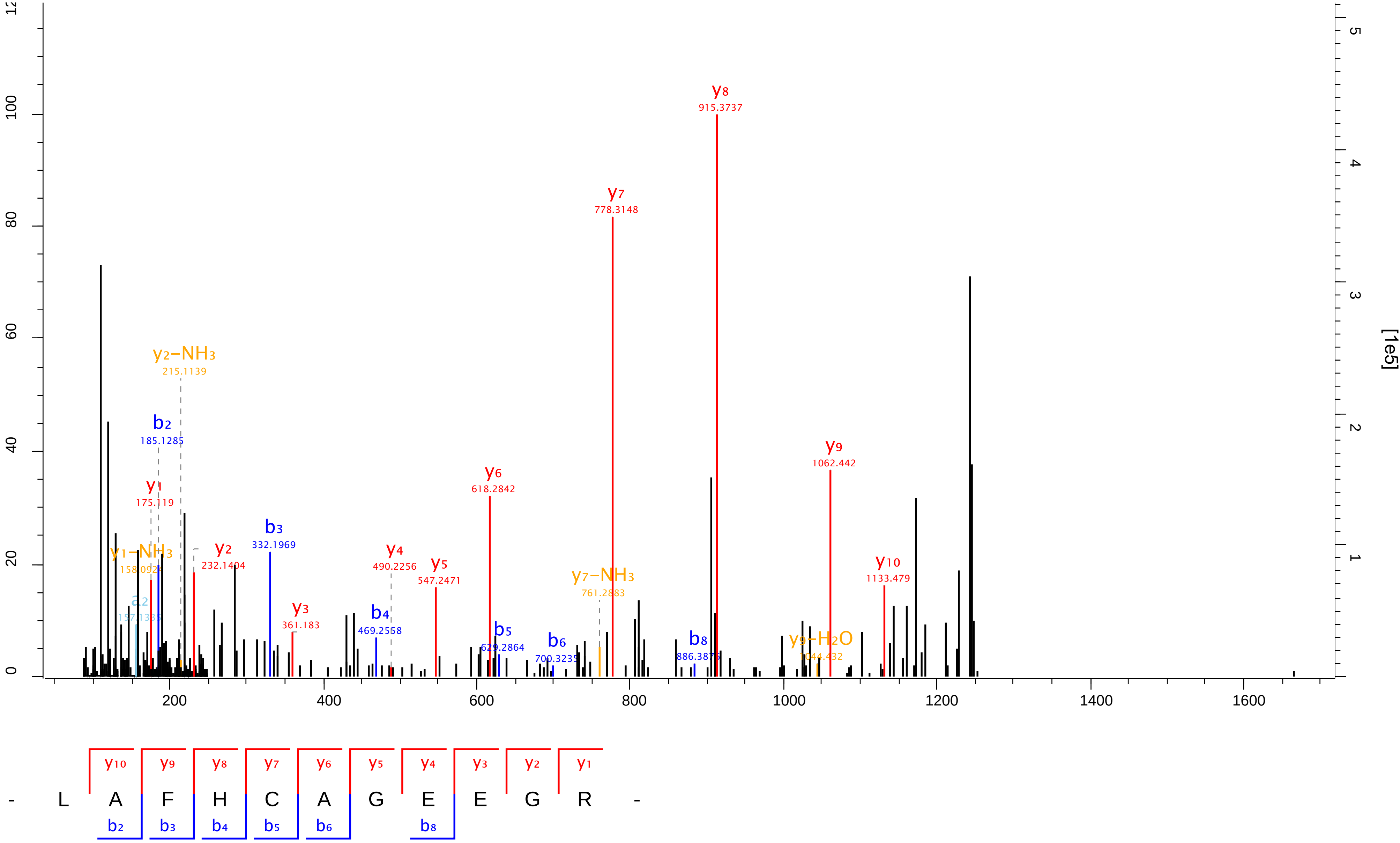

Raw file	Scan	Method	Score	m/z
QEplus003083	7350	FTMS; HCD	83.75	649.03

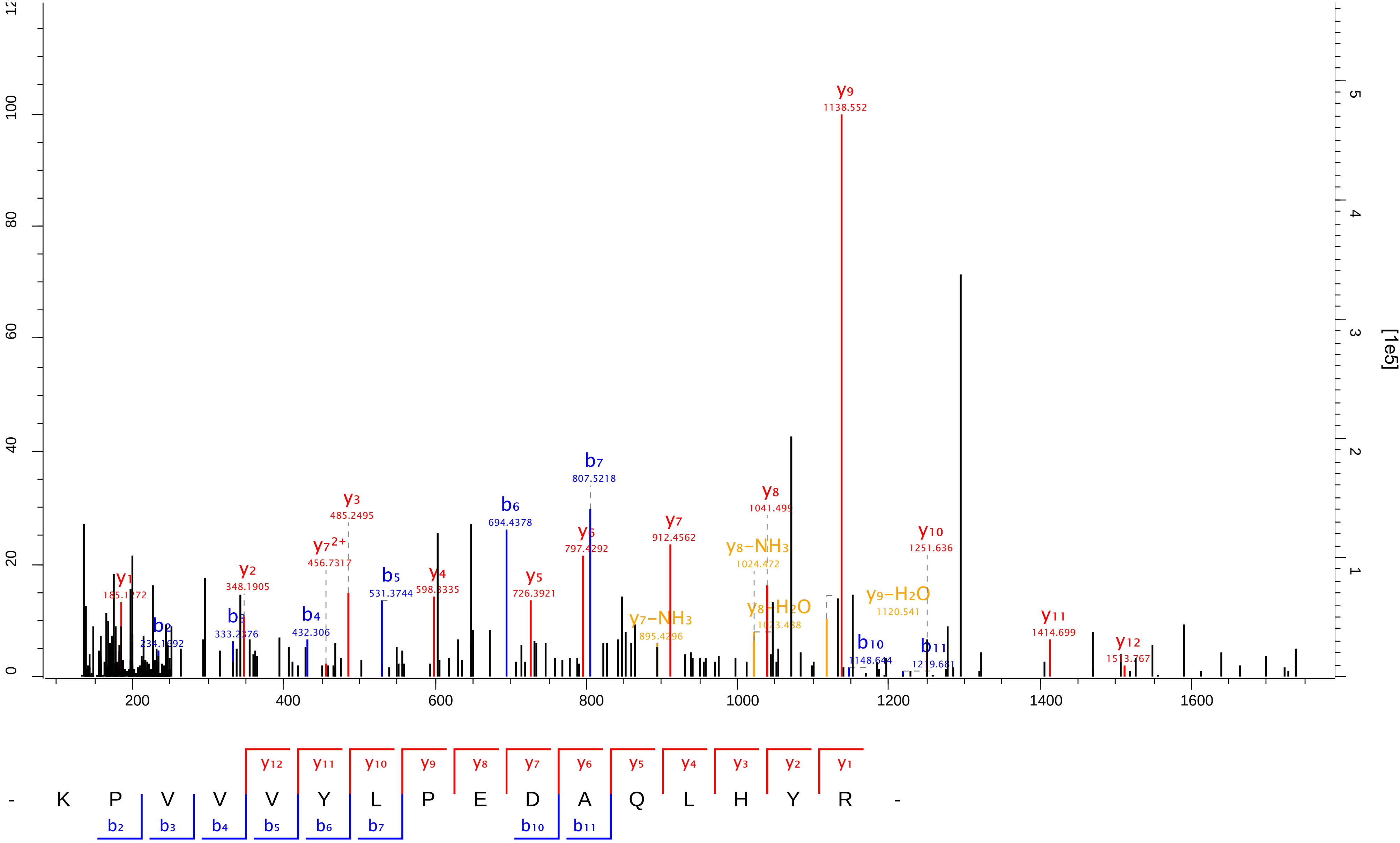

Raw file	Scan	Method	Score	m/z
QEplus003083	7605	FTMS; HCD	91.87	532.28

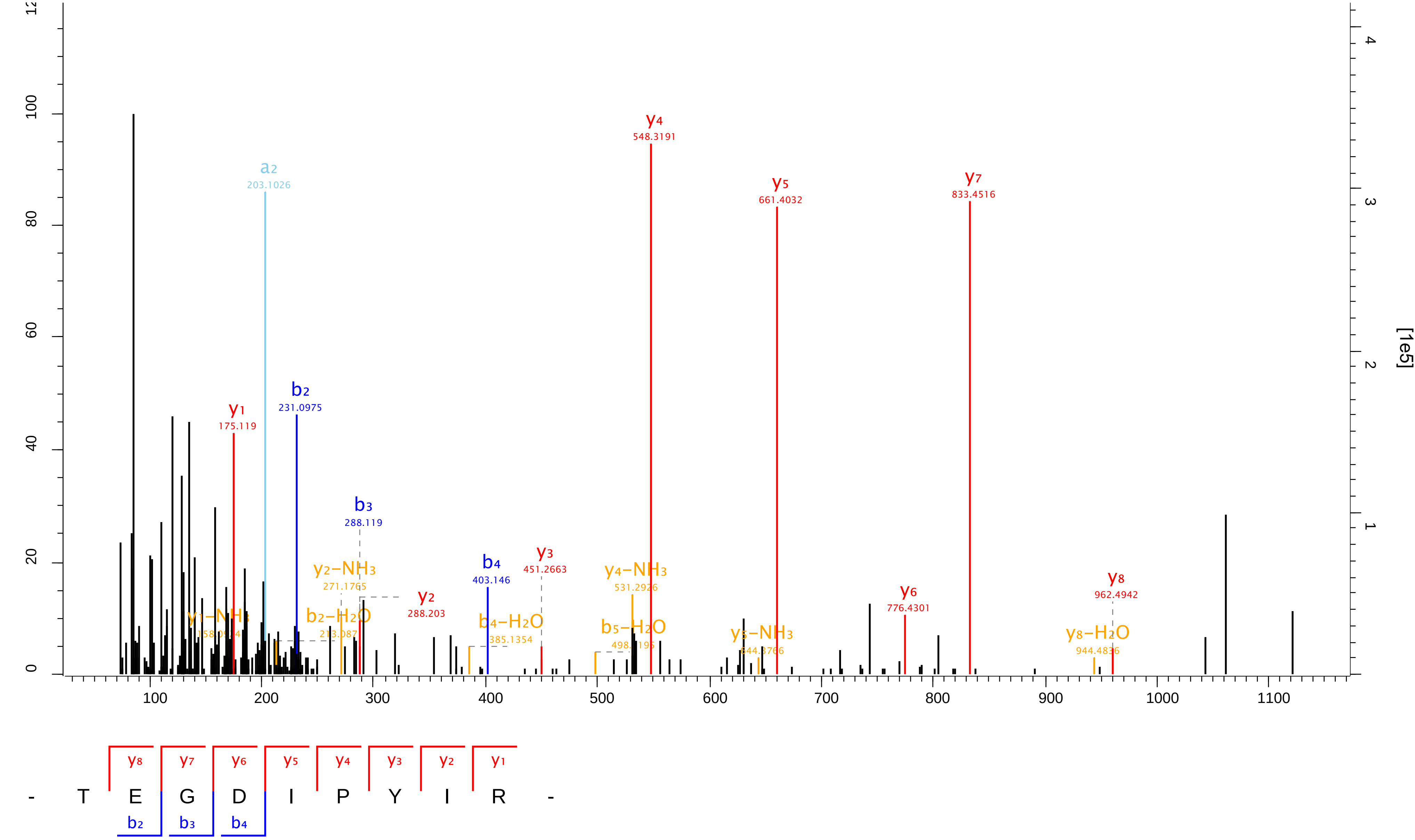

Raw file	Scan	Method	Score	m/z
QEplus003083	9238	FTMS; HCD	56.04	705.68

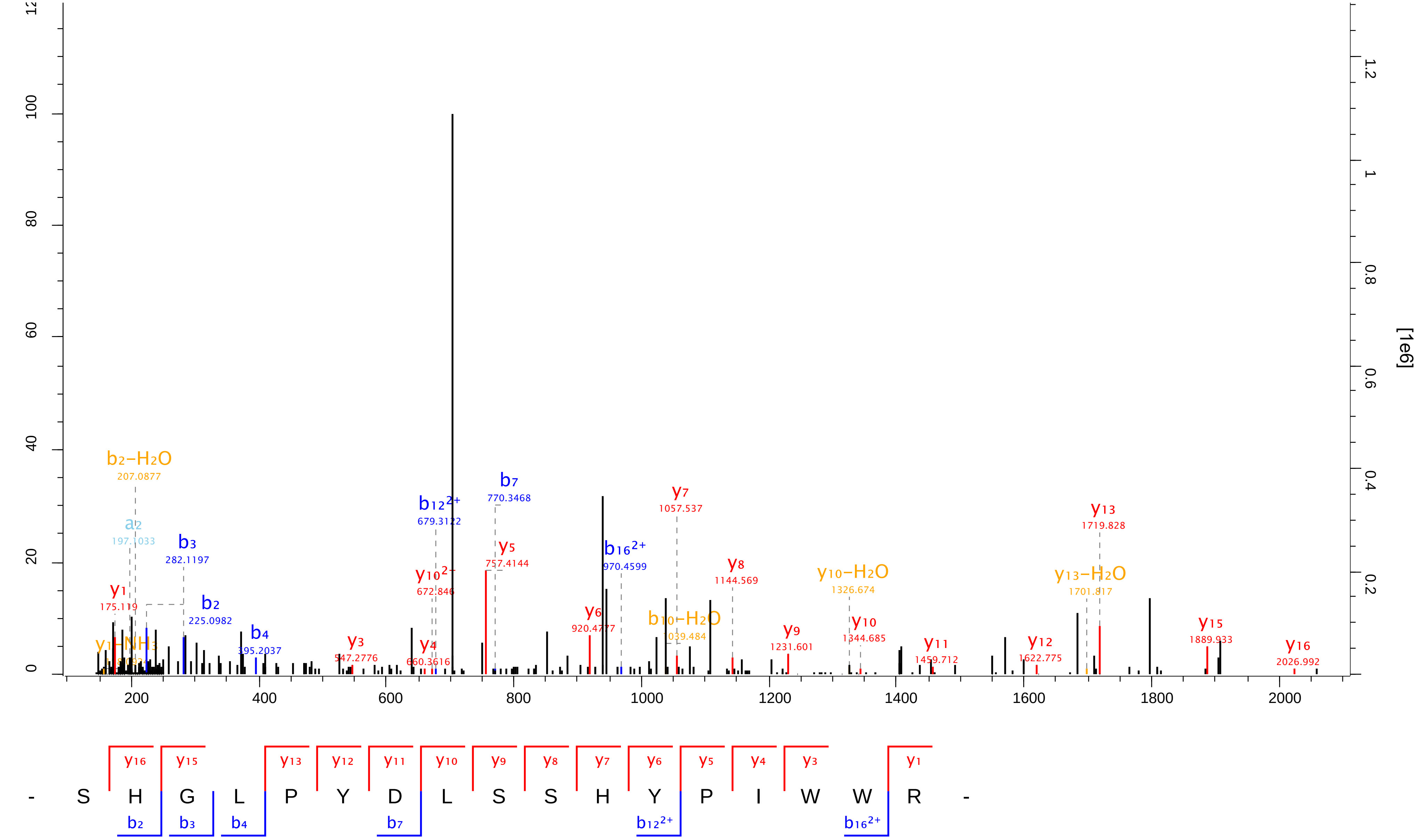

Raw file	Scan	Method	Score	m/z
QEplus003083	9574	FTMS; HCD	72.93	564.32

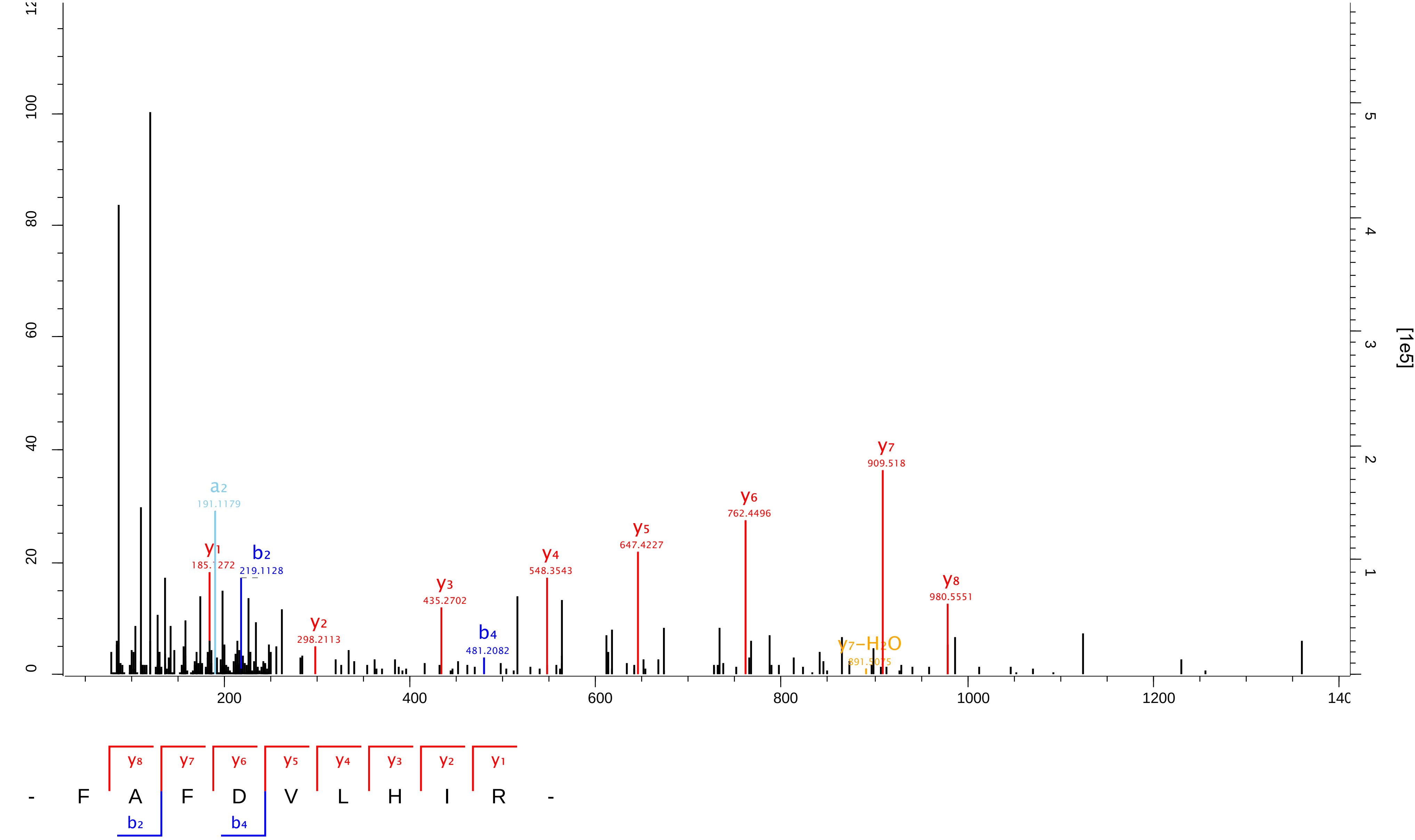

Raw file	Scan	Method	Score	m/z
QEplus003083	9831	FTMS; HCD	98.05	550.86

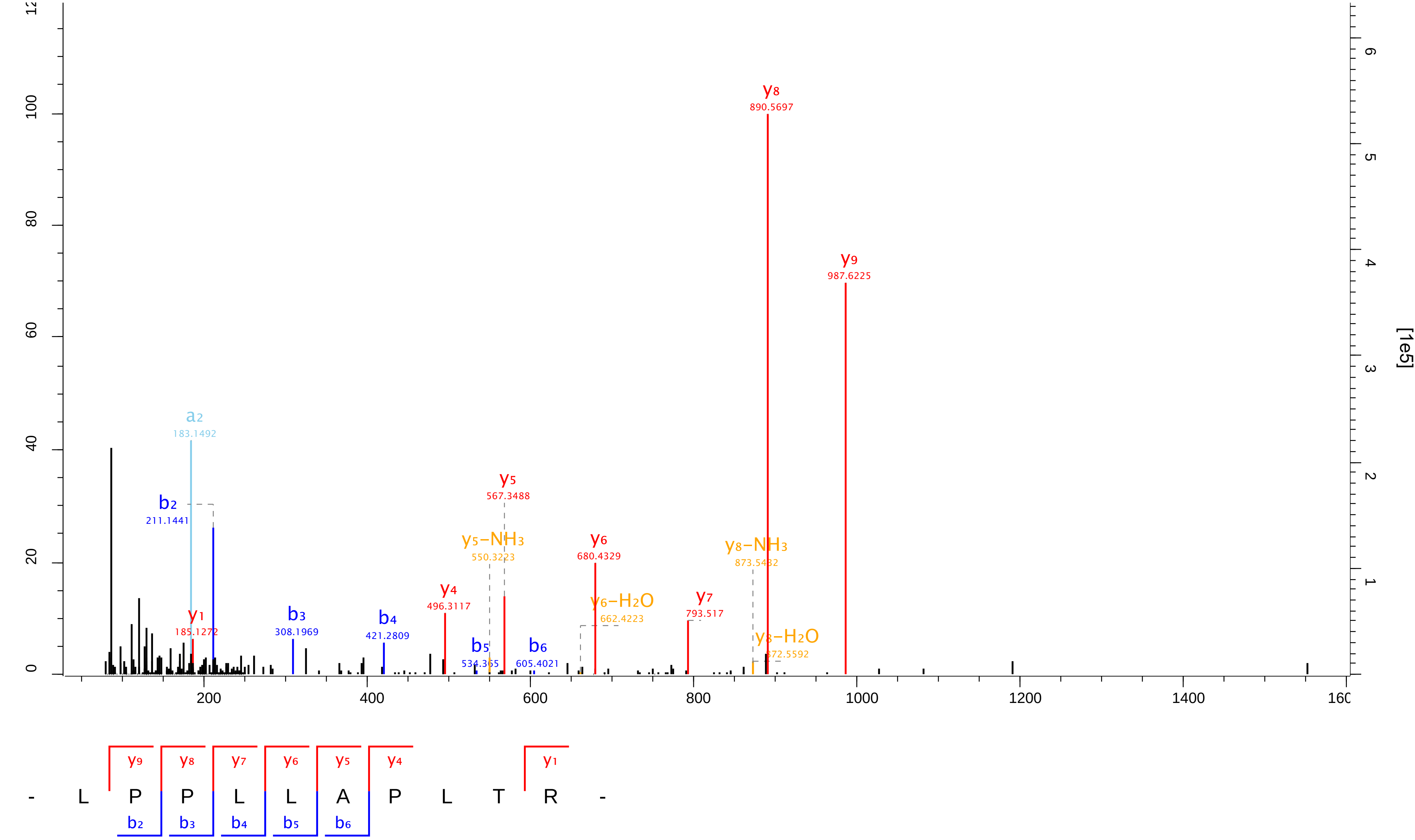

Raw file	Scan	Method	Score	m/z
QEplus003084	10076	FTMS; HCD	71.32	811.45

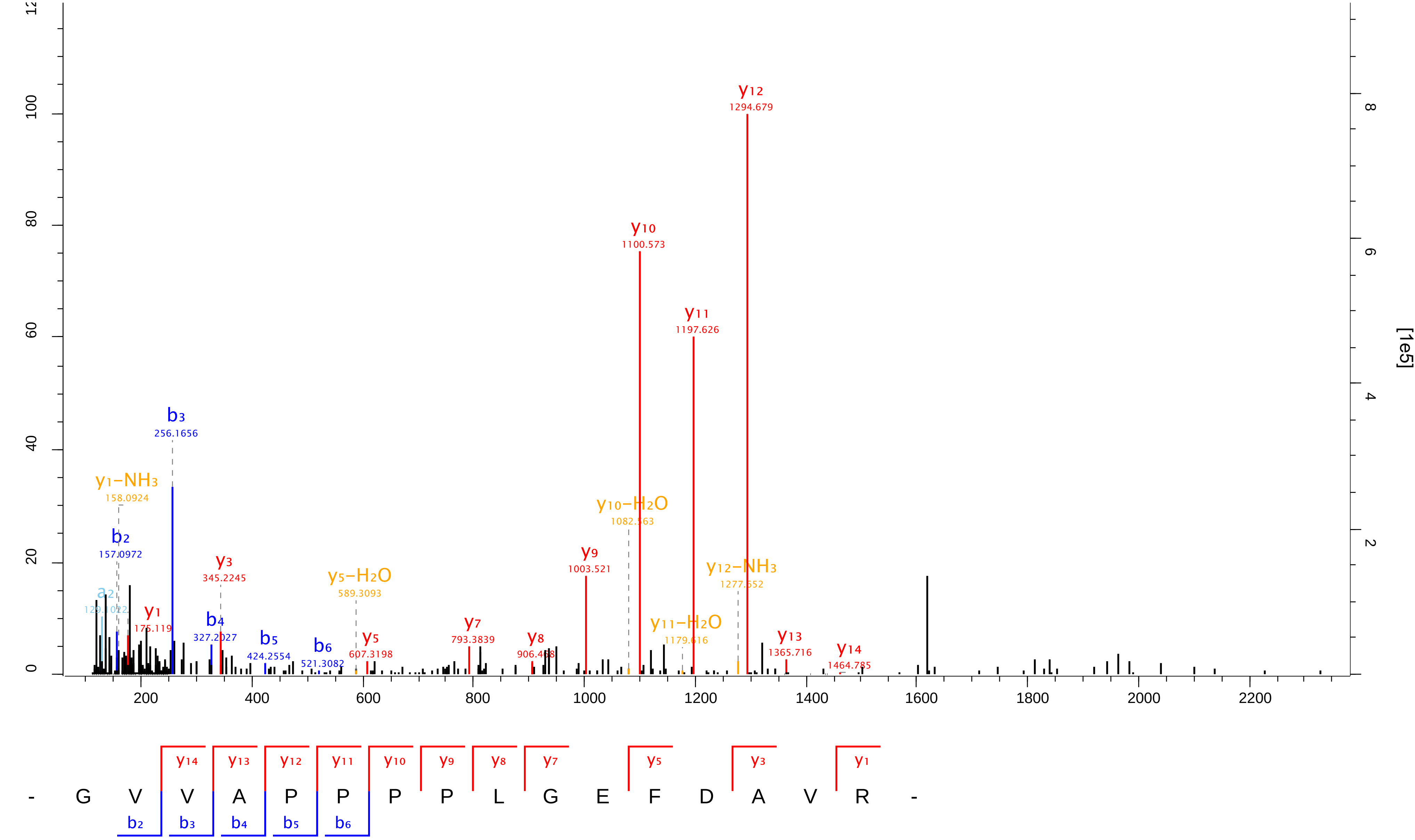

Raw file	Scan	Method	Score	m/z
QEplus003084	5776	FTMS; HCD	63.21	576.3

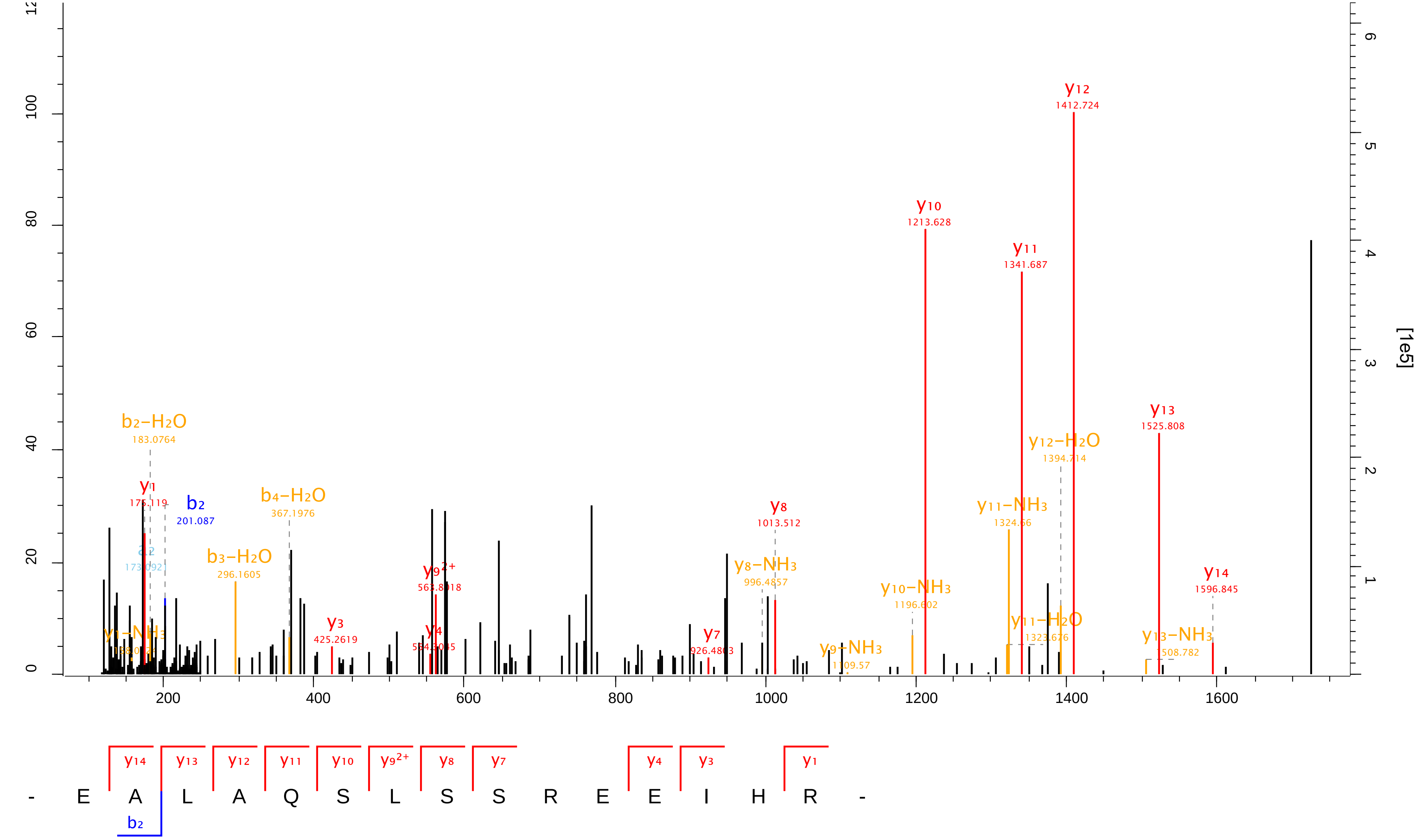

Raw file	Scan	Method	Score	m/z
QEplus003084	6375	FTMS; HCD	76.45	952.47

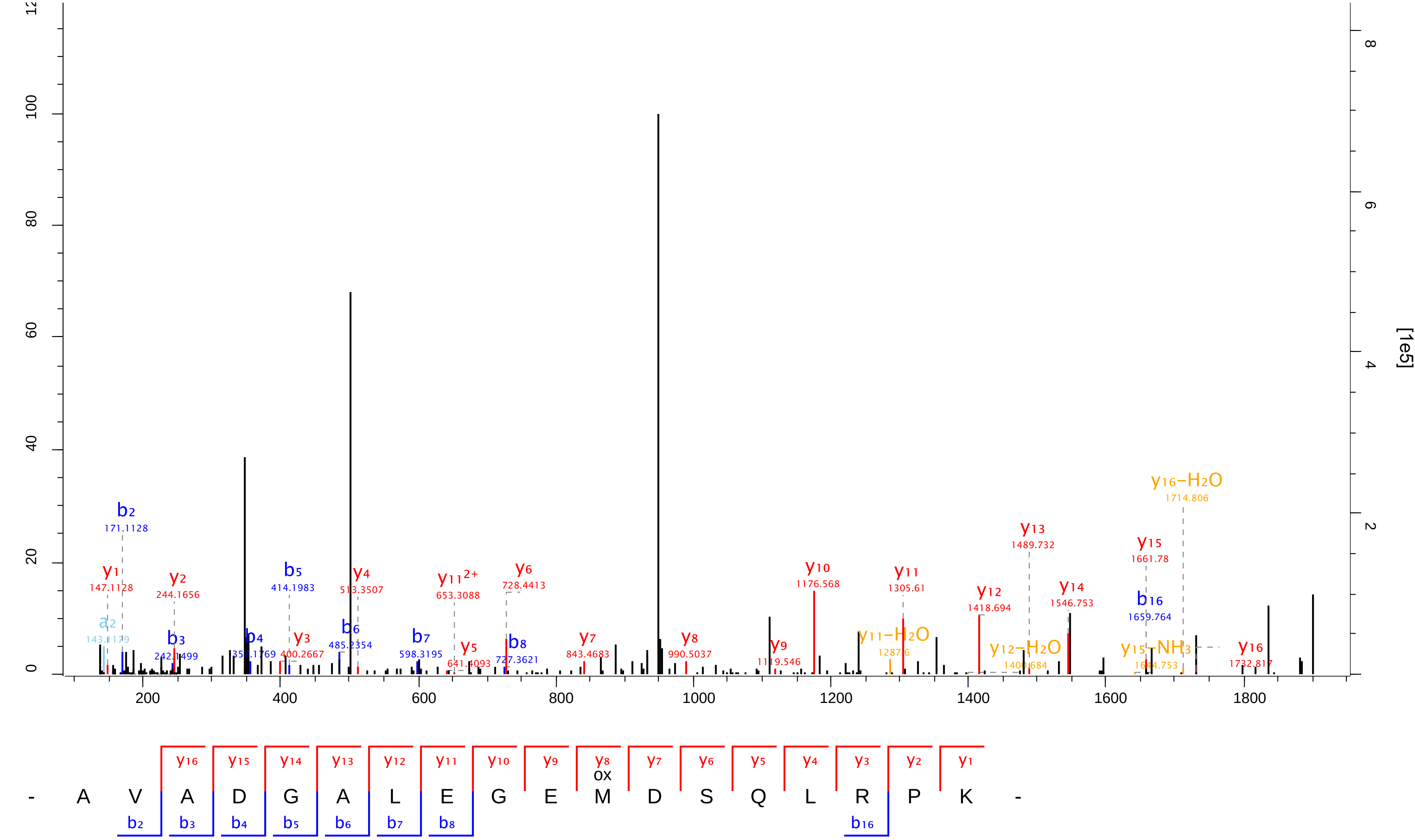

Raw file	Scan	Method	Score	m/z
QEplus003084	6721	FTMS; HCD	231.13	751.42

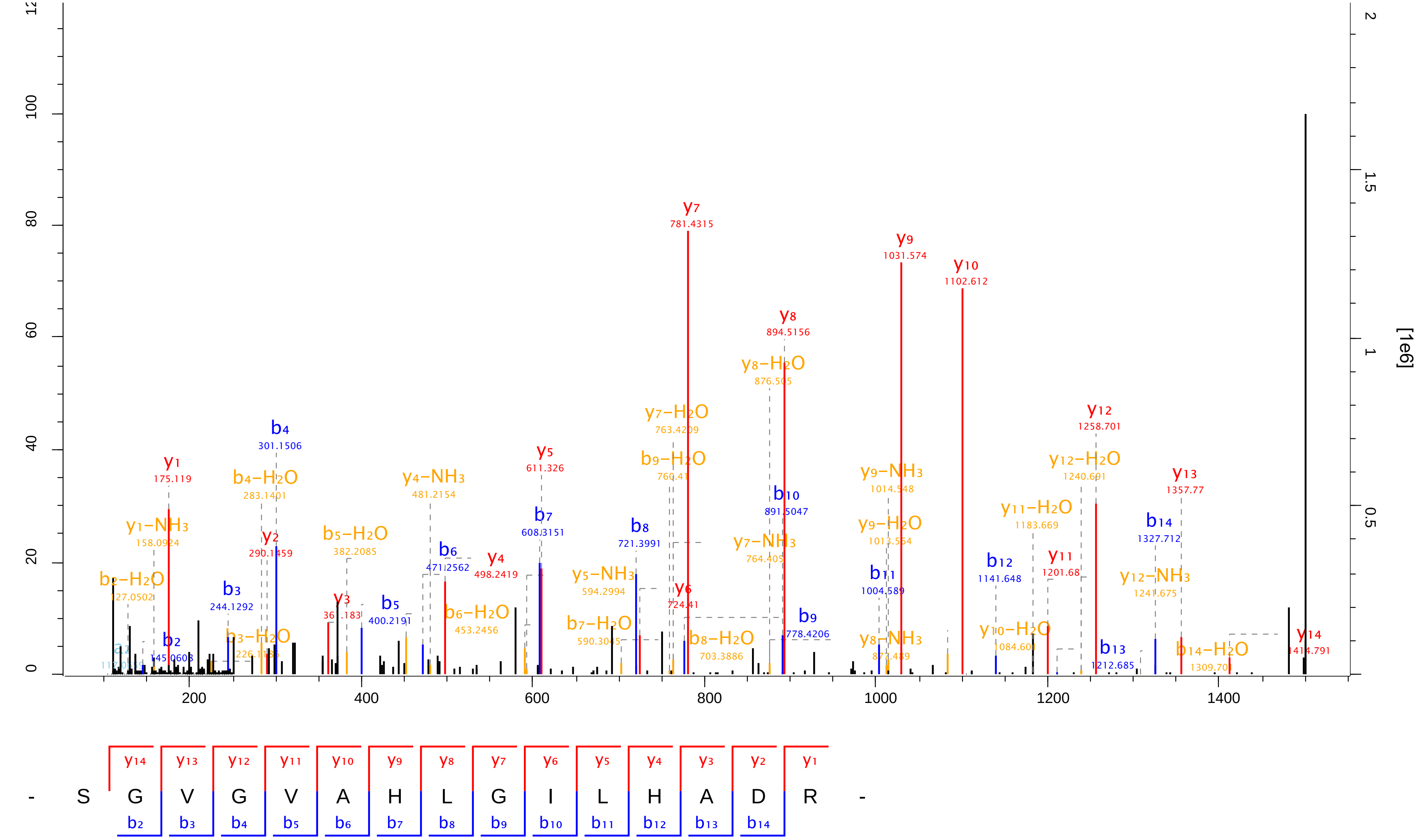

Raw file	Scan	Method	Score	m/z
QEplus003088	13865	FTMS; HCD	79.49	990.51

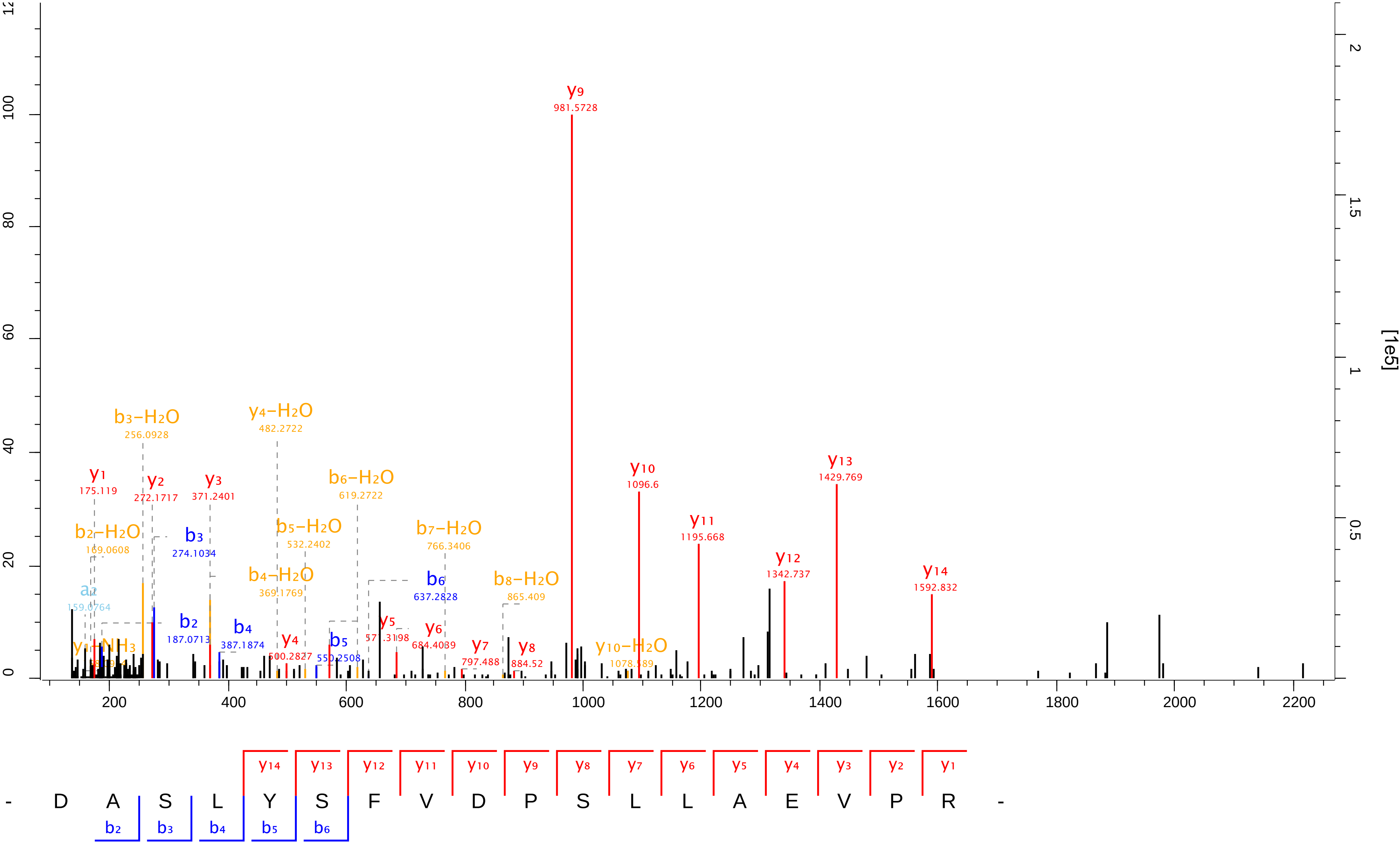

Raw file	Scan	Method	Score	m/z
QEplus003089	10300	FTMS; HCD	88.39	542.35

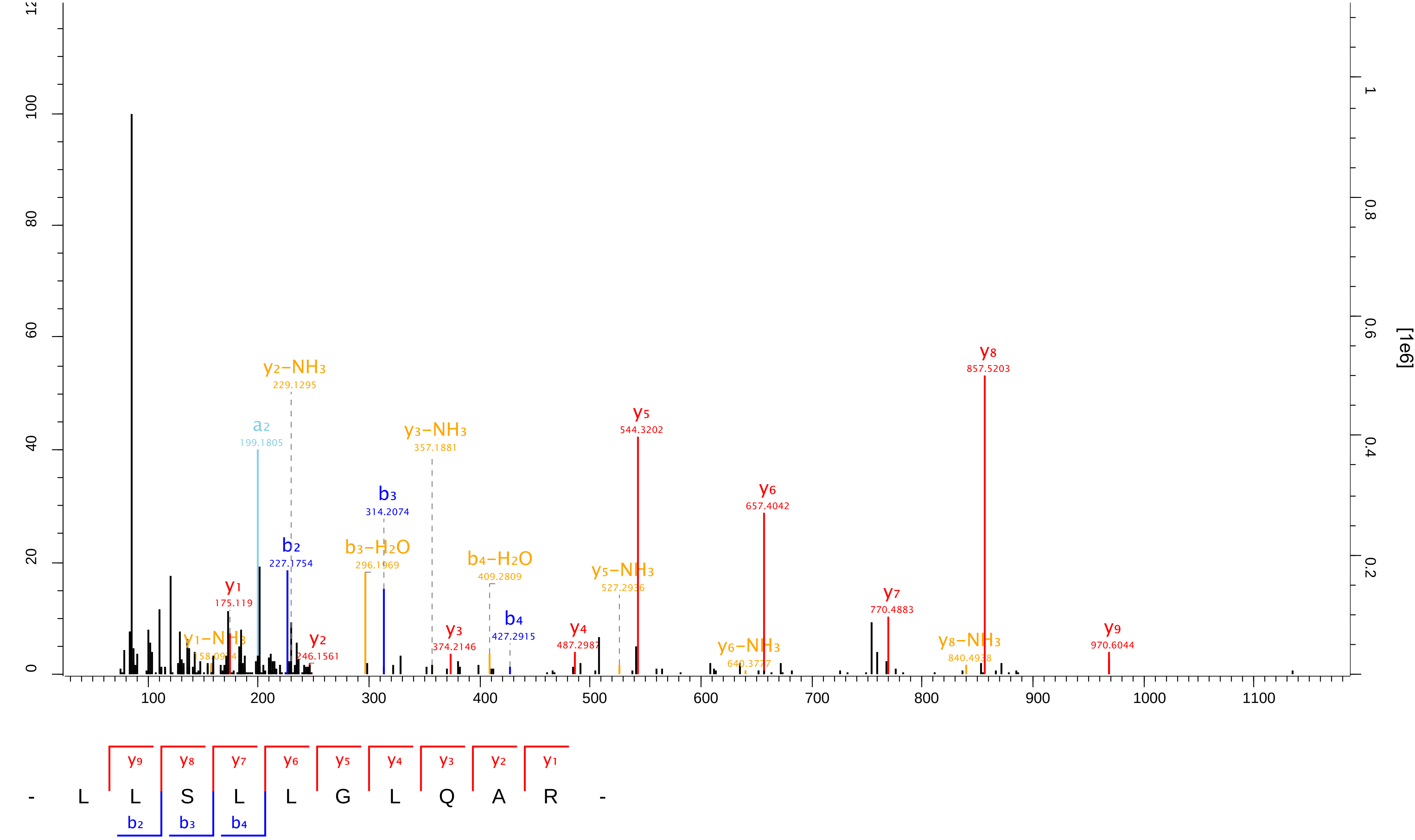

Raw file	Scan	Method	Score	m/z
QEplus003089	6247	FTMS; HCD	84.57	450.25

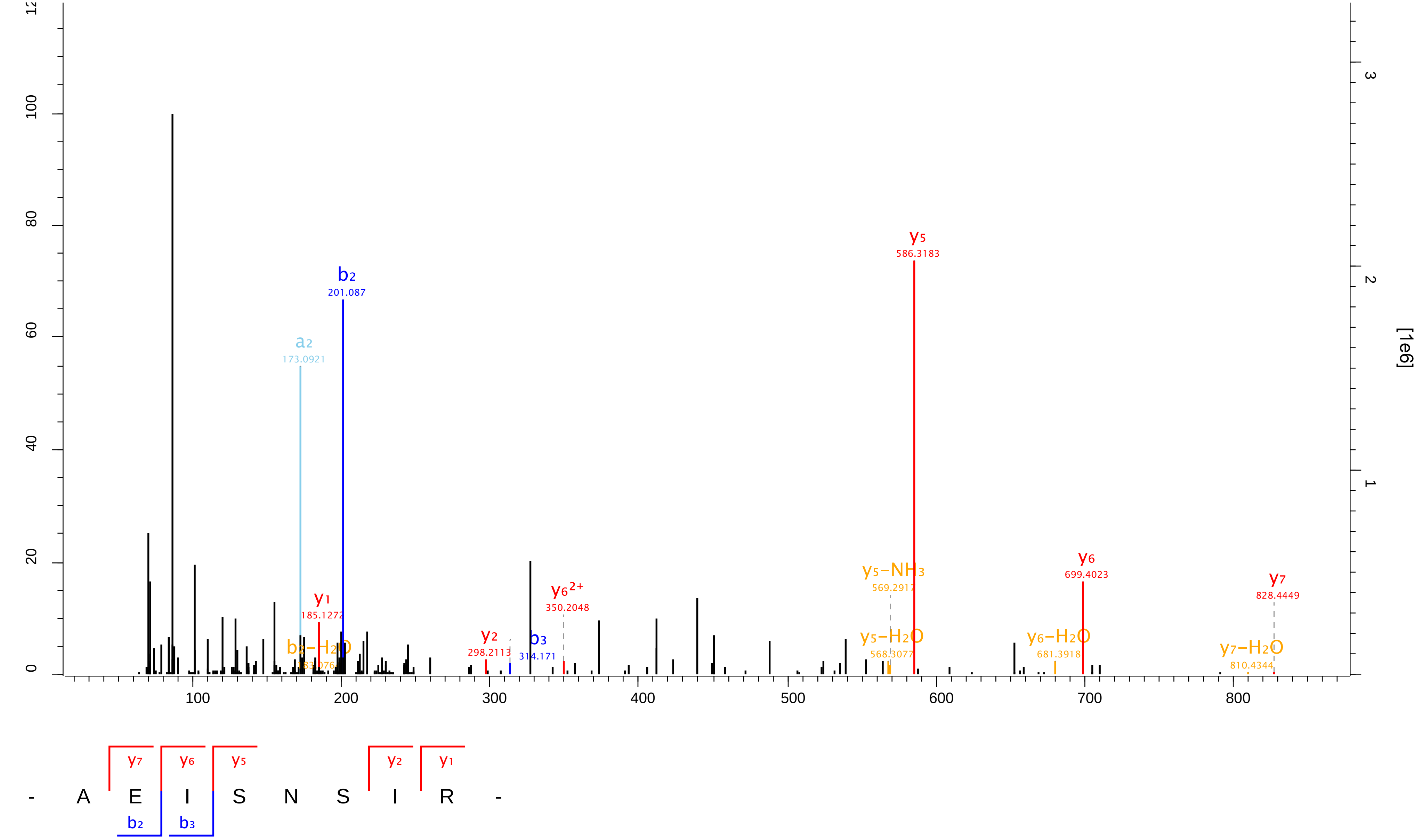

Raw file	Scan	Method	Score	m/z
QEplus003090	12125	FTMS; HCD	72.29	749.41

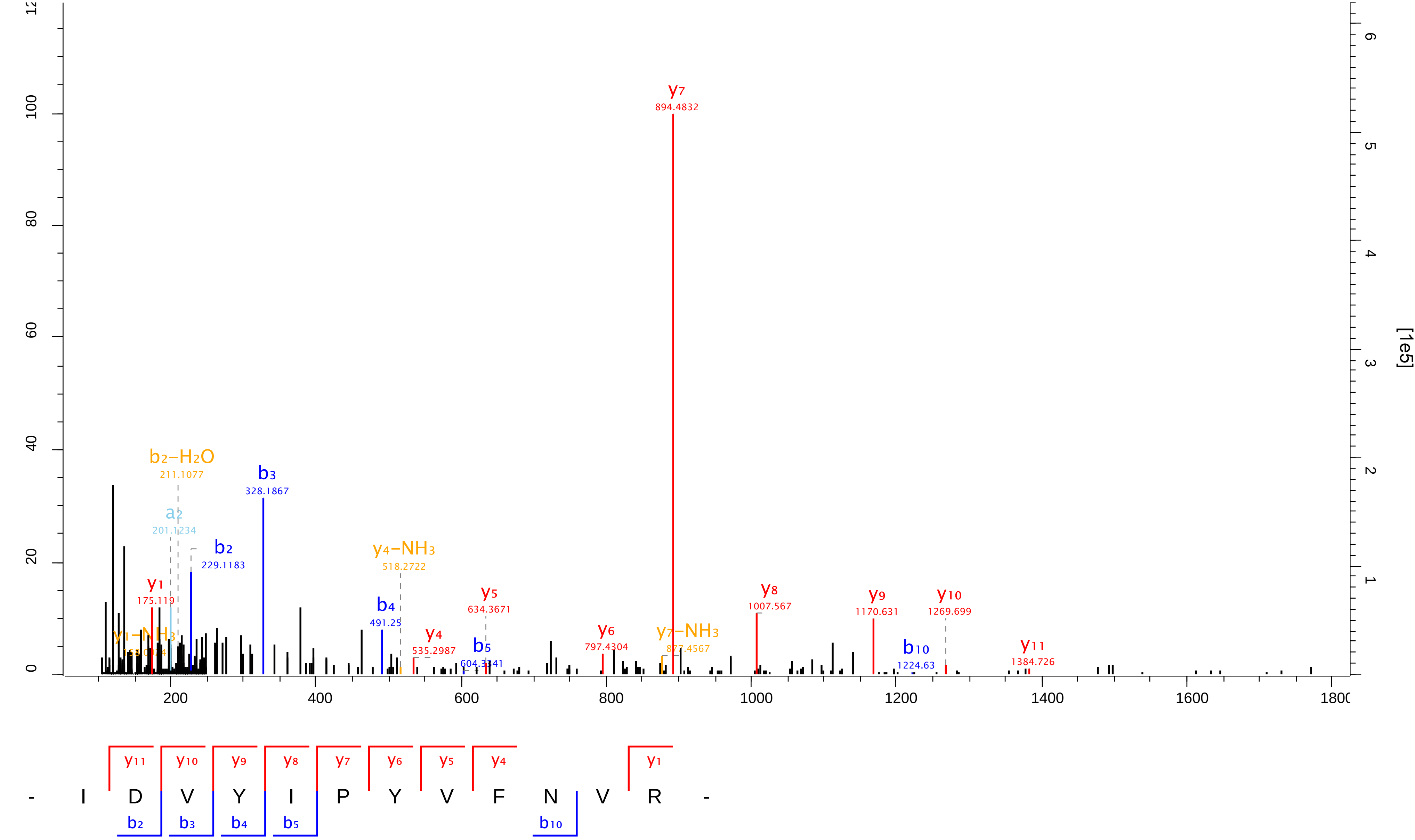

Raw file	Scan	Method	Score	m/z
QEplus003090	12930	FTMS; HCD	166.9	603.99

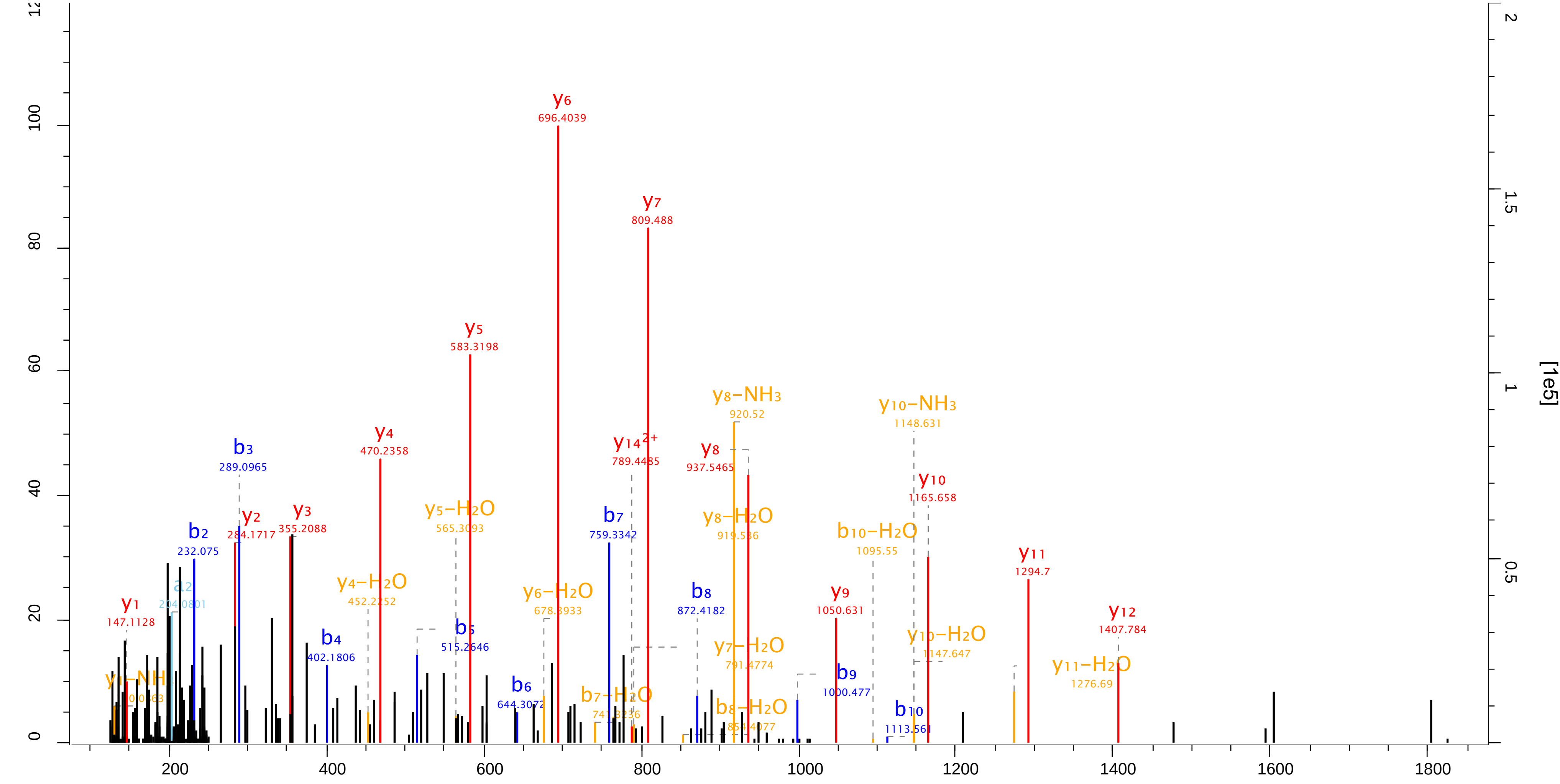

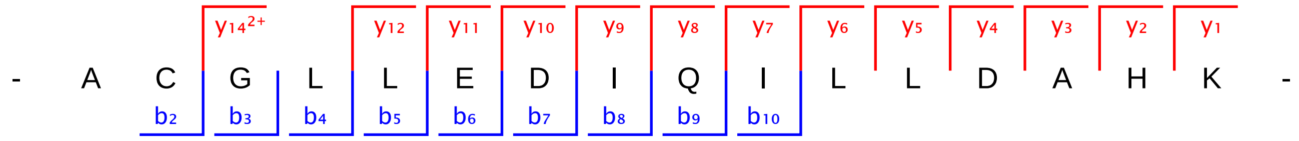

Raw file	Scan	Method	Score	m/z
QEplus003090	13557	FTMS; HCD	53.6	650.38

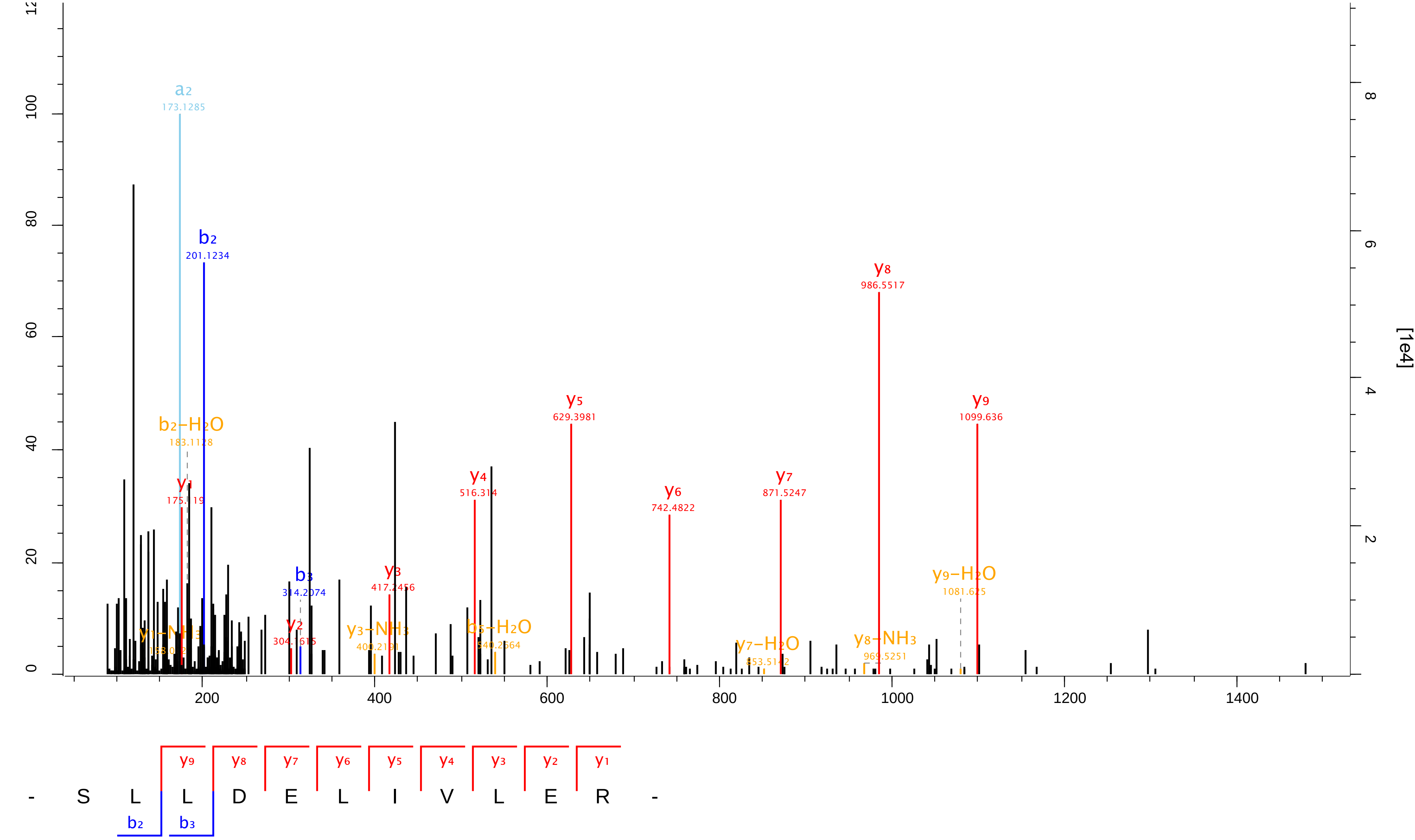

Raw file	Scan	Method	Score	m/z
QEplus003090	4002	FTMS; HCD	64.24	506.61

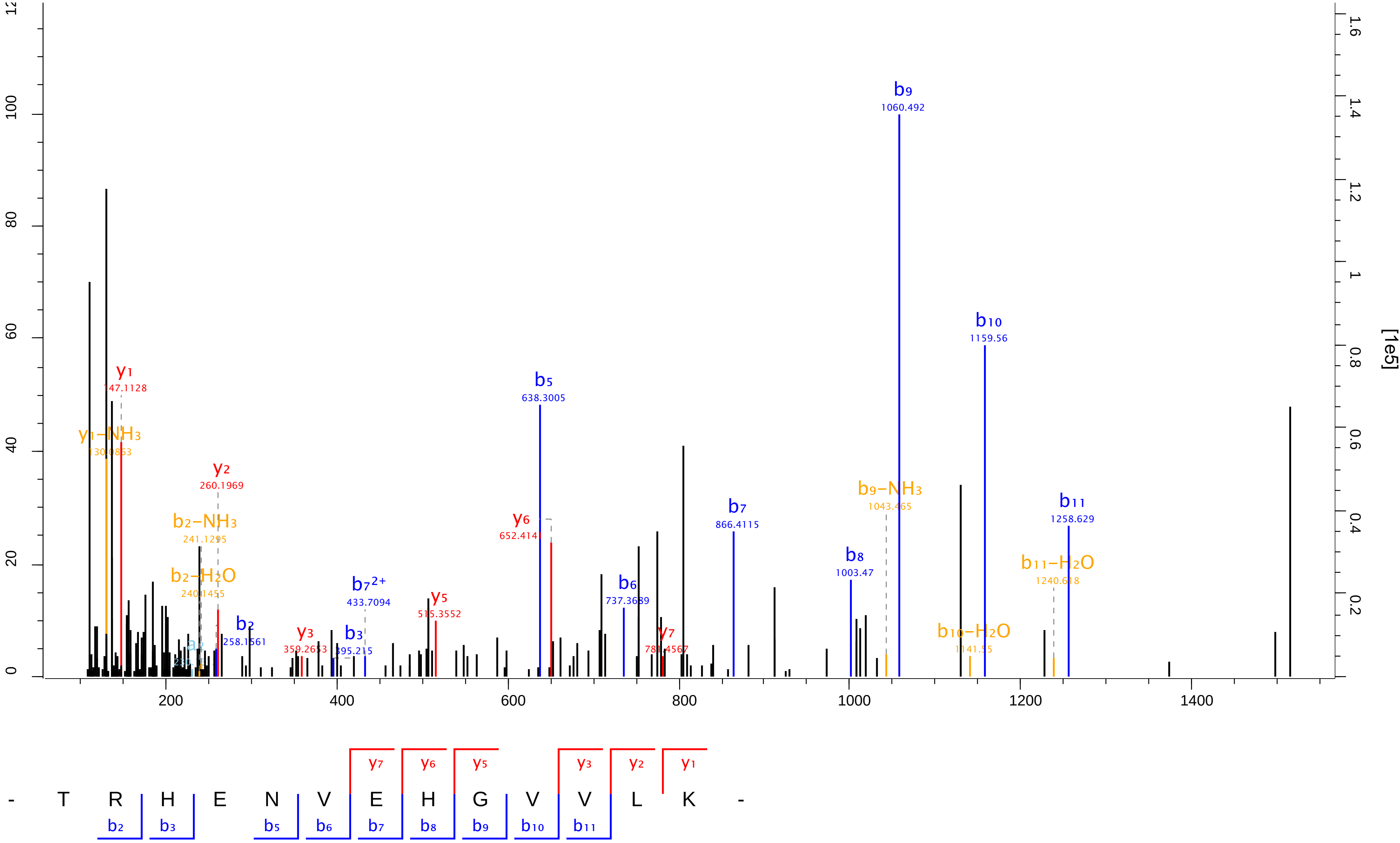

Raw file	Scan	Method	Score	m/z
QEplus003090	7236	FTMS; HCD	97.24	746.42

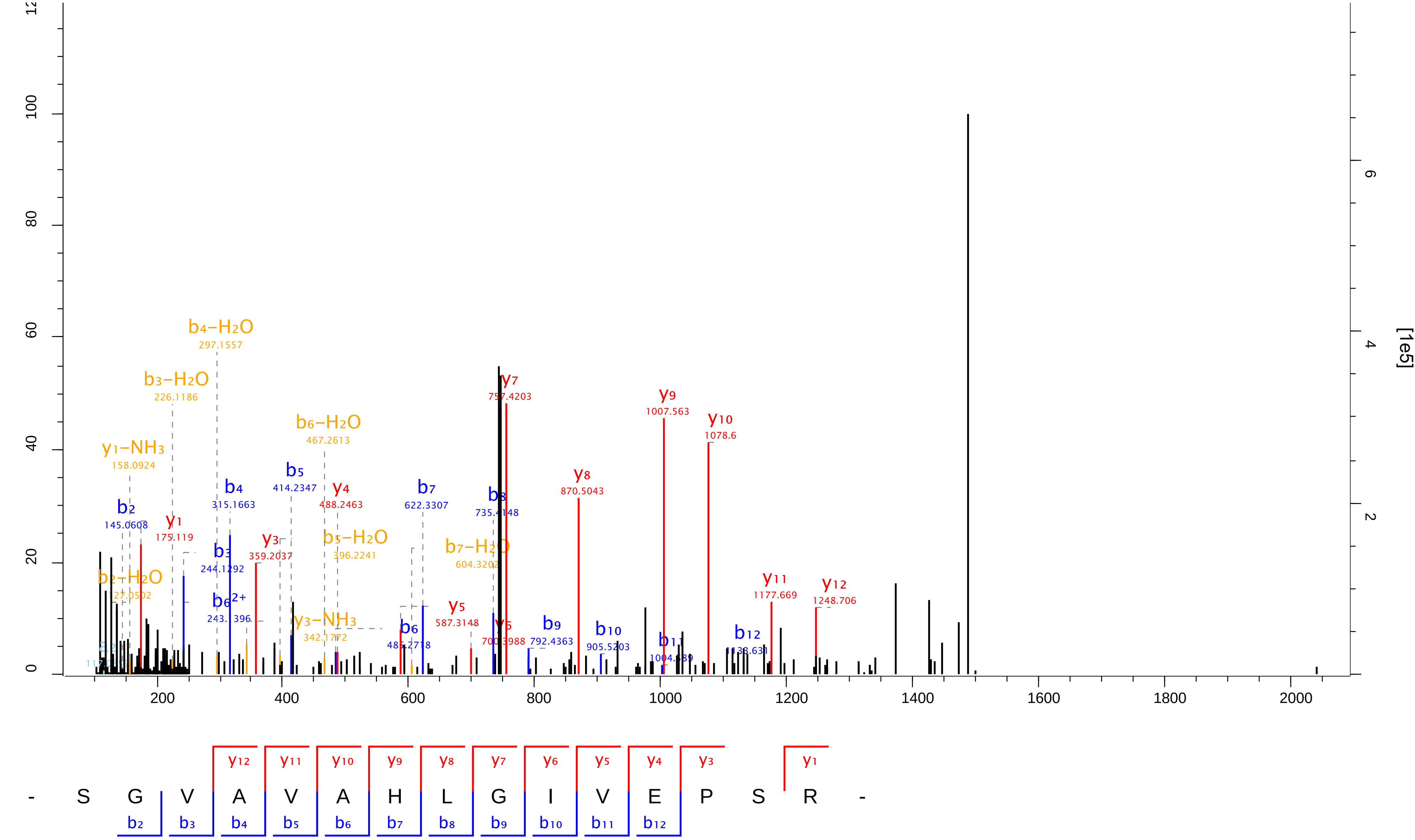

Raw file	Scan	Method	Score	m/z
QEplus003090	7845	FTMS; HCD	114.7	548.63

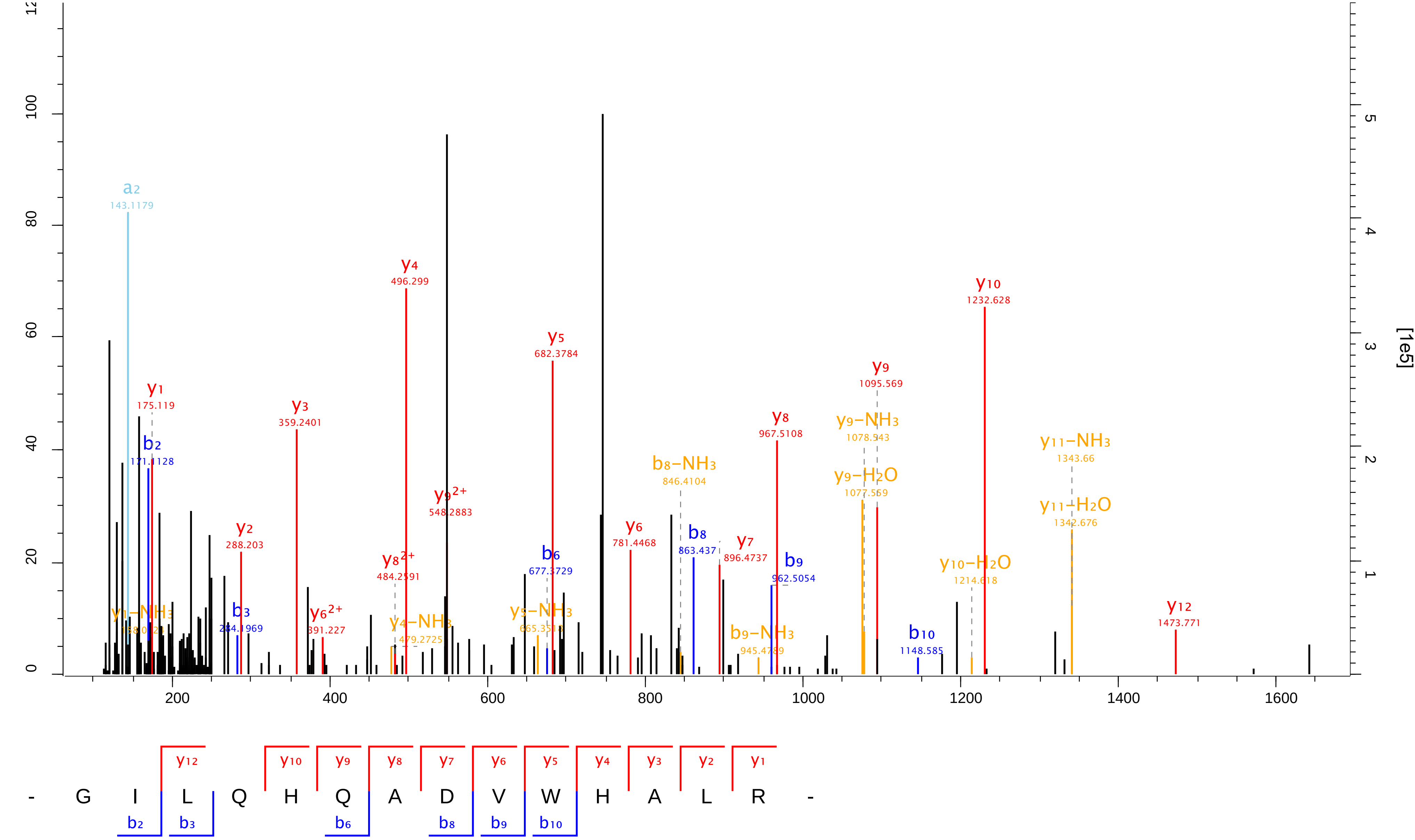

Raw file	Scan	Method	Score	m/z
QEplus003091	12996	FTMS; HCD	71.88	711.4

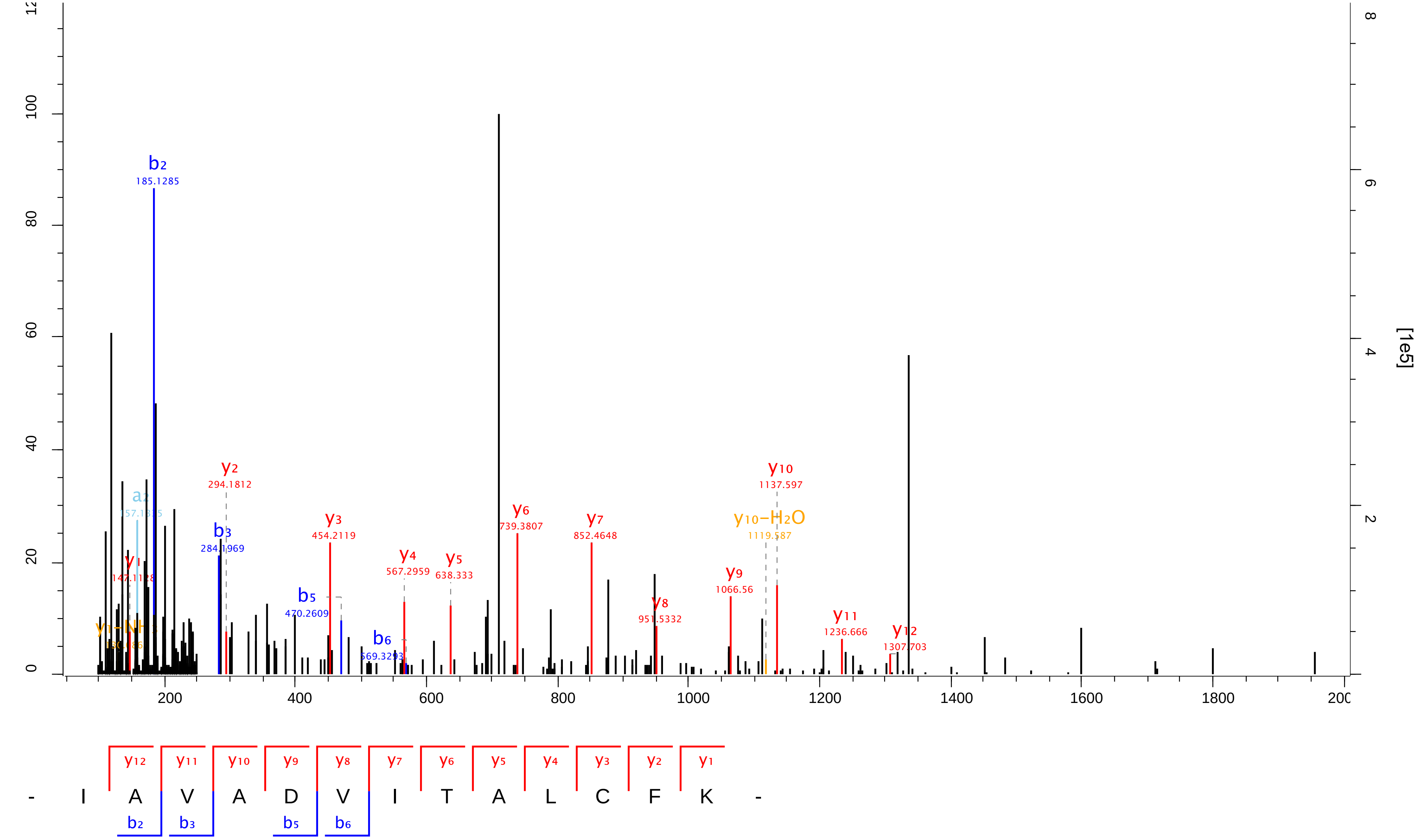

Raw file	Scan	Method	Score	m/z
QEplus003091	14091	FTMS; HCD	44.1	638.03

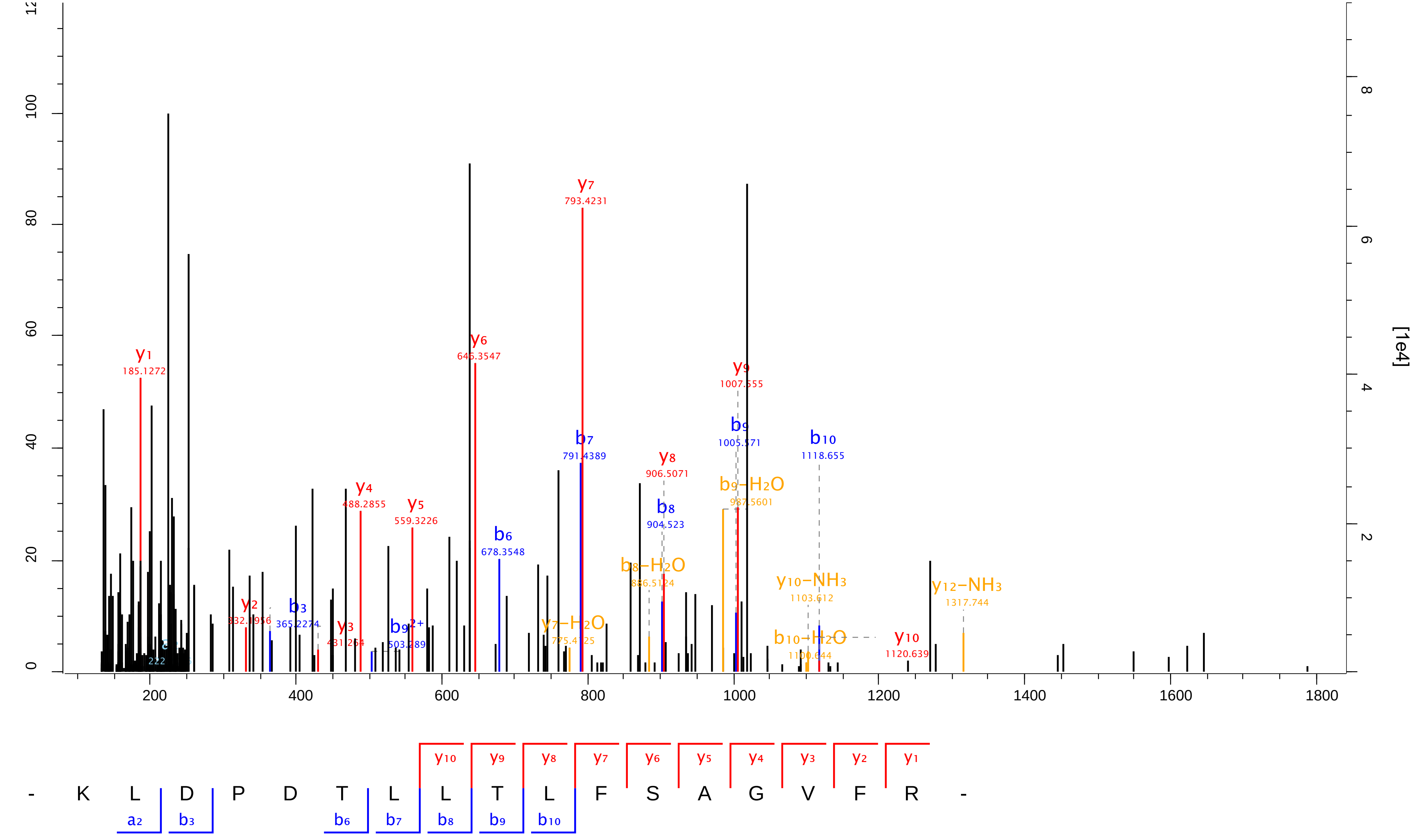

Raw file	Scan	Method	Score	m/z
QEplus003091	14439	FTMS; HCD	100.11	818.93

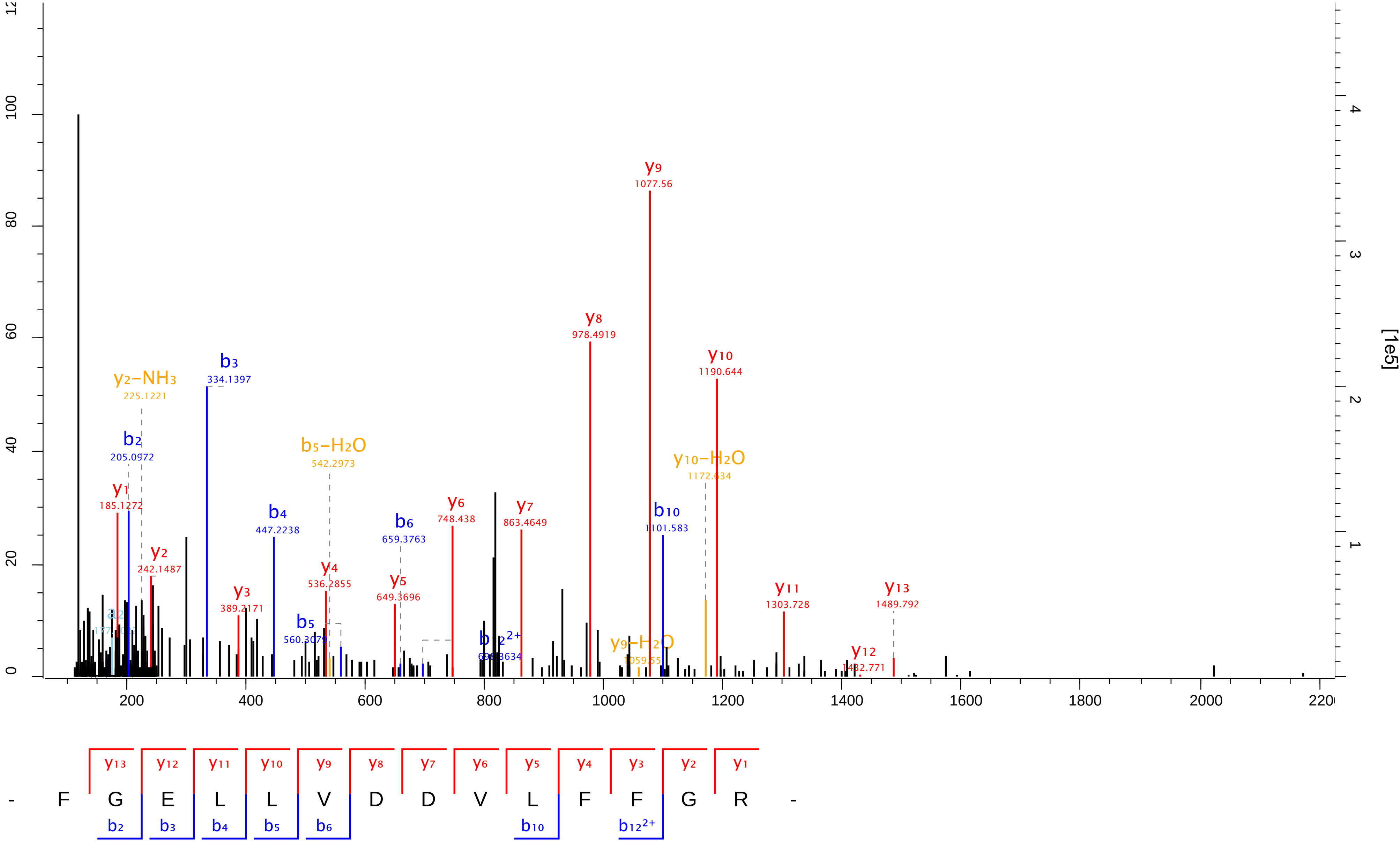

Raw file	Scan	Method	Score	m/z
QEplus003091	14506	FTMS; HCD	95.43	692.91

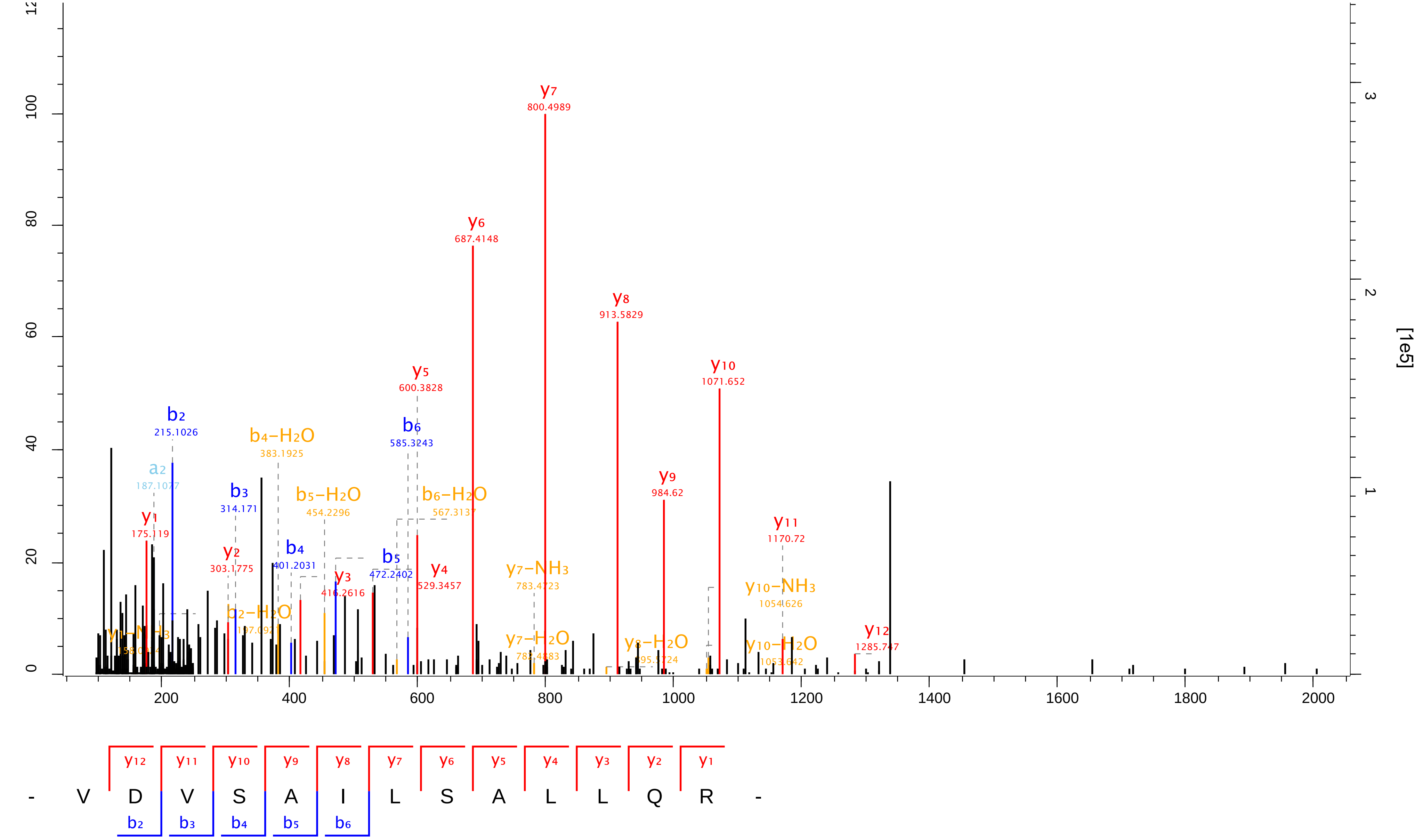

Raw file	Scan	Method	Score	m/z
QEplus003091	8248	FTMS; HCD	85.26	448.74

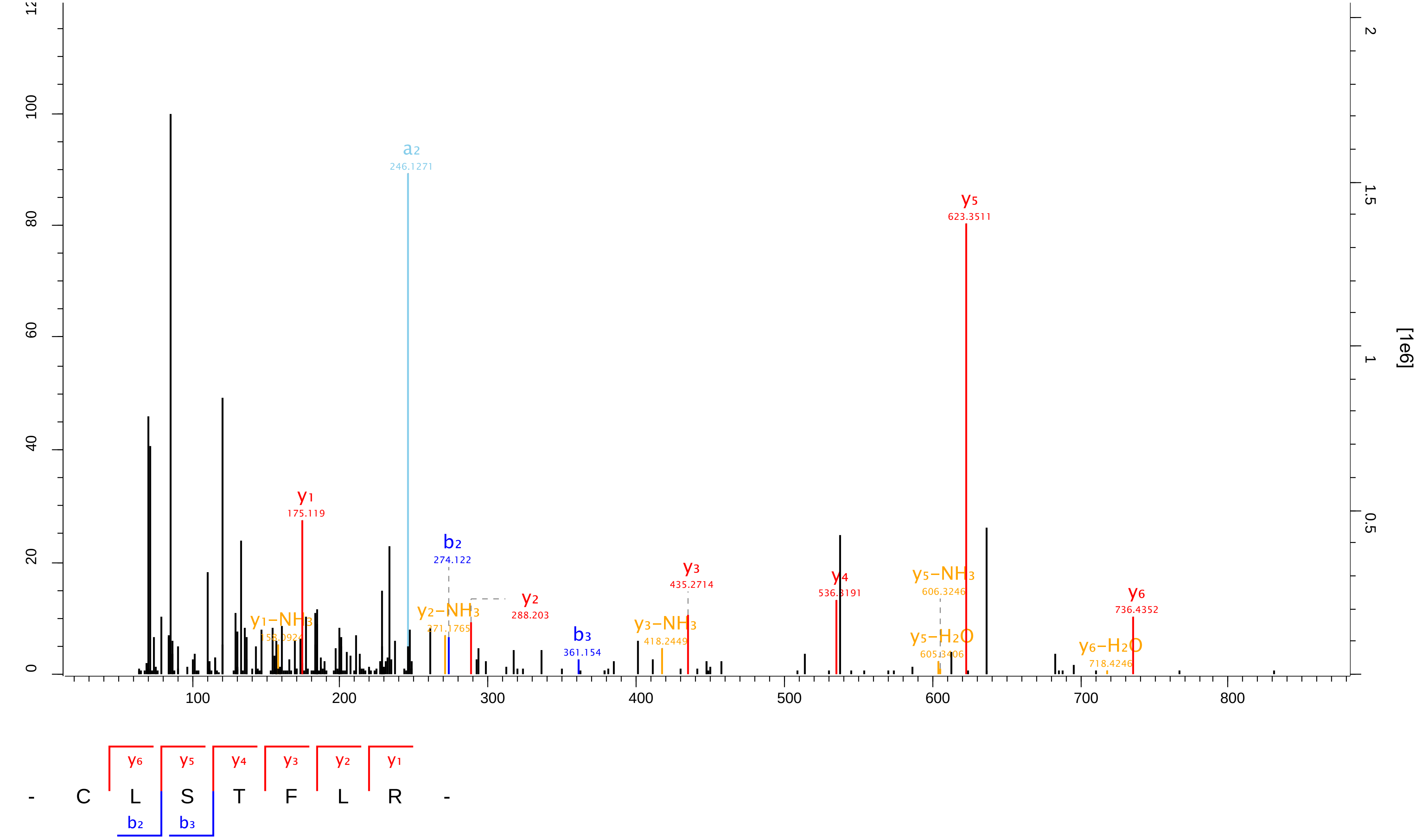

Raw file	Scan	Method	Score	m/z
QEplus003092	11123	FTMS; HCD	65.84	615.36

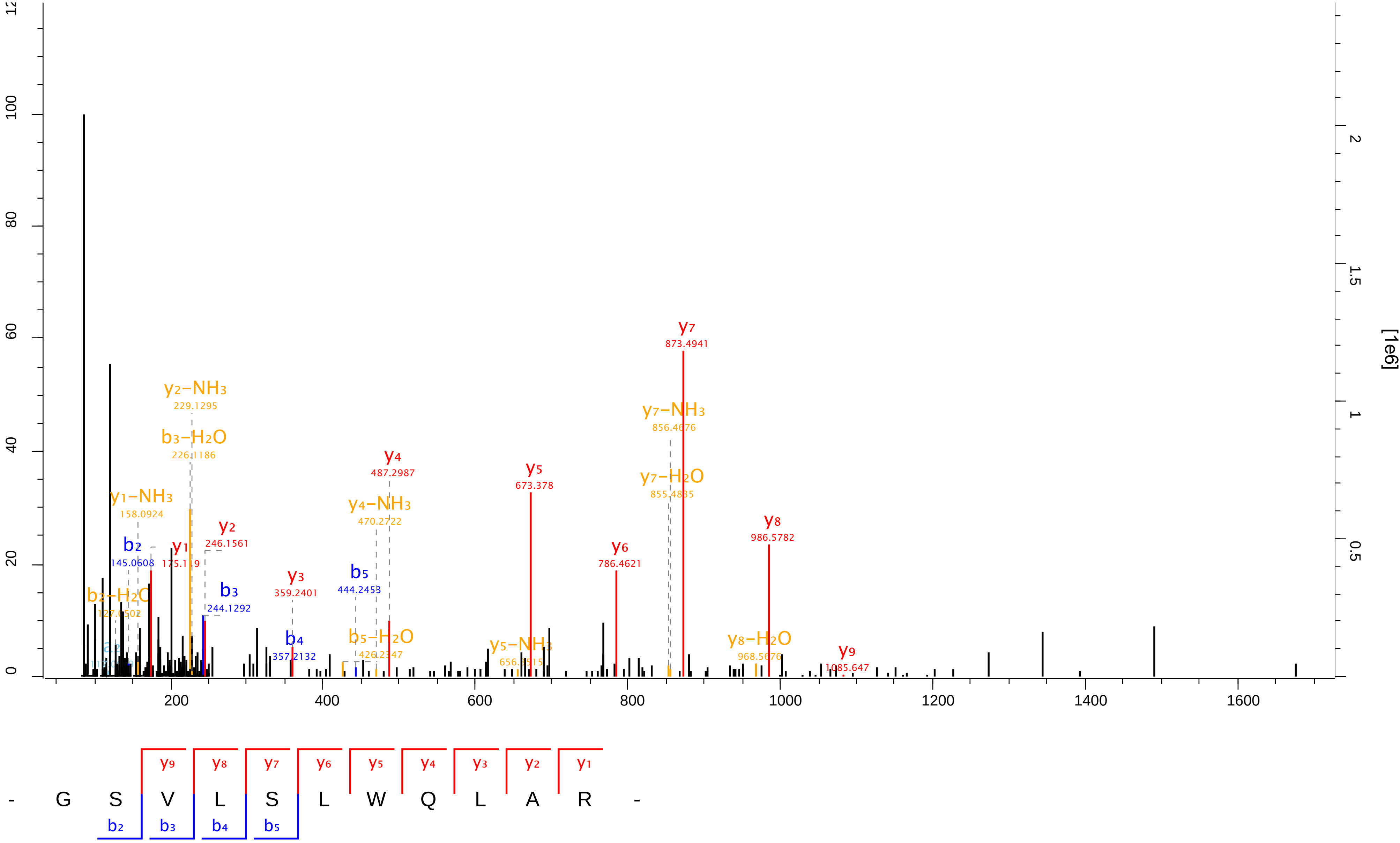

Raw file	Scan	Method	Score	m/z
QEplus003092	12416	FTMS; HCD	72.88	553.8

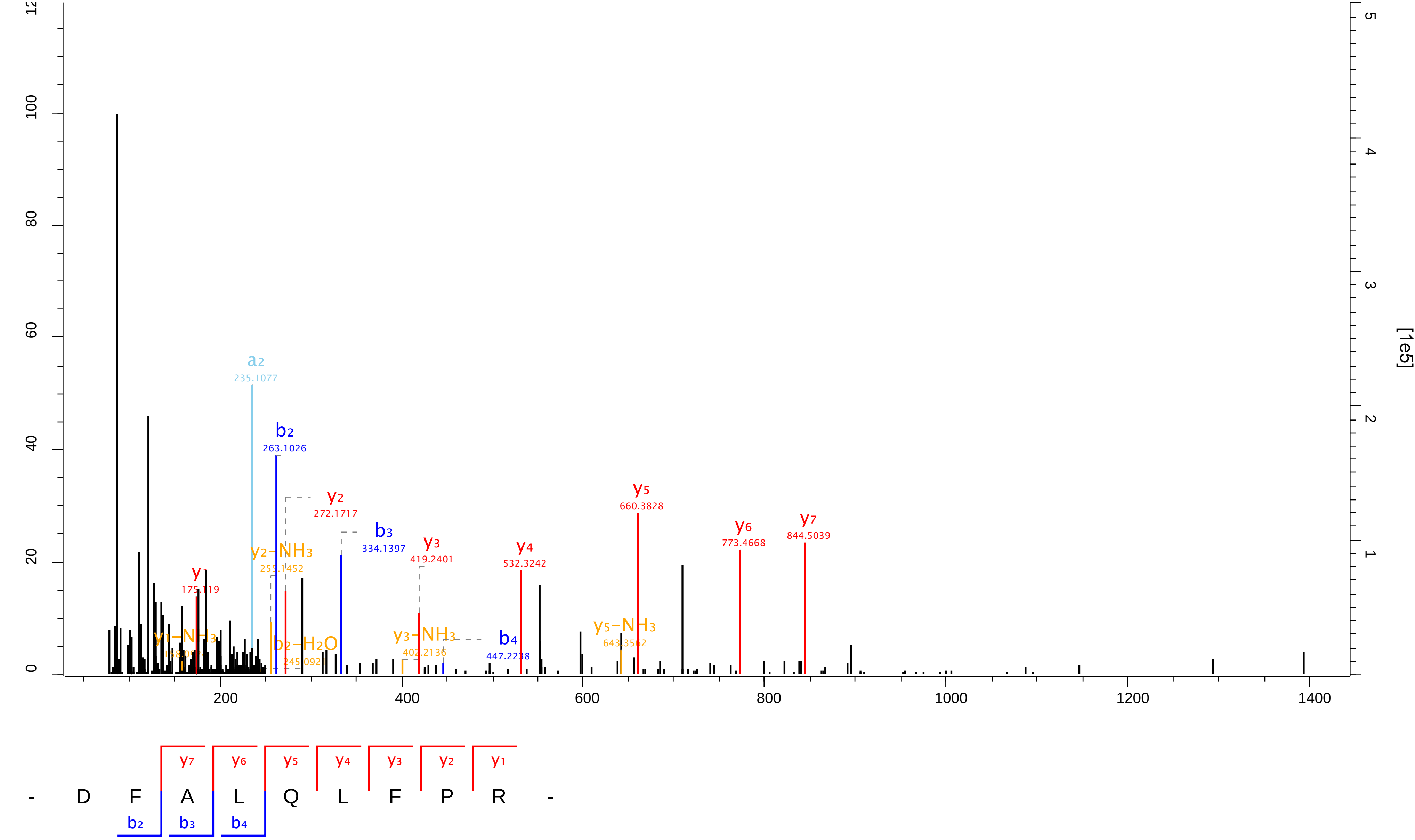

Raw file	Scan	Method	Score	m/z
QEplus003092	13082	FTMS; HCD	106.42	843.76

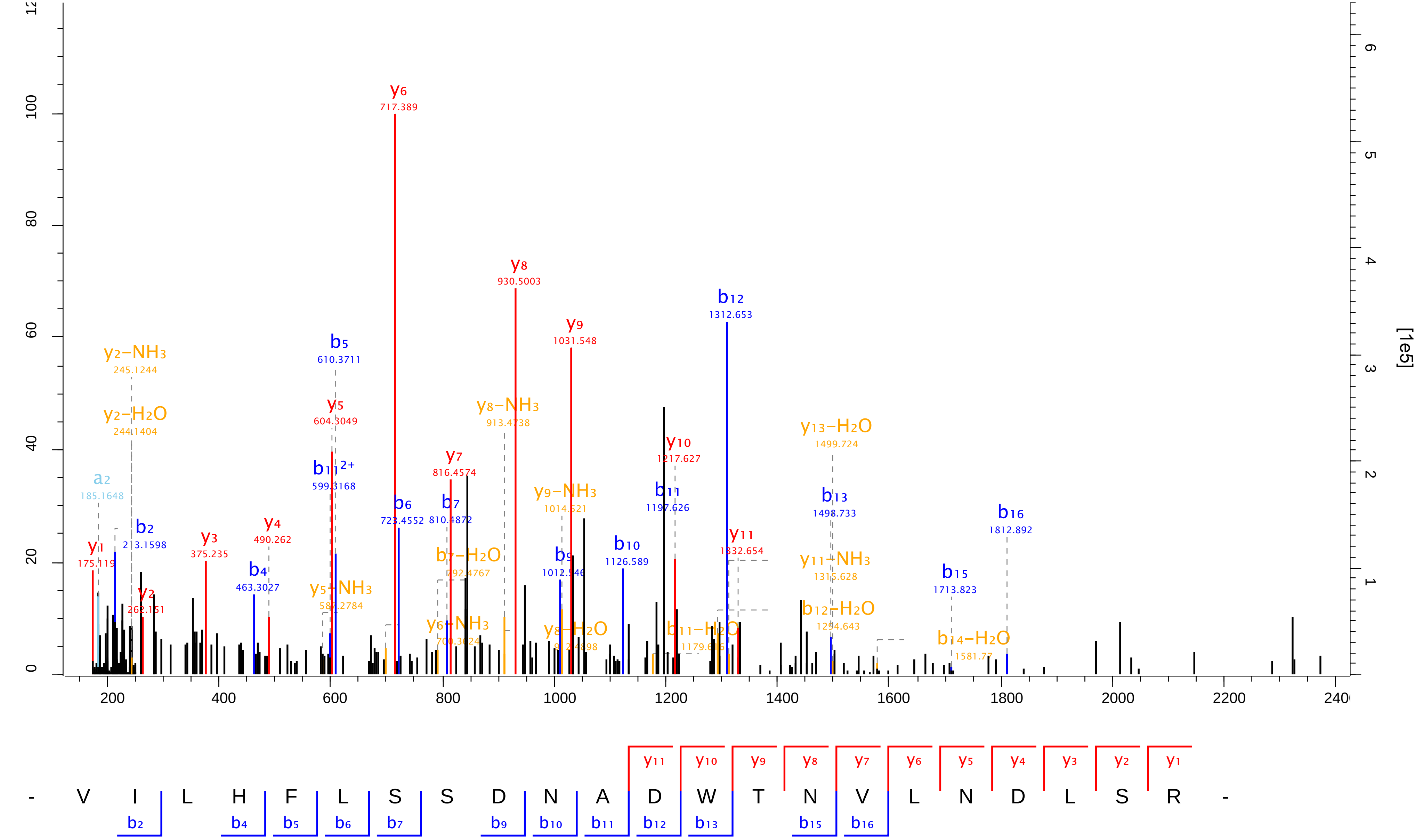

Raw file	Scan	Method	Score	m/z
QEplus003092	13712	FTMS; HCD	122.7	682.38

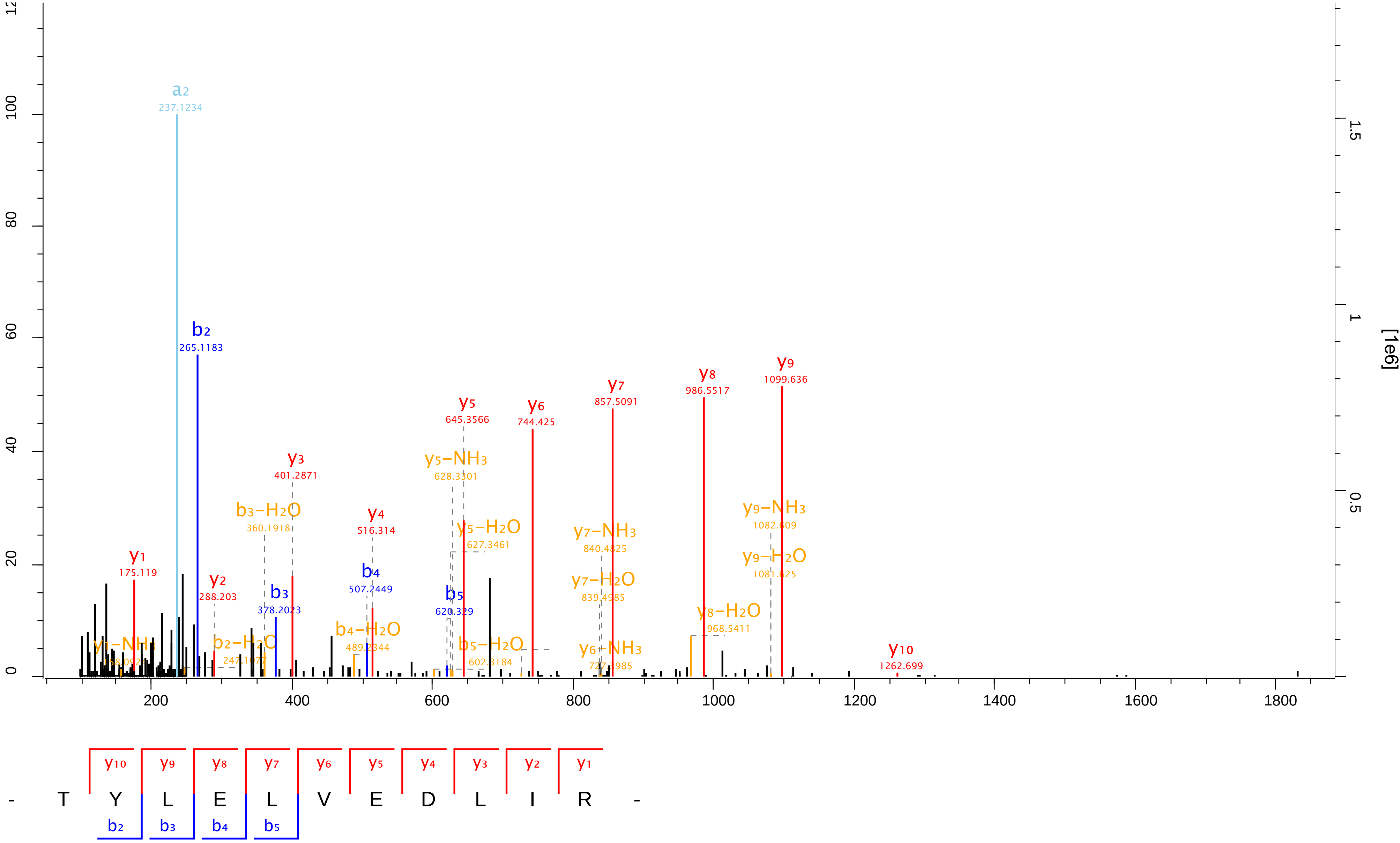

Raw file	Scan	Method	Score	m/z
QEplus003092	14393	FTMS; HCD	87.25	788.94

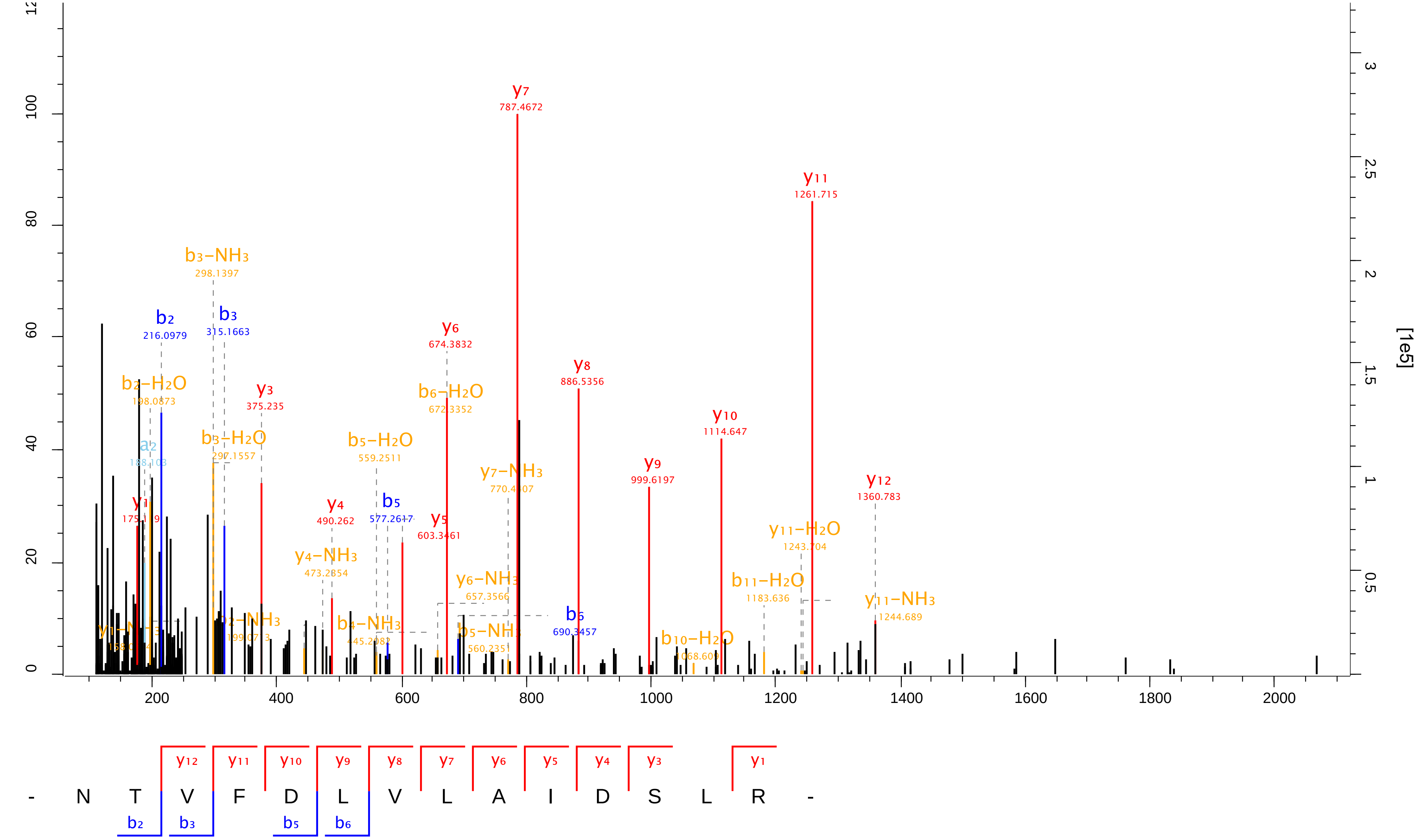

Raw file	Scan	Method	Score	m/z
QEplus003093	10833	FTMS; HCD	107.32	479.3

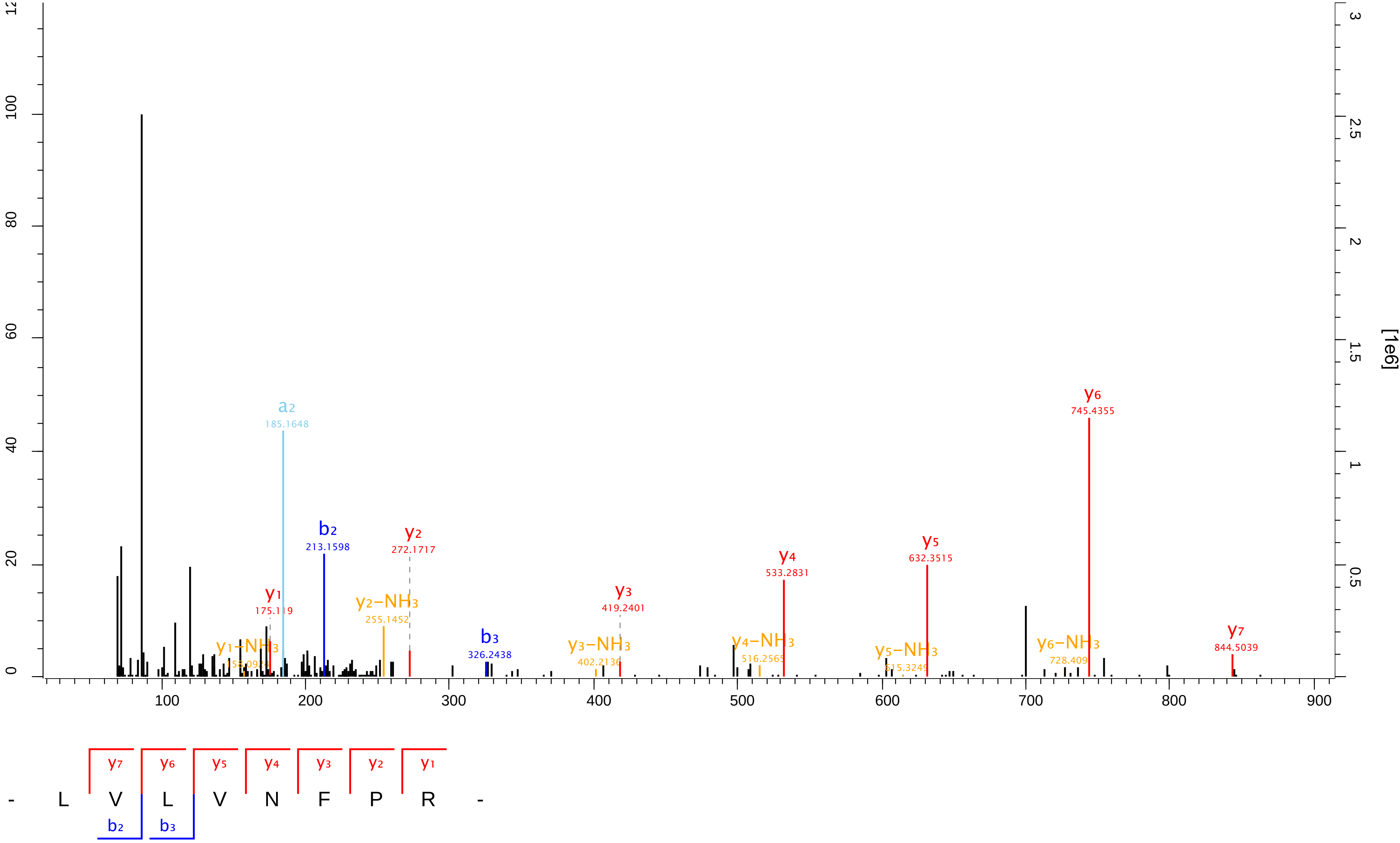

Raw file	Scan	Method	Score	m/z
QEplus003093	12202	FTMS; HCD	54.83	745.4

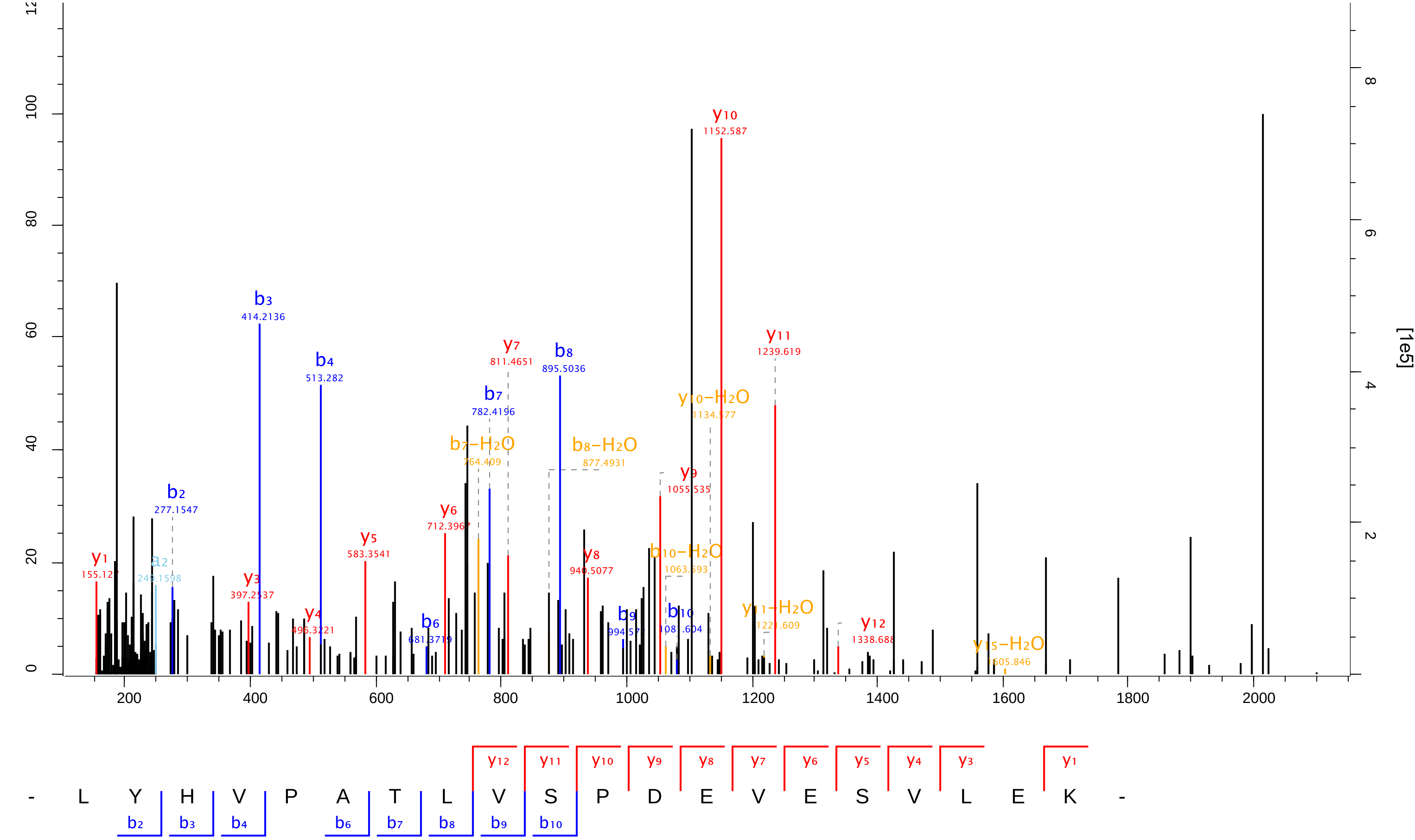

Raw file	Scan	Method	Score	m/z
QEplus003093	12468	FTMS; HCD	54.26	585.84

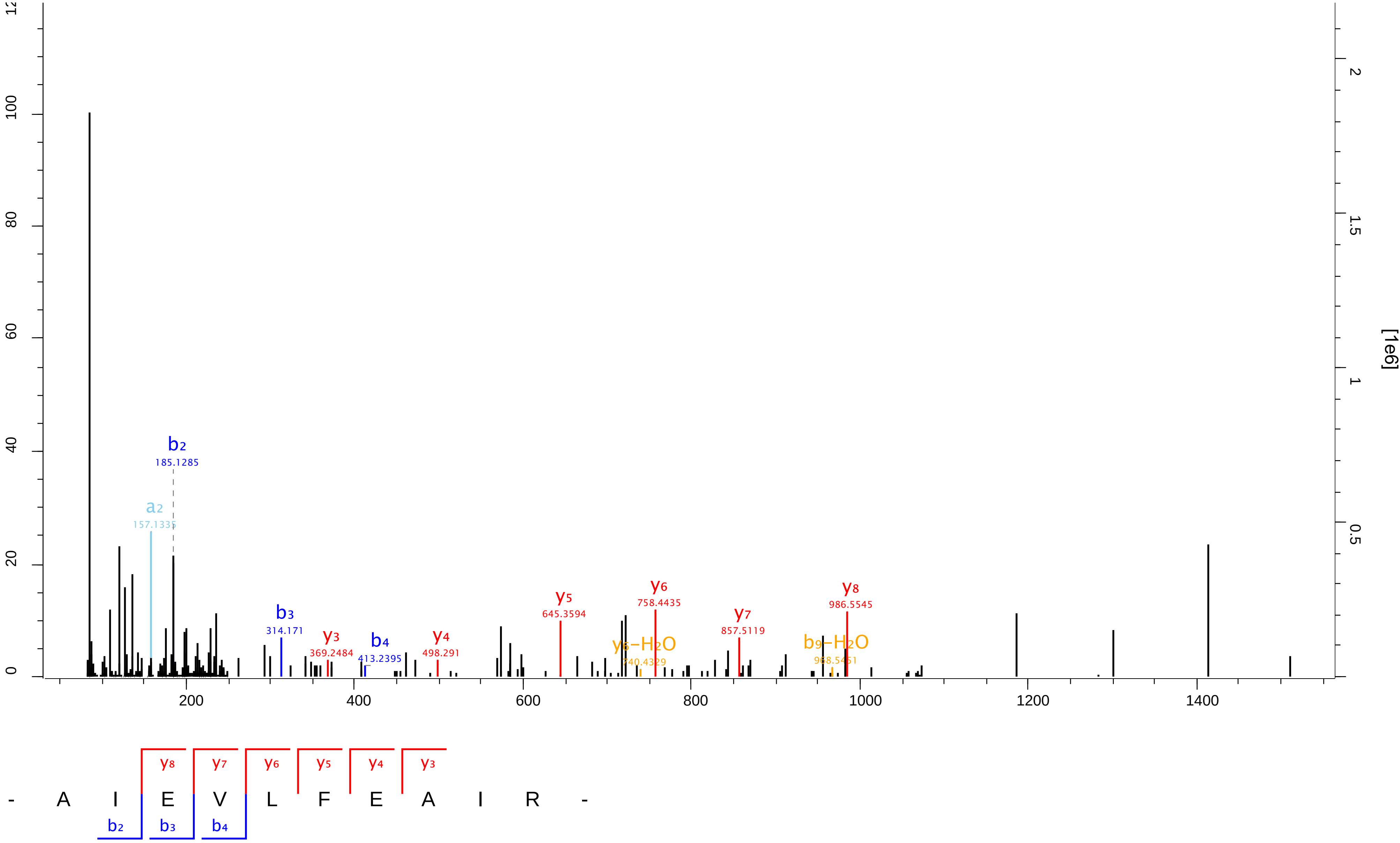

Raw file	Scan	Method	Score	m/z
QEplus003093	14462	FTMS; HCD	107.98	602.34

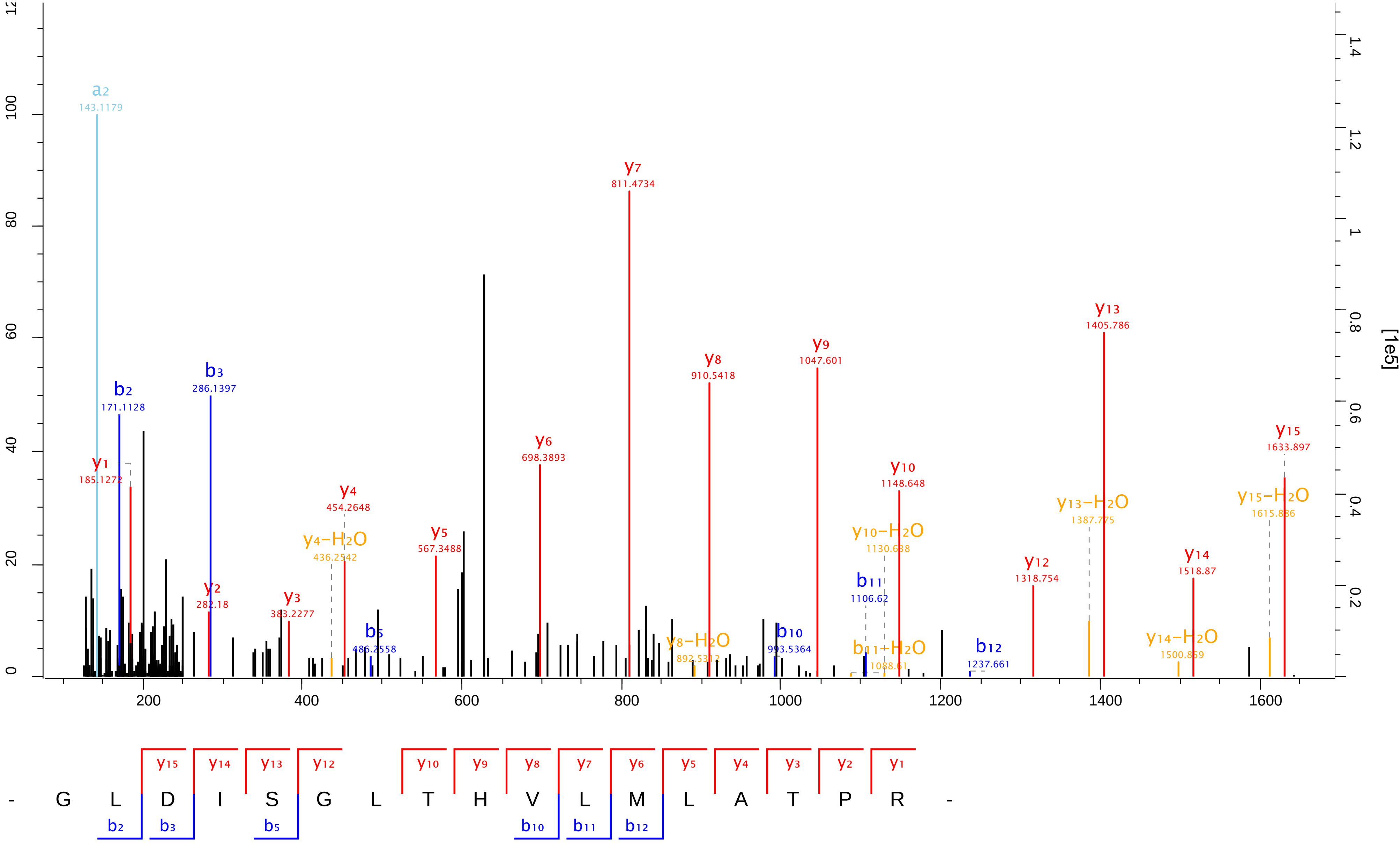

Raw file	Scan	Method	Score	m/z
QEplus003093	14507	FTMS; HCD	80.75	620.31

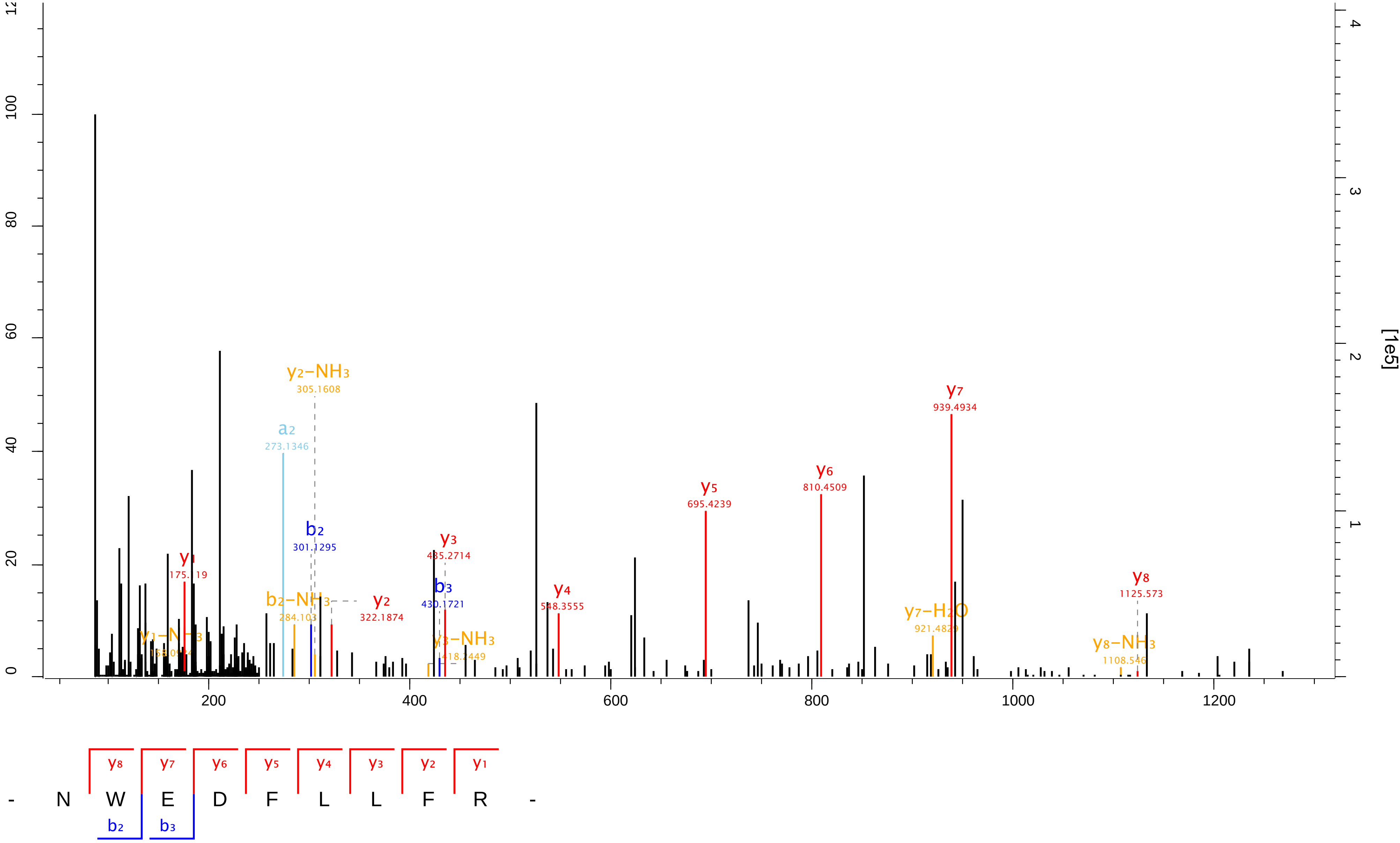

Raw file	Scan	Method	Score	m/z
QEplus003093	14613	FTMS; HCD	102.89	983

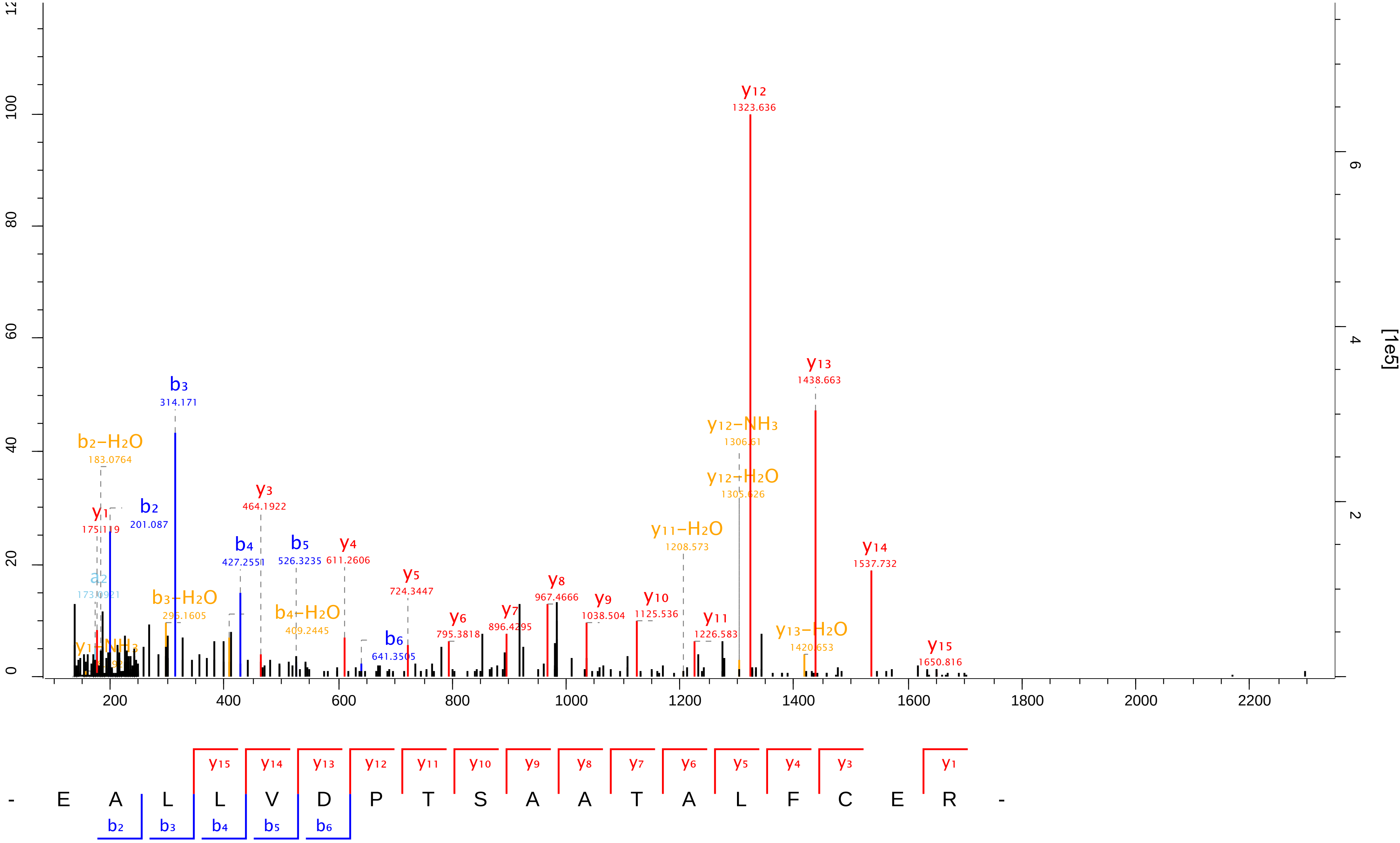

Raw file	Scan	Method	Score	m/z
QEplus003093	7921	FTMS; HCD	62.47	599.84

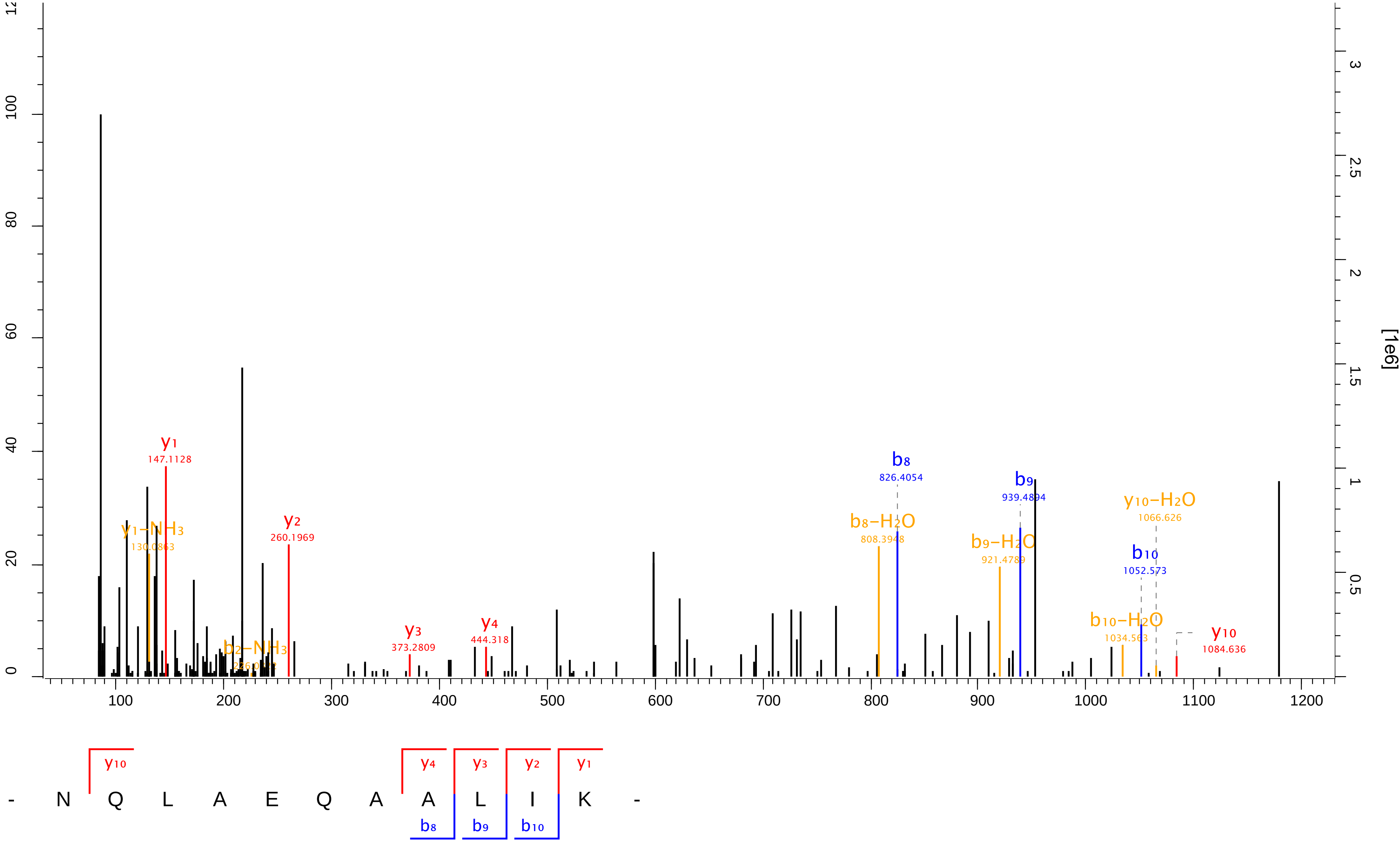

Raw file	Scan	Method	Score	m/z
QEplus003095	14248	FTMS; HCD	68.11	653.89

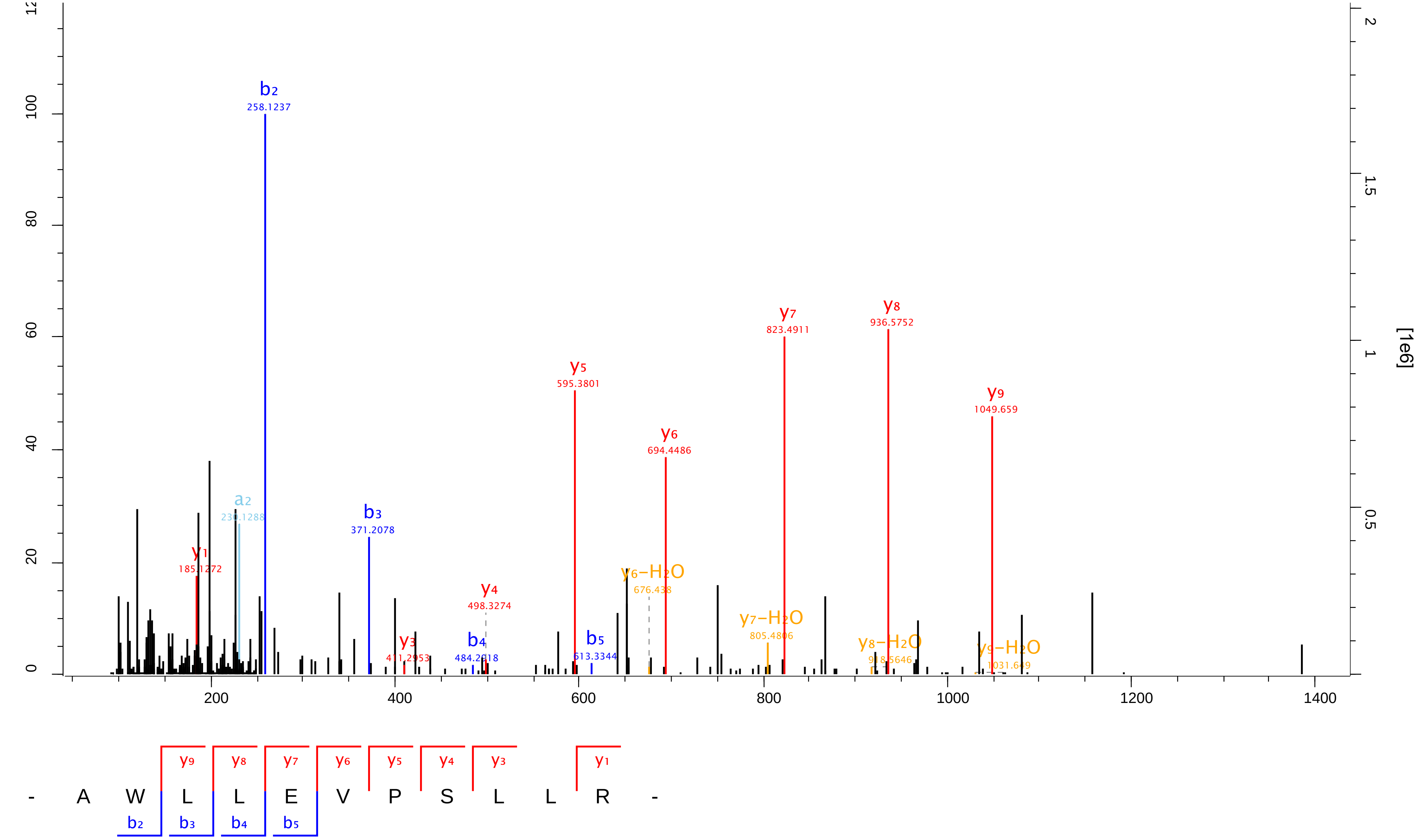

Raw file	Scan	Method	Score	m/z
QEplus003096	11206	FTMS; HCD	85.81	523.82

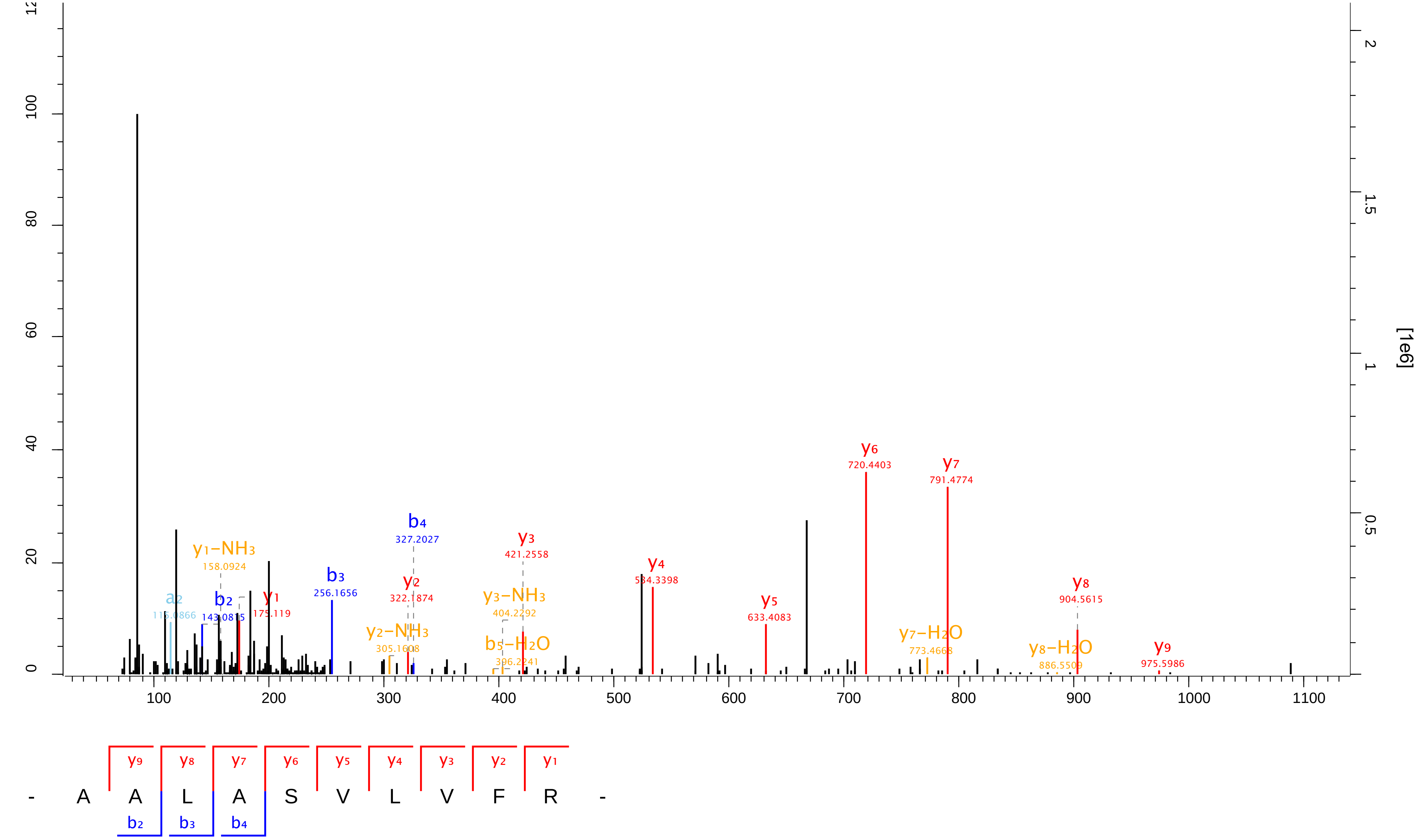

Raw file	Scan	Method	Score	m/z
QEplus003096	11263	FTMS; HCD	117.93	463.31

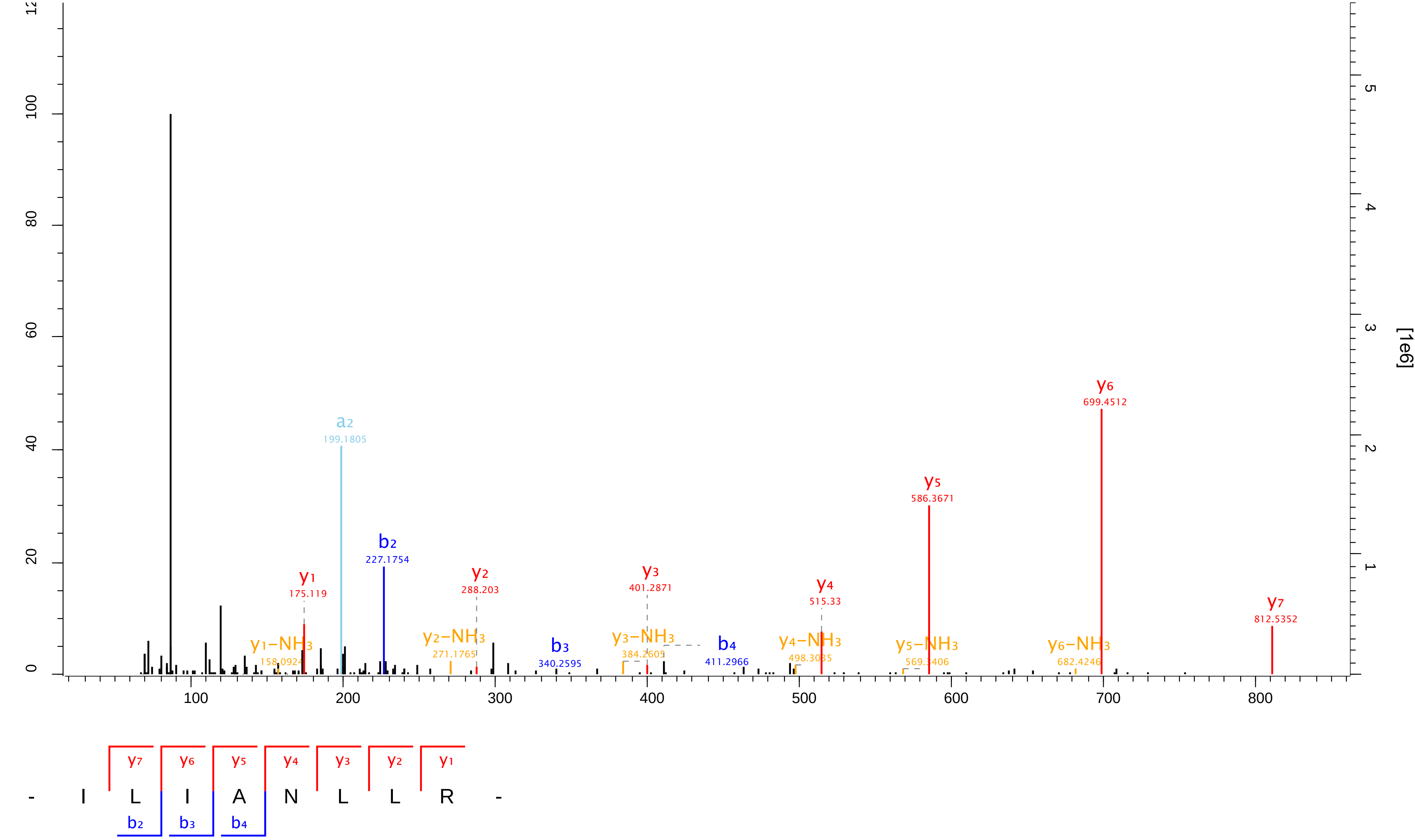

Raw file	Scan	Method	Score	m/z
QEplus003096	12404	FTMS; HCD	62.46	566.84

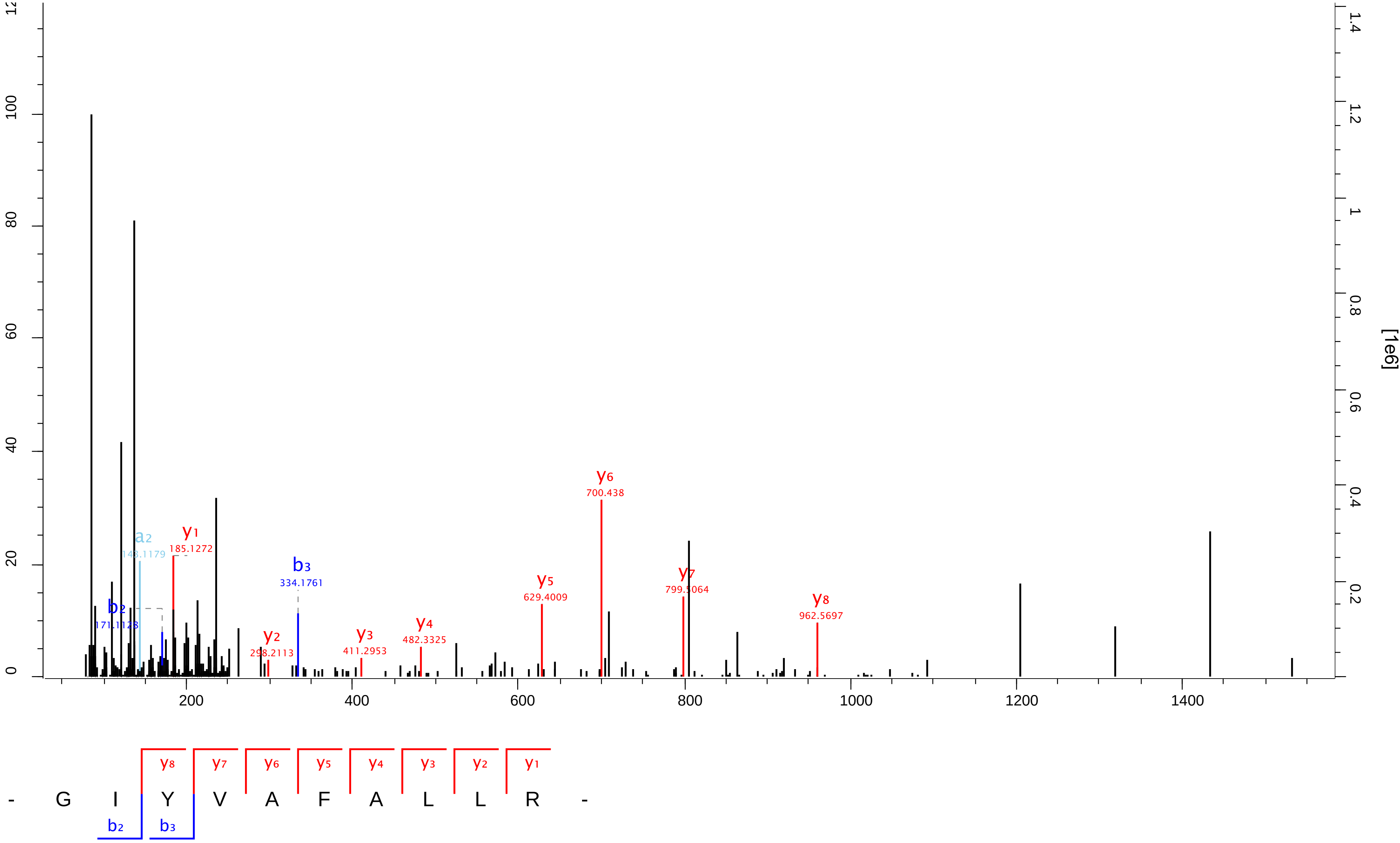

Raw file	Scan	Method	Score	m/z
QEplus003096	13189	FTMS; HCD	128.07	904.5

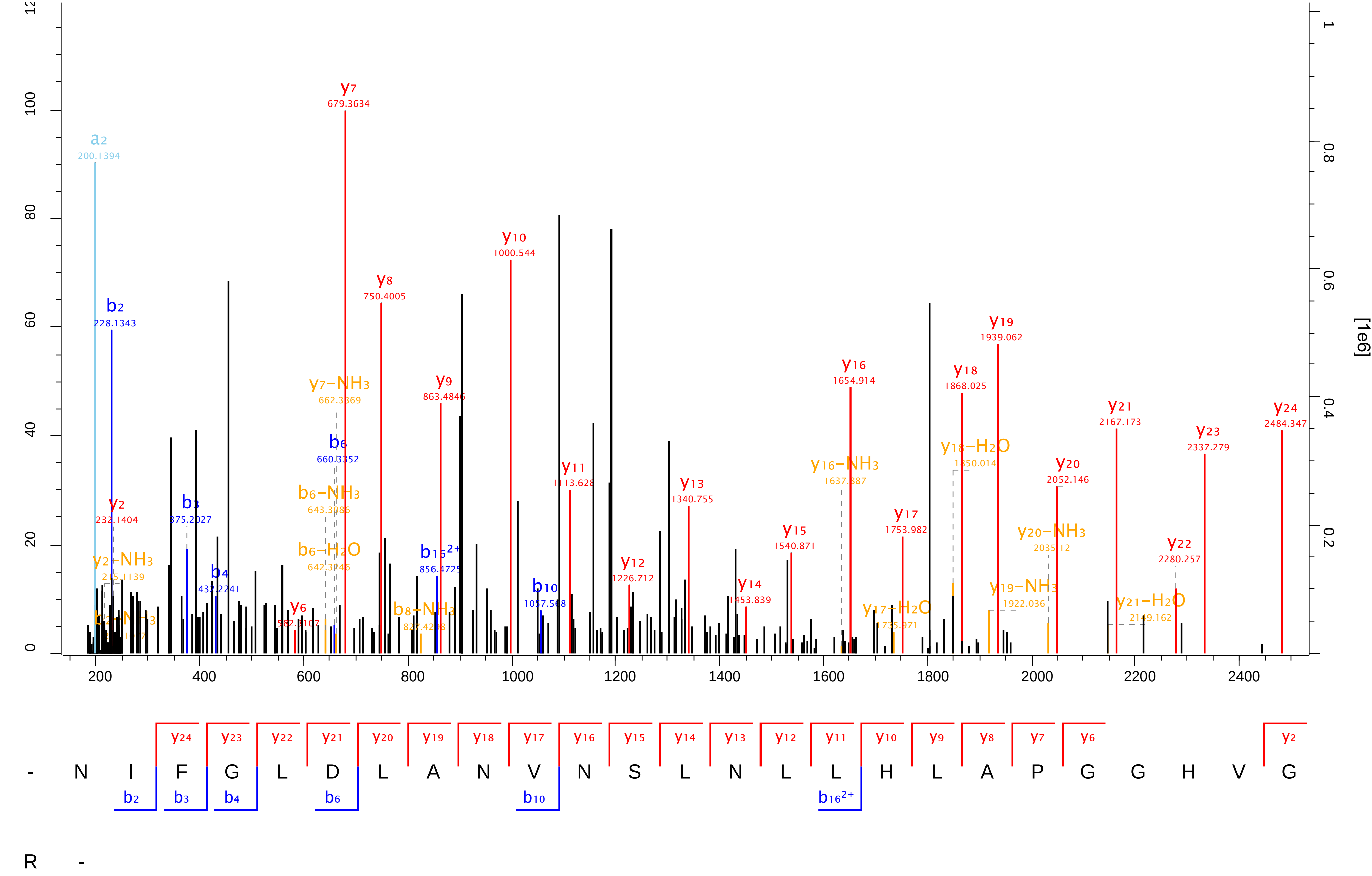

Raw file	Scan	Method	Score	m/z
QEplus003096	14896	FTMS; HCD	111.34	921.99

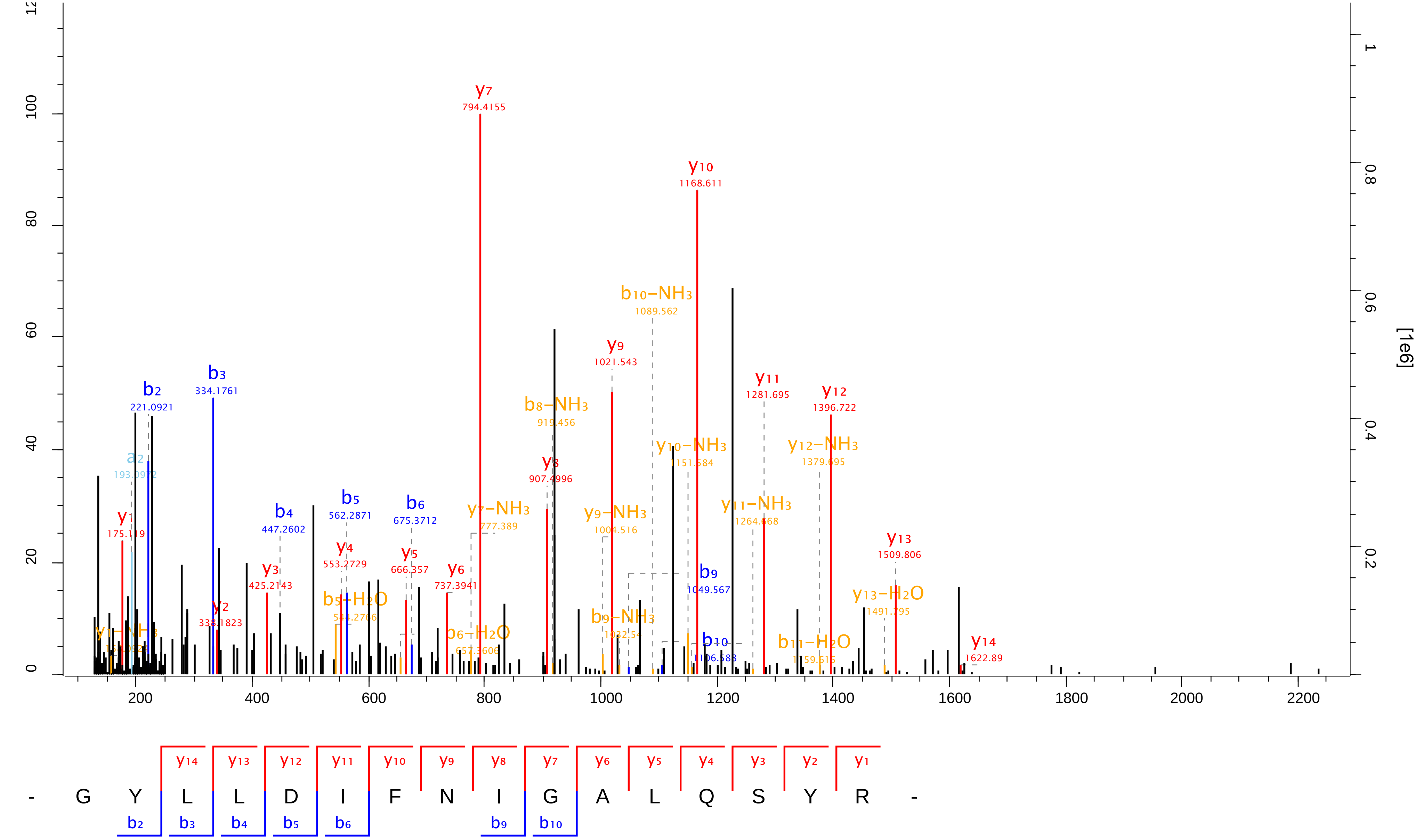

Raw file	Scan	Method	Score	m/z
QEplus003096	15506	FTMS; HCD	113.33	989.09

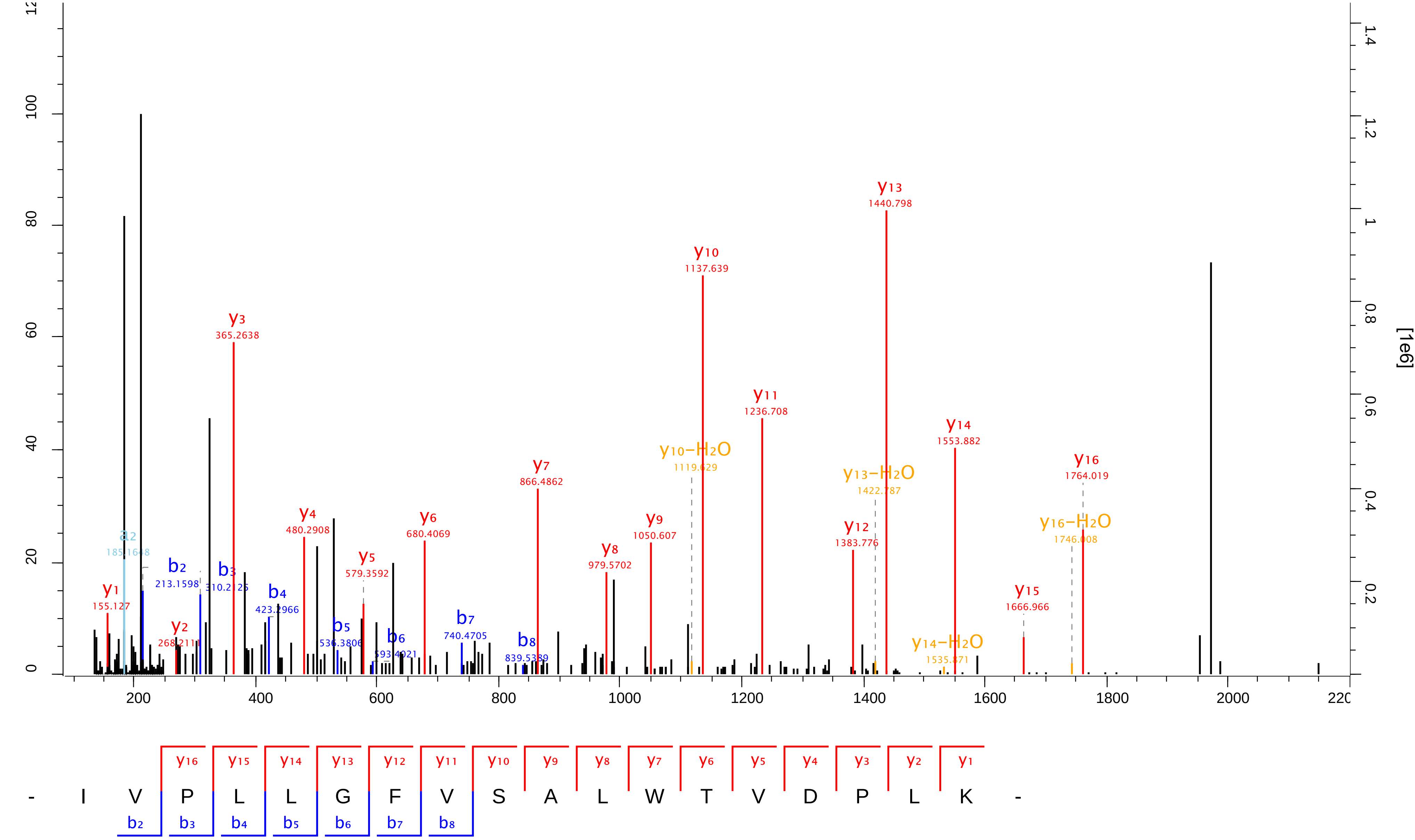

Raw file	Scan	Method	Score	m/z
QEplus003096	15739	FTMS; HCD	59.23	715.42

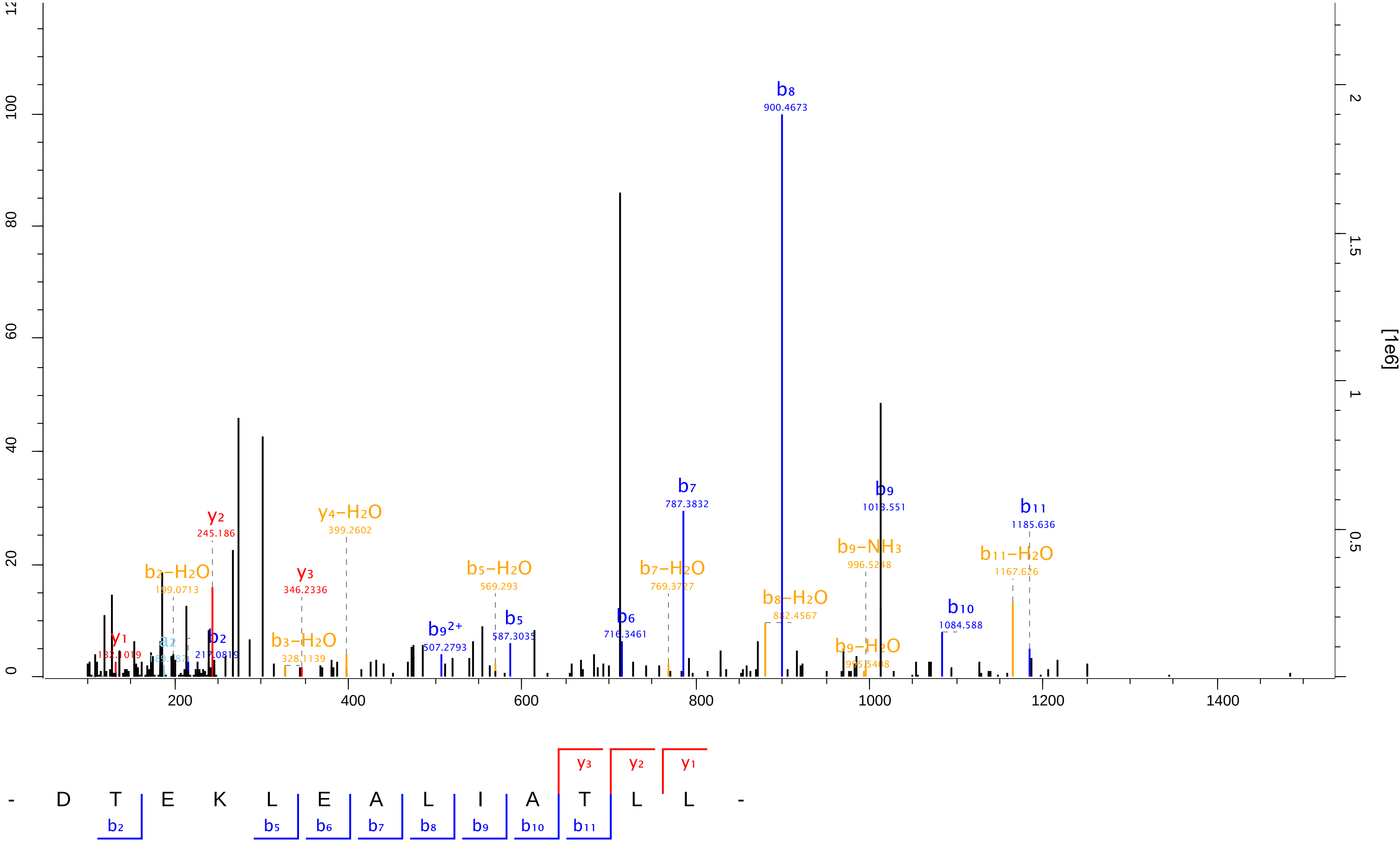

Raw file	Scan	Method	Score	m/z
QEplus003097	11436	FTMS; HCD	66.06	577.82

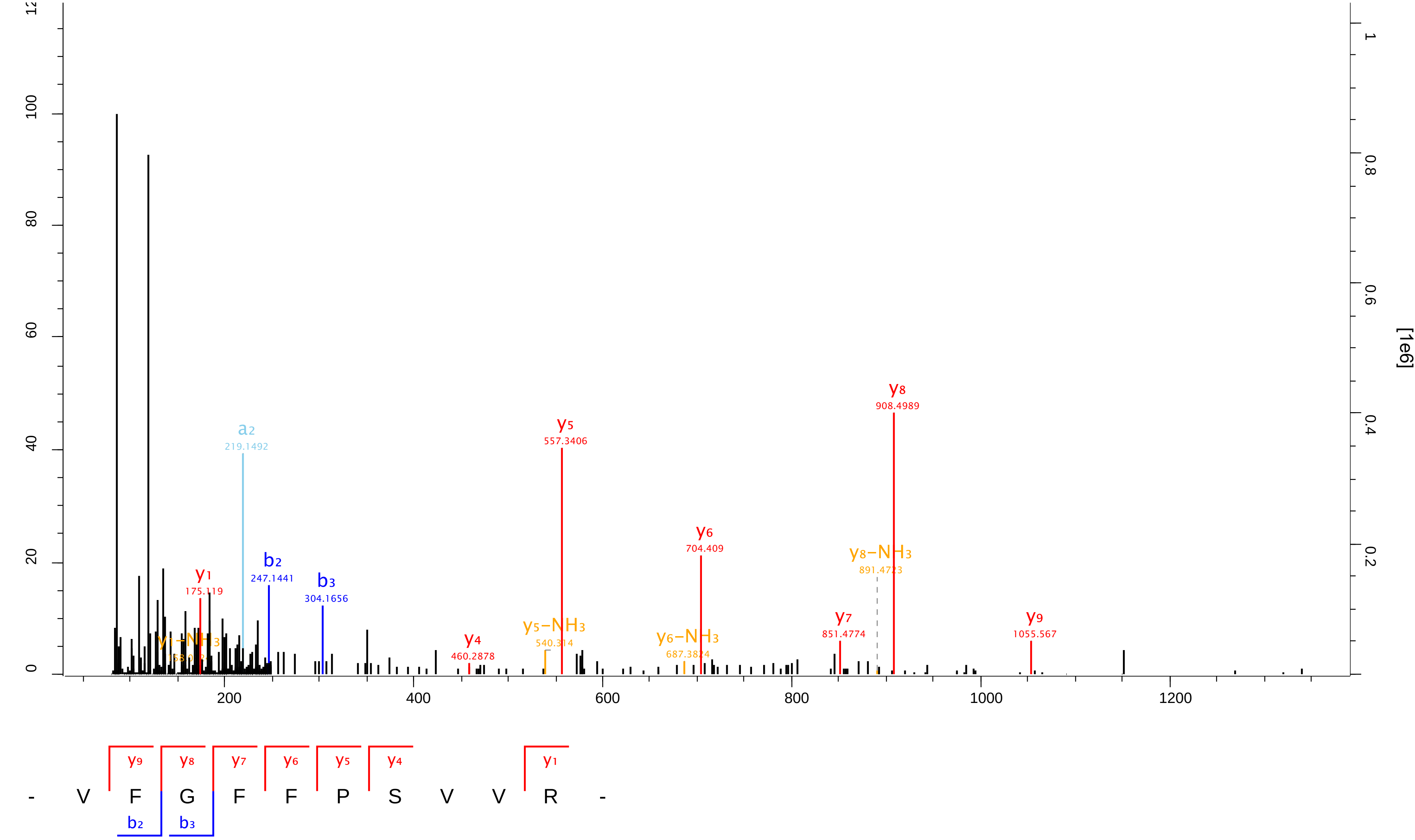

Raw file	Scan	Method	Score	m/z
QEplus003097	11706	FTMS; HCD	125.53	760.93

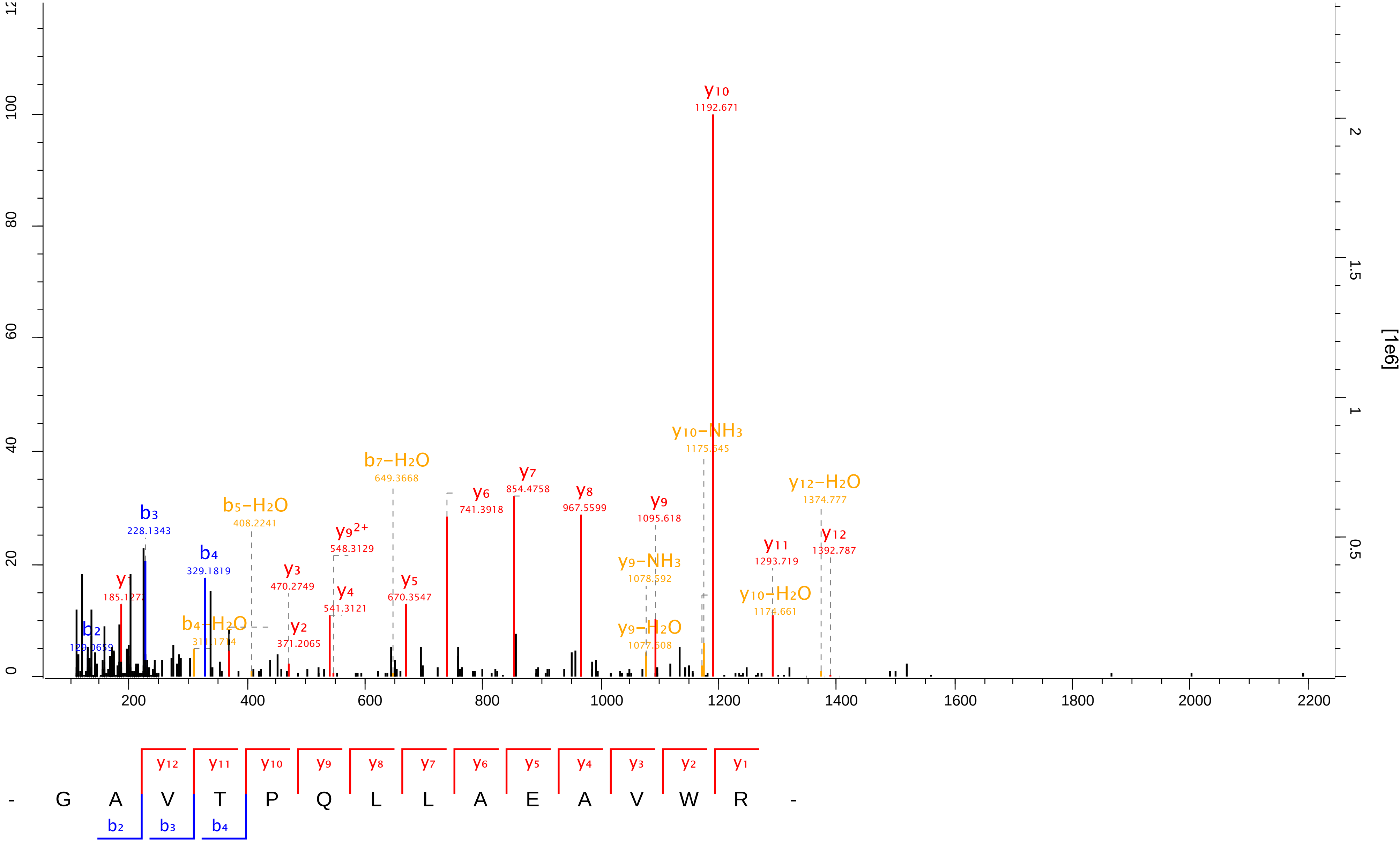

Raw file	Scan	Method	Score	m/z
QEplus003097	12084	FTMS; HCD	117.08	760.96

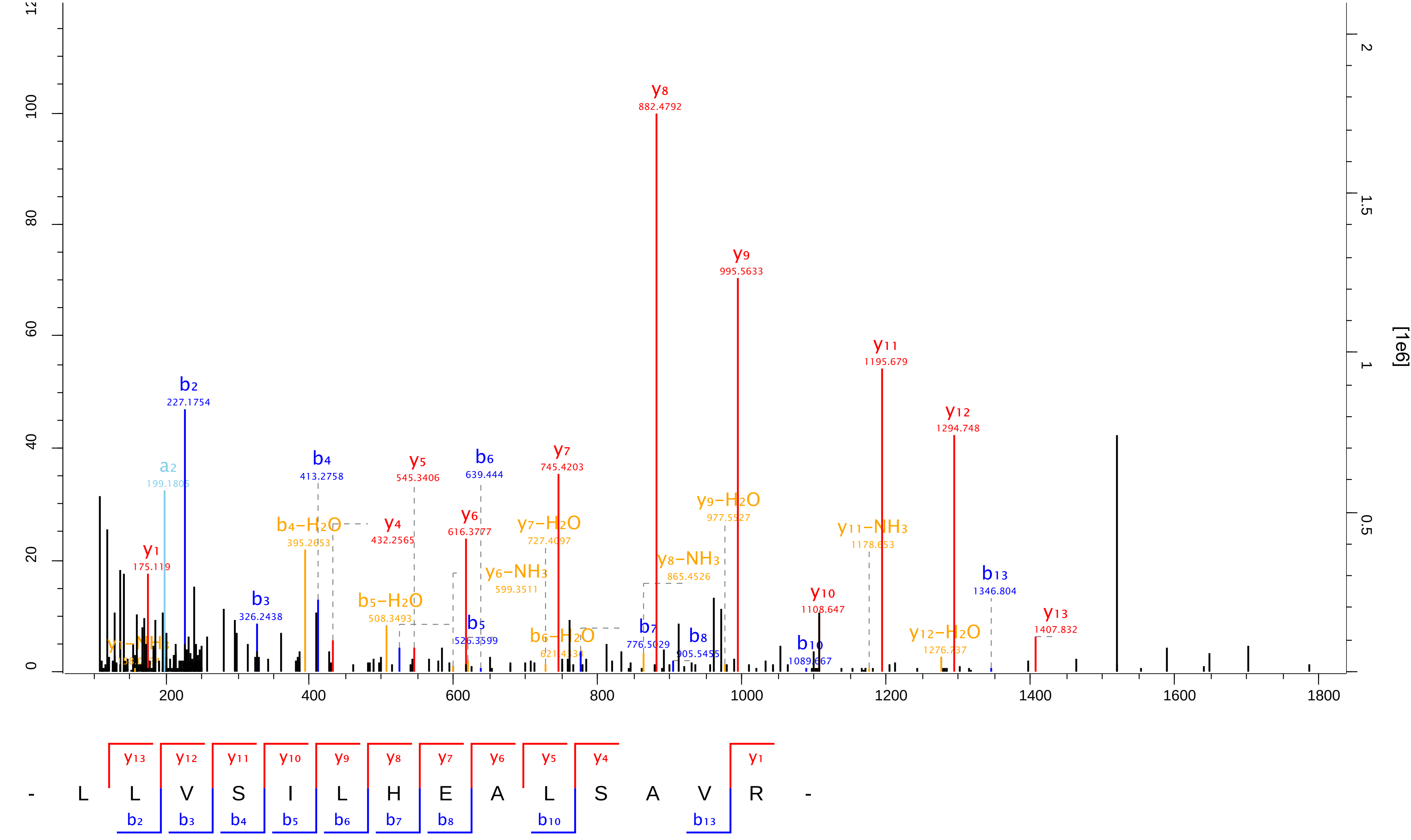

Raw file	Scan	Method	Score	m/z
QEplus003097	12697	FTMS; HCD	49.62	869

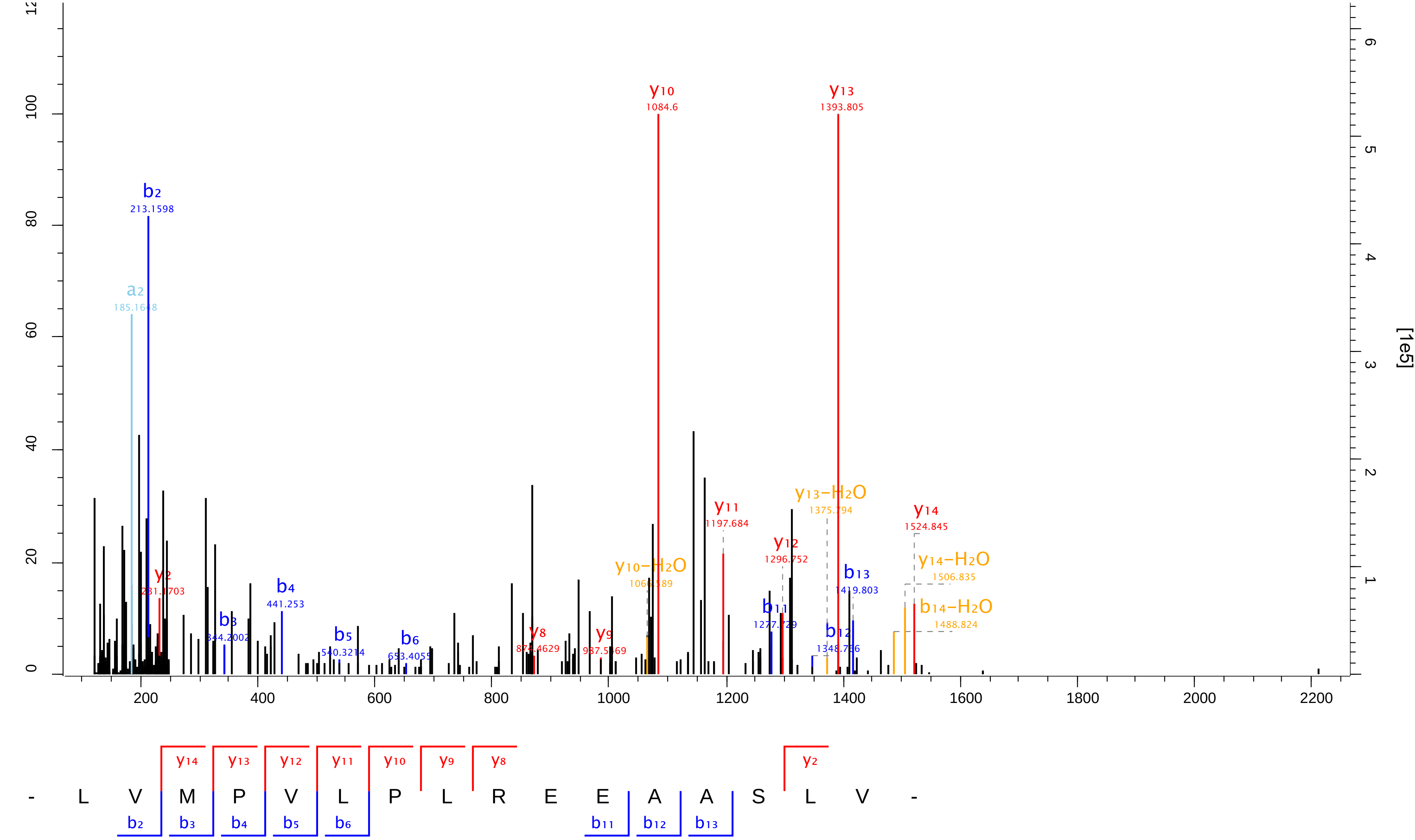

Raw file	Scan	Method	Score	m/z
QEplus003097	13116	FTMS; HCD	76.07	678.88

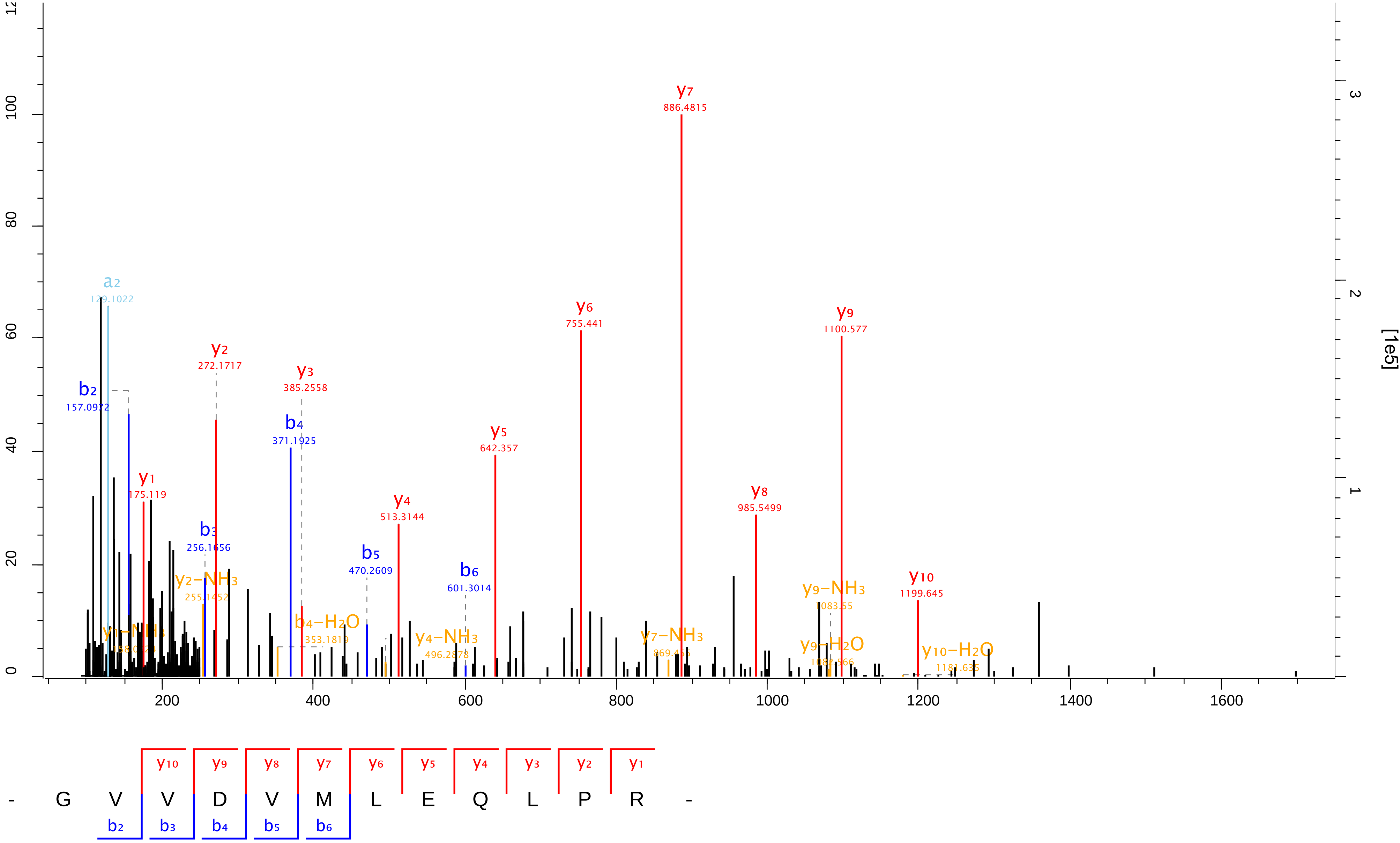

Raw file	Scan	Method	Score	m/z
QEplus003097	4506	FTMS; HCD	98.74	543.28

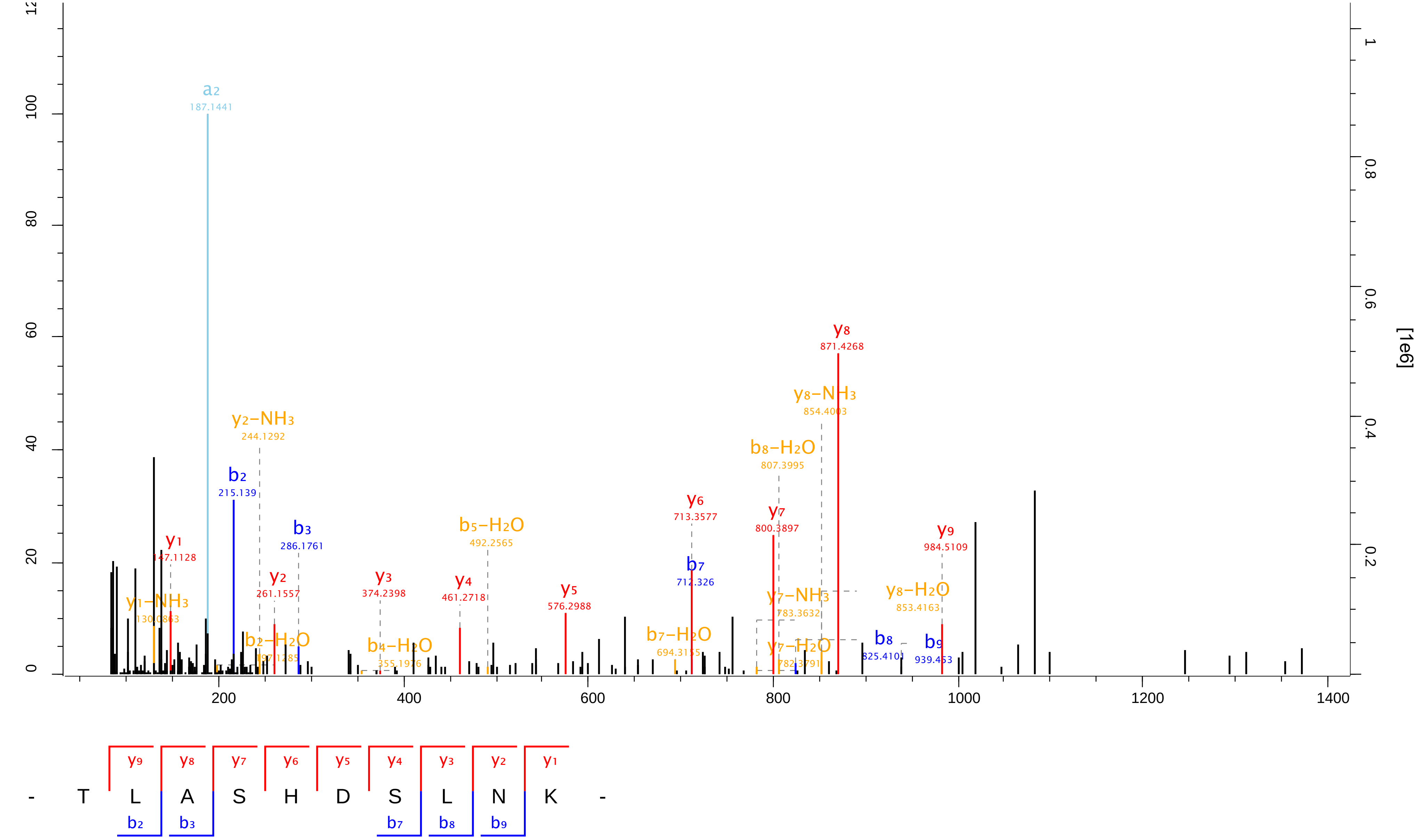

Raw file	Scan	Method	Score	m/z
QEplus003097	9740	FTMS; HCD	47.71	596.32

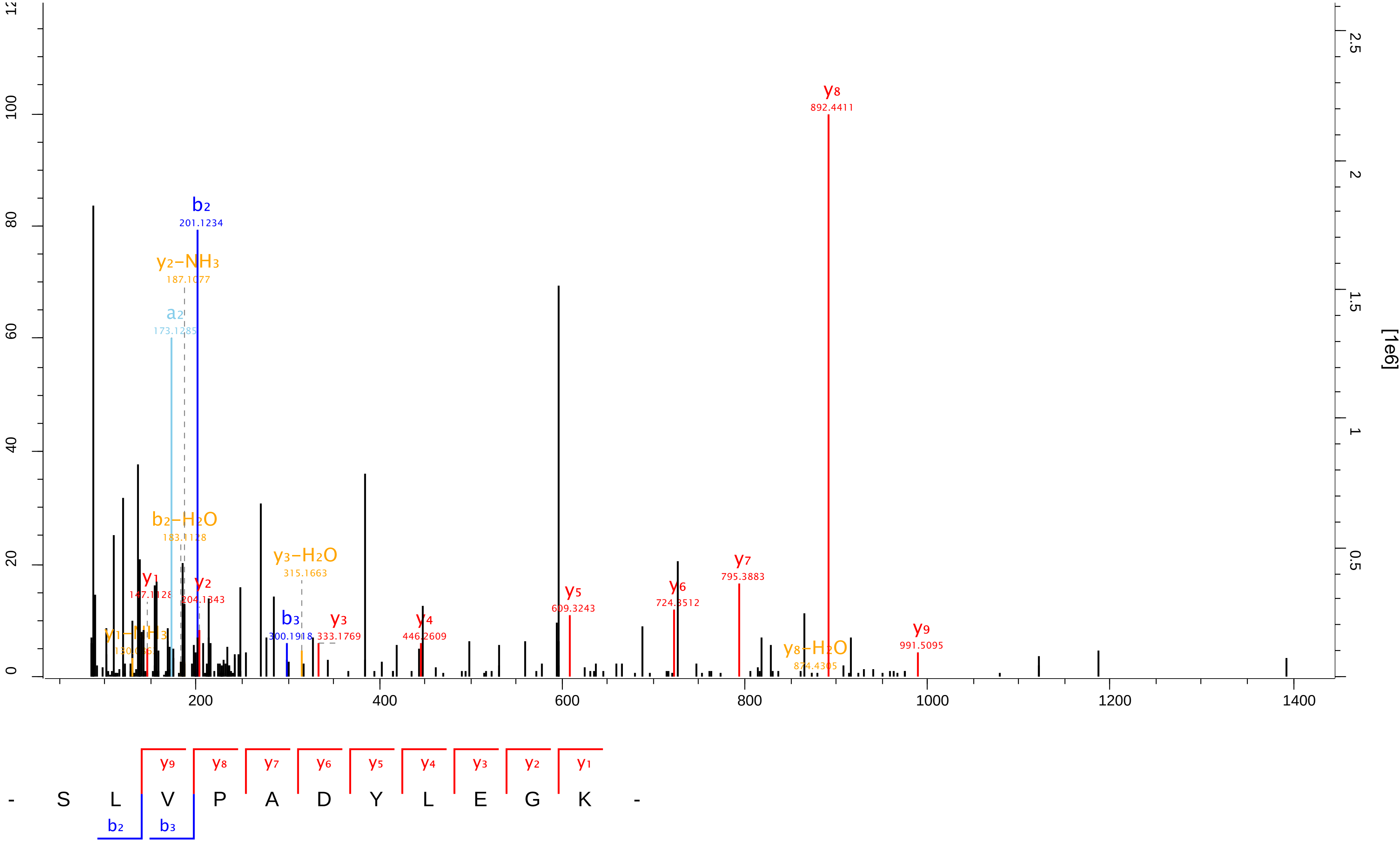

Raw file	Scan	Method	Score	m/z
QEplus003098	10721	FTMS; HCD	117.39	541.81

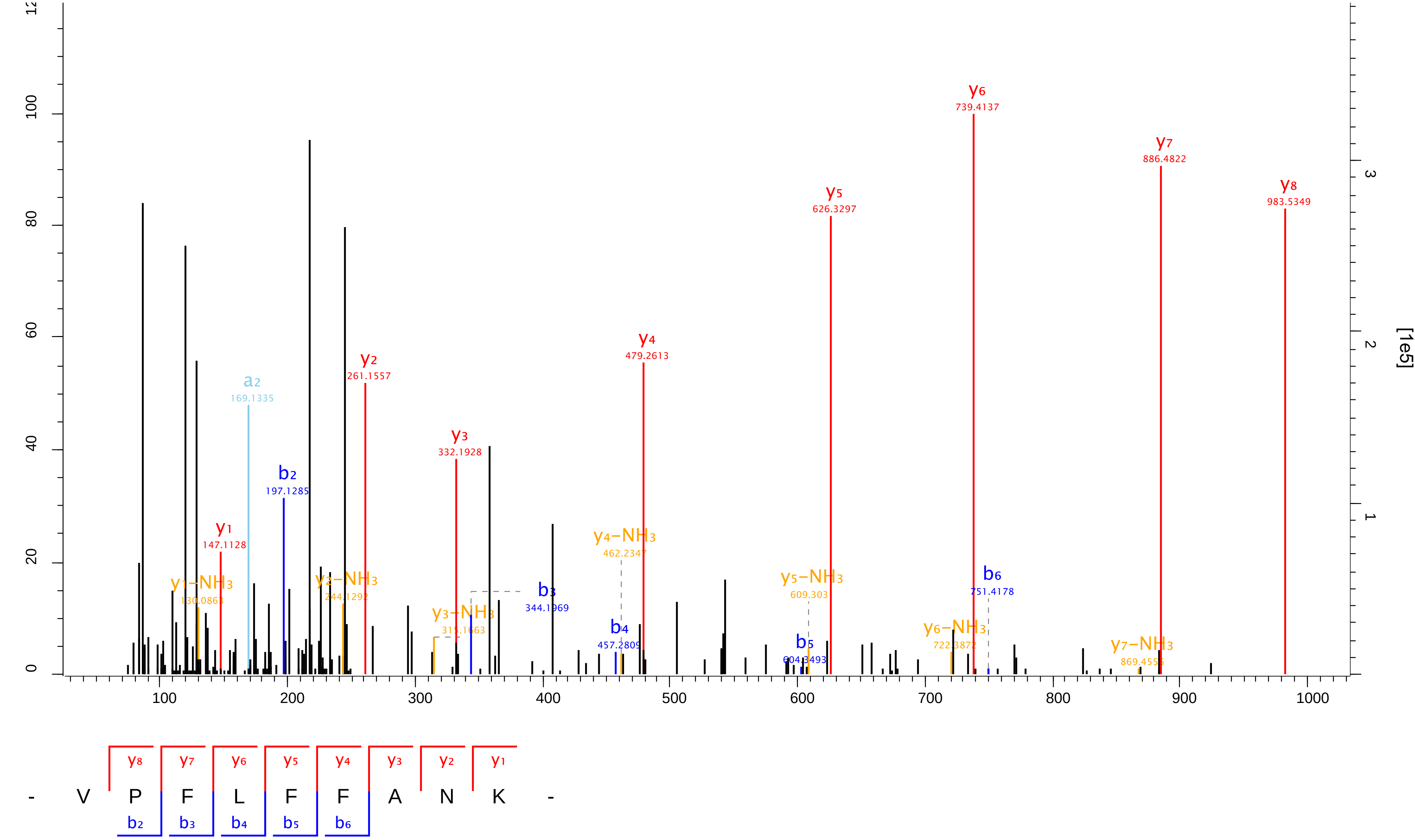

Raw file	Scan	Method	Score	m/z
QEplus003098	11034	FTMS; HCD	60.16	676.91

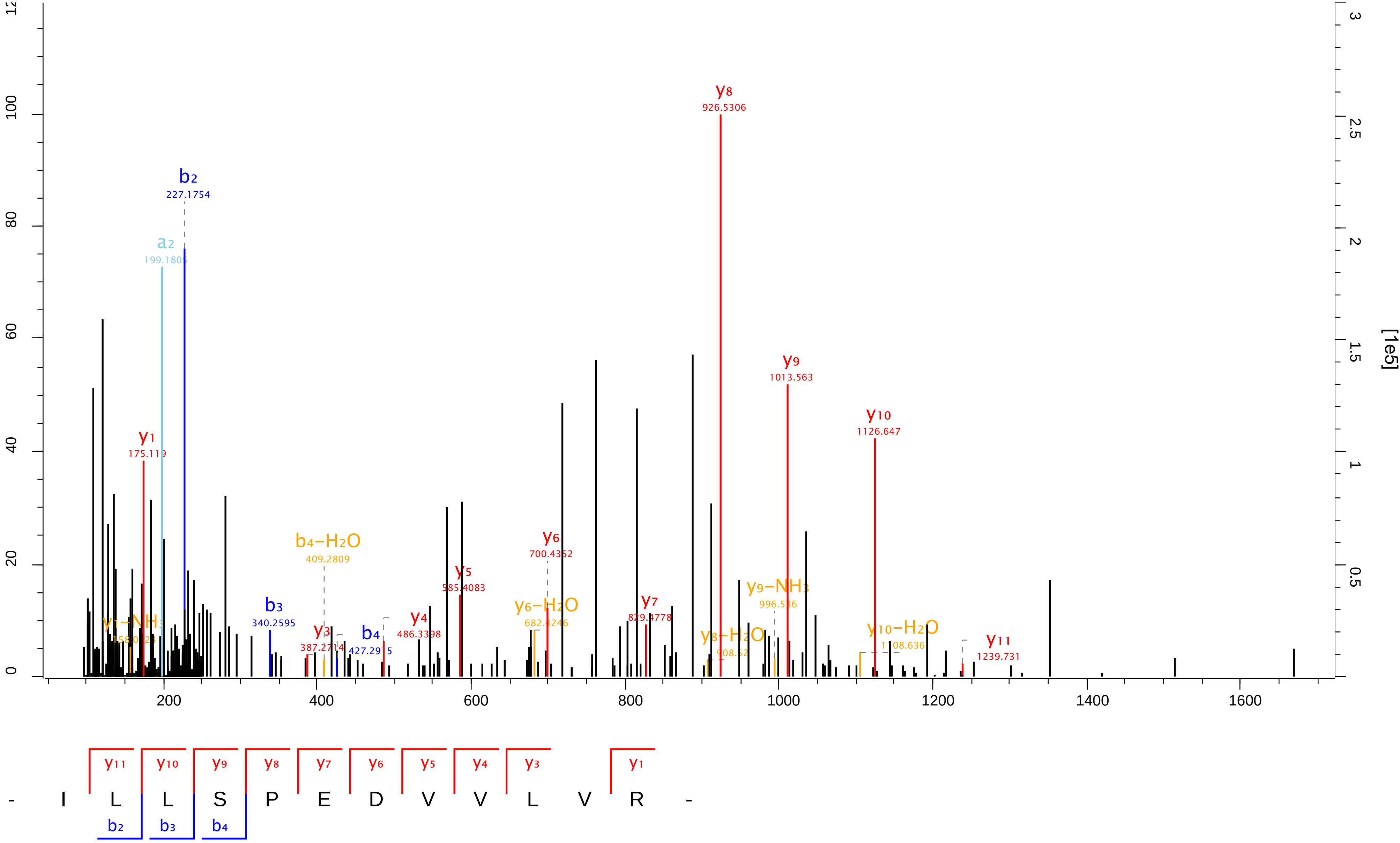

Raw file	Scan	Method	Score	m/z
QEplus003098	13086	FTMS; HCD	101.62	1137.6

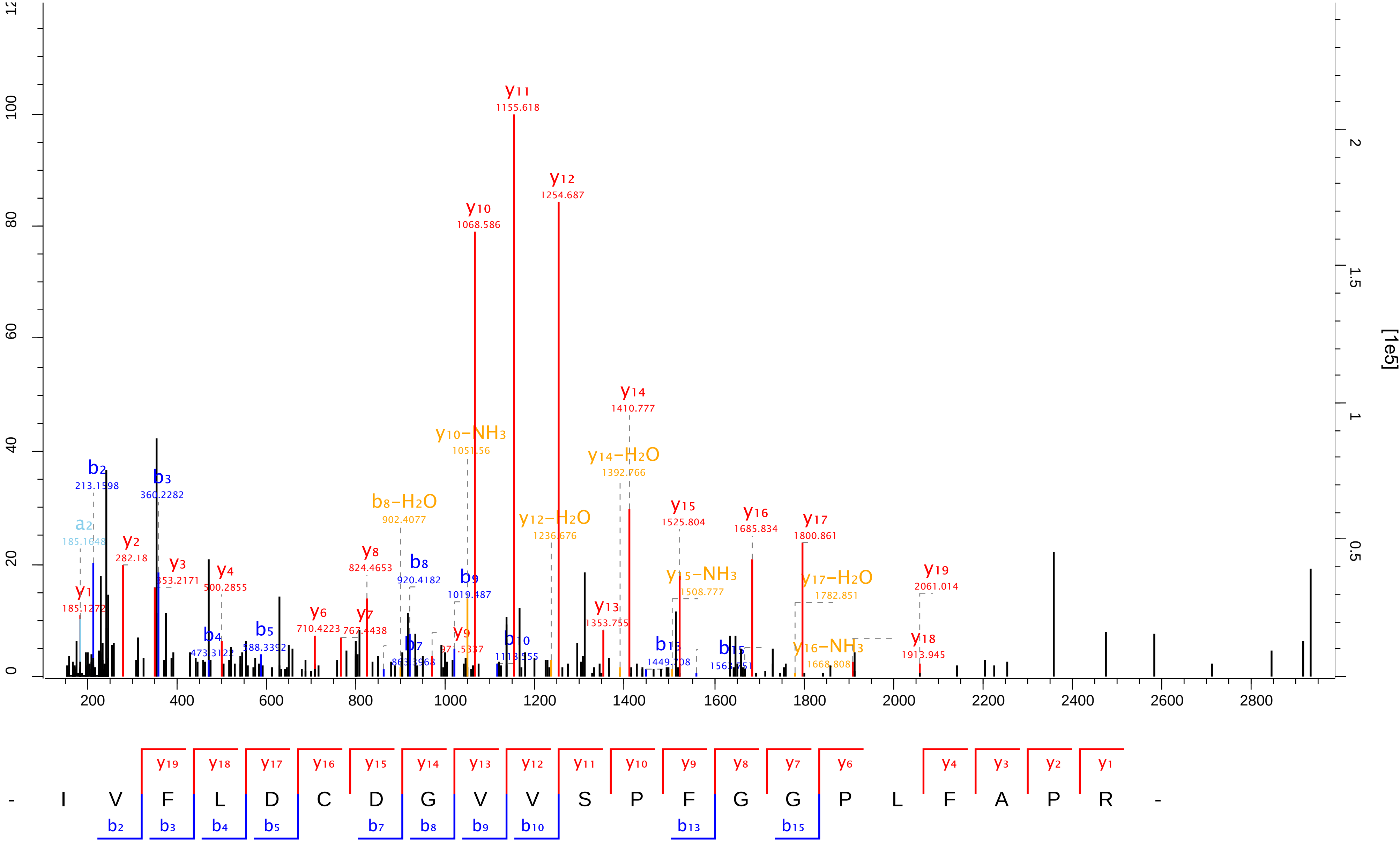

Raw file	Scan	Method	Score	m/z
QEplus003098	13171	FTMS; HCD	160.55	959.47

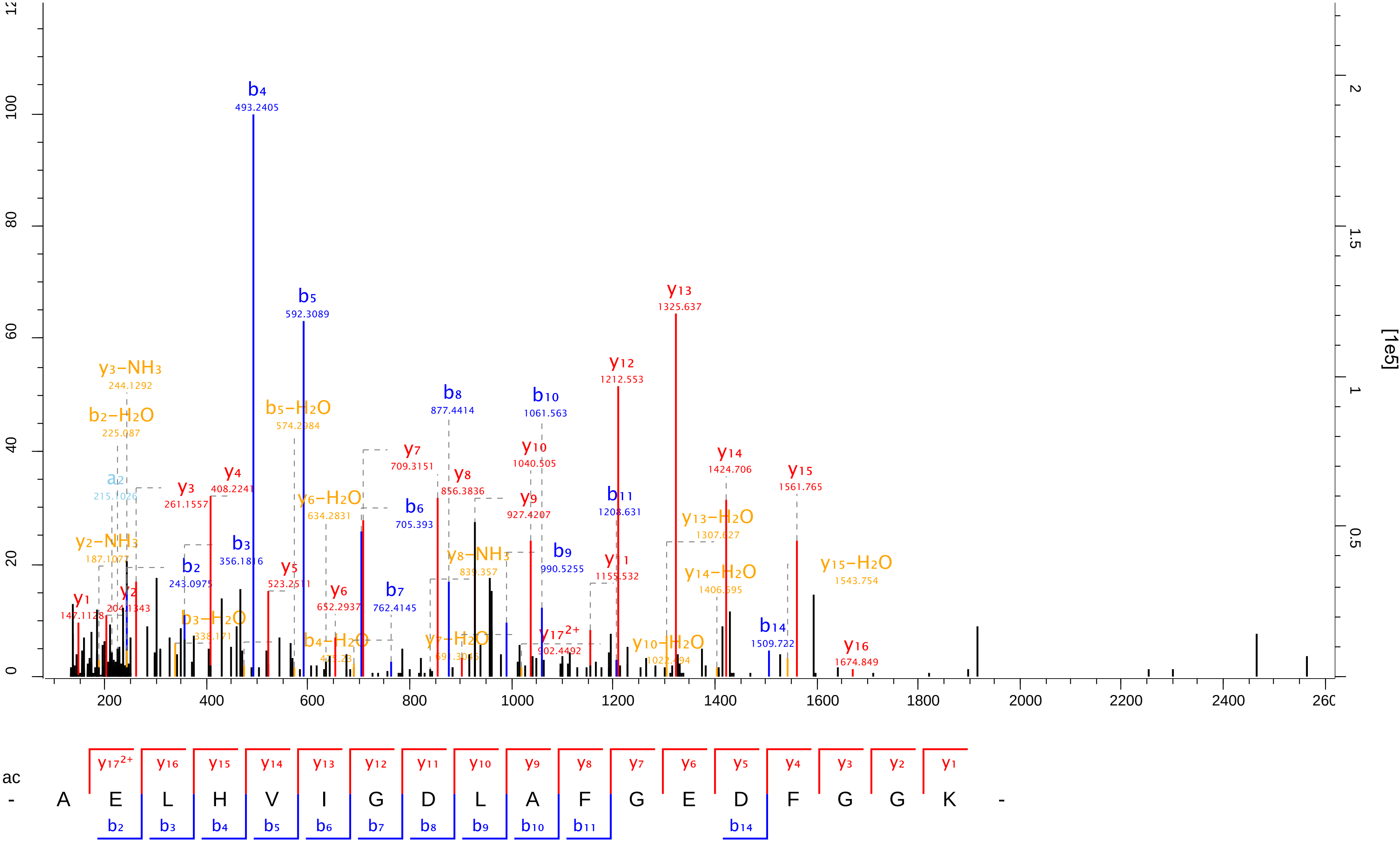

Raw file	Scan	Method	Score	m/z
QEplus003098	13173	FTMS; HCD	126.63	988.55

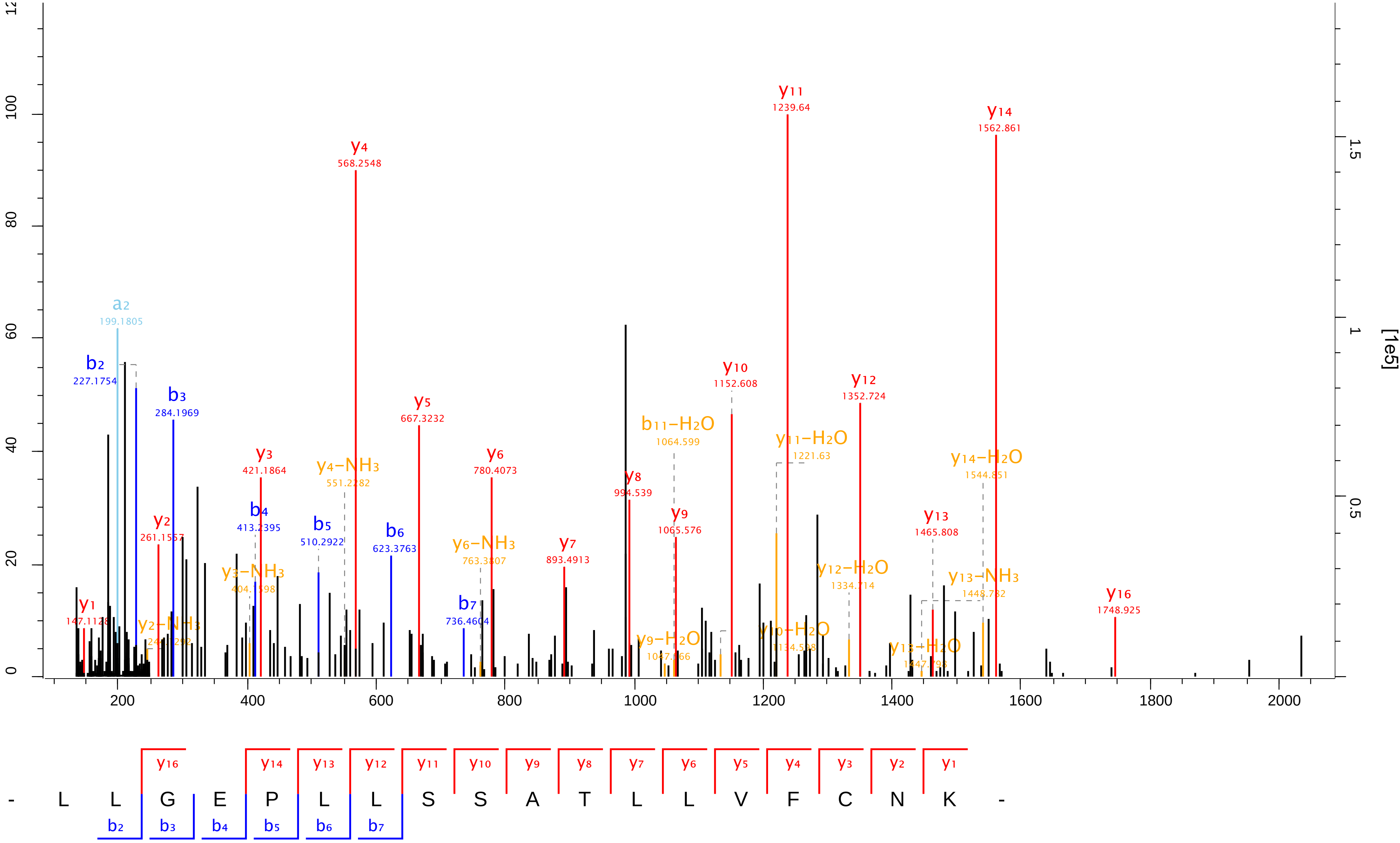

Raw file	Scan	Method	Score	m/z
QEplus003098	13276	FTMS; HCD	90.71	736.42

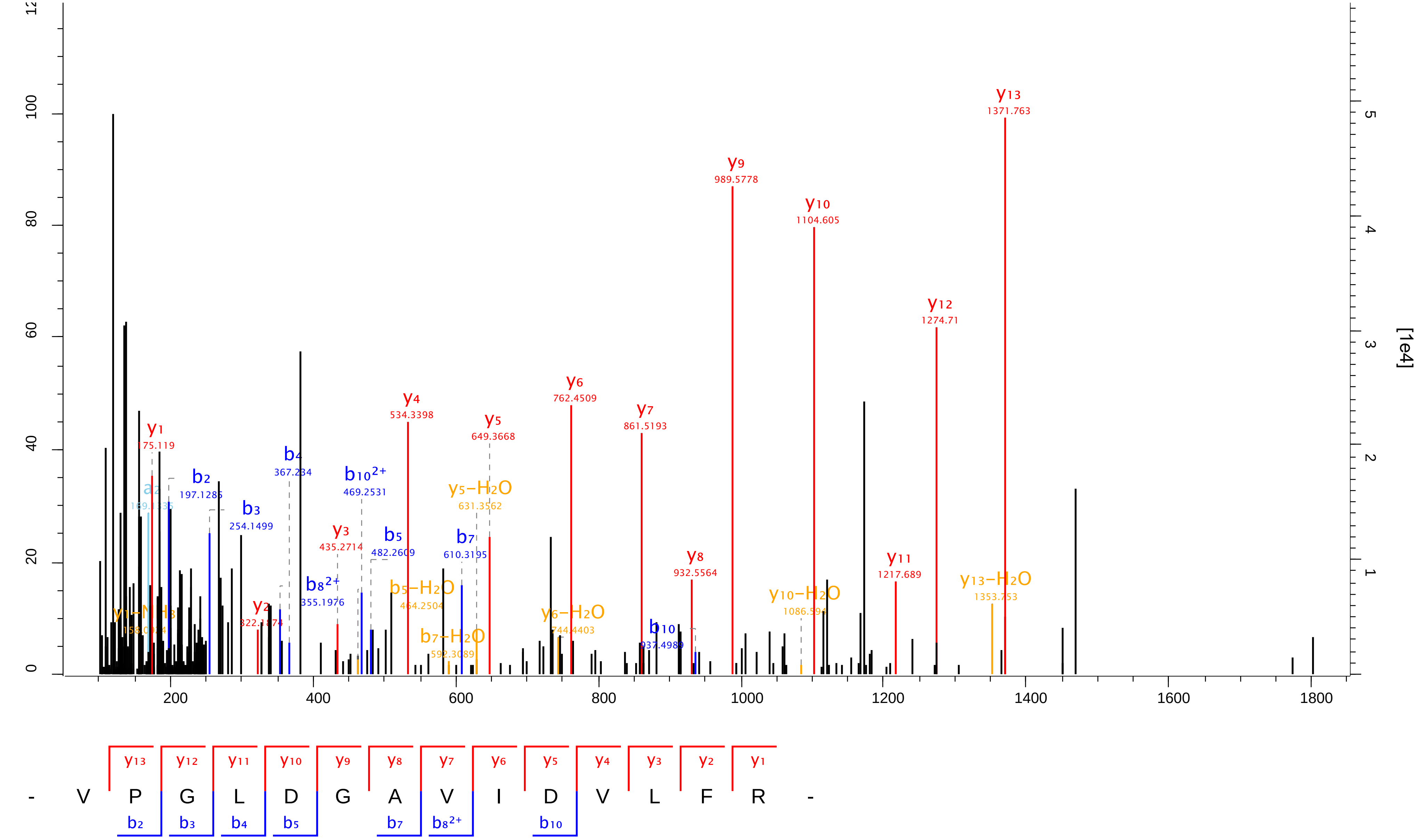

Raw file	Scan	Method	Score	m/z
QEplus003098	14306	FTMS; HCD	68.42	1089.57

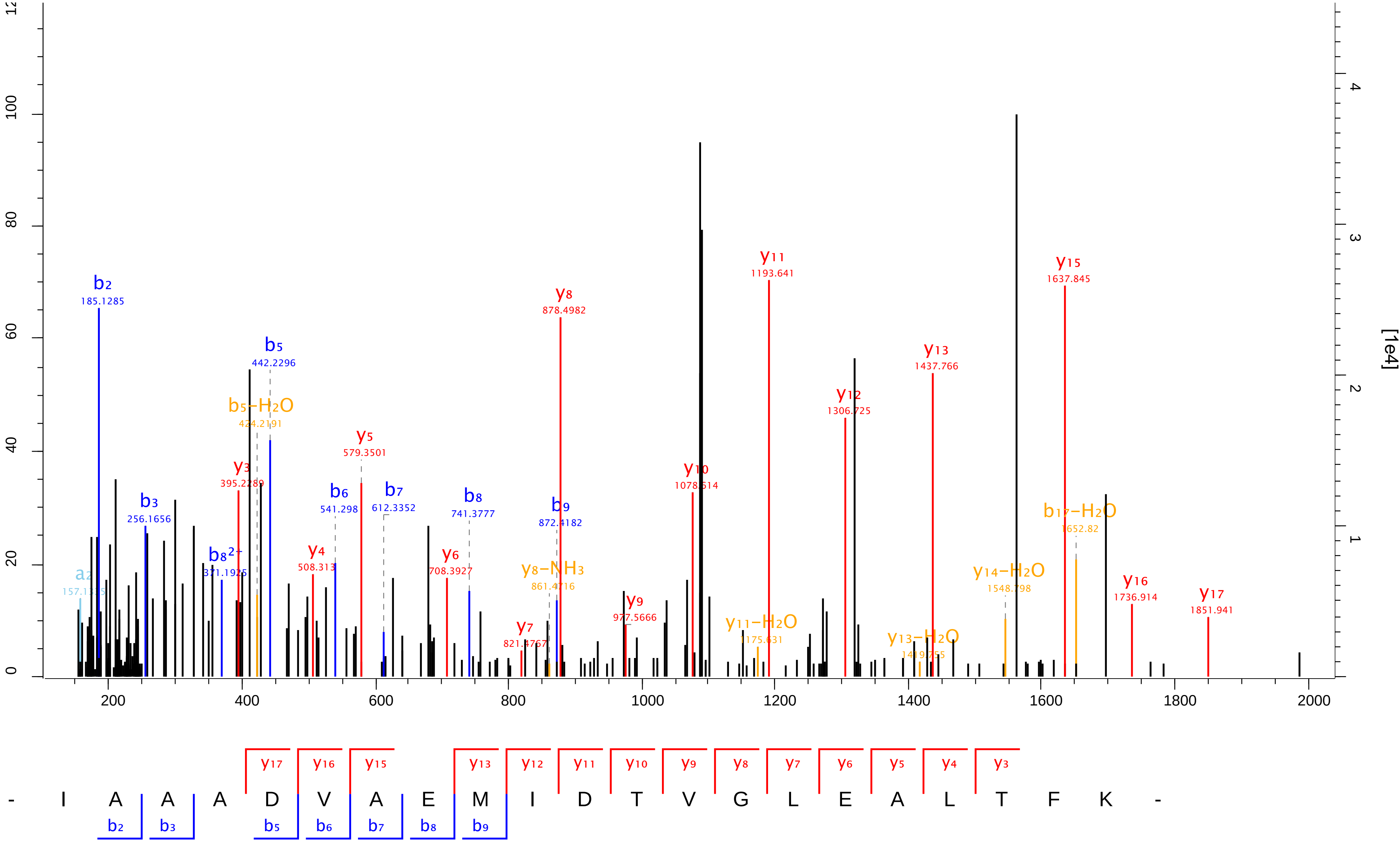

Raw file	Scan	Method	Score	m/z
QEplus003098	9198	FTMS; HCD	48.98	378.9

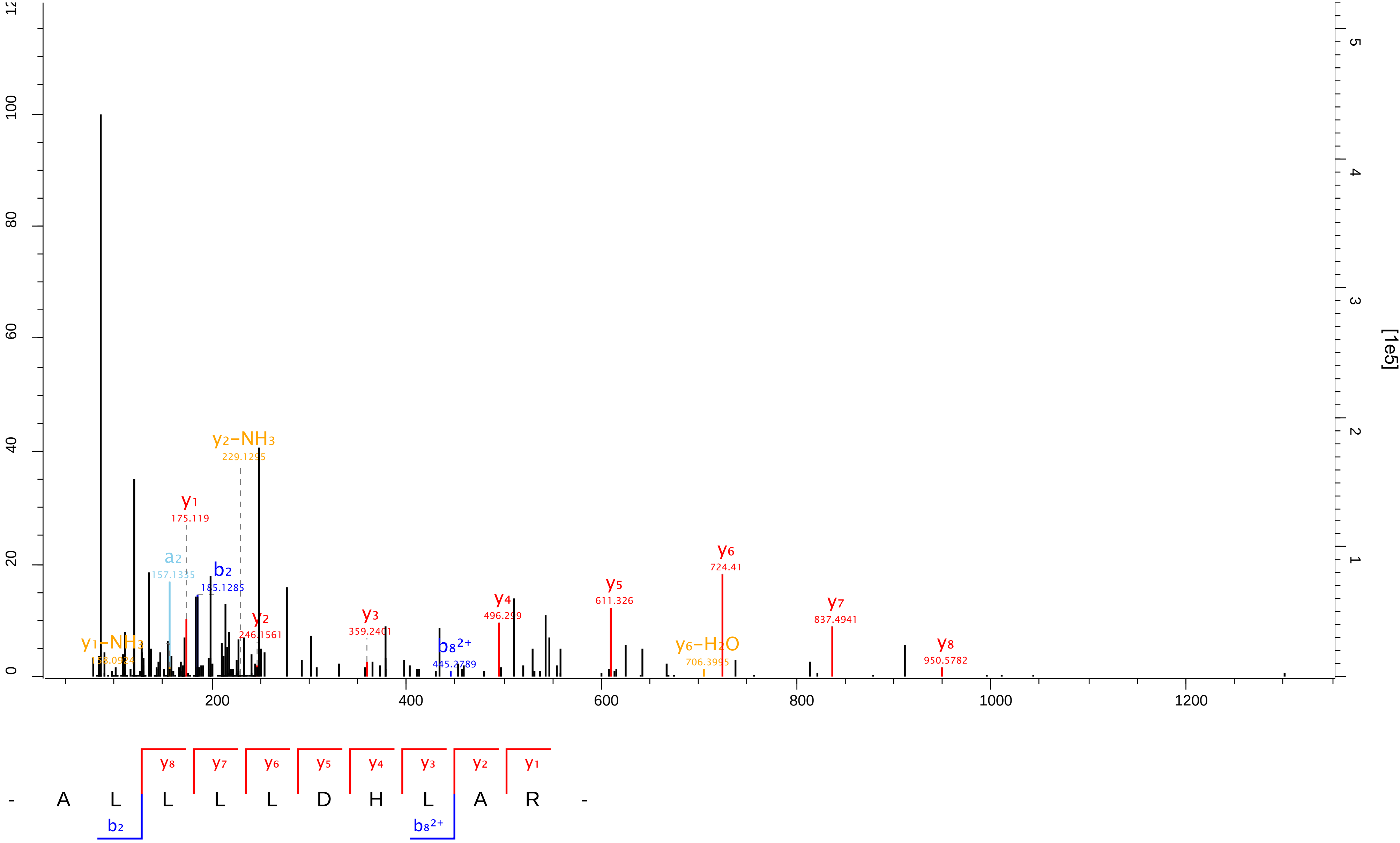

Raw file	Scan	Method	Score	m/z
QEplus003099	10225	FTMS; HCD	126.24	443.78

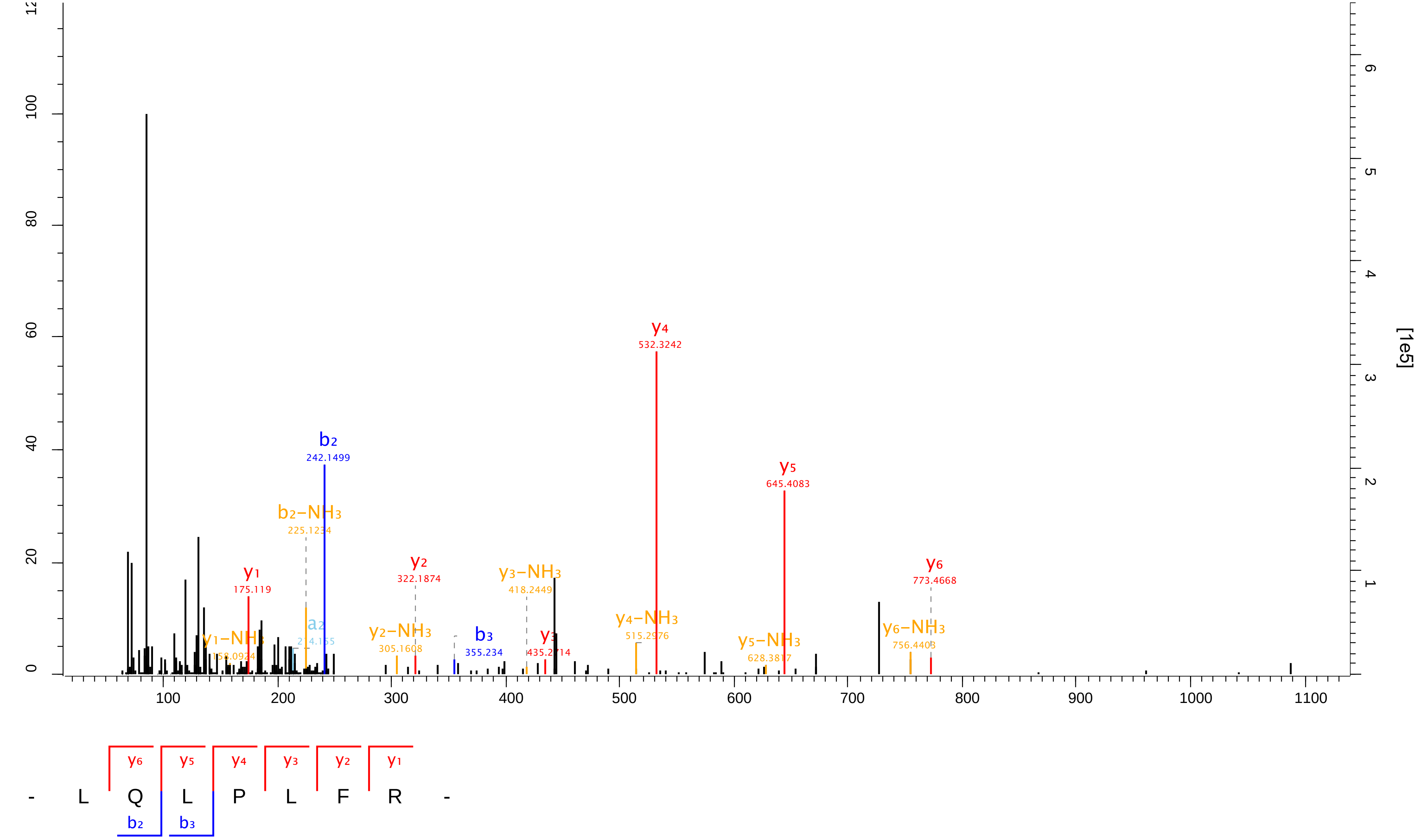

Raw file	Scan	Method	Score	m/z
QEplus003099	11539	FTMS; HCD	154.34	657.84

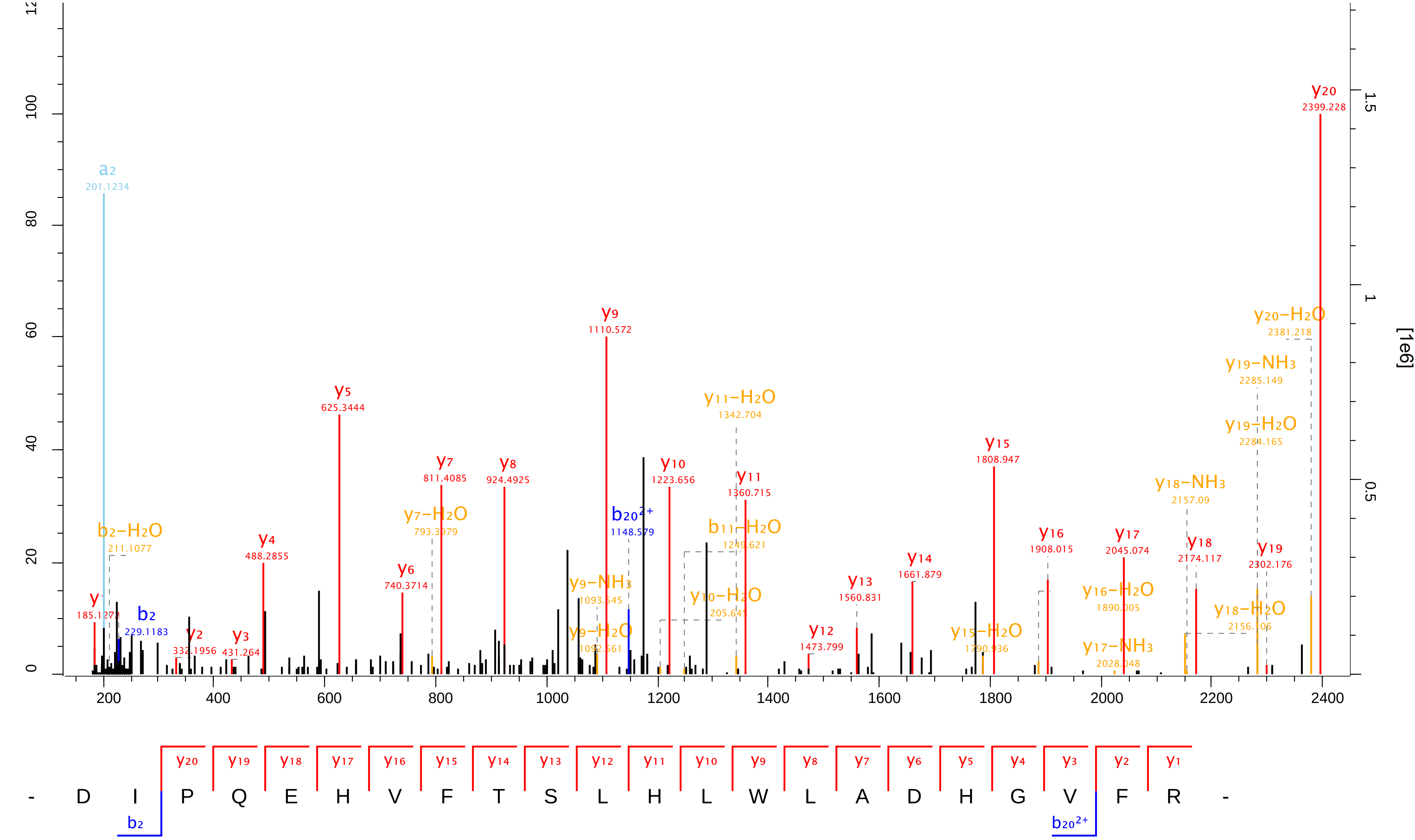

Raw file	Scan	Method	Score	m/z
QEplus003099	12182	FTMS; HCD	84.38	707.03

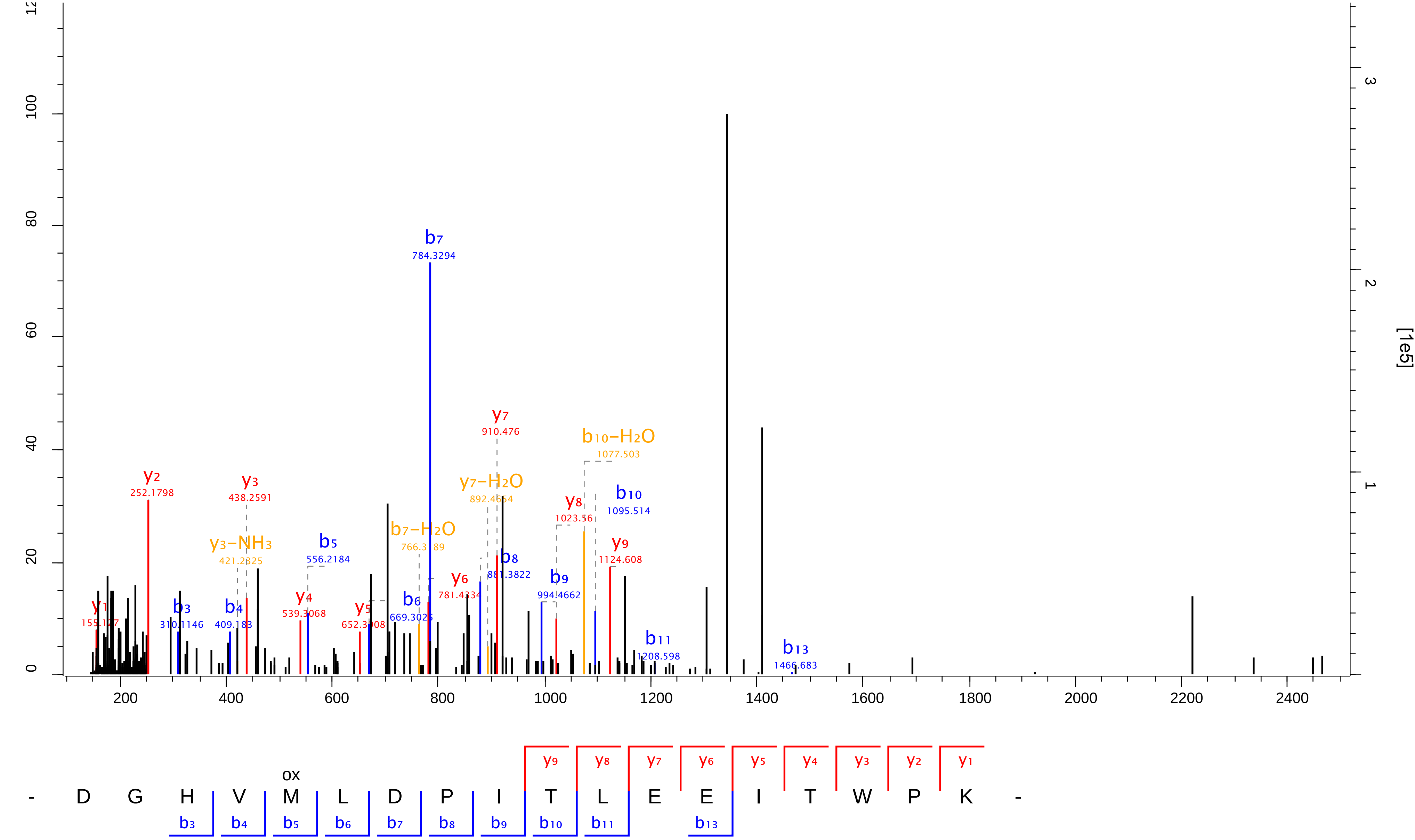

Raw file	Scan	Method	Score	m/z
QEplus003099	13058	FTMS; HCD	94.69	614.86

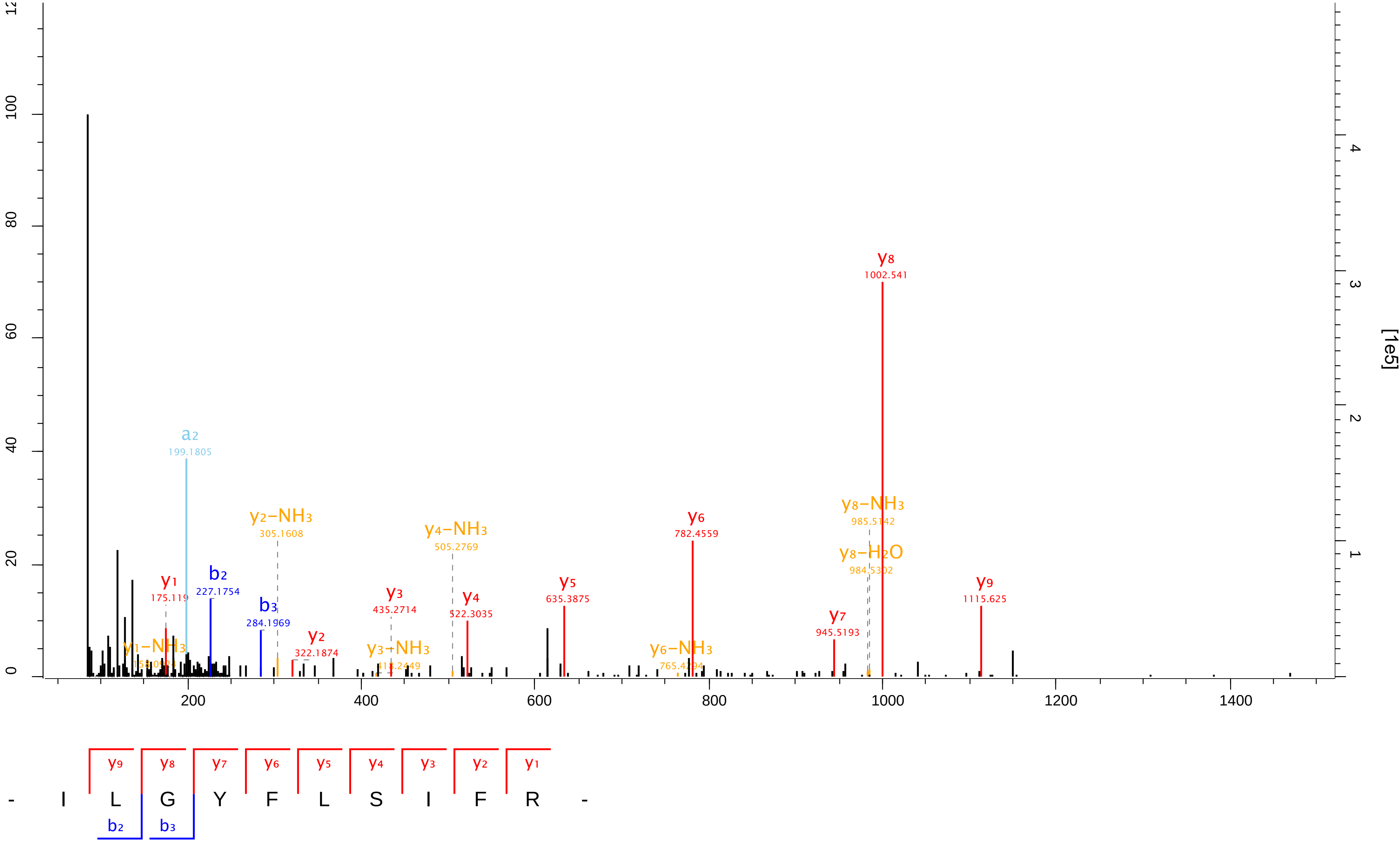

Raw file	Scan	Method	Score	m/z
QEplus003099	14784	FTMS; HCD	197.4	1298.71

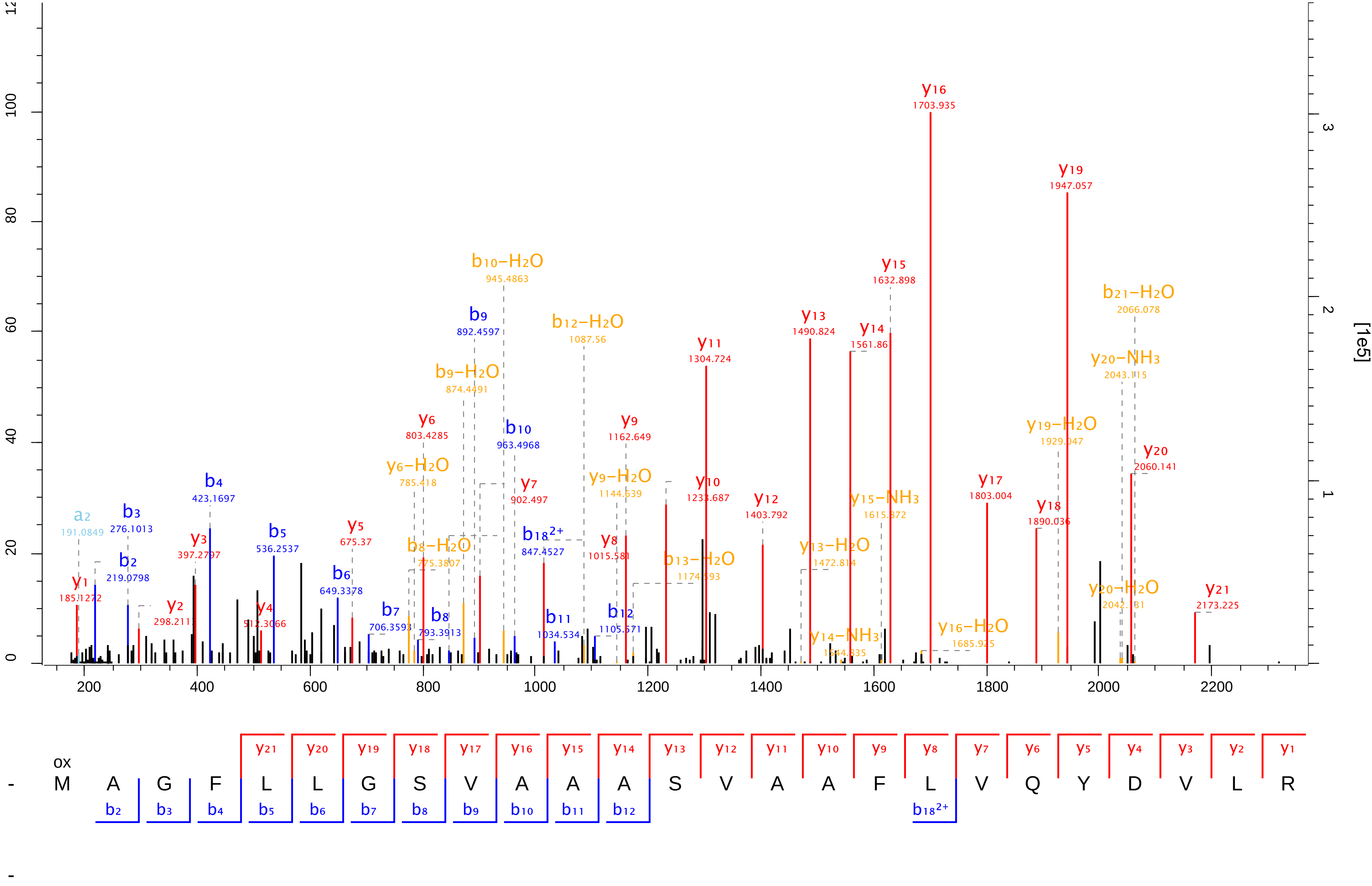

Raw file	Scan	Method	Score	m/z
QEplus003099	3375	FTMS; HCD	60.35	451.59

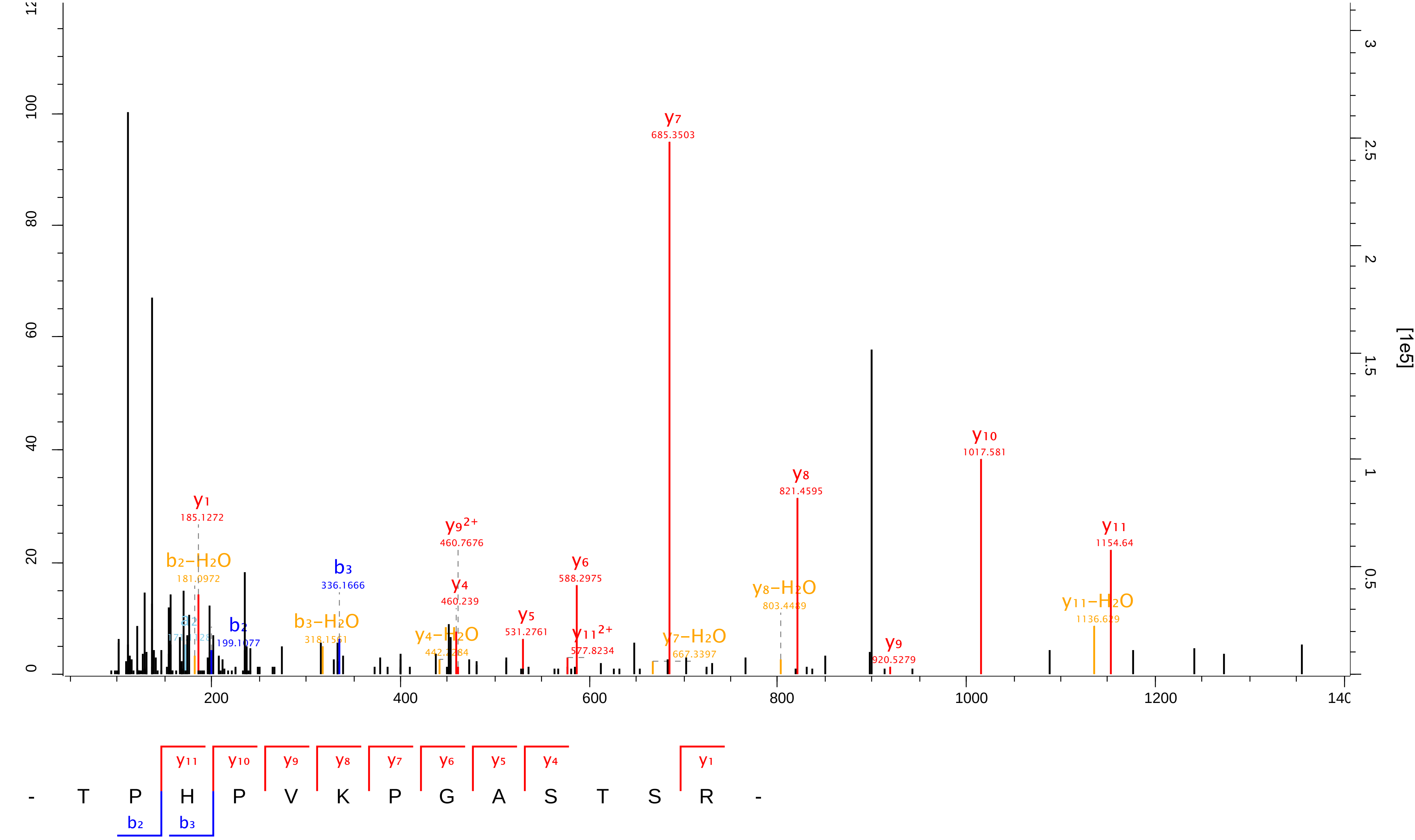

Raw file	Scan	Method	Score	m/z
QEplus003099	4148	FTMS; HCD	59.71	534.29

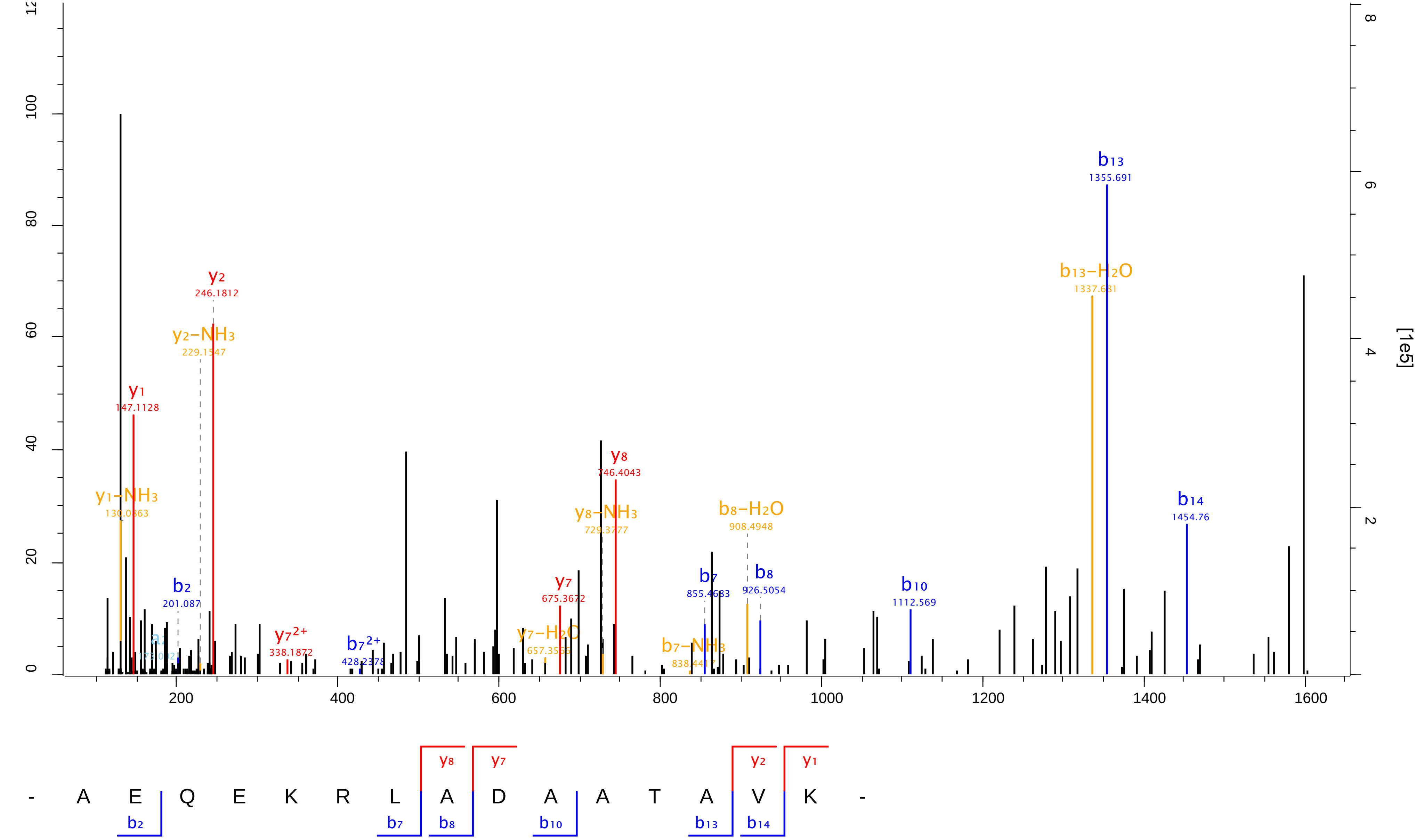

Raw file	Scan	Method	Score	m/z
QEplus003099	4829	FTMS; HCD	57.93	554.28

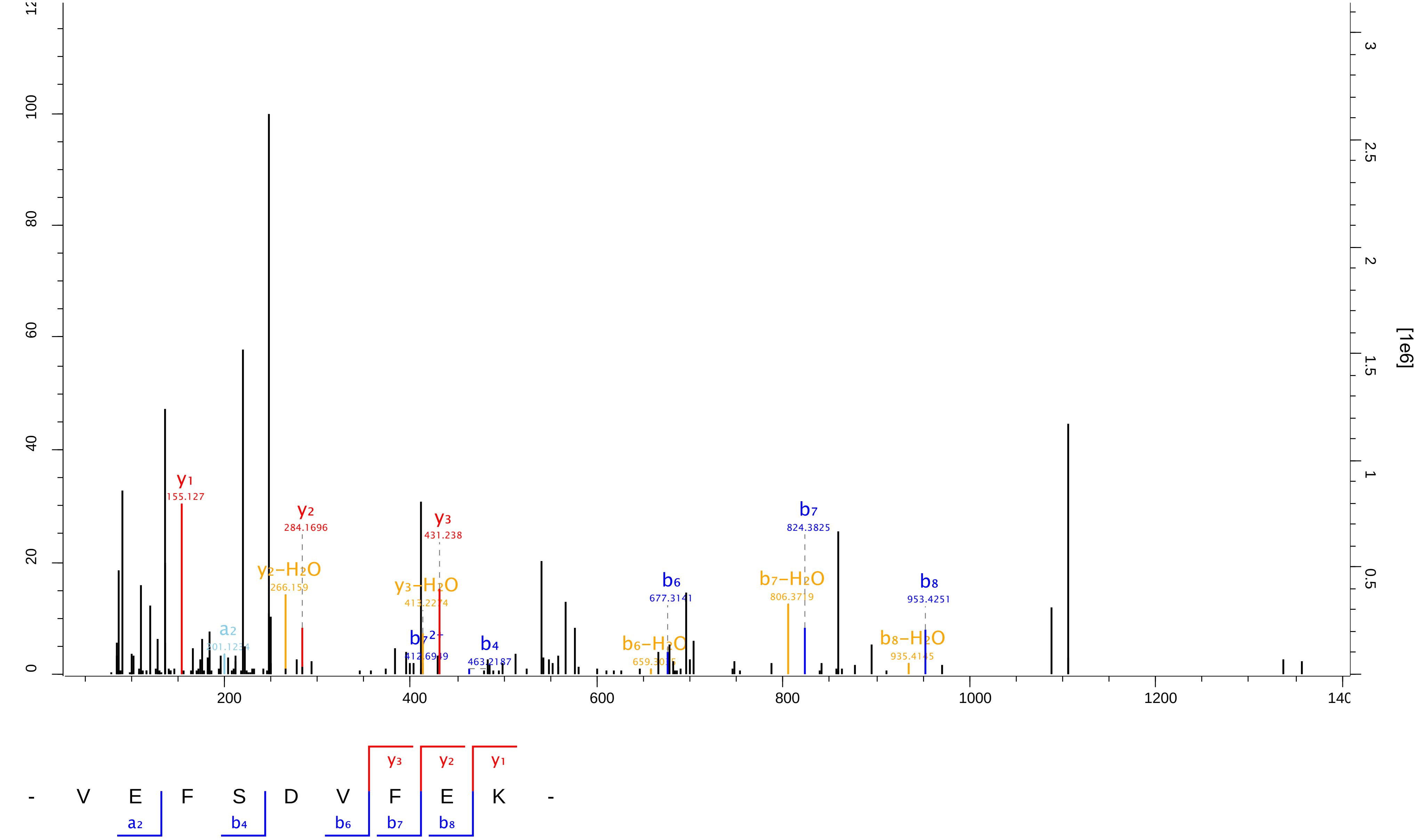

Raw file	Scan	Method	Score	m/z
QEplus003099	6396	FTMS; HCD	215.23	880.47

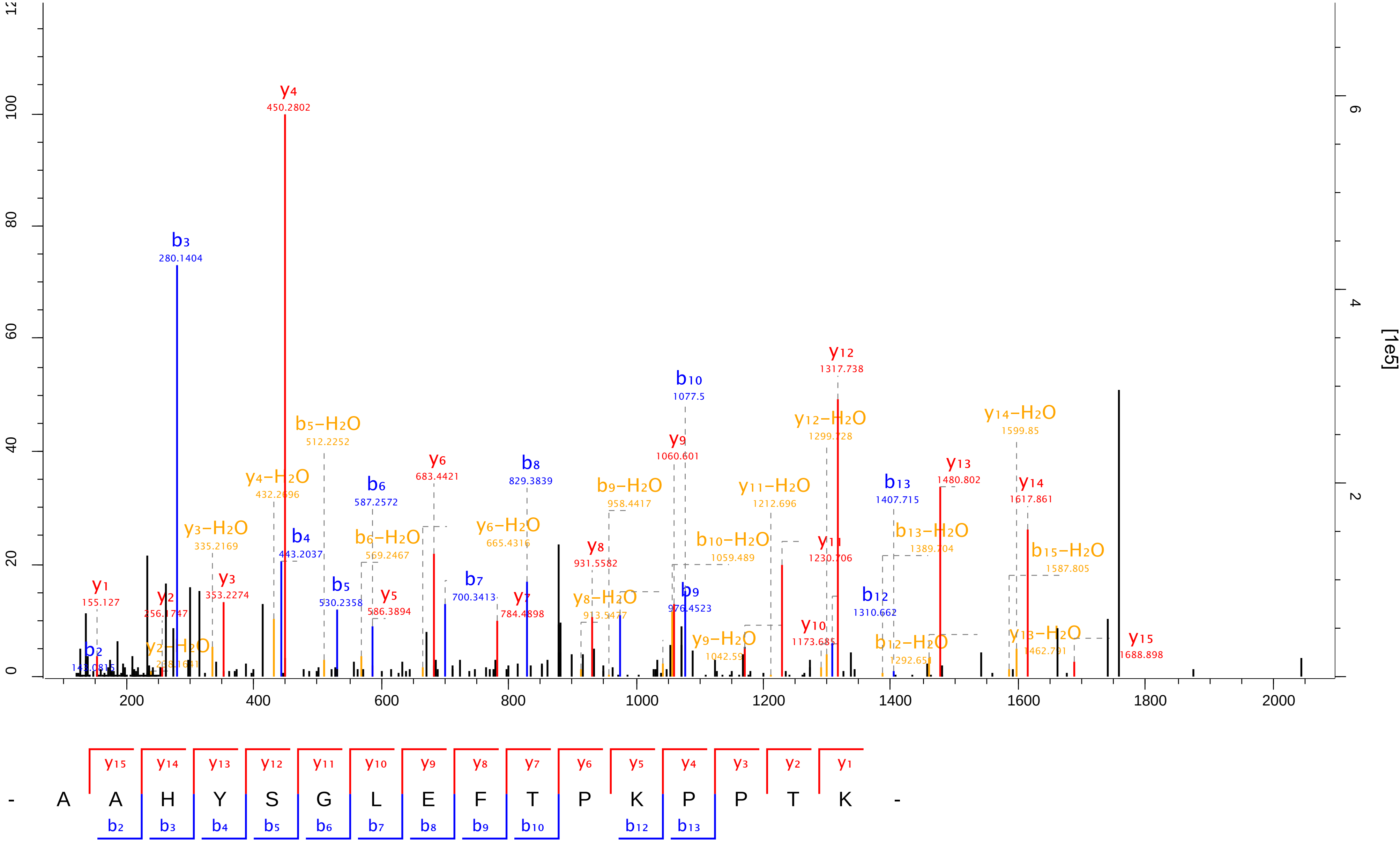

Raw file	Scan	Method	Score	m/z
QEplus003099	6519	FTMS; HCD	128.95	835.41

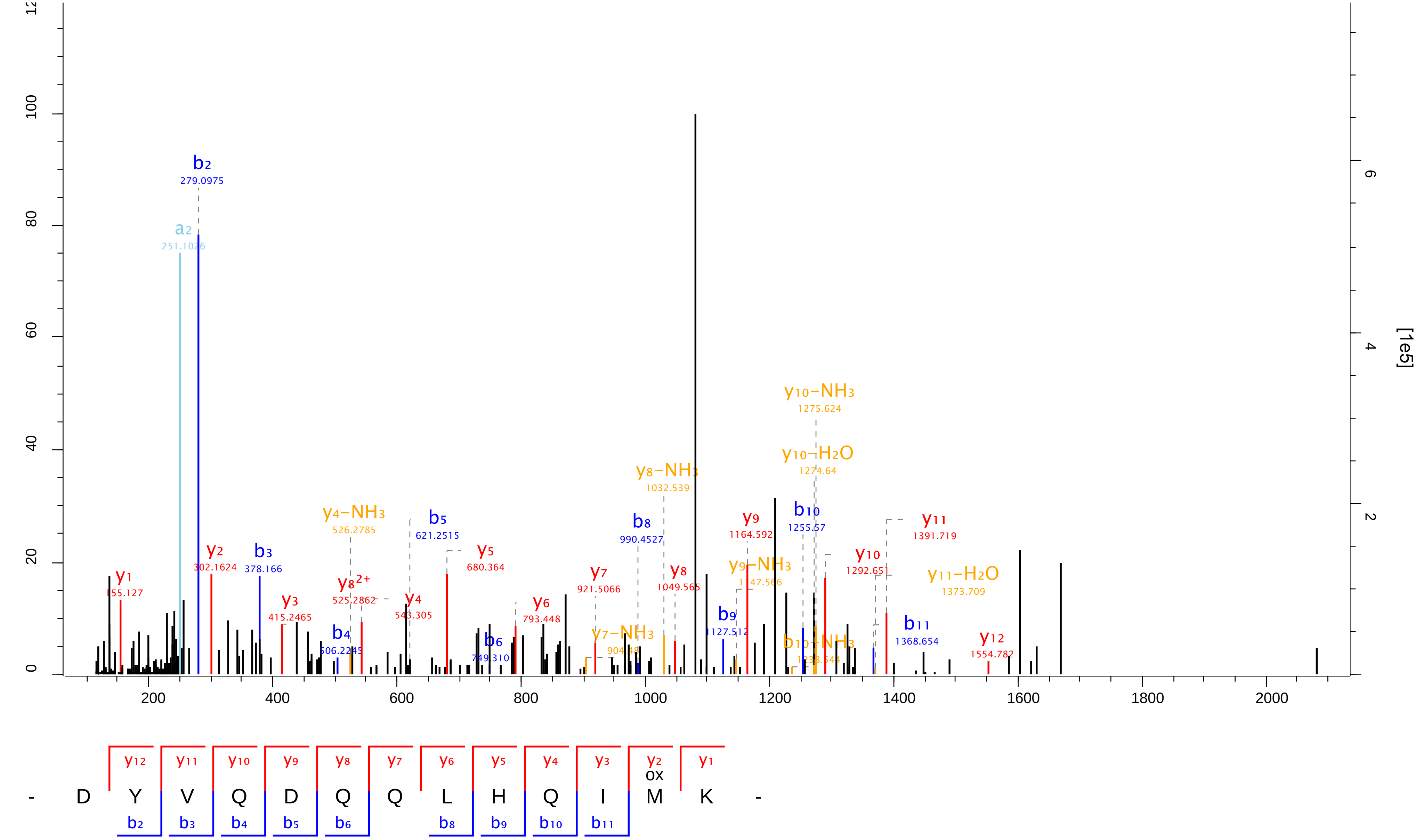

Raw file	Scan	Method	Score	m/z
QEplus003099	6692	FTMS; HCD	54.34	562.84

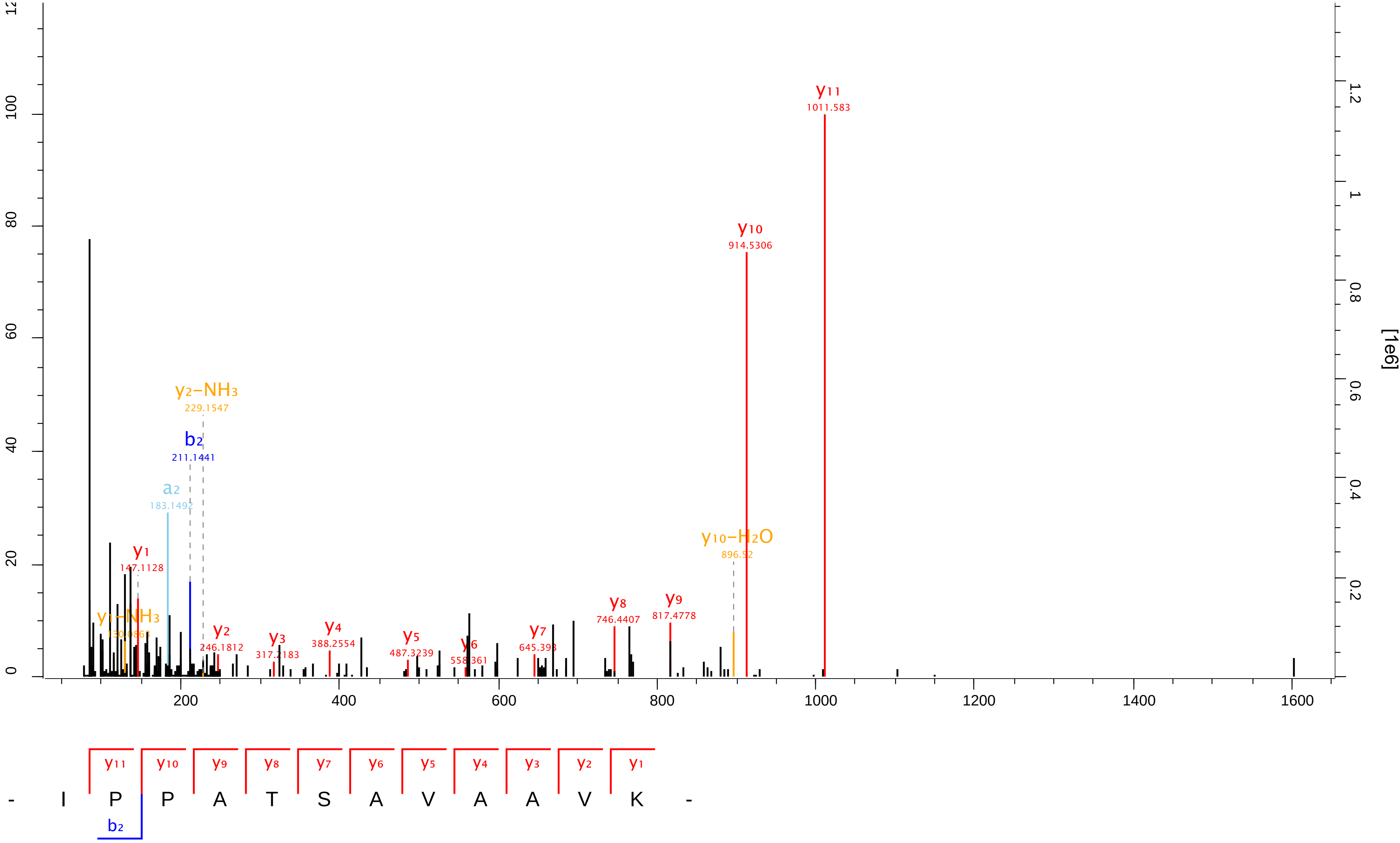

Raw file	Scan	Method	Score	m/z
QEplus003099	8798	FTMS; HCD	88.37	586.83

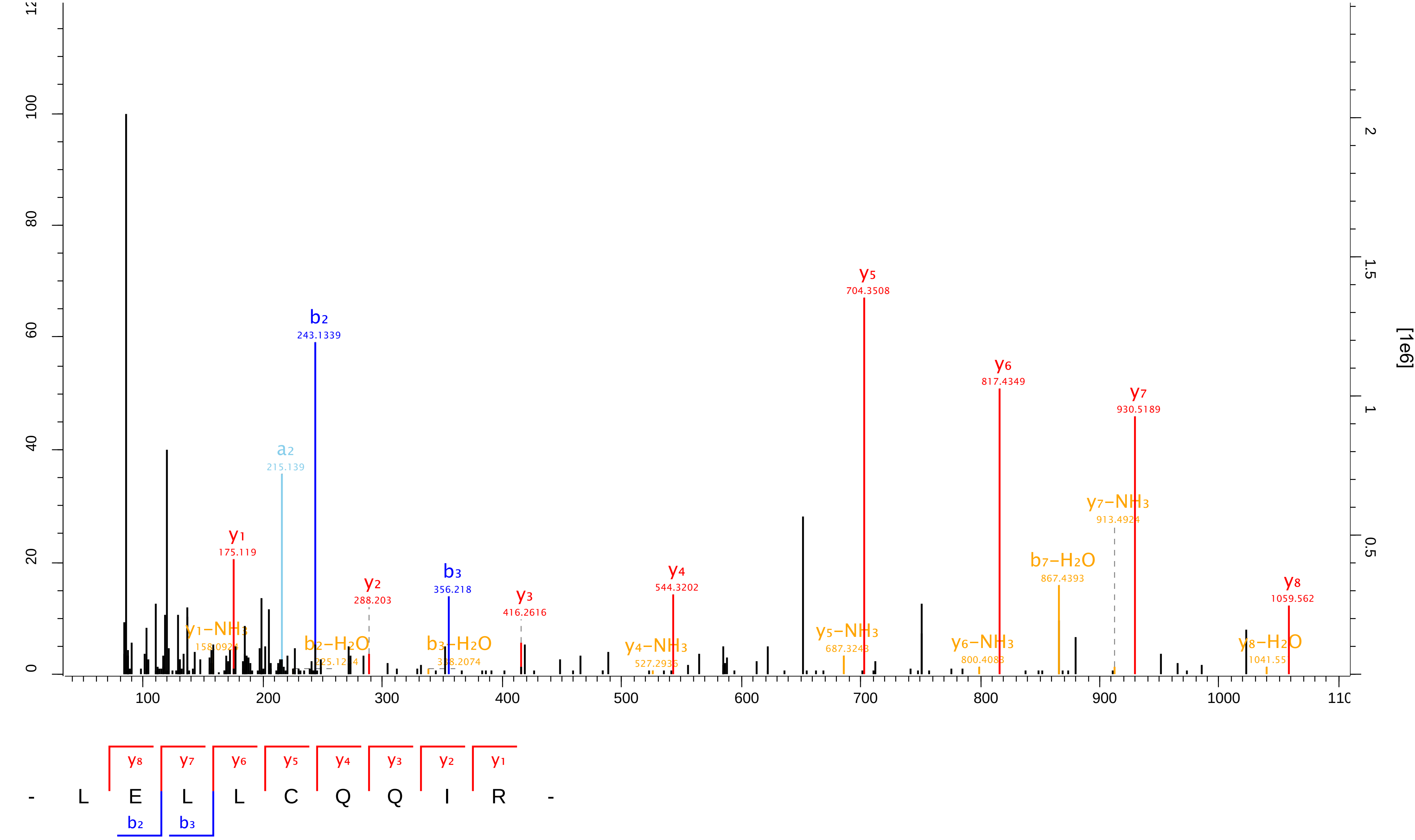

Raw file	Scan	Method	Score	m/z
QEplus003099	8872	FTMS; HCD	170.02	1052

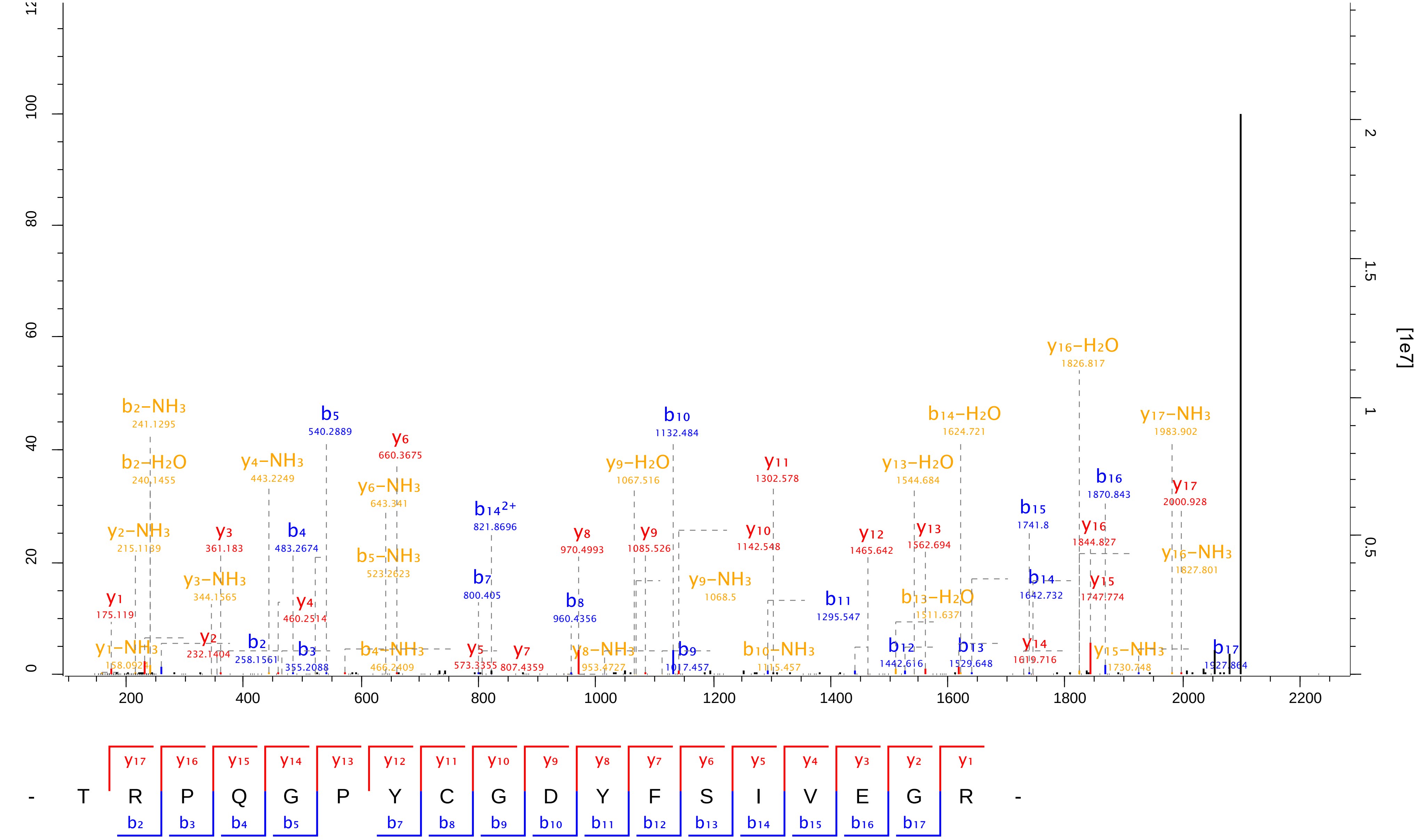

Raw file	Scan	Method	Score	m/z
QEplus003102	10880	FTMS; HCD	58.23	536.34

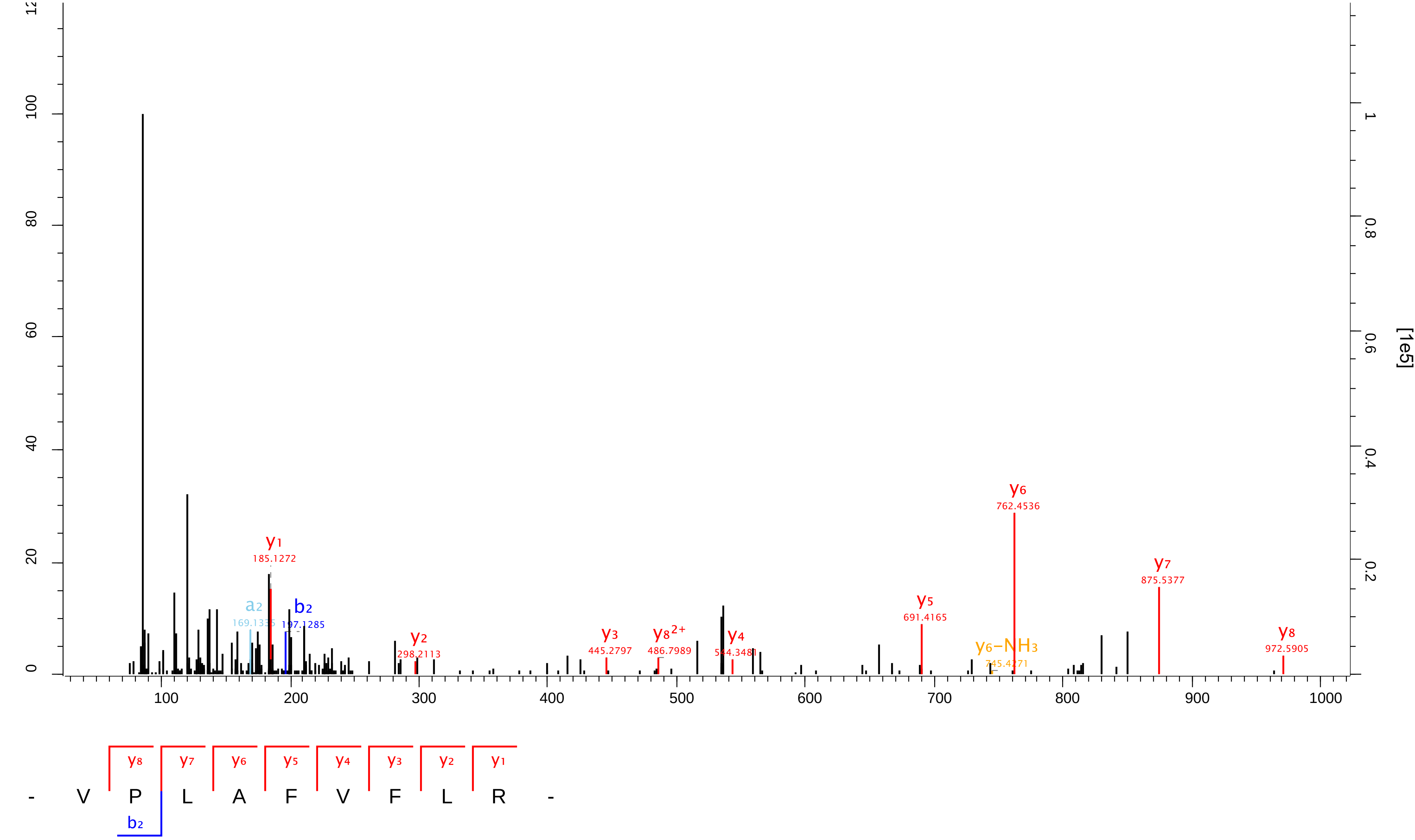

Raw file	Scan	Method	Score	m/z
QEplus003102	11561	FTMS; HCD	69.82	540.28

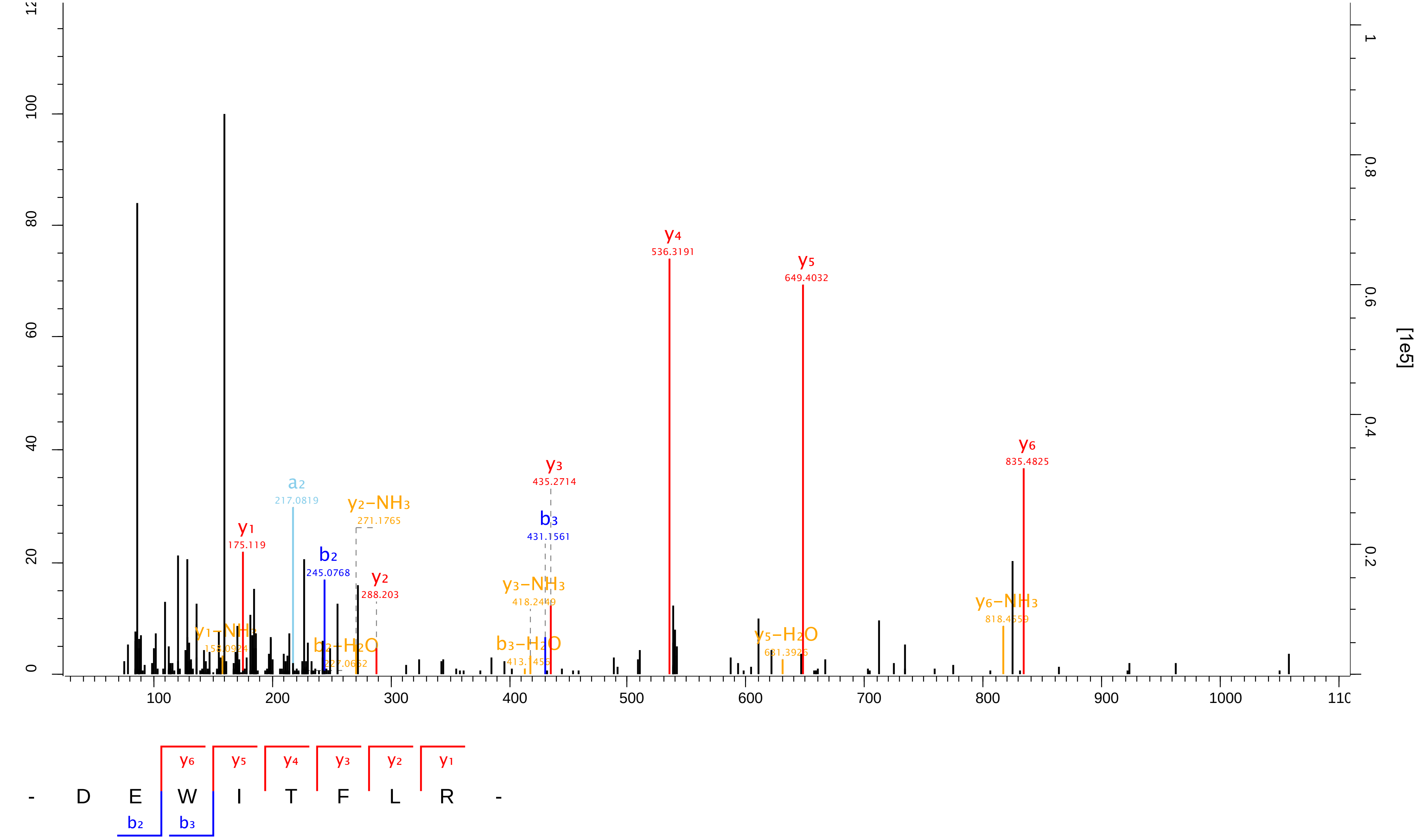

Raw file	Scan	Method	Score	m/z
QEplus003102	12254	FTMS; HCD	83.54	934.44

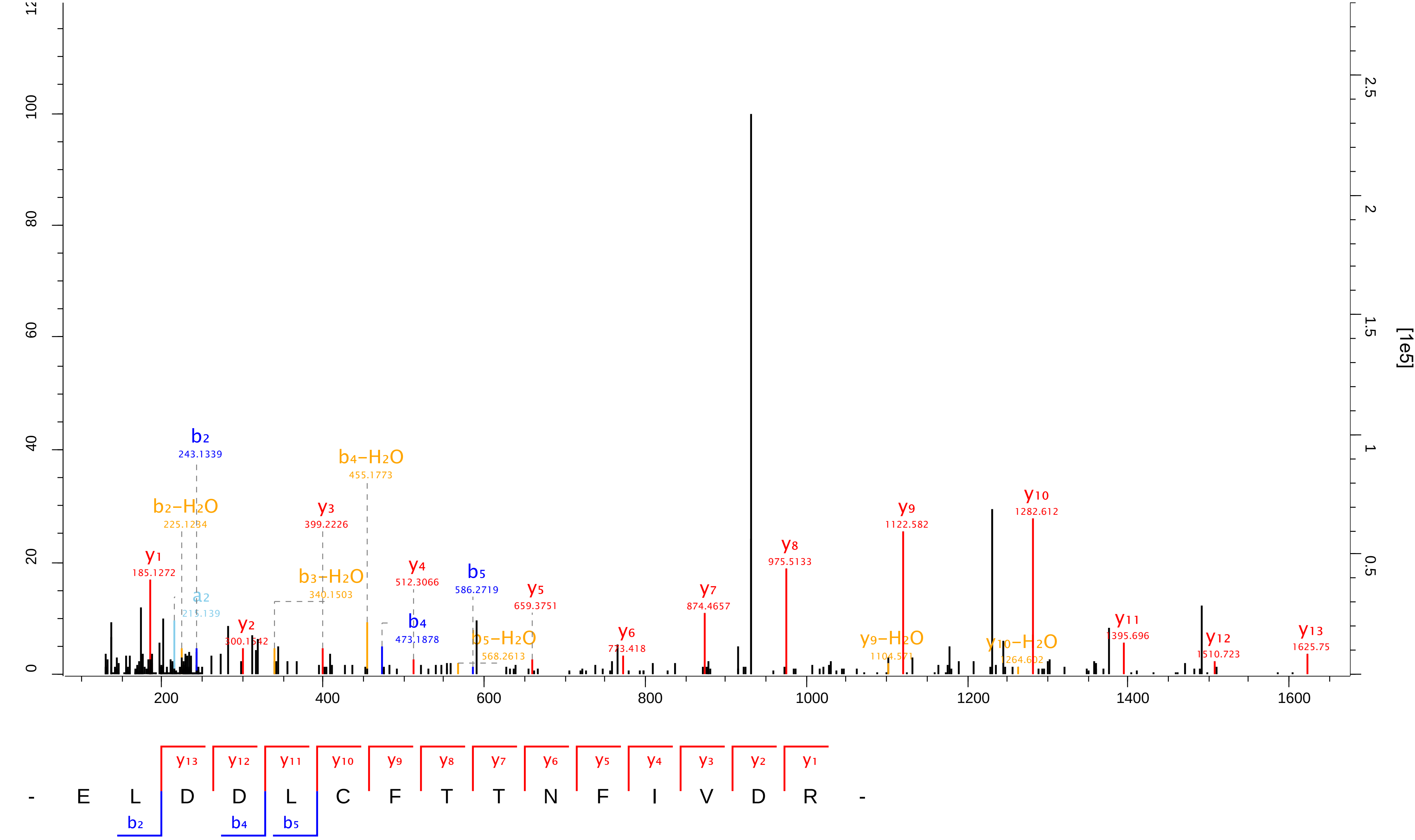

Raw file	Scan	Method	Score	m/z
QEplus003102	12594	FTMS; HCD	115.55	1025.56

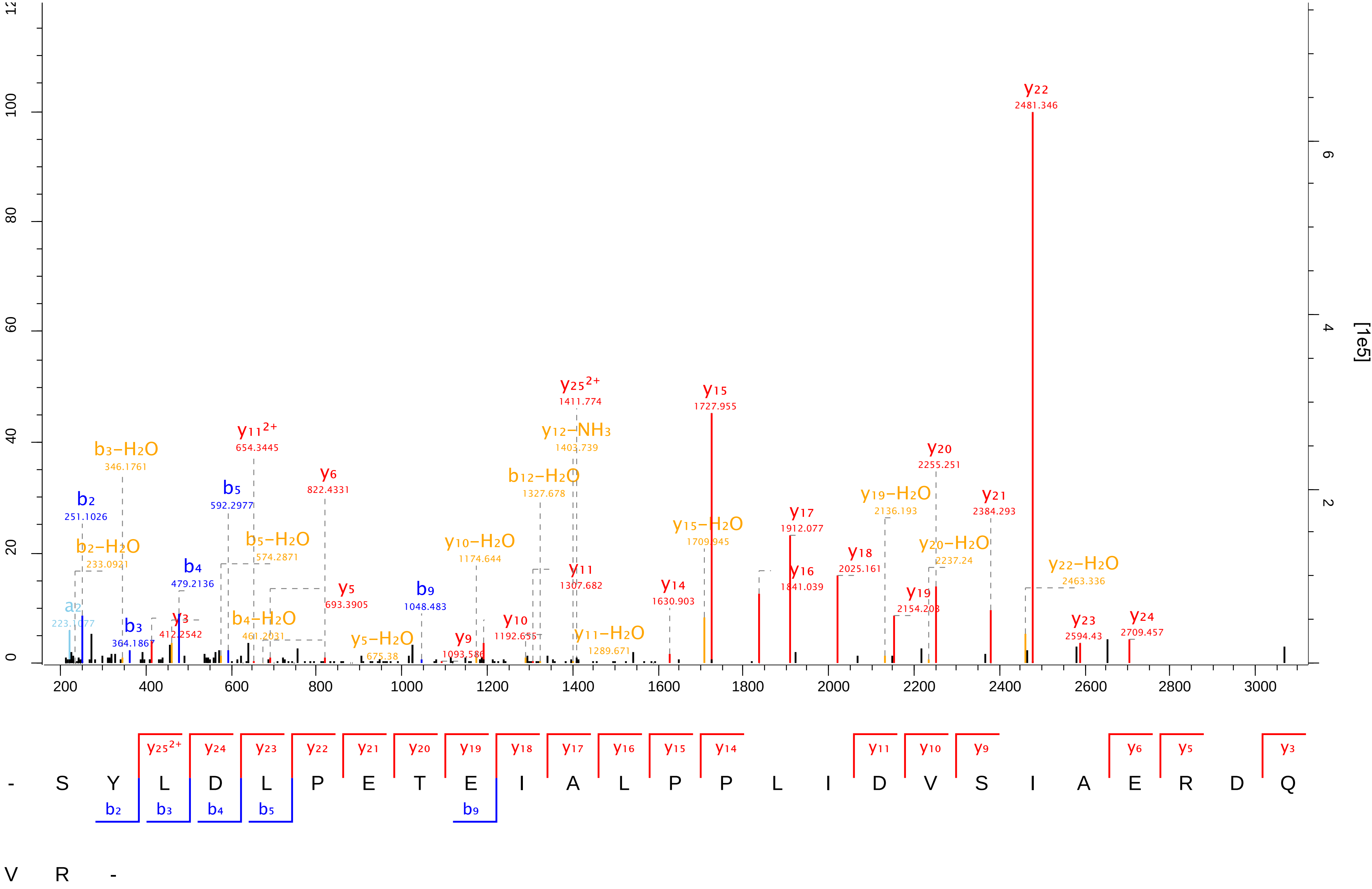

Raw file	Scan	Method	Score	m/z
QEplus003102	6951	FTMS; HCD	62	472.26

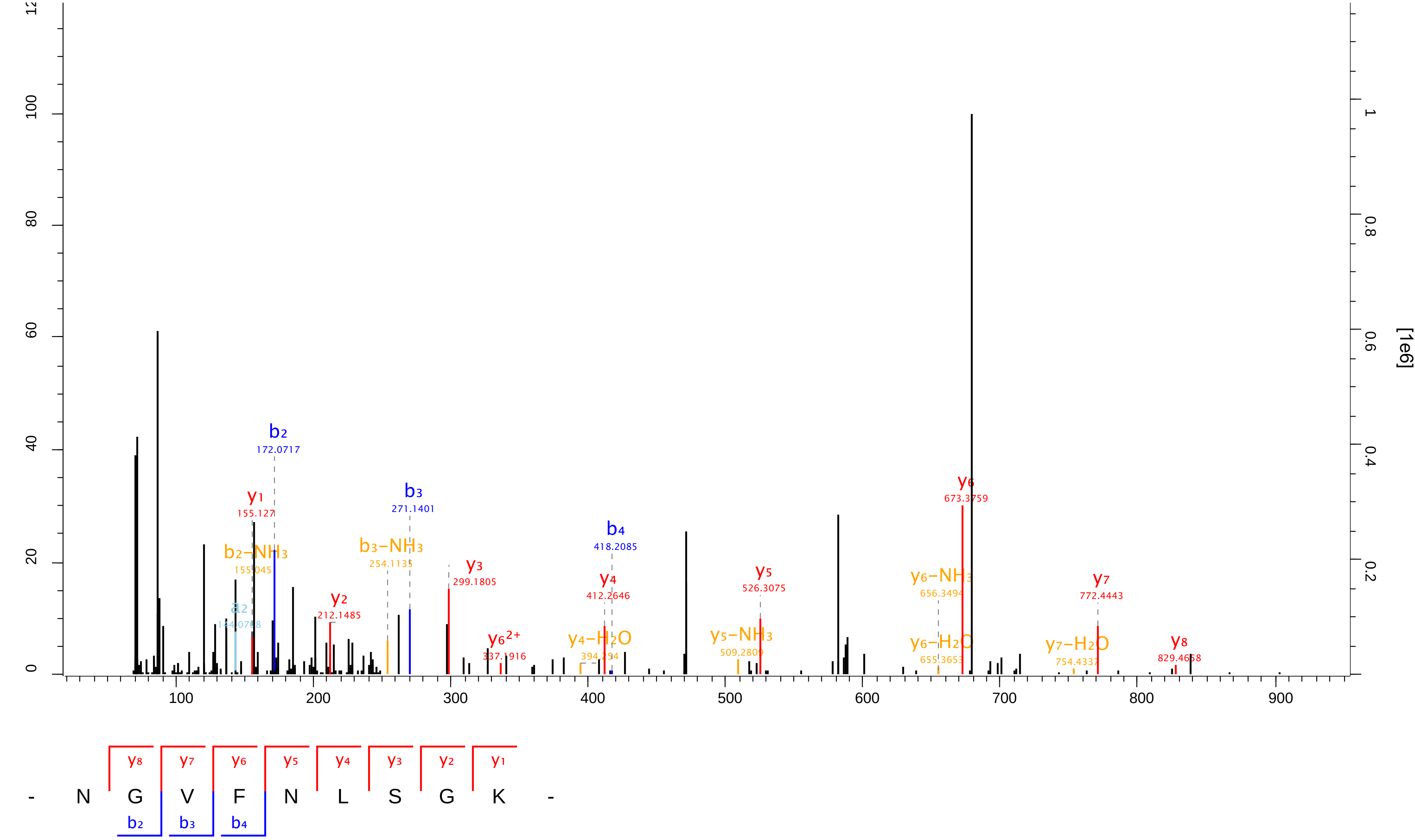

Raw file	Scan	Method	Score	m/z
QEplus003103	13698	FTMS; HCD	102.52	856.49

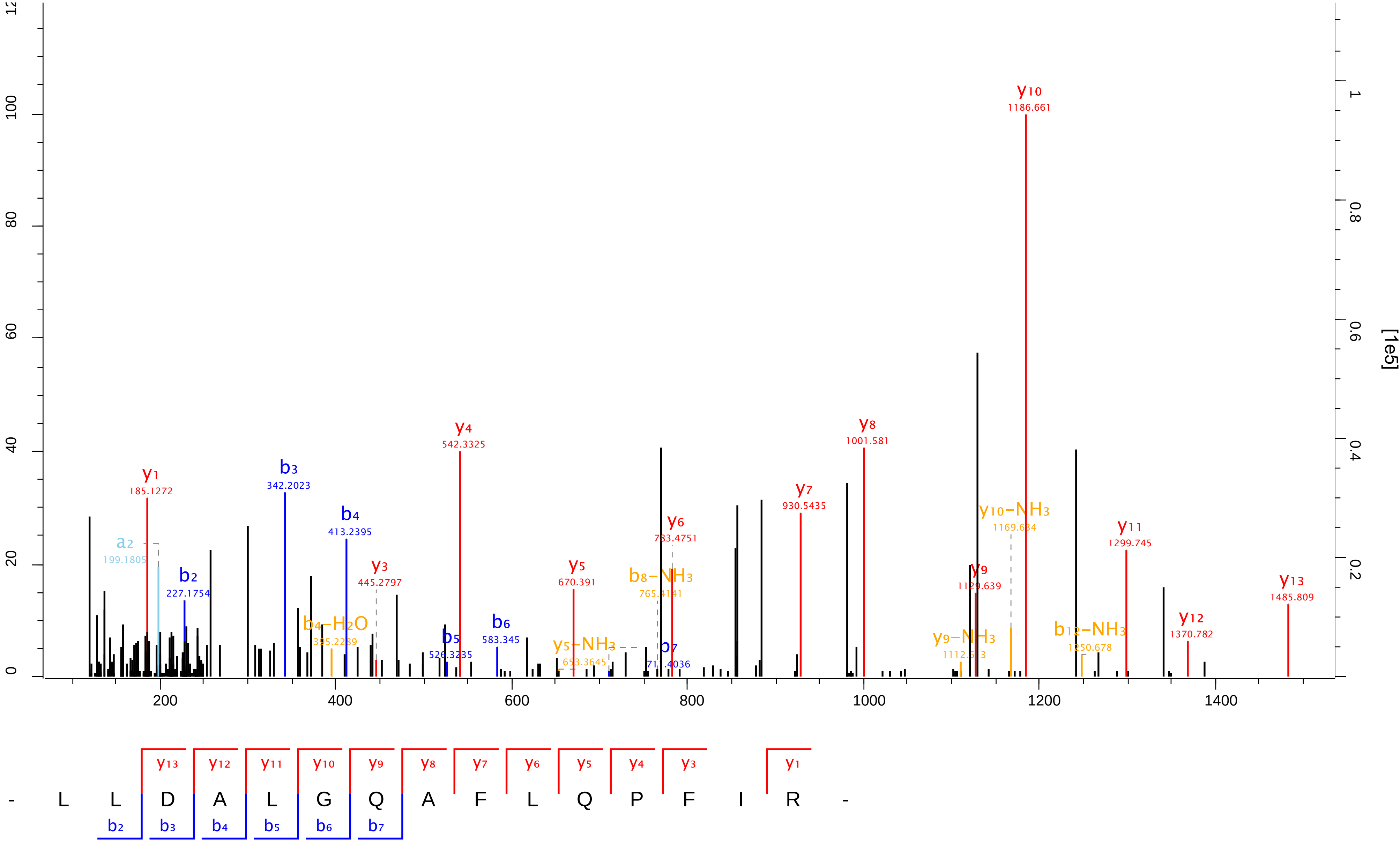

Raw file	Scan	Method	Score	m/z
QEplus003103	9903	FTMS; HCD	80.1	530.8

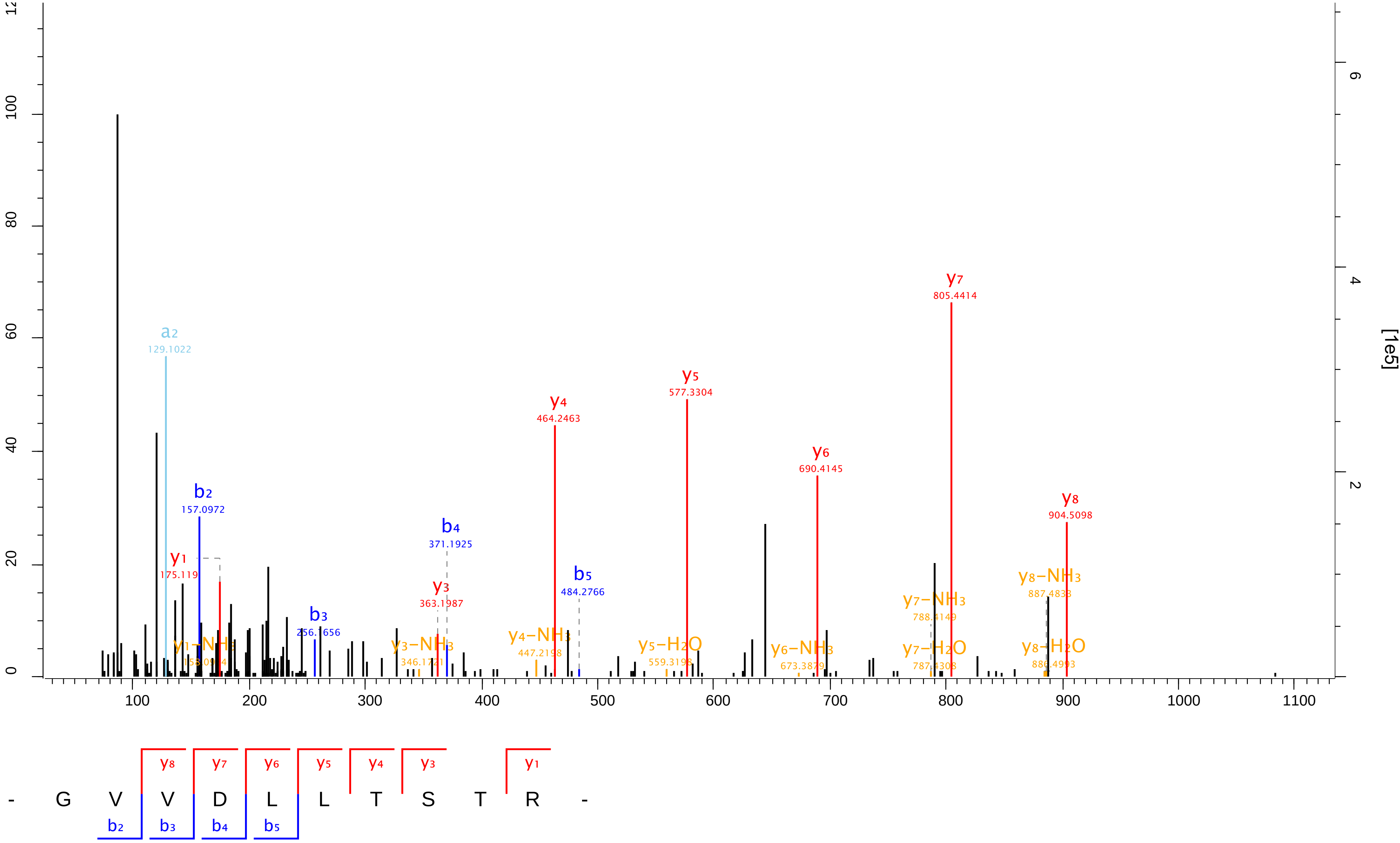

Raw file	Scan	Method	Score	m/z
QEplus003105	14169	FTMS; HCD	133.48	786.43

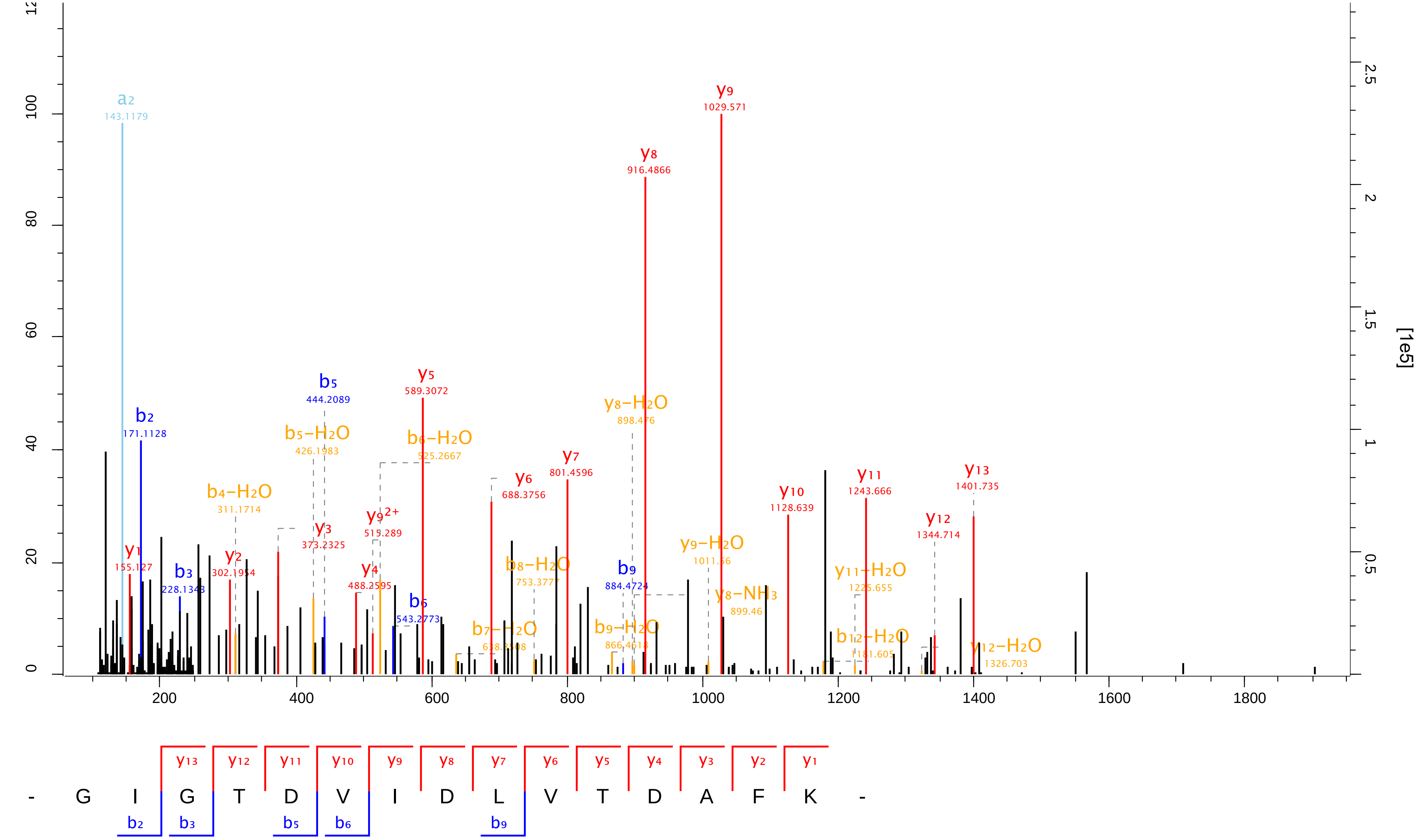

Raw file	Scan	Method	Score	m/z
QEplus003105	5904	FTMS; HCD	60.72	464.28

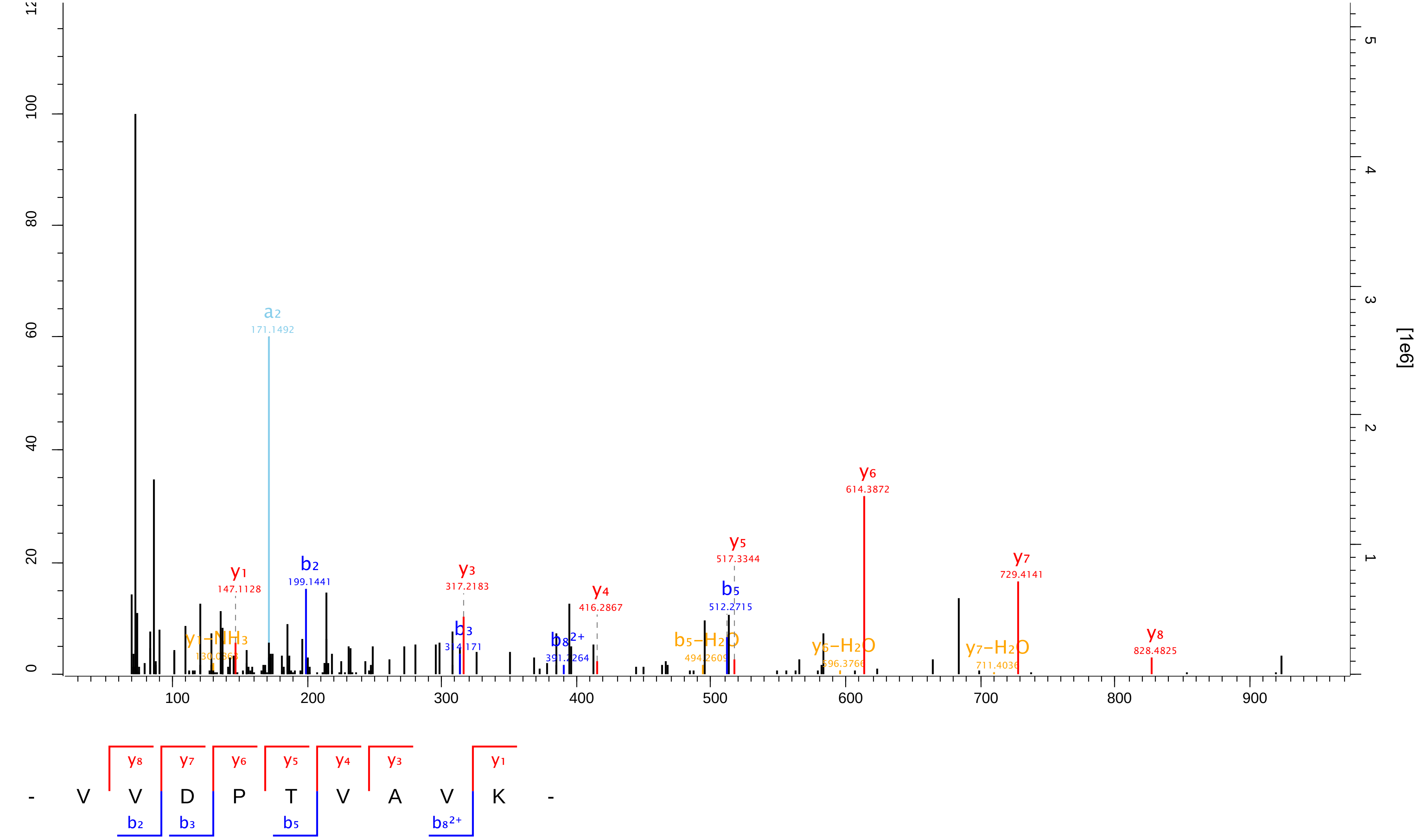

Raw file	Scan	Method	Score	m/z
QEplus003105	8120	FTMS; HCD	77.92	431.76

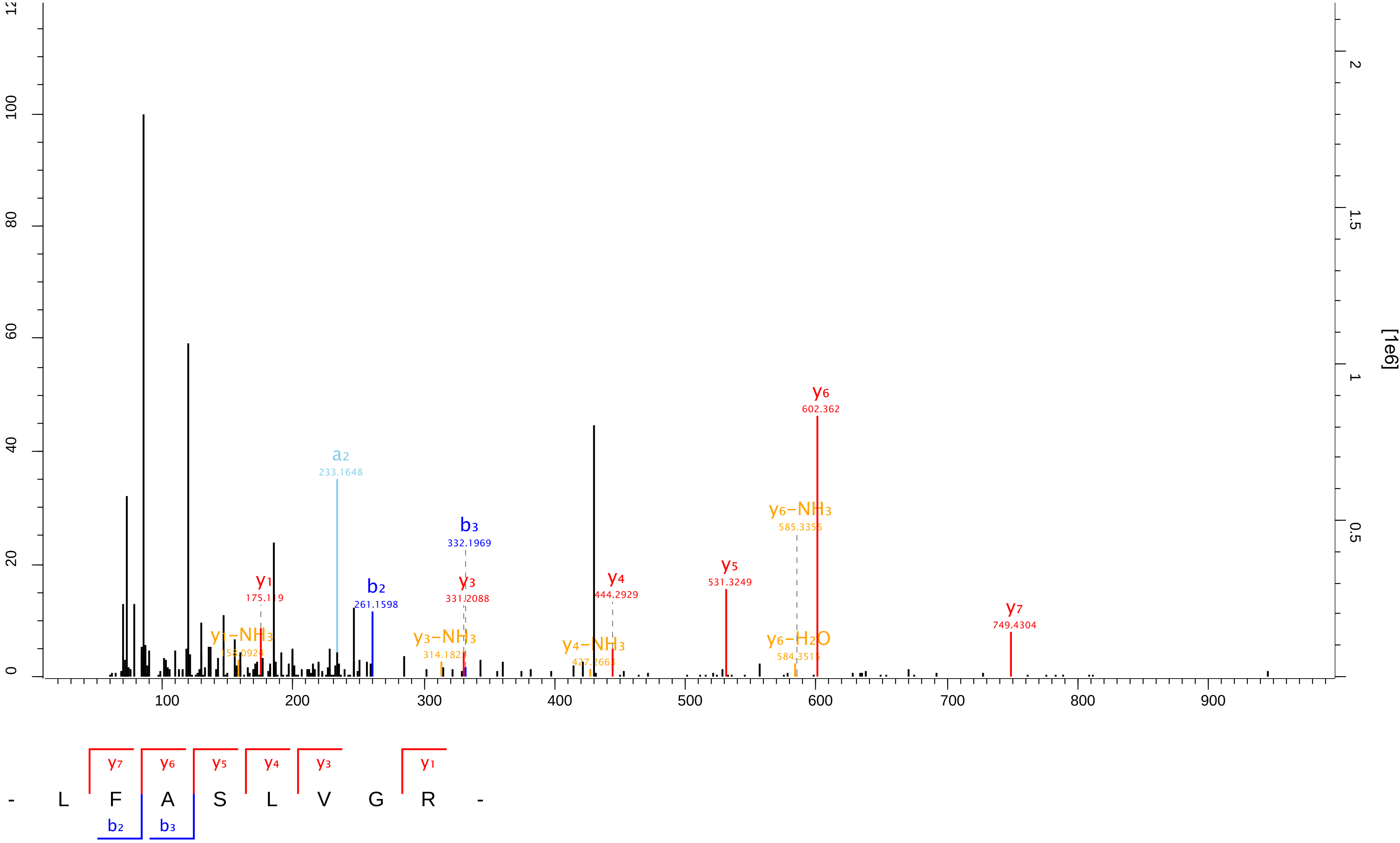

Raw file	Scan	Method	Score	m/z
QEplus003106	3602	FTMS; HCD	62.17	391.56

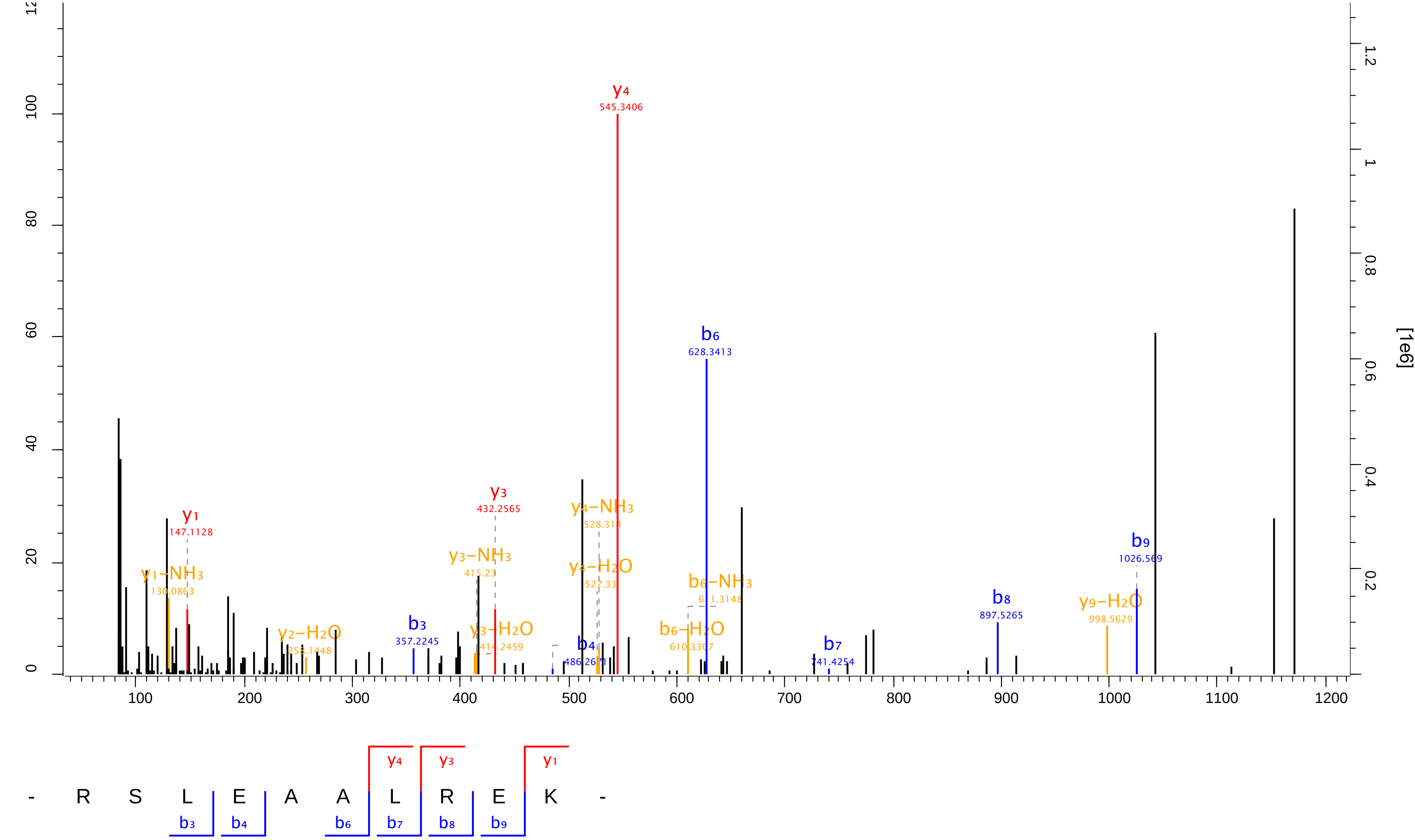

Raw file	Scan	Method	Score	m/z
QEplus003106	6797	FTMS; HCD	155.08	778.94

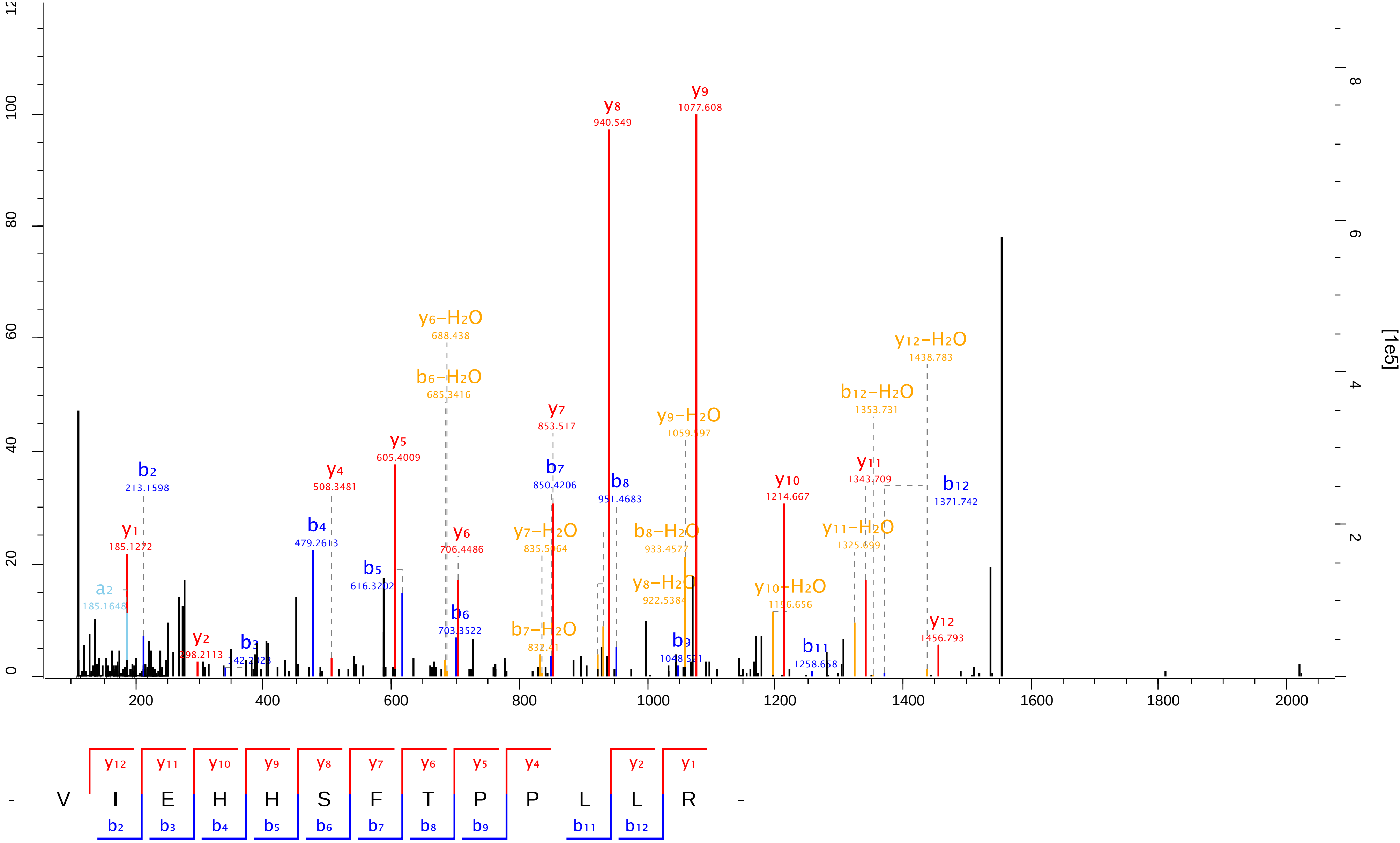

Raw file	Scan	Method	Score	m/z
QEplus003107	11359	FTMS; HCD	81.34	557.35

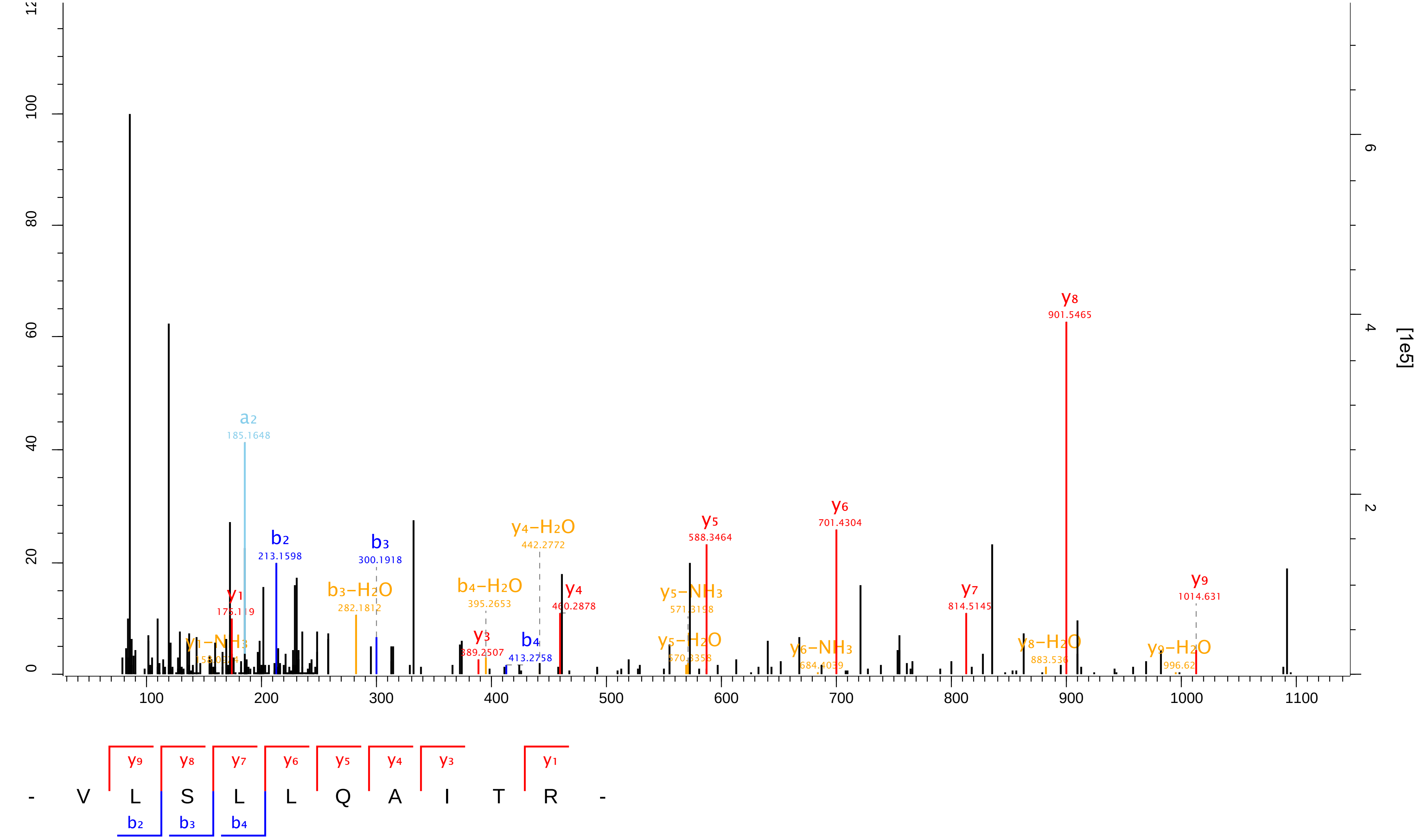

Raw file	Scan	Method	Score	m/z
QEplus003107	12498	FTMS; HCD	96.34	603.39

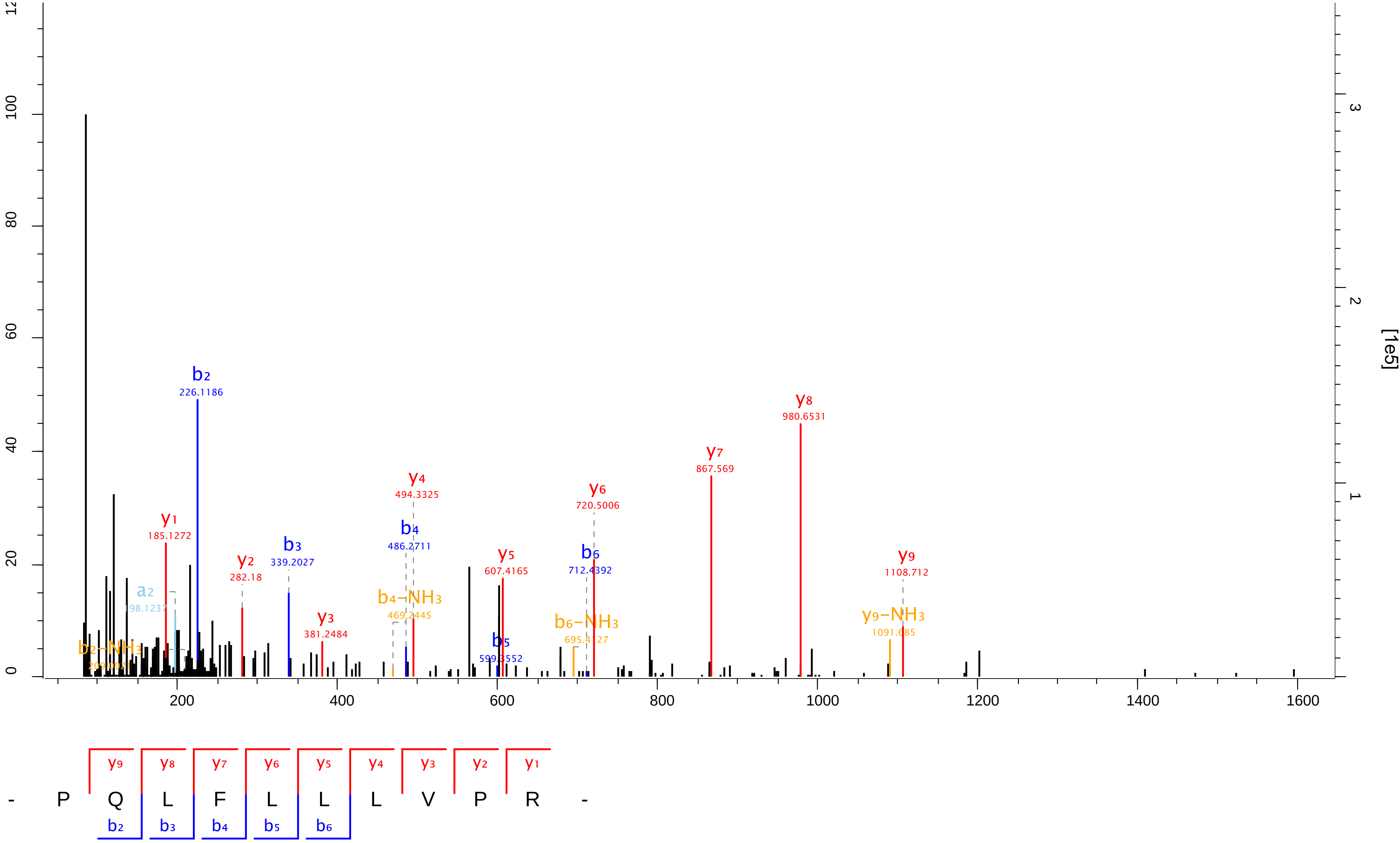

Raw file	Scan	Method	Score	m/z
QEplus003107	12593	FTMS; HCD	77.92	509.28

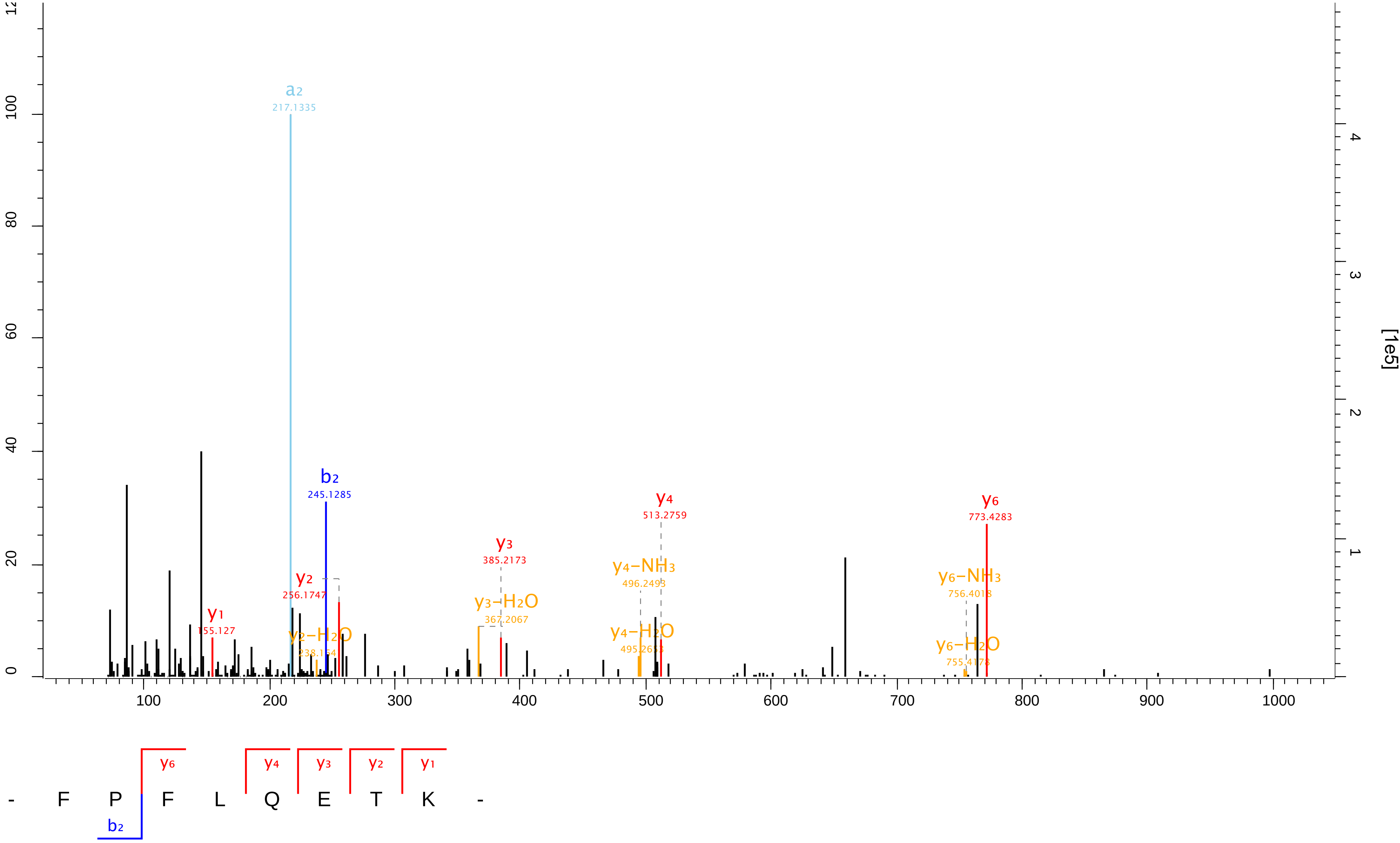

Raw file	Scan	Method	Score	m/z
QEplus003107	12622	FTMS; HCD	90.43	621.38

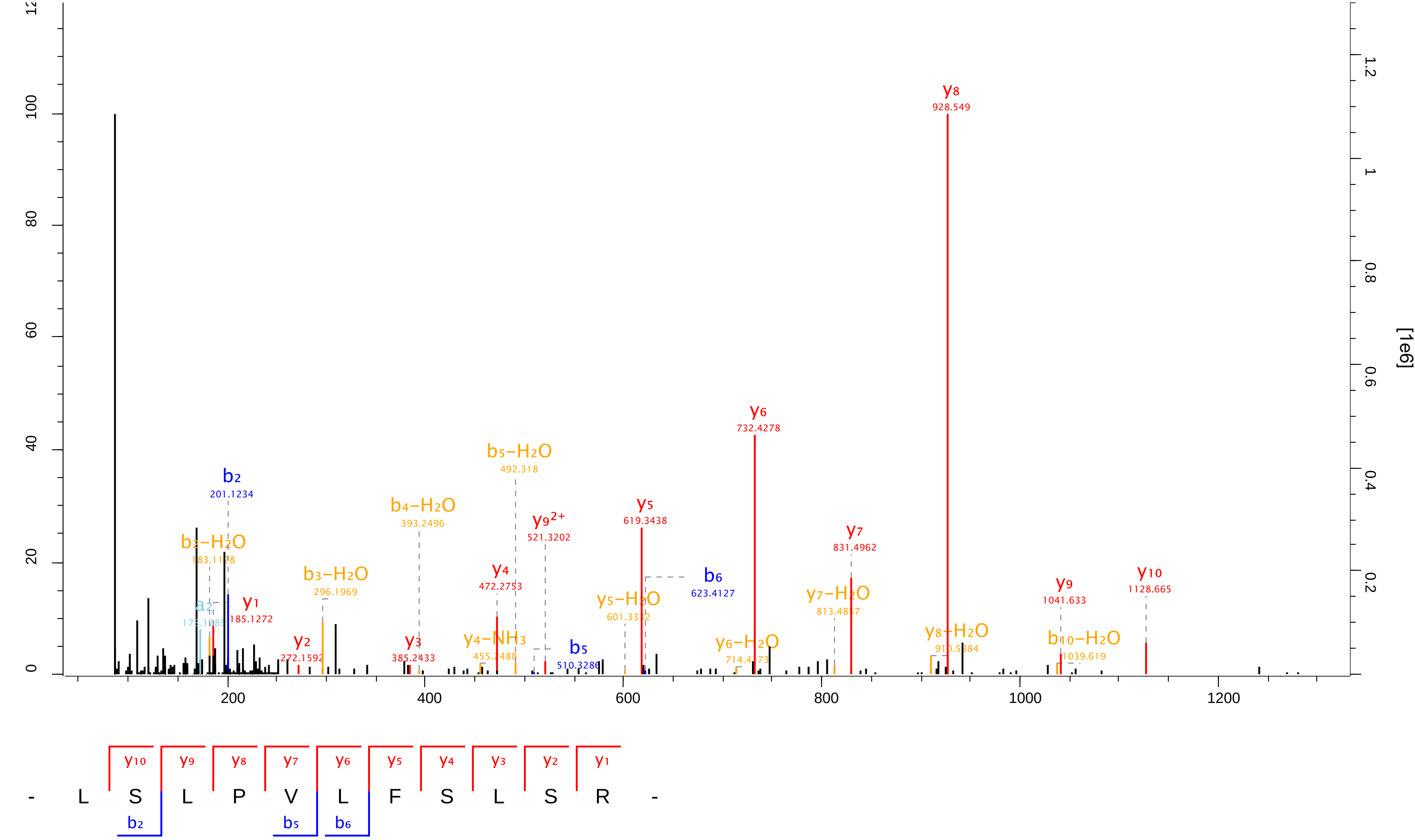

Raw file	Scan	Method	Score	m/z
QEplus003107	14014	FTMS; HCD	90.05	640.85

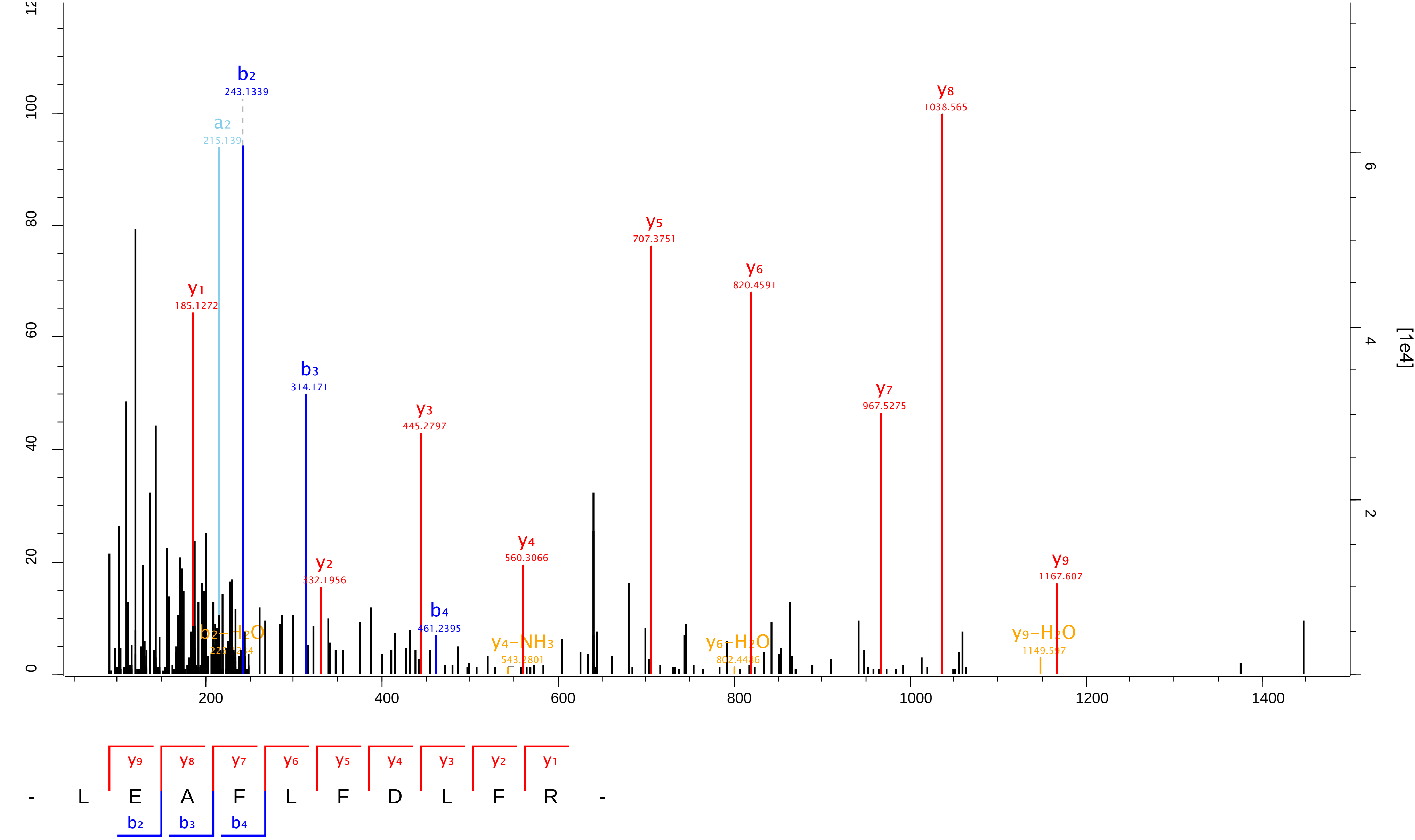

Raw file	Scan	Method	Score	m/z
QEplus003107	6834	FTMS; HCD	58.7	517.26

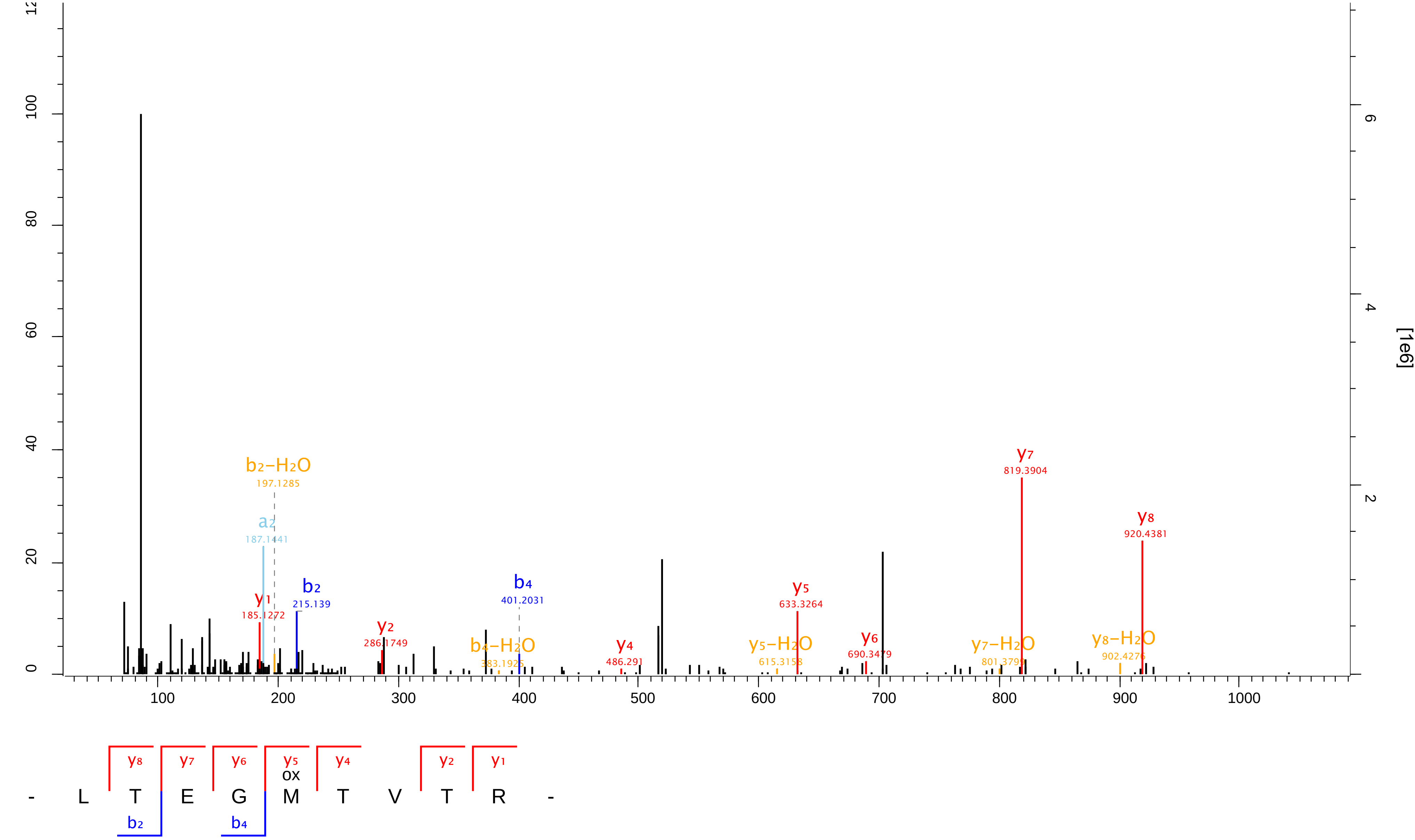

Raw file	Scan	Method	Score	m/z
QEplus003109	14679	FTMS; HCD	99.28	628.39

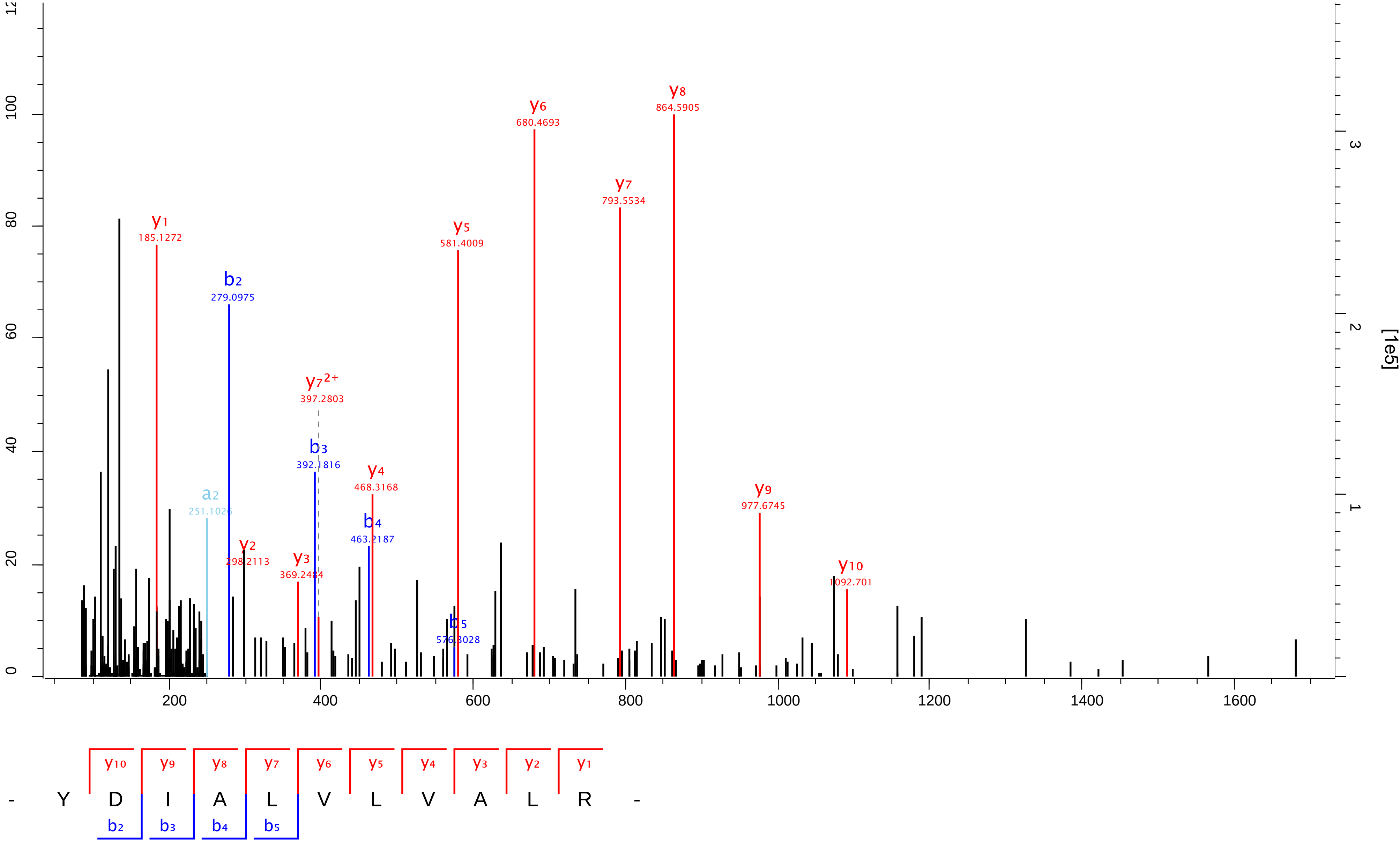

Raw file	Scan	Method	Score	m/z
QEplus003109	4480	FTMS; HCD	137.8	594.64

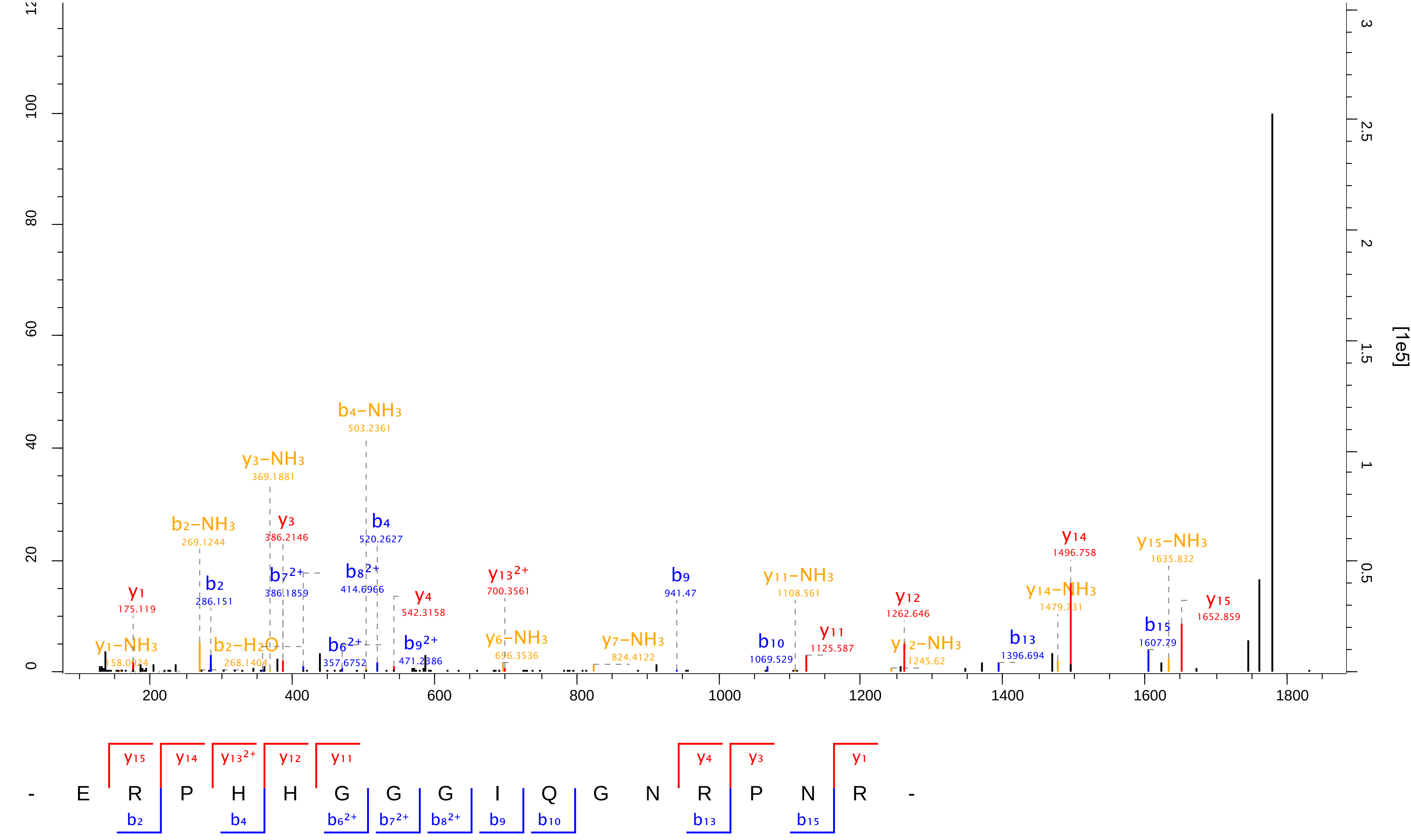

Raw file	Scan	Method	Score	m/z
QEplus003109	5617	FTMS; HCD	56.71	698.98

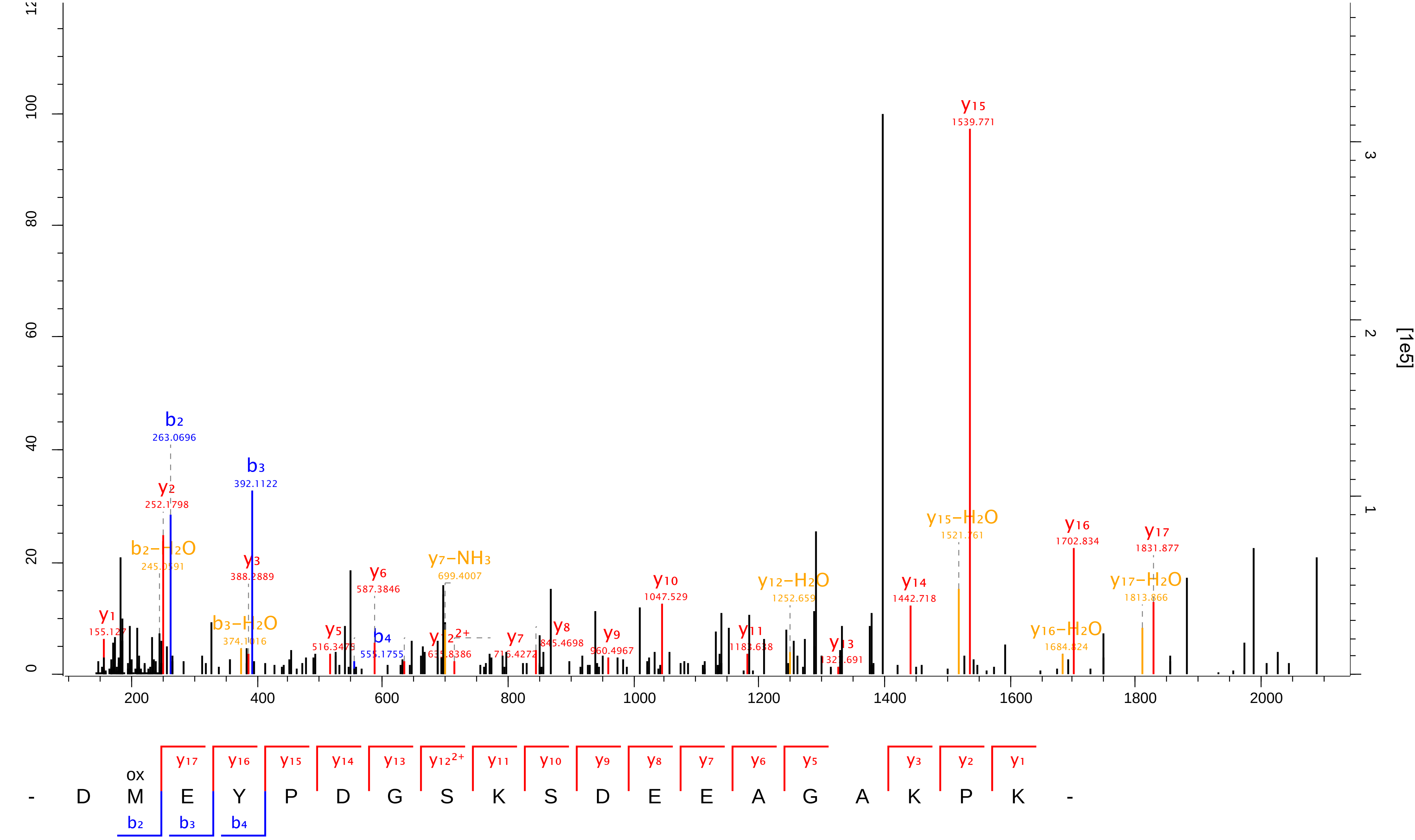

Raw file	Scan	Method	Score	m/z
QEplus003109	6641	FTMS; HCD	91.41	668.02

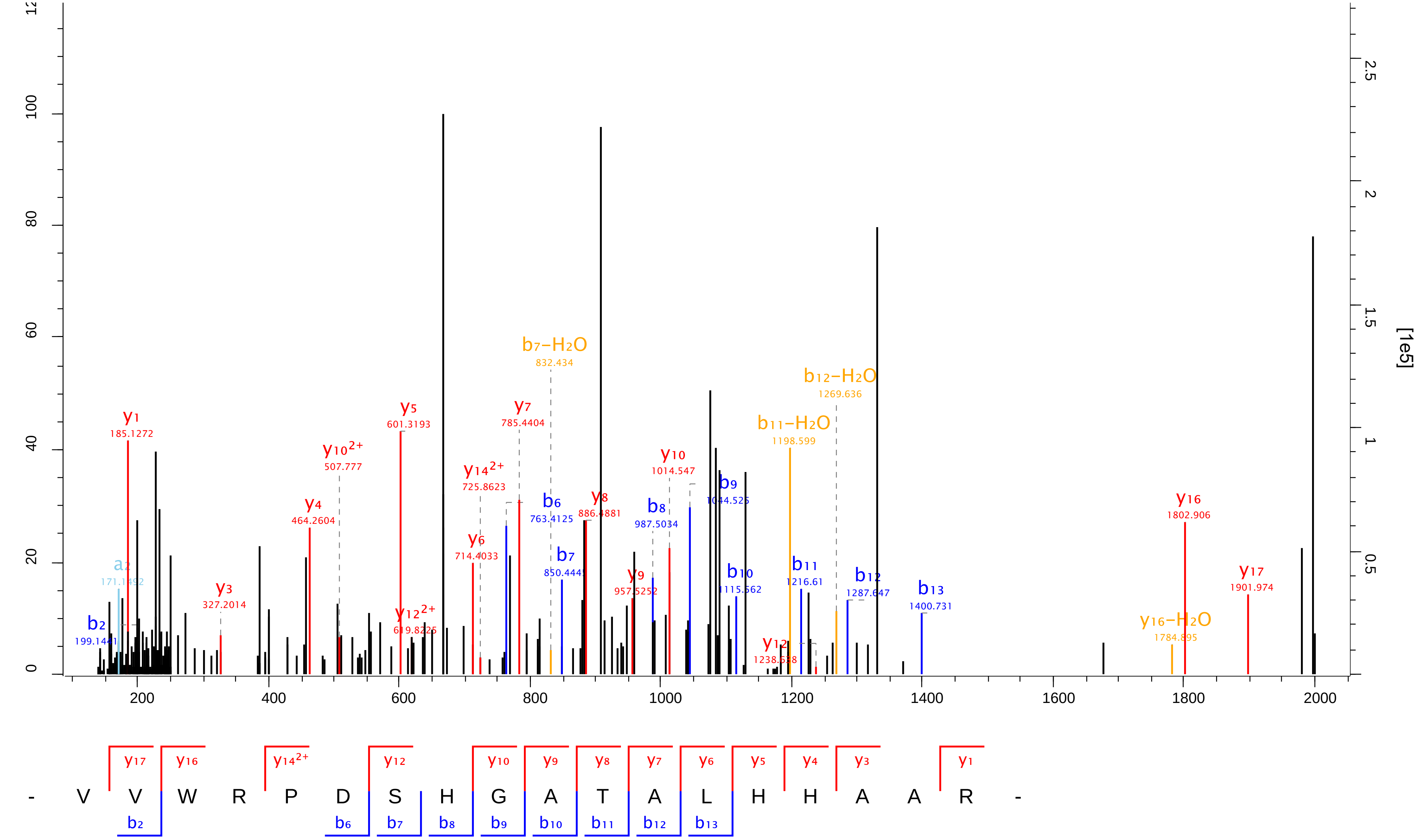

Raw file	Scan	Method	Score	m/z
QEplus003109	7838	FTMS; HCD	64.22	608.84

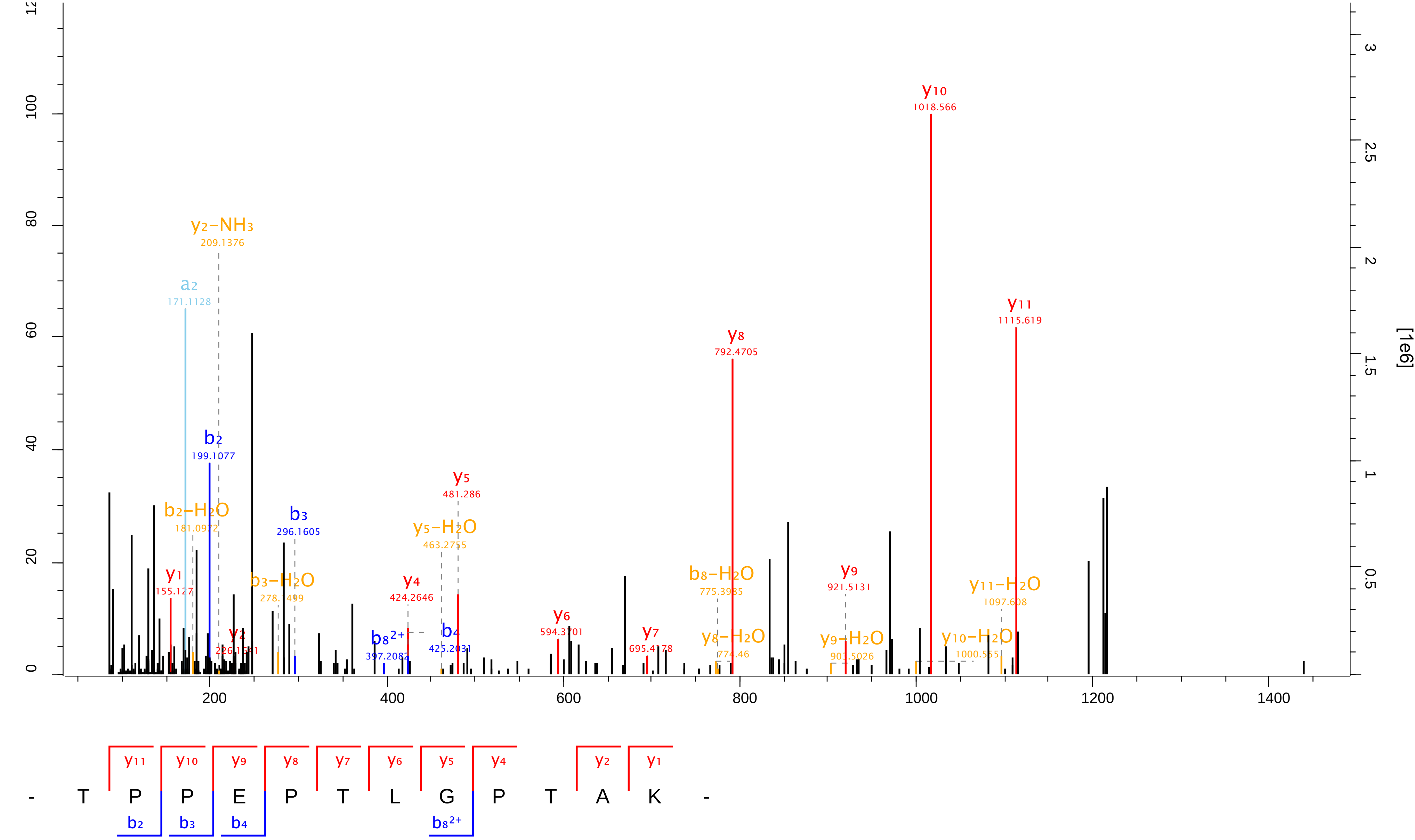

Raw file	Scan	Method	Score	m/z
QEplus003110	11771	FTMS; HCD	80.37	714.91

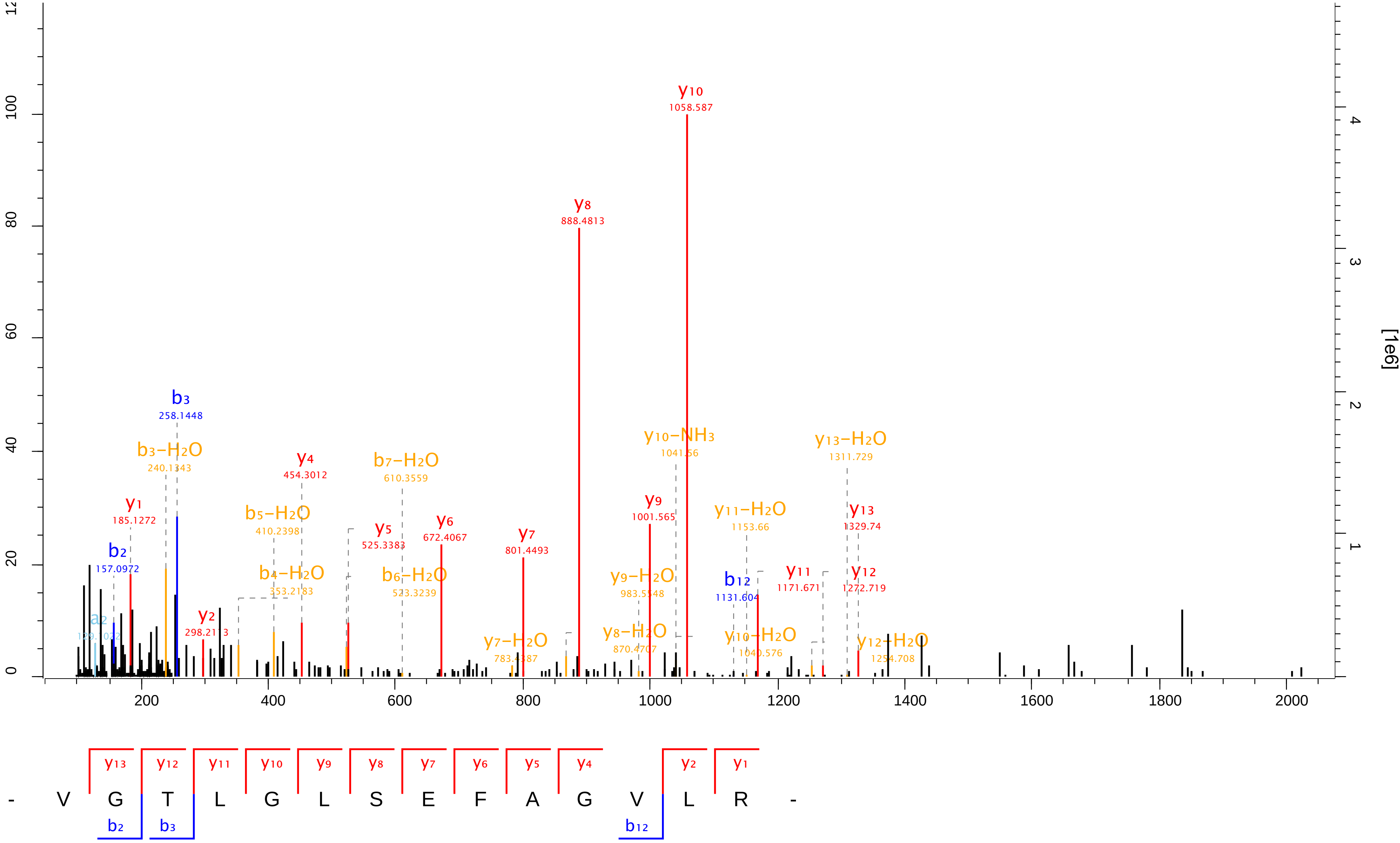

Raw file	Scan	Method	Score	m/z
QEplus003110	12432	FTMS; HCD	87.65	867.49

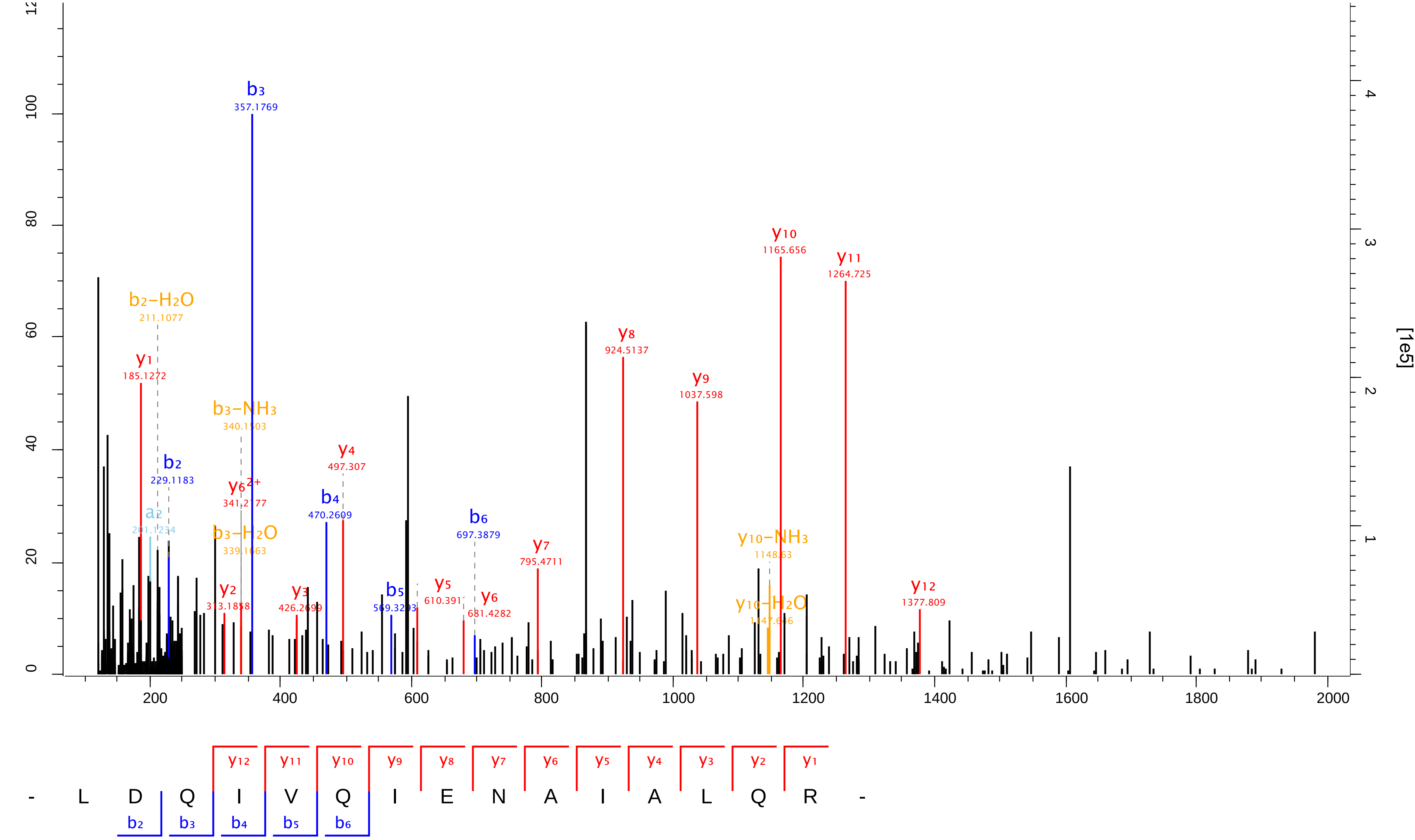

Raw file	Scan	Method	Score	m/z
QEplus003110	12705	FTMS; HCD	77.2	747.93

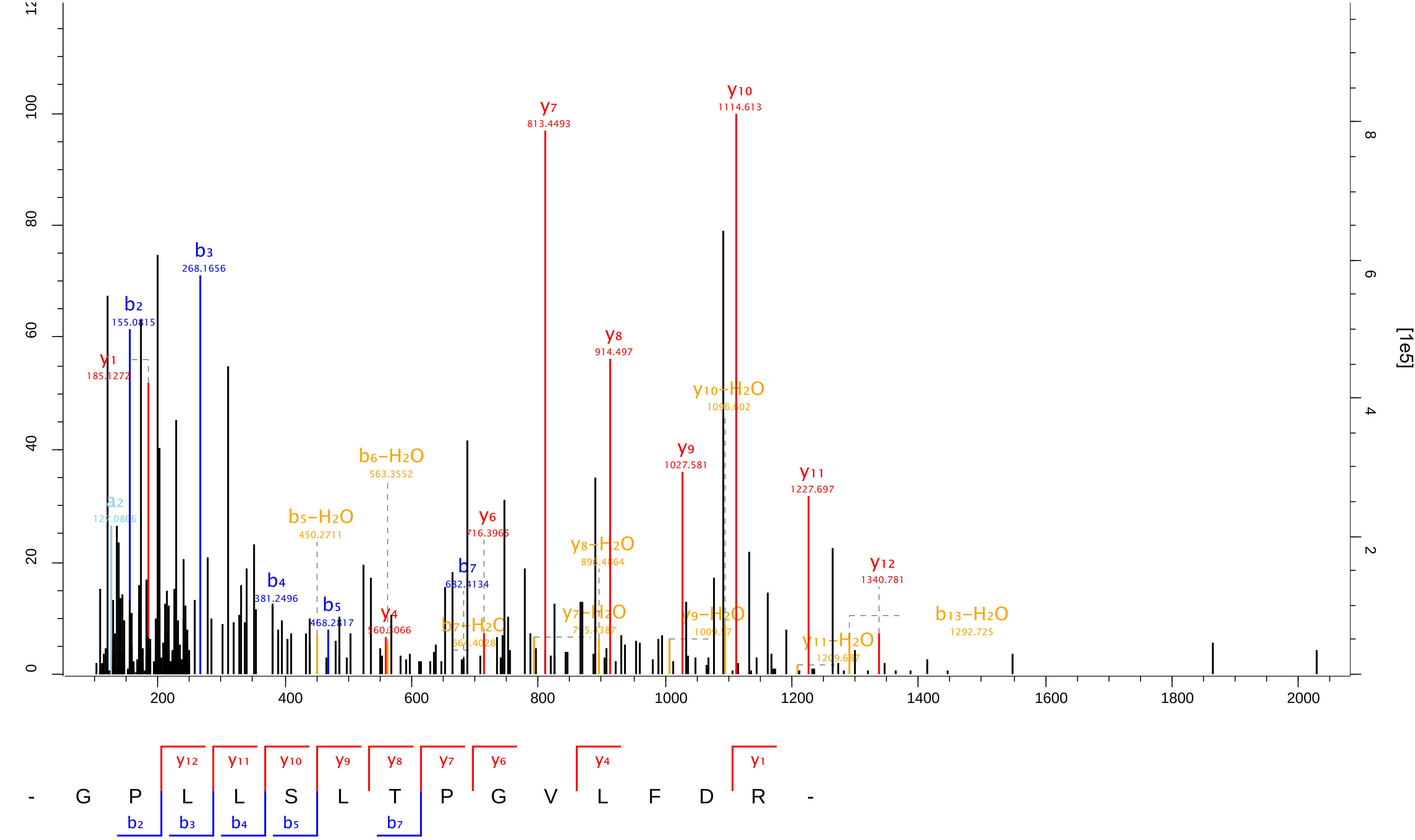

Raw file	Scan	Method	Score	m/z
QEplus003110	12944	FTMS; HCD	64.37	1066.54

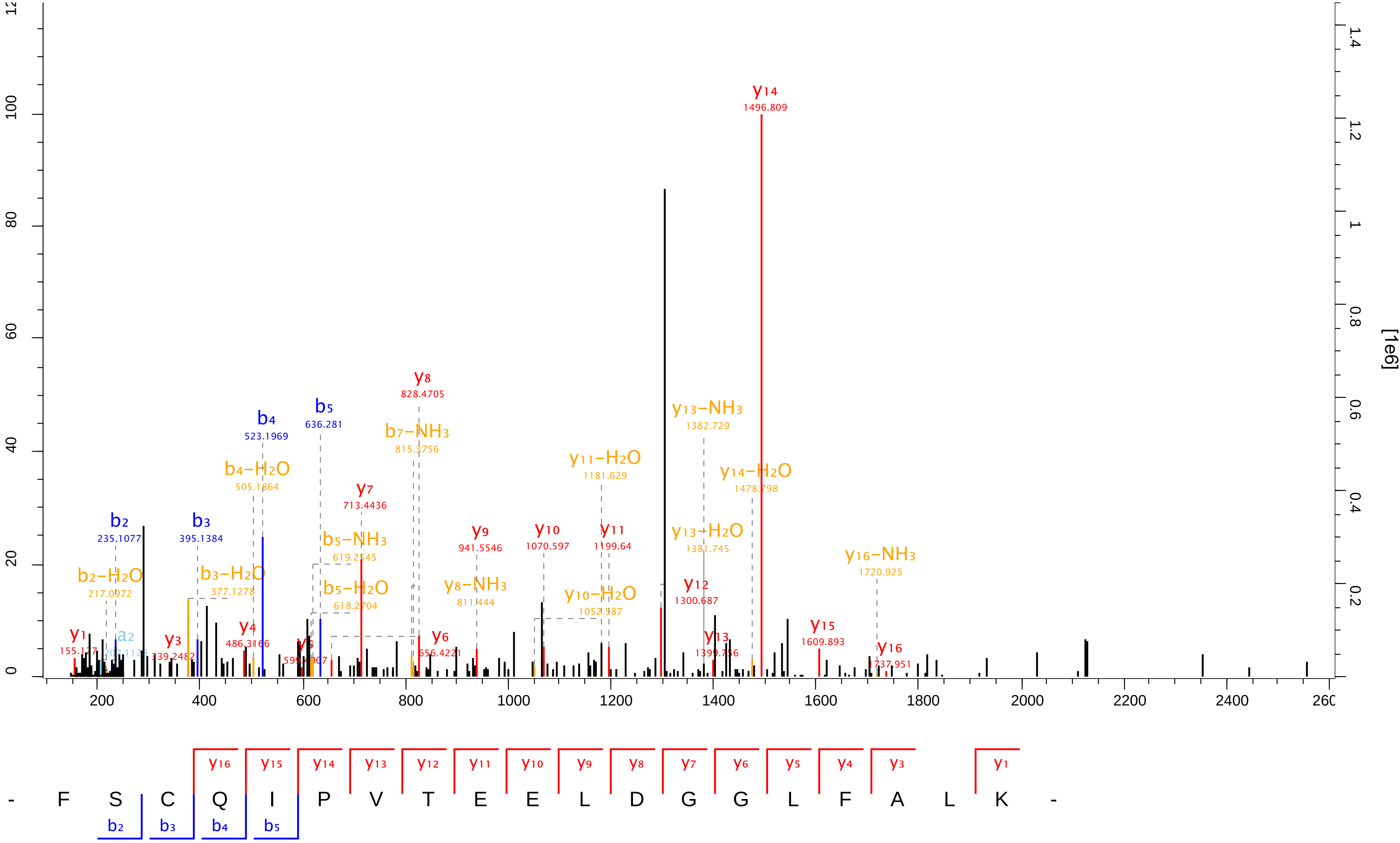

Raw file	Scan	Method	Score	m/z
QEplus003110	13885	FTMS; HCD	223.5	1011.52

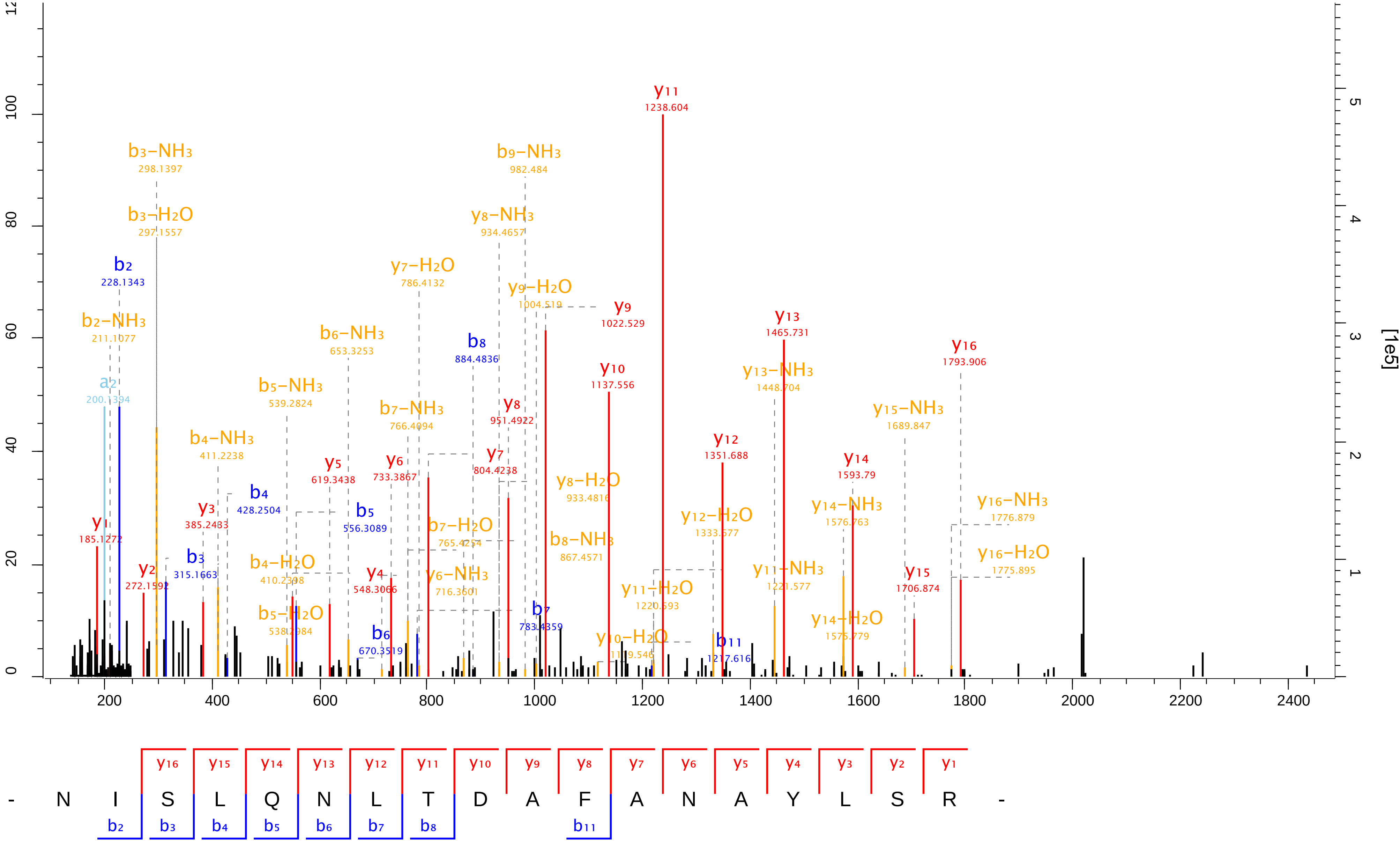

Raw file	Scan	Method	Score	m/z
QEplus003110	6091	FTMS; HCD	101.64	427.27

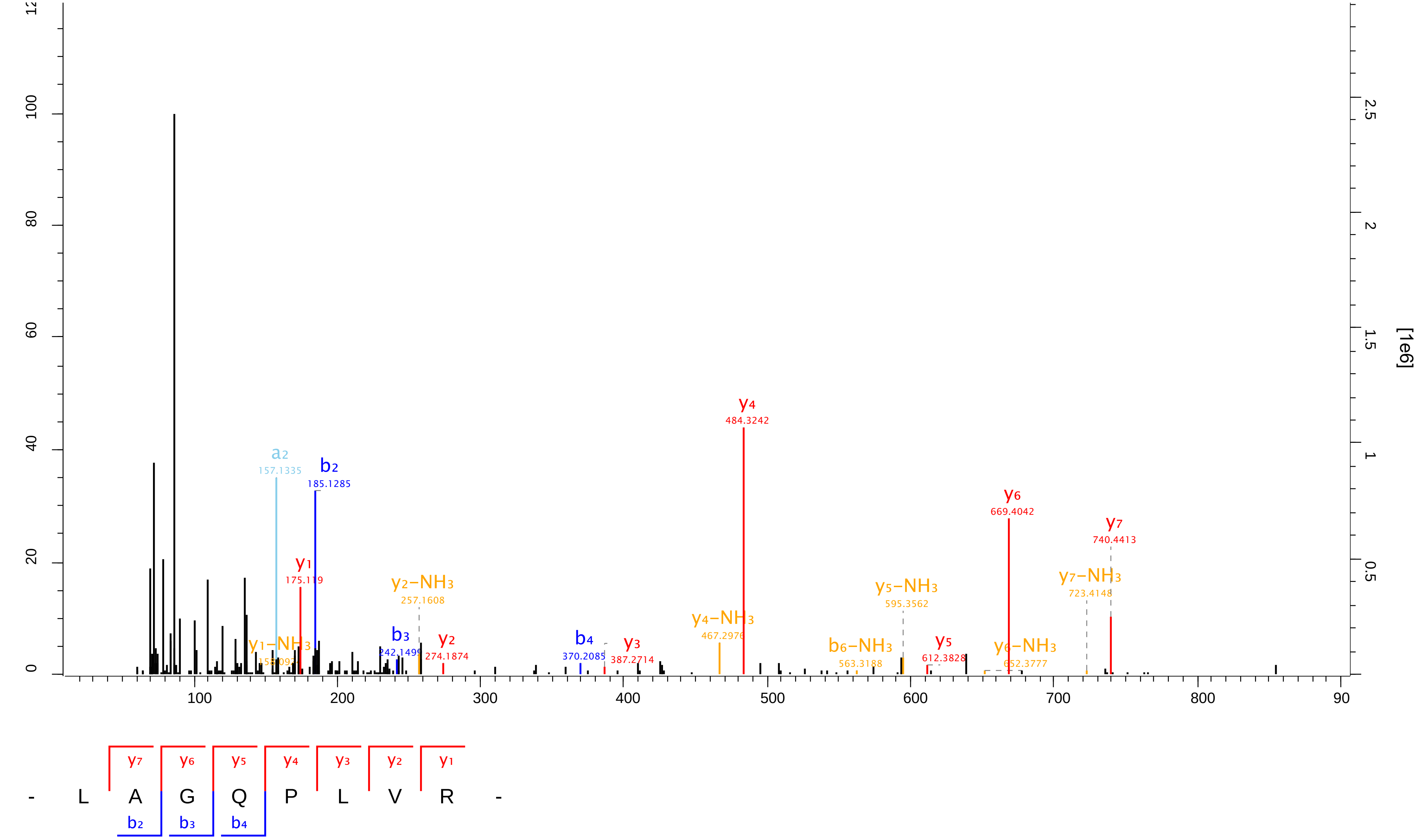

Raw file	Scan	Method	Score	m/z
QEplus003111	13721	FTMS; HCD	189.1	773.95

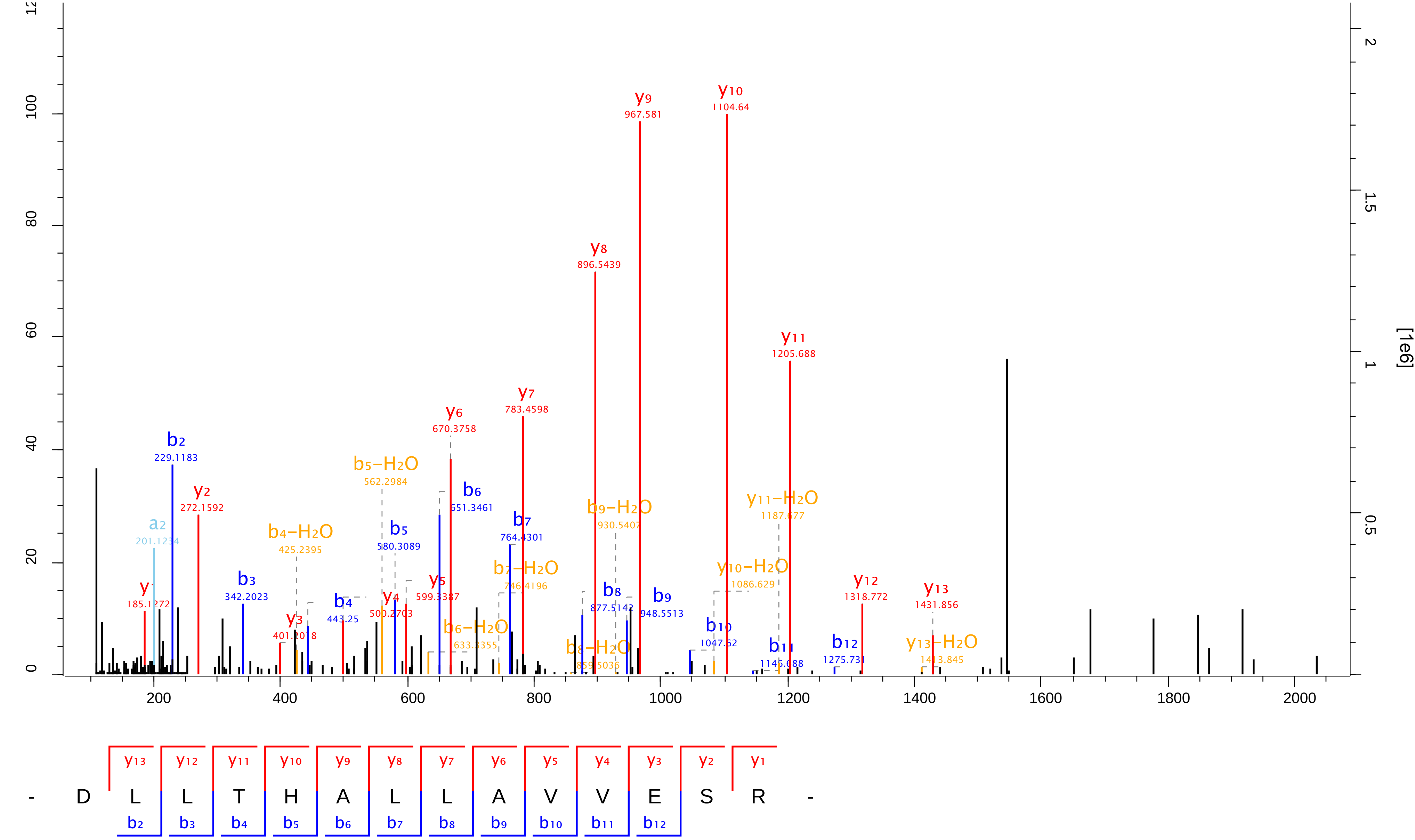

Raw file	Scan	Method	Score	m/z
QEplus003111	5795	FTMS; HCD	72.64	548.8

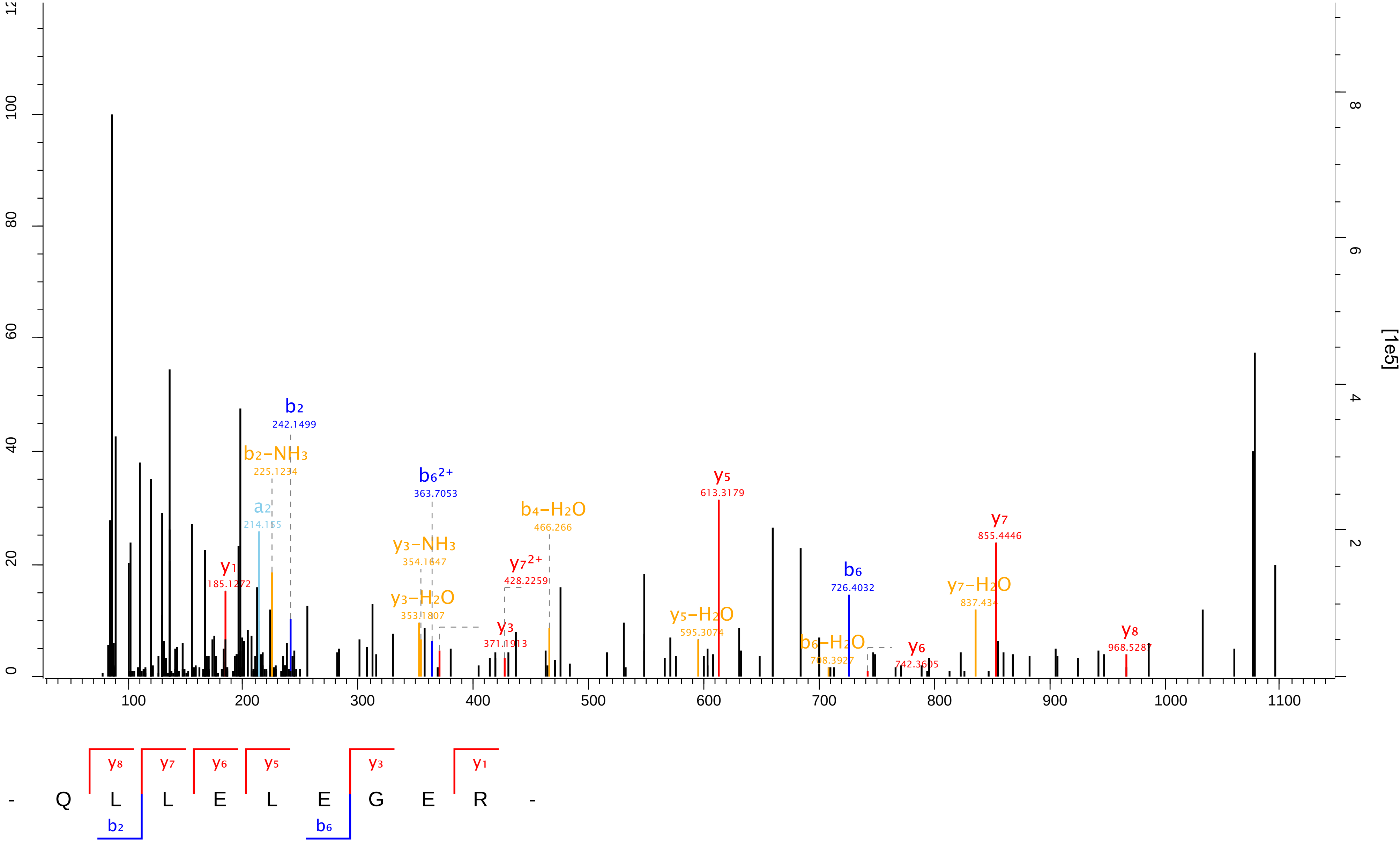

Raw file	Scan	Method	Score	m/z
QEplus003111	6205	FTMS; HCD	76.33	642.86

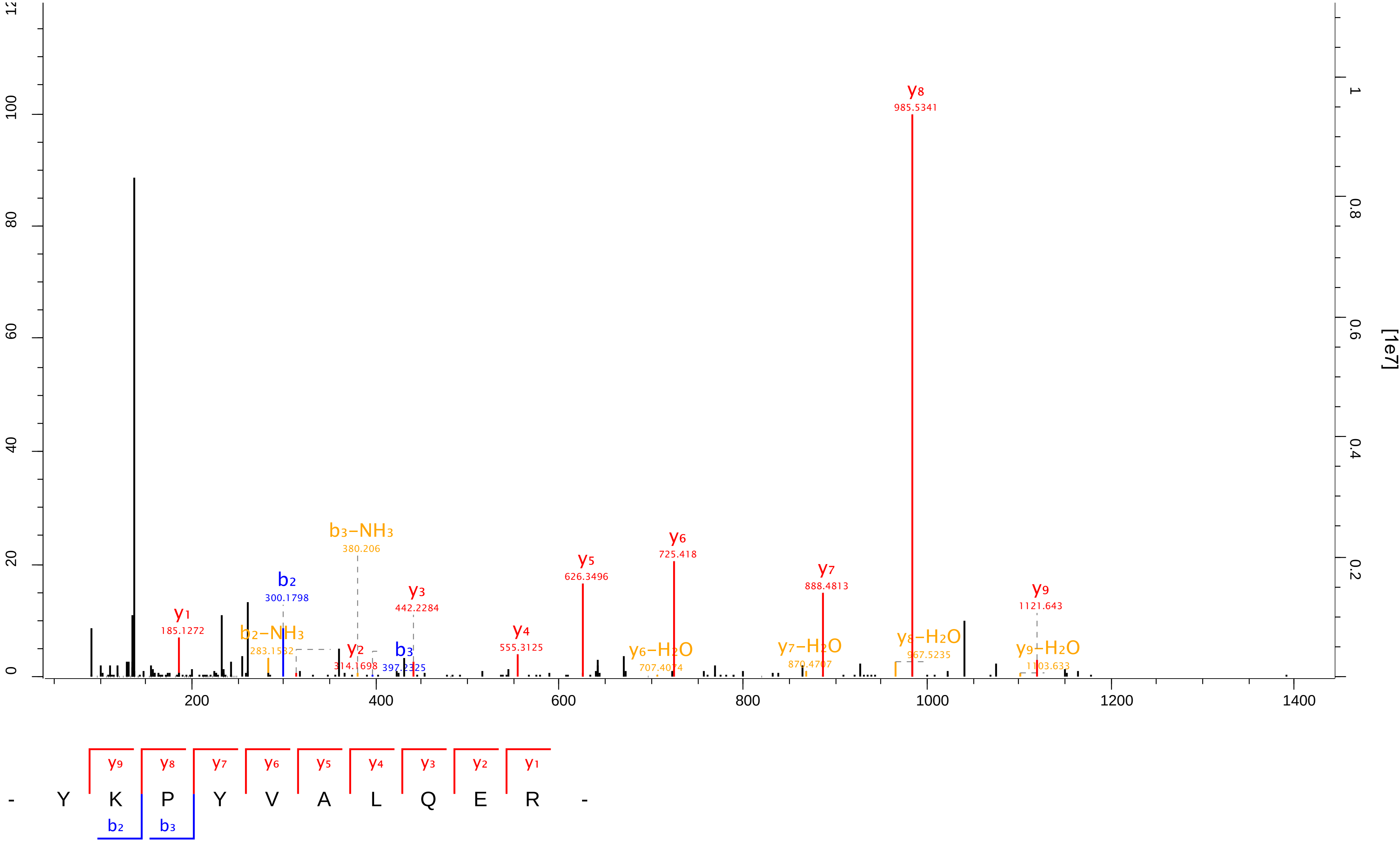

Raw file	Scan	Method	Score	m/z
QEplus003112	10623	FTMS; HCD	53.08	590.34

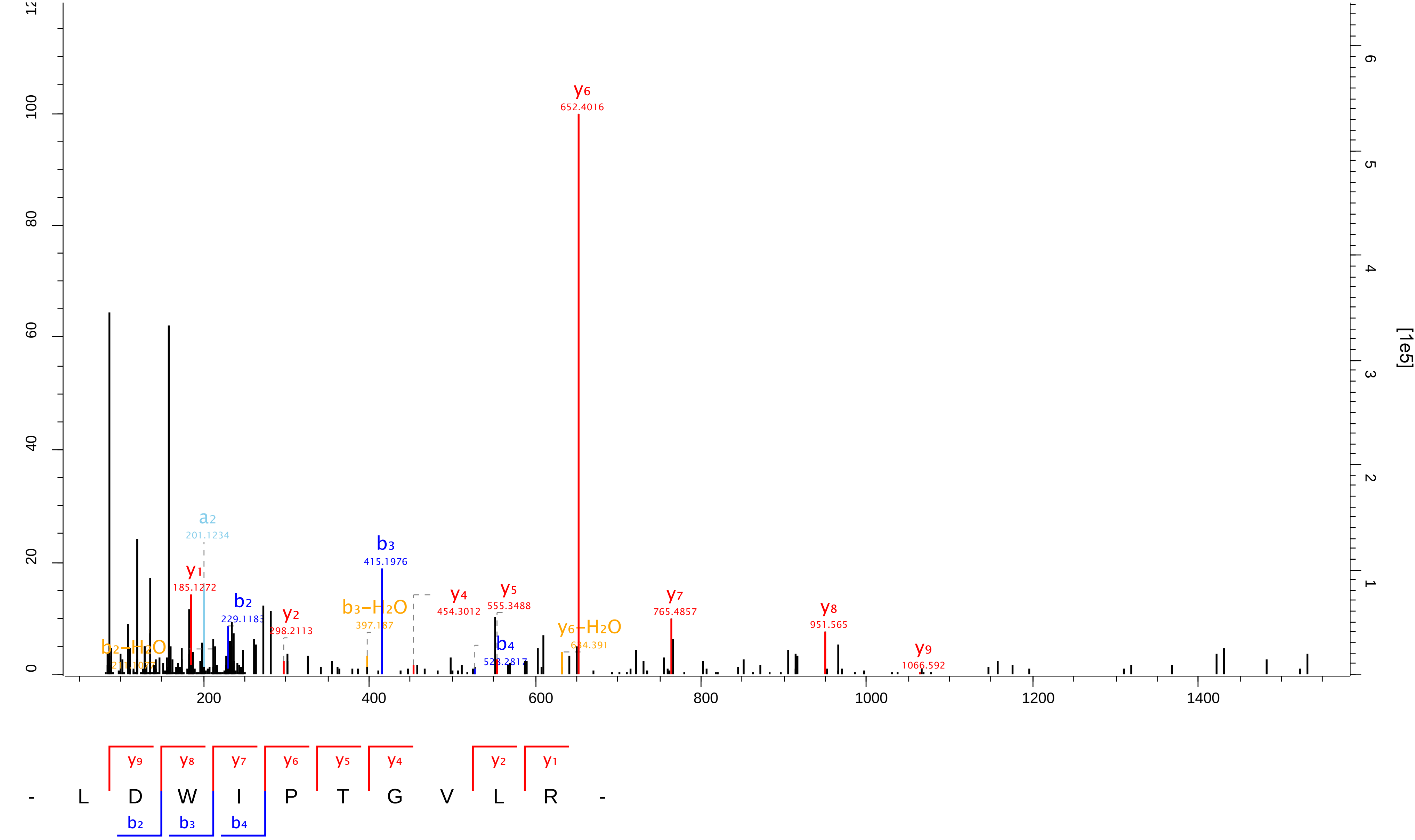

Raw file	Scan	Method	Score	m/z
QEplus003112	10702	FTMS; HCD	61.11	662.87

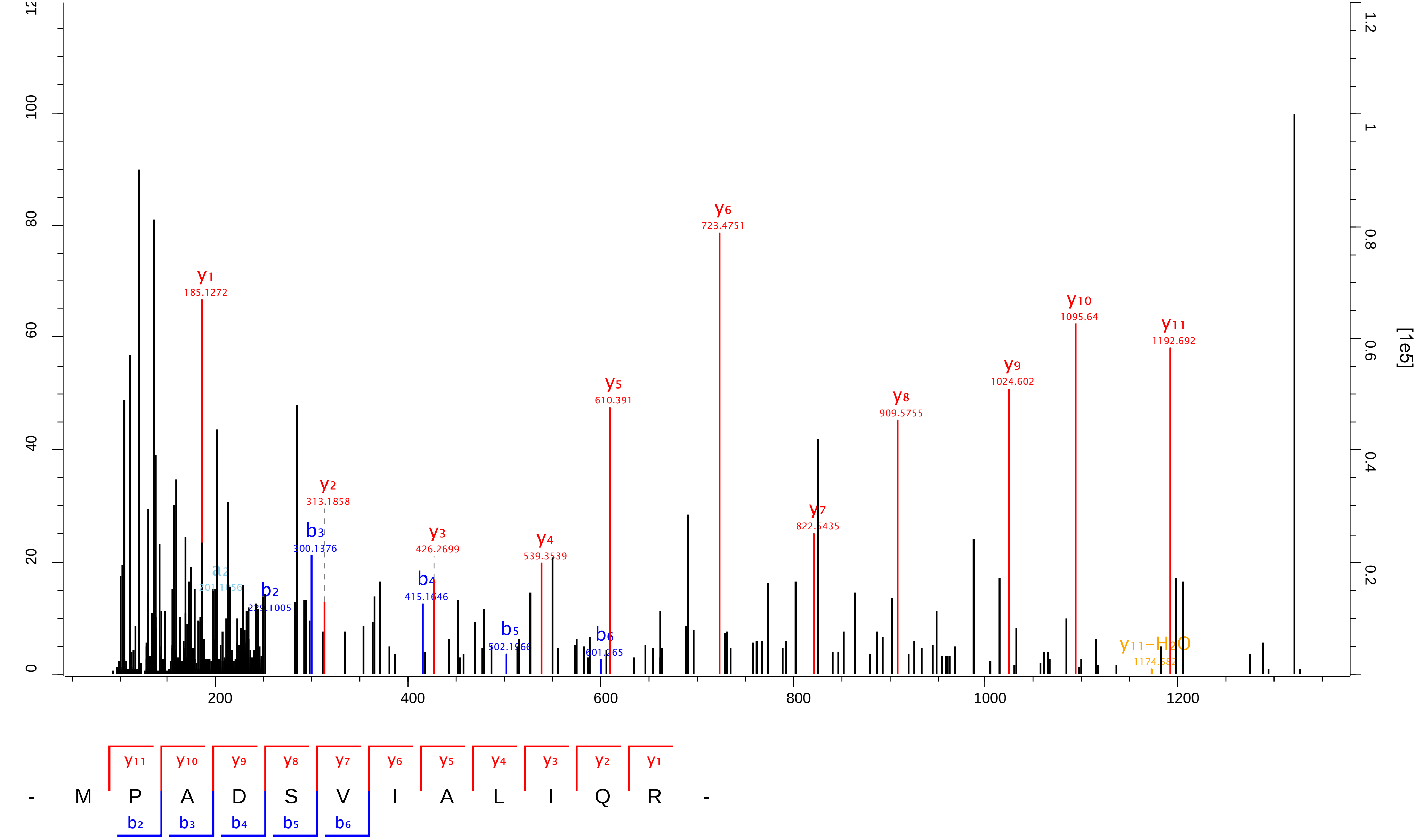

Raw file	Scan	Method	Score	m/z
QEplus003112	13257	FTMS; HCD	126.84	932.03

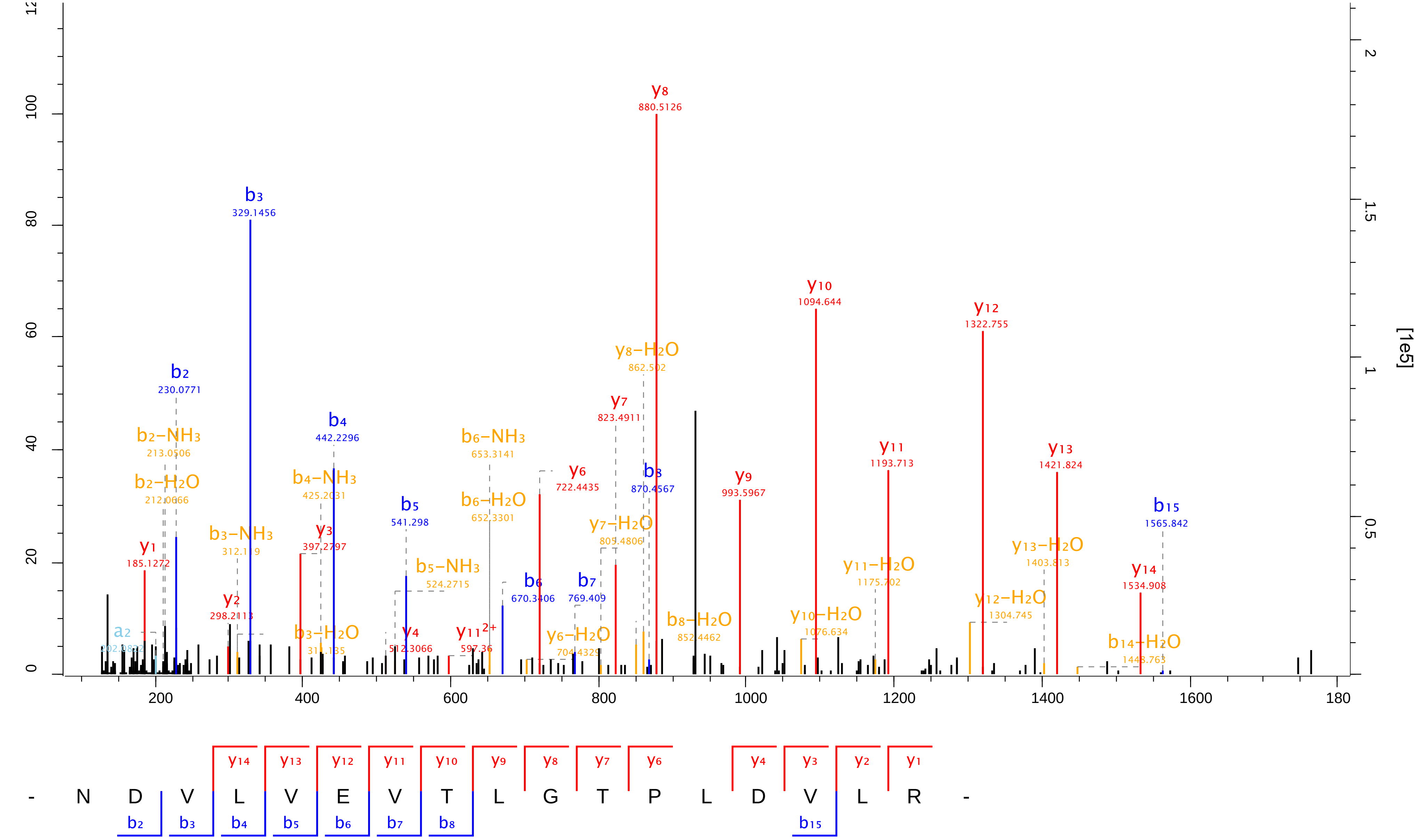

Raw file	Scan	Method	Score	m/z
QEplus003112	13440	FTMS; HCD	51.29	610.34

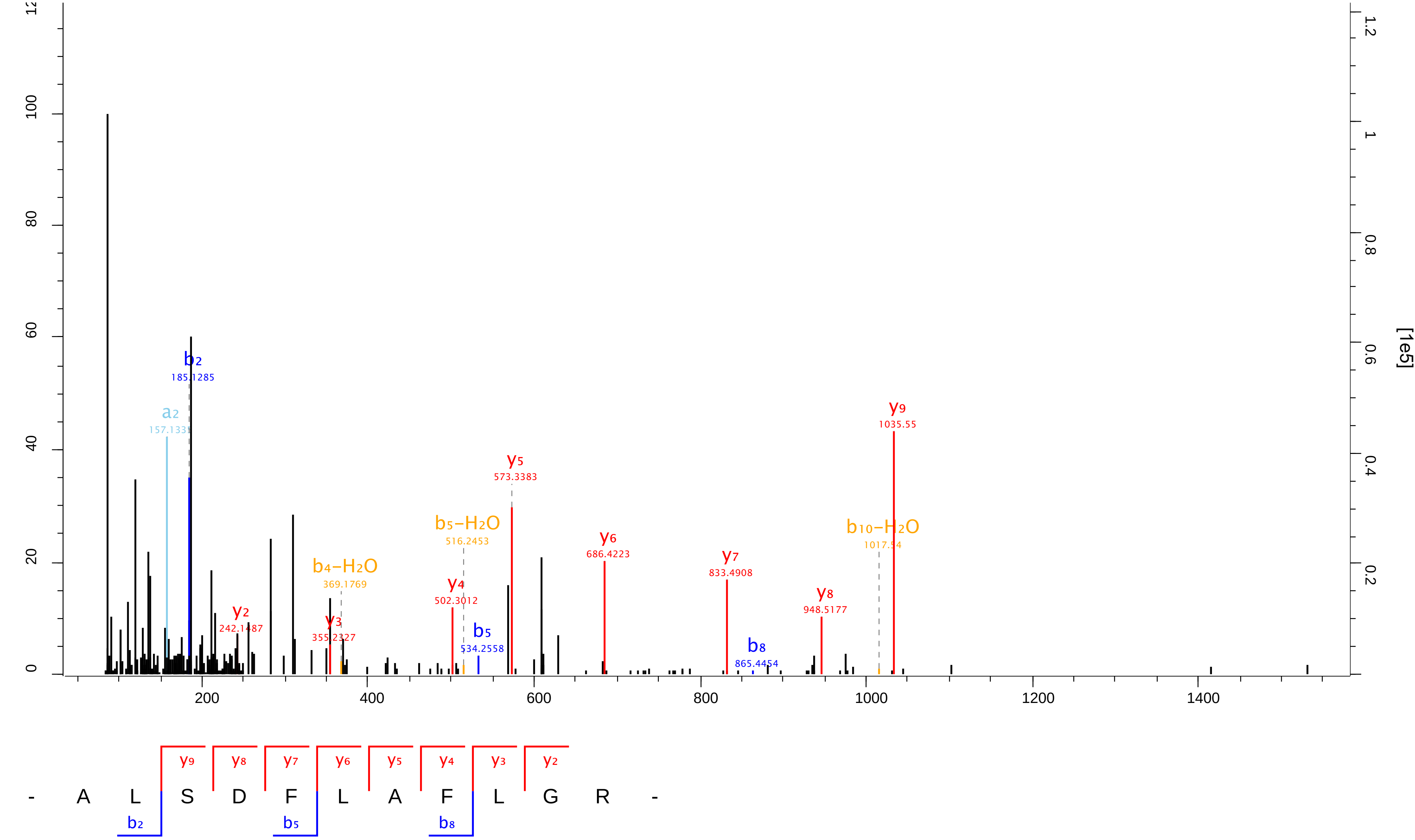

Raw file	Scan	Method	Score	m/z
QEplus003112	4865	FTMS; HCD	96.1	610.31

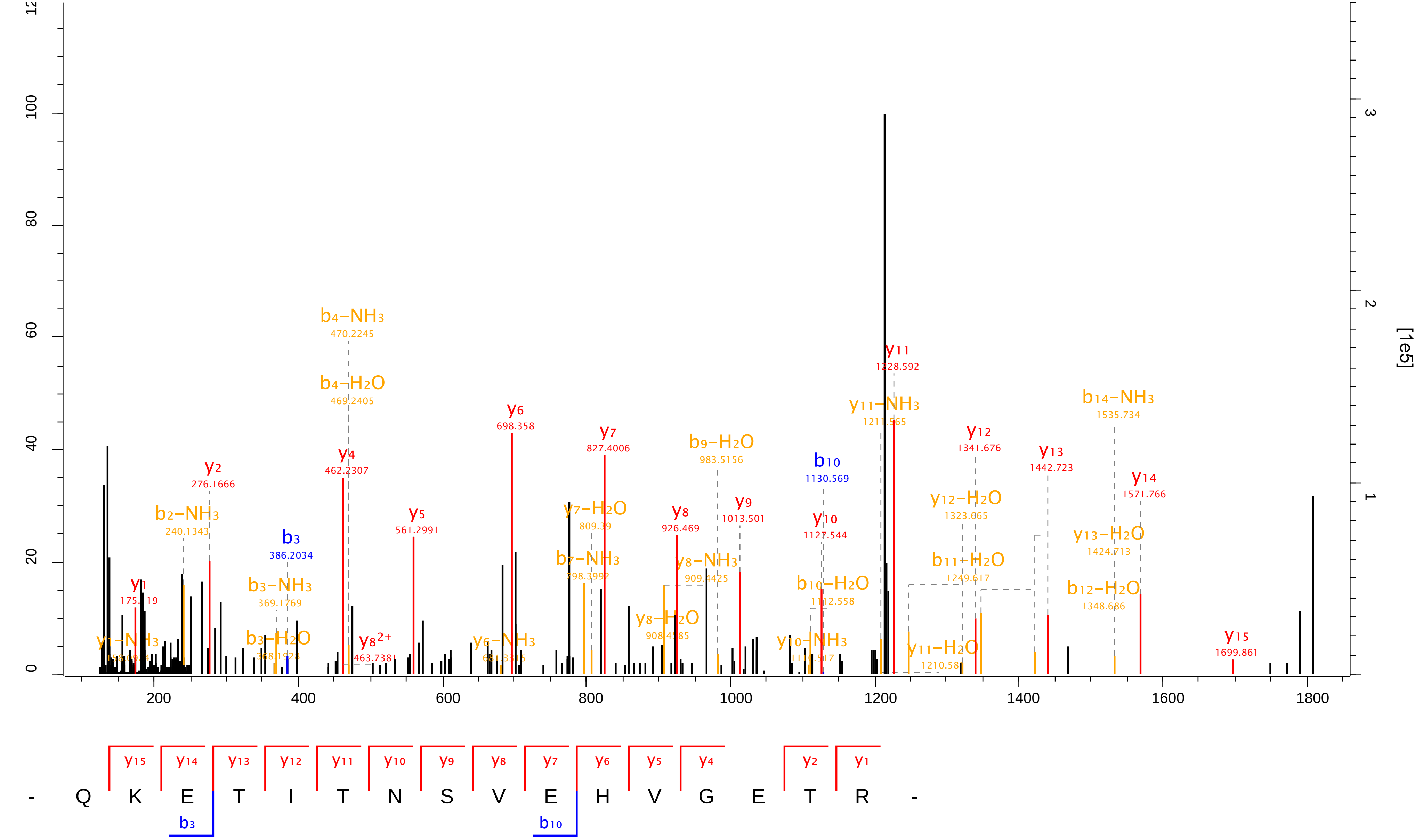

Raw file	Scan	Method	Score	m/z
QEplus003112	5016	FTMS; HCD	67.33	464.76

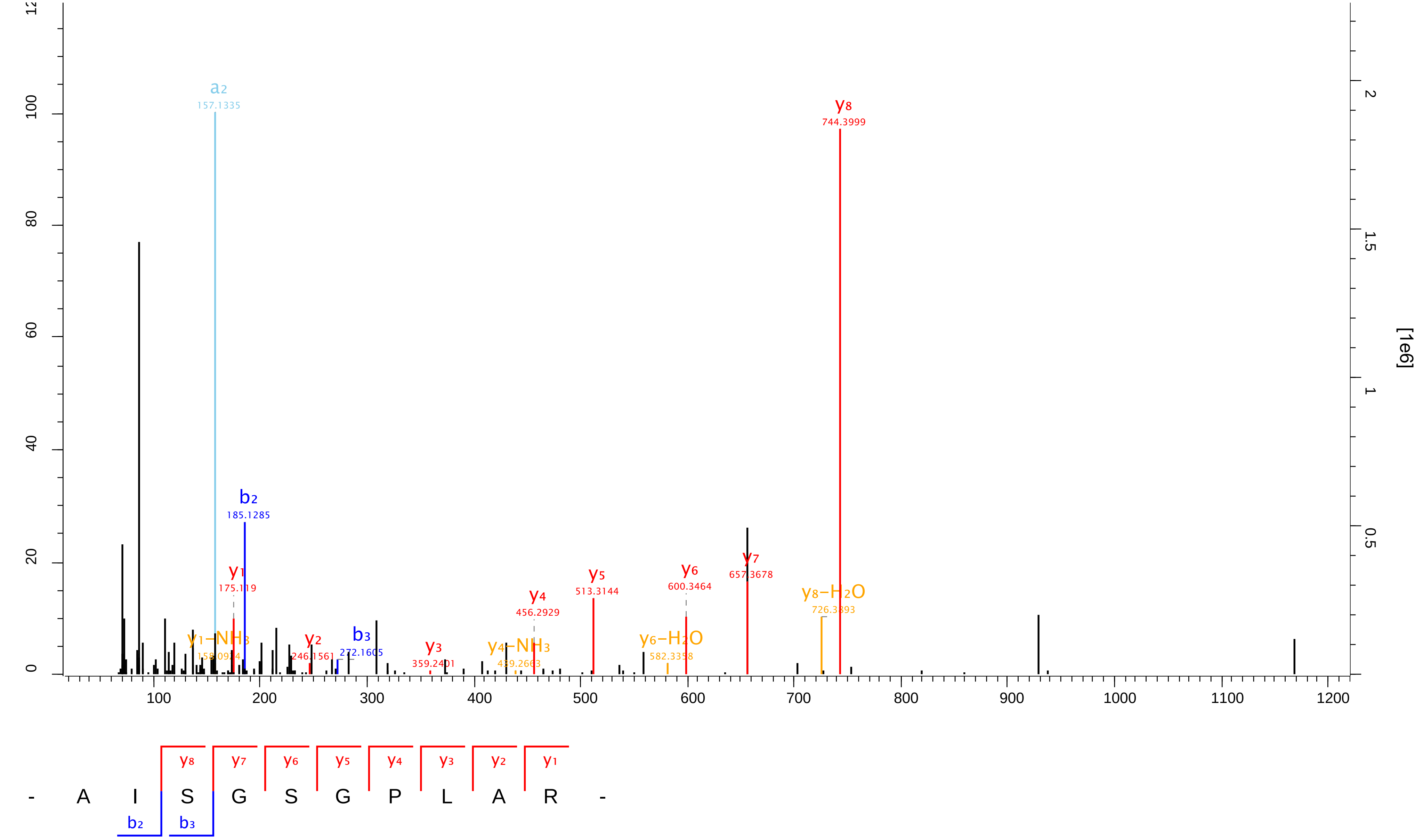

Raw file	Scan	Method	Score	m/z
QEplus003113	13506	FTMS; HCD	157.58	939.99

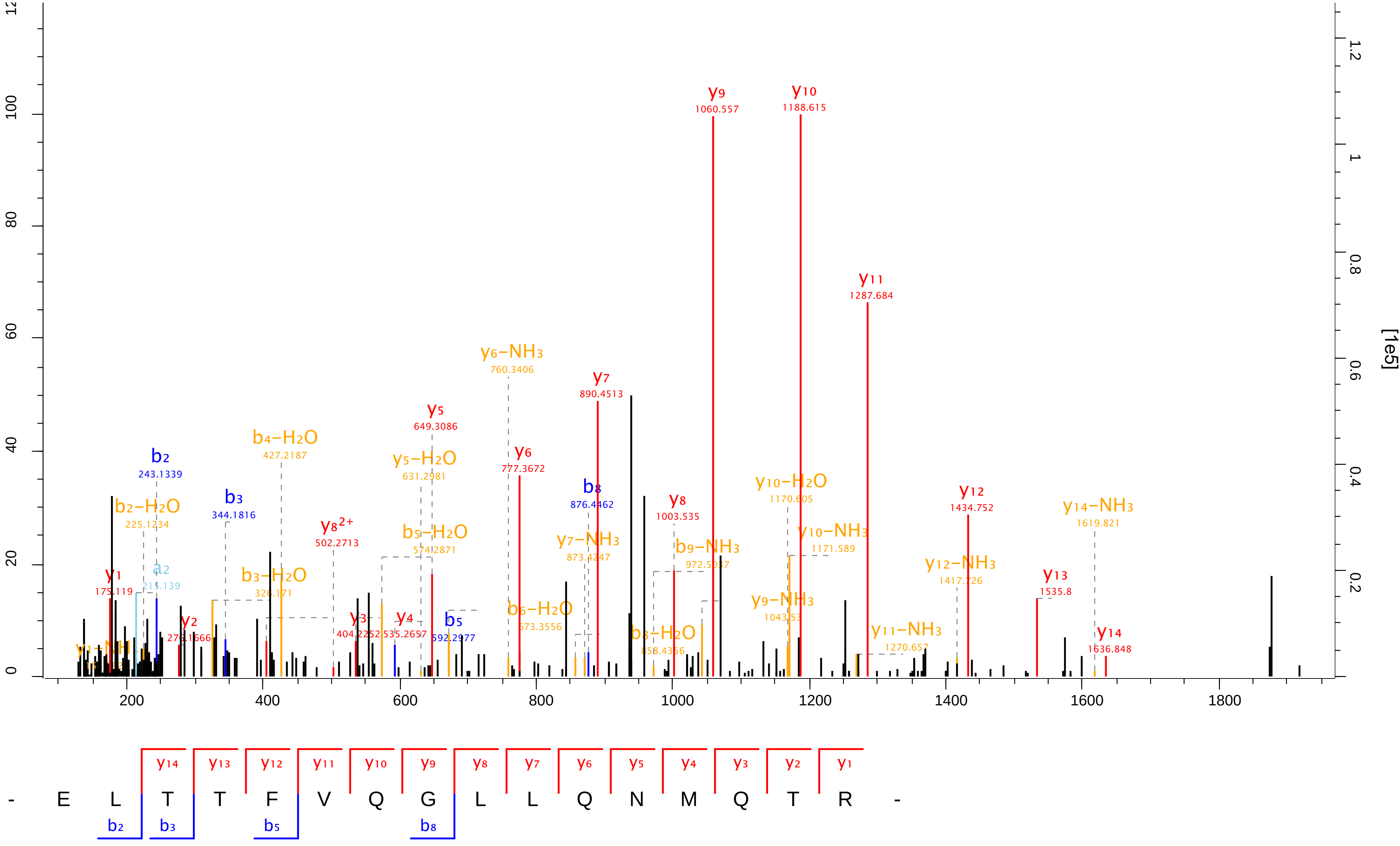

Raw file	Scan	Method	Score	m/z
QEplus003113	7276	FTMS; HCD	52.53	659

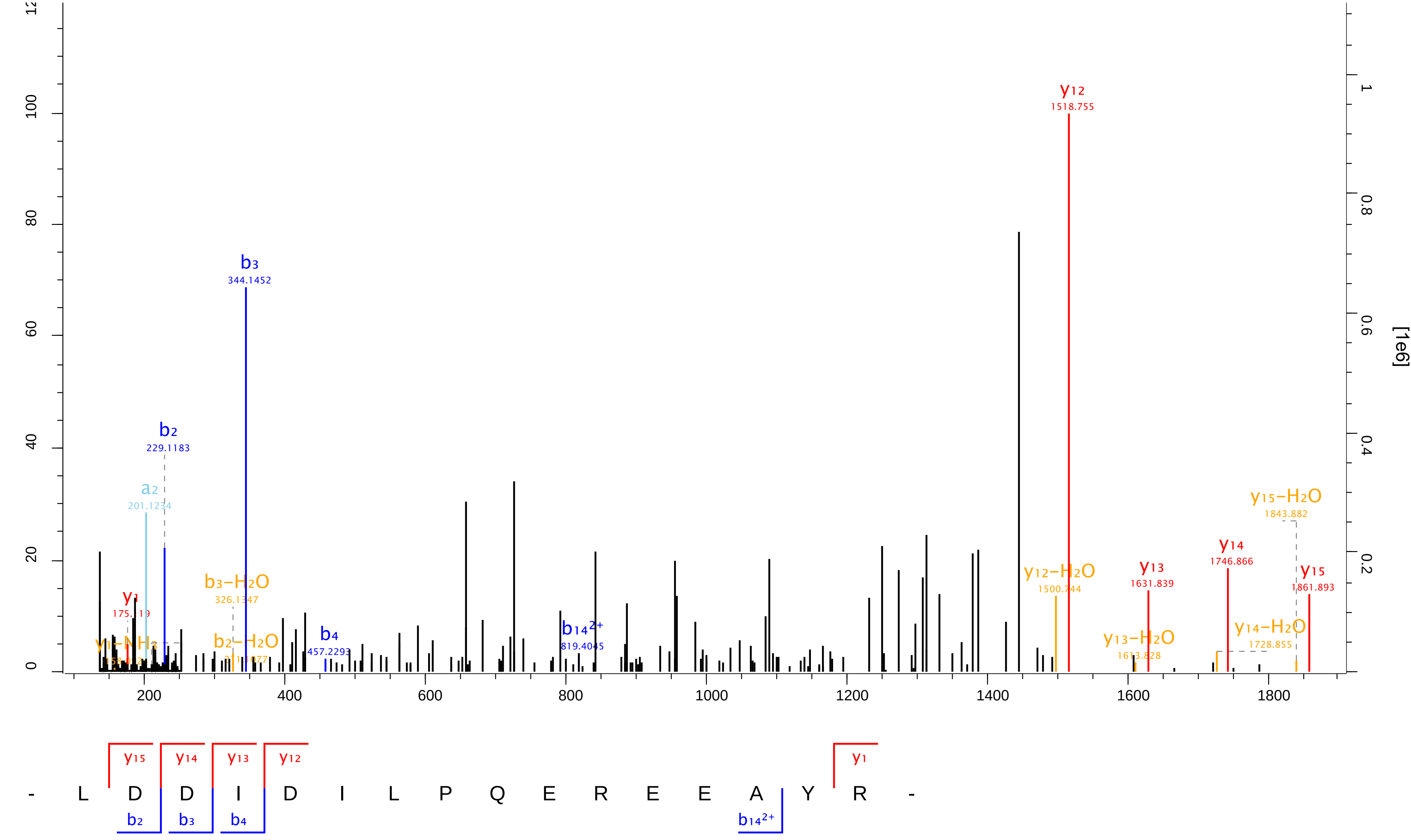

Raw file	Scan	Method	Score	m/z
QEplus003113	8780	FTMS; HCD	92.9	730.4

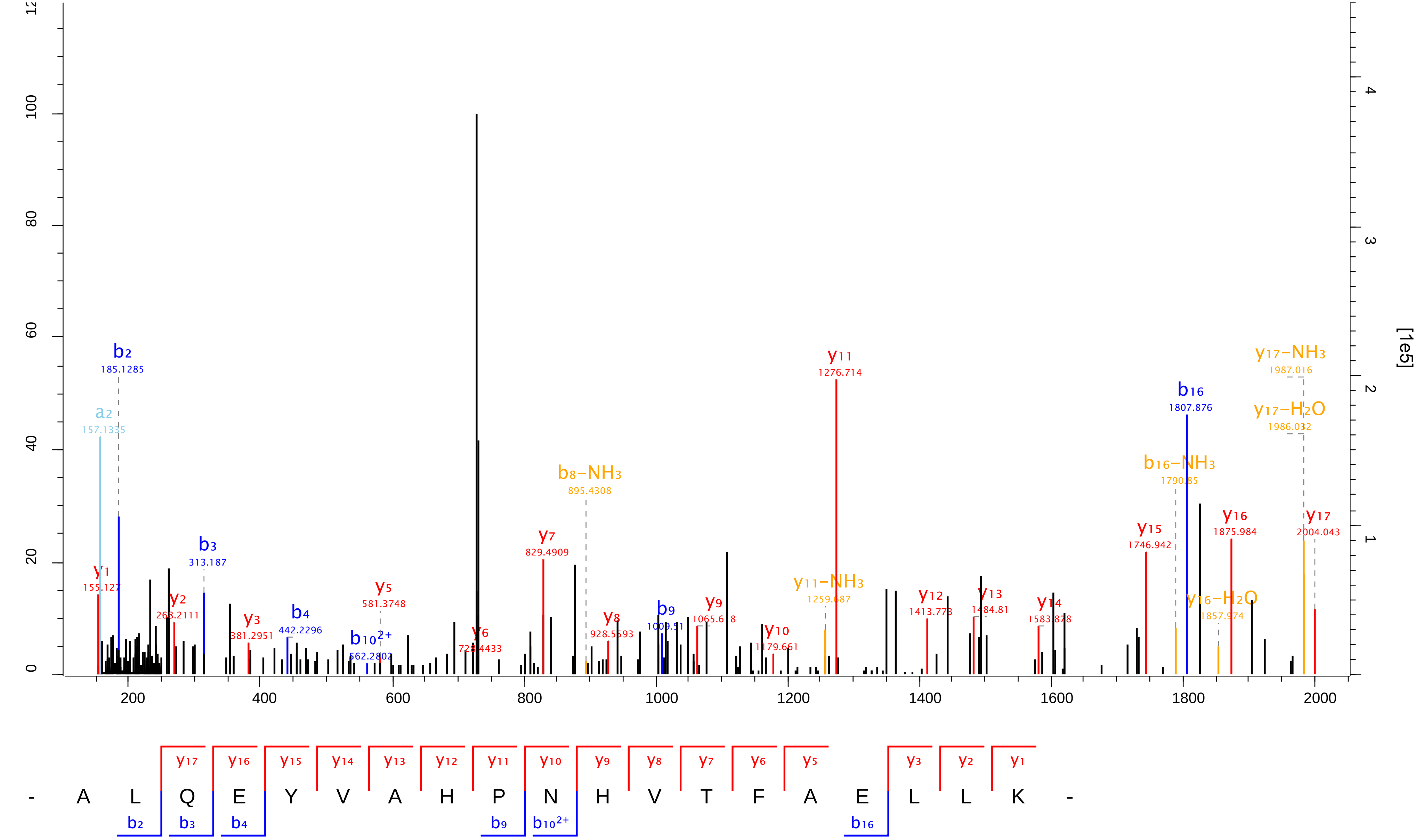

Supplement: Supplementary Data 14 — Annotated MS/MS spectra of peptides used for single-peptide protein identifications in gradient-purified mitochondria of [file ncomms15272-s15.pdf]
